# Social media use and health risk behaviours in young people: systematic review and meta-analysis

**DOI:** 10.1136/bmj-2022-073552

**Published:** 2023-11-29

**Authors:** Amrit Kaur Purba, Rachel M Thomson, Paul M Henery, Anna Pearce, Marion Henderson, S Vittal Katikireddi

**Affiliations:** 1 Medical Research Council/Chief Scientist Office, Social and Public Health Sciences Unit, University of Glasgow, Glasgow, UK; 2Public Health Scotland, Edinburgh, UK; 3School of Social Work and Social Policy, University of Strathclyde, Glasgow, UK

## Abstract

**Objectives:**

To examine the association between social media use and health risk behaviours in adolescents (defined as those 10-19 years).

**Design:**

Systematic review and meta-analysis.

**Data sources:**

EMBASE, Medline, APA PsycINFO, SocINDEX, CINAHL, SSRN, SocArXic, PsyArXiv, medRxiv, and Google Scholar (1 January 1997 to 6 June 2022).

**Methods:**

Health risk behaviours were defined as use of alcohol, drugs, tobacco, electronic nicotine delivery systems, unhealthy dietary behaviour, inadequate physical activity, gambling, and anti-social, sexual risk, and multiple risk behaviours. Included studies reported a social media variable (ie, time spent, frequency of use, exposure to health risk behaviour content, or other social media activities) and one or more relevant outcomes. Screening and risk of bias assessments were completed independently by two reviewers. Synthesis without meta-analysis based on effect direction and random-effects meta-analyses was used. Effect modification was explored using meta-regression and stratification. Certainty of evidence was assessed using GRADE (Grading of Recommendations, Assessment, Development and Evaluations).

**Results:**

Of 17 077 studies screened, 126 were included (73 included in meta-analyses). The final sample included 1 431 534 adolescents (mean age 15.0 years). Synthesis without meta-analysis indicated harmful associations between social media and all health risk behaviours in most included studies, except inadequate physical activity where beneficial associations were reported in 63.6% of studies. Frequent (*v* infrequent) social media use was associated with increased alcohol consumption (odds ratio 1.48 (95% confidence interval 1.35 to 1.62); n=383 068), drug use (1.28 (1.05 to 1.56); n=117 646), tobacco use (1.85, 1.49 to 2.30; n=424 326), sexual risk behaviours (1.77 (1.48 to 2.12); n=47 280), anti-social behaviour (1.73 (1.44 to 2.06); n=54 993), multiple risk behaviours (1.75 (1.30 to 2.35); n=43 571), and gambling (2.84 (2.04 to 3.97); n=26 537). Exposure to content showcasing health risk behaviours on social media (*v* no exposure) was associated with increased odds of use of electronic nicotine delivery systems (1.73 (1.34 to 2.23); n=721 322), unhealthy dietary behaviours (2.48 (2.08 to 2.97); n=9892), and alcohol consumption (2.43 (1.25 to 4.71); n=14 731). For alcohol consumption, stronger associations were identified for exposure to user generated content (3.21 (2.37 to 4.33)) versus marketer generated content (2.12 (1.06 to 4.24)). For time spent on social media, use for at least 2 h per day (*v* <2 h) increased odds of alcohol consumption (2.12 (1.53 to 2.95); n=12 390). GRADE certainty was moderate for unhealthy dietary behaviour, low for alcohol use, and very low for other investigated outcomes.

**Conclusions:**

Social media use is associated with adverse health risk behaviours in young people, but further high quality research is needed to establish causality, understand effects on health inequalities, and determine which aspects of social media are most harmful.

**Study registration:**

PROSPERO, CRD42020179766.

## Introduction

Social media has revolutionised the communication landscape, with approximately 139 million adolescents (defined here as 10-19 year olds) using Instagram and 120.2 million using Facebook globally in 2022.^1^ ^2^ Social media is defined as websites and applications that host numerous user activities, for example, creation and sharing of content, social networking, and microblogging. Its diverse and inherently social nature has supported adolescents’ need for autonomy, social connectedness, and relatedness.^3^ ^4^ ^5^ ^6^ Recognised by the World Health Organization as a powerful medium to promote health, the use of social media to elicit positive behaviour change is well documented, including increased physical activity and healthy diets, increased accessibility to health information, and peer, social, and emotional support.^7^ ^8^ ^9^

**Fig 1:**
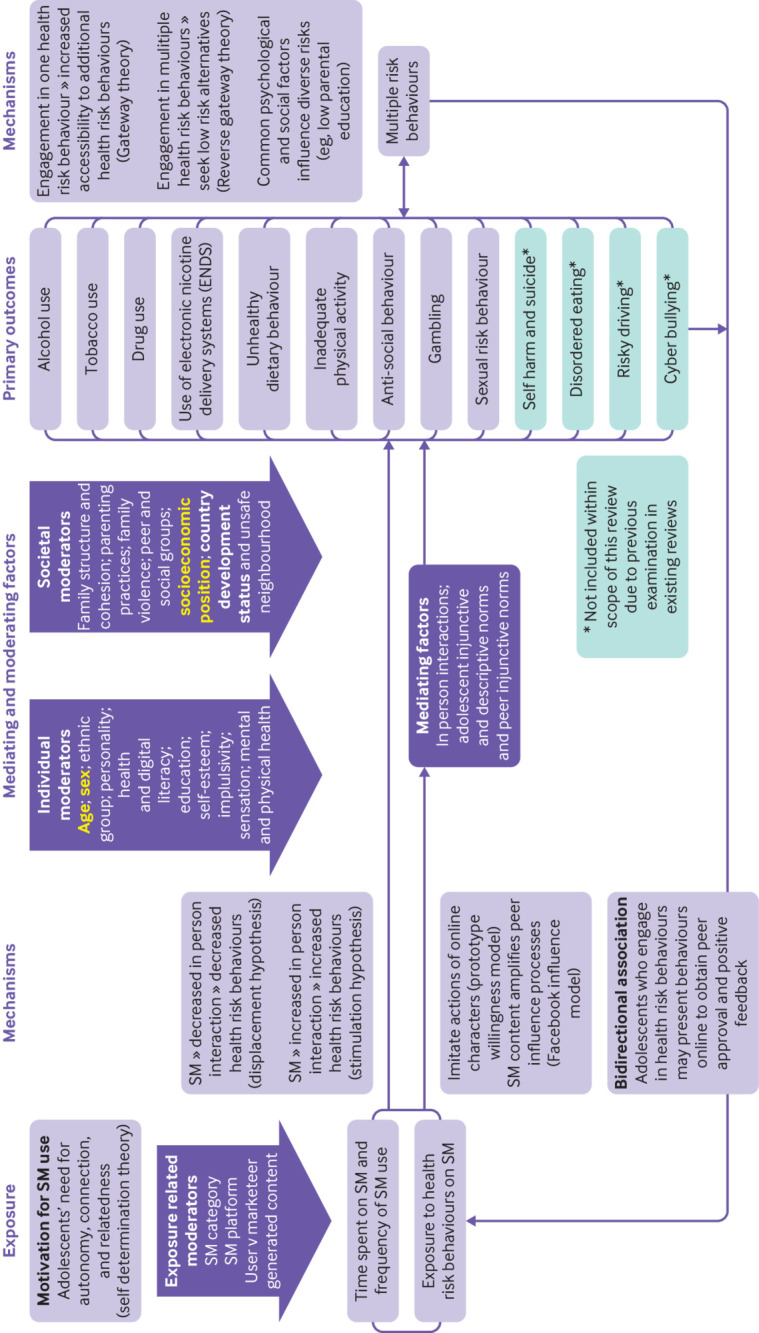
Logic model illustrating the pathways between social media and health risk behaviours in adolescents. Variables considered important potential confounders in this study are indicated in yellow bold and were selected a priori by the researchers' following expert advisory group consultation and identification of variables considered key confounders in the literature. Variables deemed potential effect modifiers for exploration in this study are indicated in bold. SM=social media

Despite its ubiquitous use and potential benefits, harmful effects on health risk behaviours of adolescents (eg, substance use and risky sexual behaviour) are possible, at least partly due to aggravated peer pressure and social norms.^3^ ^10^ ^11^ Numerous pathways may exist between social media and health risk behaviours (fig 1). Social media use might displace more traditional in-person interactions, thereby increasing physical inactivity. Marketer generated (eg, advertisements and influencers)^12^ ^13^ ^14^ ^15^ ^16^ and user generated (eg, user and peer posts) content can display consumption of unhealthy commodities.^17^ ^18^ Exposure to such content from traditional media (eg, film and television) has been shown to affect health risk behaviours in adolescents (eg, substance use and an unhealthy diet),^19^ ^20^ with experimental and longitudinal research suggesting online content also influences behaviours offline.^21^ ^22^ ^23^ ^24^ ^25^ ^26^

Adolescence is a period that denotes adoption of lifelong behaviours—health consequences are therefore potentially immediate and lifelong.^27^ ^28^ ^29^ Immediate consequences include (but are not limited to) alcohol and drug related injury, low educational attainment and depression (for alcohol and drug use), and sexually transmitted diseases and teenage pregnancy (for sexual risk behaviour).^30^ Yet, these represent relatively extreme outcomes and for most adolescents these behaviours, if experimental and short lived, will have limited harms and can be considered a normal part of adolescent development. However, some health behaviours, such as poor diet, inadequate physical activity, and alcohol consumption, can be set in adolescence and carry lifelong consequences.^27^ ^28^ ^29^ Anti-social behaviour is associated with adverse consequences such as criminality and psychosocial malfunctioning, of which the long term effects extend to causing substantial distress for others, emphasising the public health relevance of this risk behaviour.^31^

Existing reviews focus on university and college populations (and are therefore not representative of all adolescents); assess social media under the broad scope of digital media and internet use; do not assess risk of bias; and examine few health risk behaviours (ie, substance use and sexual risk behaviour).^20^ ^32^ ^33^ ^34^ ^35^ ^36^ Differential effects by socioeconomic position, specifically whether more disadvantaged groups are more susceptible to harm from social media, consequently resulting in a widening of health inequalities, and those between high and low middle income countries have also not been explored.^23^ ^37^ ^38^ ^39^ Prior research investigating social media’s effect on adolescent mental health suggests age and sex differences, in which greater negative effects exist for female and younger adolescents (compared with male and older adolescents). However, these potential differences are yet to be examined in relation to health risk behaviours.^40^ ^41^ Vannucci and colleagues explored the association between social media and adolescent substance use and risky sexual behaviour.^42^ The review’s synthesis of electronic media use (defined as electronic media with a direct component involving social interactions with others (2022 personal communication with A Vannucci)) with social media and reliance on pooled correlations inhibits any explicit conclusions about the size of associations resulting from social media use specifically. Due to the high risk of confounding and reverse causation in studies in this area (which largely rely on observational data), assessment of the quality of evidence is important—an area that has been limited in other reviews.^32^ ^42^

We aimed to systematically review the evidence on social media use and adolescent health risk behaviours using five objectives. Firstly, we wanted to explore how social media use is measured in studies examining its relationship with adolescent health risk behaviours (ie,alcohol, drug, tobacco, electronic nicotine delivery system use, unhealthy dietary behaviour, inadequate physical activity, gambling, anti-social behaviour, sexual risk behaviour, and multiple risk behaviours). Secondly, we wanted to investigate the association between time spent on social media and frequency of use on adolescent health-risk behaviours. Thirdly, we aimed to explore the association between exposure to health risk behaviour content displayed on social media and adolescent health risk behaviours, and if any relationship differs by content viewed (user or marketer generated). Fourthly, we wanted to investigate if any relationships differ by social media platform/category used, age, sex, socioeconomic position, and development status of study setting. Finally, we wanted to evaluate the certainty of evidence using Grading of Recommendations Assessment, Development, and Evaluation (GRADE).

## Methods

We followed the Preferred Reporting Items for Systematic Reviews and Meta-Analyses (PRISMA) and Synthesis Without Meta-analysis (SWiM) reporting guidance.^43^ ^44^ We published a prespecified protocol, including a logic model (fig 1; further background in protocol^45^ ^46^) that was used to identify important confounders and effect modifiers. This study is registered with PROSPERO, CRD42020179766.^45^ Protocol deviations are reported in appendix 1.

### Search methods for identification of studies

EMBASE, Medline, APA PsycINFO, SocINDEX, CINAHL, SSRN, SocArXic, PsyArXiv, and medRxiv were searched from 1 January 1997 (first recognisable social media site “Six Degrees” launched) to 6 June 2022, using a comprehensive strategy developed with an information scientist (appendix 2). We scrutinised the first 30 hits in Google Scholar, screened reference lists of included studies and relevant systematic reviews, and contacted subject experts to identify additional, planned, ongoing, or unpublished studies. Filters for study types and geographical location or language limits were not applied.^47^ We were not able to translate non-English language studies; these studies are reported in appendix 3.^47^

### Study inclusion and exclusion criteria

The precise age range that adolescence encompasses is debated. Following the World Health Organization’s definition,^48^ ^49^ our population of interest was adolescents inclusive, defined as those aged 10-19 years. Studies focusing on college or university participants (of all ages) were excluded due to the differing nature of social media use and health risk behaviours in these groups. Studies including some participants who were not at college or university alongside participants who were at these institutions were included if relevant data for participants who were not at college or university participants could be extracted.^13^ ^22^ ^50^ The exposure of interest was use of any social media category in the SAGE social media categorisation^51^ (social networking, microblogging, media sharing, geographical location based, bookmarking, social news, collaborative authoring sites, web conferencing, and scheduling and meeting; appendix 4). Online (social) gambling (eg, simulated gambling via Facebook) and online (social) gaming were eligible due to their inclusion of core social media functionalities, namely user interaction.^52^ ^53^ ^54^ Dating platforms on social media were excluded because most are restricted to users 18 years and over.^55^ ^56^ ^57^

Social media variables were classified into time spent (eg, hours per day), frequency of use (eg, daily, weekly, or general use), exposure to content displaying health risk behaviour (eg, alcohol advertising on Facebook), and other social media activities (eg, strategies to manage online presence). The process used to classify the social media category, platform, and type of health risk behaviour content (user generated or marketer generated) of reported social media variables is provided in appendix 4.

The comparator group was individuals with no or differing levels of time spent, frequency of use, or variable.

Outcome selection was guided by preliminary evidence,^58^ the logic model (fig 1), and an advisory group (appendix 5).^59^ Eligible outcomes were alcohol, drug, tobacco, electronic nicotine delivery systems use, sexual risk behaviour, gambling (not via social media, eg, lottery, scratch cards), unhealthy dietary behaviour, inadequate physical activity, anti-social behaviour, and multiple risk behaviours (at least two of the aforementioned behaviours) (appendix 6).

We deemed studies reporting quantitative data from primary research eligible.

### Selection of studies

Records were de-duplicated in Mendeley^60^ and imported to Covidence^61^ for screening. Eligibility criteria were piloted on 100 studies and all titles and abstracts and full texts were independently screened by AKP and a second reviewer (PMH, RT, AP, or MH), with conflicts resolved via consensus or discussion with a third reviewer (SVK). Where eligible studies contained overlapping or duplicate data, a set of decision rules (appendix 7) considered alignment with our population, exposure, comparator, and outcome criteria to select unique data for synthesis.

### Data extraction and risk of bias assessment

Data were extracted in Microsoft Excel (version 2309) by AKP and checked by a second reviewer (PMH, RT, AP, or MH) (appendix 8). Risk of bias assessment was conducted independently at datapoint or outcome level by AKP and a second reviewer (PMH, RT, AP, or MH) using an adapted version of the Newcastle-Ottawa scale for cross-sectional and cohort studies,^62^ and the Cochrane RoB-2 tool for randomised studies.^63^ The Newcastle-Ottawa scale was adapted to incorporate insights from the Cochrane ROBINS-I risk of bias tool, with assistance from GRADE Public Health Group members.^64^ This included assessing adjustment for pre-identified critical confounding domains (ie, sex, age, and any measure of socioeconomic position (such as parental academic qualifications), other justifiable confounders, attrition, and missing data (appendix 9). Conflicts were resolved via consensus or discussion with a third reviewer (SVK).

Our risk of bias assessments informed data synthesis and certainty, assessed using GRADE.^59^

### Data synthesis

#### Synthesis without meta-analysis (SWiM)

Within SWiM, effect direction was coded as beneficial or harmful for each outcome at the study level, with findings categorised as inconsistent if less than 70% of extracted datapoints reported a consistent effect direction.^44^ ^65^ As per Cochrane guidance, statistical significance was not considered.^66^ Sign tests assessed evidence of effect where there were at least three studies within a synthesis. We produced modified effect direction plots (created using RStudio.V1.2.5.^67^ *),* displaying risk of bias results.^65^

#### Primary meta-analyses

We performed meta-analyses by outcome for time spent on social media, frequency of social media use, and exposure to content displaying health risk behaviour, but not for other social media activities because of heterogeneity. Given anticipated heterogeneity in study designs, settings, and measures, we used random-effect models, using the DerSimonian and Laird estimator.^68^ The proportion of total heterogeneity due to between study heterogeneity was measured using the I^2^ statistic.^69^ Since most reported outcomes for binary exposures were binary, statistical approaches were conducted to re-express continuous outcome data as odds ratios as per the Cochrane handbook, thus allowing binary and continuous outcome data to be combined.^69^ ^70^ ^71^ ^72^ For continuous outcomes, data were pooled to produce standardised β coefficients (Std. Beta) or standardised mean differences.^69^ ^70^ ^71^ ^72^ Continuous exposures are infrequently reported in systematic reviews, meaning that best practice recommendations are not available within the Cochrane handbook^69^ and interpretation of such a synthesis is difficult, therefore, we opted to not combine continuous and binary outcome data. Instead, continuous exposure and continuous outcomes were pooled separately as standardised β coefficients or standardised mean differences.^69^ ^70^ ^71^ ^72^ Continuous exposure and binary outcomes were pooled separately as odds ratios (appendix 10). We conducted transformations using guidance outlined in the Cochrane handbook, and in some instances, using the Campbell Collaboration online effect size calculator.^69^ ^70^ ^71^ ^72^

Where at least 10 studies were included in a meta-analysis, meta-regression explored heterogeneity by the following characteristics identified a priori: content viewed of health risk behaviour on social media (user *v* marketer generated), social media category (eg, social networking), social media platform (eg, Facebook), sex, average socioeconomic position of participants, development status of study setting (high *v* low-middle income country),^73^ and average age of participants (<16 *v* ≥16 years, as existing evidence shows that risk behaviours tend to peak at age 16 years and most behaviours become acceptable (albeit not necessarily legal) from a societal perspective).^74^ We used Stata version 16 for all statistical analysis.^75^

#### Subgroup and sensitivity analyses

We stratified meta-analyses by the above characteristics if at least one subgroup had two or more studies and investigated potential bias by examining results by study design (cross-sectional *v* cohort or randomised control trial); adjustment for pre-identified critical confounding domains (age, sex, and socioeconomic position); risk of bias; and excluding datapoints with samples containing individuals outside our eligible age range (10-19 years).

#### Publication bias

Publication bias or small study effects were assessed using funnel plots and the Egger’s test when 10 or more studies were included in a meta-analysis.^76^ ^77^

#### Certainty of the evidence

Certainty was assessed using GRADE,^59^ which combines information on risk of bias, imprecision, inconsistency, indirectness, and publication bias.^59^ As per GRADE, advisory group members ranked the importance of outcomes via an online survey (appendix 5), and assessed certainty for the top seven ranked outcomes (alcohol, drug, tobacco, electronic nicotine delivery system use, sexual risk behaviour, gambling, and multiple risk behaviours) using a four category system (very low to high).^59^ Observational evidence automatically started at low with the ability to upgrade or downgrade.^59^ ^78^

### Patient and public involvement

Advisory group members included policy, non-governmental, and academic stakeholders who provided guidance during protocol development and the review stages (appendix 5). Summaries for the public and policy makers will be co-produced with additional public representatives and advisory group members.

## Results

### Description of studies

**Fig 2:**
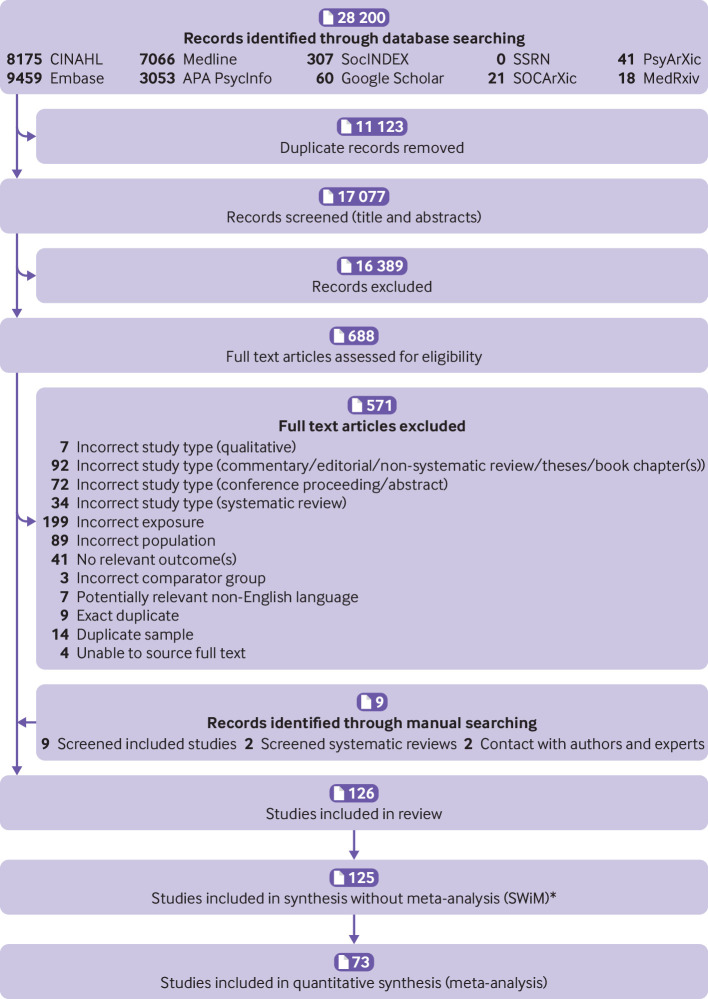
PRISMA flow diagram. APA=American Psychological Association.*One study^92^ was not included in the synthesis without meta-analysis (SWiM) as this resulted in counting of study participants twice; we were able to include estimates from this study in meta-analyses stratified by outcome where this issue did not occur

Of 17 077 studies screened, 688 full text studies were assessed, with 126 included (73 in the meta-analysis; fig 2). The final sample included 1 431 534 adolescents (mean age of 15.0 years). Most included studies were cross-sectional (n=99; 79%) and investigated high income countries (n=113; 90%),^73^ with 44 studies (35%) investigating US adolescents. Appendix 11 shows the geographical distribution of included study populations. Included and excluded study characteristics are presented in appendix 11 and 12.

For 122 included cross-sectional and cohort studies, 57 (47%) of studies were graded high risk of bias, 31 (25%) were moderate, and 34 (28%) were low. Of the four randomised controlled trials included, two were graded with some concerns and two as low risk of bias (appendix 13). Reviewer risk of bias agreement was strong (κ=0.91).^79^

### Social media measures reported 

Within included studies, many social media exposure measures were reported, with most investigating multiple measures (appendix 14). All were incorporated in our exploration of how social media use is measured, therefore, the number of datapoints reported differs across syntheses.

In total, 253 social media measures were reported: 135 (53%) assessed frequency, 61 (24%) assessed exposure to content displaying health risk behaviour, 45 (18%) assessed time spent, and 12 (5%) other social media activities. Despite our broad definition of social media, most included studies assessed a narrow range of social media categories (or adopted a broad definition). Social networking sites was the most common category investigated (56%; n=141). Of those social media measures investigating a specific platform (n=86), Facebook was most investigated (n=40), followed by Twitter (n=10).

Of those 61 measures assessing exposure to content displaying a health risk behaviour, 36 (59%) assessed marketer generated content, 16 (26%) assessed user generated content, and nine (15%) assessed both types of content. In total, 134 (53%) of the 253 social media measures provided sufficient information to differentiate between use that was active (eg, positing and commenting on posts; n=90) or passive (eg, observing others, content, or watching videos; n=44). Exposure ascertainment primarily used unvalidated adolescent self-report surveys (n=221) with a minority using data-driven codes, validated adolescent self-report questionnaires and/or clinical records (n=32).

### Social media use and health risk behaviours

#### Alcohol use

**Fig 3:**
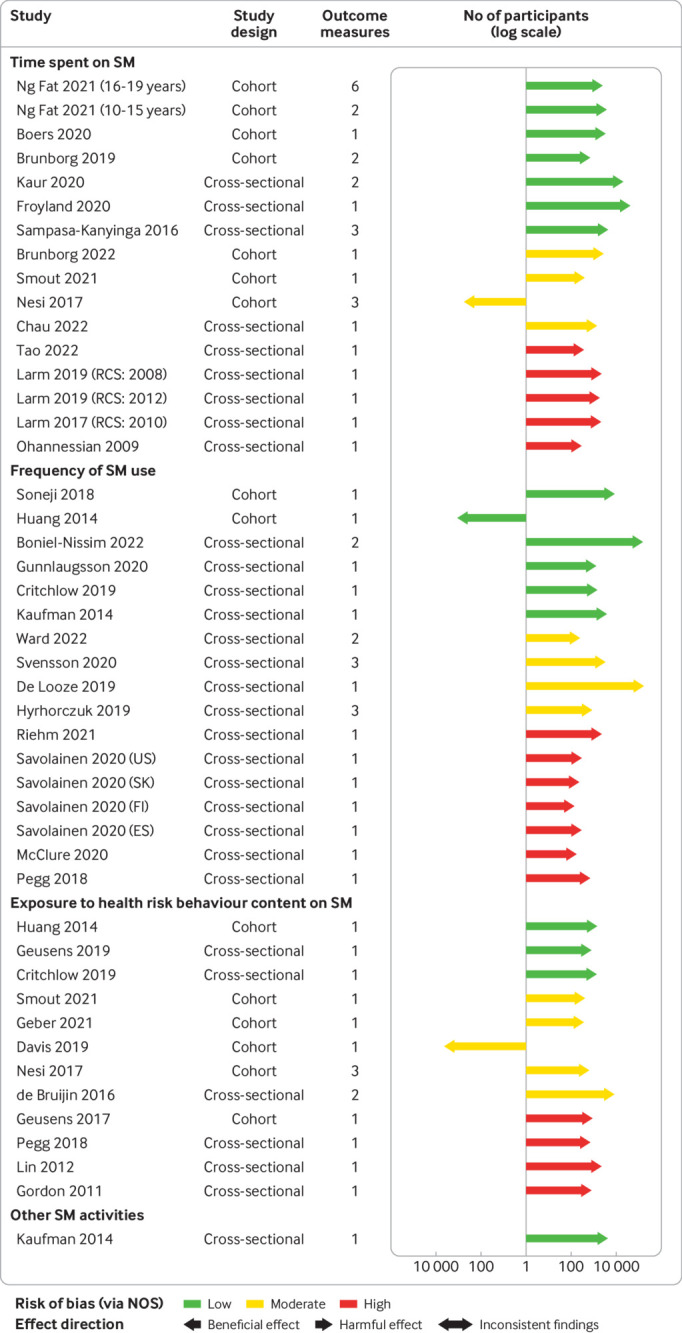
Effect direction plot for studies of the association between social media use and adolescent alcohol use, by social media exposure. Arrow size indicates sample size; arrow colour indicates study risk of bias. Sample size is represented by the size of the arrow, measured on a log scale. Outcome measure is number of outcome measures synthesised within each study. Studies organised by risk of bias grade, study design, and year of publication. Repeat cross-sectional studies, multiple study populations from different countries, and age subsets originating from the same study reported as separate studies. ESP=Spain; FIN=Finland; KOR=South Korea; NOS=assessed via adapted Newcastle Ottawa Scale; RCS=repeat cross-sectional study; SM=social media

Alcohol use was the most extensively studied outcome (appendix 15). For time spent, 15/16 studies (93.8%) reported harmful associations (95% confidence interval 71.7% to 98.9%; n=100 354; sign test P<0.001), 16/17 studies (94.1%) for frequency (73.0% to 99.0%; n=390 843; sign test P<0.001), and 11/12 studies (91.7%) for exposure to content displaying health risk behaviour (64.6% to 98.5%; n=24 247; sign test P=0.006). The category other social media activities was investigated by one study (ie, participants had a Facebook account) that reported a harmful association (95% confidence interval 20.7% to 100%; n=4485; fig 3 for effect direction plot).

**Fig 4:**
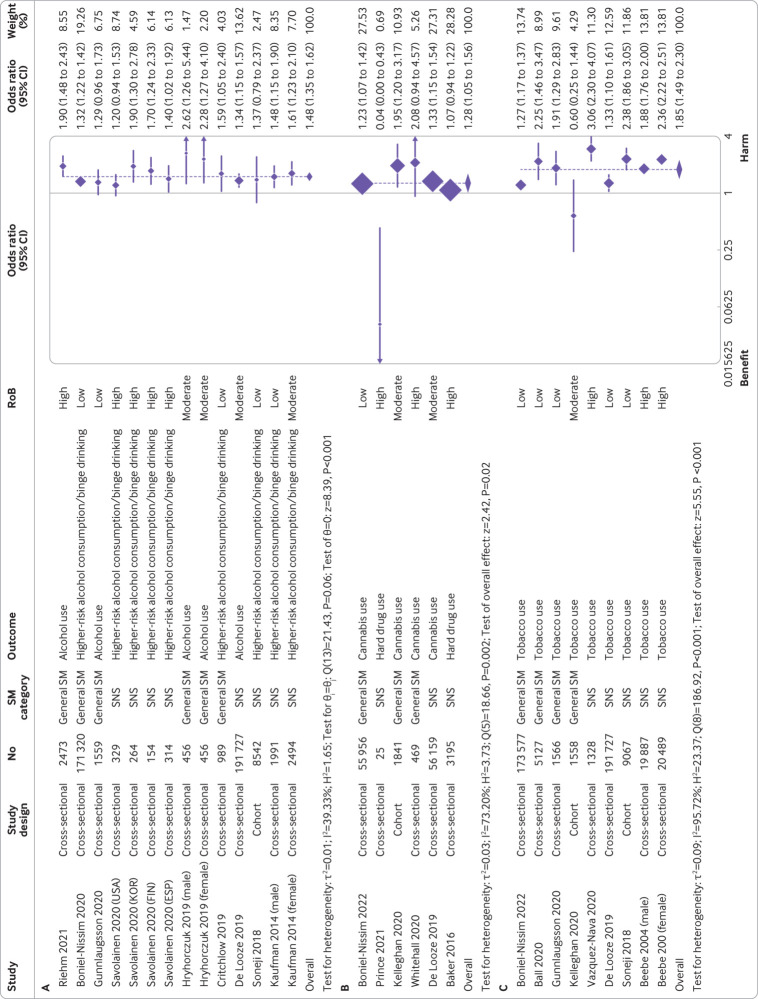
Forest plots for association between frequency of social media use and A) alcohol use, B) drug use, and C) tobacco use. (A) Binary exposure (frequent or daily *v* infrequent or non-daily) and binary or continuous alcohol use outcome meta-analysis, with OR used as common metric (N=383 068). (B) Binary exposure (frequent/daily *v* infrequent/non-daily) and binary or continuous drug use outcome meta-analysis, with OR used as common metric (N=117 645). (C) Binary exposure (frequent *v* infrequent) and binary or continuous tobacco use outcome meta-analysis, with OR used as common metric (N=424 326). Hard drugs were defined by the cited papers as prescription drugs without a doctor’s prescription (eg, OxyContin), cocaine crack, methamphetamine, ecstasy, heroin, or opioids. CI=confidence interval; ESP=Spain; FIN=Finland; KOR=South Korea; OR=odds ratio; RoB=Risk of bias; SM=social media; SNS=Social networking sites

In meta-analyses, frequent or daily (*v* infrequent or non-daily) social media use was associated with increased alcohol consumption (odds ratio 1.48 (95% confidence interval 1.35 to 1.62); I^2^=39.3%; n=383 068; fig 4A). In stratified analyses (appendix 16, p162-167), effect sizes were larger for adolescents 16 years or older compared with participants who were younger than 16 years (1.80 (1.46 to 2.22) *v* 1.34 (1.26 to 1.44); P<0.01 for test of differences). Social networking sites were associated with increased alcohol consumption, while microblogging or media sharing sites had an unclear association (P=0.03).

Social media use for 2 h or more (*v* <2 h per day) was associated with increased alcohol consumption (odds ratio 2.12 (95% confidence interval 1.53 to 2.95); I^2^=82.0%; n=12 390), as was exposure (*v* no exposure) to content displaying health risk behaviours (2.43 (1.25 to 4.71); I^2^=98.0%; n=14 731; appendix 16, p168). Stratified analyses for time spent and exposure to health risk behaviour content generally did not show important differences by age and social media category (appendix 16, p169-171). Associations were slightly stronger for exposure to health risk behaviour content in user generated (3.21 (2.37 to 4.33)) versus marketer generated content (2.35 (1.30 to 4.22); P=0.28; appendix 16, p172). Meta-analyses for frequency of use, time spent on social media, and exposure to content displaying health risk behaviour (assessed on a continuous scale) showed similar findings (appendix 16, p173-174). On stratification (appendix 16, p175-179), for exposure to content displaying health risk behaviour, associations were larger for adolescents 16 years or older versus younger than 16 years (Std.Beta 0.35 (0.29 to 0.42) *v* 0.09 (0.05 to 0.13); P<0.001). The results indicated that for every one standard deviation increase in exposure to content displaying health risk behaviour, alcohol consumption increased by 0.35 standard deviation for older adolescents compared with 0.09 standard deviation for younger adolescents.

#### Drug use

For drug use, across all exposures investigated, 86.6% of studies (n=13/15; 53.3% low/moderate risk of bias) reported harmful associations (appendix 16, p180). The pooled odds ratio for frequent or daily use (*v* infrequent or non-daily) was 1.28 ((95% confidence interval 1.05 to 1.56), I^2^=73.2%; n=117 645) (fig 4B). Stratification showed no clear differences (appendix 16, p182-184). Few studies (n=3) assessed time spent on social media with estimates suggestive of harm (odds ratio 1.45 (95% confidence interval 0.80 to 2.64); I^2^=87.4%; n=7357 for ≤1 h *v* >1 h/day) (appendix 16, p185).

#### Tobacco use

For tobacco use, 88.9% (n=16/18; 50.0% low risk of bias) studies reported harmful associations of social media use (appendix 16, p 186). Frequent (*v* infrequent) use was associated with increased tobacco use (odds ratio 1.85 (95% confidence interval 1.49 to 2.30); I^2^=95.7%; n=424 326) (fig 4C), as was exposure (*v* no exposure) to content displaying health risk behaviours (specifically, marketer generated content) (1.79 (1.63 to 1.96); I^2^=0.00%; n=22 882) (appendix 16, p188). In stratified analyses (appendix 16, p189-193) for frequency of use, stronger associations were observed for low and middle income countries versus for high income countries (2.47 (1.56 to 3.91) *v* 1.72 (1.35 to 2.19); P=0.17), and for use of social networking sites versus for general social media (2.09 (1.72 to 2.53) *v* 1.48 (1.01 to 2.18; P=0.29).

#### Electronic nicotine delivery system use

Across all exposures investigated, 88.9% of studies (n=8/9; 77.8% low/moderate risk of bias) reported harmful associations on electronic nicotine delivery system use (appendix 16, p194). Exposure to content displaying health risk behaviour (specifically marketer generated content) (*v* no exposure) was associated with increased electronic nicotine delivery system use (odds ratio 1.73 (95% confidence interval 1.34 to 2.23); I^2^=63.4%; n=721 322) (appendix 16, p195). No clear differences were identified on stratification (appendix 16, p196-197).

#### Sexual risk behaviour

**Fig 5:**
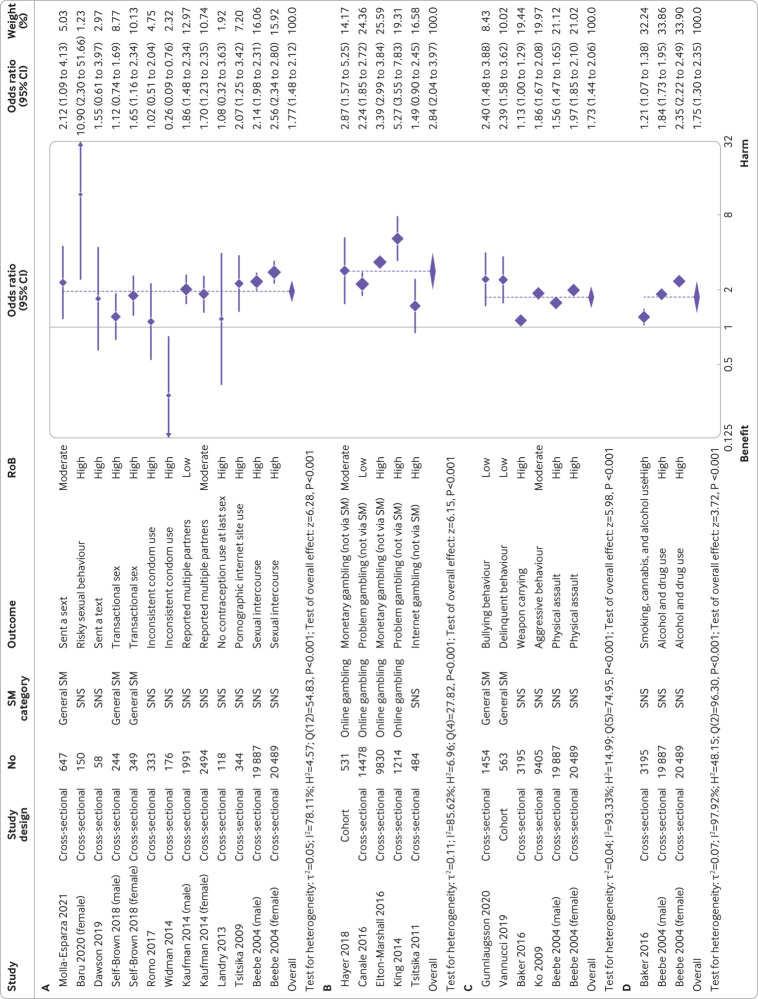
Forest plots for association between frequency of social media use and A) sexual risk behaviour, B) gambling, C) anti-social behaviour, and D) multiple risk behaviours. (A) Forest plot for binary exposure (frequent/at all *v* infrequent/not at all) and binary/continuous sexual risk behaviour outcome meta-analysis, with OR used as common metric. N=47 280. (B) Forest plot for binary exposure (frequent/at all *v* infrequent/not at all) and binary/continuous gambling outcome meta-analysis, with OR used as common metric. N=26 537. (C) Forest plot for binary exposure (frequent/at all *v* infrequent/not at all) and binary/continuous anti-social behaviour outcome meta-analysis, with OR used as common metric. N=54 993. (D) Forest plot for binary exposure (frequent/at all *v* infrequent/not at all) and binary/continuous multiple risk behaviours outcome meta-analysis, with OR used as common metric. N=43 571. CI=confidence interval; n=Number of study participants; OR=odds ratio; RoB=Risk of bias; SM=Social media; SNS=Social networking sites

After excluding one study with inconsistent findings, across all exposures investigated 90.3% (n=28/31; 67.7% high risk of bias) reported harmful associations for sexual risk behaviours (appendix 16, p 198). Frequent or at all use (*v* infrequent or not at all) was associated with increased sexual risk behaviours (eg, sending a so-called sext, transactional sex, and inconsistent condom use) (odds ratio 1.77 (95% confidence interval 1.48 to 2.12); I^2^=78.1%; n=47 280) (fig 5A). Meta-regression (coefficient −0.37 (−0.70 to −0.05); P=0.03) (appendix 16, p276) and stratified analyses (appendix 16, p200-206) suggested stronger associations for younger versus older adolescents (<16 years *v* ≥16 years), but no moderation effects were by social media category (P=0.13) or study setting (P=0.49). Few studies assessed associations for time spent on social media (appendix 16, p207).

#### Gambling

After excluding one study that had inconsistent findings, across all exposures investigated, all six studies investigating gambling reported harmful associations (appendix 16, p208). Frequent or at all use (*v* infrequent or not at all) was associated with increased gambling (not via social media) (odds ratio 2.84 (95% confidence interval 2.04 to 3.97); I^2^=85.6%; n=26 537) (fig 5B). On differentiation by social media category, a relatively large association was found for online gambling via social media (3.22 (2.32 to 4.49)), however, associations were not present for social networking sites and general social media (appendix 16, p211).

#### Anti-social behaviour

Across all exposures investigated, all 16 studies (43.8% low/moderate risk of bias) that investigated anti-social behaviour showed harmful associations (appendix 16, p212). Frequent or at all use (*v* infrequent or not at all) was associated with increased anti-social behaviour (eg, bullying, physical assault, and aggressive/delinquent behaviour) (odds ratio 1.73 (1.44 to 2.06); I^2^=93.3%; n=54 993) (fig 5C), with time spent similarly associated with increased risk (appendix 16, p214). No subgroup differences were noted (appendix 16, p215-217).

#### Inadequate physical activity

For inadequate physical activity, after excluding three studies with inconsistent findings, 36.4% of studies (n=4/11; 72.7% low/moderate risk of bias) reported harmful associations across all exposures investigated (appendix 16, p218). No association between time spent on social media (assessed on a continuous scale) and adolescent engagement in physical activity was seen (Std.Beta −0.00 (95% confidence interval −0.02 to 0.01); I^2^=59.8%; n=37 417) (appendix 16, p219), with no important differences across subgroups (appendix 16, p220-222).

#### Unhealthy dietary behaviour

Across all exposures investigated, all 13 studies (including four randomised controlled trials: two rated low risk of bias and two some concerns) that investigated unhealthy dietary behaviour showed harmful associations, with most at low risk of bias (61.5%) (appendix 16, p223). Exposure to health risk behaviour content (specifically marketer generated content) was associated with increased consumption of unhealthy food (odds ratio 2.48 (95% confidence interval 2.08 to 2.97); I^2^=0.00%; n=7892) when compared with adolescents who had no exposure (appendix 16, p224-225).

#### Multiple risk behaviours

For multiple risk behaviours, all nine studies showed harmful associations across all exposures investigated (appendix 16, p226). The pooled odds ratio for frequent and at all social media use (*v* infrequent and not at all) was 1.75 ((95% confidence interval 1.30 to 2.35); I^2^=97.9%; n=43 571) (fig 5D), but the few studies precluded stratification.

#### Sensitivity analyses

For electronic nicotine delivery system use, associations were stronger for cohort study datapoints (odds ratio 2.13 (95% confidence interval 1.72 to 2.64) *v* 1.43 (1.20 to 1.69) for cross-sectional datapoints; P=0.004) (appendix 16, p228) but no clear differences were seen for other outcomes (appendix 16, p229-240). Although based on few studies, for unhealthy dietary behaviour a stronger association was found for the randomised controlled trial datapoint versus for the cross-sectional datapoints (3.21 (1.63 to 6.30) *v* 2.48 (2.08 to 2.97); P=0.44) (appendix 16, p241).

When stratifying by adjustment for critical confounding domains, no clear differences were identified (appendix 16, p242-253), with some exceptions. Associations were stronger for unadjusted versus adjusted datapoints for exposure to content displaying health risk behaviour and alcohol use (Std.Beta 0.28 (0.14 to 0.43) *v* 0.07 (0.03 to 0.12); P=0.008) and for frequent (*v* infrequent) social media use and alcohol use (odds ratio 1.54 (95% confidence interval 1.36 to 1.78) *v* 1.34 (1.24 to 1.44); P=0.06) (appendix 16, p254-255).

For alcohol use, effect sizes were generally stronger for moderate and high risk of bias datapoints (*v* low) (appendix 16, p256-257), excluding time spent (≥2 *v* <2 h per day) and exposure to health risk behaviour content (*v* no exposure) where low (compared with moderate and high) risk of bias datapoints displayed stronger associations (appendix 16, p258-259). For drug use and sexual risk and anti-social behaviour, no differences were detectable or low/moderate risk of bias datapoints showed stronger associations (compared with high) (appendix 16, p260-264). For tobacco use and gambling, stronger associations were found for high risk of bias datapoints or no clear differences were identified (appendix 16, p265-267). No clear differences by risk of bias were observed for the remaining outcomes (appendix 16, p268-269).

When we excluded datapoints that overlapped the age range of 10-19 years, a marginal reduction in effect size (appendix 16, p270) or no important differences were noted (appendix 16, p271-274).

#### Publication bias

Funnel plots and Egger’s test results suggested some publication bias in the meta-analysis investigating frequent or at all social media use (*v* infrequent or not at all) and sexual risk behaviours (P=0.04; bias towards the null) (appendix 17). Insufficient data precluded investigation of other outcomes.

#### Certainty of the evidence

As frequency was the most investigated exposure, and continuous and binary exposures reported similar effects, we focused the GRADE assessment on the binary exposure of frequency of use. We report harmful effects on alcohol use with low certainty, and with drug, tobacco, electronic nicotine delivery system use, sexual risk behaviours, gambling, and multiple risk behaviours with very low certainty.

We conducted a post-hoc GRADE assessment for exposure to content displaying health risk behaviour (*v* no exposure) and unhealthy dietary behaviour because of the substantial difference in quality of evidence observed (four randomised controlled trials); we report moderate GRADE certainty (table 1, appendix 18).^59^

**Table 1:** Condensed summary of findings and certainty of evidence (as per GRADE)

Outcome	No. of participants (studies)	Anticipated absolute effects (95% CI)		Odds ratio (95% CI)	Certainty of evidence
		Risk in the control group with infrequent social media use	Risk in the exposed group with frequent social media use		
**Frequency of social media use (frequent *v* infrequent) **					
Alcohol use	383 068 (n=9)	48.9%	58.6% (56.4% to 60.8%)	1.48 (1.35 to 1.62)	Low
Drug use	117 645 (n=6)	17.0%	20.8% (17.7% to 24.2%)	1.28 (1.05 to 1.56)	Very low*
Tobacco use	424 326 (n=8)	12.1%	20.3% (17.0% to 24.0%)	1.85 (1.49 to 2.30)	Very low†
Electronic nicotine delivery system use (effect direction)	18 047 (n=3)	66.7% of studies reported harmful association of social media use on adolescent use (20.8% to 93.9%)			Very low‡
Sexual risk behaviour	47 280 (n=10)	37.0%	50.9% (46.5% to 55.4%)	1.77 (1.48 to 2.12)	Very low§
Gambling	26 537 (n=5)	21.4%	43.6% (35.7% to 52.0%)	2.84 (2.04 to 3.97)	Very low*
Multiple risk behaviours	43 571 (n=2)	41.3%	55.2% (47.8% to 62.3%)	1.75 (1.30 to 2.35)	Very low¶
**Exposure to health-risk behaviour content on social media (exposure *v* no exposure)**					
Unhealthy dietary behaviour (effect direction)	521 (n=4 randomised control trials)	All studies report harmful effect of social media use on adolescent engagement (51.0% to 100.0%)			Moderate**

The risk in the intervention group (and its 95% confidence interval) is based on the assumed risk in the comparison group and the relative effect of the intervention (and its 95% CI). Studies were all observational studies unless otherwise stated. For full GRADE results, appendix 18. CI=confidence interval; GRADE=Grading of Recommendations Assessment, Development and Evaluation; OR=odds ratio.

* Downgraded by one level for risk of bias of included studies.

† Downgraded by two levels for inconsistency and risk of bias of included studies.

‡ Downgraded by two levels for imprecision and risk of bias of included studies.

§ Downgraded by two levels for publication bias, and risk of bias of included studies.

¶ Downgraded by two levels for inconsistency and risk of bias of included studies.

** Downgraded by one level for indirectness.

## Discussion

### Principal findings

Our systematic review suggests that social media use is adversely associated with several health risk behaviours for adolescents, including increased alcohol, drug, tobacco, and electronic nicotine delivery system use; gambling; and sexual risk, behaviours that are anti-social, unhealthy dietary, and multiple risk. Exposure to health risk behaviour content on social media had the strongest evidence of harm, particularly in relation to alcohol use and unhealthy dietary behaviour (moderate GRADE certainty).

### Strengths and limitations of this study

Our study had a comprehensive scope, included randomised controlled trials and adjusted estimates, assessed risk of bias and certainty of the evidence using GRADE, and focused on social media specifically (rather than digital technologies).^59^ To our knowledge, no other review has synthesised the evidence of health risk behaviours among adolescents associated with social media categories, platforms, and content, and considered whether social media impacts vary across social groups. Generally, for alcohol use, larger associations were noted for adolescents aged ≥16 years (*v* <16 years), and for exposure, to user generated content (*v* marketer generated content). For tobacco use, larger associations were observed for low and middle income countries (*v* high income countries). We followed up a preregistered protocol with decisions about critical confounding domains and stratified analyses informed by a comprehensive literature review, logic model (fig 1), and advisory group consultation.^45^ Searches covered the period from 1997 to 2022. The nature of social media use has changed dramatically across this period, but most data (66.9% of studies) were collected in the past eight years and so should be generalisable to the current social media environment.

However, objective social media measures were rare with self-report most common. When assessing frequency of use, most studies compared frequent versus infrequent, others compared daily versus non-daily, and in some studies, any use versus none. These exposure categories were combined in meta-analyses due to limited data availability, but frequency (assessed via continuous scale) reported similar findings. Some meta-analyses were based on few studies, yielding more uncertain estimates. However, meta-analysis is feasible even with two studies, and whether a meta-analysis should be conducted where possible is debated.^66^ ^80^ A meta-analysis was performed when three or more studies were available for a given synthesis and this meta-analysis was complemented with a narrative synthesis using the SWiM reporting guideline and effect direction plots.^44^ ^65^ ^80^ As recommended by Cochrane,^81^ adjustments for multiple tests were not conducted. Instead, effect sizes were the focus of interpretation where possible, outcomes and analyses of interest were prespecified in the published protocol,^45^ subgroup analyses were interpreted with caution, and results were not selected for emphasis on the basis of a statistically significant P value, with all conducted analyses presented. Moreover, although the review focused on harmful risk behaviours, social media may have positive or negligible harmful influences on some outcomes, such as physical activity and drug use; thus, a holistic view should be taken when interpreting the review findings.

Our sensitivity analysis by confounder adjustment, focused on critical confounding domains (ie, age, sex, and socioeconomic position). We acknowledge that other shared risk factors may exist between social media and health risk behaviours (eg, parental health risk behaviours). Cross-sectional studies are subject to reverse causation, as reflected in the logic model (fig 1). A bidirectional association may therefore exist, with adolescents who engage in health risk behaviours to be more inclined to use social media to obtain peer approval and positive feedback. However, we identified harmful associations across study designs, including longitudinal studies, which adjusted for baseline measures of outcomes and randomised controlled trials.

Included randomised controlled trials involved random assignment of study participants to existing or manipulated social media posts (where all authors stipulated the means by which they tried to mimic the actual social media environment). For example, De Jans and colleagues identified a harmful association between exposure to manipulated Instagram posts showing a fictitious influencer promoting a snack that was low in nutritional value (*v* high) and unhealthy snack consumption.^82^ A limitation of this study was its use of a fictitious influencer, which may limit its validity. Yet, Folkvord and colleagues overcame this limitation through use of existing Instagram posts showing a popular social influencer consuming energy dense snacks (*v* vegetables), subsequently finding participants exposed to the energy dense snack condition consumed fewer vegetables when compared with participants exposed to the vegetable condition.^83^ The use of existing Instagram posts from a popular social influencer among the target group of participants helped to improve external validity. Thus, the moderate GRADE rating of certainty for the included randomised controlled trials suggests a causal effect of content displaying health risk behaviour on unhealthy dietary behaviour, although these studies still had limitations (eg, no real-time exposure to social media).

### Comparison with other studies

Previous reviews have focused on social media use to deliver behaviour change interventions, finding that this platform has potential.^9^ ^84^ ^85^ Less attention has been paid to the implications of social media itself for health. Vannucci and colleagues identified cross-sectional correlations between social media use and substance use and risky sexual behaviour in adolescents, however, they were unable to separate out general electronic media use (electronic media with a direct component involving social interactions with others (2022 personal communication with A Vannucci)) from social media use; although, they did include some exploratory sensitivity analyses of potential differences by type of social media assessment.^42^ Curtis and colleagues reported correlations between alcohol related social media content and alcohol consumption and alcohol related problems in young adults; however, the authors did not explore if associations differed between exposure to user or marketer generated content.^32^ Importantly, both reviews did not incorporate adjusted estimates or identify randomised controlled trial evidence and did not formally assess risk of bias of the underlying evidence.^32^ ^42^

### Policy implications

As social media reaches diverse populations, reporting of population characteristics and disaggregating results by socio-demographic groups should be prioritised. With most studies conducted in high income countries, research into low and middle income countries is needed.^34^ SWiM findings suggested that social media use may present beneficial effects on adolescent engagement in physical activity, although meta-analysis (based on four cross-sectional studies) did not substantiate this conclusion. Further research into this outcome would allow health policy makers to potentially harness the benefits social media use could present on adolescent health. Moreover, many of the risk behaviours investigated can be experimental during adolescence, and the extent to which these behaviours affect health may vary. Longitudinal research tracking adolescents into adulthood would help to study this gap. Well conducted randomised trials studying risk behaviours over and above unhealthy dietary behaviour would yield more robust evidence than currently available and have been shown to be feasible. Addressing the limitations of existing randomised controlled trials and use of real time monitoring data of social media use would allow for more definitive causal conclusions on the effects of social media activity on adolescent health risk behaviours.

The methodological limitations in the evidence may reflect limited access to data required to investigate social media’s health implications, adding weight to calls to compel social media corporations to share data with researchers.^86^ ^87^ In the absence of real time objective data, the development of generalisable, validated measures of social media use (considering social media activities performed eg, active or passive use) would facilitate comparability across studies. Awareness of the aspects of social media most harmful to adolescents (eg, user and marketer generated content), could support development and expedite introduction of the delayed UK Online Safety Bill, aimed at securing adolescents’ online safety.^86^ ^88^ The importance of exposure to marketer generated content identified in this article in potentially promoting health risk behaviours highlights gaps in the Bill, which largely focuses on user generated content, and the unmet need for legislation targeting influencer marketing.^86^ ^89^ ^90^ Further research into this area could prove fruitful for informing regulation.

Adopting a multisector approach to securing adolescent online safety by improving digital literacy, school education, and resource provision to parents, educators, and health professionals might help to improve understanding of the different aspects of social media use (eg, time spent, exposure to health risk behaviour content) and the potential risks or benefits they present to adolescent health.^91^

### Conclusion

Our article finds predominantly harmful associations between social media use and adolescent health risk behaviours. However, this finding is based largely on cross-sectional studies, using self-reported measures of social media use, and is at risk of residual confounding due to many confounders that remain unadjusted for. Experimental and risk taking behaviours are an inherent part of adolescence; however, as safeguards for a digital world are still evolving, precaution across academic, governmental, health and educational sectors may be warranted before the risks adolescents’ use of social media is fully understood.

**What is already known on this topic**

Social media use has rapidly expanded, and it is now recognised as a platform to promote health, but concerns exist over its potential impact on adolescent health risk behaviours
Reviews have identified harmful associations between social media and some risk behaviours
These studies were not of university and college populations, did not investigate social media explicitly or consider different aspects of social media use, and did not critically appraise studies

**What this study adds**

Our systematic review shows social media use is associated with several adverse health risk behaviours in adolescents, although evidence for causality remains limited
Exposure to content showing health risk behaviours has stronger evidence for adverse effects, particularly in relation to an unhealthy diet (which had the best quality evidence) and alcohol use

## Data Availability

Data analysed were based on published data. Template data forms, the data extracted from included studies, and data used for analyses are available from the corresponding author on reasonable request. The study protocol is published on PROSPERO: https://www.crd.york.ac.uk/prospero/display_record.php?RecordID=179766 (ID: CRD42020179766)

## Web extra

material supplied by authors

Web appendix: Online appendix

## Notes

Contributors: AKP, SVK, AP, and MH drafted the study protocol. AKP conducted literature searches, and all authors contributed to the screening process and selection of included studies. AKP conducted data extraction and risk of bias assessments; data were checked and an independent risk of bias scoring undertaken by SVK, AP, MH, RT, and PMH. AKP completed all data analysis, had full access to all the data in the study, and takes responsibility for the integrity of the data and the accuracy of the data analysis. All authors critically reviewed and approved the manuscript. The corresponding author attests that all listed authors meet authorship criteria and that no other meeting the criteria have been omitted. AKP acts as guarantor.

The lead author (AKP) affirms that the manuscript is an honest, precise, and transparent account of the review reported, with no important aspects of the review omitted. Any discrepancies from the review as planned (and, registered) have been explained.

Dissemination to participants and related patient and public communities: The findings will be disseminated via conference presentations, press releases, and social media outlets. The authors will disseminate findings to media organisations and press councils, and also national health organisations that are instrumental in the development of social media recommendations for adolescents. Additionally, the findings will be disseminated via advisory group members working across academic, governmental, and charity sectors.

Provenance and peer review: Not commissioned; externally peer reviewed.

## References

[1] Kemp S. Facebook statistics and trends. DataReportal. 2022. https://datareportal.com/essential-facebook-stats [accessed 29 August 2022]

[2] Kemp S. Instagram statistics and trends. DataReportal. 2022. https://datareportal.com/essential-instagram-stats [accessed 29 August 2022]

[3] Gebremeskel RH Sessoms K Krehnbrink M Haney CJ Coyne-Beasley T . Social media use and adolescent risk taking behavior. J Adolesc Health 2014;54:S46-7. 10.1016/j.jadohealth.2013.10.106.

[4] Chou WY Hunt YM Beckjord EB Moser RP Hesse BW . Social media use in the United States: implications for health communication. J Med Internet Res 2009;11:e48. 10.2196/jmir.1249. 19945947 PMC2802563

[5] Aichner T Grünfelder M Maurer O Jegeni D . Twenty-five years of social media: A review of social media applications and definitions from 1994 to 2019. Cyberpsychol Behav Soc Netw 2021;24:215-22. 10.1089/cyber.2020.0134. 33847527 PMC8064945

[6] Boniel-Nissim M van den Eijnden RJJM Furstova J . International perspectives on social media use among adolescents: implications for mental and social well-being and substance use. Comput Human Behav 2022;129. 10.1016/j.chb.2021.107144.

[7] World Health Organization. Global action plan on physical activity 2018-2030: more active people for a healthier world. Geneva: WHO; 2018. https://apps.who.int/iris/bitstream/handle/10665/272722/9789241514187-eng.pdf

[8] Hamm MP Shulhan J Williams G Milne A Scott SD Hartling L . A systematic review of the use and effectiveness of social media in child health. BMC Pediatr 2014;14:138. 10.1186/1471-2431-14-138. 24886048 PMC4047773

[9] Goodyear VA Wood G Skinner B Thompson JL . The effect of social media interventions on physical activity and dietary behaviours in young people and adults: a systematic review. Int J Behav Nutr Phys Act 2021;18:72. 10.1186/s12966-021-01138-3. 34090469 PMC8180076

[10] Anderson M Jiang J . Teens, social media and technology 2018. Pew Research Center, 2018. https://www.pewresearch.org/internet/2018/05/31/teens-social-media-technology-2018/.

[11] Internet Matters. From survive to thrive: supporting digital family life after lockdown. UK: Internet Matters; 2021. https://www.internetmatters.org/wp-content/uploads/2021/05/Internet-Matters-From-Survive-to-Thrive-Report.pdf

[12] Moreno MA Whitehill JM . Influence of social media on alcohol use in adolescents and young adults. Alcohol Res 2014;36:91-100. 26259003 10.35946/arcr.v36.1.09 PMC4432862

[13] Qutteina Y Hallez L Mennes N De Backer C Smits T . What do adolescents see on social media? A diary study of food marketing images on social media. Front Psychol 2019;10:2637. 10.3389/fpsyg.2019.02637. 31824391 PMC6883917

[14] Winpenny EM Marteau TM Nolte E . Exposure of children and adolescents to alcohol marketing on social media websites. Alcohol 2014;49:154-9. 10.1093/alcalc/agt174. 24293506 PMC3932831

[15] Alruwaily A Mangold C Greene T . Child social media influencers and unhealthy food product placement. Pediatrics 2020;146:e20194057. 10.1542/peds.2019-4057. 33106342 PMC7786816

[16] Sacks G Looi ESY . The advertising policies of major social media platforms overlook the imperative to restrict the exposure of children and adolescents to the promotion of unhealthy foods and beverages. Int J Environ Res Public Health 2020;17:4172. 10.3390/ijerph17114172. 32545343 PMC7312784

[17] Moreno MA Parks M Richardson LP . What are adolescents showing the world about their health risk behaviors on MySpace? MedGenMed 2007;9:9. 18311359 PMC2234280

[18] Laestadius LI Wahl MM . Mobilizing social media users to become advertisers: Corporate hashtag campaigns as a public health concern. Digit Health 2017;3:2055207617710802. 10.1177/2055207617710802. 29942600 PMC6001194

[19] Jackson KM Janssen T Gabrielli J . Media/marketing influences on adolescent and young adult substance abuse. Curr Addict Rep 2018;5:146-57. 10.1007/s40429-018-0199-6. 30393590 PMC6208350

[20] Stiglic N Viner RM . Effects of screentime on the health and well-being of children and adolescents: a systematic review of reviews. BMJ Open 2019;9:e023191. 10.1136/bmjopen-2018-023191. 30606703 PMC6326346

[21] Moreno MA Briner LR Williams A Walker L Christakis DA . Real use or “real cool”: adolescents speak out about displayed alcohol references on social networking websites. J Adolesc Health 2009;45:420-2. 10.1016/j.jadohealth.2009.04.015. 19766949

[22] Litt DM Stock ML . Adolescent alcohol-related risk cognitions: the roles of social norms and social networking sites. Psychol Addict Behav 2011;25:708-13. 10.1037/a0024226. 21644803

[23] Huang GC Unger JB Soto D . Peer influences: the impact of online and offline friendship networks on adolescent smoking and alcohol use. J Adolesc Health 2014;54:508-14. 10.1016/j.jadohealth.2013.07.001. 24012065 PMC4694047

[24] Papasolomou I Melanthiou Y . Social media: Marketing public relations’ new best friend. J Promot Manage 2012;18:319-28. 10.1080/10496491.2012.696458.

[25] Sawyer SM Afifi RA Bearinger LH . Adolescence: a foundation for future health. Lancet 2012;379:1630-40. 10.1016/S0140-6736(12)60072-5. 22538178

[26] Nesi J Rothenberg WA Hussong AM Jackson KM . Friends’ alcohol-related social networking site activity predicts escalations in adolescent drinking: Mediation by peer norms. J Adolesc Health 2017;60:641-7. 10.1016/j.jadohealth.2017.01.009. 28325545 PMC6402495

[27] Mokdad AH Forouzanfar MH Daoud F . Global burden of diseases, injuries, and risk factors for young people’s health during 1990-2013: a systematic analysis for the Global Burden of Disease Study 2013. Lancet 2016;387:2383-401. 10.1016/S0140-6736(16)00648-6. 27174305

[28] Murray CJL Aravkin AY Zheng P GBD 2019 Risk Factors Collaborators . Global burden of 87 risk factors in 204 countries and territories, 1990-2019: a systematic analysis for the Global Burden of Disease Study 2019. Lancet 2020;396:1223-49. 10.1016/S0140-6736(20)30752-2. 33069327 PMC7566194

[29] Gopinath B Flood VM Burlutsky G Mitchell P . Combined influence of health behaviors on total and cause-specific mortality. Arch Intern Med 2010;170:1605-7. 10.1001/archinternmed.2010.303. 20876415

[30] UK Cabinet Office. Risk behaviours and negative outcomes. Trends in risk behaviours and negative outcomes amongst children and young people. UK: UK Cabinet Office; 2014. https://assets.publishing.service.gov.uk/government/uploads/system/uploads/attachment_data/file/452169/data_pack_risk_behaviours_and_negative_outcomes.pdf

[31] Otto C Kaman A Erhart M . Risk and resource factors of antisocial behaviour in children and adolescents: results of the longitudinal BELLA study. Child Adolesc Psychiatry Ment Health 2021;15:61. 10.1186/s13034-021-00412-3. 34686200 PMC8539834

[32] Curtis BL Lookatch SJ Ramo DE McKay JR Feinn RS Kranzler HR . Meta-analysis of the association of alcohol-related social media use with alcohol consumption and alcohol-related problems in adolescents and young adults. Alcohol Clin Exp Res 2018;42:978-86. 10.1111/acer.13642. 29786874 PMC5984178

[33] Frost RL Rickwood DJ . A systematic review of the mental health outcomes associated with Facebook use. Comput Human Behav 2017;76:576-600. 10.1016/j.chb.2017.08.001.

[34] Orben A . Teenagers, screens and social media: a narrative review of reviews and key studies. Soc Psychiatry Psychiatr Epidemiol 2020;55:407-14. 10.1007/s00127-019-01825-4. 31925481

[35] Lanthier-Labonté S Dufour M Milot DM Loslier J . Is problematic Internet use associated with alcohol and cannabis use among youth? A systematic review. Addict Behav 2020;106:106331. 32151892 10.1016/j.addbeh.2020.106331

[36] Fs A Khani A Daud F . A systematic review of immersive social media activities and risk factors for sexual boundary violations among adolescents. IIUM Med J Malaysia. 2021;20:159-70. 10.31436/imjm.v20i1.1766.

[37] Marino C Gini G Vieno A Spada MM . The associations between problematic Facebook use, psychological distress and well-being among adolescents and young adults: A systematic review and meta-analysis. J Affect Disord 2018;226:274-81. 10.1016/j.jad.2017.10.007. 29024900

[38] Livingstone S Nandi A Banaji S Stoilova M . Young adolescents and digital media: Uses, risks and opportunities in low- and middle-income countries: A rapid evidence review. Gender and Adolescence Global Evidence, 2017.

[39] Diderichsen F Evans T Whithead M . The social basis of disparities in health. In: Evans T Whitehead M Diderichsen F Bhuiya A Wirth M , eds. Challenging inequities in health: From ethics to action. Oxford University Press, 2001, 10.1093/acprof:oso/9780195137408.003.0002.

[40] Keles B McCrae N Grealish A . A systematic review: The influence of social media on depression, anxiety and psychological distress in adolescents. Int J Adolesc Youth 2019;00:1-15. 10.1080/02673843.2019.1590851.

[41] Kelly Y Zilanawala A Booker C Sacker A . Social media use and adolescent mental health: Findings from the UK Millennium Cohort Study. EClinicalMedicine 2019;6:59-68. 10.1016/j.eclinm.2018.12.005. 31193561 PMC6537508

[42] Vannucci A Simpson EG Gagnon S Ohannessian CM . Social media use and risky behaviors in adolescents: A meta-analysis. J Adolesc 2020;79:258-74. 10.1016/j.adolescence.2020.01.014. 32018149

[43] Page MJ McKenzie JE Bossuyt PM . The PRISMA 2020 statement: an updated guideline for reporting systematic reviews. BMJ 2021;372:n71. 10.1136/bmj.n71. 33782057 PMC8005924

[44] Campbell M McKenzie JE Sowden A . Synthesis without meta-analysis (SWiM) in systematic reviews: reporting guideline. BMJ 2020;368:l6890. 10.1136/bmj.l6890. 31948937 PMC7190266

[45] Purba AK, Henery PH, Thomson RM, Pearce A, Henderson M KS. Does social media influence adolescent engagement in health risk behaviours? A protocol for a systematic review and meta-analysis. PROSPERO 2020 CRD42020179766.

[46] Purba AK Henery PM Thomson RM Pearce A Henderson M Katikireddi SV . Does social media influence adolescent engagement in health risk behaviours? A protocol for a systematic review and meta-analysis. 2020. https://www.gla.ac.uk/media/Media_718614_smxx.pdf

[47] Lefebvre C Glanville J Briscoe S . Searching for and selecting studies. In: Higgins J Thomas J Chandler J , eds. Cochrane Handbook for Systematic Reviews of Interventions version 6.2 (updated February 2021). Cochrane, 2021.

[48] World Health Organization. Adolescent health. Geneva: World Health Organization; 2020. https://www.who.int/health-topics/adolescent-health [accessed 8 January 2022].

[49] Sawyer SM Azzopardi PS Wickremarathne D Patton GC . The age of adolescence. Lancet Child Adolesc Health 2018;2:223-8. 10.1016/S2352-4642(18)30022-1. 30169257

[50] Moreno MA Parks MR Zimmerman FJ Brito TE Christakis DA . Display of health risk behaviors on MySpace by adolescents: prevalence and associations. Arch Pediatr Adolesc Med 2009;163:27-34. 10.1001/archpediatrics.2008.528 19124700

[51] Sloan L Quan-Haase A . The SAGE handbook of social media research methods. SAGE Publications Ltd, 2017.

[52] Parke J Wardle J Rigbye J Parke A . Exploring social gambling: scoping, classification and evidence review. Gambling Comission, 2012, https://eprints.lincoln.ac.uk/id/eprint/16412/1/Social%20Gambling.pdf.

[53] Aburahmah L Al Rawi H Izz Y Syed L . Online social gaming and social networking sites. Procedia Comput Sci 2016;82:72-9. 10.1016/j.procs.2016.04.011.

[54] Kaakinen M Sirola A Savolainen I Oksanen A . Young people and gambling content in social media: An experimental insight. Drug Alcohol Rev 2020;39:152-61. 10.1111/dar.13010. 31815340

[55] Bumble. Signing up & getting started. USA: Bumble; 2021. https://bumble.com/en/help/how-old-do-i-need-to-be-to-use-bumble

[56] Tinder. Tinder overview. USA: Tinder; 2021. https://www.help.tinder.com/hc/en-us/articles/4403779020941-What-happens-if-I-ve-been-age-restricted-on-Tinder

[57] Grindr. Grindr terms and conditions of service. USA: Grindr; 2020. https://www.grindr.com/terms-of-service/ [accessed 4 August 2021]

[58] MacArthur G Caldwell DM Redmore J . Individual-, family-, and school-level interventions targeting multiple risk behaviours in young people. Cochrane Database Syst Rev 2018;10:CD009927. 10.1002/14651858.CD009927.pub2. 30288738 PMC6517301

[59] Guyatt GH Oxman AD Schünemann HJ Tugwell P Knottnerus A . GRADE guidelines: a new series of articles in the Journal of Clinical Epidemiology. J Clin Epidemiol 2011;64:380-2. 10.1016/j.jclinepi.2010.09.011. 21185693

[60] Mendeley Ltd . Mendeley Desktop (Version 1.19.4). Mendeley, 2019.

[61] Veritas Health Innovation . Covidence Systematic Review Software. Veritas Health Innovation, 2020.

[62] Wells G Shea B O’Connell D Peterson J . The Newcastle-Ottawa Scale (NOS) for assessing the quality of nonrandomised studies in meta-analyses. The Ottawa Hospital Research Institute, 2000, https://www.ohri.ca/programs/clinical_epidemiology/oxford.asp.

[63] The Cochrane Collaboration . Revised Cochrane risk-of-bias tool for randomized trials (RoB 2). London, UK: The Cochrane Collaboration; 2021. https://methods.cochrane.org/bias/resources/rob-2-revised-cochrane-risk-bias-tool-randomized-trials

[64] Sterne JAC Hernán MA Reeves BC . ROBINS-I: a tool for assessing risk of bias in non-randomised studies of interventions. BMJ 2016;355:i4919. 10.1136/bmj.i4919. 27733354 PMC5062054

[65] Boon MH Thomson H . The effect direction plot revisited: application of the 2019 Cochrane Handbook guidance on alternative synthesis methods. Res Synth Methods 2021;12:29-33. 10.1002/jrsm.1458. 32979023 PMC7821279

[66] McKenzie JE Brennan SE . Synthezing and presenting findings using other methods. In: Higgins J Thomas J Chandler J , eds. Cochrane Handbook for Systematic Reviews of Interventions version 6.2. Cochrane, 2021.

[67] RStudio Team . RStudio: Integrated Development for R. RStudio. PBC, 2020.

[68] Veroniki AA Jackson D Viechtbauer W . Methods to estimate the between-study variance and its uncertainty in meta-analysis. Res Synth Methods 2016;7:55-79. 10.1002/jrsm.1164. 26332144 PMC4950030

[69] Deeks JJ Higgins JPT Altman DG . Analysing data and undertaking meta-analyses. In: Higgins JPT Thomas J Chandler J Cumpston M Li T Page MJWV , eds. Cochrane Handbook for Systematic Reviews of Interventions version 6.2 (updated February 2021). Cochrane, 2021.

[70] Schünemann H Oxman A Vist G . Interpreting results and drawing conclusions. In: Higgins G Green S , eds. Cochrane Handbook for Systematic Reviews of Interventions version 5.1.0. Cochrane, 2011.

[71] Wilson DB. Practical meta-analysis effect size calculator. https://campbellcollaboration.org/research-resources/effect-size-calculator.html. [accessed 04 March 2020].

[72] Higgins JPT Li T Deeks JJ . Choosing effect measures and computing estimates of effect. In: Higgins J Thomas J Chandler J , eds. Cochrane Handbook for Systematic Reviews of Interventions version 6.2 (updated February 2021). Cochrane, 2021.

[73] The World Bank. World bank country and lending groups country classification. USA: The World Bank; 2021. https://datahelpdesk.worldbank.org/knowledgebase/articles/906519-world-bank-country-and-lending-groups

[74] Harden KP Kretsch N Mann FD . Beyond dual systems: A genetically-informed, latent factor model of behavioral and self-report measures related to adolescent risk-taking. Dev Cogn Neurosci 2017;25:221-34. 10.1016/j.dcn.2016.12.007. 28082127 PMC6886471

[75] StataCorp . Stata Statistical Software: Release 16. StataCorp LLC, 2019.

[76] Peters JL Sutton AJ Jones DR Abrams KR Rushton L . Contour-enhanced meta-analysis funnel plots help distinguish publication bias from other causes of asymmetry. J Clin Epidemiol 2008;61:991-6. 10.1016/j.jclinepi.2007.11.010. 18538991

[77] Ioannidis JPA Trikalinos TA . The appropriateness of asymmetry tests for publication bias in meta-analyses: A large survey. Can Med Assoc J 2007;176:1091-1096. 17420491 10.1503/cmaj.060410 PMC1839799

[78] McMaster University. Developed by Evidence Prime, Inc. GRADEpro GDT: GRADEpro Guideline Development Tool. US: Evidence Prime; 2020. https://www.gradepro.org.

[79] McHugh ML . Interrater reliability: the kappa statistic. Biochem Med (Zagreb) 2012;22:276-82. 10.11613/BM.2012.031 23092060 PMC3900052

[80] Ioannidis JPA Patsopoulos NA Rothstein HR . Reasons or excuses for avoiding meta-analysis in forest plots. BMJ 2008;336:1413-1415. 10.1136/bmj.a117 18566080 PMC2432114

[81] Higgins J Green S . Multiplicity in systematic reviews. In: Higgins J Green S , eds. Cochrane Handbook for Systematic Reviews of Interventions version 5.1.0. Cochrane, 2011.

[82] De Jans S Spielvogel I Naderer B Hudders L . Digital food marketing to children: How an influencer’s lifestyle can stimulate healthy food choices among children. Appetite 2021;162:105182. 10.1016/j.appet.2021.105182 33667499

[83] Folkvord F de Bruijne M . The effect of the promotion of vegetables by a social influencer on adolescents’ subsequent vegetable intake: A pilot study. Int J Environ Res Public Health 2020;17:2243. 10.3390/ijerph17072243. 32225032 PMC7177819

[84] Chau MM Burgermaster M Mamykina L . The use of social media in nutrition interventions for adolescents and young adults-A systematic review. Int J Med Inform 2018;120:77-91. 10.1016/j.ijmedinf.2018.10.001. 30409348 PMC6983924

[85] Günther L Schleberger S Pischke CR . Effectiveness of social media-based interventions for the promotion of physical activity: Scoping review. Int J Environ Res Public Health 2021;18:13018. 10.3390/ijerph182413018. 34948628 PMC8702047

[86] House of Lords House of Commons Joint Committee on the Draft Online Safety Bill. Draft Online Safety Bill Report of Session 2021-22. UK: House of Lords House of Commons; 2021. https://committees.parliament.uk/committee/534/draft-online-safety-bill-joint-committee/

[87] Senate of the United States. To support research about the impact of digital communication platforms on society by providing privacy-protected, secure pathways for independent research on data held by large internet companies. 117th Congress USA: Senate of the United States; 2021. https://www.coons.senate.gov/imo/media/doc/text_pata_117.pdf

[88] Carah N Brodmerkel S . Alcohol marketing in the era of digital media platforms. J Stud Alcohol Drugs 2021;82:18-27. 10.15288/jsad.2021.82.18. 33573719

[89] Department for Digital. Culture, media and sport. Draft online safety bill 2021. London, UK: HM Government; 2021. https://assets.publishing.service.gov.uk/government/uploads/system/uploads/attachment_data/file/985033/Draft_Online_Safety_Bill_Bookmarked.pdf

[90] Michaelsen F Collini L Jacob C . The impact of influencers on advertising and consumer protection in the single market. Think Tank European Parliament, 2022, https://www.europarl.europa.eu/thinktank/en/document/IPOL_STU(2022)703350.

[91] Jones D, Labour MP, West BN, Kendall L, Labour MP, West L. House of Commons Science and Technology Committee. Impact of social media and screen-use on young people’s health. London: House of Commons; 2019. https://publications.parliament.uk/pa/cm201719/cmselect/cmsctech/822/822.pdf

[92] Geusens F Beullens K . Strategic self-presentation or authentic communication? Predicting adolescents’ alcohol references on social media. J Stud Alcohol Drugs 2017;78:124-33. 10.15288/jsad.2017.78.124. 27936372

