# Supplementary material for: Social media use and health risk behaviours in young people: systematic review and meta-analysis
Source: BMJ. 2023 Nov 29;383:e073552. doi: 10.1136/bmj-2022-073552 (PMC10685288; doi:10.1136/bmj-2022-073552)
Supplement: Supplementary file 1 — Web appendix: Online appendix [file pura073552.ww.pdf]

Supplementary appendix
to
Social media use and adolescent engagement in health-risk behaviours: A systematic review and meta-analysis

Dr Amrit Kaur Purba (PhD)
Dr Rachel M Thomson (MPH)
Dr Paul M Henery (PhD)
Dr Anna Pearce (PhD)
Professor Marion Henderson (PhD)
Professor S Vittal Katikireddi (PhD)

Table of Contents

Appendix 1. Deviations from protocol 3
Appendix 2. Details of search strategies conducted 4
Appendix 3. Potentially relevant non-English reports 12
Appendix 4. Process of social media categorisation 13
Appendix 5. Advisory group 15
Appendix 6. Included outcomes 16
Appendix 7. Meta-analyses and synthesis without meta-analysis (SWiM) decision rules 17
Appendix 8. Data extraction form exemplar- study and datapoint level variables 20
Appendix 9. Newcastle-Ottawa Scale (NOS) risk of bias assessment 28
  Adapted Newcastle Ottawa Scale (NOS): cross-sectional studies 28
  Adapted Newcastle Ottawa Scale (NOS): cohort studies 31
  Assessing domain level and overall risk of bias (RoB) for included datapoints 33
Appendix 10. Process for data transformations for meta-analysis 35
Appendix 11. Characteristics of included studies 36
Appendix 12. Characteristics of excluded studies 63
Appendix 13. Risk of bias domain and overall grade for included datapoints and studies 104
Appendix 14. Social media measures reported in included studies 113
Appendix 15. Exposure and outcome combinations amenable to meta-analysis 152
Appendix 16. Synthesis without meta-analysis (SWiM), meta-analyses, meta-regression, subgroup, and sensitivity analyses 158
  Alcohol use 158
  Drug use 179
  Tobacco use 185
  Use of electronic nicotine delivery systems (ENDS) 193
  Sexual risk behaviour 197
  Gambling 207
  Anti-social behaviour 211
  Inadequate physical activity 217
  Unhealthy dietary behaviour 222
  Multiple risk behaviours 225
  Sensitivity analyses 227
Appendix 17. Assessment of publication bias/small study effects 275
Appendix 18. Summary of findings and certainty of evidence 276
Appendix 19. PRISMA checklists 279
Appendix 20. Registered/published protocols 284
References 285

Appendix 1. Deviations from protocol

To improve the interpretability and comparability of the review findings, we made the following explanatory clarifications and minor protocol deviations.

Clarifications to and deviations from protocol as published:

- Updated the search to include all eligible studies from 01/1997-06/2022.
- Clarified online dating platforms were not included under the exposure social media use.
- Clarified randomised control trials were eligible for inclusion.
- Clarified the risk of bias tool to be used for randomised control trials is the Cochrane RoB-2 tool.
- We did not include ethnicity as a critical confounding factor when assessing study adjustment as the potential role of ethnicity as a confounder was not deemed substantial given the likely homogenous populations investigated within many included studies.
- We included online (social) gaming and online (social) gambling within included exposures following discussions with advisory group members, due to their emerging placement in social media platforms and the overlap in functionalities they share with social media.
- For planned subgroup analyses/meta-regression we originally stated if two or fewer studies were found in a given sub-category of a binary/multi-categorical moderator, formal moderation analysis would not be conducted for that specific variable. Due to the limited number of included studies, we allowed for more leniency using the data available and the decision was made to perform subgroup analyses/meta-regression if at least one subgroup had two or more studies, noting the requirement to interpret any conclusions with caution. The same rule was applied when conducting sensitivity analyses.
- Where duplicate data were identified across multiple studies, we prioritised inclusion of studies which had the longest period of follow up, followed by studies which had the largest most representative sample size, and then by most recent. We did not anticipate there would be many cohort studies identified during protocol development, thus we did not initially prioritise the inclusion of studies with longer follow up periods.
- Following discussions with statistical experts post publication of the protocol, the decision was made to combine binary exposure and binary/continuous outcomes in line with guidance provide by Cochrane,[1] expressed as odds ratios. For continuous exposure measures, we stated estimates would be converted to standardised regression coefficients/correlations. Where possible we converted regression coefficients to standardised regression coefficients. We used the recent method recommended by Mathur and Vanderwheele[2] which facilitates the conversion of a Pearson correlation coefficient to standardised mean difference.
- We conducted a post-hoc GRADE assessment for the exposure to health-risk behaviour content on social media and outcome unhealthy dietary behaviour due to the substantial differences in the studies used to assess this exposure/outcome combination (specifically investigation by randomised control trials).

Appendix 2. Details of search strategies conducted

All searches outlined in Table A were initially conducted on 30.04.2020 and repeated on 06.06.2022.

Table A. Sources searched and corresponding hits

Sources searched	Date of initial search	Number of hits	Date of updated search	Number of hits	Total number of hits
**Electronic databases**					
CINAHL	30.04.2020	6,740	06.06.2022	1,435	8,175
EMBASE	30.04.2020	6,896	06.06.2022	2,563	9,459
MEDLINE	30.04.2020	5,253	06.06.2022	1,813	7,066
APA PsychINFO	30.04.2020	2,545	06.06.2022	508	3,053
SocINDEX	30.04.2020	2,45	06.06.2022	62	307
**Pre-print repositories**					
SSRN	30.04.2020	0	06.06.2022	0	0
SocArXic	30.04.2020	11	06.06.2022	10	21
PsyArXiv	30.04.2020	6	06.06.2022	35	41
medRxiv	30.04.2020	18	06.06.2022	0	18
**Internet search engine**					
Google Scholar	30.04.2020	30	06.06.2022	30	60
**Total number of hits**					**28,200**
**Total number of hits following removal of duplicates**					**17,077**

Legend: Abbreviations: APA = American Psychological Association; EMBASE = Excerpta Medical Database; and MEDLINE = Medical Literature Analysis and Retrieval System Online.

Table B. Cumulative Index to Nursing and Allied Health Literature (CINAHL) search strategy

String number	String
1	(MH "Adolescence+") OR (MH "Child+") OR (MH "Students+") OR (MH "Students, High School") OR (MH "Schools, Middle") OR (MH "Schools, Secondary")
2	TI ("young people" OR youth OR "school child*" OR teen* OR "young person*" OR "middle school" OR middle-school OR "secondary school" OR "high school"OR iGen OR "generation Z" OR "gen Z") OR AB ("young people" OR youth OR "school child*" OR teen* OR "young person*" OR "middle school" OR middle-school OR "secondary school" OR "high school" OR iGen OR "generation Z" OR "gen Z")
3	S1 OR S2
4	(MH "Social Networking+") OR (MH "Social Media+") OR (MH "Smartphone") OR (MH "Internet+") OR (MH "Screen Time") OR (MH "Instant Messaging")
5	TI ("screen time" OR "social media" OR "social network* site" OR "social networking" OR "social-networking" OR "web 2.0" OR "online game*" OR "online gaming" OR "online social gaming" OR hashtag OR "instant messag*" OR instagram OR "Whats App" OR whatsapp OR facebook OR twitter OR linkedin OR youtube OR "you tube" OR tumblr OR vine OR snapchat OR myspace OR bebo OR reddit OR neknominate OR myspace OR wickr OR telegram OR whisper OR "kik messenger" OR "Tencent QQ" OR wechat OR meetup OR tiktok OR hinge OR happn OR bumble OR grindr OR Tinder OR "inner circle" OR periscope OR twitch) N2 (usage OR use*)) OR AB ("screen time" OR "social media" OR "social network* site" OR "social networking" OR "social-networking" OR "web 2.0" OR "online game*" OR "online gaming" OR "online social gaming" OR hashtag OR "instant messag*" OR instagram OR "Whats App" OR whatsapp OR facebook OR twitter OR linkedin OR youtube OR "you tube" OR tumblr OR vine OR snapchat OR myspace OR bebo OR reddit OR neknominate OR myspace OR wickr OR telegram OR whisper OR "kik messenger" OR "Tencent QQ" OR wechat OR meetup OR tiktok OR hinge OR happn OR bumble OR grindr OR Tinder OR "inner circle" OR periscope OR twitch) N2 (usage or use*))
6	TI ("screen time" OR "social media" OR "social networking" OR "social-networking" OR "social network* site*" OR "web 2.0" OR "online game*" OR "online gaming" OR "online social gaming" OR hashtag OR "instant messag*" OR instagram OR "Whats App" OR whatsapp OR facebook OR twitter OR linkedin OR youtube OR "you tube" OR tumblr OR vine OR snapchat OR myspace OR bebo OR reddit OR neknominate OR myspace OR wickr OR telegram OR whisper OR "kik messenger" OR "Tencent QQ" OR wechat OR meetup OR tiktok OR hinge OR happn OR bumble OR grindr OR Tinder OR "inner circle" OR periscope OR twitch) OR AB ("screen time" OR "social media" OR "social networking" OR "social network* site*" OR "social-networking" OR "web 2.0" OR "online game*" OR "online gaming" OR "online social gaming" OR hashtag OR "instant messag*" OR instagram OR "Whats App" OR whatsapp OR facebook OR twitter OR linkedin OR youtube OR "you tube" OR tumblr OR vine OR snapchat OR myspace OR bebo OR reddit OR neknominate OR myspace OR wickr OR telegram OR whisper OR "kik messenger" OR "Tencent QQ" OR wechat OR meetup OR tiktok OR hinge OR happn OR bumble OR grindr OR Tinder OR "inner circle" OR periscope OR twitch)
7	S4 OR S5 OR S6
8	(MH "Risk Taking Behavior+") OR (MH "Substance Abuse+") OR (MH "Substance Abusers+")
9	TI ("substance use*" OR "substance misuse*" OR risk-behav* OR "risk behav*" OR "risky behav*" OR "risk-taking behav*" OR "multiple risk behav*") OR AB ("substance use*" OR "substance misuse*" OR risk-behav* OR "risk behav*" OR "risky behav*" OR "risk-taking behav*" OR "multiple risk behav*")
10	S8 OR S9
11	(MH "Tobacco+") OR (MH "Smoking+") OR (MH "Tobacco Products+")

12	TI ("adolescent smok*" OR "chewing tobacco" OR "tobacco dependence" OR "tobacco use" OR "tobacco consumption" OR "tobacco snuff" OR cigarette OR "smoking initiation" OR "smoking behav*") OR AB ("adolescent smok*" OR "chewing tobacco" OR "tobacco dependence" OR "tobacco use" OR "tobacco consumption" OR "tobacco snuff" OR cigarette OR "smoking initiation" OR "smoking behav*")
13	S11 OR S12
14	(MH "Electronic Cigarettes")
15	TI ("electronic nicotine delivery system*" OR e-cigarette* OR Juul OR vaping OR vape) OR AB ("electronic nicotine delivery system*" OR e-cigarette* OR Juul OR vaping OR vape)
16	S14 OR S15
17	(MH "Drinking Behavior+") OR (MH "Alcohol Abuse+") OR (MH "Alcoholic Intoxication+")
18	TI ("underage drinking" OR "under-age drinking" OR "under age drinking" OR temperance OR "alcohol use*" OR "alcohol intake" OR "problem drinking" OR "alcoholism" OR "alcohol abstinence" OR "drinking behaviour" OR "alcohol consumption" OR "binge drinking") OR AB ("underage drinking" OR "under-age drinking" OR "under age drinking" OR temperance OR "alcohol use*" OR "alcohol intake" OR "problem drinking" OR "alcoholism" OR "alcohol abstinence" OR "drinking behaviour" OR "alcohol consumption" OR "binge drinking")
19	S17 OR S18
20	(MH "Street Drugs+") OR (MH "Drugs") OR (MH "Cannabis") OR (MH "Cocaine+")
21	TI ("cannabis use*" OR "cannabis addict*" OR "illicit drug*" OR "drug abuse*" OR "drug use*" OR "drug misuse*" OR weed OR skunk OR marijuana OR "special k" OR crack OR methamphetamine* OR ecstasy OR heroin OR LSD OR steroid* OR ketamine OR MDMA OR GHB OR GBL) OR AB ("cannabis use*" OR "cannabis addict*" OR "illicit drug*" OR "drug abuse*" OR "drug use*" OR "drug misuse*" OR weed OR skunk OR marijuana OR "special k" OR crack OR methamphetamine* OR ecstasy OR heroin OR LSD OR steroid* OR ketamine OR MDMA OR GHB OR GBL)
22	S20 OR S21
23	(MH "Juvenile Delinquency") OR (MH "Theft+") OR (MH "Disruptive Behavior") OR (MH "Gangs") OR (MH "Violence+")
24	TI ("antisocial behav*" OR "anti-social behav*" OR "social problem*" OR assault OR fighting OR steal* OR shoplift* OR vandal* OR "public nuisance") OR AB ("antisocial behav*" OR "anti-social behav*" OR "social problem*" OR assault OR fighting OR steal* OR shoplift* OR vandal* OR "public nuisance")
25	S23 OR S24
26	(MH "Pregnancy in Adolescence+") OR (MH "Sexually Transmitted Diseases+") OR (MH "Pregnancy, Unwanted") OR (MH "Sex+") OR (MH "Unsafe Sex") OR (MH "HIV Infections+")
27	TI (sexting OR sex-text OR "sex text" OR "sexual behav*" OR "sexual intercourse" OR "sexually transmitted infection*" OR STIs OR STDs OR "teen* pregnancy" OR "unprotected sex" OR "first intercourse" OR "casual sexual relations*" OR "intimate sexual contact" OR "under age sex" OR "underage sex" OR "under-age sex" OR "underage pregnancy" OR "under-age pregnancy" OR "under age pregnancy") OR AB (sexting OR sex-text OR "sex text" OR "sexual behav*" OR "sexual intercourse" OR "sexually transmitted infection*" OR STIs OR STDs OR "teen* pregnancy" OR "unprotected sex" OR "first intercourse" OR "casual sexual relations*" OR "intimate sexual contact" OR "under age sex" OR "underage sex" OR "under-age sex" OR "underage pregnancy" OR "under-age pregnancy" OR "under age pregnancy")
28	S26 OR S27
29	(MH "Gambling")
30	TI (gambling OR betting) OR AB (gambling OR betting)
31	S29 OR S30
32	(MH "Sweetened Beverages") OR (MH "Fast Foods") OR (MH "Adolescent Nutrition") OR (MH "Eating Behavior+")
33	TI ("unhealthy diet*" OR "poor diet*" OR "dietary behav*" OR "sugary drink*" OR sweet*) OR AB ("unhealthy diet*" OR "poor diet*" OR "dietary behav*" OR "sugary drink*" OR sweet*)
34	S32 OR S33
35	(MH "Physical Activity") OR (MH "Exercise+") OR (MH "Physical Fitness+") OR (MM "Life Style, Sedentary")
36	TI ("physical inactiv*" OR "physical activ*" OR exercis* OR sport*) OR AB ("physical inactiv*" OR "physical activ*" OR exercis* OR sport*)
37	S35 OR S36
38	S10 OR S13 OR S16 OR S19 OR S22 OR S25 OR S28 OR S31 OR S34 OR S37
39	S3 AND S7 AND S38

Legend: *Initial search:* date of search-30.04.2020. Interface-EBSCOhost. Database and coverage-Cumulative Index to Nursing and Allied Health Literature (CINAHL), 1981 to present. Limits applied-01.01.1997 to 30.04.2020. *Updated search:* date of search-06.06.2022. Interface- EBSCOhost. Database and coverage-CINAHL, 1981 to present. Limits applied-01.04.2020 to 31.06.2022.

Table C. Excerpta Medical Database (EMBASE) search strategy

String number	String
1	adolescent/ or child/ or juvenile/
2	middle school student/ or student/ or high school student/
3	("young people" or youth or "school child*" or teen* or "young person*" or "middle school" or middle-school or "secondary school" or "high school" or iGen or "generation Z" or "gen Z").ab,ti.
4	1 or 2 or 3
5	online social network/ or social media/ or smart phone/ or internet/ or screen time/
6	(("screen time" or "social media" or "social networking" or "social-networking" or "social network* site*" or "web 2.0" or "online game*" or "online gaming" or "online social gaming" or hashtag or "instant messag*" or instagram or "Whats App" or whatsapp or facebook or twitter or linkedin or youtube or "you tube" or tumblr or vine or snapchat or myspace or bebo or reddit or neknominate or myspace or wickr or telegram or whisper or "kik messenger" or "Tencent QQ" or wechat or meetup or tiktok or hinge or happn or bumble or grindr or Tinder or "inner circle" or periscope or twitch) adj2 (usage or use*)).ab,ti.

7	("screen time" or "social media" or "social networking" or "social-networking" or "social network* site*" or "web 2.0" or "online game*" or "online gaming" or "online social gaming" or hashtag or "instant messag*" or instagram or "Whats App" or whatsapp or facebook or twitter or linkedin or youtube or "you tube" or tumblr or vine or snapchat or myspace or bebo or reddit or neknominate or myspace or wickr or telegram or whisper or "kik messenger" or "Tencent QQ" or wechat or meetup or tiktok or hinge or happn or bumble or grindr or Tinder or "inner circle" or periscope or twitch).ab,ti.
8	5 or 6 or 7
9	high risk behavior/ or "substance use"/ or substance abuse/
10	("substance misuse*" or "substance use behav*" or "risk taking behav*" or "risk-taking behav*" or "risk behav*" or "risk-behav*" or "risky behav*" or "multiple risk behav*").ab,ti.
11	9 or 10
12	chewing tobacco/ or tobacco/ or smokeless tobacco/ or tobacco dependence/ or "tobacco use"/ or tobacco consumption/ or tobacco snuff/ or cigarette/ or cigarette smoking/ or adolescent smoking/ or smoking/
13	("smoking initiation" or "smoking behav*").ab,ti.
14	12 or 13
15	exp electronic cigarette/
16	("electronic nicotine delivery system*" or e-cigarette* or Juul or vaping or vape).ab,ti.
17	15 or 16
18	underage drinking/ or binge drinking/ or alcohol consumption/ or drinking behavior/ or alcohol abstinence/ or alcoholism/ or alcohol abuse/
19	("alcohol intoxication" or "problem drinking" or "alcohol intake" or "alcohol use*" or temperance or "under-age drinking" or "under age drinking" or "underage drinking").ab,ti.
20	18 or 19
21	"cannabis use"/ or cannabis addiction/ or illicit drug/ or drug abuse/
22	("street drug*" or "drug use*" or "drug misuse*" or weed or skunk or cannabis or marijuana or cocaine or "special k" or crack or methamphetamine* or ecstasy or heroin or LSD or steroid* or ketamine or MDMA or GHB or GBL).ab,ti.
23	21 or 22
24	antisocial behavior/ or social problem/ or assault/ or physical violence/ or gang/ or fighting/ or theft/ or juvenile delinquency/
25	(steal* or shoplift* or vandal* or "public nuisance" or "physical assault" or "anti-social behav*").ab,ti.
26	24 or 25
27	adolescent pregnancy/ or sexting/ or sexually transmitted disease/ or unwanted pregnancy/ or sexual behavior/ or sexual intercourse/ or acquired immune deficiency syndrome/ or Human immunodeficiency virus/
28	("unwanted pregnancy" or "sexually transmitted infection*" or STIs or STDs or "teen* pregnancy" or "unprotected sex*" or "first intercourse" or "casual sexual relations*" or "intimate sexual contact" or "under age sex" or "underage sex*" or "under-age sex*" or " underage pregnancy" or " under age pregnancy" or "under-age pregnancy" or sex-text or "sex text" or "sexual behav*" or "sexual risk").ab,ti.
29	27 or 28
30	gambling/
31	(betting or gambling).ab,ti.
32	30 or 31
33	unhealthy diet/ or sugar-sweetened beverage/ or fast food/ or adolescent nutrition/
34	("poor diet*" or "dietary behav*" or "eating behav*" or "sugary drink*" or sweet*).ab,ti.
35	33 or 34
36	physical inactivity/ or exercise/ or physical activity/ or fitness/ or sedentary lifestyle/
37	("physical inactiv*" or "physical activ*" or exercis* or sport*).ab,ti.
38	36 or 37
39	11 or 14 or 17 or 20 or 23 or 26 or 29 or 32 or 35 or 38
40	4 and 8 and 39
41	limit 40 to yr="1997 -Current"

Legend: *Initial search:* date of search-30.04.2020. Interface-Ovid. Database and coverage-Excerpta Medical Database (EMBASE) 1947 to present, updated daily. Limits applied-1997 to 30.04.2020. *Updated search:* date of search-06.06.2022. Interface-Ovid. Database and coverage-Embase 1947 to present, updated daily. Limits applied-2020 to 06.06.2022.

Table D. Medical Literature Analysis and Retrieval System Online (MEDLINE) search strategy

String number	String
1	adolescent/ or child/ or students/
2	("young people" or youth or "school child*" or teen* or "young person*" or "middle school" or "middle-school" or "secondary school" or "high school" or iGen or "generation Z" or "gen Z").ab,ti.
3	1 or 2
4	online social networking/ or social media/ or smartphone/ or internet/ or screen time/
5	(("screen time" or "social media" or "social networking" or "social-networking" or "social network* site*" or "web 2.0" or "online game*" or "online gaming" or "online social gaming" or hashtag or "instant messag*" or instagram or "Whats App" or whatsapp or facebook or twitter or linkedin or youtube or "you tube" or tumblr or vine or snapchat or myspace or bebo or reddit or neknominate or myspace or wickr or telegram or whisper or "kik messenger" or "Tencent QQ" or wechat or meetup or tiktok or hinge or happn or bumble or grindr or Tinder or "inner circle" or periscope or twitch) adj2 (usage or use*)).ab,ti.
6	("screen time" or "social media" or "social networking" or "social-networking" or "social network* site*" or "web 2.0" or "online game*" or "online gaming" or "online social gaming" or hashtag or "instant messag*" or instagram or "Whats App" or whatsapp or facebook or twitter or linkedin or youtube or "you tube" or tumblr or vine or snapchat or myspace or bebo or reddit or neknominate or myspace or wickr or telegram or whisper or "kik messenger" or "Tencent QQ" or wechat or meetup or tiktok or hinge or happn or bumble or grindr or Tinder or "inner circle" or periscope or twitch).ab,ti.

7	4 or 5 or 6
8	Risk-Taking/ or exp Substance-Related Disorders/
9	("substance use*" or "substance abuse*" or "substance misuse*" or "risk taking behav*" or "risk-taking behav*" or "risk behav*" or "risk-behav*" or "risky behav*" or "multiple risk behav*").ab,ti.
10	8 or 9
11	exp "Tobacco Use"/ or exp Smoking/ or Tobacco/
12	("chewing tobacco" or "smokeless tobacco" or "tobacco dependence" or "tobacco consumption" or "tobacco snuff" or "cigarette smoking" or "adolescent smok*" or "smoking initiation" or "smoking behav*" or cigarette*).ab,ti.
13	11 or 12
14	Electronic Nicotine Delivery Systems/
15	("electronic cigarette*" or "e-cigarette*" or Juul or vaping or vape).ab,ti.
16	14 or 15
17	alcohol drinking/ or binge drinking/ or underage drinking/ or drinking behavior/ or alcohol abstinence/ or temperance/ or alcoholism/
18	("alcohol consumption" or "alcohol abuse*" or "alcohol intoxication" or "problem drinking" or "alcohol intake" or "alcohol use*" or "under-age drinking" or "under age drinking" or "underage drinking").ab,ti.
19	17 or 18
20	"Marijuana Use"/ or Marijuana Abuse/ or exp Illicit Drugs/ or exp Drug Misuse/
21	("cannabis use*" or "cannabis addict*" or "drug abuse*" or "street drug*" or "drug use*" or "drug misuse*" or weed or skunk or cannabis or marijuana or cocaine or "special k" or crack or methamphetamine* or ecstasy or heroin or LSD or steroid* or ketamine or MDMA or GHB or GBL).ab,ti.
22	20 or 21
23	social problems/ or juvenile delinquency/ or violence/ or theft/
24	("anti-social behav*" or "antisocial behav*" or assault or gang or fight* or steal* or shoplift* or vandal* or "public nuisance" or "physical assault").ab,ti.
25	23 or 24
26	pregnancy in adolescence/ or pregnancy, unwanted/ or sexual behavior/ or unsafe sex/ or exp Sexually Transmitted Diseases/ or exp HIV infections/
27	(sexting or sex-text or "sex text" or "sexual intercourse" or "unwanted pregnancy" or "sexually transmitted infection*" or STIs or STDs or "teen* pregnancy" or "unprotected sex*" or "first intercourse" or "casual sexual relations*" or "intimate sexual contact" or "under age sex" or "underage sex*" or "under-age sex*" or "underage pregnancy" or "under age pregnancy" or "under-age pregnancy" or "sexual behav*" or "sexual risk").ab,ti.
28	26 or 27
29	Gambling/
30	(betting or gambling).ab,ti.
31	29 or 30
32	Diet/ or Sugar-Sweetened Beverages/ or Fast Foods/
33	("unhealthy diet*" or "adolescent nutrition" or "poor diet*" or "dietary behav*" or "eating behav*" or "sugary drink*" or sweet*).ab,ti.
34	32 or 33
35	Sedentary Behavior/ or exp Exercise/ or exp Physical Fitness/
36	("physical inactiv*" or "physical activ*" or exercis* or sport*).ab,ti.
37	35 or 36
38	10 or 13 or 16 or 19 or 22 or 25 or 28 or 31 or 34 or 37
39	3 and 7 and 38
40	limit 39 to yr="1997 -Current"

Legend: *Initial search:* date of search-30.04.2020. Interface-Ovid. Database and coverage-Medical Literature Analysis and Retrieval System Online (MEDLINE)(R),1946 to present. Limits applied-1997 to 30.04.2020. *Updated search:* date of search-06.06.2022. Interface-Ovid. Database and coverage- MEDLINE(R),1946 to present. Limits applied-2020 to 06.06.2022.

Table E. American Psychological Association (APA) PsycINFO search strategy

String number	String
1	(DE "Middle School Students" OR DE "High School Students" OR DE "Students")
2	TI (adolescent* OR child* OR "young people" OR youth OR "school child*" OR teen* OR "young person*" OR "middle school" OR middle-school OR "secondary school" OR "high school" OR iGen OR "generation Z" OR "gen Z") OR AB (adolescent* OR child* OR "young people" OR youth OR "school child*" OR teen* OR "young person*" OR "middle school" OR middle-school OR "secondary school" OR "high school" OR iGen OR "generation Z" OR "gen Z")
3	S1 OR S2
4	(DE "Online Social Networks" OR DE "Internet" OR DE "Social Media" OR DE "Smartphones" OR DE "Screen Time")
5	TI ("screen time" OR "social media" OR "social network* site*" OR"social networking" OR "social-networking" OR "web 2.0" OR "online game*" OR "online gaming" OR "online social gaming" OR hashtag OR "instant messag*" OR instagram OR "Whats App" OR whatsapp OR facebook OR twitter OR linkedin OR youtube OR "you tube" OR tumblr OR vine OR snapchat OR myspace OR bebo OR reddit OR neknominate OR myspace OR wickr OR telegram OR whisper OR "kik messenger" OR "Tencent QQ" OR wechat OR meetup OR tiktok OR hinge OR happn OR bumble OR grindr OR Tinder OR "inner circle" OR periscope OR twitch) N2 (usage OR use*)) OR AB ("screen time" OR "social media" OR "social network* site*" OR "social networking" OR "social-networking" OR "web 2.0" OR "online game*" OR "online gaming" OR "online social gaming" OR hashtag OR "instant messag*" OR instagram OR "Whats App" OR whatsapp OR facebook OR twitter OR linkedin OR youtube OR "you tube" OR tumblr OR vine OR snapchat OR myspace OR bebo OR reddit OR neknominate OR myspace OR wickr OR telegram OR whisper OR "kik messenger" OR "Tencent QQ" OR wechat OR

	meetup OR tiktok OR hinge OR happn OR bumble OR grindr OR Tinder OR "inner circle" OR periscope OR twitch) N2 (usage OR use*))
6	TI ("screen time" OR "social media" OR "social network* site*" OR "social networking" OR "social-networking" OR "web 2.0" OR "online game*" OR "online gaming" OR "online social gaming" OR hashtag OR "instant messag*" OR instagram OR "Whats App" OR whatsapp OR facebook OR twitter OR linkedin OR youtube OR "you tube" OR tumblr OR vine OR snapchat OR myspace OR bebo OR reddit OR neknominate OR myspace OR wickr OR telegram OR whisper OR "kik messenger" OR "Tencent QQ" OR wechat OR meetup OR tiktok OR hinge OR happn OR bumble OR grindr OR Tinder OR "inner circle" OR periscope OR twitch) OR AB ("screen time" OR "social media" OR "social networking" OR "social network* site*" OR "social-networking" OR "web 2.0" OR "online game*" OR "online gaming" OR "online social gaming" OR hashtag OR "instant messag*" OR instagram OR "Whats App" OR whatsapp OR facebook OR twitter OR linkedin OR youtube OR "you tube" OR tumblr OR vine OR snapchat OR myspace OR bebo OR reddit OR neknominate OR myspace OR wickr OR telegram OR whisper OR "kik messenger" OR "Tencent QQ" OR wechat OR meetup OR tiktok OR hinge OR happn OR bumble OR grindr OR Tinder OR "inner circle" OR periscope OR twitch)
7	S4 OR S5 OR S6
8	(DE "Risk Taking" OR DE "Substance Use Disorder")
9	TI ("substance use*" OR "substance abuse*" OR "substance misuse*" OR "risk-behav*" OR "risk taking behav*" OR "risk-taking behav*" OR "risk behav*" OR "risky behav*" OR "multiple risk behav*") OR AB ("substance use*" OR "substance abuse*" OR "substance misuse*" OR "risk-behav*" OR "risk taking behav*" OR "risk-taking behav*" OR "risk behav*" OR "risky behav*" OR "multiple risk behav*")
10	S8 OR S9
11	(DE "Tobacco Smoking" OR DE "Smokeless Tobacco" OR DE "Tobacco Use Disorder")
12	TI ("adolescent smok*" OR "chewing tobacco" OR "tobacco dependence" OR "tobacco use" OR "tobacco consumption" OR "tobacco snuff" OR cigarette* OR "smoking initiation" OR "smoking behav*") OR AB ("adolescent smok*" OR "chewing tobacco" OR "tobacco dependence" OR "tobacco use" OR "tobacco consumption" OR "tobacco snuff" OR cigarette* OR "smoking initiation" OR "smoking behav*")
13	S11 OR S12
14	DE "Electronic Cigarettes"
15	TI ("electronic nicotine delivery system*" OR e-cigarette* OR Juul OR vaping OR vape) OR AB ("electronic nicotine delivery system*" OR e-cigarette* OR Juul OR vaping OR vape)
16	S14 OR S15
17	(DE "Underage Drinking" OR DE "Binge Drinking" OR DE "Drinking Behavior" OR DE "Alcohol Abuse" OR DE "Alcohol Drinking Patterns" OR DE "Alcohol Intoxication" OR DE "Sobriety" OR DE "Alcoholism" OR DE "Alcohol Use Disorder")
18	TI ("alcohol consumption" OR "alcohol abstinence" OR "alcohol abuse*" OR "alcohol use*" OR "problem drinking" OR "alcohol intake" OR temperance OR "under-age drinking" OR "under age drinking" OR "underage drinking") OR AB ("alcohol consumption" OR "alcohol abstinence" OR "alcohol abuse*" OR "alcohol use*" OR "problem drinking" OR "alcohol intake" OR temperance OR "under-age drinking" OR "under age drinking" OR "underage drinking")
19	S17 OR S18
20	DE "Cannabis" OR DE "Hashish" OR DE "Marijuana" OR DE "Cannabinoids" OR DE "Cannabis Use Disorder" OR DE "Drug Abuse" OR DE "Cocaine" OR DE "Drug Usage"
21	TI ("street drug*" OR"cannabis use*" OR "cannabis addict*" OR "illicit drug*" OR "drug abuse*" OR "drug use*" OR "drug misuse*" OR weed OR skunk OR marijuana OR "special k" OR crack OR methamphetamine* OR ecstasy OR heroin OR LSD OR steroid* OR ketamine OR MDMA OR GHB OR GBL) OR AB ("street drug*" OR "cannabis use*" OR "cannabis addict*" OR "illicit drug*" OR "drug abuse*" OR "drug use*" OR "drug misuse*" OR weed OR skunk OR marijuana OR "special k" OR crack OR methamphetamine* OR ecstasy OR heroin OR LSD OR steroid* OR ketamine OR MDMA OR GHB OR GBL)
22	S20 OR S21
23	DE "Antisocial Behavior" OR DE "Juvenile Delinquency" OR DE "Violence" OR DE "Gangs" OR DE "Social Issues" OR DE "Theft" OR DE "Vandalism"
24	TI ("anti-social behav*" OR shoplift* OR "social problem*" OR assault OR fighting OR steal* OR "public nuisance") OR AB ("anti-social behav*" OR shoplift* OR "social problem*" OR assault OR fighting OR steal* OR "public nuisance")
25	S23 OR S24
26	DE "Adolescent Pregnancy"OR DE "Sexting" OR DE "Sexually Transmitted Diseases" OR DE "Sexual Risk Taking" OR DE "Sexual Intercourse (Human)" OR DE "AIDS" OR DE "HIV"
27	TI ("unwanted pregnancy" OR "sexually transmitted infection*" OR STIs OR STDs OR "teen* pregnancy" OR "unprotected sex" OR "first intercourse" OR "casual sexual relations*" OR "intimate sexual contact" OR "underage sex" OR "under-age sex" OR "under age sex" OR "underage pregnancy" OR "under-age pregnancy" OR "under age pregnancy" OR sex-text OR "sex text") OR AB ("unwanted pregnancy" OR "sexually transmitted infection*" OR STIs OR STDs OR "teen* pregnancy" OR "unprotected sex" OR "first intercourse" OR "casual sexual relations*" OR "intimate sexual contact" OR "underage sex" OR "under-age sex" OR "under age sex" OR "underage pregnancy" OR "under-age pregnancy" OR "under age pregnancy" OR sex-text OR "sex text")
28	S26 OR S27
29	DE "Gambling"
30	TI ("betting OR gambling) OR AB ("betting OR gambling)
31	S29 OR S30
32	DE "Diets" OR DE "Eating Behavior" OR DE "Fast Food"
33	TI ("unhealthy diet*" OR "poor diet*" OR "dietary behav*" OR "sugary drink*" OR sweet* OR "sugar-sweetened beverage*" OR "sugar sweetened beverage*" OR "adolescent nutrition") OR AB ("unhealthy diet*" OR "poor diet*" OR "dietary behav*" OR "sugary drink*" OR sweet* OR "sugar-sweetened beverage*" OR "sugar sweetened beverage*" OR "adolescent nutrition")
34	S32 OR S33
35	(DE "Physical Activity" OR DE "Exercise" OR DE "Physical Fitness" OR DE "Sedentary Behavior")
36	TI ("physical inactiv*" OR "physical activ*" OR exercis* OR sport*) OR AB ("physical inactiv*" OR "physical activ" OR exercis* OR sport*)

37	S35 OR S36
38	S10 OR S13 OR S16 OR S19 OR S22 OR S25 OR S28 OR S31 OR S34 OR S37
39	S3 AND S7 AND S38

Legend: _Initial search:_ date of search-30.04.2020. Interface- EBSCOhost. Database and coverage-American Psychological Association (APA) PsycINFO, 1800s to present. Limits applied-01.01.1997 to 30.04.2020. _Updated search:_ date of search-06.06.2022. Interface-EBSCOhost. Database and coverage-APA PsycINFO, 1800s to present. Limits applied-01.04.2020 to 31.06.2022.

Table F. SocINDEX search strategy

String number	String
1	DE "STUDENTS" OR DE "MIDDLE school students" OR DE "HIGH school students" OR DE "ADOLESCENCE" OR DE "CHILDREN" OR DE "TEENAGERS" OR DE "YOUTH"
2	TI ("young people" OR "school child*" OR teen* OR "young person*" OR "middle school" OR middle-school OR "secondary school" OR "high school" OR iGen OR "generation Z" OR "gen Z") OR AB ("young people" OR "school child*" OR teen* OR "young person*" OR "middle school" OR middle-school OR "secondary school" OR "high school" OR iGen OR "generation Z" OR "gen Z")
3	S1 OR S2
4	DE "SOCIAL media" OR DE "INTERNET" OR DE "SOCIAL networking mobile apps"
5	TI ("smart phone" OR smartphone OR "screen time" OR "social media" OR "social network* site*" OR "social networking" OR "social-networking" OR "web 2.0" OR "online game*" OR "online gaming" OR "online social gaming" OR hashtag OR "instant messag*" OR instagram OR "Whats App" OR whatsapp OR facebook OR twitter OR linkedin OR youtube OR "you tube" OR tumblr OR vine OR snapchat OR myspace OR bebo OR reddit OR neknominate OR myspace OR wickr OR telegram OR whisper OR "kik messenger" OR "Tencent QQ" OR wechat OR meetup OR tiktok OR hinge OR happn OR bumble OR grindr OR Tinder OR "inner circle" OR periscope OR twitch) N2 (usage or use*)) OR AB ("smart phone" OR smartphone OR "screen time" OR "social media" OR "social network* site*" OR "social networking" OR "social-networking" OR "web 2.0" OR "online game*" OR "online gaming" OR "online social gaming" OR hashtag OR "instant messag*" OR instagram OR "Whats App" OR whatsapp OR facebook OR twitter OR linkedin OR youtube OR "you tube" OR tumblr OR vine OR snapchat OR myspace OR bebo OR reddit OR neknominate OR myspace OR wickr OR telegram OR whisper OR "kik messenger" OR "Tencent QQ" OR wechat OR meetup OR tiktok OR hinge OR happn OR bumble OR grindr OR Tinder OR "inner circle" OR periscope OR twitch) N2 (usage or use*))
6	TI ("smart phone" OR "smartphone" OR "screen time" OR "social media" OR "social network* site*" OR "social networking" OR "social-networking" OR "web 2.0" OR "online game*" OR "online gaming" OR "online social gaming" OR hashtag OR "instant messag*" OR instagram OR "Whats App" OR whatsapp OR facebook OR twitter OR linkedin OR youtube OR "you tube" OR tumblr OR vine OR snapchat OR myspace OR bebo OR reddit OR neknominate OR myspace OR wickr OR telegram OR whisper OR "kik messenger" OR "Tencent QQ" OR wechat OR meetup OR tiktok OR hinge OR happn OR bumble OR grindr OR Tinder OR "inner circle" OR periscope OR twitch) OR AB ("smart phone" OR "smartphone" OR "screen time" OR "social media" OR "social network* site*" OR "social networking" OR "social-networking" OR "web 2.0" OR "online game*" OR "online gaming" OR "online social gaming" OR hashtag OR "instant messag*" OR instagram OR "Whats App" OR whatsapp OR facebook OR twitter OR linkedin OR youtube OR "you tube" OR tumblr OR vine OR snapchat OR myspace OR bebo OR reddit OR neknominate OR myspace OR wickr OR telegram OR whisper OR "kik messenger" OR "Tencent QQ" OR wechat OR meetup OR tiktok OR hinge OR happn OR bumble OR grindr OR Tinder OR "inner circle" OR periscope OR twitch)
7	S4 OR S5 OR S6
8	DE "RISK-taking behavior" OR DE "SUBSTANCE abuse"
9	TI ("substance misuse*" OR "substance use*" OR risk-behav* OR "risk behav*" OR "risky behav*" OR "risk-taking behav*" OR "risk taking behav*" OR "multiple risk behav*") OR AB ("substance misuse*" OR "substance use*" OR risk-behav* OR "risk behav*" OR "risky behav*" OR "risk-taking behav*" OR "risk taking behav*" OR "multiple risk behav*")
10	S8 OR S9
11	DE "SMOKING" OR DE "TOBACCO use" OR DE "CIGARETTE smokers" OR DE "CIGARETTES"
12	TI ("adolescent smok*" OR "chewing tobacco" OR "tobacco dependence" OR "tobacco consumption" OR "tobacco snuff" OR "smoking initiation" OR "smoking behav*") OR AB (("adolescent smok*" OR "chewing tobacco" OR "tobacco dependence" OR "tobacco consumption" OR "tobacco snuff" OR "smoking initiation" OR "smoking behav*")
13	S11 OR S12
14	TI ("electronic nicotine delivery system*" OR "electronic cigarette*" OR e-cigarette* OR Juul OR vaping OR vape) OR AB ("electronic nicotine delivery system*" OR "electronic cigarette*" OR e-cigarette* OR Juul OR vaping OR vape)
15	DE "UNDERAGE drinking" OR DE "BINGE drinking" OR DE "ALCOHOL drinking" OR DE "ALCOHOLIC intoxication" OR DE "DRINKING behavior" OR DE "ALCOHOLISM" OR DE "TEMPERANCE" OR DE "YOUTH & alcohol"
16	TI ("alcohol consumption" OR "alcohol abstinence" OR "alcohol abuse*" OR "under age drinking" OR "underage drinking" OR "under-age drinking" OR "alcohol use*" OR "alcohol intake" OR "problem drinking") OR AB ("alcohol consumption" OR "alcohol abstinence" OR "alcohol abuse*" OR "under age drinking" OR "underage drinking" OR "under-age drinking" OR "alcohol use*" OR "alcohol intake" OR "problem drinking")
17	S15 OR S16
18	DE "MARIJUANA abuse" OR DE "DRUG abuse" OR DE "MARIJUANA" OR DE "DRUGS of abuse" OR DE "DRUGS" OR DE "COCAINE" OR DE "COCAINE abuse"
19	TI ("drug use*" OR "drug misuse*" OR "drug abuse*" OR "illicit drug*" OR "cannabis use*" OR "cannabis addict*" OR "illicit drug*" OR weed OR skunk OR marijuana OR "special k" OR crack OR methamphetamine* OR ecstasy OR heroin OR LSD OR steroid* OR ketamine OR MDMA OR GHB OR GBL) OR AB ("drug use*" OR "drug misuse*" OR "drug abuse*" OR "illicit drug*" OR "cannabis use*" OR "cannabis addict*" OR "illicit drug*"OR weed OR skunk OR marijuana OR "special k" OR crack OR methamphetamine* OR ecstasy OR heroin OR LSD OR steroid* OR ketamine OR MDMA OR GHB OR GBL)
20	S18 OR S19

21	DE "JUVENILE delinquency" OR DE "SCHOOL violence" OR DE "GANGS" OR DE "SCHOOL vandalism" OR DE "YOUTH gangs" OR DE "SOCIAL problems"
22	TI ("antisocial behav*" OR "anti-social behav*" OR assault OR fighting OR steal* OR shoplift* OR vandal* OR "public nuisance") OR AB ("antisocial behav*" OR "anti-social behav*" OR assault OR fighting OR steal* OR shoplift* OR vandal* OR "public nuisance")
23	S21 OR S22
24	DE "TEENAGE pregnancy" OR DE "SEXUALLY transmitted diseases" OR DE "UNWANTED pregnancy" OR "SEXUAL intercourse" OR DE "AIDS"
25	TI ("adolescent pregnancy" OR "teen* pregnancy" OR sexting OR sex-text OR "sex text" OR "sexual behav*" OR "human immunodeficiency virus" OR HIV OR "sexually transmitted infection*" OR STIs OR STDs OR "unprotected sex" OR "first intercourse" OR "casual sexual relations*" OR "intimate sexual contact" OR "under age sex" OR "under-age sex" OR "underage sex" OR "under age pregnancy" OR "underage pregnancy" OR "under-age pregnancy") OR AB ("adolescent pregnancy" OR "teen* pregnancy" OR sexting OR sex-text OR "sex text" OR "sexual behav*" OR "human immunodeficiency virus" OR HIV OR "sexually transmitted infection*" OR STIs OR STDs OR "unprotected sex" OR "first intercourse" OR "casual sexual relations*" OR "intimate sexual contact" OR "under age sex" OR "under-age sex" OR "underage sex" OR "under age pregnancy" OR "underage pregnancy" OR "under-age pregnancy")
26	S24 OR S25
27	DE "GAMBLING behavior"
28	TI (betting OR gambling) OR AB (betting OR gambling)
29	S27 OR S28
30	TI ("unhealthy diet*" OR "sugar-sweetened beverage*" OR "sugar sweetened beverage*" OR "fast food" OR "adolescent nutrition" OR "poor diet*" OR "dietary behav*" OR "eating behav*" OR "sugary drink*" OR sweet*) OR AB ("unhealthy diet*" OR "sugar-sweetened beverage*" OR "sugar sweetened beverage*" OR "fast food" OR "adolescent nutrition" OR "poor diet*" OR "dietary behav*" OR "eating behav*" OR "sugary drink*" OR sweet*)
31	DE "PHYSICAL fitness" OR DE "EXERCISE"
32	TI ("physical inactiv*" OR "physical activ*" OR exercis* OR sport* OR sedentary) OR AB (("physical inactiv*" OR "physical activ*" OR exercis* OR sport* OR sedentary)
33	S31 OR S32
34	S10 OR S13 OR S14 OR S17 OR S20 OR S23 OR S26 OR S29 OR S30 OR S33
35	S3 AND S7 AND S34

Legend: *Initial search:* date of search-30.04.2020. Interface-EBSCOhost. Database and coverage-SocINDEX with Full Text, 1908 to present. Limits applied-01.01.1997 to 30.04.2020. *Updated search:* date of search-06.06.2022. Interface-EBSCOhost. Database and coverage-SocINDEX with Full Text, 1908 to present. Limits applied-01.04.2020 to 31.06.2022.

Table G. Social Science Research Network (SSRN e-library vis SSRN) search strategy

String	Initial search: records identified for screening	Updated search: records identified for screening
(child* OR adolescent*) AND ("online social network*" OR "social media" OR "social network* site") AND risk behav*	0	0
(child* OR adolescent*) AND ("online social network*" OR "social media" OR "social network* site") AND (alcohol, OR drug*OR e-cigarette*OR smok* OR tobacco OR sex* OR diet* OR "physical inactiv*" OR antisocial OR anti-social OR gambl*)	0	0
(child* OR adolescent*) AND (facebook OR twitter OR instagram) AND risk behav*	0	0
(child* OR adolescent*) AND (facebook OR twitter OR instagram) AND (alcohol, OR drug*OR e-cigarette*OR smok* OR tobacco OR sex* OR diet* OR "physical inactiv*" OR antisocial OR anti-social OR gambl*)	0	0

Legend: *Initial search:* date of search-30.04.2020. Search/limits options-title, abstract and key words (no other limits available). *Updated search:* date of search-06.06.2022. Search/limits options-title, abstract and key words (no other limits available).

Table H. SocArXic Preprints search strategy

String	Initial search: records identified for screening	Updated search: records identified for screening
(child* OR adolescent* OR student* OR juvenile OR youth OR "young people" OR teen*) AND ("online social network*" OR "social media" OR internet OR "smart phone" OR "screen time" OR "social network* site" OR "social networking" OR "social-networking" OR facebook OR twitter OR instagram) AND (risk behav* OR "substance use" OR alcohol OR drink* OR tobacco OR smok* OR drug* OR e-cigarette* OR cannabis OR "antisocial behav*" OR "sexually transmitted disease*" OR "sexual behav*" OR sexting OR gambl* OR "unhealthy diet*" OR "physical inactiv*" OR sedentary)	11	10

Legend: *Initial search:* date of search-30.04.2020. Search/limits- no limit/filters/advance search option available. *Updated search:* date of search-06.06.2022. Search/limits-no limit/filters/advance search option available.

Table I. PsyArXiv Preprints search strategy

String	Initial search: records identified for screening	Updated search: records identified for screening
(child* OR adolescent* OR student* OR juvenile OR youth OR "young people" OR teen*) AND ("online social network*" OR "social media" OR internet OR "smart phone" OR "screen time" OR "social network* site" OR "social networking" OR "social-networking" OR facebook OR twitter OR instagram) AND (risk behav* OR "substance use" OR alcohol OR drink* OR tobacco OR smok* OR drug* OR e-cigarette* OR cannabis OR "antisocial behav*" OR "sexually transmitted disease*" OR "sexual behav*" OR sexting OR gambl* OR "unhealthy diet*" OR "physical inactiv*" OR sedentary)	6	35

Legend: _Initial search:_ date of search-30.04.2020. Search/limits-no limit/filters/advance search option available. _Updated search:_ date of search-06.06.2022. Search/limits-no limit/filters/advance search option available.

Table J. medRxiv Preprints search strategy

String	Initial search: records identified for screening	Updated search: records identified for screening
child* AND ("social network* site") AND risk behav*	18	0
child* AND ("social network* site") AND (alcohol, OR drug*OR e-cigarette*OR smok* OR tobacco OR sex* OR diet* OR "physical inactiv*" OR antisocial OR anti-social OR gambl*)	0	0
adolescent*AND ("social network* site") AND risk behav*	0	0
adolescent*AND ("social network* site") AND (alcohol, OR drug*OR e-cigarette*OR smok* OR tobacco OR sex* OR diet* OR "physical inactiv*" OR antisocial OR anti-social OR gambl*)	0	0
child* AND ("social media") AND risk behav*	0	0
child* AND ("social media") AND (alcohol, OR drug*OR e-cigarette*OR smok* OR tobacco OR sex* OR diet* OR "physical inactiv*" OR antisocial OR anti-social OR gambl*)	0	0
adolescent*AND ("social media") AND risk behav*	0	0
adolescent*AND ("social media") AND (alcohol, OR drug*OR e-cigarette*OR smok* OR tobacco OR sex* OR diet* OR "physical inactiv*" OR antisocial OR anti-social OR gambl*)	0	0

Legend: _Initial search:_ date of search-30.04.2020. Search/limits-limited to 01.01.1997-30.04.2020; title, abstract and all terms. _Updated search:_ date of search-06.06.2022. Search/limits-limited to 30.04.2020-06.06.2022; title, abstract and all terms.

Table K. Google scholar via Google search strategy

String	Initial search: records identified for screening	Updated search: records identified for screening
(adolescent OR child) AND ("social media") AND ("risk behaviour" OR “risk behavior”)	First 30 records	First 30 records

Legend: _Initial search:_ date of search-30.04.2020. Search/limits-limited to 1997-2020; advanced search function “find all words” selected. _Updated search:_ date of search-06.06.2022. Search/limits-limited to 2020-2022; advanced search function “find all words” selected.

Appendix 3. Potentially relevant non-English reports

Table A. Record of potentially relevant non-English records excluded at full-text screening

Citation	Language
Blasco V and Bernal S. Patrón de uso de internet y control parental de redes sociales como predictor de sexting en adolescentes: una perspectiva de género. *Revista de Psicología y Educación*. 2019;14(1):16-26	Spanish
Blazquez Barba M, Gomez Romero D, Frontaura Fernández I, Camacho Ojeda A, Rodriguez Salas FM, Toriz Cano H. Use of new technologies by adolescents in the search for health information. *Atencion Primaria*. 2018;50(9):547-552	Spanish
González M, Fernández ME, Urturi A, et al. Use and risks of information and communication technologies in the adolescents from 13 to 18 years. *Acta Pediatrica Espanola*. 2015; 73:146-151	Spanish
Marotta R, Rapetto U, Vismara MFM, et al. Impact and risks of new information technologies in adolescents: results of a survey conducted on 1534 subjects. G di *Neuropsichiatr dell'Età Evol*. 2018;38(1):9–13	Spanish
Pedersen W. Mobile phones, web chat, and sex among Norwegian adolescents. *Tidsskr Nor Laegeforen*. 2004;1;124(13-14):1756-1759	Norwegian
Richter M, Heilmann K, Moor I. The good, the bad and the ugly: the relationship between social media use, subjective health and risk behavior among children and adolescents. *Gesundheitswesen*. 2020;83(3):198-207	German
Stulhofer A, Vukasović T, Perišić K, Sušac N, Marjanović B, Bauer M, et al. Internet and sexual compulsivity. *Socijalna Psihijatrija*. 2005; 33:190-200	Croatian

Appendix 4. Process of social media categorisation

All social media categories outlined in the SAGE Social Media Categorisation[3] (Table A) were eligible for inclusion. Online (social) gambling and online (social) gaming were deemed eligible exposures due to their inclusion of core social media functionalities, namely interaction between users.[4,5,6]

As the functionalities of social media platforms overlap, and social media platforms may fall into several social media categories, we made efforts to categorise using the initial premise/purpose of the platform if stated. For example, the social media platform Instagram possesses functionalities central to social networking sites however its initial premise/purpose was to facilitate media-sharing. Therefore, where a study reported Instagram use, this was classified under the social media category media-sharing. Where an included study reported use of social media overall, this was classified as general social media use. Thus, we attempted to apply a consistent process to classification drawing upon the information reported within studies. This was conducted during data extraction by the lead author (AKP) and then reviewed by a second reviewer.

A similar process was applied when classifying the type of health-risk behaviour content (user-generated or marketer-generated content) for those datapoints investigating exposure to health-risk behaviour content on social media. Where the exposure pertained to exposure to advertisements, marketing, or influencer content, marketer-generated content was selected. Where the exposure pertained to user/peer posts displaying risk behaviour content, user-generated content was selected. Where there was insufficient information reported to facilitate accurate classification, both marketer and user-generated content were selected and the datapoint was not used in any subsequent stratified analyses or meta-regression.

We also made efforts to classify reported exposures into those assessing active social media use (online behaviours which facilitate direct exchange among users, e.g., commenting, liking, sending messages and otherwise engaging with other users) and passive use (monitoring of others/content without direct engagement, e.g., browsing/scrolling).[7]

Table A. Social media categories eligible for inclusion

Social media category	Definitions	Example platforms
**Social media categories outlined in SAGE social media categorisation**		
Social networking sites	Web-based services which facilitate individual construction of a public or semi-public profile within a bounded system, compose a list of other users with which they share a connection, and view and traverse their list of connections as well as those created by others within the system.[8]	Facebook<br>Snapchat<br>Instagram<br>WhatsApp<br>Twitter<br>LinkedIn<br>WeChat<br>Chat rooms<br>Instant messaging
Microblogging sites	Services which are centred on short updates which are forwarded to anyone subscribed to receive the updates.[3]	Twitter<br>Tumblr
Blogs and forums	Online forums which allow forum members to have conversations by posting messages. Blog comments are attached to blogs and usually the discussion is focussed on the topic of the blog post.[3]	LiveJournal<br>WordPress
Media-sharing sites	Services which facilitate uploading and sharing of media including pictures and videos. The majority of services have other social features such as profiles, commenting etc.[3]	YouTube<br>Pinterest<br>Instagram<br>Snapchat<br>Facebook
Geo-location-based sites	Services which allow users to connect and exchange messages based on their location.[9]	Foursquare<br>Tinder
Bookmarking sites	A website which ranks references (bookmarks) to other websites contributed by users who use the site. Users can add comments to the bookmarks and make then private or public. The act of bookmarking indicates to others that an individual is interested in a given resource.[10]	Delicious<br>StumbleUpon<br>Twitter
Social news sites	Services that allow individuals to post news items or links to outside articles and then facilitates user voting of the items. The voting is the primary social aspect, as items which get the most votes are displayed the most prominently. The community of users decide which news items are seen my more people.[3]	Reddit<br>Digg
Collaborative authoring sites	Web-based services which allow users to create content and allow individuals with access to the service to modify, edit or review that content.[11]	Wikipedia<br>Google Docs
Web conferencing	An umbrella term for types of online collaborative services including web-seminars (webinars), webcasts, and peer-level web meetings.[12]	Skype<br>Zoom

Scheduling and meeting	Web-based services which facilitate group-based decisions regarding event.[3]	Microsoft Outlook<br>Doodle<br>Google Calendar
**Additional social media categories eligible for inclusion**		
Online gaming (social gaming)	A video game which offers online interactions with other players.[13]	Multiplayer role-playing games (e.g., World of Warcraft)<br>Social instant games (e.g., Candy Crush)
Online gambling (social gambling)	Any kind of gambling conducted on social media/online platforms, which have varying degrees of social game features[4] (not including gambling via internet websites with no social game/social media features).[4]	Social networking sites real money gambling (e.g., Bingo Appy via Facebook)<br>Simulated gambling via social networking site applications (e.g., City Ville)

Appendix 5. Advisory group

We established an advisory group of experts and policy makers in the field of social media and adolescent health-risk behaviours to provide guidance during protocol development and the review stages. Recruited via expert stakeholders, members included patient/public representatives and stakeholders from policy, non-governmental, and academic sectors (Table A). In line with Grading of Recommendations Assessment, Development, and Evaluation (GRADE) approach,[14] the advisory group members ranked pre-selected outcomes according to their relative importance on a 9-point Likert scale (categories: 1-3 – of limited importance; 4 to 6 – importance; 7 to 9 – critical),[14] completed via an online survey (Table B). The review advisory group members were provided with detailed background information on the review. At the protocol stage, group members were requested to provide feedback on several factors including the relevance of the review's question, population focus, search strategy, ongoing or published studies, and grey literature selection.[15] Feedback was received during in person meetings, via Zoom or email. During the review stage advisory group members were contacted to identify relevant ongoing, planned, and unpublished studies.

Table A. Advisory group members

Name	Organisation	Period of involvement
Kirsty Blenkins	UK Health Security Agency, Office for Health Improvement and Disparities, London, UK	2020 to present
Lee Craig	Public Health Scotland, Glasgow, UK	2020 to present
Neil Coles	We Are With You, Kent, UK	2020 to February 2021
Nicholas Hickmott	We Are With You, Kent, UK	2020 to present
Professor John Holmes	Alcohol Policy, University of Sheffield, UK	2020 to present
Rachel Macpherson	Scottish Government, Edinburgh, UK	2020 to present
Dr Ross Whitehead	Public Health Scotland, Edinburgh, UK	2020 to July 2021
Dr Richard Purves	University of Stirling, Stirling, UK	2020 to present

Table B. Feedback from advisory group members (online survey)

**Rank outcomes according to their relative importance for the scope of the reviews and general public health decision-making in the context of social media use [a,b]**			
**Outcome**	**Mean score**	**Rank**	**Rating**
Multiple risk behaviours	7.50	1	Critical
Alcohol use	7.16	2	Critical
Drug use	7.00	3	Critical
Tobacco use	6.50	4	Important
Use of ENDS	5.83	5	Important
Sexual risk behaviours	5.60	6	Important
Gambling	5.16	7	Important
Antisocial behaviour	5.00	8	Important
Inadequate physical activity	5.00	8	Important
Unhealthy dietary behaviours	5.00	8	Important
**How well do the presented outcomes cover the review scope?**			
**Answers**	**Rating**	**Number of responses**	
Important outcomes presented	71%	5	
Important outcomes missing	29%	2	
Comments on missing outcomes (2):	(1) Selling and advertising of illicit substances (2) Mental health-related outcomes and bullying		

Legend: [a] 9-point Likert scale (categories: 1 to 3- of limited importance; 4 to 6- important; 7 to 9 – critical). [b] Seven members of the advisory group responded to the survey. Abbreviations: ENDS = Electronic nicotine delivery systems.

Appendix 6. Included outcomes

Table A. Definitions and illustrative examples of included outcomes

Outcome	Definition	Illustrative examples
Multiple risk behaviours	Two or more of the below outcomes.	Substance use (alcohol, tobacco, and drug use)
Alcohol use	The drinking of beverages containing ethyl alcohol.[16]	Weekly alcohol use<br>Frequency of alcohol use<br>Problem, binge, or hazardous drinking
Drug use	Use of drugs for psychotropic rather than medical purposes, potentially including both legal and illegal substances.[17]	Ever used cannabis<br>Illicit drug use<br>Frequency of drug use
Tobacco use	The practice of smoking tobacco and inhaling tobacco smoke.[18]	Ever smoked a cigarette<br>Frequency of tobacco use
Use of electronic nicotine delivery systems (ENDS)	Umbrella term for vapes, vaporisers, vape pens, e-cigarettes, and e-pipes. ENDS are non-combustible tobacco products which use an e-liquid, containing nicotine.[19]	Ever tried an e-cigarette<br>Frequency of e-cigarette use
Sexual risk behaviour	Initiation of sexual activity at an early age, engaging in unnatural or unprotected sexual intercourse, having sexual intercourse with multiple partners, engaging in paid or irregular or incentive-driven sex or sexual intercourse with an injecting drug user or under the influence (especially intoxication) of psychoactive substances, which may result in sexually transmitted infections, unintended/early pregnancies (or abortions), or legal or interpersonal conflicts.[20]	Early age of sexual debut<br>Transactional sex<br>Unprotected sex<br>Sexual intercourse with multiple partners<br>Posting, sharing, or exchanging sexual content using electronic devices
Gambling (not via SM)	Placing something of value (usually but not always money) in hope of acquiring something of greater value.[21]	Problem gambling<br>Pathological gambling<br>Internet gambling (not via social media)
Unhealthy dietary behaviour	Umbrella term referring to all phenomena related to food choice, eating behaviour, and dietary intake/nutrition. Disordered eating not considered[22]	Low level of fruit and vegetable consumption<br>High fat/sugar/salt diet<br>Low-fibre diet
Inadequate physical activity	Doing no or very little physical activity at work, at home, for transport or in discretionary time.[23]	Physically active for <60 minutes per day on <5 days a week<br>Low levels of physical activity
Anti-social behaviour	Any action which violates social norms in ways which reflect disregard for others, or which reflect the violation of another's rights.[16]	Violence<br>Criminal damage<br>Graffiti/vandalism<br>Aggregated assault<br>Assault with or without injury<br>Stealing/theft<br>Carrying a weapon

Appendix 7. Meta-analyses and synthesis without meta-analysis (SWiM) decision rules

The below guidance outlines the decision rules used when selecting datapoints/studies for inclusion in meta-analyses and synthesis without meta-analysis (SWiM).

Duplicate studies (i.e., those looking at the same population, exposure combination, outcomes and during overlapping time period)

- Select study with longest follow up period
- If studies have the same follow up period, select largest (or most representative)
- If studies are the same size, choose the most recent

Where a study includes multiple repeat cross-sectional samples, or investigates multiple study populations from different settings, these should be entered as separate datapoints, however, will be classified under the primary study from which they originate.

There may be instances of duplicate datapoints, but if some datapoints are unique (investigate different outcomes) between studies, the overall study should be retained, and individual duplicate datapoints will be removed at the next stage.

Duplicate/overlapping datapoints

Eight options:
1. Include in meta-analysis
2. Include in stratified analysis (sensitivity/subgroup analysis)
3. Include in meta-analysis and stratified analysis
4. Include in SWiM
5. Include in meta-analysis and SWiM
6. Include in stratified analysis and SWiM
7. Include in all analyses
8. Exclude from all analyses (where sex-stratified datapoints are selected for inclusion and whole sample datapoint is not used)

Selection of datapoints for meta-analysis

- In ensuring independence of data, only one effect size per outcome from each study should be used in each meta-analysis. However, datapoints not included in meta-analyses due to potential double counting of participants may be used within stratified analyses
- Meta-analysis should be performed for each exposure (time spent on social media, frequency of social media use and exposure to health-risk behaviour content on social media), by common metric (standardised beta, standardised mean difference, and odds ratio), and by outcome
- Meta-analysis should be conducted when ≥3 datapoints are available for a specific synthesis
- Meta-analyses should be conducted at the datapoint/outcome level, and all forest plots presented should report the risk of bias grade at the datapoint/outcome level

Exposure

Multiple comparison groups:

Select the largest comparator group, unless this would affect the comparability of results within studies, in which case select the most common/unifying comparison group. Where a common/unifying comparison group cannot be identified for a datapoint, report in SWiM.

Same outcome assessed by multiple exposures:

The most used exposure should be selected (i.e., the exposure for which most studies contribute a result). The below criteria should additionally be considered, in order of importance:
1. Ways of measuring the same construct: select validated rather than subjective/self-report exposure measure
2. Analysis: select exposure pertaining to adjusted estimate (i.e., adjusted for pre-specified critical confounding domains) or estimate which can be meta-analysed

3. Timepoint: select exposure pertaining to datapoint assessing the longest/last occasion measured unless not relevant

Multiple exposure groups from a single datapoint:
Approach taken to overcome a unit of analysis error for a datapoint that could contribute multiple, correlated comparisons, as per Cochrane guidance.[24]
1. Combine exposure groups to create a single pair-wise comparison comparable to other included datapoints in the meta-analysis
2. Select one pair of exposure groups and exclude the others, ensuring the groups selected are comparable with other included datapoints in the meta-analysis

Where a second datapoint originating from the same study, with the same exposure, is identified for inclusion in SWiM, the same groups used in the meta-analysis (via selection of one pair of exposure groups/combing exposure groups to create a single pair-wise comparison) should be used to enhance comparability.

Different exposure periods (e.g., past week, current):
Where possible, ensure exposure periods of datapoints to be included in meta-analysis align. Due to heterogeneity of exposures reported across studies, it is anticipated this may not be possible and each meta-analysis will include datapoints with varying exposure periods.

Outcome
If an adjusted datapoint cannot be converted to a common effect, and summary data or an unadjusted datapoint can be converted to a common effect, use this within meta-analysis. If neither the adjusted/unadjusted datapoint or summary data reported by the study can be converted to a common effect (e.g., change scores, outcome trajectory) report using SWiM.

Multiple outcome measures for the same outcome:
Where studies report multiple measures of the same behaviour (e.g., weekly alcohol consumption, frequency of binge drinking) the most common outcome measure should be selected (i.e., the outcome for which most studies contribute a result). The below criteria should additionally be considered, in order of importance:
1. Ways of measuring the same construct: select validated rather than subjective/self-report outcome measure
2. Analysis: select outcome measure pertaining to adjusted estimate (i.e., adjusted for pre-specified critical confounding domains) or estimate which can be meta-analysed
3. Timepoint: select outcome pertaining to datapoint assessing the longest/last occasion measured unless not relevant

Different outcome periods (e.g., past week, past month use):
Where possible, ensure that outcome periods of datapoints to be included in meta-analysis align. Due to heterogeneity of outcomes reported across studies, it is anticipated this may not be possible and each meta-analysis will include datapoints with varying outcome periods.

Varying time points of follow up for an outcome:
- Use the longest time point/last occasion measured unless not relevant (e.g., sexual intercourse measured at Time 1 (study sample aged 15) should be selected instead of sexual intercourse measured at Time 2 (study sample aged 16) where age of consent is 16 years in study setting)
- Where multiple time points are reported, these should be extracted separately, and sensitivity analysis conducted to explore any differences by study design (cross-sectional vs longitudinal)

Sex
- If sex-stratified datapoints reported, use both
- If whole sample estimate (male and female combined) alongside datapoint for a single sex reported, use the whole sample estimate
- If only one datapoint is reported pertaining to a single sex, use this

Selection of datapoints for SWiM (vote counting based on effect direction)

- Where effect estimates are incompletely reported or where study characteristics such as study design, exposures or outcomes are too diverse to provide a meaningful summary effect estimate, report datapoint using SWiM

- Where exposed and unexposed groups reported in a datapoint do not align with the meta-analysis exposed and unexposed group report using SWiM
- Datapoints reporting trajectory of outcome/change in outcome/change scores should not be used in meta-analyses and should be synthesised using SWiM
- Effect direction synthesis should be performed by exposure (time spent on social media, frequency of social media use, exposure to health-risk behaviour content and other social media activities), and by outcome domain
- Where multiple outcome measures are reported for the same exposure and same outcome, the direction of effects reported across outcome measures should be synthesised using the algorithm proposed by Cochrane,[25,26] based on the proportion of effects which are in a consistent direction. Note it is possible for one study assessing one exposure and one outcome measure to demonstrate an unclear/conflicting/inconsistent effect[26]
- Where age subsets, study populations from different countries, and repeat cross sectional samples are reported in the same study, these should be entered as separate studies for purposes of SWiM, to maximise use of the available data
- SWiM should be conducted at the study level, and all effect direction plots presented, should report the study risk of bias grade

Exposure

Same outcome assessed by multiple exposures:

The most used exposure selected (i.e., the exposure for which most studies contribute a result). The below criteria will additionally be considered, in order of importance:

1. Ways of measuring the same construct: select validated rather than subjective/self-report exposure measure
2. Analysis: select exposure pertaining to adjusted estimate (i.e., adjusted for pre-specified critical confounding domains). If all datapoints fail to adjust for pre-specified critical confounders, consider adjustment for other justifiable confounders
3. Time point: select exposure pertaining to datapoint assessing the longest/last occasion measured unless not relevant

Multiple exposure groups from a single datapoint:

- Where a datapoint is reported in the meta-analysis and a pairwise comparison is selected/exposure groups are combined, when reporting this datapoint in SWiM ensure the same comparison is used to enhance comparability
- If a datapoint is to be reported in SWiM, and similar datapoints (with the same multiple exposure groups) originating from the same study have not been reported in meta-analyses or SWiM, then select a specific pairwise comparison/combine exposure groups, ensuring efforts are made to maximise use of all data, and the comparison aligns with other datapoints reported in SWiM synthesis

Outcome

Multiple outcome measures reported for the same outcome:

- Where there are multiple outcome measures investigating the same outcome, aggregate these using the effect direction algorithm: report direction of effect where ≥70% of outcomes report similar direction. If <70% of outcomes report consistent direction of effect, then report inconsistent findings[26]
- The largest sample size across all aggregated datapoints will be reported in the effect direction plot

Multiple time points presented for an outcome from the same study:

- Where there is a cross-sectional and cohort datapoint originating from same study investigating the exact same exposure and outcome, the direction of effect should be aggregated as above. The study design should be reported as a cohort study, and the associated cohort study risk of bias grade reported within the effect direction plot. The sample size reported should reflect that of the sample used in the cohort datapoint
- If datapoints, are reported for different follow up periods (e.g., time 1- time 2 and time 1- time 3) aggregate the direction of effect for all datapoints as above and report the associated risk of bias grade and sample size for the longest follow up period

Sex

If sex-stratified datapoints are reported, aggregate these when reporting effect direction and discard the whole sample estimate.

Appendix 8. Data extraction form exemplar- study and datapoint level variables

Table A. Data extraction form: study level variables

Field	Brief description	Guidance	Permissible entries
date	Date of data extraction by lead author	Not for completion by second-checker	
source	Where did we find the study?	If this is a relevant publication screened in Covidence	Cov
		If this is a relevant publication found via refence list of systematic review	SR
		If this is a relevant publication identified via manual searching of reference lists of included studies	M
		If this is a relevant publication identified via expert correspondence	E
study_ID	Internal reference number	Source = Covidence	#3343 use the number allocated within Covidence
		Source = Systematic Review	#SR
		Source = Reference list of included studies	#M
		Source = Expert correspondence	#E
second_checker	Name of person doing second checking	To be completed by second-checker	
second_checker_date	Date of second checking	To be completed by second-checker	
first_author	Surname of first author		
year_pub	Year of publication		
published	Where was the study published	If study published in journal	Name of the journal
title	Title of the study		
study_aim	Aim of study	Brief free-text description of the studies aim	
author_contact	Study corresponding author contact details	Insert email of corresponding author	
publication_cat	What type of publication is this?		Journal
			Preprint
length	Was the study cross sectional or longitudinal?	No repeated measures	Cross-sectional
		At least two waves of data collection on the same individuals	Longitudinal
country	Country of study	Where was the study carried out? (including location and social context)	e.g., Toronto Canada (5 Southern Toronto High Schools)
setting	Was the setting classified as a high or middle- or low-income country at the time of the study?	See World Bank Classification: https://datahelpdesk.worldbank.org/knowledgebase/articles/906519. If a study is looking at a range of countries which are a mixture of high- and low-income countries, select all options that apply	High income
			Middle income
			Low income
study_years	When did the study run?	Years in which study ran, including any follow-up. Can be expressed either as a range or a list (if certain years were excluded)	

Field	Brief description	Guidance	Permissible entries
study_design	What kind of study?		Cross-sectional<br>Cohort<br>Randomised trial of intervention<br>Non-randomised trial of intervention<br>Cross-sectional analysis of cohort<br>Cross-sectional analysis of intervention<br>Natural experiment<br>Panel study<br>Repeat cross-sectional<br>Systematic review (primary data not available)
data_source_cat	Was primary or secondary data used in this study?		Primary<br>Secondary
data_source	If secondary data used, what is the name of the data source?	Insert name of data source	
recruitment_ strategy	Recruitment setting/strategy	Free-text description of how participants were reached and recruited into study (e.g., sampling frame, sampling technique, location)	
data_collection	Study data collection method	Free-text description of how and where data was collected from participants (e.g., online survey in home setting, paper survey conducted in classroom, telephone survey)	
response_rate_%	Response rate as percentage	Those who completed baseline/those invited to participate	
inc_criteria	Record study inclusion criteria	Description covering initial inclusion criteria for participation and analytical sample (if we are interested in a subset of the whole sample)	
total_ participants	Total number of study participants in whole sample and analytical sample (if available)	If not reported for analytical sample extract for whole sample	
average_age	Average age of whole sample and analytical sample (if available)	If not reported for analytical sample extract for whole sample	Report mean if available
age_range1	Age range (indicator of spread) for whole sample and analytical sample (if available)	If not reported for analytical sample extract for whole sample	Report standard deviation if available
age_range2	Sample age range covered in whole sample and analytical sample (if available)	If not reported for analytical sample extract for whole sample	Report range if available
percent_male	What percentage of the whole sample and analytical sample (if available) were male?	If not reported for analytical sample extract for whole sample	
ethnicity	What is the ethnicity of study participants in the whole sample and analytical sample (if available)	If not reported for analytical sample extract for whole sample	
sep	What is the socioeconomic position of the whole sample and analytical sample (if available)	Record the scale/measure used and the distribution amongst study participants in the analytical sample. If not reported for analytical sample extract for whole sample	
conflicts_of_ interest	Any possible conflicts of interest?		Yes<br>No<br>Not reported (NR)

Field	Brief description	Guidance	Permissible entries
funding_source	Was the study funded by an organisation?	If yes	Record the name of funding bodies
		If no	No
		If not reported	Not reported (NR)
ethical_approval	Was ethnical approval obtained for the study?	If yes	Yes
		If no	No
		If not reported	Not reported (NR)
		If not required (e.g., secondary data)	Not required
study_notes	Anything else worth recording in relation to study information		

Table B. Data extraction form: datapoint level variables

Field	Brief description	Guidance	Permissible entries
dpID	Internal reference number for datapoint		
dpnum	Datapoint number		
fu_length	What was the length of follow up for this specific datapoint?	If cross-sectional study	Not applicable (NA)
		For longitudinal studies, what was the length of follow-up for the specific datapoint?	Number of months/years
sg_gender/sex	For subgroup analysis, what is the gender/sex of the analytical sample this datapoint relates to?	If sex reported, select either Sex Male, Sex Female, Sex both. If gender reported, select either Gender Male, Gender Female, Gender Both	Gender Male
			Gender Female
			Gender Both
			Sex Male
			Sex Female
			Sex Both
sg_age	For subgroup analysis, what is the average age of the analytical sample this datapoint relates to?	If not available for analytical sample, report for whole sample	
sg_sep	For subgroup analysis, what is the socioeconomic position (SEP) of the analytical sample this datapoint refers to?	If not available for analytical sample, report for whole sample	
		If only includes those with low baseline SEP	Low SEP
		If only includes those on high baseline SEP	High SEP
		If includes a mix of low and high baseline SEP	Mixed- general population
sg_setting	For subgroup analysis what is the World Bank grouping of the country this datapoint refers to at the time of the study?	If not available for analytical sample, report for whole sample	High income
			Middle income
			Low income
exp_def	How was the exposure defined within the study?	Authors' description of social media use as per methods	e.g., frequency of social media networking site use, daily time spent using social media (hours/day)
exp_duration	When did data collection for the exposure occur?	Record when data collection for exposure occurred	
time_period_exposure	What time period was the exposure measuring?	Record the time period for which the exposure measures	e.g., ever, current

Field	Brief description	Guidance	Permissible entries
sg_exp_cat	What social media category is under study for the datapoint?	Record which social media category is examined for specific data point.<br>A number of social media platforms will fall under several social media types, for example, Twitter is a social networking site and a microblogging site.<br>If study authors state the specific type of social media record this, if they do not, make an assessment and record the social media type you think best represents the social media platform(s) under study, considering the initial premise of the platform.<br>Where it is impossible to determine which category is under study, select "Social Media" from the drop-down list	Social networking sites (e.g., Facebook, Whats App, Snapchat, Myspace, Whisper, Instant messaging)
			Microblogging sites (e.g., Twitter, Tumblr)
			Blogs and forums (e.g., Wordpress, Live journal, discussion boards, pin boards)
			Media-sharing sites (e.g., YouTube, Pinterest, Instagram)
			Geo-location- based sites (e.g., Foursquare, Tinder, Grindr)
			Book marking sites (e.g., Delicious, Twitter, StumbleUpon)
			Social news sites (e.g., Reddit, Digg)
			Collaborative authoring sites (e.g., Wikipedia, Google Docs)
			Web conferencing (e.g., Skype, zoom)
			Scheduling and meeting (e.g., Microsoft outlook, Doodle, Google Calendar)
			Online gambling
			Online gaming
			Online gaming
			Social media
exp_platform	What social media platform is under study for the datapoint?	Record the specific social media platforms under study (e.g., Facebook, Reddit) for the specific datapoint or record the examples provided in relation to the datapoint under investigation	e.g., Facebook specified<br>e.g., Examples: Facebook, Twitter
sg_exp_content	What type of social media content is understudy for the datapoint?	If user-generated content (e.g., content produced by the user, friends, others in the social media network)	User-generated
		If marketer-generated content (e.g., advertisements & influencer content)	Marketer-generated
		If content is both user-generated and marketer-generated content	User and Marketer-generated
		If social media content is not specifically under investigation (e.g., time spent on social media/frequency of social media use) and we cannot distinguish what type of content the participant is exposed to	Not applicable (NA)
exp_ascertain	How was social media use measured for the datapoint?	Free-text description of measurement tool/instrument (e.g., specific scale, survey question, objective measures of social media usage tracked by mobile phones/electronic devices)<br>For a scale, provide the name of the scale, upper and lower limits, and whether a high or low score is favourable and state definitions of any thresholds if appropriate<br>For survey questions, state the name of the survey, question, if it is self-report (or if a proxy has been used state this), question response options, whether a high or low score is favourable, and definitions of any thresholds/categories created if appropriate<br>Record if objective/validated/self-report measure	

Field	Brief description	Guidance	Permissible entries
exp_measure_ type	What type of measurement is the exposure?		Binary<br>Continuous<br>Categorical<br>Ordinal
exp_mean	Mean of exposure measure	Mean and/or proportion (n/%) of analytical sample or whole sample if not available<br>If exposure is ordinal/categorical, record number of those exposed in each exposure group	
exp_SD	Standard deviation of exposure measure		
outcome_ domain	What outcome does the datapoint report on?	Note 'multiple risk behaviours' should only be used where the analysis has specifically looked at 'multiple risk behaviours' as a single outcome (2 or more of the individual risk behaviours under investigation)	Alcohol use<br>Tobacco use<br>Drug use<br>Use of ENDS<br>Unhealthy dietary behaviour<br>Inadequate physical activity<br>Antisocial behaviour<br>Gambling<br>Sexual risk behaviour<br>Multiple risk behaviours
outcome_def	How was the outcome defined within the study?	Authors' description of outcome as per methods	e.g., frequency of drinking alcohol
outcome_ duration	When did data collection occur for the outcome?	Record when data collection occurred for outcome	e.g., 2004 (wave 2)
time_period_ outcome	What time period was the outcome measuring?	Record the time period for which the outcome measures	e.g., ever, current
outcome_ acertain	How was the outcome measured for the datapoint?	Free-text description of measurement tool/instrument<br>For a scale, provide the name of the scale, upper and lower limits, and whether a high or low score is favourable and definitions of any thresholds if appropriate<br>For survey questions, state the name of the survey, question, if it is self-report (or if a proxy has been used state this), question response options, whether a high or low score is favourable, and definitions of any thresholds/categories created if appropriate<br>Record if validated tool/medical records/self-report/independent blind assessment	e.g., AUDIT-C. Response categories...
outcome_ measure_type	What type of measurement is the outcome?		Binary<br>Categorical<br>Continuous<br>Ordinal

Field	Brief description	Guidance	Permissible entries
outcome_mean	Mean of outcome measure	Mean and/or proportion (n/%) of analytical sample or whole sample if not available<br>If exposure is ordinal/categorical, present number of those with outcome in each exposure group	
outcome_SD	Standard deviation of outcome measure		
analytical_ sample	Number of participants used in the analytical sample for the datapoint<br>Present for whole sample if possible		
dp_measure	What effect measure is reported for the datapoint?	Where possible record adjusted measures for data extraction purposes if unavailable record unadjusted estimates<br>Where both adjusted and unadjusted measures are presented, record adjusted estimates in data extraction form and state unadjusted estimates are available<br>If outcome measure is not listed, input as free text	Correlation coefficient (Pearson's)<br>Correlation coefficient (Spearman's)<br>Correlation coefficient (Point-biserial)<br>Correlation coefficient (Phi)<br>Standardised path coefficient (adjusted)<br>Standardised path coefficient (unadjusted)<br>Unstandardised path coefficient (adjusted)<br>Unstandardised path coefficient (unadjusted)<br>Standardised regression coefficient (adjusted)<br>Standardised regression coefficient (unadjusted)<br>Unstandardised regression coefficient (adjusted)<br>Unstandardised regression coefficient (unadjusted)<br>Standardised linear regression coefficient (adjusted)<br>Standardised linear regression coefficient (unadjusted)<br>Unstandardised linear regression coefficient (adjusted)<br>Unstandardised linear regression coefficient (unadjusted)<br>Odds ratio (adjusted<br>Odds ratio (unadjusted)<br>Risk Ratio (adjusted<br>Risk Ratio (unadjusted)<br>Mean<br>Median<br>Chi square<br>F-statistic<br>T-statistic<br>Raw summary data extracted
dp_analysis_ type	What type of analysis was conducted?	Insert brief statement on analysis method	
analysis_desc	Describe the analysis used for investigation of the datapoint as per methods	Free-text description of analysis method used	

Field	Brief description	Guidance	Permissible entries
incomplete_ outcome_ data	Was there any missing data (e.g., unit & item missingness)? How was this managed? Were sampling/non-response weights used?	Describe the completeness of outcome data for each data point, including attrition (e.g., loss to follow up, withdrawn, non-response) and exclusions from the analysis. Record if missing data handled appropriately or if weights (e.g., non-response and selection) were employed	
dp_adjustment	If applicable, what confounders were adjusted for?	If adjusted estimates are presented, record all confounders controlled for	
mediators_effect modifiers	Were mediators/effect modifiers investigated?	State if mediator/moderator investigated Record the name of the mediator/moderator	e.g., mediator: self-esteem
exp_group	What is the exposed group?		
dp_point_est	The datapoint estimate of interest		
n_numerator	People in numerator (with outcome) for group of interest	If available	
n_denominator	People in denominator for group of interest	If available	
other_denom	Other denominator	Use this to record the analytical sample or total number of individuals exposed for continuous exposures	
lower_ci	Lower 95% confidence interval (CI) of main point estimate (if applicable)		
upper_ci	Upper 95% CI of main point estimate (if applicable)		
sd_se	Standard deviation/standard error (SE) of point estimate (if applicable)	If SE provided, note and mark (e.g., SE=)	
t_z_stat	T or Z statistic (if applicable)		
p_value	p value of main point estimate		
sig_5%	Is the point estimate of interest significant at 5% level?	Record if significance level has been set at a level other than 5%	Yes
			No, significance level set at (XXX)
comp_group	Description of comparator group	If exposure measure is continuous or point estimate is a correlation, state 'Baseline' here to demonstrate the comparator group is those people with a different level of exposure than the exposed group	e.g., low social media use (<2 hours per day).
datapoint_notes	Location of data point extracted & study author reporting	Report the location of datapoint, numerators and denominators extracted within each study Record any issues regarding study author reporting	
comp_point_est	Point estimate for comparator group		
comp_ numerator	People in numerator (with outcome) for comparator group estimate	If available	
comp_ denominator	People in denominator for comparator group estimate	If available	
other_denom	Other denominator		
comp_lower_ci	Lower 95% CI of comparator estimate (if applicable)		
comp_upper_ci	Upper 95% CI of comparator estimate (if applicable)		

Field	Brief description	Guidance	Permissible entries
comp_sd_se	Standard deviation/standard error of comparator estimate (if applicable)	If SE provided, note and mark (e.g., SE=)	
comp_t_z_stat	T or Z statistic if applicable of comparator estimate (if applicable)		
comp_pvalue	p value of comparator estimate		
comp_sig5%	Is p value significant at 5% level?	Record if significance level has been set at a level other than 5%	Yes
			No, significance level set at (XXX)
comp2…	Fields for second comparator group - replicate those for the first		
other_pot_ relevant_ datapoints	Were other potentially relevant estimates reported for the exposure-outcome combination (datapoint) investigated?	If other relevant estimates recorded e.g., correlations/standardised estimates, record the estimates and state their location in paper<br>If unadjusted estimates provided state this	
correspondence_ required	Do you need to contact the study authors for any reason (e.g., accessing original data, requesting additional info, verifying study details)?	Add information on required author correspondence	
akp_notes		Lead author to insert any notes to aid interpretation/support data extracted	
secondchecker_notes		Second checkers to insert any notes to aid interpretation/support data extracted	

Appendix 9. Newcastle-Ottawa Scale (NOS) risk of bias assessment

For cross-sectional and cohort studies, an adapted version of the Newcastle-Ottawa Scale (NOS) for assessing risk of bias (RoB) in non-randomised studies was used.[27] This appendix presents the adapted NOS used when assessing RoB of non-randomised studies and the algorithms used when assessing domain level and overall RoB of included datapoints. To ensure a standardised process to NOS RoB assessment, a detailed guidance document prepared by AKP was circulated to the review team. Those studies reporting baseline data from an interventional study were appraised as per cross-sectional studies. RoB assessment was conducted at the datapoint/outcome level. An overall RoB grade was assigned to each study through consideration of the most commonly reported RoB grade across included datapoints from a study, prioritising the overall grade assigned to datapoints which were investigated via the primary analysis conducted in a study.

Adapted Newcastle Ottawa Scale (NOS): cross-sectional studies

Used when assessing cross-sectional studies, cross-sectional analysis of cohort studies, cross-sectional analysis of intervention studies and repeat cross-sectional studies

Domain A - Selection

Selection - Representativeness of original sample
- a) Good representativeness of the target population (e.g., all subjects, random sampling)
- b) Selection process does not ensure representativeness, but it is clearly described (e.g., non- probability sampling)
- c) Selected group of users, with the potential for selection bias
- d) No description

Selection - Non-respondents
- a) Comparability between respondents and non-respondents’ characteristics established and/or response rate ≥75% of original sample and (if applicable) those with and without missing data established and adjusted for
- b) Comparability between respondents and non-respondents is unsatisfactory or response rate <75% or (if applicable) missing data addressed inappropriately
- c) No description or unclear

Figure A. Algorithm to assess the Selection domain of the adapted Newcastle Ottawa Scale for cross-sectional studies

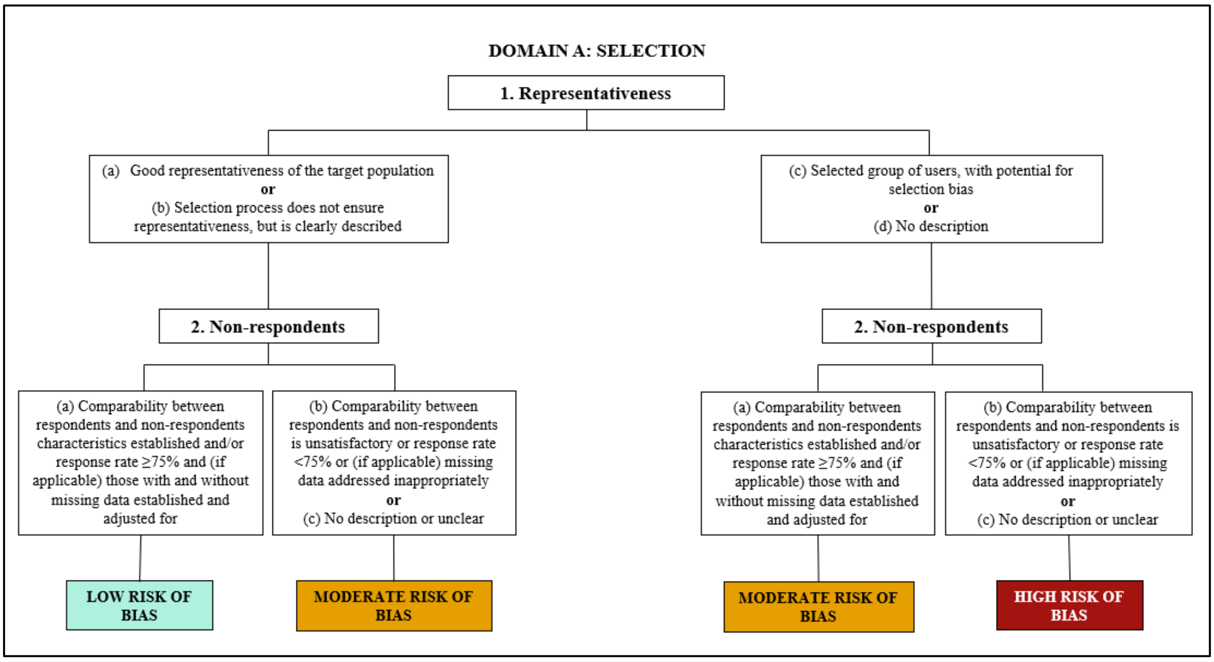

Domain B - Exposure

Exposure - Ascertainment of exposure

a) Objectively recorded social media usage data, independent of user reports
b) Validated measurement tool
c) Non-validated measurement tool, but the tool is available or described
d) No description or unclear

Figure B. Algorithm to assess the Exposure domain of the adapted Newcastle Ottawa Scale for cross-sectional studies

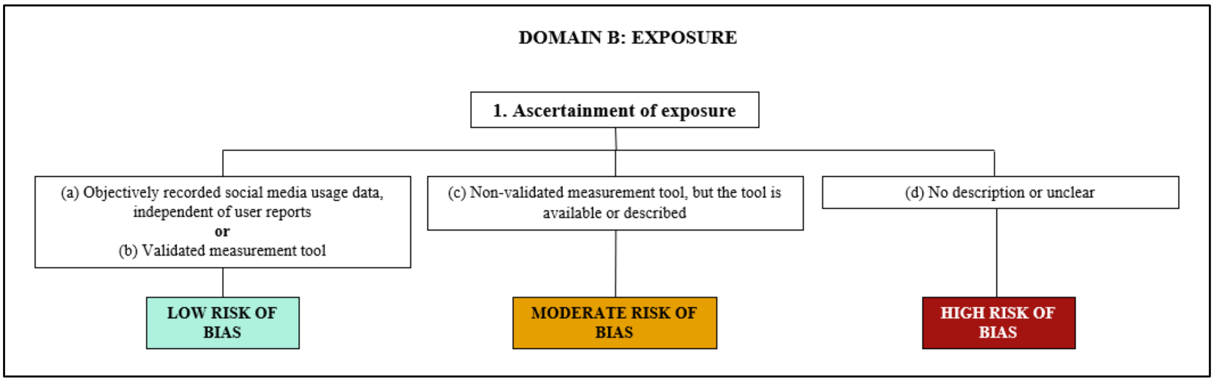

Domain C - Comparability

Comparability - Based on analysis of interest. Confounding factors are controlled.

(a) The study controls for key confounders age, sex/gender, and socioeconomic position (e.g., parental educational attainment, employment, income, area-level deprivation)
(b) The study controls for key confounders age, sex/gender, and socioeconomic position (e.g., parental educational attainment, employment, income, area-level deprivation) **and** the study controls for an alternative set of justifiable confounders (e.g., sensation seeking, peer influence or proxy measures for age, sex/gender, or socioeconomic position)
(c) The study controls for an alternative set of justifiable confounders (e.g., sensation seeking, peer influence or proxy measures for age, sex/gender, or socioeconomic position)
(d) No adjustment for potential confounders, no description or unclear

Figure C. Algorithm to assess the Comparability domain of the adapted Newcastle Ottawa Scale for cross-sectional studies

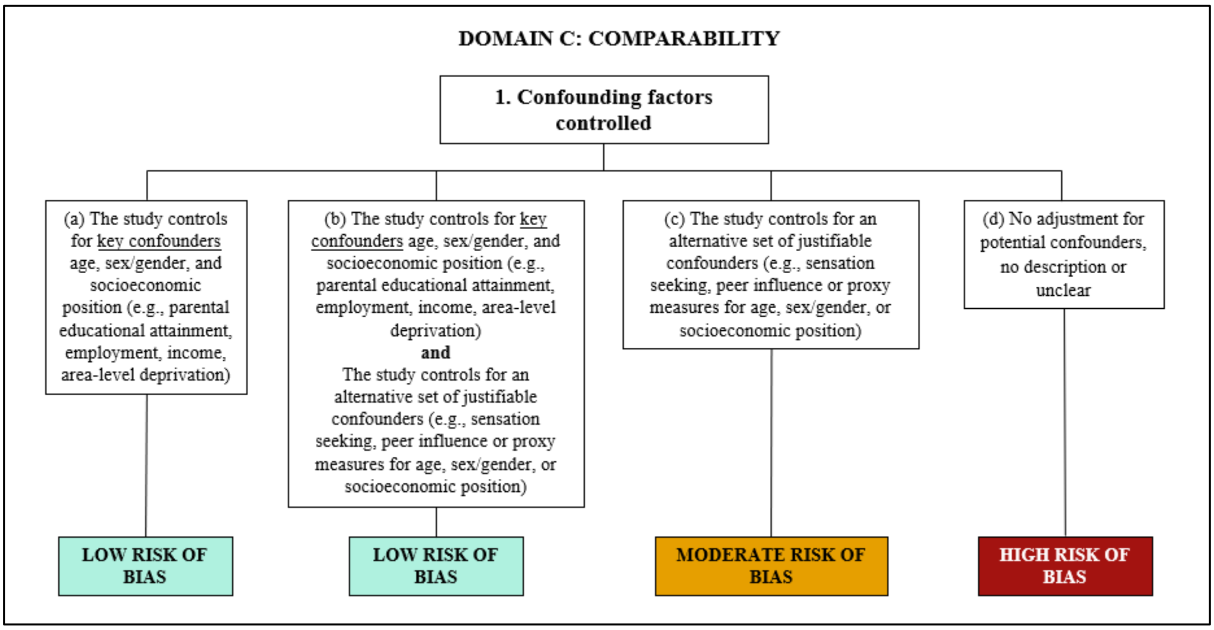

Domain D - Outcome

Outcome- Assessment of outcome
  (a) Independent clinical assessment or validated measurement tool
  (b) Medical/administrative records
  (c) Self-report
  (d) No description, or other inadequate

Figure D. Algorithm to assess the Outcome domain of the adapted Newcastle Ottawa Scale for cross-sectional studies

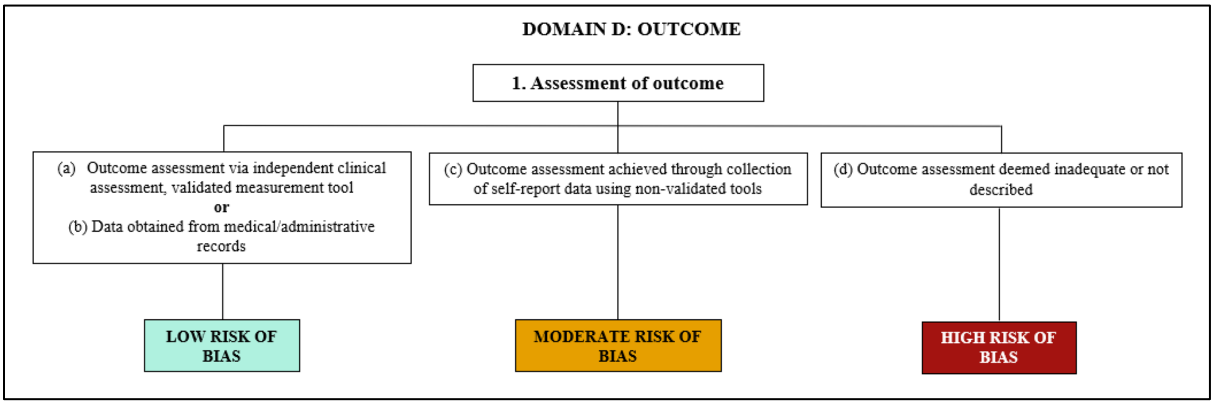

Adapted Newcastle Ottawa Scale (NOS): cohort studies

Used when assessing cohort and panel studies

Domain A - Selection

Selection - Representativeness of the original sample
- a) Good representativeness of the target population (e.g., all subjects, random sampling)
- b) Selection process does not ensure representativeness, but it is clearly described (e.g., non- probability sampling)
- c) Selected group of users, with the potential for selection bias
- d) No description

Selection - Selection of the comparator group
- a) Drawn from the same community as the exposed cohort
- b) Drawn from a different source
- c) No description of the derivation of the non-exposed cohort

Figure E. Algorithm to assess the <u>Selection</u> domain of the adapted Newcastle Ottawa Scale for cohort studies

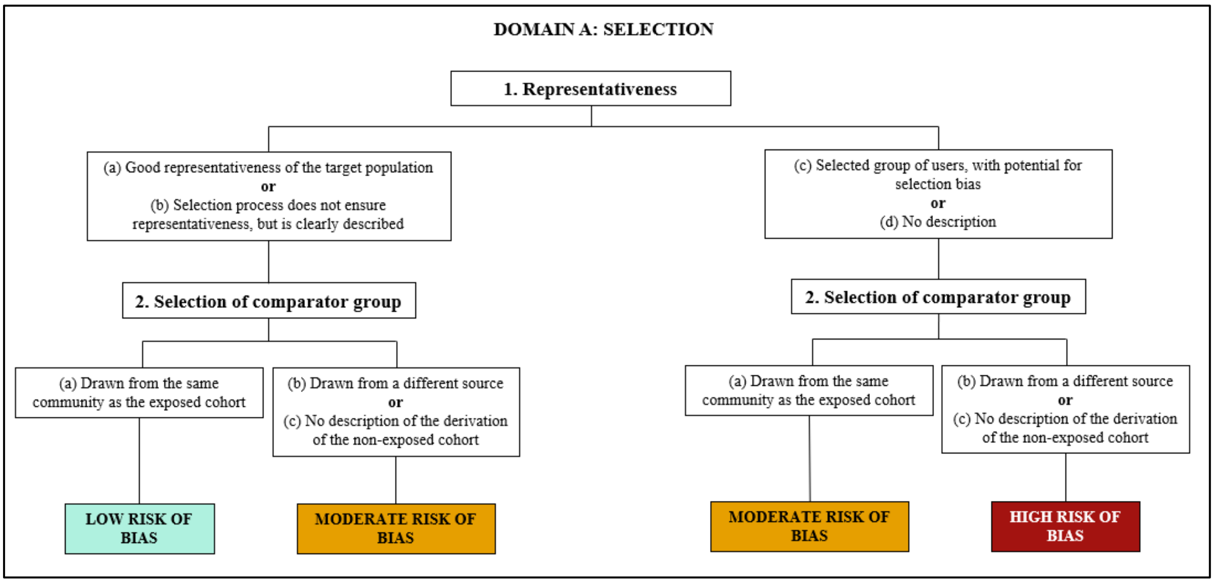

Domain B - Exposure

Exposure - Ascertainment of exposure
- a) Objectively recorded social media usage data, independent of user reports
- b) Validated measurement tool
- c) Non-validated measurement tool, but the tool is available or described
- d) No description or unclear

Figure F. Algorithm to assess the <u>Exposure</u> domain of the adapted Newcastle Ottawa Scale for cohort studies

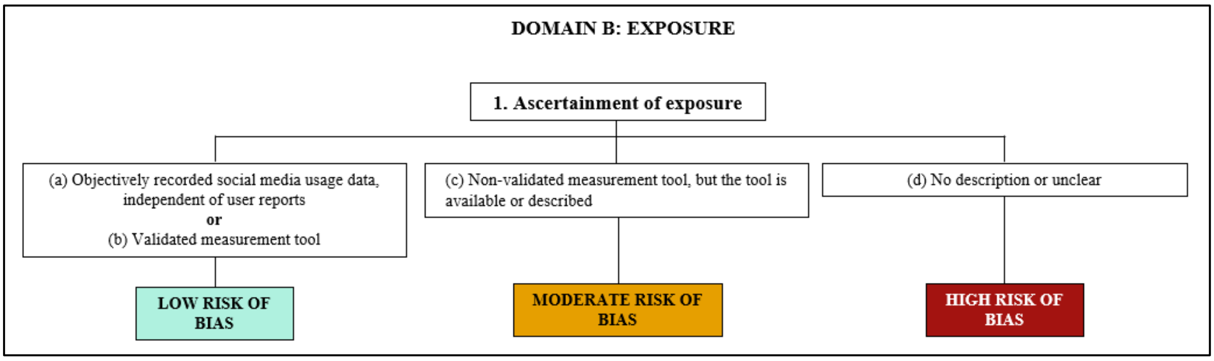

Domain C - Comparability

Comparability - Based on analysis of interest. Confounding factors are controlled.

a) The study controls for <u>key confounders</u> age, sex/gender, and socioeconomic position (e.g., parental educational attainment, employment, income, area-level deprivation)
b) The study controls for <u>key confounders</u> age, sex/gender, and socioeconomic position (e.g., parental educational attainment, employment, income, area-level deprivation) **and** the study controls for an alternative set of justifiable confounders (e.g., sensation seeking, peer influence or proxy measures for age, sex/gender, or socioeconomic position)
c) The study controls for an alternative set of justifiable confounders (e.g., sensation seeking, peer influence or proxy measures for age, sex/gender, or socioeconomic position)
d) No adjustment for potential confounders, no description or unclear

Comparability - Accounts for baseline measure of outcome

a) Yes
b) No

Figure G. Algorithm to assess the <u>Comparability</u> domain of the adapted Newcastle Ottawa Scale for cohort studies

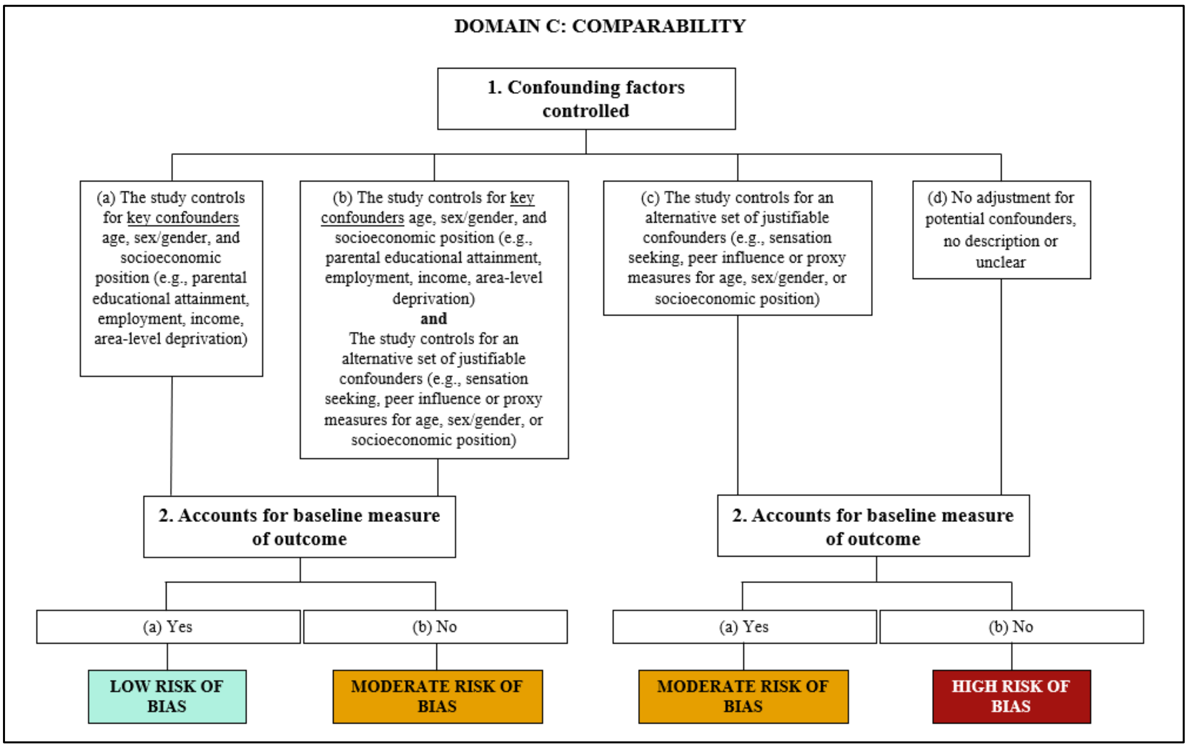

Domain D - Outcome

Outcome - Assessment of outcome
(a) Independent clinical assessment or validated measurement tool
(b) Medical/administrative records
(c) Self-report
(d) No description, or other inadequate

Outcome - Adequacy of follow up
a) Complete follow up (on all relevant variables)- all subjects accounted for
b) Subjects lost to follow up or due to missing data <25%, unlikely to introduce bias, or accounted for using weights, imputation etc.
c) Loss to follow up substantial (≥25%) and/or likely to introduce bias
d) Not described or unclear

Figure H. Algorithm to assess the <u>Outcome</u> domain of the adapted Newcastle Ottawa Scale for cohort studies

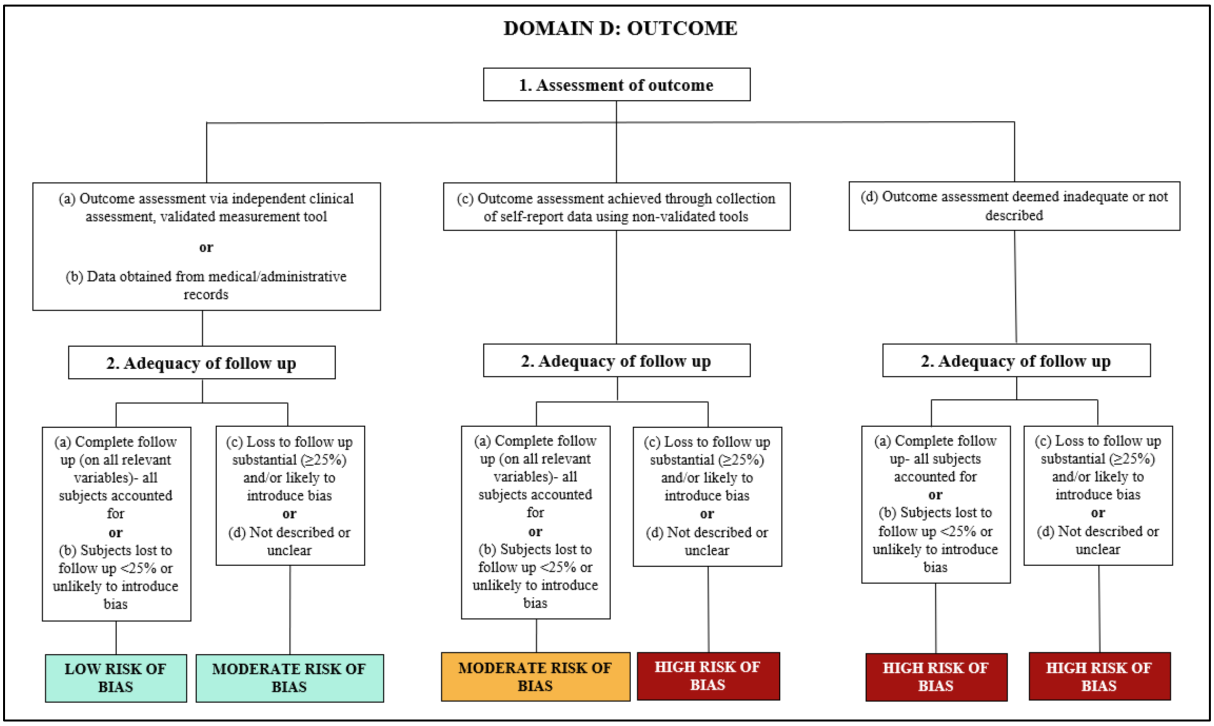

Assessing domain level and overall risk of bias (RoB) for included datapoints

For both cross-sectional studies and cohort studies, the algorithms presented above were used to grade each domain using the response options selected for each signalling question. Each domain was allocated either a low, moderate, or high RoB grade as illustrated in Table A.

Table A. Available domain level risk of bias (RoB) grades

Domain	Risk of bias judgement		
Selection	Low risk of bias	Moderate risk of bias	High risk of bias
Exposure	Low risk of bias	Moderate risk of bias	High risk of bias
Comparability	Low risk of bias	Moderate risk of bias	High risk of bias
Outcome	Low risk of bias	Moderate risk of bias	High risk of bias

Once all domains were graded, Table B was used to reach an <u>overall RoB judgement for each datapoint</u> using the grades applied for each domain.

Table B. Algorithm to classify overall risk of bias (RoB) grade

**Overall risk of bias judgement**	**Criteria**
Low risk of bias	Study is not judged to be at high risk of bias for any domain **and** is judged to be at low risk of bias for either the Exposure or Comparability domain
Moderate risk of bias	Study does not meet criteria for either High risk of bias or Low risk of bias
High risk of bias	Study is judged to be at high risk of bias in at least one domain

Appendix 10. Process for data transformations for meta-analysis

Data transformations were conducted according to guidance within the Cochrane Handbook,[28,29] and using the Campbell Collaboration online effect size calculator.[30]

Since most reported outcomes for binary exposures were binary, statistical approaches were conducted to re-express continuous outcome data as odds ratios (ORs) as per the Cochrane Handbook, thus allowing binary and continuous outcome data to be combined.[28–31] For continuous outcomes, data were pooled to produce standardised beta coefficients (Std. Beta) or standardised mean differences (SMDs).[28–31] For continuous exposures - which are infrequently reported in systematic reviews, meaning best practice recommendations are not available within the Cochrane Handbook,[29] and the difficulties in interpretation of such a synthesis, we opted not to combine continuous and binary outcome data. Instead, continuous exposure and continuous outcomes were pooled separately as standardised beta coefficients or standardised mean differences (SMDs).[28–31] Continuous exposure and binary outcomes were pooled separately as ORs.

Beta coefficients were converted to standardised beta coefficients. Pearson correlation coefficients were converted to standardised mean differences adopting the method outlined by Mathur and VanderWeele.[2] Here, where studies failed to report the standard deviation (SD) of the exposure, efforts were made to contact study authors to obtain the information required for transformation. Where this was not possible, as recommended a substitute estimate was extracted from a second comparable included study (n = 1) or subsample of the study used to estimate *r* and the N term within the formula replaced with the size of the second sample used to estimate the exposure SD.[2]

Presenting multiple exposure groups for one study (for example, if there was a shared reference group) means that multiple, correlated comparisons would result and therefore standard errors could be underestimated. Efforts were therefore made to combine groups to create a single pairwise comparison as recommended by Cochrane.[24] This was only possible when included studies reported the required raw data (e.g., sample sizes, number of participants with the outcome in each exposure group) for all exposure groups. Where this was not possible one pair of exposure groups was selected and the others excluded. Groups were combined/selected ensuring comparability with other included datapoints within the meta-analysis, as per the meta-analysis decision rules presented in Appendix 7. For subgroup analysis, in some instances the analysis includes datapoints not used within primary meta-analysis due to potential double counting of study participants. For example, where multiple datapoints were reported within the same study assessing different social media categories/platforms/content, they were included within separate subgroups.

As per the Cochrane Handbook, where studies reported a p value of <0.05 in the absence of the exact value and this was required to determine the standard error of an estimate, to facilitate inclusion in the meta-analysis, the p-value was assumed to be 0.05.[28] Where data were insufficiently reported for standardisation or transformation, study investigators were contacted by email (n= 6 responses received).

Appendix 11. Characteristics of included studies

Table A. Characteristics of included studies (n=126 studies; 338 datapoints)

Author and year	Study design	Study period	Country	Equity	Participants	Mean age [range]	Risk of bias	N°. of dp	MA?	Exposure	Exposure measure	Outcome measure	N
Anastario 2020[32]	Cross-sectional	NR	USA	High income country	Youth attending 5 schools located on or near a tribal reservation in Montana	15.7 [14-18]	Mod	2	Yes	Freq. of SM use	Freq. of using Twitter to talk or learn about sex or any topic related to sex	No use of a condom at last sexual encounter	146
										Freq. of SM use	Freq. of using Facebook to talk or learn about sex or any topic related to sex	No use of a condom at last sexual encounter	146
Baker 2016[33]	Cross-sectional	2009	USA	High income country	Grade 6-12 urban school district students’ part of a federally funded project on school related initiatives	NR	High	3	Yes	Freq. of SM use	Freq. of SNS use	Soft drug use (smoking, marijuana, alcohol) in the past month	3,195
										Freq. of SM use	Freq. of SNS use	Hard drug use (lifetime and past year)	3,195
										Freq. of SM use	Freq. of SNS use	Weapon carrying in the past month	3,195
Baldwin 2018[34]	Cross-sectional	2014	Australia	High income country with mixed SEP	Adolescents residing in New South Wales	NR [10-16]	Low	7	Yes	Exposure to health-risk behaviour content	Watched food/beverage brand YouTube videos	Freq. of unhealthy food consumption	417
										Exposure to health-risk behaviour content	Seen favourite food advertised on SM	Freq. of unhealthy food consumption	417
										Exposure to health-risk behaviour content	Liked a food/beverage brand on Facebook	Freq. of unhealthy food consumption	204
										Exposure to health-risk behaviour content	Seen favourite food advertised on SM	Freq. of unhealthy drink consumption	417
										Exposure to health-risk behaviour content	Seen favourite food advertised on SM	Freq. of unhealthy food & drink consumption	407
										Freq. of SM use	Freq. of logging in, or checking Facebook account	Freq. of unhealthy food consumption	204
										Freq. of SM use	Freq. of logging in, or checking Facebook account	Freq. of unhealthy drink consumption	204

Author and year	Study design	Study period	Country	Equity	Participants	Mean age [range]	Risk of bias	N°. of dp	MA?	Exposure	Exposure measure	Outcome measure	N
Ball 2020[35]	Repeat cross-sectional	2016 & 2018	New Zealand	High income country with mixed SEP	Year 10 students' part of the Youth Insights Survey	NR [14-15]	Low	2	Yes	Freq. of SM use	Freq. of using SM (status updates, uploading photos or videos) in the past week	Current smoking (defined as smoking at least monthly)	5,127
										Freq. of SM use	Freq. of online gambling in the past week	Current smoking (defined as smoking at least monthly)	5,127
Baru 2020[36]	Cross-sectional	2019	Ethiopia	Low-middle income country with mixed SEP	Sexually active unmarried young female internal migrants residing in Barayu Town	18.9 [15-24]	High	1	Yes	Freq. of SM use	Freq. of SM use	Risk sexual behaviour (incl. multiple sexual partners; sex without condoms or inconsistent condom use; initiation of sex before the age of 18 years; sexual intercourse under the influence of substances)	150
Baumgartner 2012[37]	Cohort	2018	Netherlands	High income country	Adolescents	14.5 [12-18]	Mod	1	Yes	Freq. of SM use	Freq. of online communication	Online sexual risk behaviours (incl. searching for someone on the internet to have sex with; sending a photo or video in which they were partly naked to someone they knew only online)	1,345
Bayraktar 2007[38]	Cross-sectional	NR	Cyprus	HIC	Elementary and high school students residing in North Cyprus	14.4 [NR]	High	1	No	Exposure to health-risk behaviour content	Online gaming: fighting games	Anti-social aggression	686
Beebe 2004[39]	Cross-sectional	2001	USA	High income country	Grade 9 school students' part of the Minnesota Student Survey	14.7 [13-17]	High	12	Yes	Freq. of SM use	Presence of internet chat room use	Tobacco use in the past year	40,376
										Freq. of SM use	Presence of internet chat room use	Alcohol/ drug use in the past year	40,376
										Freq. of SM use	Presence of internet chat room use	Sexual intercourse ever	40,376
										Freq. of SM use	Presence of internet chat room use	Physical assault in the past year	40,376
										Freq. of SM use	Presence of internet chat room use	Vandalism in the past year	40,376
										Freq. of SM use	Presence of internet chat room use	Truant in the past month	40,376
Boers 2020[40]	Cohort	NR	Canada	High income country with low SEP	Grade 7 school students, part of the Co-Venture Preventure study	12.7 [NR]	Low	1	Yes	Time spent on SM	Time spent on SM per day	Freq. of alcohol consumption	3,612

Author and year	Study design	Study period	Country	Equity	Participants	Mean age [range]	Risk of bias	N°. of dp	MA?	Exposure	Exposure measure	Outcome measure	N
Boniel-Nissim 2022[41]	Cross-sectional	2017-2018	42 countries and regions across Europe, North America, and the Middle East	High income country with mixed SEP	School students, part of the Health Behaviour in School-aged Children Survey	13.6 [11-15]	Low	4	Yes	Freq. of SM use	Freq. of online contact with others via SM *(via validated tool)*	Smoking (≥1 × in the last month) *(via validated tool)*	173,577
										Freq. of SM use	Freq. of online contact with others via SM *(via validated tool)*	Alcohol consumption (≥3 × in the last month) *(via validated tool)*	172,723
										Freq. of SM use	Freq. of online contact with others via SM *(via validated tool)*	Drunkenness (≥1 × in the last month) *(via validated tool)*	171,320
										Freq. of SM use	Freq. of online contact with others via SM *(via validated tool)*	Cannabis use (≥1 × in the last month) *(via validated tool)*	55,956
Booker 2015[42]	Cross-sectional	2009	UK	High income country with mixed SEP	Sample members of the youth panel of The UK Household Longitudinal Study	NR [10-15]	High	1	No	Time spent on SM	Time spent chatting on social websites on a normal school day	Sports participation	4,899
Brunborg 2019[43]	Cohort	2014-2015	Norway	High income country	Grade 8-10 and 1-2nd year high school students' part of the pilot Monitoring Young Lifestyles Project	15.2 [13-17]	Low	4	No	Time spent on SM	Change in hrs of SM use per day (Δ=T2–T1)	Change in episodic heavy drinking freq.	763
										Time spent on SM	Change in hrs of SM use per day (Δ=T2–T1)	Change in conduct problems *(via SDQ)*	763
										Time spent on SM	Average number of hrs spent on SM per day in the past year	Episodic heavy drinking freq. in the past year	763
										Time spent on SM	Average number of hrs spent on SM per day in the past year	Conduct problems in the past year *(via SDQ)*	763
Brunborg 2022[44]	Cohort	2017-2020	Norway	High income country with mixed SEP	Middle school adolescents' part of the MyLife Study	14.3 [12.8-16.8]	Mod	1	No	Time spent on SM	Average number of hrs spent on SM per day	Change in alcohol use *(via AUDIT-C)*	3,096

Author and year	Study design	Study period	Country	Equity	Participants	Mean age [range]	Risk of bias	N°. of dp	MA?	Exposure	Exposure measure	Outcome measure	N
Camenga 2018[45]	Cohort	2013-2014	USA	High income country with mixed SEP	High and middle school students' part of a longitudinal school-based cohort study	14.1 [NR]	High	4	Yes	Exposure to health-risk behaviour content	Exposure to e-cigarette advertisements on Facebook	Ever e-cigarette use	1,742
										Exposure to health-risk behaviour content	Exposure to e-cigarette advertisements on Twitter	Ever e-cigarette use	1,742
										Exposure to health-risk behaviour content	Exposure to e-cigarette advertisements on YouTube	Ever e-cigarette use	1,742
										Exposure to health-risk behaviour content	Exposure to e-cigarette advertisements on Pinterest/Google +	Ever e-cigarette use	1,742
Canale 2016[46]	Cross-sectional	2013	Italy	High income country with mixed SEP	High school students' part of the European School Survey Project on Alcohol and Other Drugs Italia	17.2 [15-19]	Low	3	Yes	Freq. of SM use	Freq. of using internet for leisure activities (e.g., online chatting)	Problem gambling *(via SOGS-RA)*	14,478
										Freq. of SM use	Freq. of online gambling in past year	Problem gambling *(via SOGS-RA)*	14,478
										Freq. of SM use	Freq. of using internet for leisure activities (e.g., online chatting)	At-risk gambling *(via SOGS-RA)*	14,478
Casaló 2022[47]	Cross-sectional	2016-2017	Spain	High income country with mixed SEP	Secondary education students, part of the National Survey on Drug Use Among High School Students in Spain	NR [14-18]	Low	4	Yes	Time spent on SM	Time spent on SNS per day	Sports frequency 1-3 days per year	35,369
										Time spent on SM	Time spent on SNS per day	Sports frequency 1-3 days per month	35,369
										Time spent on SM	Time spent on SNS per day	Sports frequency 1-4 days per week	35,369
										Time spent on SM	Time spent on SNS per day	Sports frequency 5-7 days per week	35,369
Cavazos-Rehg 2014[48]	Cross-sectional	2011	USA	High income country with mixed SEP	Grade 6-12 school students' part of the National Youth Tobacco Survey	NR [11-17]	High	1	Yes	Exposure to health-risk behaviour content	Exposure to tobacco ads/promotions via Facebook/ Myspace in the past month	Used any form of tobacco in the past month	15,673

Author and year	Study design	Study period	Country	Equity	Participants	Mean age [range]	Risk of bias	N°. of dp	MA?	Exposure	Exposure measure	Outcome measure	N
Chang 2016[49]	Cohort	2010-2011	Taiwan	High income country with mixed SEP	Grade 10 students from 26 high schools in Taipei City and New Taipei City	NR	High	2	Yes	Freq. of SM use	Freq. of online game use during past week	Incidence of unwanted online sexual solicitation perpetration in the past year	1,981
										Freq. of SM use	Freq. of chat room use during past week	Incidence of unwanted online sexual solicitation perpetration in the past year	1,981
Chapin 2018[50]	Cross-sectional	2016-2017	USA	High income country with mixed SEP	Middle and high school students' part of the Empowering Latino Youth Project evaluation	14.1 [12-18]	High	2	No	Other SM activities	Number of SM platforms used	Experience with electronic violence (perpetration) in the past month	1,167
										Other SM activities	Number of SM platforms used	Experience with face-to-face violence (perpetration) in the past month	1,167
Chau 2022[51]	Cross-sectional	2010	France	High income country with mixed SEP	Students attending 3 middle schools (2 public and 1 private) in the Lorraine region of North-eastern France	13.5 [10-18]	Mod	5	Yes	Time spent on SM	Time spent on discussion forums and chatting online during a weekday	Alcohol use in the past month	1,559
										Time spent on SM	Time spent on discussion forums and chatting online during a weekday	Tobacco use in the past month	1,559
										Time spent on SM	Time spent on discussion forums and chatting online during a weekday	Cannabis use in the past month	1,559
										Time spent on SM	Time spent on discussion forums and chatting online during a weekday	Perpetrated violence *(via validated tool)*	1,559
										Time spent on SM	Time spent on discussion forums and chatting online during a weekday	Illicit drug use in the past month	1,559
Chen 2019[52]	Cross-sectional	2018	Belgium	High income country with mixed SEP	School students' part of the New Media Study	16.4 [15-18]	High	2	Yes	Time spent on SM	Time spent on SM on a regular weekday and weekend day	Experience with risky (anti-social) selfie behaviour	686
										Exposure to health-risk behaviour content	Exposure to risky selfie descriptive norms	Experience with risky selfie (anti-social) behaviour	686
Coates 2019[53]	RCT	2017	UK	High income country	School students without food allergies	10.1 [9-11]	Some concerns (RoB-2)	1	Yes	Exposure to health-risk behaviour content	Exposure to unhealthy mock Instagram influencer marketing *(objectively recorded)*	Caloric intake (kcal): consumption of unhealthy snacks *(objectively recorded)*	117

Author and year	Study design	Study period	Country	Equity	Participants	Mean age [range]	Risk of bias	N°. of dp	MA?	Exposure	Exposure measure	Outcome measure	N
Coyne 2013[54]	Cross-sectional	2010	USA	High income country with mixed SEP	Families with an adolescent aged 11-14 who used SNS who were part of a larger study on family life	14.4 [11-14]	High	1	Yes	Time spent on SM	Time spent on SNS on a typical day	Delinquency *(via validated tool)*	491
Coyne 2018[55]	Cohort	2009-2014	USA	High income country with mixed SEP	Families with an adolescent aged 11-14 who used SNS who were part of the Flourishing Families Project	13.5 [10-14]	High	2	No	Time spent on SM	Time spent on SNS on a typical day	Physical aggression	457
										Time spent on SM	Time spent on SNS on a typical day	Relational aggression	457
Critchlow 2019[56]	Cross-sectional	2017	UK	High income country with mixed SEP	Sample members of the UK Youth Alcohol Policy Survey	15.2 [11-19]	Low	3	Yes	Freq. of SM use	SM apps used at least weekly in the past week	Higher-risk alcohol consumption in current drinkers *(via AUDIT-C)*	989
										Exposure to health-risk behaviour content	Participation with alcohol marketing on SM in the past month	Higher-risk alcohol consumption in current drinkers *(via AUDIT-C)*	1,387
										Exposure to health-risk behaviour content	Participation with user-created alcohol promotion on SM in the past month	Higher-risk alcohol consumption in current drinkers *(via AUDIT-C)*	1,591
da Costa 2021[57]	Cross-sectional	2019	Brazil	Low-middle income country with mixed SEP	High school students enrolled in high school courses integrated to professional courses, part of the Longitudinal Study of the Lifestyle of Adolescents	16.3 [14-18]	Low	3	Yes	Time spent on SM	Time spent on SM on a typical weekday and weekend day	Sedentary behaviour in the last 4 days *(via Actigraph accelerometer)*	718
										Time spent on SM	Time spent on SM on a typical weekday and weekend day	Light intensity physical activity in the last 4 days *(via Actigraph accelerometer)*	718
										Time spent on SM	Time spent on SM on a typical weekday and weekend day	Moderate to vigorous physical activity in the last 4 days *(via Actigraph accelerometer)*	718

**Author and year**	**Study design**	**Study period**	**Country**	**Equity**	**Participants**	**Mean age [range]**	**Risk of bias**	**N°. of dp**	**MA?**	**Exposure**	**Exposure measure**	**Outcome measure**	**N**
Dai 2022[58]	Cross-sectional	2019	China	High income country with mixed SEP	Junior, senior high and vocational high school students in Shanghai	13.7 [13-18]	Low	2	Yes	Exposure to health-risk behaviour content	Exposure to e-cigarette advertisements on SM *(via validated tool)*	Ever e-cigarette use *(via validated tool)*	708,765
										Exposure to health-risk behaviour content	Exposure to e-cigarette advertisements on SM *(via validated tool)*	E-cigarette use in the past month *(via validated tool)*	708,765
Davis 2019[59]	Cohort	2010-2016	USA	HIC Mixed SEP	Grade 6-7 middle school students' part of the CHOICE USA alcohol and drug use prevention program	13.2 [12-15]	Mod	1	No	Exposure to health-risk behaviour content	Substance related media exposure via SM in the past 3 months	Freq. of alcohol use in the past month	4840
Dawson 2019[60]	Cross-sectional	2016-2017	USA	High income country with mixed SEP	Students with previous diagnosis of ADHD part of the BEST Project	14.5 [NR]	High	3	Yes	Freq. of SM use	Presence of SNS use	Ever sent a sext	58
										Freq. of SM use	Number of participant posts on Facebook (posted by participant) over 2-month period *(objectively recorded)*	Ever sent a sext	34
										Exposure to health-risk behaviour content	% of participant posts sharing inappropriate content on Facebook over 2-month period *(objectively recorded)*	Ever sent a sext	34
de Bruijn 2016[61]	Cross-sectional	2012	Germany, Italy, Netherlands, and Poland	High income country	Urban and rural school students	14.1 [NR]	Mod	2	Yes	Exposure to health-risk behaviour content	Ever use of an alcohol branded SM page	Onset of drinking	9,032
										Exposure to health-risk behaviour content	Ever used alcohol branded SM page	Binge drinking in the past month	9,032
De Jans 2021[62]	RCT	2020	Belgium	High income country with mixed SEP	School students from 3 primary schools	10.0 [8-12]	Low	1	No	Exposure to health-risk behaviour content	Exposure to snack with low nutritional value (mini donut) on Instagram *(objectively recorded)*	Consumption of snack high in nutritional value (strawberries) *(objectively recorded)*	190
De Looze 2019[63]	Cross-sectional	2002-2014	European and North American Countries	High income country with mixed SEP	Sample members of the Health Behaviour in School-aged Children Survey	13.5 [13.1-13.8]	Mod	3	Yes	Freq. of SM use	Freq. of electronic media communication with friends	Weekly alcohol use	191,727
										Freq. of SM use	Freq. of electronic media communication with friends	Weekly smoking	191,727
										Freq. of SM use	Freq. of electronic media communication with friends	Lifetime cannabis use	56,159

Author and year	Study design	Study period	Country	Equity	Participants	Mean age [range]	Risk of bias	N°. of dp	MA?	Exposure	Exposure measure	Outcome measure	N
Doornwaard 2014[64]	Cross-sectional	2012	Netherlands	High income country	Elementary and high school students' part of the Studies on Trajectories of Adolescent Relationships and Sexuality	15.0 [11-18]	High	1	No	Exposure to health-risk behaviour content	Exposure to displays of sexual references on Facebook over 3-month period *(objectively recorded)*	Experience with sexual behaviours *(via validated tool)*	104
Doornwaard 2015[65]	Cross-sectional	2011	Netherlands	High income country	Grade 7-10 students' part of the Studies on Trajectories of Adolescent Relationships and Sexuality	14.0 [11-17]	Mod	4	Yes	Time spent on SM	Time spent on SNS (most frequently used platform) per day	Ever experience with sexual behaviours	1,132
										Time spent on SM	Time spent on SNS (most frequently used platform) per day	Freq. of sex related online behaviours	1,132
Elton-Marshall 2016[66]	Cross-sectional	2012-2013	Canada	High income country with mixed SEP	Grade 9-12 school students' part of the Canadian Youth Smoking Survey who responded to the Youth Gambling Survey supplement	16.5 [13-19]	High	2	Yes	Freq. of SM use	Freq. of playing free simulated gambling games on Facebook in the past 3 months	Freq. of gambling for money (not via SM)	9,830
										Freq. of SM use	Freq. of online gambling participation in the past 3 months	Problem gambling severity *(via CAGI/GPSS)*	3,682
Erreygers 2017[67]	Cross-sectional	2015	Belgium	High income country with mixed SEP	Grade 7 school students' part of a larger study	13.6 [NR]	High	1	No	Freq. of SM use	Freq. of online gaming in the past 6 months	Performing online anti-social behaviours in the past month	1,720
Floros 2013[68]	Cross-sectional	2010	Greece	High income country with mixed SEP	High school students' part of the Hippocrates Study	15.1 [12-19]	High	2	No	Freq. of SM use	Freq. of using SNS in the past year	Internet gambling (not via SM) freq. in the past year	2,017
										Freq. of SM use	Freq. of using SNS in the past year	Pathological gambling past year *(via DSM-IV-MR-J)*	2,017
Folkvord 2020[69]	RCT	2018	Netherlands	High income country	Grade 1-2 secondary school students	14.1 [13-16]	Low (RoB-2)	1	No	Exposure to health-risk behaviour content	Exposure to manipulated popular influencer Instagram post showing energy dense foods *(objectively recorded)*	Vegetable intake *(objectively recorded)*	88

Author and year	Study design	Study period	Country	Equity	Participants	Mean age [range]	Risk of bias	N°. of dp	MA?	Exposure	Exposure measure	Outcome measure	N
Froyland 2020[70]	Cross-sectional	2015 & 2018	Norway	High income country	All junior and senior high school students in Oslo part of the Young in Oslo Surveys	NR [13-18]	Low	8	Yes	Time spent on SM	Time spent on SM per day	Physical fighting (with and without weapons) in the past 12 months	47,655
										Time spent on SM	Time spent on SM per day	School truancy in the past 12 months	47,655
										Time spent on SM	Time spent on SM per day	Alcohol intoxication in the past 12 months	47,655
										Time spent on SM	Time spent on SM per day	Cannabis use in the past 12 months	47,655
Gascoyne 2021[71]	Cross-sectional	2018	Australia	High income country with mixed SEP	Secondary school students' part of the National Secondary Students' Diet and Activity Survey	NR [12-17]	Low	2	Yes	Exposure to health-risk behaviour content	Liked/shared posts related to a food or drink product or brand (e.g., soft drink, fast food)	High intake of unhealthy food	7,358
										Exposure to health-risk behaviour content	Liked/shared posts related to a food or drink product or brand (e.g., soft drink, fast food)	High intake of unhealthy drinks	7,358
Gazendam 2021[72]	Cross-sectional	2018	Canada	High income country with mixed SEP	Grade 9-10 students, part of the Canadian Health Behaviour in School-aged Children Survey	15.4 [NR]	Low	2	No	Time spent on SM	Time spent on SM per day	Early sexual intercourse (15 years or younger)	6,123
Geber 2021[73]	Cohort	2019-2020	Switzerland	High income country	1st year students at 4 secondary schools	15.1 [13-17]	Mod	1	Yes	Exposure to health-risk behaviour content	Exposure to alcohol related content on Instagram and Snapchat	Drinking behaviour	402
Geusens 2017[74]	Cross-sectional	2015	Belgium	High income country with mixed SEP	5th, 6th and 7th year secondary school students' part of the Flemish Alcohol and Media Survey Research Project	17.2 [16-20]	Mod	2	Yes	Exposure to health-risk behaviour content	Perceived number of friends sharing alcohol references online	Self-reported drinking behaviour *(via AUDIT)*	2,935
Geusens 2017[75]	Cohort	2015-2016	Belgium	High income country	5th, 6th and 7th year secondary school students' part of the Flemish Alcohol and Media Survey Research Project	17.0 [16-20]	High	2	No	Exposure to health-risk behaviour content	Freq. of sharing alcohol references on SNS	Binge drinking in the last 12 months	998
Geusens 2019[76]	Cross-sectional	2014	Belgium	High income country with mixed SEP	3rd and 4th year secondary school students' part of a larger study	14.9 [14-16]	Low	2	Yes	Exposure to health-risk behaviour content	Freq. of exposure to peer alcohol references on SNS	Alcohol consumption	886
										Exposure to health-risk behaviour content	Freq. of sharing of alcohol references on SNS	Alcohol consumption	886

Author and year	Study design	Study period	Country	Equity	Participants	Mean age [range]	Risk of bias	N°. of dp	MA?	Exposure	Exposure measure	Outcome measure	N
Gomez 2019[77]	Cross-sectional	2018	Spain	High income country	Secondary and baccalaureate students	14.4 [12-17]	High	1	No	Other SM activities	Signed up to more than 5 SNS	Online gambling & betting (not via SM)	3,772
Gordon 2011[78]	Cross-sectional	2006-2007	UK	High income country with mixed SEP	2nd year high school students' part of the Assessing the Cumulative Impact of Alcohol Marketing on Youth Drinking Study	13.0 [12-14]	High	2	Yes	Exposure to health-risk behaviour content	Awareness of alcohol marketing on SNS	Drinking status	912
										Exposure to health-risk behaviour content	Used SNS containing alcohol brands or logos	Drinking status	912
Gregg 2018[79]	Cross-sectional	2015	USA	High income country	High school students from 1 suburban high school	16.2 [NR]	Mod	1	No	Freq. of SM use	Freq. of electronic communication	Freq. of sending sexts *(via SBS)*	314
Gunnlaugsson 2020[80]	Cross-sectional	2017	Guinea-Bissau	Low-middle income country with mixed SEP	Students from 16 secondary schools in Bissau	NR [14-19+]	Low	3	Yes	Freq. of SM use	Freq. of SM use *(via validated tool)*	Participated in bullying behaviour in the past 12 months *(via validated tool)*	1,454
										Freq. of SM use	Freq. of SM use *(via validated tool)*	Lifetime experience of smoking cigarettes *(via validated tool)*	1,566
										Freq. of SM use	Freq. of SM use *(via validated tool)*	Lifetime experience of drinking alcohol *(via validated tool)*	1,559
Hamilton 2020[81]	Cross-sectional	2020	USA	High income country with high SEP	Adolescent girls residing in Pennsylvania part of larger longitudinal study	15.06 [12-17]	High	1	No	Time spent on SM	Time spent on SNS per day	Physical activity	93
Hayer 2018[82]	Cohort	2015-2016	Germany	High income country	Grade 6-10 school students in Northern Germany	13.4 [11-19]	Mod	2	Yes	Freq. of SM use	Freq. of participation in any simulated gambling on social networks in the past year	Freq. of monetary gambling (not via SM) in the past year	531
										Freq. of SM use	Freq. of participation in simulated gambling from home on social networks in the past year	Freq. of participation in monetary gambling (not via SM) in the past year	531
Holtz 2011[83]	Cross-sectional	2007	Austria	High income country	Rural and urban school students	12.7 [10-14]	Mod	1	No	Freq. of SM use	Freq. of communicational internet use (e.g., chatrooms, social platforms like Myspace)	Delinquent and aggressive behaviours in the past 6 months *(via YSR)*	205

Author and year	Study design	Study period	Country	Equity	Participants	Mean age [range]	Risk of bias	N°. of dp	MA?	Exposure	Exposure measure	Outcome measure	N
Hryhorczuk 2019[84]	Cross-sectional	2011	Ukraine	Low-middle income country	Sample members of the Family and Children of Ukraine Birth Cohort Study	16.2 [15.1-18.2]	Mod	6	Yes	Freq. of SM use	Freq. of SM use	Used alcohol in the past month	912
										Freq. of SM use	Freq. of SM use	Used alcohol in the past year	917
										Freq. of SM use	Freq. of SM use	Ever used alcohol	967
Hrywna 2020[85]	Cross-sectional	2018	USA	High income country	Grade 9-12 school students' part of the New Jersey Youth Tobacco Survey	NR	Mod	2	Yes	Exposure to health-risk behaviour content	Liked/followed a tobacco brand on SM in the past year	Current use of e-cigarette or Juul (use on ≥1 day of the past month)	4,183
										Exposure to health-risk behaviour content	Liked/followed a tobacco brand on SM in the past year	Frequent use of e-cigarette or Juul (use on ≥20 days of the past month)	4,183
Huang 2012[86]	Cross-sectional	2007	China	High income country with mixed SEP	Grade 10 academic and vocational school students' part of the Trans-disciplinary Tobacco and Alcohol Use Research Centre Study	15.8 [13-19]	Low	1	No	Freq. of SM use	Freq. of social internet activity (online gaming, chatting with real friends, chatting with online friends) in the past week	Cigarette smoking in the past month	2,931
Huang 2014[87]	Cohort	2010-2011	USA	High income country with low SEP	Grade 10 school students' part of the Social Network Study	15.1 [NR]	Low	4	Yes	Freq. of SM use	Freq. of Myspace use	Alcohol use in the past month	1,315
										Freq. of SM use	Freq. of Facebook use	Ever smoking	1,315
										Exposure to health-risk behaviour content	Number of friends who posted risky pictures partying/ drinking	Alcohol use in the past month	1,315
										Exposure to health-risk behaviour content	Number of friends who posted risky pictures partying/ drinking	Ever smoking	1,315

Author and year	Study design	Study period	Country	Equity	Participants	Mean age [range]	Risk of bias	N°. of dp	MA?	Exposure	Exposure measure	Outcome measure	N
Jeong 2022[88]	Cross-sectional	2020	South Korea	High income country	Adolescents part of the Consumer Behaviour Survey for Food conducted by the Korea Rural Economic Institute	16.1 [NR]	High	2	No	Freq. of SM use	Freq. of SM use	Eats food considering calories and nutrients *(via validated tool)*	622
										Freq. of SM use	Freq. of SM use	Eats carefully selected food for one's own health *(via validated tool)*	622
Jiang 2018[89]	Cross-sectional	NR	China	High income country	Young inpatients enrolled at one of the largest addiction clinics in China	16.8 [13-19]	High	1	No	Freq. of SM use	Freq. of online gaming *(via clinical records)*	Involvement in risk behaviours (e.g., skipping school, smoking) *(via clinical records)*	467
Kaufman 2014[90]	Cross-sectional	2012	South Africa	Low-middle income country with mixed SEP	Grade 9 school students, part of a 2-year cluster-randomised trial	NR [12-20]	Low	8	Yes	Freq. of SM use	Freq. of SM use	Hazardous alcohol use in the past year *(via AUDIT)*	4,485
										Freq. of SM use	Freq. of SM use	Reported multiple partners in the past year	4,485
										Other SM activities	Has a Facebook account	Hazardous alcohol use in past year *(via AUDIT)*	4,485
										Other SM activities	Has a Facebook account	Reported multiple partners in the past year	4,485
Kaur 2020[91]	Cross-sectional	2018	USA	High income country with mixed SEP	Grade 8,10 and 12 school students' part of the Monitoring the Future Survey	15.1 [NR]	Low	6	No	Time spent on SM	Time spent on SNS per day	Binge drinking in the past 2 weeks	22,980
										Time spent on SM	Time spent on SNS per day	Drinking in the past month	23,150
										Time spent on SM	Time spent on SNS per day	Cannabis use in the past month	23,167
										Time spent on SM	Time spent on SNS per day	Flavour vaping in the past month in 8th and 10th grade students	6,967
										Time spent on SM	Time spent on SNS per day	Cannabis vaping in the past month in 8th and 10th grade students	7,003
										Time spent on SM	Time spent on SNS per day	Nicotine vaping in the past month in 8th and 10th grade students	6,980

Author and year	Study design	Study period	Country	Equity	Participants	Mean age [range]	Risk of bias	N°. of dp	MA?	Exposure	Exposure measure	Outcome measure	N
Kelleghan 2020[92]	Cohort	2015-2017	USA	High income country with mixed SEP	High School students' part of the Happiness & Health Study	16.5 [NR]	Mod	6	Yes	Freq. of SM use	Freq. of SM posting (posting photos, video or statuses and sharing others content)	Any cannabis use initiation (incl. reported use of combustible cannabis, blunts, and edible, vaporized, or synthetic cannabis)	1,841
										Freq. of SM use	Freq. of SM posting (posting photos, video or statuses and sharing others content)	Combustible cannabis use initiation	1,841
										Freq. of SM use	Freq. of SM posting (posting photos, video or statuses and sharing others content)	Other cannabis use initiation (incl. reported use of edible, vaporized, or synthetic cannabis)	1,841
										Freq. of SM use	Freq. of SM posting (posting photos, video or statuses and sharing others content)	Any tobacco use initiation (incl. reported use of a few puffs of a cigarette, a whole cigarette, e-cigarettes with tobacco, smokeless tobacco, big cigars, little cigars/ cigarillos, and hookah water pipe)	1,558
										Freq. of SM use	Freq. of SM posting (posting photos, video or statuses and sharing others content)	Combustible cigarette use initiation (incl. reported use of a few puffs of a cigarette or a whole cigarette)	1,558
										Freq. of SM use	Freq. of SM posting (posting photos, video or statuses and sharing others content)	E-cigarette use initiation	1,558
King 2014[93]	Cross-sectional	2012	Australia	High income country	Secondary school students in Metropolitan region of Adelaide	14.9 [12-17]	High	1	Yes	Freq. of SM use	Ever use of simulated gambling via SNS applications (Facebook apps)	Freq. of problem gambling *(via DSM-IV-MR-J)*	1,214
Ko 2009[94]	Cross-sectional	2004	Taiwan	High income country with mixed SEP	Junior high and senior high/vocational school students' part of the Project for Health of Adolescents	14.6-14.9 [NR]	Mod	3	Yes	Freq. of SM use	Ever online gaming	Aggressive behaviours in the past year	9,405
										Freq. of SM use	Ever online chatting	Aggressive behaviours in the past year	9,405
										Freq. of SM use	Ever online gambling	Aggressive behaviours in the past year	9,405

Author and year	Study design	Study period	Country	Equity	Participants	Mean age [range]	Risk of bias	N°. of dp	MA?	Exposure	Exposure measure	Outcome measure	N
Kontostoli 2022[95]	Cross-sectional	2015-2016	UK	High income country with mixed SEP	Adolescents part of the Millennium Cohort Study	14.2 [NR]	Low	6	No	Time Spent on SM	Time spent browsing and updating SNS on a weekday	Moderate-to-vigorous physical activity *(via accelerometer)*	4,546
										Time Spent on SM	Time spent browsing and updating SNS on a weekday	Overall physical activity *(via accelerometer)*	4,546
										Time Spent on SM	Time spent browsing and updating SNS on a weekday	Sedentary behaviour	3,551
Koutamanis 2015[96]	Cross-sectional	2012	Netherlands	High income country	Families with ≥2 adolescents aged 10-15	12.6 [12-15]	High	1	No	Freq. of SM use	Freq. of online social exploration on SNS	Freq. of risky online self-presentation	758
Kwon 2022[97]	Cross-sectional	2017	South Korea	High income country with mixed SEP	Middle and high school students' part of the Korea Youth Health Risk Behaviour Web Based Online Survey	15.0 [12-18]	Low	4	No	Freq. of SM use	Freq. of SNS use in the past 30 days	Moderate to vigorous aerobic physical activity at least 5 days per week or vigorous physical activity at least 3 days per week in the past 7 days	53,133
										Freq. of SM use	Freq. of SNS use in the past 30 days	Muscle-strengthening activity for at least 3 days per week in past 7 days	53,133
Landry 2013[98]	Cross-sectional	2011-2012	USA	High income country	Grade 9-10 Latino high school students in Maryland	15.7 [13-19]	High	2	Yes	Freq. of SM use	Freq. of logging into SM sites	No contraception use at last sex	118
										Other SM activities	Has a Facebook account	No contraception use at last sex	118
Larm 2017[99]	Cross-sectional	2010	Sweden	High income country	Grade 9 primary school students' part of the Survey of Adolescent Life in Vastmanland	NR [15-16]	High	2	Yes	Time spent on SM	Online social network chatting	Alcohol use *(via AUDIT-C)*	2,439
Larm 2019[100]	Repeat cross-sectional	2008 2010 2012	Sweden	High income country	Grade 9 students' part of the Survey of Adolescent Life in Vastmanland	NR [15-16]	High	2	Yes	Time spent on SM	Time spent on SM/ chatting per day	Drinking in the past year *(via AUDIT-C)*	2605 (RCS: 2008)<br><br>2045 (RCS: 2012)
Lee 2015[101]	Cross-sectional	2012	South Korea	High income country	Grade 1-3 middle school and Grade 5-6 elementary school students residing in Incheon	NR [11-16]	High	1	No	Time spent on SNS per day	Time spent on SNS per day	Negative SNS behaviours (e.g., real money trading in SNS games, exposed to porn on SNS)	500

Author and year	Study design	Study period	Country	Equity	Participants	Mean age [range]	Risk of bias	N°. of dp	MA?	Exposure	Exposure measure	Outcome measure	N
Lee 2019[102]	Cohort	2013-2016	USA	High income country with mixed SEP	Non-institutionalised adolescents' part of the Population Assessment of Tobacco and Health Study	NR [12-17]	High	2	No	Freq. of SM use	Freq. of visiting social networking account	Initiation of ENDS in the past year	8,704
Lee 2021[103]	Cross-sectional	2017-2018	China	High income country	Students from 1 secondary school	18.4 [NR]	High	2	Yes	Time Spent on SM	Daytime use of social networks *(objectively recorded)*	Number of steps in the past 7 days *(via accelerometer)*	32
										Time Spent on SM	Daytime use of social networks *(objectively recorded)*	Moderate to vigorous physical activity in the past week *(via accelerometer)*	32
Lee 2021[104]	Cross-sectional	2019	USA	High income country	Middle and high school students' part of the Florida Youth Tobacco Survey	NR [NR]	Mod	2	No	Freq. of SM use	Freq. of Facebook use	Experimental vaping (vaped but not in the past month)	10,475
										Freq. of SM use	Freq. of Facebook use	Current vaping in the past month	10,475
Lin 2012[105]	Cross-sectional	NR	New Zealand	High income country with mixed SEP	Student's part of a larger study	NR [13-14]	High	2	Yes	Exposure to health-risk behaviour content	Awareness of alcohol marketing on SNS	Drinking status in the past year	2,538
										Exposure to health-risk behaviour content	Used SNS containing alcohol brands or logos	Drinking status in the past year	2,538
Lipsky 2017[106]	Cohort	2010-2014	USA	HIC Mixed SEP	Grade 10 school students' part of the NEXT Generation Health Study	16.5 [NR]	Low	3	No	Time spent on SM	Time spent on social networking per day	Healthy eating (conformance to US Dietary Guidelines 2010) *(via HEI, ASA24)*	566
										Time spent on SM	Time spent on social networking per day	Intake of empty calories *(via HEI, ASA24)*	566
										Time spent on SM	Time spent on social networking per day	Intake of whole plant foods *(via ASA24)*	566
Longobardi 2021[107]	Cross-sectional	NR	Italy	High income country	Grade 7-13 school students' part of a larger study on SM use	15 [NR]	High	1	Yes	Time spent on SM	Time spent on SM per day	Sexting and online exhibitionism *(via validated tool)*	229
McClure 2020[108]	Cross-sectional	2015-2016	USA	HIC Mixed SEP	Adolescents recruited from general paediatric clinics in New England	14.5 [12-17]	High	1	No	Freq. of SM use	Freq. of SM use	Ever drinking	202

Author and year	Study design	Study period	Country	Equity	Participants	Mean age [range]	Risk of bias	N°. of dp	MA?	Exposure	Exposure measure	Outcome measure	N
Merrill 2019[109]	Cross-sectional	2015	USA	High income country with mixed SEP	Grade 9-12 school students' part of the Youth Risk Behaviour Surveillance System Survey	NR [12-18+ older]	High	1	Yes	Time spent on SM	Time spent on SM per day	Risky sexual behaviours in sexually active participants	5,603
Michael 2016[110]	Cross-sectional	2015	Nigeria	Low-middle income country	Adolescents residing in Bayelsa State Capital	15.1 [10-19]	High	1	No	Time spent on SM	Average time spent on SM per day	No use of contraception during sexual intercourse	262
Moitra 2022[111]	Cross-sectional	2021	India	Low-middle income country with mixed SEP	Grade 6-10 students from 6 private schools and 4 government aided schools in Mumbai	13.2 [10-15]	Low	2	Yes	Time spent on SM	Time spent using SNS on a typical weekend and weekday	Healthy eating habits	1,298
										Time spent on SM	Time spent using SNS on a typical weekend and weekday	Physical activity level *(via PAQ-C/-A)*	1,298
Mojica 2014[112]	Cross-sectional	2010	USA	High income country	Female adolescents recruited via Girls Scouts of Southwest Texas as part of an intervention planning grant	NR [11-14]	High	3	No	Time spent on SM	Average time spent on SNS per week	5+ days of physically activity in the past week	110
										Time spent on SM	Average time spent on SNS per week	Daily physical education class in an average week	110
										Time spent on SM	Average time spent on SNS per week	Played on 1+ sports team in the past year	110
Molla-Esparza 2021[113]	Cross-sectional	2015	Spain	High income country	Adolescents from 2 secondary charter schools and 2 state schools in the south of Valencia	13.7 [12-18]	Mod	3	Yes	Freq. of SM use	Freq. of using SM platforms	Sent a sext	647
										Other SM activities	Number of SM platforms used	Sent a sext	647
										Other SM activities	Number of SM platforms used	Forwarded a sext	647
Nesi 2017[114]	Cohort	2009-2013	USA	High income country with mixed SEP	Grade 6-8 middle school students' part of a larger study	15.8 [NR]	Mod	6	Yes	Exposure to health-risk behaviour content	Ever exposed to friends' SNS alcohol content	Initiation of drinking	658
										Exposure to health-risk behaviour content	Ever exposed to friends' SNS alcohol content	Initiation of becoming drunk	658
										Exposure to health-risk behaviour content	Ever exposed to friends' SNS alcohol content	Initiation of heavy episodic drinking	658
										Time spent on SM	Average time on Facebook per day	Initiation of drinking	658
										Time spent on SM	Average time on Facebook per day	Initiation of becoming drunk	658
										Time spent on SM	Average time on Facebook per day	Initiation of heavy episodic drinking	658

Author and year	Study design	Study period	Country	Equity	Participants	Mean age [range]	Risk of bias	N°. of dp	MA?	Exposure	Exposure measure	Outcome measure	N
Nesi 2019[115]	Cohort	2015-2016	USA	High income country with low SEP	Grade 7-8 rural, lower-middle class school students' part of a larger study	16.0 [NR]	High	10	Yes	Freq. of SM use	Freq. of daily SM use	No. of sexual partners in the past year	716
										Freq. of SM use	Posted selfies on Instagram over 1 month period *(objectively recorded)*	No. of sexual partners in the past year	233
										Freq. of SM use	Posted selfies on Instagram over 1 month period *(objectively recorded)*	Substance use (alcohol use and past year marijuana and cigarette use)	233
										Other SM activities	Online status-seeking strategy use	No. of sexual partners in the past year	716
										Other SM activities	Online status-seeking strategy use	Substance use (alcohol and past year marijuana and cigarette use)	716
Ng Fat 2021[116]	Cohort	2011-2016	UK	High income country with mixed SEP	Sample members of the UK Household Longitudinal Survey	12.5 (10-15 year olds) 17.5 (16-19 year olds) [10-19]	Low	8	Yes	Time spent on SM	Time spent on SM on a normal weekday	Increase in drink freq. in the past 3 years (in those 10-15 years)	856
										Time spent on SM	Time spent on SM on a normal weekday	Increase in drink freq. in the past 3 years (in those 16-19 years)	511
										Time spent on SM	Time spent on SM on a normal weekday	Increase in binge drink freq. in the past 3 years (in those 16-19 years)	1,057
										Time spent on SM	Time spent on SM on a normal weekday	Drank alcohol in the past month (in those 10-15 years)	4,093
										Time spent on SM	Time spent on SM on a normal weekday	Drank alcohol 1-3 times a month in the past month (in those 16-19 years)	2,689
										Time spent on SM	Time spent on SM on a normal weekday	Drank alcohol at least weekly in the past month (in those 16-19 years)	2,689
										Time spent on SM	Time spent on SM on a normal weekday	Binge drinking 1-2 times a month (in those 16-19 years)	2,687
										Time spent on SM	Time spent on SM on a normal weekday	Binge drinking ≥3 times a month (in those 16-19 years)	2,687

Author and year	Study design	Study period	Country	Equity	Participants	Mean age [range]	Risk of bias	N°. of dp	MA?	Exposure	Exposure measure	Outcome measure	N
Ngqangashe 2021[117]	RCT	NR	Belgium	High income country with mixed SEP	Students in Flanders part of a larger research project on food media use	13.9 [12-14]	Some concerns	1	No	Exposure to health-risk behaviour content	Watched YouTube Tasty video portraying preparation of sweet snacks *(objectively recorded)*	Food choice behaviour (choosing fruit over a sweet snack) *(objectively recorded)*	126
Ohannessian 2009[118]	Cross-sectional	2006	USA	High income country	Grade 9-10 high school students	15.0 [14-16]	High	2	No	Time spent on SM	Time spent emailing and instant messaging on an average/typical day	Freq. of daily alcohol consumption in the past 6 months	328
										Time spent on SM	Time spent emailing and instant messaging on an average/typical day	Smoking onset	328
Pegg 2018[119]	Cross-sectional	2014	Australia	High income country	Year 12 students' part of the Youth Activity Participation Survey	17.3 [NR]	High	2	Yes	Exposure to health-risk behaviour content	SNS alcohol exposure in the past 6 months	Alcohol use in the past 6 months	793
										Freq. of SM use	Freq. of SNS use (intensity)	Alcohol use in the past 6 months	793
Perez 2022[120]	Cohort	2014-2016	USA	High income country with mixed SEP	Adolescents part of the Population Assessment of Tobacco and Health Study	NR [12-17]	Low	1	No	Exposure to health-risk behaviour content	Exposure to tobacco related content on SM in the past 12 months (including e-cigarettes)	Dual use of e-cigarettes and at least one combustible product in the past month	16,109,064
Prince 2021[121]	Cross-sectional	2015-2017	USA	High income country with mixed SEP	Grade 7-12 students living on or near reservations part of the Our Youth, Our Future Survey	14.78 [NR]	High	1	Yes	Freq. of SM use	Freq. of Snapchat use	Past month opioid use	25

Author and year	Study design	Study period	Country	Equity	Participants	Mean age [range]	Risk of bias	N°. of dp	MA?	Exposure	Exposure measure	Outcome measure	N
Qutteina 2022[122]	Cross-sectional	2019	Belgium	High income country with mixed SEP	Adolescents attending 18 secondary schools across Flanders	15 [11-19]	Mod	5	No	Exposure to health-risk behaviour content	Exposure to non-core foods (energy dense, low nutrient: sweetened drinks, sweets, salty/savoury snacks) on SM	Freq. of sweet intake in the past month	1,002
										Exposure to health-risk behaviour content	Exposure to non-core foods (energy dense, low nutrient: sweetened drinks, sweets, salty/savoury snacks) on SM	Consumption of sweets in the past month (g/day)	1,002
										Exposure to health-risk behaviour content	Exposure to non-core foods (energy dense, low nutrient: sweetened drinks, sweets, salty/savoury snacks) on SM	Freq. of soft drink intake in the past month	1,002
										Exposure to health-risk behaviour content	Exposure to non-core foods (energy dense, low nutrient: sweetened drinks, sweets, salty/savoury snacks) on SM	Consumption of soft drinks in the past month (ml/day)	1,002
										Exposure to health-risk behaviour content	Exposure to non-core foods (energy dense, low nutrient: sweetened drinks, sweets, salty/savoury snacks) on SM	Freq. of fried food intake in the past month	1,002
Riehm 2021[123]	Cross-sectional	2015	USA	High income country with mixed SEP	Students from 10 public high schools in Los Angeles part of the Happiness & Health Study	16.5 [NR]	High	1	Yes	Freq. of SM use	Freq. of posting own photos, images, videos, status updates, or blogs on SM	Ever use of alcohol	2,373
Roditis 2016[124]	Cross-sectional	2014-2015	USA	High income country	Grade 9 and 12 high school students residing in California	16.1 [NR]	Mod	2	No	Other SM activities	Ever seen a message posted on SM about the risks/bad things of using marijuana	Ever use of marijuana	786
										Exposure to health-risk behaviour content	Ever seen a message posted on SM about the benefits/ good things of using marijuana	Ever use of marijuana	786

Author and year	Study design	Study period	Country	Equity	Participants	Mean age [range]	Risk of bias	N°. of dp	MA?	Exposure	Exposure measure	Outcome measure	N
Romo 2017[125]	Cross-sectional	2014	USA	High income country with mixed SEP	Adolescents recruited from 3 primary care paediatric clinics in Northern Manhattan	16.0 [13-21]	High	8	Yes	Freq. of SM use	Freq. of SNS use *(via validated tool)*	Inconsistent condom use overall and at last sex	333
										Freq. of SM use	Freq. of SM app use *(via validated tool)*	Inconsistent condom use overall and at last sex	333
										Freq. of SM use	Freq. of SNS use *(via validated tool)*	4 or more lifetime partners in sexually active participants	333
										Freq. of SM use	Freq. of SNS use *(via validated tool)*	More than 1 partner in the past 3 months in sexually active participants	333
										Freq. of SM use	Freq. of SNS use *(via validated tool)*	STI diagnosis ever in sexually active participants	333
										Freq. of SM use	Freq. of SNS use *(via validated tool)*	History of no use of long-term acting reversible contraception in sexually active female participants	NR
										Freq. of SM use	Freq. of SNS use *(via validated tool)*	History of no use of regular forms of hormonal contraception in sexually female active participants	NR
										Freq. of SM use	Freq. of SNS use *(via validated tool)*	History of no use of emergency contraception in sexually active female participants	NR
Rutter 2021[126]	Cross-sectional	2019	USA	High income country with mixed SEP	Adolescents in the USA	14.6 [12-17]	Mod	1	No	Freq. of SM use	Freq. of SM use (checking and posting)	Physical activity	4,592
Sampasa-Kanyinga 2015[127]	Cross-sectional	2013	Canada	High income country with mixed SEP	Grade 11-12 high school students' part of the Ontario Student Drug Use and Health Survey	15.2 [NR]	High	2	Yes	Time spent on SM	Time spent on SM websites either posting or browsing per day	Cannabis use in the past year	5,329
										Time spent on SM	Time spent on SM websites either posting or browsing per day	Tobacco use in the past year	5,329

Author and year	Study design	Study period	Country	Equity	Participants	Mean age [range]	Risk of bias	N°. of dp	MA?	Exposure	Exposure measure	Outcome measure	N
Sampasa-Kanyinga 2015[128]	Cross-sectional	2013	Canada	High income country with mixed SEP	Grade 7-12 high school students' part of the Ontario Student Drug Use and Health Survey	15.2 [11-19]	Low	3	No	Time spent on SM	Time spent on SM websites either posting or browsing per day	Skipping breakfast in the past 5 days	9,858
										Time spent on SM	Time spent on SM websites either posting or browsing per day	Sugar-sweetened beverage consumption in the past 7 days	9,858
										Time spent on SM	Time spent on SM websites either posting or browsing per day	Energy drink consumption in the past 7 days	9,858
Sampasa Kanyinga 2016[129]	Cross-sectional	2013	Canada	High income country with mixed SEP	Grade 7-12 high school students' part of the Ontario Student Drug Use and Health Survey	15.2 [11-20]	Low	6	Yes	Time spent on SM	Time spent on SM websites either posting or browsing per day	Occasional alcohol use in the past year	4,814
										Time spent on SM	Time spent on SM websites either posting or browsing per day	Regular alcohol use in the past year	4,814
										Time spent on SM	Time spent on SM websites either posting or browsing per day	Binge drinking in the past month	4,814
Sampasa-Kanyinga 2016[130]	Cross-sectional	2013	Canada	High income country with mixed SEP	Grade 7-12 public high school students' part of the Ontario Student Drug Use and Health Survey	15.3 [11-19]	Low	2	No	Time spent on SM	Time spent on SM websites either posting or browsing per day	Not meeting physical activity recommendation in past week (≥60 minutes per day of moderate to vigorous physical activity on all 7 days)	9,388
Sampasa-Kanyinga 2018[131]	Cross-sectional	2013	Canada	High income country with mixed SEP	Grade 9-10 high school students' part of the Ontario Student Drug Use and Health Survey	16.1 [NR]	Mod	1	No	Time spent on SM	Time spent on SM websites either posting or browsing per day	Ever use of e-cigarettes	2,841
Sandercock 2016[132]	Cross-sectional	2014	UK	HIC Mixed SEP	Grade 6-11 junior and high school students residing in the East of England	13.5 [NR]	Low	5	No	Time spent on SM	Time spent on SM on a normal day	Daily sedentary time	678
										Time spent on SM	Time spent on SM on a normal day	High sedentary time	678
										Time spent on SM	Time spent on SM on a normal day	Low cardio-respiratory fitness in female participants	308

Author and year	Study design	Study period	Country	Equity	Participants	Mean age [range]	Risk of bias	N°. of dp	MA?	Exposure	Exposure measure	Outcome measure	N
Savolainen 2020[133]	Cross-sectional	2017-2019	USA, South Korea, Finland, and Spain	High income country	Adolescents in 4 countries across 4 continents	NR [15-17]	High	20	Yes	Freq. of SM use	Freq. of Facebook use *(via validated tool)*	Hazardous alcohol use *(via AUDIT-C)*	329 (USA)<br><br>264 (KOR)<br><br>154 (FIN)<br><br>314 (ESP)
										Freq. of SM use	Freq. of YouTube use *(via validated tool)*	Hazardous alcohol use *(via AUDIT-C)*	As above
										Freq. of SM use	Freq. of Twitter use *(via validated tool)*	Hazardous alcohol use *(via AUDIT-C)*	As above
										Freq. of SM use	Freq. of Instagram use *(via validated tool)*	Hazardous alcohol use *(via AUDIT-C)*	As above
										Freq. of SM use	Freq. of instant messaging *(via validated tool)*	Hazardous alcohol use *(via AUDIT-C)*	As above
Self-Brown 2018[134]	Cross-sectional	2014	Uganda	Low-middle income country with low SEP	Adolescents living in slums part of the Kampala Youth Survey	17.0 [12-18]	High	2	Yes	Freq. of SM use	Presence of SM use *(via validated tool)*	Transactional sex in sexually active youth *(via validated tool)*	593
Shan 2022[135]	Cohort	2013-2018	USA	High income country with mixed SEP	Adolescents part of the Population Assessment of Health and Tobacco Study	NR [12-14]	Low	2	Yes	Exposure to health-risk behaviour content	Followed tobacco brands (e.g., Marlboro, Newport, American Spirit, Vuse) on Facebook/Twitter or other SM sites	Initiation of cigarettes	6,557
										Exposure to health-risk behaviour content	Followed tobacco brands (e.g., Marlboro, Newport, American Spirit, Vuse) on Facebook/Twitter or other SM sites	Initiation of e-cigarettes	6,632

Author and year	Study design	Study period	Country	Equity	Participants	Mean age [range]	Risk of bias	N°. of dp	MA?	Exposure	Exposure measure	Outcome measure	N
Sharma 2021[136]	Cross-sectional	NR	India	Low-middle income country with mixed SEP	Grade 9-12 students in Barwala village, Delhi	NR	High	1	Yes	Exposure to health-risk behaviour content	Exposure to tobacco adverts on SM	Smokeless tobacco use	652
Shimoga 2019[137]	Cross-sectional	2014-2015	USA	High income country with mixed SEP	Grade 8,10 and 12 school students' part of the Monitoring the Future Survey	NR	Low	1	No	Freq. of SM use	Freq. of SM use	Freq. of vigorous physical activity	19,543
Smout 2021[138]	Cohort	2012-2015	Australia	High income country	Middle school students' part of the CAP study	13.4 [13-16]	Mod	2	Yes	Time Spent on SM	Time spent on SM on a typical day	Days drinking per month	441
										Exposure to health-risk behaviour content	Exposure to peer-generated content on SM depicting risky substance use	Days drinking per month	441
Soneji 2018[139]	Cohort	2013-2015	USA	High income country with mixed SEP	Sample members of the Population Assessment of Tobacco and Health Study	NR [12-17]	Low	4	Yes	Freq. of SM use	Freq. of social networking account use	Initiation of binge drinking in the past month	8,542
										Freq. of SM use	Freq. of social networking account use	Initiation of tobacco use in the past month	9,067
										Freq. of SM use	Freq. of social networking account use	Increased frequency of tobacco product use	11,996
										Freq. of SM use	Freq. of social networking account use	Progression from tobacco single-product to poly-product use (i.e., ≥ 2 products)	10,928
Stevens 2017[140]	Cross-sectional	2013-2014	USA	High income country with low SEP	Sample members of a larger mixed methods study	18.2 [13-24]	Low	2	No	Other SM activities	Exposure to contraception information on SNS in the past month	No condom use at last intercourse in sexually active participants	172
										Other SM activities	Exposure to contraception information on SNS in the past month	No contraception use at last intercourse in sexually active participants	175
Suwanwong 2021[141]	Cross-sectional	2017	Thailand	Low-middle income country	Adolescents part of the Cigarette Smoking and Alcohol Drinking Behaviour Survey	NR [15-19]	High	2	No	Other SM activities	Exposure to anti-smoking SM campaign	Smoking status (occasional smoker)	5,669
										Other SM activities	Exposure to anti-smoking SM campaign	Smoking status (daily smoker)	5,851

Author and year	Study design	Study period	Country	Equity	Participants	Mean age [range]	Risk of bias	N°. of dp	MA?	Exposure	Exposure measure	Outcome measure	N
Svensson 2020[142]	Repeat cross-sectional	2016-2019	Sweden	High income country	Students from 17 secondary schools in 8 small municipalities in the county of Skåne part of the Öckerö project	NR [14-15]	Mod	3	No	Freq. of SM use	Freq. of posting information on Facebook, Instagram, Snapchat, or other SM	Lifetime alcohol use	3,733
										Freq. of SM use	Freq. of posting information on Facebook, Instagram, Snapchat, or other SM	Drunkenness in the past year	3,733
										Freq. of SM use	Freq. of posting information on Facebook, Instagram, Snapchat, or other SM	Drunkenness in the past month	3,733
Tao 2022[143]	Cross-sectional	2020-2021	USA	High income country with mixed SEP	Adolescents	16.5 [15-18]	High	2	Yes	Time Spent on SM	Average time on SM per week	Alcohol use disorder *(via AUDIT*)	407
										Time Spent on SM	Average time on SM per week	Illicit drug use problems *(via validated tool)*	407
Trangenstein 2019[144]	Cross-sectional	2018	USA	High income country with mixed SEP	Adolescents residing in USA states with legalised retail cannabis	NR [15-19]	High	2	No	Exposure to health-risk behaviour content	Liked/ followed cannabis business pages on Facebook, Twitter and/or Instagram	Cannabis use in the past year	482
										Exposure to health-risk behaviour content	Liked/ followed cannabis business pages on Facebook, Twitter and/or Instagram	Cannabis use in the past 28 days	482
Tsitsika 2009[145]	Cross-sectional	2007-2008	Greece	High income country with mixed SEP	Grade 9-10 urban district school students in Athens	14.9 [NR]	High	1	Yes	Freq. of SM use	Ever accessed the internet to visit chat rooms	Freq. of pornographic internet site use	344
Tsitsika 2011[146]	Cross-sectional	2007-2008	Greens	High income country with mixed SEP	Grade 9-10 school students in the urban district of Athens	14.9 [NR]	High	1	Yes	Freq. of SM use	Presence of internet chat room use	Internet gambling practices (not via SM) at least once per week	484
Vandenbosch 2016[147]	Cross-sectional	2010	Belgium	High income country with mixed SEP	School students' part of the MORES Panel Study	15.4 [12-18]	High	1	No	Freq. of SM use	Freq. of chat room use	Use of erotic contact websites in the past 6 months	1,163
Vannucci 2019[148]	Cohort	2016-2017	USA	High income country with mixed SEP	Grade 7-8 middle school students' part of the PANDA Research Project	12.7 [11-14]	Low	1	Yes	Freq. of SM use	Freq. of SM use	Delinquent behaviours in the past 6 months *(via validated tool)*	563

Author and year	Study design	Study period	Country	Equity	Participants	Mean age [range]	Risk of bias	N°. of dp	MA?	Exposure	Exposure measure	Outcome measure	N
Vazquez-Nava 2020[149]	Cross-sectional	NR	Mexico	Low-middle income country with mixed SEP	Urban school students in North-eastern Mexico	NR [13-19]	High	1	Yes	Freq. of SM use	Presence of use of social networks WhatsApp/Facebook *(via validated tool)*	Tobacco smoking *(via validated tool)*	1,328
Vente 2020[150]	Cross-sectional	2016-2018	USA	High income country	Adolescents seen at a paediatric clinic at an urban medical centre	16.8 [12-21]	High	2	No	Freq. of SM use	Use of ≥4 SM applications per day	Sexting	179
										Time Spent on SM	Time spent on SM per day	Sexting	179
Wana 2019[151]	Cross-sectional	2017	Ethiopia	Low-middle income country	Pre-college students residing in Adama Town	NR	Mod	1	No	Freq. of SM use	Presence of SM use	Risky sexual behaviour	346
Ward 2022[152]	Cross-sectional	2017-2018	USA	High income country	Adolescents in the Seattle metro area part of a larger longitudinal experimental study	18.4 [15-20]	Mod	3	Yes	Freq. of SM use	Freq. of Facebook use per day	Past month typical drinks per week	274
										Freq. of SM use	Freq. of Instagram use per day	Past month typical drinks per week	274
										Freq. of SM use	Freq. of Facebook use per day	Past month peak drinks per occasion	274
Whitehill 2020[153]	Cross-sectional	2018	USA	High income country with mixed SEP	Adolescents (aged 15-19) residing in US states with legalised retail cannabis	NR [15-19]	High	4	Yes	Freq. of SM use	Freq. of SM use	Lifetime cannabis use	469
										Freq. of SM use	Presence of Facebook use	Lifetime cannabis use	469
										Time spent on SM	Time spent on SM per day	Lifetime cannabis use	469
										Freq. of SM use	Presence of Instagram use	Lifetime cannabis use	469
Widman 2014[154]	Cross-sectional	2014	USA	High income country	Grade 9-10 high school students' part of a larger study	17.4 [16-19]	High	2	Yes	Freq. of SM use	Used technology based sexual communication to communicate with dating partners about using condoms	Inconsistent condom use in the past 6 months	176
											Used technology based sexual communication to communicate with dating partners about risk of pregnancy	Inconsistent condom use in the past 6 months	176
Worku 2022	Cross-sectional	2021	Ethiopia	Low-middle income country with low SEP	Female high school students at selected high schools of Yeka Sub-city, Addis Ababa	NR [14-16]	Mod	1	No	Time Spent on SM	Stayed more than 2 hrs/day on SM	Low dietary diversity *(via FANTA)*	284

Author and year	Study design	Study period	Country	Equity	Participants	Mean age [range]	Risk of bias	N°. of dp	MA?	Exposure	Exposure measure	Outcome measure	N
Wulff 2021[155]	Cross-sectional	2015	Germany	High income country	Adolescent obesity therapy participants	NR [11-17]	Mod	1	No	Freq. of SM use	Freq. of WhatsApp use	Physical inactivity (exercised 0-2 days per week)	228
Yao 2022[156]	Cross-sectional	NR	China	High income country	Grade 4-10 elementary and middle school students	13.35 [6-18]	Mod	2	No	Exposure to health-risk behaviour content	Exposure to content (including text and pictures) about drinking or smoking (e.g., saw drinking-related information)	Tobacco and alcohol use	1,491

Legend: Where exposure ascertainment was via objectively recorded social media usage data, independent of user reports or via a validated measurement tool, this is stated in italics; where outcome ascertainment was via independent clinical assessment, a validated tool, or medical/administrative records, this is stated in italics. All other measures are self-report. An adapted Newcastle Ottawa Scale (NOS) was used to assess risk of bias for cross-sectional and cohort studies and the Cochrane risk of bias 2 tool for randomised trials (RoB-2) was used for randomised control trial studies. All included studies were assessed using synthesis without meta-analysis (SWiM), excluding one study[74] which was included due to potential double counting of study participants; we were however able to include estimates from this studies in meta-analysis stratified by outcome where this issue did not occur. Abbreviations: ADHD = Attention deficit hyperactivity disorder; ASA24 = Dietary Assessment Tool; AUDIT/C = Alcohol Use Disorders Identification Test; CAGI/GPSS = Gambling Problem Severity Subscale of the Canadian Adolescent Gambling Index; dp = number of datapoints; DSM-IV-MR-J = Diagnostic and Statistical Manual of Mental Disorders-IV-Multiple Response- Adapted for Juveniles (assessment of adolescent gambling); ESP = Spain; FIN = Finland; Freq = Frequency; HEI = Healthy Eating Index; KOR = South Korea; MA = Study included in meta-analysis, subgroup or sensitivity analysis; Mod = Moderate RoB; N = Number of study participants; NR = Not reported; PAQ-C/A = Physical Activity Questionnaire for Children and Adolescents; RCS = Repeat cross-sectional study; RCT = Randomised control trial; RoB-2 = Cochrane risk of bias 2 tool for randomised trials; SBS = Sexting Behaviour Scale; SDQ = Strengths and Difficulties Questionnaire; SEP = Socioeconomic position; SOGS-RA = South Oaks Gambling Screen-Revised for Adolescents; T = Timepoint; UK = United Kingdom; USA = United States; WHO = World Health Organisation; YSR = Youth Self-Report; and Δ = Change.

Figure A. Map of geographical distribution of included study populations

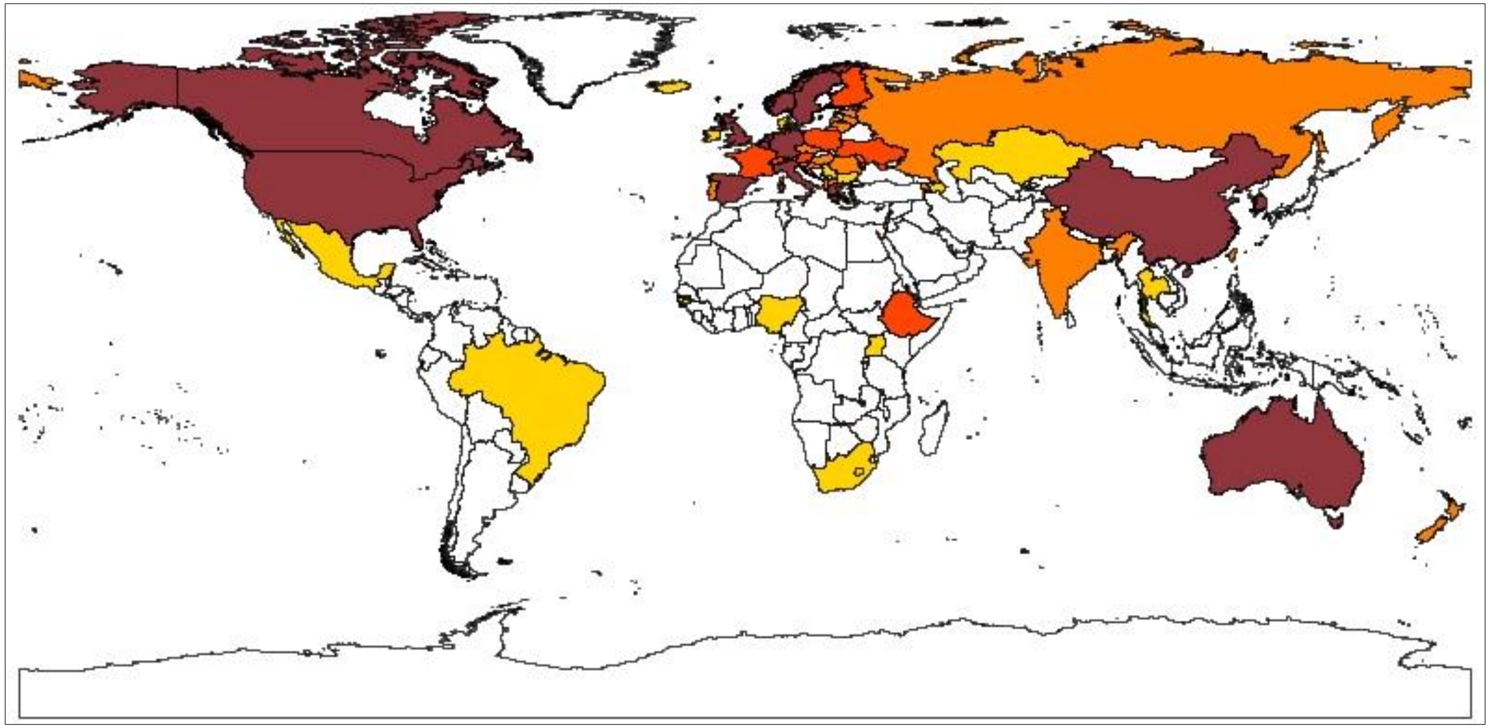

Key

	>4 studies
	3 studies
	2 studies
	1 study
	No data

Legend: Studies undertaken in more than one country contribute multiple datapoints to the map. [41,61,63,133,]

Appendix 12. Characteristics of excluded studies

Table A. List of studies excluded at full text screening (n=571 studies) with reasons for exclusion

Author and year	Title	Publication source	Reason for exclusion
Abara 2014	Understanding internet sex-seeking behaviour and sexual risk among young men who have sex with men: evidence from a cross-sectional study.	Sexually Transmitted Infections	Incorrect population
Abdi 2015	Personal, social, and environmental risk factors of problematic gambling among high school adolescents in Addis Ababa, Ethiopia	Journal of Gambling Studies	Incorrect exposure
Abed-Ali 2018	Violence among high school female students in Baghdad city	Indian Journal of Public Health Research and Development	Incorrect exposure
Acar 2020	Eating attitudes and physical appearance comparison with others in daily life versus on social media in adolescents	Journal of Adolescent Health	Incorrect study type: conference proceeding or abstract
Adam 2011	When do online sexual fantasies become reality? The contribution of erotic chatting via the Internet to sexual risk-taking in gay and other men who have sex with men.	Health Education Research	Incorrect exposure
Adams 2010	Correlates of physical activity in young American Indian children: lessons learned from the Wisconsin Nutrition and Growth Study	Journal of Public Health Management and Practice	Incorrect exposure
Adams 2019	Predictors of overweight and obesity in American Indian families with young children	Journal of Nutrition Education and Behaviour	Incorrect exposure
Adebayo 2006	Gender, internet use, and sexual behaviour orientation among young Nigerians	Cyberpsychology and Behaviour	Incorrect exposure
Afolabi 2015	Media exposure and weight concern?	Child and Adolescent Health Issues	Incorrect study type: commentary/editorial/non-systematic review/theses/book chapter(s)
Agaku 2014	Trends in exposure to pro-tobacco advertisements over the Internet, in newspapers/magazines, and at retail stores among U.S. middle and high school students, 2000-2012	Preventive Medicine	Incorrect exposure
Aggio 2012	Temporal relationships between screen-time and physical activity with cardiorespiratory fitness in English schoolchildren: a 2-year longitudinal study.	Preventive Medicine	Incorrect exposure
Agurcia-Parker 2009	An investigation into the relationship between screen time, consumption of advertised foods, and physical activity among Texas 4th grade elementary school children	Dissertation Abstracts International: Section B: The Sciences and Engineering	Incorrect study type: commentary/editorial/non-systematic review/theses/book chapter(s)
Ahern 2015	Risky behaviours and social networking sites	Journal of Psychosocial Nursing & Mental Health Services	Incorrect study type: commentary/editorial/non-systematic review/theses/book chapter(s)
Aires 2010	A 3-year longitudinal analysis of changes in fitness, physical activity, fatness, and screen time	Acta Paediatrica, International Journal of Paediatrics	Incorrect exposure
Aires 2010	A 3-year longitudinal analysis of changes in Body Mass Index	International Journal of Sports Medicine	Incorrect exposure

Author and year	Title	Publication source	Reason for exclusion
Al-Ajlouni 2018	Partner meeting venue typology and sexual risk behaviours among French men who have sex with men.	International Journal of STD & AIDS	Incorrect exposure
Albert 2018	#consumingitall: Understanding the complex relationship between media consumption and eating behaviours	Dissertation Abstracts International: Section B: The Sciences and Engineering	Incorrect study type: commentary/editorial/non-systematic review/theses/book chapter(s)
Albury 2018	Young people, digital media research and counter public sexual health	Sexualities	Incorrect study type: commentary/editorial/non-systematic review/theses/book chapter(s)
Alghadir 2020	Differences among Saudi and expatriate students: Body composition indices, sitting time associated with media use and physical activity pattern	International Journal of Environmental Research and Public Health	Incorrect exposure
Alhabash 2021	Trick or drink: offline and social media hierarchical normative influences on Halloween celebration drinking	Health Communication	Incorrect population
Al-Hamdani 2021	Perceptions and experiences of vaping among youth and young adult e-cigarette users: considering age, gender, and tobacco use	Journal of Adolescent Health	Incorrect population
Al-Hamdani 2022	Do perceptions and experiences of vaping among youth and young adults differ by device type?	Addiction Research & Theory	No relevant outcome(s)
Al-Hazzaa 2011	Physical activity, sedentary behaviours, and dietary habits among Saudi adolescents relative to age, gender, and region	The International Journal of Behavioural Nutrition and Physical Activity	Incorrect exposure
Al-Hazzaa 2019	Activity energy expenditure, screen time and dietary habits relative to gender among Saudi youth: interactions of gender with obesity status and selected lifestyle behaviours	Asia Pacific Journal of Clinical Nutrition	Incorrect exposure
Alhusaini 2020	Cross-cultural variation in BMI, sedentary behaviour, and physical activity in international schoolgirls residing in Saudi Arabia	International Journal of Environmental Research and Public Health	Incorrect exposure
Allen 2017	Mobile phone and internet use mostly for sex-seeking and associations with sexually transmitted infections and sample characteristics among black/African American and Hispanic/Latino men who have sex with men in 3 US cities	Sexually Transmitted Diseases	Incorrect population
Allen 2018	The dirt on clean eating: a cross sectional analysis of dietary intake, restrained eating, and opinions about clean eating among women.	Nutrients	Incorrect population
Allender 2011	Associations between activity-related behaviours and standardized BMI among Australian adolescents	Journal of Science and Medicine in Sport	Incorrect exposure
Alosaimi 2016	Smartphone addiction among university students in Riyadh, Saudi Arabia.	Saudi Medical Journal	Incorrect population

Author and year	Title	Publication source	Reason for exclusion
Altenburg 2012	Direction of the association between body fatness and self-reported screen time in Dutch adolescents	International Journal of Behavioural Nutrition and Physical Activity	Incorrect exposure
Altenburg 2017	Actual and perceived weight status and its association with slimming and energy-balance related behaviours in 10- to 12-year-old European children: the ENERGY-project	Paediatric Obesity	Incorrect exposure
Alvarez-Jimenez 2019	HORYZONS trial: Protocol for a randomised controlled trial of a moderated online social therapy to maintain treatment effects from first-episode psychosis services	BMJ Open	Incorrect study type: commentary/editorial/non-systematic review/theses/book chapter(s)
Ammouri 2004	Correlates of exercise participation in adolescents	Correlates of Exercise Participation in Adolescents	Incorrect exposure
Amornsriwata-nakul 2017	Are Thai children and youth sufficiently active? Prevalence and correlates of physical activity from a nationally representative cross-sectional study	International Journal of Behavioural Nutrition and Physical Activity	Incorrect exposure
Andrie 2019	Gambling involvement and problem gambling correlates among European adolescents: results from the European Network for Addictive Behaviour study.	Social Psychiatry and Psychiatric Epidemiology	Incorrect exposure
Arie 2014	Doctors and teachers receive new guidance on the internet's effect on young people's sex lives and relationships	BMJ	Incorrect study type: commentary/editorial/non-systematic review/theses/book chapter(s)
Armstrong 2018	An exploration of how simulated gambling games may promote gambling with money	Journal of Gambling Studies	Incorrect study type: commentary/editorial/non-systematic review/theses/book chapter(s)
Arriscado 2014	Factors associated with low adherence to a Mediterranean diet in healthy children in northern Spain	Appetite	Incorrect exposure
Arsad 2021	A systematic review of immersive social media activities and risk factors for sexual boundary violations among adolescents	IUM Medical Journal Malaysia	Incorrect study type: systematic review
Arseniev-Koehler 2014	Peer influence on undergraduates' intention to get drunk by communication formats	Alcoholism: Clinical and Experimental Research	Incorrect study type: conference proceeding or abstract
Asad 2015	Screen-based behaviours of adolescents in Bangladesh	European Journal of Epidemiology	Incorrect exposure
Aschbrenner 2019	Randomized trial of a lifestyle intervention for young adults with serious mental illness in community mental health centres	Schizophrenia Bulletin	Incorrect study type: conference proceeding or abstract
Ashford 2017	Advertising exposure and use of e-cigarettes among female current and former tobacco users of childbearing age	Public Health Nursing	Incorrect population

Author and year	Title	Publication source	Reason for exclusion
Asut 2019	Relationships between screen time, internet addiction and other lifestyle behaviours with obesity among secondary school students in the Turkish Republic of Northern Cyprus	Turkish Journal of Paediatrics	Incorrect exposure
Athauda 2020	Factors influencing alcohol use among adolescents in South Asia: a systematic review	Journal of Studies on Alcohol and Drugs	Incorrect study type: systematic review
Atkin 2013	Determinants of change in children's sedentary time	Plos One	Incorrect exposure
Atkin 2021	Adolescent time use and mental health: a cross-sectional, compositional analysis in the Millennium Cohort Study	BMJ Open	Incorrect exposure
Atkinson 2010	Online behaviours of adolescents: victims, perpetrators, and Web 2.0.	Journal of Sexual Aggression	Incorrect study type: commentary/editorial/non-systematic review/theses/book chapter(s)
Attwood 2017	Using a mobile health application to reduce alcohol consumption: a mixed-methods evaluation of the drinkaware track & calculate units' application	BMC Public Health	Incorrect exposure
Atwood 2017	Adolescent problematic digital behaviours associated with mobile devices	North American Journal of Psychology	Incorrect exposure
Bae 2018	Selective exposure to misleading information in the new media environment by at-risk youth: A study of pro-smoking YouTube videos	Dissertation Abstracts International Section A: Humanities and Social Sciences	Incorrect study type: commentary/editorial/non-systematic review/theses/book chapter(s)
Bai 2016	The associations of youth physical activity and screen time with fatness and fitness: The 2012 NHANES national youth fitness survey	Plos One	Incorrect exposure
Baird 2016	Social media and substance use	Journal of Addictions Nursing	Incorrect study type: commentary/editorial/non-systematic review/theses/book chapter(s)
Baird 2019	Teens and vaping: what you need to know	Journal of Addictions Nursing	Incorrect study type: commentary/editorial/non-systematic review/theses/book chapter(s)
Bakhali 2016	Exploring the impact of information seeking behaviours of online health consumers in the Arab world	Studies in Health Technology & Informatics	Incorrect population
Balding 2015	Young People into 2015: The health-related behaviour questionnaire results for over 78,000 young people	Education & Health	Incorrect study type: commentary/editorial/non-systematic review/theses/book chapter(s)
Bareghamyan 2021	Sexual and reproductive health of adolescent girls	Akusherstvo i Ginekologiya	Unable to source full text

Author and year	Title	Publication source	Reason for exclusion
Barman-Adhikari 2016	Social networking technology use and engagement in HIV-related risk and protective behaviours among homeless youth	Journal of Health Communication	Incorrect population
Barnes 2015	Maternal correlates of objectively measured physical activity in girls	Maternal and Child Health Journal	Incorrect exposure
Barrere 2015	Oncogenic human papillomavirus infections in 18- to 24-year-old female online daters	Sexually Transmitted diseases	Incorrect exposure
Bass III 2016	Living life online: talking to parents about social media	Contemporary Paediatrics	Incorrect study type: commentary/editorial/non-systematic review/theses/book chapter(s)
Bauermeister 2014	Sexting among young men who have sex with men: results from a national survey	Journal of Adolescent Health	Incorrect exposure
Baumgartner 2010	Assessing causality in the relationship between adolescents' risky sexual online behaviour and their perceptions of this behaviour	Journal of Youth and Adolescence	Duplicate sample
Baumgartner 2012	Unwanted online sexual solicitation and online sexual risk behaviour	Encyclopaedia of Cyber Behaviour, Vols. I - III.	Incorrect population
Bell 2015	The debate over digital technology and young people	BMJ	Incorrect study type: commentary/editorial/non-systematic review/theses/book chapter(s)
Benotsch 2013	Sexting, substance use, and sexual risk behaviour in young adults	Journal of Adolescent Health	Incorrect exposure
Berchtold 2018	Daily internet time: towards an evidence-based recommendation?	European Journal of Public Health	Incorrect exposure
Bergman 2016	The association between alcohol use disorder and social network site engagement among treatment seeking emerging adults	Alcoholism: Clinical and Experimental Research	Incorrect study type: conference proceeding or abstract
Bergman 2018	Instagram participation and substance use among emerging adults: the potential perils of peer belonging	Cyberpsychology, Behaviour and Social Networking	Incorrect population
Bergman 2020	Associations between substance use and Instagram participation to inform social network-based screening models: multimodal cross-sectional study	Journal of Medical Internet Research	Incorrect population
Berner 2013	Lifestyle and depressive risk factors associated with problematic internet use in adolescents in an Arabian Gulf culture	Journal of Addiction Medicine	Incorrect population

Author and year	Title	Publication source	Reason for exclusion
Bernstein 2018	Child and adolescent psychiatry case studies: a broad range of ethical dilemmas	Journal of the American Academy of Child and Adolescent Psychiatry	Incorrect study type: conference proceeding or abstract
Beullens 2016	A conditional process analysis on the relationship between the use of social networking sites, attitudes, peer norms, and adolescents' intentions to consume alcohol	Media Psychology	No relevant outcome(s)
Beutel 2011	Regular and problematic leisure-time Internet use in the community: results from a German population-based survey	Cyberpsychology, Behaviour and Social Networking	Incorrect population
Bevelander 2018	Youth's social network structures and peer influences: study protocol MyMovez project - Phase I	BMC Public Health	Incorrect study type: commentary/editorial/non-systematic review/theses/book chapter(s)
Bharucha 2018	Social network use and youth well-being: a study in India	Safer Communities	Incorrect study type: qualitative
Bhuyan 2019	How vaping became fire: Snap streaks, social influencers, and bubble gum	American Sociological Association	Incorrect study type: conference proceeding or abstract
Bickham 2020	Dating app use and sexual risk behaviours: examining aspects of use and motivation	Journal of Adolescent Health	Incorrect study type: conference proceeding or abstract
Biddle 2014	Interventions designed to reduce sedentary behaviours in young people: a review of reviews	British Journal of Sports Medicine	Incorrect study type: systematic review
Bilgrami 2017	Health implications of new-age technologies: a systematic review	Minerva Pediatrica	Incorrect study type: systematic review
Black 2013	Actual versus perceived peer sexual risk behaviour in online youth social networks	Translational Behavioural Medicine	Incorrect exposure
Blanchard 2013	Adolescent perceptions of digital play: A study in third-person effects	Dissertation Abstracts International Section A: Humanities and Social Sciences	Incorrect study type: commentary/editorial/non-systematic review/theses/book chapter(s)
Blasco 2019	Pattern of internet use and parental monitoring of social networks as a predictor of sexting in adolescents: A gender per	Revista de Psicología y Educación	Potentially relevant non-English language
Blaszczynski 2016	Mental health and online, land-based, and mixed gamblers	Journal of Gambling Studies	Incorrect population
Blaya 2015	The young people and risk-taking on the Internet	Neuropsychiatrie de l'Enfance et de l'Adolescence	Incorrect study type: commentary/editorial/non-systematic review/theses/book chapter(s)

Author and year	Title	Publication source	Reason for exclusion
Blazquez Barba 2018	Use of new technologies by adolescents in the search for health information	Atencion Primaria	Potentially relevant non-English language
Bleakley 2011	A model of adolescents' seeking of sexual content in their media choices	Journal of Sex Research	Incorrect exposure
Bobkowski 2012	'Hit me up and we can get down': US youths' risk behaviours and sexual self-disclosure in MySpace profiles	Journal of Children and Media	Incorrect population
Boggs 2017	The impact of exposure to alcohol advertisements on adolescents: A literature review	International Public Health Journal	Incorrect study type: commentary/editorial/non-systematic review/theses/book chapter(s)
Bonnaire 2012	Internet gambling: What are the risks?	L'Encéphale	Incorrect study type: commentary/editorial/non-systematic review/theses/book chapter(s)
Borajy 2019	Relationship of electronic device usage with obesity and speech delay in children	Family Medicine & Primary Care Review	Incorrect exposure
Borden 2019	Vaping marketers take aim at youth through social media	Chest Physician	Incorrect study type: commentary/editorial/non-systematic review/theses/book chapter(s)
Bousono Serrano 2017	Substance use or abuse, internet use, psychopathology, and suicidal ideation in adolescents	Adicciones	Incorrect exposure
Boyland 2016	Advertising as a cue to consume: a systematic review and meta-analysis of the effects of acute exposure to unhealthy food and non-alcoholic beverage advertising on intake in children and adults	The American Journal of Clinical Nutrition	Incorrect study type: systematic review
Boyle 2018	The social mindfeed project: Using objective assessment methods to better understand the nature of social-media based peer alcohol influence	Alcoholism: Clinical and Experimental Research	Incorrect study type: conference proceeding or abstract
Bozzola 2019	Adolescence, smartphone and tablets: A review of the literature	Italian Journal of Pediatrics	Incorrect study type: conference proceeding or abstract
Brailovskaia 2020	Relationship between depression symptoms, physical activity, and addictive social media use	Cyber Psychology, Behavior & Social Networking	Incorrect exposure
Branley 2018	Risky behaviour via social media: The role of reasoned and social reactive pathways.	Computers in Human Behaviour	Incorrect population
Braun-Courville 2009	Exposure to sexually explicit web sites and adolescent sexual attitudes and behaviours	Journal of Adolescent Health	Incorrect exposure

Author and year	Title	Publication source	Reason for exclusion
Broaddus 2015	Social media use and high-risk sexual behaviour among black men who have sex with men: a three-city study	AIDS and Behaviour	Incorrect population
Brown 2011	Older and newer media: Patterns of use and effects on adolescents' health and wellbeing	Journal of Research on Adolescence	Incorrect study type: commentary/editorial/non-systematic review/theses/book chapter(s)
Brown 2012	Too much, much too young	Therapy Today	Incorrect study type: commentary/editorial/non-systematic review/theses/book chapter(s)
Brunborg 2017	Social media use and episodic heavy drinking among adolescents	Psychological Reports	Duplicate sample
Brunelle 2012	Internet gambling, substance use, and delinquent behaviour: an adolescent deviant behaviour involvement pattern	Psychology of Addictive Behaviours	Incorrect exposure
Buchanan 2018	The effects of digital marketing of unhealthy commodities on young people: A systematic review	Nutrients	Incorrect study type: systematic review
Buhi 2011	Evaluating the internet as an std risk environment for teens: Findings from the communication, health, and teens (ch@t) study	Sexually Transmitted Infections	Incorrect study type: conference proceeding or abstract
Buhi 2013	Evaluating the internet as a sexually transmitted disease risk environment for teens: Findings from the communication, health, and teens study	Sexually Transmitted Diseases	Incorrect exposure
Buhi 2013	Teens, the internet, and STD Risk: Findings and lessons learned from the communication, health, and teens (CH@T) study	Sexually Transmitted Infections	Incorrect study type: conference proceeding or abstract
Bunnell 2015	Intentions to smoke cigarettes among never-smoking US middle and high school electronic cigarette users: National youth tobacco survey, 2011-2013	Nicotine and Tobacco Research	Incorrect exposure
Burgos 2013	The relationship between risky behaviours and perceived victimization in individuals who participate in social networking websites	Dissertation Abstracts International: Section B: The Sciences and Engineering	Incorrect study type: commentary/editorial/non-systematic review/theses/book chapter(s)
Burke 2019	The paradoxical outcomes of observing others' exercise behaviour on social network sites: friends' exercise posts, exercise attitudes, and weight concern	Health Communication	Incorrect population
Burns 2021	Social media preference and condom use behaviours: an analysis of digital spaces with young African American males	Health Education & Behaviour	Incorrect study type: qualitative
Butdabut 2021	Factors predicting sexual risk behaviours of adolescents in North-Eastern Thailand	Studies in Health Technology and Informatics	Incorrect exposure

Author and year	Title	Publication source	Reason for exclusion
Cabrera-Nguyen 2016	Young adults' exposure to alcohol- and marijuana-related content on twitter	Journal of Studies on Alcohol and Drugs	Incorrect population
Caravaca Sanchez 2016	Prevalence and patterns of traditional bullying victimization and cyber-teasing among college population in Spain	BMC Public Health	Incorrect exposure
Card 2017	Exploring the role of sex-seeking apps and websites in the social and sexual lives of gay, bisexual, and other men who have sex with men: a cross-sectional study	Sexual Health	Incorrect exposure
Carrotte 2015	Predictors of "Liking" three types of health and fitness-related content on social media: A cross-sectional study	Journal of Medical Internet Research	Incorrect population
Carrotte 2016	Who 'likes' alcohol? Young Australians' engagement with alcohol marketing via social media and related alcohol consumption patterns	Australian And New Zealand Journal of Public Health	Incorrect population
Castren 2022	Risk factors for excessive social media use differ from those of gambling and gaming in Finnish youth	International Journal of Environmental Research and Public Health	Incorrect exposure
Castro-Calvo 2018	Building bridges between substance and behavioural addictions: Alcohol consumption and their predictive power over internet and cybersex use and abuse in adolescents	Alcoholism: Clinical and Experimental Research	Incorrect study type: conference proceeding or abstract
Cavazos-Rehg 2021	Exploring How Social Media Exposure and Interactions Are Associated with ENDS and Tobacco Use in Adolescents from the PATH Study	Nicotine and Tobacco Research	Duplicate sample
Cemelli 2016	Video games impact lifestyle behaviors in adults	Topics in Clinical Nutrition	Incorrect population
Cen Chen-Sankey 2019	E-cigarette marketing exposure and subsequent experimentation among youth and young adults	Paediatrics	Incorrect exposure
Chan 2017	The role of gay identity confusion and outness in sex-seeking on mobile dating apps among men who have sex with men: a conditional process analysis	Journal of Homosexuality	Incorrect exposure
Chiao 2014	Adolescent Internet use and its relationship to cigarette smoking and alcohol use: A prospective cohort study	Addictive Behaviours	Incorrect exposure
Children & Young People Now 2008	Youth work support needed in new world of social networking	Children & Young People Now	Incorrect study type: commentary/editorial/non-systematic review/theses/book chapter(s)
Cho 2019	Mechanisms of social media effects on attitudes toward e-cigarette use: motivations, mediators, and moderators in a national survey of adolescents	Journal of Medical Internet Research	No relevant outcome(s)

Author and year	Title	Publication source	Reason for exclusion
Chortatos 2020	Comparing three screen-based sedentary behaviours' effect upon adolescents' participation in physical activity: The ESSENS study	Plos One	Incorrect exposure
Clayton 2013	Loneliness, anxiousness, and substance use as predictors of Facebook use	Computers in Human Behaviour	Incorrect population
Coates 2018	Does social media food marketing influence children's food intake and preferences?	Obesity Facts	Incorrect study type: conference proceeding or abstract
Cook 2013	Online network influences on emerging adults' alcohol and drug use	Journal of Youth and Adolescence	Incorrect population
Cookingham 2015	The impact of social media on the sexual and social wellness of adolescents	Journal of Paediatric and Adolescent Gynaecology	Incorrect study type: commentary/editorial/non-systematic review/theses/book chapter(s)
Coreas 2021	Smoking susceptibility and tobacco media engagement among youth never smokers	Paediatrics	Duplicate sample
Cox 2021	Profiles of parenting in the digital age: associations with adolescent alcohol and marijuana use	Journal of Studies on Alcohol and Drugs	Incorrect exposure
Cruz 2016	Use of social networking applications (apps) and meeting sites in patients with acute HIV infection in a specialized clinic in Mexico City	Journal of the International AIDS Society	Incorrect study type: conference proceeding or abstract
Cruz 2019	Tobacco marketing and subsequent use of cigarettes, e-cigarettes, and hookah in adolescents	Nicotine and Tobacco Research	Incorrect exposure
Cubitt 2014	Social networking and risk-taking behaviour: the Lynx effect	Journal of the International Society for Burn Injuries	Incorrect study type: commentary/editorial/non-systematic review/theses/book chapter(s)
Cui 2018	Patterns of online and offline connectedness among gay, bisexual, and other men who have sex with men	AIDS & Behaviour	Incorrect exposure
Cureau 2018	Associations of multiple unhealthy lifestyle behaviours with overweight/obesity and abdominal obesity among Brazilian adolescents: A country-wide survey	Nutrition, Metabolism and Cardiovascular Diseases	Incorrect exposure
Curlee 2021	The role of social media use in adolescent alcohol use accounting for peer alcohol use	Dissertation Abstracts International: Section B: The Sciences and Engineering	Incorrect study type: commentary/editorial/non-systematic review/theses/book chapter(s)
Curtis 2018	Meta-analysis of the association of alcohol-related social media use with alcohol consumption and alcohol-related problems in adolescents and young adults	Alcoholism: Clinical and Experimental Research	Incorrect study type: systematic review

Author and year	Title	Publication source	Reason for exclusion
da Costa 2020	Association between lifestyle behaviours and health-related quality of life in a sample of Brazilian adolescents	International Journal of Environmental Research and Public Health	No relevant outcome(s)
da Costa 2022	Movement behaviours and their association with depressive symptoms in Brazilian adolescents: A cross-sectional study	Journal of Sport and Health Science	Duplicate sample
Dai 2017	Geographic variations in electronic cigarette advertisements on Twitter in the United States	International Journal of Public Health	Incorrect population
Dalisay 2022	Exposure to tobacco and betel nut content on social media, risk perceptions, and susceptibility to peer influence among early adolescents in Guam	Addictive Behaviours Reports	No relevant outcome(s)
D'Angelo 2019	Facebook-induced friend shift and identity shift: a longitudinal study of Facebook posting and collegiate drinking	Cyberpsychology, Behaviour and Social networking	Incorrect population
Das 2016	Interventions for adolescent substance abuse: an overview of systematic reviews	Journal of Adolescent Health	Incorrect study type: systematic review
Davis 2021	Temporal, sex-specific, social media-based alcohol influences during the transition to college	Substance Use & Misuse	Incorrect population
Dawson 2019	Exploring technology-mediated social interactions among adolescents with ADHD	Dissertation Abstracts International: Section B: The Sciences and Engineering	Incorrect study type: commentary/editorial/non-systematic review/theses/book chapter(s)
de Bruijn 2016	European longitudinal study on the relationship between adolescents' alcohol marketing exposure and alcohol use	Addiction	Incorrect exposure
Deforche 2015	Changes in weight, physical activity, sedentary behaviour, and dietary intake during the transition to higher education: A prospective study	International Journal of Behavioural Nutrition and Physical Activity	Incorrect exposure
Depue 2015	Encoded exposure to tobacco use in social media predicts subsequent smoking behaviour	American Journal of Health Promotion	Incorrect population
De-Sola 2019	Cell phone use habits among the Spanish population: contribution of applications to problematic use	Frontiers in Psychiatry	Incorrect exposure
Diaz 2022	Online tobacco advertising and current chew, dip, snuff, and snus use among youth and young adults, 2018-2019	International Journal of Environmental Research and Public Health	Incorrect population
Divecha 2012	Tweeting about testing: Do low-income, parenting adolescents and young adults use new media technologies to communicate about sexual health?	Perspectives on Sexual and Reproductive Health	Incorrect exposure

Author and year	Title	Publication source	Reason for exclusion
Doggett 2019	Examining the association between exposure to various screen time sedentary behaviours and cannabis use among youth in the COMPASS study	Society of Social Medicine	Incorrect exposure
Dolcini 2014	A new window into adolescents' worlds: The impact of online social interaction on risk behaviour	Journal of Adolescent Health	Incorrect study type: commentary/editorial/non-systematic review/theses/book chapter(s)
Domingues-Montanari 2017	Clinical and psychological effects of excessive screen time on children	Journal of Paediatrics and Child Health	Incorrect study type: commentary/editorial/non-systematic review/theses/book chapter(s)
Doornwaard 2015	Adolescents' use of sexually explicit Internet material and their sexual attitudes and behaviour: Parallel development and directional effects	Developmental Psychology	Incorrect exposure
Dowdell 2011	Original research: online social networking patterns among adolescents, young adults, and sexual offenders	The American Journal of Nursing	No relevant outcome(s)
Dowdell 2011	Risky internet behaviours of middle-school students: Communication with online strangers and offline contact	Computers Informatics Nursing	No relevant outcome(s)
Dowdell 2022	Problematic behaviours and predicting online risk behaviours in high school students	The Journal of School Nursing	Incorrect exposure
Dowell 2009	Clustering of internet risk behaviours in a middle school student population	Journal of School Health	Incorrect exposure
Drescher 2011	Caffeine and screen time in adolescence: associations with short sleep and obesity	Journal of Clinical Sleep Medicine	Incorrect exposure
Dubuc 2020	Lifestyle habits predict academic performance in high school students: The adolescent student academic performance longitudinal study (ASAP)	International Journal of Environmental Research and Public Health	No relevant outcome(s)
Dumas 2019	Am I cool now? Examining the relations between need for popularity, alcohol-related social media posts and heavy drinking among emerging adults	Alcoholism: Clinical and Experimental Research	Incorrect study type: conference proceeding or abstract
Dumas 2021	Everyone loves my beer pong pics! examining feedback on social network sites and its role in shaping young adult binge drinking behaviour	Alcoholism: Clinical and Experimental Research	Incorrect study type: conference proceeding or abstract
Dumas 2021	Likelihood of posting alcohol-related content scale	Alcoholism: Clinical and Experimental Research	Incorrect study type: conference proceeding or abstract
Dunaev 2016	Seeking safe sex information: Social media use, gossip, and sexual health behaviour among minority youth	Journal of Adolescent Health	Incorrect study type: conference proceeding or abstract

Author and year	Title	Publication source	Reason for exclusion
Dunlop 2016	Exposure to internet-based tobacco advertising and branding: results from population surveys of Australian youth 2010-2013	Journal of Medical Internet Research	Incorrect exposure
Duplaga 2020	The use of fitness influencers' websites by young adult women: a cross-sectional study	International Journal of Environmental Research and Public Health	Incorrect population
Durbin 2018	Social media and adolescents: What are the health risks?	Clinical Advisor	Incorrect study type: commentary/editorial/non-systematic review/theses/book chapter(s)
Edler 2022	The role of personality traits and social support in relations of health-related behaviours and depressive symptoms	BMC Psychiatry	Incorrect population
Effertz 2018	The effect of online gambling on gambling problems and resulting economic health costs in Germany	European Journal of Health Economics	Incorrect population
Elavsky 2017	Who are mobile app users from healthy lifestyle websites? Analysis of patterns of app use and user characteristics	Translational Behavioural Medicine	Incorrect exposure
Eleuteri 2017	Identity, relationships, sexuality, and risky behaviours of adolescents in the context of social media	Journal of Physical Education, Recreation & Dance	Incorrect study type: commentary/editorial/non-systematic review/theses/book chapter(s)
Emery 2014	Wanna know about vaping? Patterns of message exposure, seeking and sharing information about e-cigarettes across media platforms	Tobacco Control	Incorrect population
Emory 2019	Lesbian, gay, bisexual, and transgender (LGBT) view it differently than non-LGBT: exposure to tobacco-related couponing, e-cigarette advertisements, and anti-tobacco messages on social and traditional media	Journal of the Society for Research on Nicotine and Tobacco	Incorrect population
Englander 2017	Social media sex: Exploitation or everlasting love?	Journal of the American Academy of Child and Adolescent Psychiatry	Incorrect study type: conference proceeding or abstract
Epstein 2011	Adolescent computer use and alcohol use: What are the role of quantity and content of computer use?	Addictive Behaviours	Incorrect exposure
Epstein-Ngo 2013	Alcohol use, dating aggression, and mindfulness in high risk youth: Preliminary analyses	Alcoholism: Clinical and Experimental Research	Incorrect study type: conference proceeding or abstract
Epstein-Ngo 2014	Alcohol, drugs, and other factors associated with digital dating violence among high risk urban youth	Alcoholism: Clinical and Experimental Research	Incorrect study type: conference proceeding or abstract
Erevik 2017	Sharing of alcohol-related content on social networking sites: frequency, content, and correlates	Journal of Studies on Alcohol and Drugs	Incorrect population

Author and year	Title	Publication source	Reason for exclusion
Escobar-Chaves 2005	Impact of the media on adolescent sexual attitudes and behaviours	Paediatrics	Incorrect study type: systematic review
Eugene 2015	It's more than just a "sext"- a brief discussion on sexting activity among teens	Journal of Adolescent Health	Incorrect study type: commentary/editorial/non-systematic review/theses/book chapter(s)
Evans 2019	Outcomes of the Adelante community social marketing campaign for Latino youth	Health Education Research	Incorrect exposure
Faulkner 2017	'Unintended' audiences of alcohol advertising: exposure and drinking behaviours among Australian adolescents	Journal of Substance Use	Incorrect exposure
Fielding-Singh 2021	Tobacco product promotions remain ubiquitous and are associated with use and susceptibility to use among adolescents	Nicotine and Tobacco Research	Incorrect exposure
Fife 2019	STI testing and documentation via a phone application (APP): Experience with the safe app	Sexually Transmitted Infections	Incorrect study type: conference proceeding or abstract
Floros 2015	Adolescent online gambling in Cyprus: associated school performance and psychopathology	Journal of Gambling Studies	Incorrect exposure
Folkvord 2016	Food advertising and eating behaviour in children	Current Opinion in Behavioural Sciences	Incorrect study type: commentary/editorial/non-systematic review/theses/book chapter(s)
Ford-Jones 2003	Impact of media use on children and youth	Paediatrics and Child Health	Incorrect study type: commentary/editorial/non-systematic review/theses/book chapter(s)
Forsyth 2013	The effect of the internet on teen and young adult tobacco use: A literature review	Journal of Paediatric Health Care	Incorrect study type: systematic review
Frankis 2017	Regular STI testing amongst men who have sex with men and use social media is suboptimal - a cross-sectional study	International journal of STD & AIDS	Incorrect exposure
Friedman 2011	GYT (Get Yourself Tested) Campaign: Getting young people talking and tested and sparking a social movement	Sexually Transmitted Infections	Incorrect study type: conference proceeding or abstract
Friedman 2013	Do you GYT? Evaluation of the first two years of the united states' national get yourself tested campaign	Sexually Transmitted Infections	Incorrect study type: conference proceeding or abstract
Fung 2018	Public health implications of image-based social media: a systematic review of Instagram, Pinterest, Tumblr, and Flickr	The Permanente Journal	Incorrect study type: systematic review

Author and year	Title	Publication source	Reason for exclusion
Gabrielli 2019	A new recall of alcohol marketing scale for youth: measurement properties and associations with youth drinking status	Journal of Studies on Alcohol and Drugs	Duplicate sample
Gainsbury 2014	Are psychology university student gamblers representative of non-university students and general gamblers? a comparative analysis	Journal of Gambling Studies	Incorrect exposure
Gansner 2017	"The internet made me do it": social media and potential for violence in adolescents	Psychiatric Times	Incorrect study type: commentary/editorial/non-systematic review/theses/book chapter(s)
Gebremeskel 2014	Social media use and adolescent risk-taking behaviour	Journal of Adolescent Health	Incorrect study type: conference proceeding or abstract
Geisner 2012	Differences between athletes and non-athletes in risk and health behaviours in graduating high school seniors	Journal of Child & Adolescent Substance Abuse	Incorrect exposure
Gentzke 2022	Tobacco product use and associated factors among middle and high school students - National Youth Tobacco Survey, United States, 2021	Centers for Disease Control and Prevention Surveillance Summaries	Incorrect exposure
Geusens 2016	The association between social networking sites and alcohol abuse among Belgian adolescents: The role of attitudes and social norms	Journal of Media Psychology: Theories, Methods, and Applications	Duplicate sample
Gilliam 2014	Digital media and sexually transmitted infections	Current Opinion in Obstetrics & Gynaecology	Incorrect study type: commentary/editorial/non-systematic review/theses/book chapter(s)
Godinho 2014	Characteristics associated with media use in early adolescence	Cadernos de Saude Publica	Incorrect exposure
Gold 2011	A systematic examination of the use of online social networking sites for sexual health promotion	BMC Public Health	Incorrect study type: systematic review
Golpe 2017	The relationship between consumption of alcohol and other drugs and problematic Internet use among adolescents	Adicciones	Incorrect exposure
Gomez 2020	Minors and online gambling: prevalence and related variables	Journal of Gambling Studies	Exact duplicate
Gommans 2015	Frequent electronic media communication with friends is associated with higher adolescent substance use	International Journal of Public Health	Incorrect exposure
Gonzalez 2015	Use and risks of information and communication technologies in the adolescents from 13 to 18 years	Acta Pediatrica Espanola	Potentially relevant non-English language
Govindappa 2014	Internet use and risk-taking behaviours among adolescents	Indian Journal of Paediatrics	Incorrect study type: commentary/editorial/non-systematic review/theses/book chapter(s)

Author and year	Title	Publication source	Reason for exclusion
Grant 2014	Social norms and social networking sites: The role of Facebook in predicting alcohol use among first-year undergraduate students	Alcoholism: Clinical and Experimental Research	Incorrect study type: conference proceeding or abstract
Greene 2020	Social media use among adolescents being evaluated for sexual abuse	Journal of Paediatric and Adolescent Gynaecology	Incorrect study type: conference proceeding or abstract
Griffiths 2010	Adolescent gambling on the internet: A review	International Journal of Adolescent Medicine and Health	Incorrect study type: commentary/editorial/non-systematic review/theses/book chapter(s)
Griffiths 2013	Adolescent gambling via social networking sites: A brief overview	Education & Health	Incorrect study type: commentary/editorial/non-systematic review/theses/book chapter(s)
Groom 2021	The influence of friends on teen vaping: a mixed-methods approach	International Journal of Environmental Research and Public Health	No relevant outcome(s)
Guerrero 2019	Screen time and problem behaviours in children: exploring the mediating role of sleep duration	International Journal of Behavioural Nutrition & Physical Activity	Incorrect population
Gulec 2020	Social media usage and health promoting lifestyle in profile related socio-demographic factors in Turkey	Health Promotion Perspectives	Incorrect population
Gumus 2021	The relationship between adolescents' social media addiction and eating behaviours	Clinical Nutrition	Incorrect study type: conference proceeding or abstract
Gupta 2016	A systematic review of the impact of exposure to internet-based alcohol-related content on young people's alcohol use behaviours	Alcohol and Alcoholism	Incorrect study type: systematic review
Gupta 2018	The association between exposure to social media alcohol marketing and youth alcohol use behaviours in India and Australia	BMC Public Health	Incorrect population
Gutierrez 2013	Internet and cell phone usage associated with risky situations of child sexual exploitation	Salud Mental	No relevant outcome(s)
Gutierrez 2015	The impact of e-cigarette advertisements on e-cigarette initiation among middle and high school students	Drug and Alcohol Dependence	Incorrect study type: conference proceeding or abstract
Guy 2012	Internet pornography and adolescent health: Early findings on effects of online pornography on adolescents show associations with risky behaviour	Medical Journal of Australia	Incorrect study type: commentary/editorial/non-systematic review/theses/book chapter(s)
Hadjipanayis 2019	Social media and children: what is the paediatrician's role?	European Journal of Paediatrics	Incorrect study type: commentary/editorial/non-systematic review/theses/book chapter(s)

Author and year	Title	Publication source	Reason for exclusion
Hakim 2018	Correlates of attempting to quit smoking among adults in Bangladesh	Addictive Behaviours Reports	Incorrect population
Hamm 2014	A systematic review of the use and effectiveness of social media in child health	BMC Paediatrics	Incorrect study type: systematic review
Han 2021	Identifying emerging predictors for adolescent electronic nicotine delivery systems use: A machine learning analysis of the Population Assessment of Tobacco and Health Study	Preventive Medicine	Duplicate sample
Hands 2011	The associations between physical activity, screen time and weight from 6 to 14 yrs: The Raine Study	Journal of Science and Medicine in Sport	Incorrect exposure
Hansen 2018	Electronic cigarette marketing and smoking behaviour in adolescence: A cross-sectional study	ERJ Open Research	Incorrect exposure
Hardon 2014	Ethnographies of youth drug use in Asia	International Journal of Drug Policy	Incorrect study type: commentary/editorial/non-systematic review/theses/book chapter(s)
Hassan 2010	Using technology to improve adolescent healthcare	Current Opinion in Paediatrics	Incorrect study type: commentary/editorial/non-systematic review/theses/book chapter(s)
Hendriks 2021	Causal effects of alcohol-related Facebook posts on drinking behaviour: longitudinal experimental study	Journal of Medical Internet Research	Incorrect population
Henry 2009	Food and beverage brands that market to children and adolescents on the Internet: a content analysis of branded web sites	Journal of Nutrition Education & Behaviour	Incorrect exposure
Henzel 2021	Hooked on virtual social life. Problematic social media use and associations with mental distress and addictive disorders	Plos One	Incorrect exposure
Herbert 2017	Exposure and engagement with tobacco- and e-cigarette related social media	Journal of Adolescent Health	Incorrect exposure
Hieftje 2013	Electronic media-based health interventions promoting behaviour change in youth: A systematic review	JAMA Paediatrics	Incorrect study type: systematic review
Hill 2019	Prevalence and correlates of lifetime and recent HIV testing among men who have sex with men (MSM) who use mobile geo-social networking applications in Greater Tokyo.	Plos One	Incorrect exposure
Hingle 2013	up34 stealth health: youth innovation, mobile technology, online social networking, and informal learning to promote physical activity	Journal of Nutrition Education & Behaviour	Incorrect study type: conference proceeding or abstract

Author and year	Title	Publication source	Reason for exclusion
Ho 2001	Computer usage and its relationship with adolescent lifestyle in Hong Kong	Journal of Adolescent Health	Incorrect exposure
Hoare 2020	Association of child and adolescent mental health with adolescent health behaviours in the UK Millennium Cohort	JAMA Network Open	Incorrect exposure
Hoffmann 2019	High sedentary time in children is not only due to screen media use: A cross-sectional study	BMC Paediatrics	Incorrect exposure
Hollingdale 2014	The effect of online violent video games on levels of aggression	Plos One	Incorrect population
Holloway 2014	Acceptability of smartphone application-based HIV prevention among young men who have sex with men	AIDS and behaviour	Incorrect population
Holt 2012	HIV testing, gay community involvement and internet use: social and behavioural correlates of HIV testing among Australian men who have sex with men	AIDS and behaviour	Incorrect population
Hospers 2002	Chatters on the Internet: a special target group for HIV prevention	AIDS Care - Psychological and Socio-Medical Aspects of AIDS/HIV	Incorrect population
Hospers 2005	A new meeting place: Chatting on the internet, e-dating and sexual risk behaviour among Dutch men who have sex with men	AIDS	Incorrect exposure
Howe 2016	Gotta catch'em all! Pokemon GO and physical activity among young adults: difference in differences study	BMJ	Incorrect exposure
Huang 2012	The effects of online and offline friendship networks and media use on alcohol and smoking behaviours	Alcoholism: Clinical and Experimental Research	Incorrect study type: conference proceeding or abstract
Huang 2014	The interplay of friendship networks and social networking sites: longitudinal analysis of selection and influence effects on adolescent smoking and alcohol use	American Journal of Public Health	Incorrect study type: commentary/editorial/non-systematic review/theses/book chapter(s)
Huang 2017	Trends and correlates of hookah use among high school students in North Carolina	North Carolina Medical Journal	Incorrect exposure
Hur 2013	Growing up in the web of social networking: Adolescent development and social media	Adolescent Psychiatry	Incorrect study type: commentary/editorial/non-systematic review/theses/book chapter(s)
Hutton 2019	mhealth interventions to reduce alcohol use in young people: a systematic review of the literature	Comprehensive Child and Adolescent Nursing	Incorrect study type: systematic review

Author and year	Title	Publication source	Reason for exclusion
Hwang 2009	Being young and feeling blue in Taiwan: Examining adolescent depressive mood and online and offline activities	New Media & Society	No relevant outcome(s)
Ilakkuvan 2019	Patterns of social media use and their relationship to health risks among young adults	The Journal of Adolescent Health	Incorrect population
Ioannidis 2018	Problematic internet use as an age-related multifaceted problem: Evidence from a two-site survey	Addictive Behaviours	Incorrect population
Ishaque 2012	Frequency of and factors leading to obesity and overweight in school children	Journal of Ayub Medical College, Abbottabad	Incorrect exposure
Janikian 2015	Adolescent gambling in seven European countries: Prevalence and related emotional and behavioural problems	Journal of Behavioural Addictions	Incorrect study type: conference proceeding or abstract
Jardine 2020	The Dark Web and cannabis use in the United States: Evidence from a big data research design	International Journal of Drug Policy	Incorrect exposure
Jaronko 2019	Leisure computer usage and perceived body weight, diet, and physical activity	Dissertation Abstracts International: Section B: The Sciences and Engineering	Incorrect study type: commentary/editorial/non-systematic review/theses/book chapter(s)
Jenkins 2019	Youth appeal in recreational marijuana promotions across three social media platforms	Journal of Adolescent Health	Incorrect study type: conference proceeding or abstract
Johnson 2014	Social media use and physical activity: Searching for opportunities to connect adolescents and older adults for health promotion	Journal of Adolescent Health	Incorrect study type: conference proceeding or abstract
Jones 2014	The impact of health education transmitted via social media or text messaging on adolescent and young adult risky sexual behavior: A systematic review of the literature	Sexually Transmitted Diseases	Incorrect study type: systematic review
Jones 2016	Association between young Australian's drinking behaviours and their interactions with alcohol brands on Facebook: results of an online survey	Alcohol and Alcoholism	Incorrect population
Jones Jayanetti 2018	Pizza, burgers, and booze: online marketing and promotion of food and drink to university students	Australian and New Zealand journal of public health	Incorrect population
Jonsson 2015	Online sexual behaviours among Swedish youth: associations to background factors, behaviours, and abuse	European Child and Adolescent Psychiatry	Incorrect exposure
Kairouz 2012	Are online gamblers more at risk than offline gamblers?	Cyberpsychology, Behaviour and Social Networking	Incorrect population

Author and year	Title	Publication source	Reason for exclusion
Kandola 2021	Prospective relationships of adolescents' screen-based sedentary behaviour with depressive symptoms: the Millennium Cohort Study	Psychological Medicine	No relevant outcome(s)
Kandola 2022	Impact on adolescent mental health of replacing screen-use with exercise: a prospective cohort study	Journal of Affective Disorders	No relevant outcome(s)
Kaplan 2012	Social networking and teen drug use: tremendous potential to help and potential to harm?	Psychiatric Times	Incorrect study type: commentary/editorial/non-systematic review/theses/book chapter(s)
Kazemi 2017	Systematic review of surveillance by social media platforms for illicit drug use	Journal of Public Health	Incorrect study type: systematic review
Keihner 2009	Psychosocial, socioeconomic, behavioural, and environmental risk factors for BMI and overweight among 9- to 11-year-old children	Californian Journal of Health Promotion	Incorrect exposure
Kemp 2020	'Social screens' and 'the mainstream': longitudinal competitors of non-organized physical activity in the transition from childhood to adolescence	The International Journal of Behavioural Nutrition and Physical Activity	Incorrect exposure
Kennewell 2022	The relationships between school children's wellbeing, socio-economic disadvantage, and after-school activities: a cross-sectional study	BMC Paediatrics	Incorrect exposure
Kerekes 2021	Changes in adolescents' psychosocial functioning and well-being as a consequence of long-term covid-19 restrictions	International Journal of Environmental Research and Public Health	No relevant outcome(s)
Kerr 2018	Associations between problem alcohol use and active and passive social media posts	Journal of Adolescent Health	Incorrect study type: conference proceeding or abstract
Ketchoo 2013	Smoking behaviour and associated factors of illicit cigarette consumption in a border province of southern Thailand	Tobacco Control	Incorrect exposure
Khajeheian 2018	Effect of social media on child obesity: Application of structural equation modelling with the Taguchi method	International Journal of Environmental Research and Public Health	No relevant outcome(s)
Khundadze 2017	Impact of internet gambling on mental and psychological health of children of various ages	Georgian Medical News	Incorrect exposure
Kim 2015	International note: Teen users' problematic online behaviour: Using panel data from South Korea	Journal of Adolescence	Incorrect exposure
Kim 2017	A path model of school violence perpetration: introducing online game addiction as a new risk factor	Journal of Interpersonal Violence	Incorrect exposure

Author and year	Title	Publication source	Reason for exclusion
Kim 2021	Parental mental health and children's behaviours and media usage during COVID-19-related school closures	Journal of Korean Medical Science	Incorrect exposure
King 2007	Surf and turf wars online--growing implications of Internet gang violence	Journal of Adolescent Health	Incorrect study type: commentary/editorial/non-systematic review/theses/book chapter(s)
Klainman 2015	Comparison of smoking habits between Jewish and Arabic youth in Israel	European Journal of Preventive Cardiology	Incorrect study type: conference proceeding or abstract
Kleppang 2021	Lifestyle habits and depressive symptoms in Norwegian adolescents: a national cross-sectional study	BMC Public Health	No relevant outcome(s)
Ko 2008	The association between Internet addiction and problematic alcohol use in adolescents: the problem behaviour model	Cyberpsychology & Behaviour: the impact of the Internet, multimedia and virtual reality on behaviour and society	Incorrect exposure
Kocturk 2018	A modern danger for adolescents: from online flirtation to sexual abuse.	Journal of Psychiatry & Neurological Sciences	Incorrect exposure
Korogoda 2016	Developmental neuroscience explaining why adolescents engage in risky behaviours	Journal of Psychosocial Nursing & Mental Health Services	Incorrect study type: commentary/editorial/non-systematic review/theses/book chapter(s)
Kranzler 2019	Youth social media use and health outcomes: #diggingdeeper	Journal of Adolescent Health	Incorrect study type: commentary/editorial/non-systematic review/theses/book chapter(s)
Krauss 2017	Marijuana advertising exposure among current marijuana users in the U.S	Drug And Alcohol Dependence	Incorrect exposure
Kristiansen 2022	Adolescent gambling advertising awareness: A national survey	International Journal of Social Welfare	Incorrect exposure
Kurten 2021	Mothers matter: using regression tree algorithms to predict adolescents' sharing of drunk references on social media	International Journal of Environmental Research and Public Health	No relevant outcome(s)
Kuss 2017	Social networking sites and addiction: Ten lessons learned	International Journal of Environmental Research and Public Health	Incorrect study type: commentary/editorial/non-systematic review/theses/book chapter(s)
Kwon 2020	Factors associated with adolescents' internet use duration by suicidal ideation	International Journal of Environmental Research and Public Health	Incorrect exposure
LaBrie 2021	An examination of the prospective associations between objectively assessed exposure to alcohol-related Instagram content, alcohol-specific cognitions, and first-year college drinking	Addictive Behaviours	Incorrect population

Author and year	Title	Publication source	Reason for exclusion
LaBrie 2021	Prospective relationships between objectively assessed social media use, drinking norms, and alcohol consumption among first-year students	Journal of Studies on Alcohol and Drugs	Incorrect population
Lampert 2007	Use of electronic media in adolescence: results of the German health interview and examination survey for children and adolescents	Bundesgesundheitsblatt - Gesundheitsforschung - Gesundheitsschutz	Incorrect exposure
Leatherdale 2010	Factors associated with communication-based sedentary behaviours among youth: Are talking on the phone, texting, and instant messaging new sedentary behaviours to be concerned about?	Journal of Adolescent Health	Incorrect exposure
Lee 2002	Internet and displacement effect: Children's media use and activities in Singapore	Journal of Computer-Mediated Communication	Incorrect exposure
Lee 2013	Substance abuse precedes internet addiction	Addictive Behaviours	Incorrect exposure
Lee 2015	Impact of the Internet use in the adolescence on the smoking and drinking in the early adult period: With the panel data	Journal of Behavioural Addictions	Incorrect study type: conference proceeding or abstract
Lee 2015	The association between online health information-seeking behaviours and health behaviours among Hispanics in New York city: a community-based cross-sectional study	Journal of Medical Internet Research	Incorrect population
Lee 2017	Longitudinal study shows that addictive Internet use during adolescence was associated with heavy drinking and smoking cigarettes in early adulthood	Acta Paediatric	Incorrect exposure
Lee 2019	Social networking addiction and depressive symptoms among adolescents in Korea	Journal of Behavioural Addictions	Incorrect study type: conference proceeding or abstract
Lee 2020	Youth and young adult use of pod-based electronic cigarettes from 2015 to 2019: a systematic review	JAMA Paediatrics	Incorrect study type: systematic review
Lees 2020	Screen media activity does not displace other recreational activities among 9–10-year-old youth: a cross-sectional ABCD study R	BMC Public Health	Incorrect population
Lehmkuhl 2013	The new media and their influence on children and adolescents	Kinder- und Jugendpsychiatrie und Psychotherapie	Incorrect study type: commentary/editorial/non-systematic review/theses/book chapter(s)
Lerman 2015	Using the internet to meet people and adolescent sexual risk	Journal of Adolescent Health	Incorrect study type: conference proceeding or abstract
Leung 2018	Exposure to electronic cigarette advertising and intention to use electronic cigarettes in Hong Kong adolescents	Tobacco Induced Diseases	Incorrect study type: conference proceeding or abstract

Author and year	Title	Publication source	Reason for exclusion
Leventhal 2018	New tobacco products with fewer advertising restrictions and consequences for the current generation of youths	JAMA Paediatrics	Incorrect study type: commentary/editorial/non-systematic review/theses/book chapter(s)
Lewycka 2018	Downwards trends in adolescent risk-taking behaviours in New Zealand: Exploring driving forces for change	Journal of Paediatrics and Child Health	Incorrect study type: commentary/editorial/non-systematic review/theses/book chapter(s)
Li 2017	Condom use peer norms and self-efficacy as mediators between community engagement and condom use among Chinese men who have sex with men	BMC Public Health	Incorrect exposure
Lipsky 2016	Behavioural and sociodemographic correlates of overall diet quality over 4 years in a national cohort of U.S. emerging adults	FASEB Journal	Incorrect study type: conference proceeding or abstract
Litt 2011	Adolescent alcohol use: The roles of social norms and social networking sites	Alcoholism: Clinical and Experimental Research	Incorrect study type: conference proceeding or abstract
Litt 2018	#drunktwitter: Examining the relations between alcohol-related Twitter content and alcohol willingness and use among underage young adults	Drug And Alcohol Dependence	Incorrect population
Litt 2019	A longitudinal randomized experimental study examining the impact of social networking site abstainer and drinker content on normative perceptions	Alcoholism: Clinical and Experimental Research	Incorrect study type: conference proceeding or abstract
Liu 2017	A pilot study of Pokémon go and players' physical activity	Games for Health Journal	Incorrect exposure
Liu 2021	The feasibility of using Instagram data to predict exercise identity and physical activity levels: cross-sectional observational study	Journal of Medical Internet Research	Incorrect population
Livingstone 2008	Taking risky opportunities in youthful content creation: Teenagers' use of social networking sites for intimacy, privacy, and self-expression	New Media & Society	Incorrect study type: qualitative
Livingstone 2015	What difference does 'the digital' make to children's experiences of risk?	International Journal of Public Health	Incorrect study type: commentary/editorial/non-systematic review/theses/book chapter(s)
Lizandra 2019	Screen time and moderate-to-vigorous physical activity changes and displacement in adolescence: A prospective cohort study	European Journal of Sport Science	Incorrect exposure
Long 2018	Online and health risk behaviours in high school students: an examination of bullying	Paediatric Nursing	Incorrect exposure
Lorenzo-Blanco 2021	E-cigarette use susceptibility among youth in Mexico: the roles of remote acculturation, parenting behaviours, and internet use frequency	Addictive Behaviours	Incorrect exposure

Author and year	Title	Publication source	Reason for exclusion
Lorimer 2016	Young men who have sex with men's use of social and sexual media and sex-risk associations: cross-sectional, online survey across four countries	Sexually Transmitted Infections	Incorrect population
Lou 2012	Media's contribution to sexual knowledge, attitudes, and behaviours for adolescents and young adults in Three Asian Cities	Journal of Adolescent Health	Incorrect exposure
Loukas 2019	Electronic nicotine delivery systems marketing and initiation among youth and young adults	Paediatrics	Incorrect exposure
Love 2019	How social media influences high school students to commit criminal offenses in South-eastern United States	Dissertation Abstracts International Section A: Humanities and Social Sciences	Incorrect study type: commentary/editorial/non-systematic review/theses/book chapter(s)
Lu 2018	Cross-sectional and temporal associations between cyber dating abuse victimization and mental health and substance use outcomes	Journal Of Adolescence	Incorrect exposure
Lukhele 2016	Multiple sexual partnerships and their correlates among Facebook users in Swaziland: an online cross-sectional study	African Journal of AIDS Research	Incorrect population
Luo 2018	Risk of HIV infection and its factors among men who have sex with men: a geosocial networking application-based survey in Beijing of China, 2017	Chinese Journal of Preventive Medicine	Incorrect population
Luo 2020	Comparison of HIV infection risk between 15 to 24 year-old student men who have sex with men and non-student men who have sex with men: a cross-sectional study	Chinese Journal of Preventive Medicine	Unable to source full text
Lwin 2017	Media exposure and parental mediation on fast-food consumption among children in metropolitan and suburban Indonesia	Asia Pacific Journal of Clinical Nutrition	Incorrect population
Lyons 2017	Masculinities, alcohol consumption and social networking	Youth Drinking Cultures in a Digital World	Incorrect study type: commentary/editorial/non-systematic review/theses/book chapter(s)
Lyvers 2020	Alexithymia, impulsivity, disordered social media use, mood, and alcohol use in relation to Facebook self-disclosure	Computers in Human Behaviour	Incorrect population
Maas 2019	Online sexual experiences predict subsequent sexual health and victimization outcomes among female adolescents: a latent class analysis	Journal of Youth and Adolescence	Incorrect exposure
Macapagal 2018	Hookup app use, sexual behaviour, and sexual health among adolescent men who have sex with men in the United States	Journal of Adolescent Health	Incorrect exposure
Macapagal 2019	Geosocial networking application use, characteristics of app-met sexual partners, and sexual behaviour among sexual and gender minority adolescents assigned male at birth	Journal of Sex Research	Incorrect exposure

Author and year	Title	Publication source	Reason for exclusion
MacMillan 2021	Exploring factors associated with alcohol and/or substance use during the covid-19 pandemic	International Journal of Mental Health and Addiction	Incorrect population
Mahase 2019	Social media can harm when use displaces sleep or exercise or involves bullying, finds study	BMJ	Incorrect study type: commentary/editorial/non-systematic review/theses/book chapter(s)
Malheiros 2021	Association between physical activity, screen time activities, diet patterns and daytime sleepiness in a sample of Brazilian adolescents	Sleep Medicine	No relevant outcome(s)
Marker 2019	Exploring the myth of the chubby gamer: A meta-analysis on sedentary video gaming and body mass	Social Science and Medicine	Incorrect study type: systematic review
Marks 2015	Friendship network characteristics are associated with physical activity and sedentary behaviour in early adolescence	Plos One	Incorrect exposure
Marotta 2018	Impact and risks of new information technologies in adolescents: Results of a survey conducted on 1534 subjects	Giornale di Neuropsichiatria dell'Età Evolutiva	Potentially relevant non-English language
Marques 2018	Facebook: risks and opportunities in Brazilian and Portuguese youths with different levels of psychosocial adjustment	The Spanish Journal of Psychology	No relevant outcome(s)
Masitah 2019	Social media and adolescent macro nutrition intake	Annals of Nutrition and Metabolism	Incorrect study type: conference proceeding or abstract
Mata 2011	Obesity in children and adolescents: Risks, causes, and therapy from a psychological perspective	Bundesgesundheitsblatt - Gesundheitsforschung - Gesundheitsschutz	Incorrect study type: commentary/editorial/non-systematic review/theses/book chapter(s)
Mayhew 2017	Youth and sexually explicit internet material: Separating truth from fiction	Journal of the American Academy of Child and Adolescent Psychiatry	Incorrect study type: conference proceeding or abstract
McBride 2011	Risks and benefits of social media for children and adolescents	Journal of Paediatric Nursing	Incorrect study type: commentary/editorial/non-systematic review/theses/book chapter(s)
McCarthy 2022	The influence of unhealthy food and beverage marketing through social media and advergaming on diet-related outcomes in children-a systematic review	Obesity Reviews	Incorrect study type: systematic review
McClure 2013	TV and internet alcohol marketing and underage alcohol use	Alcoholism: Clinical and Experimental Research	Incorrect study type: conference proceeding or abstract
McClure 2013	Alcohol marketing receptivity, marketing-specific cognitions, and underage binge drinking	Alcoholism: Clinical and Experimental Research	Incorrect exposure

Author and year	Title	Publication source	Reason for exclusion
McClure 2016	Internet alcohol marketing and underage alcohol use	Paediatrics	Incorrect exposure
McCreanor 2013	Youth drinking cultures, social networking, and alcohol marketing: Implications for public health	Critical Public Health	Incorrect study type: commentary/editorial/non-systematic review/theses/book chapter(s)
McFarlane 2002	Young adults on the Internet: risk behaviours for sexually transmitted diseases and HIV	Journal of Adolescent Health	Incorrect exposure
Meeus 2018	Managing positive and negative media effects among adolescents: parental mediation matters but not always	Journal of Family Communication	Incorrect exposure
Melkevik 2010	Is spending time in screen-based sedentary behaviours associated with less physical activity: A cross national investigation	International Journal of Behavioural Nutrition and Physical Activity	Incorrect exposure
Merkel 2018	Social media use and physical activity: To share or not to share?	Journal of Physical Education, Recreation & Dance	Incorrect study type: commentary/editorial/non-systematic review/theses/book chapter(s)
Miller 2021	Online peers and offline highs: an examination of online peer groups, social media homophily, and substance use	Journal of Psychoactive Drugs	Incorrect population
Mishu 2021	Predictors of cigarette smoking, smokeless tobacco consumption, and use of both forms in adolescents in South Asia: a secondary analysis of the Global Youth Tobacco Surveys	Nicotine and Tobacco Research	Incorrect exposure
Mitchell 2007	Youth internet users at risk for the most serious online sexual solicitations	American Journal of Preventive Medicine	No relevant outcome(s)
Mitchell 2014	Rural Environments and Community Health (REACH): a randomised controlled trial protocol for an online walking intervention in rural adults	BMC Public Health	Incorrect study type: commentary/editorial/non-systematic review/theses/book chapter(s)
Mitchell 2019	Physical inactivity in childhood from preschool to adolescence	ACSM's Health & Fitness Journal	Incorrect study type: commentary/editorial/non-systematic review/theses/book chapter(s)
Moitra 2021	Screen time is associated with eating habits, sleep patterns, and adiposity measures in adolescents	Obesity Facts	Incorrect study type: conference proceeding or abstract
Moreno 2012	Social networking sites and adolescent health	Paediatric Clinics of North America	Incorrect study type: commentary/editorial/non-systematic review/theses/book chapter(s)
Moreno 2014	Influence of social media on alcohol use in adolescents and young adults	Alcohol Research: Current Reviews	Incorrect study type: commentary/editorial/non-systematic review/theses/book chapter(s)

Author and year	Title	Publication source	Reason for exclusion
Moreno 2016	#Wasted: The intersection of substance use behaviours and social media in adolescents and young adults	Current Opinion in Psychology	Incorrect study type: commentary/editorial/non-systematic review/theses/book chapter(s)
Moreno 2019	Testing young adults' reactions to Facebook cues and their associations with alcohol use	Substance Use & Misuse	Incorrect exposure
Morioka 2016	Association between smoking and problematic internet use among Japanese adolescents: large-scale nationwide epidemiological study	Cyberpsychology, Behaviour and Social Networking	Incorrect exposure
Morioka 2017	The association between alcohol use and problematic internet use: A large-scale nationwide cross-sectional study of adolescents in Japan	Journal of Epidemiology	Incorrect exposure
Mu 2015	Internet use and adolescent binge drinking: Findings from the monitoring the future study	Addictive Behaviours Reports	Incorrect exposure
Mucci 2016	Prevalence of internet addiction: A pilot study in a group of Italian students	European Neuropsychopharmacology	Incorrect study type: conference proceeding or abstract
Mukadi 2018	Sexual behaviour of the school-going youth in the city of Likasi, democratic Republic of Congo	Pan African Medical Journal	Incorrect exposure
Munoz-Miralles 2016	The problematic use of Information and Communication Technologies (ICT) in adolescents by the cross sectional JOITIC study	BMC Paediatrics	Incorrect exposure
Murray 2015	A survey of the practices and perceptions of students in one catholic high school on the use of the internet regarding safety, cyberbullying, and sexting	Dissertation Abstracts International Section A: Humanities and Social Sciences	Incorrect study type: commentary/editorial/non-systematic review/theses/book chapter(s)
Namkoong 2017	Communication, reasoning, and planned behaviours: unveiling the effect of interactive communication in an anti-smoking social media campaign	Health Communication	Incorrect exposure
Nawi 2021	Risk and protective factors of drug abuse among adolescents: a systematic review	BMC Public Health	Incorrect study type: systematic review
Negriff 2018	Structural characteristics of the online social networks of maltreated youth and offline sexual risk behaviour	Child Abuse and Neglect	Incorrect comparator group
Negriff 2019	The influence of online-only friends on the substance use of young adults with a history of childhood maltreatment	Substance Use & Misuse	Incorrect exposure
Nelson 2019	Sexually explicit media use among 14-17-year-old sexual minority males in the U.S	Archives of Sexual Behaviour	Incorrect exposure

Author and year	Title	Publication source	Reason for exclusion
Ng Fat 2021	Associations between social media usage and alcohol use among youths and young adults: findings from Understanding Society	Addiction	Exact duplicate
No Authors listed 2003	Adolescents advertising and tobacco smoking	Medicine Today	Incorrect study type: commentary/editorial/non-systematic review/theses/book chapter(s)
No Authors listed 2010	The "excess" generation	Rivista Italiana di Medicina dell'Adolescenza	Unable to source full text
No authors listed 2015	Social media to combat youth drinking	Australian Nursing & Midwifery Journal	Incorrect study type: commentary/editorial/non-systematic review/theses/book chapter(s)
No authors listed 2016	Social media as a new venue for aggression and bullying	Adolescent Medicine: State of the Art Reviews	Incorrect study type: commentary/editorial/non-systematic review/theses/book chapter(s)
No authors listed 2018	The role of internet addiction on fatigue, sleep disturbances and poor life-style habits among adolescents	European Psychiatry	Incorrect study type: conference proceeding or abstract
Noel 2020	Exposure to digital alcohol marketing and alcohol use: a systematic review	Journal of Studies on Alcohol and Drugs	Incorrect study type: systematic review
Noll 2013	Association of maltreatment with high-risk internet behaviours and offline encounters	Paediatrics	No relevant outcome(s)
Norris Turner 2011	Social media and chlamydia testing by university students: A pilot study	Sexually Transmitted Infections	Incorrect study type: conference proceeding or abstract
Nunez-Smith 2010	Media exposure and tobacco, illicit drugs, and alcohol use among children and adolescents: a systematic review	Substance Abuse	Incorrect study type: systematic review
O'Brien 2021	Relationship between gender, physical activity, screen time, body mass index and wellbeing in Irish children from social disadvantage	Child Care in Practice	Incorrect exposure
O'Cathail 2011	Association of cigarette smoking with drug use and risk taking behaviour in Irish teenagers	Addictive Behaviours	Incorrect exposure
Ogunleye 2012	Prevalence of high screen time in English youth: association with deprivation and physical activity	Journal of Public Health	Incorrect exposure
Ojanen 2014	Investigating online harassment and offline violence among young people in Thailand: methodological approaches, lessons learned	Culture, Health & Sexuality	Incorrect study type: qualitative

Author and year	Title	Publication source	Reason for exclusion
O'Keeffe 2011	Clinical report - The impact of social media on children, adolescents, and families	Paediatrics	Incorrect study type: commentary/editorial/non-systematic review/theses/book chapter(s)
O'Keeffe 2011	The impact of social media on children, adolescents, and families	Paediatrics	Exact duplicate
Oksanen 2021	Social media and access to drugs online: A nationwide study in the United States and Spain among adolescents and young adults	The European Journal of Psychology Applied to Legal Context	Incorrect population
Olafsdottir 2014	Young children's screen activities, sweet drink consumption and anthropometry: Results from a prospective European study	European Journal of Clinical Nutrition	Incorrect exposure
Olaleye 2017	Social-media use and sexual behaviour among in-school adolescents in Ibadan, Nigeria	Sexually Transmitted Infections	Incorrect study type: conference proceeding or abstract
O'Sullivan 2012	Texts from last night: screen time, porn use, sexting, and chat as predictors of sexual intercourse experience among Canadian adolescents	Journal of Adolescent Health	Incorrect study type: conference proceeding or abstract
Ouellette 2019	YouTube and risky behaviours in adolescents: The "choking game"	The American Journal of Emergency Medicine	Incorrect study type: commentary/editorial/non-systematic review/theses/book chapter(s)
Pahn 2019	Impact of short message service (SMS) and social media on sexual intercourse of high school students in Cambodia	Journal of Korean Academy of Community Health Nursing	No relevant outcome(s)
Palamar 2020	Posting, texting, and related social risk behaviour while high	Substance Abuse	Incorrect exposure
Palasinski 2013	Can computer-mediated communication increase adolescents' sexually risky behaviours?	The American Psychologist	Incorrect study type: commentary/editorial/non-systematic review/theses/book chapter(s)
Palkar 2019	Digitizing interventions: An internet-based approach to reach out to the "hidden network of men who have sex with men" in Mumbai, India	Journal of the International AIDS Society	Incorrect study type: conference proceeding or abstract
Park 2011	The relation between screen time and health behaviours in Korean children	Obesity	Incorrect study type: conference proceeding or abstract
Park 2013	A systematic review of social networking sites: Innovative platforms for health research targeting adolescents and young adults	Journal of Nursing Scholarship	Incorrect study type: systematic review
Parker 2021	The use of digital platforms for adults' and adolescents' physical activity during the COVID-19 pandemic (our life at home): Survey study	Journal of Medical Internet Research	Incorrect exposure

Author and year	Title	Publication source	Reason for exclusion
Parkes 2013	Are sexual media exposure, parental restrictions on media use and co-viewing TV and DVDs with parents and friends associated with teenagers' early sexual behaviour?	Journal of Adolescence	Incorrect exposure
Patel 2013	Social media use and HIV risk behaviours in young men who have sex with men of colour in New York city: Implications for outreach and prevention	Journal of General Internal Medicine	Incorrect study type: conference proceeding or abstract
Patel 2016	Social media use and HIV related risk behaviours in young black and Latino gay and bi men and transgender individuals in New York city: implications for online interventions	Journal of Urban Health: bulletin of the New York Academy of Medicine	Incorrect population
Patrick 2015	Demographic and behavioural correlates of six sexting behaviours among Australian secondary school students	Sexual Health	Incorrect exposure
Patton 2014	Social media as a vector for youth violence: A review of the literature	Computers in Human Behaviour	Incorrect study type: commentary/editorial/non-systematic review/theses/book chapter(s)
Paulos 2010	DID video kill the radio star? - Assessing gambling and multimedia use in Luxembourg's high school students	European Psychiatry	Incorrect study type: conference proceeding or abstract
Pauwels 2016	Differential online exposure to extremist content and political violence: Testing the relative strength of social learning and competing perspectives	Terrorism and Political Violence	Incorrect population
Pedersen 2004	Mobile phones, web chat, and sex among Norwegian adolescents	Tidsskr Nor Laegeforen	Potentially relevant non-English language
Pedersen 2004	Mobile phones, web chat, and sex: A study of Norwegian adolescents based on a representative sample	Tidsskrift for den Norske Laegeforening	Exact duplicate
Peek 2014	The selfie in the digital age: from social media to sexting	Psychiatric Times	Incorrect study type: commentary/editorial/non-systematic review/theses/book chapter(s)
Peiper 2020	Differential patterns of e-cigarette and tobacco marketing exposures among youth: Associations with substance use and tobacco prevention strategies	International Journal of Drug Policy	Incorrect exposure
Peter 2011	The influence of sexually explicit internet material on sexual risk behaviour: a comparison of adolescents and adults	Journal of Health Communication	Incorrect exposure
Piguet 2015	What keeps female problematic Internet users busy online?	European Journal of Paediatrics	Incorrect exposure
Pocs 2019	Tobacco reduction on Facebook among 14-35-year-olds	Orv Hetil	Incorrect population

Author and year	Title	Publication source	Reason for exclusion
Pokhrel 2021	Exposure to e-cigarette content on social media and e-cigarette use: An ecological momentary assessment study	Addictive Behaviours Reports	Incorrect population
Post 2021	SARS-CoV-2 wave two surveillance in East Asia and the Pacific: longitudinal trend analysis	Journal of Medical Internet Research	Incorrect exposure
Potenza 2011	Correlates of at-risk/problem internet gambling in adolescents	Journal of the American Academy of Child & Adolescent Psychiatry	Incorrect exposure
Prot 2014	Long-term relations among prosocial-media use, empathy, and prosocial behaviour	Psychological Science	Incorrect exposure
Przybylski 2018	Internet filtering and adolescent exposure to online sexual material	Cyberpsychology, Behaviour and Social Networking	Incorrect exposure
Pujazon-Zazik 2010	Adolescents' self-presentation on a teen dating website: A risk content analysis	Journal of Adolescent Health	Incorrect study type: conference proceeding or abstract
Pujazon-Zazik 2010	To tweet, or not to tweet: gender differences and potential positive and negative health outcomes of adolescents' social internet use	American Journal of Men's Health	Incorrect study type: commentary/editorial/non-systematic review/theses/book chapter(s)
Queiroz 2019	Sexually transmitted infections and factors associated with condom use in dating app users in Brazil	Acta Paulista de Enfermagem	Incorrect population
Ra 2018	Association of digital media use with subsequent symptoms of attention-deficit/hyperactivity disorder among adolescents	Journal of the American Medical Association	No relevant outcome(s)
Radanielina Hita 2018	Parental mediation in the digital era: increasing children's critical thinking may help decrease positive attitudes toward alcohol	Journal of Health Communication	Incorrect population
Ragelienė 2021	The role of peers, siblings and social media for children’s healthy eating socialization: A mixed methods study	Food Quality and Preference	No relevant outcome(s)
Raggatt 2019	Correlates of reduced alcohol consumption among a sample of young Australians	Alcohol and Alcoholism	Incorrect population
Rankine 2016	The association between online risk behaviours and real life sexual behaviours among African American female adolescents	Dissertation Abstracts International: Section B: The Sciences and Engineering	Incorrect study type: commentary/editorial/non-systematic review/theses/book chapter(s)
Reid 2014	Social media use among adolescents: benefits and risks	Adolescent Psychiatry	Exact duplicate
Reid 2014	Social media use among adolescents: Benefits and risks	Adolescent Psychiatry	Incorrect study type: commentary/editorial/non-systematic review/theses/book chapter(s)

Author and year	Title	Publication source	Reason for exclusion
Rial 2018	Minors and problematic internet use: Evidence for better prevention	Computers in Human Behaviour	Incorrect exposure
Ribisl 2003	The potential of the internet as a medium to encourage and discourage youth tobacco use	Tobacco Control	Incorrect study type: commentary/editorial/non-systematic review/theses/book chapter(s)
Rice 2010	Internet use, social networking, and HIV/AIDS risk for homeless adolescents	Journal of Adolescent Health	Incorrect population
Rice 2016	Social media and digital technology use among Indigenous young people in Australia: A literature review	International Journal for Equity in Health	Incorrect study type: systematic review
Richards 2015	Impact of social media on the health of children and young people	Journal of Paediatrics and Child Health	Incorrect study type: systematic review
Richter 2020	The good, the bad and the ugly: the relationship between social media use, subjective health and risk behaviour among children and adolescents	Gesundheitswesen	Potentially relevant non-English language
Richter 2021	The good, the bad and the ugly: the relationship between social media use, subjective health and risk behaviour among children and adolescents	Gesundheitswesen	Exact duplicate
Ricketts 2015	The effect of Internet related problems on the sexting behaviours of juveniles	American Journal of Criminal Justice	Incorrect exposure
Rideout 2002	Generation Rx.com What are young people really doing online?	Marketing Health Services	Incorrect study type: commentary/editorial/non-systematic review/theses/book chapter(s)
Rodenhizer 2019	The impacts of sexual media exposure on adolescent and emerging adults' dating and sexual violence attitudes and behaviours: a critical review of the literature	Trauma, Violence & Abuse	Incorrect study type: systematic review
Rodgers 2020	A biopsychosocial model of social media use and body image concerns, disordered eating, and muscle-building behaviours among adolescent girls and boys	Journal of Youth and Adolescence	No relevant outcome(s)
Rodopman Arman 2015	Defining social reciprocity deficits in internet addiction: Evaluation of problematic internet user (PIU) adolescents in an university outpatient clinic	European Child and Adolescent Psychiatry	Incorrect study type: conference proceeding or abstract
Romer 2017	Digital media and risks for adolescent substance abuse and problematic gambling	Paediatrics	Incorrect study type: commentary/editorial/non-systematic review/theses/book chapter(s)
Romo 2016	Associations between frequent social media and sexting with sexual risk behaviours in Uganda adolescents	Sexually Transmitted Diseases	Incorrect study type: conference proceeding or abstract

Author and year	Title	Publication source	Reason for exclusion
Romo 2016	Understanding adolescent social media use: Association with sexual risk and parental monitoring factors that can influence protection	Journal of Adolescent Health	Incorrect study type: conference proceeding or abstract
Rosen 2014	Media and technology use predicts ill-being among children, preteens, and teenagers independent of the negative health impacts of exercise and eating habits	Computers in Human Behaviour	Incorrect exposure
Rosengren 2020	Online sex partner seeking and HIV testing frequency among young black sexual minority men	Journal of HIV/AIDS & Social Services	Incorrect population
Rosser 2013	The effects of gay sexually explicit media on the HIV risk behaviour of men who have sex with men	AIDS and Behaviour	Incorrect exposure
Rounsefell 2020	Social media, body image and food choices in healthy young adults: A mixed methods systematic review	Nutrition & Dietetics	Incorrect study type: systematic review
Rucker 2015	Problematic Internet use is associated with substance use in young adolescents	Acta Paediatrica	Incorrect exposure
Russell 2022	Social networking site use and alcohol use behaviors among adolescents: A latent profile analysis	Addictive Behaviors	Incorrect population
Ryu 2022	Smartphone Usage Patterns and Dietary Risk Factors in Adolescents	The Journal of nutrition	Duplicate sample
Sabramani 2021	Bullying and Its Associated Individual, Peer, Family and School Factors: Evidence from Malaysian National Secondary School Students	International journal of environmental research and public health	No relevant outcome(s)
Sampasa-Kanyinga 2020	Sex differences in the relationship between social media use, short sleep duration, and body mass index among adolescents	Sleep Health	No relevant outcome(s)
Sande 2021	Alcohol-related risks for slovene secondary school students on graduation trips: ten years later	Archives of Psychiatry Research	Incorrect exposure
Sano 2020	Relationship between prolonged media usage and lifestyle habits among junior and senior high school students	Japanese Journal of Public Health	Incorrect exposure
Santistevan 2017	Awareness of e-cigarettes and correlation of use among high school students	Dissertation Abstracts International Section A: Humanities and Social Sciences	Incorrect study type: commentary/editorial/non-systematic review/theses/book chapter(s)
Sarchiapone 2013	The use of internet in prevention	European Psychiatry	Incorrect study type: conference proceeding or abstract

Author and year	Title	Publication source	Reason for exclusion
Saunders 2016	A snapshot of the sexual experiences of bisexual black adolescent males over 1 year	Journal of Adolescent Health	Incorrect study type: conference proceeding or abstract
Savoia 2021	Adolescents' exposure to online risks: gender disparities and vulnerabilities related to online behaviours	International Journal of Environmental Research and Public Health	No relevant outcome(s)
Savolainen 2020	Online relationships and social media interaction in youth problem gambling: a four-country study	International Journal of Environmental Research and Public	Incorrect exposure
Savolainen 2021	The role of online group norms and social identity in youth problem gambling	Computers in Human Behaviour	Incorrect population
Schafer 2022	Stigma, social support, and substance use in diverse men who have sex with men and transgender women living with HIV in the US Southeast	Southern Medical Journal	Incorrect population
Scott 2016	The social influence of friends' alcohol-related content posted on social media	Alcoholism: Clinical and Experimental Research	Incorrect study type: conference proceeding or abstract
Seidenberg 2017	A national study of social media, television, radio, and internet usage of adults by sexual orientation and smoking status: implications for campaign design	International Journal of Environmental Research and Public Health	Incorrect population
Sela-Shayovitz 2012	Gangs and the web: Gang members online behavior	Journal of Contemporary Criminal Justice	Incorrect study type: qualitative
Self-Brown 2021	Individual and parental risk factors for sexual exploitation among high-risk youth in Uganda	Journal of Interpersonal Violence	Exact duplicate
Sevcikova 2013	Predictors of online and offline sexual activities and behaviours among adolescents	Cyberpsychology, Behaviour and Social Networking	Incorrect exposure
Ševčíková 2016	Girls' and boys' experience with teen sexting in early and late adolescence	Journal of Adolescence	Incorrect exposure
Sevic 2020	The relationship between the use of social networking sites and sexually explicit material, the internalization of appearance ideals and body self-surveillance: results from a longitudinal study of male adolescents	Journal of Youth and Adolescence	No relevant outcome(s)
Shamu 2020	Knowledge, attitudes, and practices of young adults towards HIV prevention: an analysis of baseline data from a community-based HIV prevention intervention study in two high HIV burden districts, South Africa	BMC Public Health	Incorrect population
Shapiro 2017	Correlates of tinder use and risky sexual behaviours in young adults	Cyberpsychology, Behaviour and Social Networking	Incorrect population

Author and year	Title	Publication source	Reason for exclusion
She 2022	Profiles of stress and coping associated with mental, behavioural, and internet use problems among adolescents during the COVID-19 pandemic: a stratified random sampling and cluster analysis	Frontiers in Public Health	Incorrect exposure
Shi 2011	Weekend television viewing and video gaming are associated with less adolescent smoking	Journal of Substance Use	Incorrect exposure
Shuai 2021	Influences of digital media use on children and adolescents with ADHD during COVID-19 pandemic	Globalization and Health	No relevant outcome(s)
Shukla 2019	Sugar-sweetened beverages and screen time: partners in crime for adolescent obesity	Journal of Paediatrics	Incorrect study type: commentary/editorial/non-systematic review/theses/book chapter(s)
Silva 2016	Type and quantity of physical activity and screen based activities of students from the 7th to the 12th grades: Characterization and association	Revista Portuguesa de Saude Publica	Incorrect exposure
Silva 2018	Prevalence of overweight and obesity and associated factors in school children and adolescents in a medium-sized Brazilian city	Clinics	Incorrect exposure
Simon 2018	Socioeconomic status and adolescent e-cigarette use: The mediating role of e-cigarette advertisement exposure	Preventive Medicine	Incorrect exposure
Sina 2022	Social media and children's and adolescents' diets - a systematic review of the underlying social and physiological mechanisms	Advances in Nutrition	Incorrect study type: systematic review
Smith 2016	Is sexual content in new media linked to sexual risk behaviour in young people? A systematic review and meta-analysis	Sexual Health	Incorrect study type: systematic review
Sobowale 2017	Understanding the role of reward processing and depression in compulsive internet use among V adolescents	Journal of the American Academy of Child and Adolescent Psychiatry	Incorrect study type: conference proceeding or abstract
Soneji 2018	Engagement with online tobacco marketing and associations with tobacco product use among US youth: findings from Wave 1 of the Population Assessment of Tobacco and Health Study	Journal of Adolescent Health	Duplicate sample
Soneji 2019	Online tobacco marketing among US adolescent sexual, gender, racial, and ethnic minorities	Addictive Behaviours	No relevant outcome(s)
Spilkova 2017	Predictors of excessive use of social media and excessive online gaming in Czech teenagers	Journal of Behavioural Addictions	Incorrect exposure
Stevens 2022	On sex, drugs, and alcohol: A mixed-method analysis of youth posts on social media in the united states	Journal of Children and Media	Incorrect exposure

Author and year	Title	Publication source	Reason for exclusion
Stiglic 2019	Effects of screentime on the health and well-being of children and adolescents: a systematic review of reviews	BMJ open	Incorrect study type: systematic review
Stoddard 2012	Permissive norms and young adults' alcohol and marijuana use: the role of online communities	Journal of Studies on Alcohol and Drugs	Incorrect population
Strizek 2020	Perceived problems with adolescent online gaming: national differences and correlations with substance use	Journal of Behavioural Addictions	Incorrect exposure
Stulhofer 2005	Internet and sexual compulsivity	Socijalna Psihijatrija	Potentially relevant non-English language
Sun 2005	Internet accessibility and usage among urban adolescents in Southern California: Implications for web-based health research	Cyberpsychology and Behaviour	Incorrect exposure
Suris 2014	Problematic internet use and substance use in adolescence	Journal of Adolescent Health	Incorrect study type: conference proceeding or abstract
Suwarni 2019	Determinants of the pornography exposure effects on Junior and Senior High School Adolescence in Sanggau District, West Kalimantan	Indian Journal of Public Health Research and Development	Incorrect exposure
Tadena 2020	The influence of social media affinity on eating attitudes and body dissatisfaction in Philippine adolescents	Child Health Nursing Research	No relevant outcome(s)
Tahir 2020	Does watching violent electronic and social media content lead to increased levels of aggression? A survey among adolescents in an urban slum of metropolitan Karachi	International Journal of Adolescent Medicine and Health	Incorrect exposure
Teunissen 2016	Friends' drinking norms and male adolescents' alcohol consumption: The moderating role of performance-based peer influence susceptibility	Journal of Adolescence	Incorrect exposure
Thammasarn 2020	Effects of food fit for fun program with social media used on health literacy and obesity prevention behaviours among senior-primary school students, in Nakhon Ratchasima Province Thailand	Indian Journal of Public Health Research and Development	Incorrect exposure
Thompson 2005	Addicted media: Substances on screen	Child and Adolescent Psychiatric Clinics of North America	Incorrect study type: commentary/editorial/non-systematic review/theses/book chapter(s)
Thonglua 2015	The association between internet use and sexual attitudes and behaviours of the secondary school students in Bangkok	Journal of Sexual Medicine	No relevant outcome(s)
Thrasher 2016	Prevalence and correlates of e-cigarette perceptions and trial among early adolescents in Mexico	Journal of Adolescent Health	Incorrect exposure

Author and year	Title	Publication source	Reason for exclusion
Tomic 2018	Associations between Croatian adolescents' use of sexually explicit material and sexual behavior: does parental monitoring play a role?	Archives of Sexual Behavior	Incorrect exposure
Törrönen 2020	How do social media-related attachments and assemblages encourage or reduce drinking among young people?	Journal of Youth Studies	Incorrect study type: qualitative
Trangenstein 2021	Cannabis Marketing and Problematic Cannabis Use Among Adolescents	Journal of studies on alcohol and drugs	Duplicate sample
Trangenstein 2022	Typology of Adolescents Exposed to Non-medical Cannabis Marketing and Associations with Consumption Patterns	Prevention Science	Duplicate sample
Tucker 2013	Cross-lagged associations between substance use-related media exposure and alcohol use during middle school	Alcoholism: Clinical and Experimental Research	Incorrect study type: conference proceeding or abstract
Tucker 2013	Cross-lagged associations between substance use-related media exposure and alcohol use during middle school	Journal of Adolescent Health	Incorrect exposure
Twenge 2022	Specification curve analysis shows that social media use is linked to poor mental health, especially among girls	Acta Psychologica	No relevant outcome(s)
Uhls 2017	Benefits and costs of social media in adolescence	Pediatrics	Incorrect study type: commentary/editorial/non-systematic review/theses/book chapter(s)
Unger 2018	Talking about tobacco on Twitter is associated with tobacco product use	Preventive Medicine	Incorrect population
van der Sanden 2021	Predictors of using social media to purchase drugs in New Zealand: Findings from a large-scale online survey	International Journal of Drug Policy	Incorrect population
Van Hulst 2020	Determinants of new onset cardiometabolic risk among normal weight children	International Journal of Obesity	Incorrect exposure
van Oosten 2015	Exploring associations between exposure to sexy online self-presentations and adolescents' sexual attitudes and behaviour	Journal of Youth and Adolescence	No relevant outcome(s)
van Oosten 2017	Sexy online self-presentation on social network sites and the willingness to engage in sexting: A comparison of gender and age	Journal of Adolescence	No relevant outcome(s)
van Oosten 2018	The importance of adolescents' sexually outgoing self-concept: differential roles of self- and other-generated sexy self-presentations in social media	Cyberpsychology, Behaviour and Social Networking	No relevant outcome(s)

Author and year	Title	Publication source	Reason for exclusion
Van Ouytsel 2016	Cyber dating abuse: Research on young people's motives and the associations of the behaviour in Flanders, Belgium	Journal of the American Academy of Child and Adolescent Psychiatry	Incorrect study type: conference proceeding or abstract
Van Ouytsel 2019	An exploratory study of sexting behaviours among heterosexual and sexual minority early adolescents	Journal of Adolescent Health	Incorrect exposure
Vandenbosch 2018	Explaining the relationship between sexually explicit internet material and casual sex: a two-step mediation model	Archives of Sexual Behavior	Incorrect exposure
Vander Wyst 2019	A social media intervention to improve nutrition knowledge and behaviours of low income, pregnant adolescents, and adult women	Plos One	Incorrect exposure
Vannucci 2020	Social media use and risky behaviours in adolescents: A meta-analysis	Journal of Adolescence	Incorrect study type: systematic review
Vaterlaus 2015	#Gettinghealthy: The perceived influence of social media on young adult health behaviours.	Computers in Human Behaviour	Incorrect study type: qualitative
Vente 2017	Social media as a vehicle for expression of self-harm and risk-taking behaviour in adolescents	Journal of the American Academy of Child and Adolescent Psychiatry	Incorrect study type: conference proceeding or abstract
Vente 2018	Evaluating high-risk behaviours in adolescents on social media	Journal of Adolescent Health	Incorrect study type: conference proceeding or abstract
Villani 2001	Impact of media on children and adolescents: A 10-year review of the research	Journal of the American Academy of Child and Adolescent Psychiatry	Incorrect study type: commentary/editorial/non-systematic review/theses/book chapter(s)
Viner 2019	Roles of cyberbullying, sleep, and physical activity in mediating the effects of social media use on mental health and wellbeing among young people in England: a secondary analysis of longitudinal data	The Lancet Child and Adolescent Health	No relevant outcome(s)
Viner 2020	Correction to Lancet Child Adolescent Health 2019: Roles of cyberbullying, sleep, and physical activity in mediating the effects of social media use on mental health and wellbeing among young people in England: a secondary analysis of longitudinal data	The Lancet Child and Adolescent Health	Incorrect study type: commentary/editorial/non-systematic review/theses/book chapter(s)
Vogel 2020	Effects of social media on adolescents' willingness and intention to use e-cigarettes: an experimental investigation	Journal of the Society for Research on Nicotine and Tobacco	No relevant outcome(s)
Wahyuni 2020	Determinants of adolescent's high-risk sexual behaviour in SMK 8 and MegaRezky Health Vocational School Makassar	Enfermería Clínica	Unable to source full text
Wahyurin 2019	Physical activity, screen time, and nutritional status in adolescents in Banyumas	Annals of Tropical Medicine and Public Health	Incorrect exposure

Author and year	Title	Publication source	Reason for exclusion
Walther 2014	Nutrition, lifestyle factors, and mental health in adolescents and young adults living in Austria.	International Journal of Adolescent Medicine And Health	Incorrect exposure
Wang 2012	Adolescent bullying involvement and psychosocial aspects of family and school life: A cross-sectional study from Guangdong province in China	Plos One	Incorrect exposure
Watchirs Smith 2013	Do new media affect adolescent sexual attitudes and behaviours? A systematic review	Sexually Transmitted Infections	Incorrect study type: conference proceeding or abstract
Welsh 2013	The sugar-sweetened beverage wars: public health and the role of the beverage industry	Current Opinion in Endocrinology, Diabetes, and Obesity	Incorrect study type: commentary/editorial/non-systematic review/theses/book chapter(s)
Werneck 2018	Social, behavioural, and biological correlates of cardiorespiratory fitness according to sex, nutritional status, and maturity status among adolescents. A cross-sectional study	Sao Paulo Medical Journal	Incorrect exposure
Westgate 2014	"I will take a shot for every 'like' I get on this status": posting alcohol-related Facebook content is linked to drinking outcomes	Journal of Studies on Alcohol and Drugs	Incorrect population
White 2015	Adolescents' and young adults' online risk taking: the role of gist and verbatim representations	Risk Analysis	Incorrect exposure
Whitehill 2015	Emerging adults' use of alcohol and social networking sites during a large street festival: A real-time interview study	Substance Abuse Treatment, Prevention, and Policy	Incorrect population
Whitehill 2020	Exposure to cannabis marketing in social and traditional media and past-year use among adolescents in states with legal retail cannabis	Journal of Adolescent Health	Exact duplicate
Whiteley 2011	African American adolescents and new media: Associations with HIV/STI risk behaviour and psychosocial variables	Ethnicity and Disease	Incorrect exposure
Wickel 2013	Variables associated with active and inactive behaviour during the after-school period	Paediatric Exercise Science	Incorrect exposure
Willoughby 2022	Social media, marijuana, and sex: an exploratory study of adolescents' intentions to use and college students' use of marijuana	Journal of Sex Research	Incorrect population
Winetrobe 2014	Associations of unprotected anal intercourse with Grindr-met partners among Grindr-using young men who have sex with men in Los Angeles	AIDS Care	Incorrect population
Winther 2014	Leisure time computer use and adolescent bone health: Findings from the tromso study-fit futures	Osteoporosis International	Incorrect study type: conference proceeding or abstract

Author and year	Title	Publication source	Reason for exclusion
Winther 2015	Leisure time computer use and adolescent bone health-findings from the Tromso Study, Fit Futures: A cross-sectional study	BMJ Open	Incorrect exposure
Xu 2018	The effect of using geosocial networking apps on the HIV incidence rate among men who have sex with men: eighteen-month prospective cohort study in Shenyang, China	Journal of Medical Internet Research	Incorrect exposure
Yau 2014	Relationships between problematic Internet use and problem-gambling severity: Findings from a high-school survey	Addictive Behaviours	Incorrect exposure
Ybarra 2006	Internet use among Ugandan adolescents: implications for HIV intervention	Plos Medicine	No relevant outcome(s)
Ybarra 2008	Linkages between internet and other media violence with seriously violent behaviour by youth	Paediatrics	Incorrect exposure
Ybarra 2014	Sexual media exposure, sexual behaviour, and sexual violence victimization in adolescence	Clinical Paediatrics	Incorrect exposure
Ybarra 2015	Can clans protect adolescent players of massively multiplayer online games from violent behaviours?	International Journal of Public Health	Incorrect comparator group
Ybarra 2016	A national study of lesbian, gay, bisexual (LGB), and non-LGB youth sexual behaviour online and in-person	Archives of Sexual Behaviour	No relevant outcome(s)
Yonker 2015	"Friending" teens: Systematic review of social media in adolescent and young adult health care	Journal of Medical Internet Research	Incorrect study type: systematic review
Yoo 2014	Associations between overuse of the internet and mental health in adolescents	Nursing & Health Sciences	Incorrect exposure
Young 2011	Online social networking technologies, HIV knowledge, and sexual risk and testing behaviours among homeless youth	AIDS & Behaviour	Incorrect population
Young 2013	Social networking and diffusion of risks and interventions among youth	Sexually Transmitted Infections	Incorrect study type: conference proceeding or abstract
Young 2018	HIV prevention and sex behaviours as organizing mechanisms in a Facebook group affiliation network among young black men who have sex with men	AIDS & Behaviour	Incorrect comparator group
Yu 2017	Predictors and the distal outcome of general Internet use: The identification of children's developmental trajectories	The British Journal of Developmental Psychology	Incorrect exposure

Author and year	Title	Publication source	Reason for exclusion
Yusriani 2020	Education through WhatsApp media in changing of smoking behaviour among senior high school students	National Public Health Journal	Incorrect exposure
Zhan 2019	Electronic cigarette usage patterns: a case study combining survey and social media data	Journal of the American Medical Informatics Association	Incorrect population
Zheng 2021	How does online e-cigarette advertisement promote youth’s e-cigarettes use? The mediating roles of social norm and risk perceptions	Health Communication	Exact duplicate
Zheng 2021	How Does Online e-cigarette Advertisement Promote Youth's e-cigarettes Use? The Mediating Roles of Social Norm and Risk Perceptions	Health Communication	Duplicate sample
Zheng 2021	Social media and E-cigarette use among US youth: Longitudinal evidence on the role of online advertisement exposure and risk perception	Addictive Behaviours	Duplicate sample
Zhou 2014	Internet use and its impact on engagement in leisure activities in China	Plos One	Incorrect exposure
Zhu 2017	Pro-smoking information scanning using social media predicts young adults' smoking behaviour	Computers in Human Behaviour	Incorrect population
Zonfrillo 2014	NekNominate: a deadly, social media-based drinking dare	Clinical Paediatrics	Incorrect study type: commentary/editorial/non-systematic review/theses/book chapter(s)

Appendix 13. Risk of bias domain and overall grade for included datapoints and studies

Table A. Risk of bias domain and overall grades for included cross-sectional and cohort datapoints (n=334), and overall study risk of bias grade (n=122), assessed using adapted Newcastle Ottawa Scale

Author and year	RoB assessment tool	Selection	Exposure	Comparability	Outcome	Overall datapoint RoB	Overall study RoB
Anastario 2020	NOS: cross-sectional studies	Moderate	Moderate	Moderate	Moderate	Moderate	Moderate
Anastario 2020	NOS: cross-sectional studies	Moderate	Moderate	Moderate	Moderate	Moderate	
Baker 2016	NOS: cross-sectional studies	High	High	High	Moderate	High	High
Baker 2016	NOS: cross-sectional studies	High	High	High	Moderate	High	
Baker 2016	NOS: cross-sectional studies	High	High	High	Moderate	High	
Baldwin 2018	NOS: cross-sectional studies	Moderate	Moderate	Low	Moderate	Low	Low
Baldwin 2018	NOS: cross-sectional studies	Moderate	Moderate	Low	Moderate	Low	
Baldwin 2018	NOS: cross-sectional studies	Moderate	Moderate	Low	Moderate	Low	
Baldwin 2018	NOS: cross-sectional studies	Moderate	Moderate	Low	Moderate	Low	
Baldwin 2018	NOS: cross-sectional studies	Moderate	Moderate	Low	Moderate	Low	
Baldwin 2018	NOS: cross-sectional studies	Moderate	Moderate	Low	Moderate	Low	
Baldwin 2018	NOS: cross-sectional studies	Moderate	Moderate	Low	Moderate	Low	
Ball 2020	NOS: cross-sectional studies	Low	Moderate	Low	Moderate	Low	Low
Ball 2020	NOS: cross-sectional studies	Low	Moderate	Low	Moderate	Low	
Baru 2020	NOS: cross-sectional studies	High	Moderate	Moderate	Moderate	High	High
Baumgartner 2012	NOS: cohort studies	Low	Moderate	Moderate	Moderate	Moderate	Moderate
Bayraktar 2007	NOS: cross-sectional studies	High	High	High	Moderate	High	High
Beebe 2004	NOS: cross-sectional studies	Moderate	Moderate	High	Moderate	High	High
Beebe 2004	NOS: cross-sectional studies	Moderate	Moderate	High	Moderate	High	
Beebe 2004	NOS: cross-sectional studies	Moderate	Moderate	High	Moderate	High	
Beebe 2004	NOS: cross-sectional studies	Moderate	Moderate	High	Moderate	High	
Beebe 2004	NOS: cross-sectional studies	Moderate	Moderate	High	Moderate	High	
Beebe 2004	NOS: cross-sectional studies	Moderate	Moderate	High	Moderate	High	
Beebe 2004	NOS: cross-sectional studies	Moderate	Moderate	High	Moderate	High	
Beebe 2004	NOS: cross-sectional studies	Moderate	Moderate	High	Moderate	High	
Beebe 2004	NOS: cross-sectional studies	Moderate	Moderate	High	Moderate	High	
Beebe 2004	NOS: cross-sectional studies	Moderate	Moderate	High	Moderate	High	
Beebe 2004	NOS: cross-sectional studies	Moderate	Moderate	High	Moderate	High	
Beebe 2004	NOS: cross-sectional studies	Moderate	Moderate	High	Moderate	High	
Boers 2020	NOS: cohort studies	Low	Moderate	Low	Moderate	Low	Low
Booker 2015	NOS: cross-sectional studies	Low	Moderate	High	Moderate	High	High
Boniel-Nissim 2022	NOS: cross-sectional studies	Moderate	Low	Low	Low	Low	Low
Boniel-Nissim 2022	NOS: cross-sectional studies	Moderate	Low	Low	Low	Low	
Boniel-Nissim 2022	NOS: cross-sectional studies	Moderate	Low	Low	Low	Low	
Boniel-Nissim 2022	NOS: cross-sectional studies	Moderate	Low	Low	Low	Low	

Author and year	RoB assessment tool	Selection	Exposure	Comparability	Outcome	Overall datapoint RoB	Overall study RoB
Brunborg 2019	NOS: cohort studies	Low	Moderate	Low	Moderate	Low	Low
Brunborg 2019	NOS: cohort studies	Low	Moderate	Low	Low	Low	
Brunborg 2019	NOS: cross-sectional studies	Low	Moderate	High	Moderate	High	
Brunborg 2019	NOS: cross-sectional studies	Low	Moderate	High	Low	High	
Brunborg 2022	NOS: cohort studies	Low	Moderate	Moderate	Moderate	Moderate	Moderate
Camenga 2018	NOS: cohort studies	Moderate	Moderate	Moderate	High	High	High
Canale 2016	NOS: cross-sectional studies	Moderate	High	Low	Low	High	Low
Canale 2016	NOS: cross-sectional studies	Moderate	High	Low	Low	High	
Canale 2016	NOS: cross-sectional studies	Moderate	Moderate	Low	Low	Low	
Casaló 2022	NOS: cross-sectional studies	Moderate	Moderate	Low	Moderate	Low	Low
Casaló 2022	NOS: cross-sectional studies	Moderate	Moderate	Low	Moderate	Low	
Casaló 2022	NOS: cross-sectional studies	Moderate	Moderate	Low	Moderate	Low	
Casaló 2022	NOS: cross-sectional studies	Moderate	Moderate	Low	Moderate	Low	
Cavazos-Rehg 2014	NOS: cross-sectional studies	Moderate	Moderate	High	Moderate	High	High
Chang 2016	NOS: cohort studies	Low	Moderate	High	High	High	High
Chang 2016	NOS: cohort studies	Low	Moderate	High	High	High	
Chapin 2018	NOS: cross-sectional studies	Moderate	Moderate	High	Moderate	High	High
Chapin 2018	NOS: cross-sectional studies	Moderate	Moderate	High	Moderate	High	
Chau 2022	NOS: cross-sectional studies	Low	Moderate	Moderate	Moderate	Moderate	Moderate
Chau 2022	NOS: cross-sectional studies	Low	Moderate	Moderate	Moderate	Moderate	
Chau 2022	NOS: cross-sectional studies	Low	Moderate	Moderate	Moderate	Moderate	
Chau 2022	NOS: cross-sectional studies	Low	Moderate	Moderate	Moderate	Moderate	
Chau 2022	NOS: cross-sectional studies	Low	Moderate	Moderate	Moderate	Moderate	
Chen 2019	NOS: cross-sectional studies	High	Moderate	High	Moderate	High	High
Chen 2019	NOS: cross-sectional studies	High	Moderate	High	Moderate	High	
Coyne 2013	NOS: cross-sectional studies	High	Moderate	High	Low	High	High
Coyne 2018	NOS: cohort studies	Low	Moderate	High	Moderate	High	High
Coyne 2018	NOS: cohort studies	Low	Moderate	High	Moderate	High	
Critchlow 2019	NOS: cross-sectional studies	Low	Moderate	Low	Low	Low	Low
Critchlow 2019	NOS: cross-sectional studies	Low	Moderate	Low	Low	Low	
Critchlow 2019	NOS: cross-sectional studies	Low	Moderate	Low	Low	Low	
da Costa 2021	NOS: cross-sectional studies	Moderate	Moderate	Low	Low	Low	Low
da Costa 2021	NOS: cross-sectional studies	Moderate	Moderate	Low	Low	Low	
da Costa 2021	NOS: cross-sectional studies	Moderate	Moderate	Low	Low	Low	
Dai 2022	NOS: cross-sectional studies	Low	Low	Moderate	Low	Low	Low
Dai 2022	NOS: cross-sectional studies	Low	Low	Moderate	Low	Low	
Davis 2019	NOS: cohort studies	Moderate	Moderate	Moderate	Moderate	Moderate	Moderate
Dawson 2019	NOS: cross-sectional studies	High	Moderate	High	Moderate	High	High
Dawson 2019	NOS: cross-sectional studies	High	Low	High	Moderate	High	
Dawson 2019	NOS: cross-sectional studies	High	Low	High	Moderate	High	
de Bruijn 2016	NOS: cross-sectional studies	Low	Moderate	Moderate	Moderate	Moderate	Moderate
de Bruijn 2016	NOS: cross-sectional studies	Low	Moderate	Moderate	Moderate	Moderate	

Author and year	RoB assessment tool	Selection	Exposure	Comparability	Outcome	Overall datapoint RoB	Overall study RoB
De Looze 2019	NOS: cross-sectional studies	Moderate	Moderate	Moderate	Moderate	Moderate	Moderate
De Looze 2019	NOS: cross-sectional studies	Moderate	Moderate	Low	Moderate	Low	
De Looze 2019	NOS: cross-sectional studies	Moderate	Moderate	Moderate	Moderate	Moderate	
Doornwaard 2014	NOS: cross-sectional studies	High	Low	High	Low	High	High
Doornwaard 2015	NOS: cross-sectional studies	Moderate	Moderate	High	Moderate	High	Moderate
Doornwaard 2015	NOS: cross-sectional studies	Moderate	Moderate	High	Moderate	High	
Doornwaard 2015	NOS: cross-sectional studies	Moderate	Moderate	Moderate	Moderate	Moderate	
Doornwaard 2015	NOS: cross-sectional studies	Moderate	Moderate	Moderate	Moderate	Moderate	
Elton-Marshall 2016	NOS: cross-sectional studies	Moderate	Moderate	High	Moderate	High	High
Elton-Marshall 2016	NOS: cross-sectional studies	Moderate	Moderate	High	Low	High	
Erreygers 2017	NOS: cross-sectional studies	Moderate	Moderate	High	Moderate	High	High
Floros 2013	NOS: cross-sectional studies	Moderate	High	Moderate	Moderate	High	High
Floros 2013	NOS: cross-sectional studies	Moderate	High	High	Low	High	
Froyland 2020	NOS: cross-sectional studies	Low	Moderate	Low	Moderate	Low	Low
Froyland 2020	NOS: cross-sectional studies	Low	Moderate	Low	Moderate	Low	
Froyland 2020	NOS: cross-sectional studies	Low	Moderate	High	Moderate	High	
Froyland 2020	NOS: cross-sectional studies	Low	Moderate	High	Moderate	High	
Froyland 2020	NOS: cross-sectional studies	Low	Moderate	High	Moderate	High	
Froyland 2020	NOS: cross-sectional studies	Low	Moderate	High	Moderate	High	
Froyland 2020	NOS: cross-sectional studies	Low	Moderate	High	Moderate	High	
Froyland 2020	NOS: cross-sectional studies	Low	Moderate	High	Moderate	High	
Gascoyne 2021	NOS: cross-sectional studies	Moderate	Moderate	Low	Moderate	Low	Low
Gascoyne 2021	NOS: cross-sectional studies	Moderate	Moderate	Low	Moderate	Low	
Gazendam 2020	NOS: cross-sectional studies	Moderate	Moderate	Low	Moderate	Low	Low
Gazendam 2020	NOS: cross-sectional studies	Moderate	Moderate	Low	Moderate	Low	
Geber 2021	NOS: cohort studies	Low	Moderate	Moderate	Moderate	Moderate	Moderate
Geusens 2017	NOS: cohort studies	Moderate	Moderate	Moderate	High	High	High
Geusens 2017	NOS: cross-sectional studies	High	Moderate	High	Moderate	High	
Geusens 2017	NOS: cross-sectional studies	Moderate	Moderate	Moderate	Low	Moderate	Moderate
Geusens 2017	NOS: cross-sectional studies	Moderate	Moderate	Moderate	Low	Moderate	
Geusens 2019	NOS: cross-sectional studies	Moderate	Moderate	Low	Moderate	Low	Low
Geusens 2019	NOS: cross-sectional studies	Moderate	Moderate	Low	Moderate	Low	
Gomez 2019	NOS: cross-sectional studies	Moderate	High	High	High	High	High
Gordon 2011	NOS: cross-sectional studies	High	Moderate	High	Moderate	High	High
Gordon 2011	NOS: cross-sectional studies	High	Moderate	High	Moderate	High	
Gregg 2018	NOS: cross-sectional studies	Moderate	Moderate	Moderate	Low	Moderate	Moderate
Gunnlaugsson 2020	NOS: cross-sectional studies	Moderate	Low	Moderate	Low	Low	Low
Gunnlaugsson 2020	NOS: cross-sectional studies	Moderate	Low	Moderate	Low	Low	
Gunnlaugsson 2020	NOS: cross-sectional studies	Moderate	Low	Moderate	Low	Low	
Hamilton 2020	NOS: cross-sectional studies	Low	Moderate	High	Moderate	High	High
Hayer 2018	NOS: cohort studies	Low	Moderate	Moderate	Moderate	Moderate	Moderate
Hayer 2018	NOS: cohort studies	Low	Moderate	Moderate	Moderate	Moderate	
Holtz 2011	NOS: cross-sectional studies	Moderate	Moderate	Moderate	Low	Moderate	Moderate

Author and year	RoB assessment tool	Selection	Exposure	Comparability	Outcome	Overall datapoint RoB	Overall study RoB
Hryhorczuk 2019	NOS: cross-sectional studies	Moderate	Moderate	Moderate	Moderate	Moderate	Moderate
Hryhorczuk 2019	NOS: cross-sectional studies	Moderate	Moderate	Moderate	Moderate	Moderate	
Hryhorczuk 2019	NOS: cross-sectional studies	Moderate	Moderate	Moderate	Moderate	Moderate	
Hryhorczuk 2019	NOS: cross-sectional studies	Moderate	Moderate	Moderate	Moderate	Moderate	
Hryhorczuk 2019	NOS: cross-sectional studies	Moderate	Moderate	Moderate	Moderate	Moderate	
Hryhorczuk 2019	NOS: cross-sectional studies	Moderate	Moderate	Moderate	Moderate	Moderate	
Hrywna 2020	NOS: cross-sectional studies	Low	Moderate	Moderate	Moderate	Moderate	Moderate
Hrywna 2020	NOS: cross-sectional studies	Low	Moderate	Moderate	Moderate	Moderate	
Huang 2012	NOS: cross-sectional studies	Moderate	Moderate	Low	Moderate	Low	Low
Huang 2014	NOS: cohort studies	Low	Moderate	Low	Moderate	Low	Low
Huang 2014	NOS: cohort studies	Low	Moderate	Low	Moderate	Low	
Huang 2014	NOS: cohort studies	Low	Moderate	Low	Moderate	Low	
Huang 2014	NOS: cohort studies	Low	Moderate	Low	Moderate	Low	
Jeong 2022	NOS: cross-sectional studies	Moderate	Moderate	High	Low	High	High
Jiang 2018	NOS: cross-sectional studies	High	Low	Low	Low	High	High
Kaufman 2014	NOS: cross-sectional studies	Moderate	Moderate	Low	Low	Low	Low
Kaufman 2014	NOS: cross-sectional studies	Moderate	Moderate	Moderate	Low	Moderate	
Kaufman 2014	NOS: cross-sectional studies	Moderate	Moderate	Low	Low	Low	
Kaufman 2014	NOS: cross-sectional studies	Moderate	Moderate	Moderate	Low	Moderate	
Kaufman 2014	NOS: cross-sectional studies	Moderate	Moderate	Low	Moderate	Low	
Kaufman 2014	NOS: cross-sectional studies	Moderate	Moderate	Moderate	Moderate	Moderate	
Kaufman 2014	NOS: cross-sectional studies	Moderate	Moderate	Low	Moderate	Low	
Kaufman 2014	NOS: cross-sectional studies	Moderate	Moderate	Moderate	Moderate	Moderate	
Kaur 2020	NOS: cross-sectional studies	Low	Moderate	Moderate	Moderate	Low	Low
Kaur 2020	NOS: cross-sectional studies	Low	Moderate	Moderate	Moderate	Low	
Kaur 2020	NOS: cross-sectional studies	Low	Moderate	Moderate	Moderate	Low	
Kaur 2020	NOS: cross-sectional studies	Low	Moderate	Moderate	Moderate	Low	
Kaur 2020	NOS: cross-sectional studies	Low	Moderate	Moderate	Moderate	Low	
Kaur 2020	NOS: cross-sectional studies	Low	Moderate	Moderate	Moderate	Low	
Kelleghan 2020	NOS: cohort studies	Low	Moderate	Moderate	Moderate	Moderate	Moderate
Kelleghan 2020	NOS: cohort studies	Low	Moderate	Moderate	Moderate	Moderate	
Kelleghan 2020	NOS: cohort studies	Low	Moderate	Moderate	Moderate	Moderate	
Kelleghan 2020	NOS: cohort studies	Low	Moderate	Moderate	Moderate	Moderate	
Kelleghan 2020	NOS: cohort studies	Low	Moderate	Moderate	Moderate	Moderate	
Kelleghan 2020	NOS: cohort studies	Low	Moderate	Moderate	Moderate	Moderate	
King 2014	NOS: cross-sectional studies	High	Moderate	High	Low	High	High
Ko 2009	NOS: cross-sectional studies	Low	Moderate	Moderate	Moderate	Moderate	Moderate
Ko 2009	NOS: cross-sectional studies	Low	Moderate	Moderate	Moderate	Moderate	
Ko 2009	NOS: cross-sectional studies	Low	Moderate	Moderate	Moderate	Moderate	

Author and year	RoB assessment tool	Selection	Exposure	Comparability	Outcome	Overall datapoint RoB	Overall study RoB
Kontostoli 2022	NOS: cross-sectional studies	Moderate	Moderate	Low	Low	Low	Low
Kontostoli 2022	NOS: cross-sectional studies	Moderate	Moderate	Low	Low	Low	
Kontostoli 2022	NOS: cross-sectional studies	Moderate	Moderate	Low	Moderate	Low	
Kontostoli 2022	NOS: cross-sectional studies	Moderate	Moderate	Low	Low	Low	
Kontostoli 2022	NOS: cross-sectional studies	Moderate	Moderate	Low	Low	Low	
Kontostoli 2022	NOS: cross-sectional studies	Moderate	Moderate	Low	Moderate	Low	
Koutamanis 2015	NOS: cross-sectional studies	Moderate	Moderate	High	Moderate	High	High
Kwon 2022	NOS: cross-sectional studies	Moderate	Moderate	Low	Moderate	Low	Low
Kwon 2022	NOS: cross-sectional studies	Moderate	Moderate	Low	Moderate	Low	
Kwon 2022	NOS: cross-sectional studies	Moderate	Moderate	Low	Moderate	Low	
Kwon 2022	NOS: cross-sectional studies	Moderate	Moderate	Low	Moderate	Low	
Landry 2013	NOS: cross-sectional studies	High	Moderate	Moderate	Moderate	High	High
Landry 2013	NOS: cross-sectional studies	High	Moderate	High	Moderate	High	
Larm 2017	NOS: cross-sectional studies	Moderate	Moderate	High	Low	High	High
Larm 2017	NOS: cross-sectional studies	Moderate	Moderate	High	Low	High	
Larm 2019	NOS: cross-sectional studies	High	Moderate	Moderate	Low	High	High
Larm 2019	NOS: cross-sectional studies	High	Moderate	Moderate	Low	High	
Lee 2015	NOS: cross-sectional studies	High	Moderate	Moderate	Moderate	High	High
Lee 2019	NOS: cohort studies	Low	Moderate	Moderate	High	High	High
Lee 2019	NOS: cohort studies	Low	Moderate	Moderate	Moderate	Moderate	
Lee 2021	NOS: cross-sectional studies	High	Low	Moderate	Low	High	High
Lee 2021	NOS: cross-sectional studies	High	Low	Moderate	Low	High	
Lee 2021	NOS: cross-sectional studies	Moderate	Moderate	Moderate	Moderate	Moderate	Moderate
Lee 2021	NOS: cross-sectional studies	Moderate	Moderate	Moderate	Moderate	Moderate	
Lin 2012	NOS: cross-sectional studies	Low	Moderate	High	Moderate	High	High
Lin 2012	NOS: cross-sectional studies	Low	Moderate	High	Moderate	High	
Lipsky 2017	NOS: cohort studies	Low	Moderate	Low	Low	Low	Low
Lipsky 2017	NOS: cohort studies	Low	Moderate	Low	Low	Low	
Lipsky 2017	NOS: cohort studies	Low	Moderate	Low	Low	Low	
Longobardi 2021	NOS: cross-sectional studies	Moderate	High	High	Low	High	High
McClure 2020	NOS: cross-sectional studies	High	Moderate	Low	Moderate	High	High
Merrill 2019	NOS: cross-sectional studies	High	Moderate	Moderate	Moderate	High	High
Michael 2016	NOS: cross-sectional studies	Moderate	High	High	High	High	High
Moitra 2022	NOS: cross-sectional studies	Moderate	Moderate	Low	Moderate	Low	Low
Moitra 2022	NOS: cross-sectional studies	Moderate	Moderate	Low	Low	Low	
Mojica 2014	NOS: cross-sectional studies	High	Moderate	Moderate	Moderate	High	High
Mojica 2014	NOS: cross-sectional studies	High	Moderate	Moderate	Moderate	High	
Mojica 2014	NOS: cross-sectional studies	High	Moderate	Moderate	Moderate	High	
Molla-Esparza 2021	NOS: cross-sectional studies	Low	Moderate	Moderate	Moderate	Moderate	Moderate
Molla-Esparza 2021	NOS: cross-sectional studies	Low	Moderate	Moderate	Moderate	Moderate	
Molla-Esparza 2021	NOS: cross-sectional studies	Low	Moderate	Moderate	Moderate	Moderate	

Author and year	RoB assessment tool	Selection	Exposure	Comparability	Outcome	Overall datapoint RoB	Overall study RoB
Nesi 2017	NOS: cohort studies	Moderate	Moderate	Moderate	Moderate	Moderate	Moderate
Nesi 2017	NOS: cohort studies	Moderate	Moderate	Moderate	Moderate	Moderate	
Nesi 2017	NOS: cohort studies	Moderate	Moderate	Moderate	Moderate	Moderate	
Nesi 2017	NOS: cohort studies	Moderate	Moderate	Moderate	Moderate	Moderate	
Nesi 2017	NOS: cohort studies	Moderate	Moderate	Moderate	Moderate	Moderate	
Nesi 2017	NOS: cohort studies	Moderate	Moderate	Moderate	Moderate	Moderate	
Nesi 2019	NOS: cohort studies	Low	Moderate	High	Moderate	High	High
Nesi 2019	NOS: cohort studies	Low	Moderate	High	Moderate	High	
Nesi 2019	NOS: cohort studies	Low	Moderate	High	Moderate	High	
Nesi 2019	NOS: cohort studies	Low	Low	High	Moderate	High	
Nesi 2019	NOS: cohort studies	Low	Low	High	Moderate	High	
Nesi 2019	NOS: cross-sectional studies	Moderate	Moderate	High	Moderate	High	
Nesi 2019	NOS: cross-sectional studies	Moderate	Moderate	High	Moderate	High	
Nesi 2019	NOS: cross-sectional studies	Moderate	Moderate	High	Moderate	High	
Nesi 2019	NOS: cross-sectional studies	Moderate	Low	High	Moderate	High	
Nesi 2019	NOS: cross-sectional studies	Moderate	Low	High	Moderate	High	
Ng Fat 2021	NOS: cohort studies	Low	Moderate	Low	Moderate	Low	Low
Ng Fat 2021	NOS: cohort studies	Low	Moderate	Low	Moderate	Low	
Ng Fat 2021	NOS: cohort studies	Low	Moderate	Low	Moderate	Low	
Ng Fat 2021	NOS: cross-sectional studies	Low	Moderate	Low	Moderate	Low	
Ng Fat 2021	NOS: cross-sectional studies	Low	Moderate	Low	Moderate	Low	
Ng Fat 2021	NOS: cross-sectional studies	Low	Moderate	Low	Moderate	Low	
Ng Fat 2021	NOS: cross-sectional studies	Low	Moderate	Low	Moderate	Low	
Ng Fat 2021	NOS: cross-sectional studies	Low	Moderate	Low	Moderate	Low	
Ohannessian 2009	NOS: cross-sectional studies	High	Moderate	High	Moderate	High	High
Ohannessian 2009	NOS: cross-sectional studies	High	Moderate	High	Moderate	High	
Pegg 2018	NOS: cross-sectional studies	High	Moderate	Moderate	Moderate	High	High
Pegg 2018	NOS: cross-sectional studies	High	Moderate	Moderate	Moderate	High	
Pérez 2022	NOS: cohort studies	Low	Moderate	Low	Moderate	Low	Low
Prince 2021	NOS: cross-sectional studies	Low	Moderate	High	Moderate	High	High
Qutteina 2022	NOS: cross-sectional studies	Low	Moderate	Moderate	Low	Moderate	Moderate
Qutteina 2022	NOS: cross-sectional studies	Low	Moderate	Moderate	Low	Moderate	
Qutteina 2022	NOS: cross-sectional studies	Low	Moderate	Moderate	Low	Moderate	
Qutteina 2022	NOS: cross-sectional studies	Low	Moderate	Moderate	Low	Moderate	
Qutteina 2022	NOS: cross-sectional studies	Low	Moderate	Moderate	Low	Moderate	
Riehm 2021	NOS: cross-sectional studies	Moderate	Moderate	High	Moderate	High	High
Roditis 2016	NOS: cross-sectional studies	Low	Moderate	Moderate	Moderate	Moderate	Moderate
Roditis 2016	NOS: cross-sectional studies	Low	Moderate	Moderate	Moderate	Moderate	

Author and year	RoB assessment tool	Selection	Exposure	Comparability	Outcome	Overall datapoint RoB	Overall study RoB
Romo 2017	NOS: cross-sectional studies	High	Low	Moderate	Low	High	High
Romo 2017	NOS: cross-sectional studies	High	Low	Moderate	Low	High	
Romo 2017	NOS: cross-sectional studies	High	Low	Moderate	Low	High	
Romo 2017	NOS: cross-sectional studies	High	Low	Moderate	Low	High	
Romo 2017	NOS: cross-sectional studies	High	Low	Moderate	Low	High	
Romo 2017	NOS: cross-sectional studies	High	Low	Moderate	Low	High	
Romo 2017	NOS: cross-sectional studies	High	Low	Moderate	Low	High	
Romo 2017	NOS: cross-sectional studies	High	Low	Moderate	Low	High	
Rutter 2021	NOS: cross-sectional studies	Moderate	Moderate	Moderate	Moderate	Moderate	Moderate
Sampasa-Kanyinga 2015	NOS: cross-sectional studies	Moderate	Moderate	Low	Moderate	Low	Low
Sampasa-Kanyinga 2015	NOS: cross-sectional studies	Moderate	Moderate	Low	Moderate	Low	
Sampasa-Kanyinga 2015	NOS: cross-sectional studies	Moderate	Moderate	Low	Moderate	Low	
Sampasa-Kanyinga 2015	NOS: cross-sectional studies	Moderate	Moderate	High	Moderate	High	High
Sampasa-Kanyinga 2015	NOS: cross-sectional studies	Moderate	Moderate	High	Moderate	High	
Sampasa-Kanyinga 2016	NOS: cross-sectional studies	Low	Moderate	Low	Moderate	Low	Low
Sampasa-Kanyinga 2016	NOS: cross-sectional studies	Low	Moderate	Low	Moderate	Low	
Sampasa-Kanyinga 2016	NOS: cross-sectional studies	Low	Moderate	Low	Moderate	Low	
Sampasa-Kanyinga 2016	NOS: cross-sectional studies	Low	Moderate	Low	Moderate	Low	
Sampasa-Kanyinga 2016	NOS: cross-sectional studies	Low	Moderate	Low	Moderate	Low	
Sampasa-Kanyinga 2016	NOS: cross-sectional studies	Low	Moderate	Low	Moderate	Low	
Sampasa-Kanyinga 2016	NOS: cross-sectional studies	Moderate	Moderate	Low	Moderate	Low	Low
Sampasa-Kanyinga 2016	NOS: cross-sectional studies	Moderate	Moderate	Low	Moderate	Low	
Sampasa-Kanyinga 2018	NOS: cross-sectional studies	Moderate	Moderate	Moderate	Moderate	Moderate	Moderate
Sandercock 2016	NOS: cross-sectional studies	Moderate	Moderate	Low	Moderate	Low	Low
Sandercock 2016	NOS: cross-sectional studies	Moderate	Moderate	Low	Moderate	Low	
Sandercock 2016	NOS: cross-sectional studies	Moderate	Moderate	Low	Moderate	Low	
Sandercock 2016	NOS: cross-sectional studies	Moderate	Moderate	Low	Moderate	Low	
Sandercock 2016	NOS: cross-sectional studies	Moderate	Moderate	Low	Low	Low	

Author and year	RoB assessment tool	Selection	Exposure	Comparability	Outcome	Overall datapoint RoB	Overall study RoB
Savolainen 2020	NOS: cross-sectional studies	High	Low	Moderate	Low	High	High
Savolainen 2020	NOS: cross-sectional studies	High	Low	Moderate	Low	High	
Savolainen 2020	NOS: cross-sectional studies	High	Low	Moderate	Low	High	
Savolainen 2020	NOS: cross-sectional studies	High	Low	Moderate	Low	High	
Savolainen 2020	NOS: cross-sectional studies	High	Low	Moderate	Low	High	
Savolainen 2020	NOS: cross-sectional studies	High	Low	Moderate	Low	High	
Savolainen 2020	NOS: cross-sectional studies	High	Low	Moderate	Low	High	
Savolainen 2020	NOS: cross-sectional studies	High	Low	Moderate	Low	High	
Savolainen 2020	NOS: cross-sectional studies	High	Low	Moderate	Low	High	
Savolainen 2020	NOS: cross-sectional studies	High	Low	Moderate	Low	High	
Savolainen 2020	NOS: cross-sectional studies	High	Low	Moderate	Low	High	
Savolainen 2020	NOS: cross-sectional studies	High	Low	Moderate	Low	High	
Savolainen 2020	NOS: cross-sectional studies	High	Low	Moderate	Low	High	
Savolainen 2020	NOS: cross-sectional studies	High	Low	Moderate	Low	High	
Savolainen 2020	NOS: cross-sectional studies	High	Low	Moderate	Low	High	
Savolainen 2020	NOS: cross-sectional studies	High	Low	Moderate	Low	High	
Savolainen 2020	NOS: cross-sectional studies	High	Low	Moderate	Low	High	
Savolainen 2020	NOS: cross-sectional studies	High	Low	Moderate	Low	High	
Savolainen 2020	NOS: cross-sectional studies	High	Low	Moderate	Low	High	
Savolainen 2020	NOS: cross-sectional studies	High	Low	Moderate	Low	High	
Self-Brown 2018	NOS: cross-sectional studies	Low	Low	High	Low	High	High
Self-Brown 2018	NOS: cross-sectional studies	Low	Low	High	Low	High	
Shan 2022	NOS: cohort studies	Low	Moderate	Low	Moderate	Low	Low
Shan 2022	NOS: cohort studies	Low	Moderate	Low	Moderate	Low	
Sharma 2021	NOS: cross-sectional studies	Low	Moderate	High	High	High	High
Shimoga 2019	NOS: cross-sectional studies	Moderate	Moderate	Low	Moderate	Low	Low
Smout 2021	NOS: cohort studies	Low	Moderate	Moderate	Moderate	Moderate	Moderate
Smout 2021	NOS: cohort studies	Low	Moderate	Moderate	Moderate	Moderate	
Soneji 2018	NOS: cohort studies	Low	Moderate	Low	Moderate	Low	Low
Soneji 2018	NOS: cohort studies	Low	Moderate	Low	Moderate	Low	
Soneji 2018	NOS: cohort studies	Low	Moderate	Low	Moderate	Low	
Soneji 2018	NOS: cohort studies	Low	Moderate	Low	Moderate	Low	
Stevens 2017	NOS: cross-sectional studies	Moderate	Moderate	Low	Moderate	Low	Low
Stevens 2017	NOS: cross-sectional studies	Moderate	Moderate	Low	Moderate	Low	
Suwanwong 2021	NOS: cross-sectional studies	High	Moderate	High	Moderate	High	High
Suwanwong 2021	NOS: cross-sectional studies	High	Moderate	High	Moderate	High	
Svensson 2020	NOS: cross-sectional studies	Low	Moderate	Moderate	Moderate	Moderate	Moderate
Svensson 2020	NOS: cross-sectional studies	Low	Moderate	Moderate	Moderate	Moderate	
Svensson 2020	NOS: cross-sectional studies	Low	Moderate	Moderate	Moderate	Moderate	
Tao 2022	NOS: cross-sectional studies	Low	Moderate	High	Low	High	High
Tao 2022	NOS: cross-sectional studies	Low	Moderate	High	Low	High	
Trangenstein 2019	NOS: cross-sectional studies	High	Moderate	High	Moderate	High	High
Trangenstein 2019	NOS: cross-sectional studies	High	Moderate	High	Moderate	High	

Author and year	RoB assessment tool	Selection	Exposure	Comparability	Outcome	Overall datapoint RoB	Overall study RoB
Tsitsika 2009	NOS: cross-sectional studies	Moderate	High	High	Moderate	High	High
Tsitsika 2011	NOS: cross-sectional studies	Low	Moderate	High	Moderate	High	High
Vandenbosch 2016	NOS: cross-sectional studies	Moderate	Moderate	High	Moderate	High	High
Vannucci 2019	NOS: cohort studies	Low	Moderate	Low	Low	Low	Low
Vannucci 2019	NOS: cross-sectional studies	Low	Moderate	High	Low	High	
Vannucci 2019	NOS: cross-sectional studies	Low	Moderate	High	Low	High	
Vannucci 2019	NOS: cross-sectional studies	Low	Moderate	High	Low	High	
Vazquez-Nava 2020	NOS: cross-sectional studies	Moderate	Low	High	Low	High	High
Vente 2020	NOS: cross-sectional studies	Moderate	Moderate	High	Moderate	High	High
Vente 2020	NOS: cross-sectional studies	Moderate	Moderate	High	Moderate	High	
Wana 2019	NOS: cross-sectional studies	Low	Moderate	Moderate	Moderate	Moderate	Moderate
Ward 2022	NOS: cross-sectional studies	Moderate	Moderate	Moderate	Moderate	Moderate	Moderate
Ward 2022	NOS: cross-sectional studies	Moderate	Moderate	Moderate	Moderate	Moderate	
Ward 2022	NOS: cross-sectional studies	Moderate	Moderate	Moderate	Moderate	Moderate	
Whitehill 2020	NOS: cross-sectional studies	High	Moderate	High	Moderate	High	High
Whitehill 2020	NOS: cross-sectional studies	High	Moderate	High	Moderate	High	
Whitehill 2020	NOS: cross-sectional studies	High	Moderate	High	Moderate	High	
Whitehill 2020	NOS: cross-sectional studies	High	Moderate	High	Moderate	High	
Widman 2014	NOS: cross-sectional studies	High	Moderate	High	Moderate	High	High
Widman 2014	NOS: cross-sectional studies	High	Moderate	High	Moderate	High	
Worku 2022	NOS: cross-sectional studies	Low	Moderate	Moderate	Low	Moderate	Moderate
Wulff 2021	NOS: cross-sectional studies	Moderate	Moderate	Moderate	Low	Moderate	Moderate
Yao 2022	NOS: cross-sectional studies	Moderate	Moderate	Moderate	Moderate	Moderate	Moderate
Yao 2022	NOS: cross-sectional studies	Moderate	Moderate	Moderate	Moderate	Moderate	

Legend: Abbreviations: NOS = Adapted Newcastle Ottawa Scale and RoB = Risk of bias.

Table B. Risk of bias domain and overall grades for included randomised control trial datapoints (n=4), and overall study risk of bias grade (n=4), assessed using Cochrane Risk of Bias 2 tool

Author and year	RoB assessment tool	Randomisation	Adherence	Missingness	Measurement	Reporting	Overall datapoint RoB	Overall study RoB
Coates 2019	RoB-2	Low	Some concerns	Low	Low	Some concerns	Some concerns	Some concerns
De Jans 2021	RoB-2	Low	Low	Low	Low	Low	Low	Low
Folkvord 2020	RoB-2	Low	Low	Low	Low	Low	Low	Low
Ngqangashe 2021	RoB-2	Some concerns	Low	Low	Low	Low	Some concerns	Some concerns

Legend: Abbreviations: RoB = Risk of bias and RoB-2 = Cochrane Risk of Bias 2 tool.

Appendix 14. Social media measures reported in included studies

Table A below outlines the social media exposure measures (n=253) used to assess social media use across included studies. Within included studies, many social media exposure measures were reported and were incorporated in our exploration of how SM use is measured in relation to adolescent health-risk behaviours, therefore the number of datapoints reported differ from those included in the review synthesis. Please also note this table records the exposure measures in their original form as reported in included studies, in some instances the exposure measures may have been transformed/specific pairwise comparisons selected for inclusion in meta-analyses/SWiM as per the decisions rules outlined in Appendix 7. The final three columns, titled *'SM platform'*, *'SM category (active/passive use)'* and *'SM content'*, used information from included studies to categorise the social media platform, category and content type under study. The categorisation was conducted using the 'Process of social media categorisation' presented in Appendix 4.

Table A. Social media measures reported in included studies

**Exposure**	**Author and year**	**Exposure definition**	**Exposure ascertainment**	**Validated/ objectively recorded**	**Exposure measure type**	**Exposure time period**	**SM platform**	***SM platform***	***SM category (active/ passive use)***	***SM content***
Time Spent on SM	Brunborg 2019	Change in hrs of SM use per day (Δ = T2 – T1)	<u>Self-report 2-item measure:</u><br>1-Frequency of SM use in the past 6/12 months. Participant asked to report on active SM use: reading, writing, watching pictures, making comments, or appointments on SM etc, and not merely the time logged on.<br>Responses: every day to not at all. Responses recoded into the average number of days per month spent on SM.<br>2-How many hrs/day usually spent on SM.<br>Responses: < 1 hr to >15 hrs/day in hourly increments.<br>Product of frequency (average days/month) and quantity (average hrs/day) divided by 30 to reflect the average number of hrs spent on SM per day.		Continuous	T1: past 12 months<br>T2: past 6 months	Examples: Facebook, Snapchat, WhatsApp, Twitter, Instagram, and Kik	Mixed platforms	SNS (active use)	NA
Time Spent on SM	Brunborg 2019	Average number of hrs spent on SM per day	<u>Self-report 2-item measure:</u><br>1-Frequency of SM use in the past 6/12 months. Respondents asked to report on active SM use: reading, writing, watching pictures, making comments, or appointments on SM etc, and not merely the time logged on.<br>Responses: every day to not at all. Responses recoded into the average number of days per month spent on SM.<br>2-How many hrs per day usually spent on SM.<br>Responses: < 1 hr to >15 hrs/day in hourly increments.<br>Product of frequency (average days/month) and quantity (average hrs/day) divided by 30 to reflect the average number of hrs spent on SM per day.		Continuous	Past 12 months	Examples: Facebook, Snapchat, WhatsApp, Twitter, Instagram, and Kik	Mixed platforms	SNS (active use)	NA

Exposure	Author and year	Exposure definition	Exposure ascertainment	Validated/ objectively recorded	Exposure measure type	Exposure time period	SM platform	*SM platform*	*SM category (active/ passive use)*	*SM content*
Time Spent on SM	Brunborg 2022	Average number of hrs spent on SM per day	Self-report 2-item measure:<br>1-Frequency of active use of SM (e.g., Facebook, Snapchat, and Instagram) in the past 30 days<br>Responses: not at all to 5-days a week<br>2-How many hrs per day spent actively using SM<br>Responses: less than 1 hr to 10 hr or more<br>Product of frequency (days per month) and quantity (average hrs/day) divided by 30 to reflect average number of hrs spent on SM per day		Continuous	Past month	Examples: Facebook, Snapchat, and Instagram	Mixed platforms	General SM (active use)	NA
Time Spent on SM	Boers 2020	Time spent on SM per day	Self-report 1-item measure:<br>1-How much time spent on Facebook, Twitter or other SNS per day.<br>Responses: 0–30 min, 30 min – 1 hr 30 min, 1 hr 30 min – 2 hrs 30 min, and ≥3 hrs 30 min/day.		Continuous	Current	Specified: Facebook, Twitter, and other SNS	Mixed platforms	SNS (unclear)	NA
Time Spent on SM	Booker 2015	Time spent chatting on social websites on a normal school day	Self-report 1-item measure:<br>1-How many hrs spent chatting or interacting with friends through a social web site like Bebo, Facebook, and Myspace on a normal school day.<br>Responses: (1) none to (5) ≥7 hrs/day.<br>3 category variable constructed: <1 hr, 1 to 3 hrs, and ≥4 hrs/day.		Continuous	Current	Examples: Bebo, Facebook, and Myspace	Mixed platforms	SNS (active use)	NA
Time Spent on SM	Casaló 2022	Time spent on SNS per day	Self-report 1-item measure:<br>1-Time spent on SNS for fun (and not to do homework/work) per day<br>Responses: no time devoted, 30 mins or less, around an hr, 2-3 hrs, 4 or more hrs/day		Continuous	Current	NR	NR	SNS (unclear)	NA
Time Spent on SM	Chau 2022	Time spent on discussion forums and chatting online during a weekday	Self-report 1-item measure:<br>1-How many hours spent with discussion forums and chatting online during a weekday<br>Responses: (1) <2 hrs (2) 2-4 hrs (3) 5 or more hrs/day		Categorical	Current	NR	NR	Blogs & Forums (active use)	NA
Time Spent on SM	Chau 2022	Time spent on discussion forums and chatting online during a weekend day	Self-report 1-item measure:<br>1-How many hours spent with discussion forums and chatting online during a weekend day<br>Responses: (1) <2 hrs (2) 2-4 hrs (3) 5 or more hrs/day		Categorical	Current	NR	NR	Blogs & Forums (active use)	NA

Exposure	Author and year	Exposure definition	Exposure ascertainment	Validated/ objectively recorded	Exposure measure type	Exposure time period	SM platform	*SM platform*	*SM category (active/ passive use)*	*SM content*
Time Spent on SM	Chen 2019	Time spent on SM on a regular weekday and weekend day	Self-report 8-item measure:<br>8 questions about time spent on Facebook, Instagram, and Snapchat on a regular weekday (Monday–Friday) and a weekend day (Saturday and Sunday).<br>Responses: (0) from 0 hrs to (10) the platform is opened throughout the day continuously.<br>Weighted score of average daily time for each SM platform computed: (time weekday × 5 + time weekend day × 2)/7.<br>SM use computed by the average score of the 3 platforms.		Continuous	Current	Specified: Facebook, Instagram, and Snapchat	Mixed platforms	SNS (unclear)	NA
Time Spent on SM	Coyne 2013	Time spent on SNS on a typical day	Self-report 1-item measure:<br>1-How much time spent on SNS in a typical day.<br>Responses: 9-point scale from (1) none to (9) >8 hrs/day.		Continuous	Current	NR	NR	SNS (unclear)	NA
Time Spent on SM	Coyne 2018	Time spent on SNS on a typical day	Self-report 1-item measure, assessed at 6 timepoints over 6 years:<br>1-How much time spent on SNS, like Facebook, on a typical day.<br>Responses: (1) none to (9) >8 hrs/day.<br>3 category variable constructed: peak users (low SM use that increases quickly after a few years and then returns to baseline), moderate users (steady SM use over time), and increasers (low SM use that increases gradually and ends high at the end of the study).		Categorical	Current	Examples: Facebook and Instagram	Mixed platforms	SNS (unclear)	NA
Time Spent on SM	da Costa 2021	Time spent on SM on a typical weekday and weekend day	Self-report 2-item measure:<br>1-Time spent engaged on SM on a typical weekday<br>2-Time spent engaged on SM on a typical weekend day<br>Daily time on SM estimated by weighting answers ([volume on weekdays x 5 + volume on weekend x 2]/7) hrs/day<br>4 category variable constructed: <2 hrs, ≥2 hrs, <4 hrs, and ≥4 hrs/day		Continuous	Current	NR	NR	General SM (unclear)	NA
Time Spent on SM	Doornwaard 2015	Time spent on SNS (most frequently used platform) per day	Self-report 1-item measure:<br>1-How much time actively spent each day on most used SNS.<br>Responses: not an SNS member, <15 mins, 15–30 mins, 30–60 mins, 1–2 hrs, 3–4 hrs, and >4 hrs/day.		Ordinal	Current	NR	NR	SNS (active use)	NA

Exposure	Author and year	Exposure definition	Exposure ascertainment	Validated/ objectively recorded	Exposure measure type	Exposure time period	SM platform	*SM platform*	*SM category (active/ passive use)*	*SM content*
Time Spent on SM	Froyland 2020	Time spent on SM per day	Self-report 1-item measure:<br>1-How much time spent daily on SM (e.g., Facebook, Instagram, etc)<br>Responses: non, <30 min, 30 min-1 hr, 1-2 hrs, 2-3 hrs, >3 hrs.		Continuous	Current	Examples: Facebook and Instagram	Mixed platforms	General SM (unclear)	NA
Time Spent on SM	Gazendam 2020	Time spent on SM per day	Self-report 1-item measure:<br>1-How many hrs a day, in free time, spent using electronic devices such as computers, tablets (like iPad) or smartphone for other purposes (e.g., tweeting, Facebook, chatting)<br>Responses: none at all, about 30 mins, about 1 hr, about 2 hrs, about ≥3 hrs/day.<br>Variable dichotomised: <3hrs/ ≥3 hrs/day		Binary	Current	Examples: Facebook, chatting, and Twitter	Mixed platforms	General SM (unclear)	NA
Time Spent on SM	Hamilton 2020	Time spent on SNS per day	Daily diary self-report:<br>1-How much time spent on SNS (e.g., TikTok, Snapchat, Instagram) per day<br>Responses: none, <30 min, 0.5–1 hr, 1-2 hrs, 2-4 hrs, 4-6 hrs, and >6 hrs/day.		Continuous	Current	Examples: TikTok, Snapchat, and Instagram	Mixed platforms	SNS (unclear)	NA
Time Spent on SM	Kaur 2020	Time spent on SNS per day	Self-report 1-item measure:<br>1-How many hrs on an average day spent on social networking web sites like Facebook, Twitter, Instagram, etc.<br>Responses: none, <1 hr, 1−2 hrs, 3−4 hrs, 5−6 hrs, 7−8 hrs, and ≥9 hrs/day.<br>4 category variable constructed: 0 hrs, <1 hr, 1-2 hrs and ≥3 hrs/day.		Binary	Current	Examples: Facebook, Twitter, and Instagram	Mixed platforms	SNS (unclear)	NA
Time Spent on SM	Kontostoli 2020	Time spent browsing and updating SNS on a weekday	Time use diary:<br>Participants recorded their behaviour in 10-min timeslots from 4 to 4 am the next day. For each 10-min timeslot, participants indicated their main activity, selecting from a pre-specified list of 44 activities, nested within 12 categories for both weekday and weekend day.<br>Participants were categorised according to whether they did (user) or did not (non-user) report time browsing and updating social networking sites.		Binary	Current	Examples: Facebook, Twitter, and Snapchat	Mixed platforms	SNS (active and passive use)	NA

**Exposure**	**Author and year**	**Exposure definition**	**Exposure ascertainment**	**Validated/ objectively recorded**	**Exposure measure type**	**Exposure time period**	**SM platform**	***SM platform***	***SM category (active/ passive use)***	***SM content***
Time Spent on SM	Kontostoli 2020	Time spent browsing and updating SNS on a weekend day	Time use diary:<br>Participants recorded their behaviour in 10-min timeslots from 4 to 4 am the next day. For each 10-min timeslot, participants indicated their main activity, selecting from a pre-specified list of 44 activities, nested within 12 categories for both weekday and weekend day.<br>Participants were categorised according to whether they did (user) or did not (non-user) report time browsing and updating social networking sites.		Binary	Current	Examples: Facebook, Twitter, and Snapchat	Mixed platforms	SNS (active and passive use)	NA
Time Spent on SM	Larm 2017	Online social networking chatting	Self-report 2-item measure:<br>1-How often chats on online SNS such as Myspace, Facebook, and others.<br>Responses: (1) never to (7) 6-7 days/week.<br>2-Average amount of time each day chatting on online SNS.<br>Responses: (1) do not chat to (5) >5 hrs/day.<br>Summarised score from 0 to 10 derived from 2 items.		Continuous	Current	Examples: Myspace and Facebook	Mixed platforms	SNS (active use)	NA
Time Spent on SM	Larm 2019	Time spent on SM/chatting per day	Self-report 1-item measure:<br>1-How many hrs per day usually spent chatting on the internet/SM sites.<br>Responses: not using computers, <1 hr/day, 1–2 hrs/day, 2–5 hrs/day, and >5 hrs/day.<br>Variable dichotomised: ≥2 hrs/day/<2 hrs/day.		Binary	Current	NR	NR	General SM (active use)	NA
Time Spent on SM	Lee 2015	Time spent on SNS per day	Self- report 7-item measure:<br>1- SNS usage time.<br>Responses: < 30 min, 30 min- 1 hr, 1-2 hrs, 2-3 hrs, and >3 hrs/day.		Binary	Current	NR	NR	SNS (unclear)	NA
Time Spent on SM	Lee 2021	Daytime use of social networks	Objective measure:<br>Smartphone assessed usage of social networks. Time spent on each session computed as the difference between the closing and opening time. Usage sessions of <1 second were discarded.<br>Daytime usage defined as the time from waking up to 1 hr before sleep, where sleeping time was identified using accelerometer data.	Yes	Continuous	Past week	Examples: FB, Twitter, Instagram, and Weibo	Mixed platforms	SNS (unclear)	NA
Time Spent on SM	Lee 2021	Bedtime use of social networks	Objective measure:<br>Smartphone assessed usage of social networks. Time spent on each session computed as the difference between the closing and opening time. Usage sessions of <1 second were discarded.<br>Bedtime usage defined as 1 hr before sleep, where sleeping time was identified using accelerometer data.	Yes	Continuous	Past week	Examples: FB, Twitter, Instagram, and Weibo	Mixed platforms	SNS (unclear)	NA

Exposure	Author and year	Exposure definition	Exposure ascertainment	Validated/ objectively recorded	Exposure measure type	Exposure time period	SM platform	*SM platform*	*SM category (active/ passive use)*	*SM content*
Time Spent on SM	Lee 2021	Use of social networks at wakeful moment during sleep	Objective measure:<br>Smartphone assessed usage of social networks. Time spent on each session computed as the difference between the closing and opening time. Usage sessions of <1 second were discarded.	Yes	Continuous	Past week	Examples: FB, Twitter, Instagram, and Weibo	Mixed platforms	SNS (unclear)	NA
Time Spent on SM	Lipsky 2017	Time spent on social networking per day	Self-report 2-item measure:<br>1-Time spent using a computer or cell phone for chatting online, internet, emailing, texting, tweeting, or similar social networking (other than for a job or school work) during weekdays.<br>2-Time spent using a computer or cell phone for chatting online, internet, emailing, texting, tweeting, or similar social networking (other than for a job or school work) during the weekend.<br>Responses: 0.5 hrs, ~1, ~2, ~3, ~4, ~5, ~6, and ~≥7 hrs/day - converted to the number of hrs per day (ranging from 0 to 7).		Continuous	Current	NR	NR	SNS (active use)	NA
Time Spent on SM	Longobardi 2021	Time spent on SM per day	Self-report measure:<br>No information provided.		Continuous	Current	NR	NR	General SM (unclear)	NA
Time Spent on SM	Merrill 2019	Time spent on SM per day	Self-report 1-item measure:<br>1-How many hrs plays video or computer games or uses a computer for something that is not schoolwork (incl. Xbox, PlayStation, an iPod, an iPad or other tablet, a smartphone, YouTube, Facebook or other social networking tools, and the Internet) on an average school day.<br>Due to the interactive nature of the items included as “computer use,” this was relabelled “social media use” by study investigators.<br>Responses: 0 hrs per average school day, <1 hr, 1 hrs, 2 hrs, 3 hrs, 4 hrs, and ≥5 hrs/day.		Continuous	Current	Examples: Xbox, PlayStation, iPod, iPad or other tablet, smartphone, YouTube, Facebook or other social networking tools and the internet	Mixed platforms	General SM (unclear)	NA
Time Spent on SM	Michael 2016	Average time spent on SM per day	Self-report 1-item measure:<br>1-How much time spent on SM per day.<br>Responses: 0-1 hrs, 2-3 hrs, 4-5 hrs, 6-7 hrs, ≥8 hrs/day<br>3 category variable constructed: low, moderate, and high.		Categorical	Current	Examples reported by participants Facebook, 2 go, Twitter, WhatsApp, and YouTube	Mixed platforms	SNS (unclear)	NA
Time Spent on SM	Moitra 2022	Time spent using SNS on a typical weekend and weekday	Self-report 1-item measure:<br>1-How much time spent on SNS on a typical weekend and weekday.<br>Responses: mins/day		Continuous	Past week	NR	NR	SNS (unclear)	NA
Time Spent on SM	Mojica 2014	Average time spent on SNS per week	Self-report 2-item measure:<br>1-Number of days SNS used in the past week.<br>2-Number of hrs per day of SNS use.<br>Items combined to create mean hrs per week.		Continuous	Current	NR	NR	SNS (unclear)	NA

Exposure	Author and year	Exposure definition	Exposure ascertainment	Validated/ objectively recorded	Exposure measure type	Exposure time period	SM platform	*SM platform*	*SM category (active/ passive use)*	*SM content*
Time Spent on SM	Nesi 2017	Average time on Facebook per day	Self-report 1-item measure:<br>1-Average time spent on Facebook per day.<br>Responses: 7-point scale (1) <10 min to (7) ≥ 4 hrs/day.		Continuous	Current	Specified: Facebook	Facebook	SNS (unclear)	NA
Time Spent on SM	Ng Fat 2021	Time spent on SM on a normal weekday	Self-report 1-item measure:<br>1-How many hrs spent chatting or interacting with friends through social websites on a normal weekday.<br>Responses: no-profile (those not on SM sites), non-daily user, <1 hr, 1–3 hrs, ≥4 hrs/day.		Ordinal	Current	Examples: Facebook, Myspace, and Bebo	Mixed platforms	SNS (active use)	NA
Time Spent on SM	Ohannessian 2009	Time spent emailing and instant messaging (IM) on an average/typical day	Self-report 1-item measure:<br>1-How much time spent emailing/instant messaging (IM) on an average/typical day.<br>Responses: (1) none to (6) ≥4 hrs/day.<br>Variable dichotomised: high levels of email or IM use (≥1 hr/day)/low levels of email or IM use.		Binary	Current	NR	NR	SNS (active use)	NA
Time Spent on SM	Sampasa-Kanyinga 2015	Time spent on SM websites either posting or browsing per day	Self-report 1-item measure:<br>1-How many hrs a day spent on SM websites such as Facebook, Twitter, Instagram, Myspace, either posting or browsing.<br>Responses: do not use, visit these websites but not daily, <1 hr, about 1 hr, 2 hrs, 3–4 hrs, and ≥5 hrs/day.		Ordinal	Current	Examples: Facebook, Twitter, Instagram, and Myspace	Mixed platforms	SNS (active and passive use)	NA
Time Spent on SM	Sampasa-Kanyinga 2015	Time spent on SM websites either posting or browsing per day	Self-report 1-item measure:<br>1-How many hrs a day spent on SM websites such as Facebook, Twitter, Instagram, Myspace, either posting or browsing.<br>Responses: do not use, visit these websites but not daily, <1 hr, about 1 hr, 2 hrs, 3–4 hrs, and ≥5 hrs/day.		Ordinal	Current	Examples: Facebook, Twitter, Instagram, and Myspace	Mixed platforms	SNS (active and passive use)	NA
Time Spent on SM	Sampasa-Kanyinga 2016	Time spent on SM websites either posting or browsing per day	Self-report 1-item measure:<br>1-How many hrs a day spent on SM websites such as Facebook, Twitter, Instagram, Myspace, either posting or browsing.<br>Responses: daily use (< 1 hr, about 1 hr, 2 hrs, 3-4 hrs, 5- 6 hrs, and ≥7 hrs/day).<br>3 category variable constructed: infrequent or no use of SNS (use SM, but not daily; use the Internet, but never visit SNS; and do not use the Internet), regular use (daily use ≤ 2 hrs), and heavy use (3-4 hrs, 5- 6 hrs, and ≥7 hrs/day).		Ordinal	Current	Examples: Facebook, Twitter, Instagram, and Myspace	Mixed platforms	SNS (active and passive use)	NA

Exposure	Author and year	Exposure definition	Exposure ascertainment	Validated/ objectively recorded	Exposure measure type	Exposure time period	SM platform	*SM platform*	*SM category (active/ passive use)*	*SM content*
Time Spent on SM	Tao 2022	Average time on SM per week	Self-report 2-item measure:<br>1-In the past month, on average, approximately how many days in each week was time spent on SM (e.g., Instagram, Snapchat, Facebook, Tumble, Reddit)<br>Responses: 0 to 7 days/week.<br>2-In the past month, on average approximately how many hours in each day was time spent on SM (e.g., Instagram, Snapchat, Facebook, Twitter, Tumblr, Reddit).<br>Average hours of SM use per week calculated by multiplying the number of hrs of SM use a day (0-24) with days of SM use per week (0-7)		Continuous	Past month	Examples: Instagram, Snapchat, Facebook, Twitter, Tumblr, and Reddit	Mixed platforms	General SM (unclear)	NA
Time Spent on SM	Vente 2020	Time spent on SM per day	Self-report 1-item measure:<br>1-Total time spent on SM per day.<br>Variable dichotomised: >5 hrs of SM use per day/5 or less hrs of SM use.		Binary	Current	NR	NR	General SM (unclear)	NA
Time Spent on SM	Worku 2022	Stayed more than 2 hrs/day on SM	Self-report 1-item measure:<br>1-Long stay on SM.<br>Variable dichotomised: >2 / ≤ 2 hrs/day on SM		Binary	Current	NR	NR	General SM (unclear)	NA
Time Spent on SM	Sampasa-Kanyinga 2016	Time spent on SM websites either posting or browsing per day	Self-report 1-item measure:<br>1-How many hrs a day spent on SM websites such as Facebook, Twitter, Instagram, Myspace, either posting or browsing.<br>Responses: do not use, visit these websites but not daily, <1 hr, about 1 hr, 2 hrs, 3–4 hrs, and ≥5 hrs/day.		Ordinal	Current	Examples: Facebook, Twitter, Instagram, and Myspace	Mixed platforms	SNS (active and passive use)	NA
Time Spent on SM	Sampasa-Kanyinga 2018	Time spent on SM websites either posting or browsing per day	Self-report 1-item measure:<br>1-How many hrs a day spent on SM websites such as Facebook, Twitter, Instagram, Myspace, either posting or browsing.<br>Responses: < 1 hr, about 1 hr, 2 hrs, 3-4 hrs, 5- 6 hrs, ≥7 hrs/day, visit these web sites but not daily, use the Internet but never visit these web sites, and do not use the Internet.<br>5 category variable constructed:<br>Infrequent or no use of SNS (visit these web sites but not daily, use the internet but never visit these web sites, and do not use the internet), <1 hr, 1 hr, 2/hrs, and ≥3 hrs/day.		Ordinal	Current	Examples: Facebook, Twitter, Instagram, and Myspace	Mixed platforms	SNS (active and passive use)	NA

Exposure	Author and year	Exposure definition	Exposure ascertainment	Validated/ objectively recorded	Exposure measure type	Exposure time period	SM platform	*SM platform*	*SM category (active/ passive use)*	*SM content*
Time Spent on SM	Sandercock 2016	Time spent on SM on a normal day	Self-report 2-item measure:<br>1-Use of SM.<br>Responses: yes/no.<br>2- If yes, asked how long spent using SM on a normal day.<br>Responses: <30 min, 30-60 min, 60-90 min, 90 min- 2 hrs, and >2 hrs/day.		Continuous	Current	NR	NR	General SM (unclear)	NA
Time Spent on SM	Smout 2021	Time spent on SM on a typical day	Self-report measure-<br>1-How many minutes spent on Facebook, Myspace, and other social networking sites on a typical day.<br>Responses greater than 12h per day truncated to 12h		Continuous	Current	Specified: Facebook, Myspace, and other social networking sites	Mixed platforms	SNS (unclear)	NA
Time Spent on SM	Whitehill 2020	Time spent on SM per day	Self-report measure:<br>Responses: <30 min, 30-60 min, 1-2 hrs, 2-4 hrs, and ≥ 4 hrs/day.<br>No further information provided.		Ordinal	Current	NR	NR	General SM (unclear)	NA
Freq. of SM use	Anastario 2020	Freq. of use of Twitter to talk/learn about sex or any topic related to sex	Self-report 1-item measure:<br>1-How often do you use Twitter to talk or learn about sex or any topic related to sex?<br>Responses: daily, a few days a week, every few weeks, less often, never use		Continuous	Current	Specified: Twitter	Twitter	Micro-blogging (active and passive use)	NA
Freq. of SM use	Anastario 2020	Freq. of use of Snapchat to talk/learn about sex or any topic related to sex	Self-report 1-item measure:<br>1-How often do you use Snapchat to talk or learn about sex or any topic related to sex?<br>Responses: daily, a few days a week, every few weeks, less often, never use		Continuous	Current	Specified: Snapchat	Snapchat	SNS (active and passive use)	NA
Freq. of SM use	Anastario 2020	Freq. of use of Facebook to talk/learn about sex or any topic related to sex	Self-report 1-item measure:<br>1-How often do you use Facebook to talk or learn about sex or any topic related to sex?<br>Responses: daily, a few days a week, every few weeks, less often, never use		Continuous	Current	Specified: Facebook	Facebook	SNS (active and passive use)	NA
Freq. of SM use	Baker 2016	Freq. of SNS use	Self-report 1- item measure:<br>1- How often used Myspace.com, Facebook.com, chat rooms or other online social networking websites.<br>Variable dichotomised: frequent SNS users (using such sites a few times per week, each week or everyday)/infrequent SNS users (using SNS never, a few times per year or a few times per month).		Binary	Current	Specified: Myspace, Facebook, chat rooms, and other SNS	Mixed platforms	SNS (unclear)	NA
Freq. of SM use	Baldwin 2018	Freq. of watching videos on YouTube	Self-report 1-item measure:<br>1-How often watch videos on YouTube.<br>Reponses: never or rarely, less than once a week, a few times a week, once a day, a few times a day, and many times every day.<br>Variable dichotomised: at least daily users/less than daily users.		Binary	Current	Specified: YouTube	YouTube	Media-sharing (passive use)	NA

Exposure	Author and year	Exposure definition	Exposure ascertainment	Validated/ objectively recorded	Exposure measure type	Exposure time period	SM platform	*SM platform*	*SM category (active/ passive use)*	*SM content*
Freq. of SM use	Baldwin 2018	Freq. of logging in, or checking Facebook account	Self-report 1-item measure:<br>1-How often login to, or check, Facebook account.<br>Response options: never or rarely, less than once a week, a few times a week, once a day, a few times a day, and many times every day.<br>Variable dichotomised: at least daily users/less than daily users.		Binary	Current	Specified: Facebook	Facebook	SNS (passive use)	NA
Freq. of SM use	Baru 2020	Freq. of SM use	Self-report 1-item measure:<br>1-Frequency of SM use (Facebook, WhatsApp, IMO, Instagram etc)<br>Response: many times a day, several times a week, once a while		Ordinal	Current	Specified: Facebook, WhatsApp, instant messenger, and Instagram	Mixed platforms	SNS (unclear)	NA
Freq. of SM use	Baumgartner 2012	Freq. of online communication	Self-report 1-item measure:<br>1- How often use instant messaging, internet chats, and SNS.<br>Responses: (0) never to (10) every day.		Continuous	Current	Specified: Instant messaging, internet chats, and SNS	Mixed platforms	SNS (unclear)	**NA**
Freq. of SM use	Ball 2020	Freq. of using SM (status updates, uploading photos or videos)	Self-report measure:<br>1-Internet activities used during the past 7 days.<br>Response options included SM activity (status updates and uploading photos or videos).		Binary	Past week	Examples: Facebook, Twitter, Instagram, Snapchat, and YouTube	NR	General SM (active use)	NA
Freq. of SM use	Ball 2020	Freq. of online gambling	Self-report measure:<br>1-Internet activities used during the past 7 days.<br>Response options included online gambling activity.		Binary	Past week	NR	NR	Online Gambling (active use)	NA
Freq. of SM use	Boniel-Nissim 2022	Freq. of online contact with others via SM	Self-report validated questionnaire:<br>Social Media Use Intensity Scale and Social Media Disorder Scale used to categorise participants into non-active users (online contact with others not at all or at most weekly and non-problematic user) and active users (online contact with others daily but not all the time and non-problematic user)	Yes	Binary	Current	NR	NR	General SM (active and passive use)	NA
Freq. of SM use	Beebe 2004	Presence of internet chat room use	Self-report 2-item measure:<br>1- Use the internet at home.<br>2- If yes, asked what internet is used for at home.<br>Those who checked the option “chat rooms” compared with those who did not.		Binary	Current	NR	NR	SNS (active use)	NA
Freq. of SM use	Canale 2016	Freq. of using internet for leisure activities	Self-report 1-item measure:<br>1-Internet used for leisure activities (e.g., online chatting, playing online games)<br>Responses: yes/no.		Binary	Current	NR	NR	General SM (active use)	NA

Exposure	Author and year	Exposure definition	Exposure ascertainment	Validated/ objectively recorded	Exposure measure type	Exposure time period	SM platform	*SM platform*	*SM category (active/ passive use)*	*SM content*
Freq. of SM use	Canale 2016	Freq. of online gambling in past year	Self-report 1-item measure:<br>1-How many occasions (if any) participated in online gambling activities.<br>Responses: 7 options from 0 times to ≥40 times.<br>Variable dichotomised: online gambler (anyone who had participated in online gambling at least once in the past 12 months)/non-online gamblers.		Binary	Past year	NR	NR	Online Gambling (active use)	NA
Freq. of SM use	Chang 2016	Freq. of chat room use during past week	Self-report 1-item measure:<br>1-How many days during the past week used chat rooms.<br>Responses: 0 to 7 days.		Continuous	Past week	NR	NR	SNS (active use)	NA
Freq. of SM use	Chang 2016	Freq. of online game use during past week	Self-report 1-item measure:<br>1-How many days during the past week played online games.<br>Responses: 0 to 7 days.		Continuous	Past week	NR	NR	Online Gaming (active use)	NA
Freq. of SM use	Critchlow 2019	SM apps used at least weekly	Self-report measure:<br>1-Which, if any, of the following apps used at least once a week: (1) Facebook; (2) Instagram; (3) Pinterest; (4) Snapchat; (5) Spotify; (6) Tumblr; (7) Twitter; (8) WhatsApp; (9) YouTube; and (10) Other, with free text box to write in.<br>Responses: yes/no/none of the above.<br>Cumulative score computed for SM apps used at least weekly (0–10), and 3 category variable constructed: high (6 or more apps), medium (4 or 5), and low use (3 or fewer).		Ordinal	Past week	Specified: Facebook, Instagram, Pinterest, Snapchat, Spotify, Tumblr, Twitter, WhatsApp, YouTube, and other	Mixed platforms	General SM (unclear)	NA
Freq. of SM use	Dawson 2019	Freq. of Facebook use	Self-report measure assessed via The Online Behaviour Demographic Questionnaire adapted from the Pew Research Centre's Internet, Science, and Tech self-report survey.[157]<br>Responses: (1) less than once a week to (6) almost constantly.		Continuous	Current	Specified: Facebook	Facebook	SNS (unclear)	NA
Freq. of SM use	Dawson 2019	Presence of SNS use	Self-report measure assessed via The Online Behaviour Demographic Questionnaire adapted from the Pew Research Centre's Internet, Science, and Tech self-report survey.[157]<br>Responses: (0) not reported, and (1) reported.		Binary	Current	Examples: Instagram, Snapchat, Facebook, Twitter, Skype, Kik, Tumblr, Pinterest, Curious Cat, Discord, and Amino	Mixed platforms	SNS (unclear)	NA
Freq. of SM use	Dawson 2019	Presence of messaging app use	Self-report measure assessed via The Online Behaviour Demographic Questionnaire adapted from the Pew Research Centre's Internet, Science, and Tech self-report survey.[157]<br>Responses: (0) not reported, and (1) reported.		Binary	Current	Example: WhatsApp	Whats-App	SNS (unclear)	NA

Exposure	Author and year	Exposure definition	Exposure ascertainment	Validated/ objectively recorded	Exposure measure type	Exposure time period	SM platform	*SM platform*	*SM category (active/ passive use)*	*SM content*
Freq. of SM use	Dawson 2019	Presence of discussion board use	Self-report measure assessed via The Online Behaviour Demographic Questionnaire adapted from the Pew Research Centre's Internet, Science, and Tech self-report survey.[157] Responses: (0) not reported, and (1) reported.		Binary	Current	Example: Reddit	Reddit	Social News Sites (unclear)	NA
Freq. of SM use	Dawson 2019	Presence of anonymous sharing app use	Self-report measure assessed via The Online Behaviour Demographic Questionnaire adapted from the Pew Research Centre's Internet, Science, and Tech self-report survey.[157] Responses: (0) not reported, and (1) reported.		Binary	Current	Example: Whisper	Whisper	SNS (unclear)	NA
Freq. of SM use	Dawson 2019	Presence of Twitter use	Self-report measure assessed via The Online Behaviour Demographic Questionnaire adapted from the Pew Research Centre's Internet, Science, and Tech self-report survey.[157] Responses: (0) not reported, and (1) reported.		Binary	Current	Specified: Twitter	Twitter	Micro-blogging (unclear)	**NA**
Freq. of SM use	Dawson 2019	Presence of Instagram use	Self-report measure assessed via The Online Behaviour Demographic Questionnaire adapted from the Pew Research Centre's Internet, Science, and Tech self-report survey.[157] Responses: (0) not reported, and (1) reported.		Binary	Current	Specified: Instagram	Instagram	Media-sharing (unclear)	NA
Freq. of SM use	Dawson 2019	Presence of Snapchat use	Self-report measure assessed via The Online Behaviour Demographic Questionnaire adapted from the Pew Research Centre's Internet, Science, and Tech self-report survey.[157] Responses: (0) not reported, and (1) reported.		Binary	Current	Specified: Snapchat	Snapchat	SNS (unclear)	NA
Freq. of SM use	Dawson 2019	Presence of Facebook use	Self-report measure assessed via The Online Behaviour Demographic Questionnaire adapted from the Pew Research Centre's Internet, Science, and Tech self-report survey.[157] Responses: (0) not reported, and (1) reported.		Binary	Current	Specified: Facebook	Facebook	SNS (unclear)	NA
Freq. of SM use	Dawson 2019	Facebook activity: keeping up with friends (passive)	Self-report measure assessed via The Online Behaviour Demographic Questionnaire adapted from the Pew Research Centre's Internet, Science, and Tech self-report survey.[157] Facebook activity coded based on posting activity: (0) no to minimal activity, 0-25th percentile, and (3) heavy user, 75th+ percentile. Responses: (0) not reported, and (1) reported.		Binary	Current	Specified: Facebook	Facebook	SNS (passive use)	NA

Exposure	Author and year	Exposure definition	Exposure ascertainment	Validated/ objectively recorded	Exposure measure type	Exposure time period	SM platform	*SM platform*	*SM category (active/ passive use)*	*SM content*
Freq. of SM use	Dawson 2019	Facebook activity: posting on own timeline (active)	Self-report measure assessed via The Online Behaviour Demographic Questionnaire adapted from the Pew Research Centre's Internet, Science, and Tech self-report survey.[157] Facebook activity coded based on posting activity: (0) no to minimal activity, 0-25th percentile, and (3) heavy user, 75th+ percentile. Responses: (0) not reported, and (1) reported.		Binary	Current	Specified: Facebook	Facebook	SNS (active use)	NA
Freq. of SM use	Dawson 2019	Facebook activity: commenting on friend's posts (active)	Self-report measure assessed via The Online Behaviour Demographic Questionnaire adapted from the Pew Research Centre's Internet, Science, and Tech self-report survey.[157] Facebook activity coded based on posting activity: (0) no to minimal activity, 0-25th percentile, and (3) heavy user, 75th+ percentile. Responses: (0) not reported, and (1) reported.		Binary	Current	Specified: Facebook	Facebook	SNS (active use)	NA
Freq. of SM use	Dawson 2019	Facebook activity: looking at videos or news stories (passive)	Self-report measure assessed via The Online Behaviour Demographic Questionnaire adapted from the Pew Research Centre's Internet, Science, and Tech self-report survey.[157] Facebook activity coded based on posting activity: (0) no to minimal activity, 0-25th percentile, and (3) heavy user, 75th+ percentile. Responses: (0) not reported, and (1) reported.		Binary	Current	Specified: Facebook	Facebook	SNS (passive use)	NA
Freq. of SM use	Dawson 2019	Facebook interactions with friends they see daily	Self-report measure assessed via The Online Behaviour Demographic Questionnaire adapted from the Pew Research Centre's Internet, Science, and Tech self-report survey.[157] Responses: (0) not reported, and (1) reported.		Binary	Current	Specified: Facebook	Facebook	SNS (active use)	NA
Freq. of SM use	Dawson 2019	Facebook interactions with friends they see occasionally	Self-report measure assessed via The Online Behaviour Demographic Questionnaire adapted from the Pew Research Centre's Internet, Science, and Tech self-report survey.[157] Responses: (0) not reported, and (1) reported.		Binary	Current	Specified: Facebook	Facebook	SNS (active use)	NA
Freq. of SM use	Dawson 2019	Facebook interactions with online friends	Self-report measure assessed via The Online Behaviour Demographic Questionnaire adapted from the Pew Research Centre's Internet, Science, and Tech self-report survey.[157] Responses: (0) not reported, and(1) reported.		Binary	Current	Specified: Facebook	Facebook	SNS (active use)	NA
Freq. of SM use	Dawson 2019	Facebook interactions with family members	Self-report measure assessed via The Online Behaviour Demographic Questionnaire adapted from the Pew Research Centre's Internet, Science, and Tech self-report survey.[157] Responses: (0) not reported, and (1) reported.		Binary	Current	Specified: Facebook	Facebook	SNS (active use)	NA

Exposure	Author and year	Exposure definition	Exposure ascertainment	Validated/ objectively recorded	Exposure measure type	Exposure time period	SM platform	*SM platform*	*SM category (active/ passive use)*	*SM content*
Freq. of SM use	Dawson 2019	Number of participant posts on Facebook (posted by participant)	Observationally coded measure, coded using Mikami and Szwedo's Facebook Coding Manual.[158]<br>Facebook profile coded over 2-month period to obtain total number of participant posted posts.	Yes	Continuous	Current	Specified: Facebook	Facebook	SNS (active use)	NA
Freq. of SM use	Dawson 2019	% of participant posts that were shared external material on Facebook	Observationally coded measure, coded using Mikami and Szwedo's Facebook Coding Manual.[158]<br>Facebook profile coded over 2-month period to obtain proportion of total participant posts that were "shared" external material (i.e., memes or web links copied and reposted from another location; types: emotional, animal related, intended humour, sports, motivational, news/politics, and music).	Yes	Continuous	Current	Specified: Facebook	Facebook	SNS (active use)	NA
Freq. of SM use	Dawson 2019	% of participant posts sharing accomplishments on Facebook	Observationally coded measure, coded using Mikami and Szwedo's Facebook Coding Manual.[158]<br>Facebook profile coded over 2-month period to obtain proportion of total participant posts sharing accomplishments (i.e., something that typically infers pride in some skillset or effort).	Yes	Continuous	Current	Specified: Facebook	Facebook	SNS (active use)	NA
Freq. of SM use	Dawson 2019	% of participant posts illustrating connection on Facebook	Observationally coded measure, coded using Mikami and Szwedo's Facebook Coding Manual.[158]<br>Facebook profile coded over 2-month period to obtain proportion of total participant posts illustrating connection with friends (e.g., meetings withing the year, specific plans for future meetings, or shared information (such as inside jokes).	Yes	Continuous	Current	Specified: Facebook	Facebook	SNS (active use)	NA
Freq. of SM use	Dawson 2019	% of participants posts sharing support on Facebook	Observationally coded measure, coded using Mikami and Szwedo's Facebook Coding Manual.[158]<br>Facebook profile coded over 2-month period to obtain proportion of total participant posts containing emotional support (i.e., posts offering encouragement, validation, compliments, or empathy).	Yes	Continuous	Current	Specified: Facebook	Facebook	SNS (active use)	NA
Freq. of SM use	De Looze 2019	Freq. of electronic media communication with friends	Self-report 3-item measure:<br>Asked how often:<br>1- contacted friends using texting/SMS;<br>2- actively contacted friends using instant messaging (e.g., Facebook chat);<br>3- contacted friends using other SM, such as Facebook (posting on wall, not chat), Myspace, Twitter, apps (e.g., Instagram), games (e.g., Xbox), YouTube.<br>Responses: hardly ever or never, less than weekly, weekly, and daily.<br>Variable dichotomised: (0) less than daily/(1) daily.		Binary	Current	Examples: Blackberry Messaging, Facebook chat, Facebook, Myspace, Twitter, Instagram, Xbox, and YouTube	Mixed platforms	SNS (active use)	NA

Exposure	Author and year	Exposure definition	Exposure ascertainment	Validated/ objectively recorded	Exposure measure type	Exposure time period	SM platform	*SM platform*	*SM category (active/ passive use)*	*SM content*
Freq. of SM use	Elton-Marshall 2016	Freq. of playing free simulated gambling games on Facebook	<u>Self-report 1-item measure:</u><br>1-Participation in any online gambling games on Facebook for fun (no money).<br>Responses: not in the past 3 months, about once per month, 2–3 times per month, about once per week, 2–6 times per week, and daily.<br>Variable dichotomised: at least monthly but less than weekly (about once per month or 2–3 times per month)/at least weekly (about once per week, 2–6 times per week, or daily).<br>Overall prevalence based on any participation (indicated about once per month or more frequent).		Binary	Past 3 months	Specified: Facebook	Facebook	Online Gambling (active use)	NA
Freq. of SM use	Elton-Marshall 2016	Freq. of online gambling participation	<u>Self-report measure:</u><br>Online gamblers: respondents who indicated that they had gambled money or something of value in the past for any of 3 online gambling activities: internet poker, sports pools online, and slot machines online.<br>Land-based gamblers: respondents who had gambled money or something of value in the past 3 months but had not participated in any of the online gambling activities.<br>No further information reported.		Binary	Past 3 months	NR	NR	Online Gambling (active use)	NA
Freq. of SM use	Erreygers 2017	Freq. of online gaming	<u>Self-report 2-item measure:</u><br>1-How often used online gaming (playing with others).<br>2-How often used online gaming (playing alone or against the computer).<br>Responses: never, just a few times, 1-4 times per month, almost every day, multiple times per day, I don't know and not applicable.<br>Confirmatory factor analyses used to generate online gaming factor.		Continuous	Past 6 months	NR	NR	Online Gaming (active use)	NA
Freq. of SM use	Floros 2013	Freq. of using SNS	<u>Self-report measure:</u><br>Internet activities measured on Likert scale for frequency.<br>No further information reported.		Continuous	Past year	NR	NR	SNS (unclear)	NA
Freq. of SM use	Floros 2013	Freq. of online discussions in real time (IRC, MSN etc)	<u>Self-report measure:</u><br>Internet activities measured on Likert scale for frequency.<br>No further information reported.		Continuous	Past year	NR	NR	SNS (active use)	NA
Freq. of SM use	Floros 2013	Freq. of online discussions with posts on boards, forums	<u>Self-report measure:</u><br>Internet activities measured on Likert scale for frequency.<br>No further information reported.		Continuous	Past year	NR	NR	Blogs & Forums (active use)	NA

**Exposure**	**Author and year**	**Exposure definition**	**Exposure ascertainment**	**Validated/ objectively recorded**	**Exposure measure type**	**Exposure time period**	**SM platform**	***SM platform***	***SM category (active/ passive use)***	***SM content***
Freq. of SM use	Gregg 2018	Freq. of electronic communication	Self-report 3-item measure:<br>1-How long used SM on a normal school day.<br>Responses: from (0) never, to (7) >4 hrs/day.<br>2-How long used SM on a normal non-school day.<br>Responses: from (0) never, to (7) >4 hrs/day.<br>3- How many text messages sent on an average day.<br>Responses: from (0) I do not text to (6) >300.<br>Responses added to produce overall estimate of SM use. Higher scores indicated more frequent use of SM.		Continuous	Current	NR	NR	General SM (unclear)	NA
Freq. of SM use	Gunnlaugsson 2020	Freq. of SM use	Self-report 1-item validated measure:<br>1-Experience of communicating in the last 12 months on SM with friends, family, and people the respondent would like to know.<br>Responses: everyday, 2-3 times a week, every week, less than monthly, and never.	Yes	Binary	Past year	NR	NR	General SM (active use)	NA
Freq. of SM use	Hayer 2018	Freq. of participation in any simulated gambling on social networks	Self-report 1-item measure:<br>1-Frequency of participation in any simulated gambling on social networks.<br>Responses (5 options): from not at all to more than 8 times a month.<br>Variable dichotomised: participation/no participation.		Binary	Past year	NR	NR	Online Gambling (active use)	NA
Freq. of SM use	Hayer 2018	Freq. of participation in any simulated gambling via apps	Self-report 1-item measure:<br>1- Frequency of participation in any simulated gambling via apps.<br>Responses (5 options): from not at all to more than 8 times a month.<br>Variable dichotomised: participation/no participation.		Binary	Past year	NR	NR	Online Gambling (active use)	NA
Freq. of SM use	Hayer 2018	Freq. of participation in simulated gambling from home on social networks	Self-report 1-item measure:<br>1-Frequency of participation in simulated gambling from home on social networks.<br>Responses (5 options): from not at all to more than 8 times a month.<br>Variable dichotomised: participation/no participation.		Binary	Past year	NR	NR	Online Gambling (active use)	NA
Freq. of SM use	Hayer 2018	Freq. of participation in simulated gambling from home via apps	Self-report 1-item measure:<br>1-Frequency of participation in simulated gambling from home via apps.<br>Responses (5 options): from not at all to more than 8 times a month.<br>Variable dichotomised: participation/no participation.		Binary	Past year	NR	NR	Online Gambling (active use)	NA
Freq. of SM use	Hayer 2018	Freq. of participation in simulated gambling when out and about on social networks	Self-report 1-item measure:<br>1-Frequency of participation in simulated gambling when out and about on social networks.<br>Responses (5 options): from not at all to more than 8 times a month.<br>Variable dichotomised: participation/no participation.		Binary	Past year	NR	NR	Online Gambling (active use)	NA

**Exposure**	**Author and year**	**Exposure definition**	**Exposure ascertainment**	**Validated/ objectively recorded**	**Exposure measure type**	**Exposure time period**	**SM platform**	***SM platform***	***SM category (active/ passive use)***	***SM content***
Freq. of SM use	Hayer 2018	Freq. of participation in simulated gambling when out and about via apps	Self-report 1-item measure:<br>1-Frequency of participation in simulated gambling when out and about via apps.<br>Responses (5 options): from not at all to more than 8 times a month.<br>Variable dichotomised: participation/no participation.		Binary	Past year	NR	NR	Online Gambling (active use)	NA
Freq. of SM use	Holtz 2011	Freq. of online gaming	Self-report 3-item measure:<br>Frequency of playing:<br>1-first person shooters online;<br>2-playing online role-playing games, and;<br>3-playing other games.<br>Responses: 1 (never) to 5 (very often).<br>Latent factor generated.		Continuous	Current	NR	NR	Online Gaming (active use)	NA
Freq. of SM use	Holtz 2011	Freq. of communicational internet use (e.g., chatrooms, social platforms like Myspace)	Self-report 3-item measure:<br>Frequency of use of:<br>1-email;<br>2-use of chatrooms;<br>3-use of social platforms like Myspace.<br>Responses: (1) never to (5) very often.<br>Latent factor created.		Continuous	Current	Example: Myspace	Myspace	SNS (active and passive use)	NA
Freq. of SM use	Hryhorczuk 2019	Freq. of SM use	Self-report measure:<br>1-How free time spent.<br>Adolescents who responded they use SM frequently or sometimes compared to those who said that they never use SM.		Binary	Current	NR	NR	General SM (unclear)	NA
Freq. of SM use	Huang 2012	Freq. of social internet activity (online gaming, chatting with real friends, chatting with online friends)	Self-report measure:<br>1-How often conducted the following computer and internet-based activities: online games, chatting with friends in real daily life and chatted with friends met online.<br>The average of the items played online games, chatting with friends in real daily life, and chatted with friends met online taken and loaded to create factor 'Social internet activity' and appropriate scale.		Continuous	Past week	NR	NR	SNS (active use)	NA
Freq. of SM use	Huang 2014	Freq. of Facebook use	Self-report 1-item measure:<br>1-How frequently SNS Facebook visited.<br>Responses: never, rarely (about once a month or less), occasionally (about once a week or less), frequently (about once every 2-3 days), and very frequently (about once a day or more).		Ordinal	Past month	Specified: Facebook	Facebook	SNS (unclear)	NA

Exposure	Author and year	Exposure definition	Exposure ascertainment	Validated/ objectively recorded	Exposure measure type	Exposure time period	SM platform	*SM platform*	*SM category (active/ passive use)*	*SM content*
Freq. of SM use	Huang 2014	Freq. of Myspace use	Self-report 1-item measure:<br>1-How frequently SNS Myspace visited.<br>Responses: never, rarely (about once a month or less), occasionally (about once a week or less), frequently (about once every 2-3 days), and very frequently (about once a day or more).		Ordinal	Past month	Specified: Myspace	Myspace	SNS (unclear)	NA
Freq. of SM use	Jeong 2022	Freq. of SM use	Self-report 1-item measure:<br>1-SM usage frequency.<br>Response: not at all, sometimes, and often<br>Variable dichotomised: SM users (sometimes, often)/non-SM user (not at all).		Binary	Current	NR	NR	General SM (unclear)	NA
Freq. of SM use	Jiang 2018	Freq. of online gaming	Exposure ascertained via clinical records:<br>5 items assessed- online gaming history, frequency of online gaming, degree of involvement, number of gaming buddies, and amount of time of online gaming on average.<br>Responses: 5-point Likert scale.	Yes	Continuous	Current	NR	NR	Online Gaming (active use)	NA
Freq. of SM use	Kaufman 2014	Freq. of SM use	Self-report 1-item measure:<br>1-How often used Facebook, Mxit, or other social networks.<br>Responses: every day, every 2–3 days, once a week, once every 2–3 weeks, never and almost never.<br>Variable dichotomised: SM used every day/ did not use SM every day.		Binary	Current	Examples: Facebook, Mxit, and other social networks	Mixed platforms	SNS (unclear)	NA
Freq. of SM use	Kelleghan 2020	Freq. of SM posting (posting photos, video or statuses and sharing others content)	Self-report 1-item measure:<br>1-How often posted own photographs, images, videos, status updates, or blogs over past week.<br>Responses: 0 times, 1-2 times per week, 1-2 times per day, and many times per day.<br>Variable dichotomised: high frequency use (multiple times per day)/less frequent use (0 times, 1-2 times per week, 1-2 times per day)		Binary	Current	NR	NR	General SM (active use)	NA
Freq. of SM use	King 2014	Ever use of simulated gambling via SNS applications	Self-report 1-item measure:<br>1-Ever tried simulated gambling via SNS applications (Facebook). Responses: yes/no.		Binary	Ever	Specified: Facebook	Facebook	Online Gambling (active use)	NA
Freq. of SM use	Ko 2009	Ever online chatting	Self-report 1-item measure:<br>1-Ever participated in online chatting.<br>No further information provided.		Binary	Ever	NR	NR	SNS (active use)	NA
Freq. of SM use	Ko 2009	Ever online gaming	Self-report 1-item measure:<br>1-Ever participated in online gaming.<br>No further information provided.		Binary	Ever	NR	NR	Online Gaming (active use)	NA
Freq. of SM use	Ko 2009	Ever online gambling	Self-report 1-item measure:<br>1-Ever participated in online gambling.<br>No further information provided.		Binary	Ever	NR	NR	Online Gambling (active use)	NA

Exposure	Author and year	Exposure definition	Exposure ascertainment	Validated/ objectively recorded	Exposure measure type	Exposure time period	SM platform	*SM platform*	*SM category (active/ passive use)*	*SM content*
Freq. of SM use	Koutamanis 2015	Freq. of online social exploration on SNS	<u>Self-report 4-item measure:</u><br>How often following things done on SNS:<br>1-invited someone to become friends;<br>2-commented on a message or picture of someone they don't know that well;<br>3-sent a message to someone they don't know that well;<br>4-asked someone whether they want to do something fun with them.<br>Responses: never, almost never, sometimes, often, and very often.<br>Average of 4 items used to create composite scale.		Continuous	Current	NR	NR	SNS (active use)	NA
Freq. of SM use	Kwon 2022	Freq. of SNS use	<u>Self-report measure:</u><br>1-Frequency of smartphone use at weekday and weekend day, and specific content accessed (e.g., SNS)		Continuous	Past month	Examples: Blogs, Instagram, Twitter, and Facebook	Mixed platforms	SNS (unclear)	NA
Freq. of SM use	Landry 2013	Freq. of logging into SM sites	<u>Self-report measure:</u><br>If internet used, how often, and if had accounts on any of the following SM sites: Facebook, Myspace, Twitter, Yahoo, YouTube, My Yearbook, Tumblr, Google buzz, Flickr, Ustream, and Other.<br>A count variable created for the number of SM accounts. If participant had an account, they were asked about their frequency of internet use and logging in to SM sites.<br>Responses: several times a day, about once a day, 3 to 5 days week, 1 to 2 days a week, every few weeks, and less often.<br>Frequency of logging in to SM sites dichotomised: daily log-in/less frequent log-in.		Binary	Current	Specified: Facebook, Myspace, Twitter, Yahoo, YouTube, My Yearbook, Tumblr, Google buzz, Flickr, Ustream, and other	Mixed platforms	SNS (unclear)	NA
Freq. of SM use	Lee 2019	Freq. of visiting social networking account	<u>Self-report 1-item measure:</u><br>1-How often visited Facebook, Google Plus, Myspace, Twitter, or other social networking account.<br>Responses: no account/use, monthly or less, weekly, and daily.		Ordinal	Current	Examples: Facebook, Google plus, Myspace, and Twitter	Mixed platforms	SNS (unclear)	NA
Freq. of SM use	Lee 2021	Freq. of Facebook use	<u>Self-report 1-item measure:</u><br>1-How often Facebook visited.<br>Responses: never, every few months, every few weeks, 1-2 days per week, 3-5 days per week, once per day, and several times per day.<br>3 category variable constructed: never/non-daily (every few months, every few weeks, 1-2 days per week, and 3-5 days per week)/daily (once per day, and several times per day)		Ordinal	Current	Specified: Facebook	Facebook	SNS (unclear)	NA

Exposure	Author and year	Exposure definition	Exposure ascertainment	Validated/ objectively recorded	Exposure measure type	Exposure time period	SM platform	*SM platform*	*SM category (active/ passive use)*	*SM content*
Freq. of SM use	Lee 2021	Freq. of Instagram use	Self-report 1-item measure:<br>1-How often Instagram visited.<br>Responses: never, every few months, every few weeks, 1-2 days per week, 3-5 days per week, once per day, and several times per day.<br>3 category variable constructed: never/non-daily (every few months, every few weeks, 1-2 days per week, and 3-5 days per week)/daily (once per day, and several times per day)		Ordinal	Current	Specified: Instagram	Instagram	Media-sharing (unclear)	NA
Freq. of SM use	Lee 2021	Freq. of Twitter use	Self-report 1-item measure:<br>1-How often Twitter visited.<br>Responses: never, every few months, every few weeks, 1-2 days per week, 3-5 days per week, once per day, and several times per day.<br>3 category variable constructed: never/non-daily (every few months, every few weeks, 1-2 days per week, and 3-5 days per week)/daily (once per day, and several times per day)		Ordinal	Current	Specified: Twitter	Twitter	Micro-blogging (unclear)	NA
Freq. of SM use	Lee 2021	Freq. of Snapchat use	Self-report 1-item measure:<br>1-How often Snapchat visited.<br>Responses: never, every few months, every few weeks, 1-2 days per week, 3-5 days per week, once per day, and several times per day.<br>3 category variable constructed: never/non-daily (every few months, every few weeks, 1-2 days per week, and 3-5 days per week)/daily (once per day, and several times per day)		Ordinal	Current	Specified: Snapchat	Snapchat	SNS (unclear)	NA
Freq. of SM use	McClure 2020	Freq. of SM use	Self-report 1-item measure:<br>1-How often SM used.<br>Responses: never, rarely, once in a while, about once a day, and many times a day.<br>Mean score calculated.		Continuous	Current	NR	NR	General SM (unclear)	NA
Freq. of SM use	Molla-Esparza 2021	Freq. of using SM platforms	Self-report 1-item measure:<br>1-How often SM used.<br>Responses: daily, several days a week, several days a month, almost never, never<br>Variable dichotomised: low usage frequency (never, almost never, and several days a month)/high usage frequency (several days a week and daily)		Binary	Current	NR	NR	General SM (unclear)	NA

Exposure	Author and year	Exposure definition	Exposure ascertainment	Validated/ objectively recorded	Exposure measure type	Exposure time period	SM platform	*SM platform*	*SM category (active/ passive use)*	*SM content*
Freq. of SM use	Nesi 2019	Posted photos with peers on Instagram	Observationally coded measure:<br>1-Photos posted with peers, where both participant and same-age peers depicted.<br>Sum of the number of photos with peers posted during the 3-month coding period taken.	Yes	Continuous	During coding period	Specified: Instagram	Instagram	Media-sharing (active use)	NA
Freq. of SM use	Nesi 2019	Freq. of daily SM use	Self-report 1-item measure:<br>1-Average daily frequency of SM use defined as any website/app that involves social interaction, i.e., Facebook, Instagram, Tumblr, Snapchat.<br>Responses: (0) I don't use this to (6) ≥5 hrs/day.		Continuous	Current	Examples: texting, Facebook, Instagram, and Snapchat	Mixed platforms	SNS (active use)	NA
Freq. of SM use	Nesi 2019	Posted selfies on Instagram	Observationally coded measure:<br>1-Selfies, or photos of the participant alone.<br>Sum of the number of selfies posted during the 3-month coding period taken.	Yes	Continuous	During coding period	Specified: Instagram	Instagram	Media-sharing (active use)	NA
Freq. of SM use	Pegg 2018	Freq. of SNS use (intensity)	Self-report 1-item measure:<br>1-How many hrs per week spent on SNS.<br>Responses: 0 hrs/week to ≥30 hrs/week.		Continuous	Current	NR	NR	SNS (unclear)	NA
Freq. of SM use	Prince 2021	Freq. of Snapchat use	Self-report 1-item measure:<br>1-How often Snapchat used.<br>Responses: never, rarely, sometimes, and often<br>Variable dichotomised: sometimes/often.		Binary	Current	Specified: Snapchat	Snapchat	SNS (unclear)	NA
Freq. of SM use	Riehm 2021	Freq. of checking SM sites	Self-report 1-item measure:<br>1-How often checked SM sites.<br>Responses: none, 1 to 2 times per week, 1 to 2 times per day, and many times per day.<br>Variable dichotomised: high frequency engagement (many times per day)/lower frequency engagement		Binary	Current	Examples: Facebook, Twitter, and Instagram	NR	General SM (passive use)	NA
Freq. of SM use	Riehm 2021	Freq. of posting own photos, images, videos, status updates, or blogs on SM	Self-report 1-item measure:<br>1-How often posted own photos, images, videos, status updates, or blogs on SM.<br>Responses: none, 1 to 2 times per week, 1 to 2 times per day, and many times per day.<br>Variable dichotomised: high frequency engagement (many times per day)/lower frequency engagement		Binary	Current	NR	NR	General SM (active use)	NA
Freq. of SM use	Riehm 2021	Freq. of liking or commenting on other people's statuses, wall posts, pictures, etc on SM	Self-report 1-item measure:<br>1-How often liked or commented on other people's statuses, wall posts, pictures, etc on SM.<br>Responses: none, 1 to 2 times per week, 1 to 2 times per day, and many times per day.<br>Variable dichotomised: high frequency engagement (many times per day)/lower frequency engagement		Binary	Current	NR	NR	General SM (active use)	NA

**Exposure**	**Author and year**	**Exposure definition**	**Exposure ascertainment**	**Validated/ objectively recorded**	**Exposure measure type**	**Exposure time period**	**SM platform**	***SM platform***	***SM category (active/ passive use)***	***SM content***
Freq. of SM use	Riehm 2021	Freq. of sharing other people's photos, images, videos, status updates, blogs, articles, news, or websites on SM	Self-report 1-item measure:<br>1-How often shared other people's photos, images, videos, status updates, blogs, articles, news, or websites on SM.<br>Responses: none, 1 to 2 times per week, 1 to 2 times per day, and many times per day.<br>Variable dichotomised: high frequency engagement (many times per day)/lower frequency engagement		Binary	Current	NR	NR	General SM (active use)	NA
Freq. of SM use	Romo 2017	Freq. of SM app use	Self-report 1-item measure via validated questionnaire:<br>1-How often visited/used apps per day or week.<br>Variable dichotomised: frequent use of visiting online SM apps (>10 times per day)/infrequent use (≤10 times per day).	Yes	Binary	Current	NR	NR	General SM (unclear)	NA
Freq. of SM use	Romo 2017	Freq. of SNS use	Self-report 1-item measure via validated questionnaire:<br>1-How often SNS visited/used per day or week.<br>Variable dichotomised: frequent use of visiting online SNS (>10 times per day)/infrequent use (≤ 10 times per day).	Yes	Binary	Current	NR	NR	SNS (unclear)	NA
Freq. of SM use	Rutter 2021	Freq. of SM use (checking and posting)	Self-report measure:<br>Panel of surveys assessing SM use and SM rules.<br>No further information reported.		Continuous	Current	NR	NR	General SM (unclear)	NA
Freq. of SM use	Savolainen 2020	Freq. of Facebook use	Self-report 1-item measure via validated questionnaire:<br>1-How often Facebook used.<br>Responses: I do not use, seldom, daily, and several times a day.<br>Variable dichotomised: daily user/non-daily user.	Yes	Binary	Current	Specified: Facebook	Facebook	SNS (unclear)	NA
Freq. of SM use	Savolainen 2020	Freq. of YouTube use	Self-report 1-item measure via validated questionnaire:<br>1-How often YouTube used.<br>Responses: I do not use, seldom, daily, and several times a day.<br>Variable dichotomised: daily user vs non-daily user.	Yes	Binary	Current	Specified: YouTube	YouTube	Media-sharing (unclear)	NA
Freq. of SM use	Savolainen 2020	Freq. of Twitter use	Self-report 1-item measure via validated questionnaire:<br>1-How often Twitter used.<br>Responses: I do not use, seldom, daily, and several times a day.<br>Variable dichotomised: daily user vs non-daily user.	Yes	Binary	Current	Specified: Twitter	Twitter	Micro-blogging (unclear)	NA
Freq. of SM use	Savolainen 2020	Freq. of Instagram use	Self-report 1-item measure via validated questionnaire:<br>1-How often Instagram used.<br>Responses: I do not use, seldom, daily, and several times a day.<br>Variable dichotomised: daily user vs non-daily user.	Yes	Binary	Current	Specified: Instagram	Instagram	Media-sharing (unclear)	NA

**Exposure**	**Author and year**	**Exposure definition**	**Exposure ascertainment**	**Validated/ objectively recorded**	**Exposure measure type**	**Exposure time period**	**SM platform**	***SM platform***	***SM category (active/ passive use)***	***SM content***
Freq. of SM use	Savolainen 2020	Freq. of instant messaging	Self-report 1-item measure via validated questionnaire:<br>1-How often instant messaging used (e.g., WhatsApp/Snapchat).<br>Responses: I do not use, seldom, daily, and several times a day.<br>Variable dichotomised: daily user vs non-daily user.	Yes	Binary	Current	Specified: Instant messaging.<br>Examples: WhatsApp, and Snapchat	Instant Messaging	SNS (active use)	NA
Freq. of SM use	Self-Brown 2018	Presence of SM use	Self-report 1-item measure via validated questionnaire:<br>1-Used any type of SM (e.g., Twitter, Facebook).<br>Responses: yes/no.	Yes	Binary	Current	Examples: Twitter and Facebook	Mixed platforms	General SM (unclear)	NA
Freq. of SM use	Shimoga 2019	Freq. of SM use	Self-report 1-item measure:<br>1-How often social networking websites like Facebook, Twitter, Instagram, etc visited.<br>Responses: never, a few times a year, one to two times a month, once a week, and every day.		Ordinal	Current	Examples: Twitter, Facebook, and Instagram	Mixed platforms	SNS (unclear)	NA
Freq. of SM use	Soneji 2018	Freq. of social networking account use	Self-report 1-item measure:<br>1-How often Facebook, Google Plus, Myspace, Twitter, or other social networking account visited.<br>Responses: several times a day, daily, weekly, monthly, and less.		Ordinal	Current	Examples: Facebook, Google plus, Myspace, Twitter, and other	Mixed platforms	SNS (unclear)	NA
Freq. of SM use	Svensson 2020	Freq. of posting information on Facebook, Instagram, Snapchat, or other SM	Self-report 1-item measure:<br>1-How often use a computer, mobile phone, or tablet to post information about yourself on Facebook, Instagram, Snapchat, or other SM.<br>Responses: never, once a month, about once a week, several times a day, everyday		Continuous	Current	Examples: Facebook, Instagram, Snapchat, or other SM	Mixed platforms	General SM (active use)	NA
Freq. of SM use	Svensson 2020	Freq. of staying in contact with and staying informed about friends via Facebook, Instagram or similar	Self-report 1-item measure:<br>1-How often use a computer, mobile phone, or tablet to stay in contact with and stay informed about friends via Facebook, Instagram or similar.<br>Responses: never, once a month, about once a week, several times a day, everyday		Continuous	Current	Examples: Facebook, Instagram, or similar	Mixed platforms	General SM (active and passive use)	NA
Freq. of SM use	Tsitsika 2009	Ever accessing the internet to visit chat rooms	Self-report measure:<br>Primary objects of interest via internet.<br>No further info provided		Binary	Current	NR	NR	SNS (active use)	NA
Freq. of SM use	Tsitsika 2011	Presence of internet chat room use	Self-report measure:<br>Use of internet chat rooms.<br>No further information provided.		Binary	Current	NR	NR	SNS (active use)	NA
Freq. of SM use	Vandenbosch 2016	Freq. of chat room use	Self-report 1-item measure:<br>1-How often usually visited chat rooms.<br>Responses: (1) never to (8) all day long.<br>3 category variable constructed: non-users, infrequent (less than once a month, and frequent (monthly to daily).		Ordinal	Current	NR	NR	SNS (active use)	NA

**Exposure**	**Author and year**	**Exposure definition**	**Exposure ascertainment**	**Validated/ objectively recorded**	**Exposure measure type**	**Exposure time period**	**SM platform**	***SM platform***	***SM category (active/ passive use)***	***SM content***
Freq. of SM use	Vannucci 2019	Freq. of SM use	Self-report measure via Technology Use Questionnaire:<br>1-How often used a range of SM platforms on a typical day (discussion boards, FB, Google +, Instagram, Pin Boards, Snapchat, Tumblr, Twitter).<br>Responses: (0) never to (8) almost constantly.<br>Total number of platforms used calculated by coding each platform as either (0) never used and (1) used at least once or more, and then summing usage scores.<br>2-How much time, overall, they spent using SM platforms on a typical day (in hrs).<br>Latent profile analyses used to identify latent subgroups of SM: high SM use (frequent daily overall use across platforms), high Instagram/Snapchat use (hourly use of Instagram + Snapchat use only, with low use of all other SM platforms), and low SM use (less than once daily use of all SM platforms).		Categorical	Current	Examples: Discussion boards, FB, Google +, Instagram, Pin Boards, Snapchat, Tumblr, and Twitter	Mixed platforms	General SM (unclear)	NA
Freq. of SM use	Vannucci 2019	Freq. of Google+ use	Self-report 1-item measure:<br>1-How often used Google + on a typical day.<br>Responses: never, less than once a week, once a week, several times a week, once a day, several times a day, once an hr, several times an hr, and almost constantly.		Continuous	Current	Specified: Google +	Google+	SNS (unclear)	NA
Freq. of SM use	Vannucci 2019	Freq. of use of pin boards	Self-report 1-item measure:<br>1-How often used pin boards on a typical day.<br>Responses: never, less than once a week, once a week, several times a week, once a day, several times a day, once an hr, several times an hr, and almost constantly.		Continuous	Current	Specified: Pin board	Discussion & Pin Boards	Blogs & Forums (unclear)	NA
Freq. of SM use	Vannucci 2019	Freq. of Facebook use	Self-report 1-item measure:<br>1-How often used Facebook on a typical day.<br>Responses: never, less than once a week, once a week, several times a week, once a day, several times a day, once an hr, several times an hr, and almost constantly.		Continuous	Current	Specified: Facebook	Facebook	SNS (unclear)	NA
Freq. of SM use	Vannucci 2019	Freq. of Instagram use	Self-report 1-item measure:<br>1- How often used Instagram on a typical day.<br>Responses: never, less than once a week, once a week, several times a week, once a day, several times a day, once an hr, several times an hr, and almost constantly.		Continuous	Current	Specified: Instagram	Instagram	SNS (unclear)	NA
Freq. of SM use	Vannucci 2019	Freq. of Snapchat use	Self-report 1-item measure:<br>1-How often used Snapchat on a typical day.<br>Responses: never, less than once a week, once a week, several times a week, once a day, several times a day, once an hr, several times an hr, and almost constantly.		Continuous	Current	Specified: Snapchat	Snapchat	SNS (unclear)	NA

Exposure	Author and year	Exposure definition	Exposure ascertainment	Validated/ objectively recorded	Exposure measure type	Exposure time period	SM platform	*SM platform*	*SM category (active/ passive use)*	*SM content*
Freq. of SM use	Vannucci 2019	Freq. of Twitter use	Self-report 1-item measure:<br>1-How often used Twitter on a typical day.<br>Responses: never, less than once a week, once a week, several times a week, once a day, several times a day, once an hr, several times an hr, and almost constantly.		Continuous	Current	Specified: Twitter	Twitter	Micro-blogging (unclear)	NA
Freq. of SM use	Vannucci 2019	Freq. of Tumblr use	Self-report 1-item measure:<br>1-How often used Tumblr on a typical day.<br>Responses: never, less than once a week, once a week, several times a week, once a day, several times a day, once an hr, several times an hr, and almost constantly.		Continuous	Current	Specified: Tumblr	Tumblr	Micro-blogging (unclear)	NA
Freq. of SM use	Vannucci 2019	Freq. of use of discussion boards	Self-report 1-item measure:<br>1-How often used discussion boards on a typical day.<br>Responses: never, less than once a week, once a week, several times a week, once a day, several times a day, once an hr, several times an hr, and almost constantly.		Continuous	Current	Specified: Discussion boards	Discussion & Pin Boards	Blogs & forums (unclear)	NA
Freq. of SM use	Vazquez-Nava 2020	Presence of use of social networks (WhatsApp/ Facebook)	Self- report measure via validated questionnaire:<br>1-Use of online messaging platforms such as WhatsApp or Facebook to communicate with their friends and other people.	Yes	Binary	Current	Specified: WhatsApp and Facebook	Mixed platforms	SNS (active use)	NA
Freq. of SM use	Vente 2020	Any SM per day	Self-report measure:<br>Total time spent on SM per day, and number and type of SM application used.		Binary	Current	NR	NR	General SM (unclear)	NA
Freq. of SM use	Vente 2020	Use of ≥4 SM applications per day	Self-report measure:<br>Total time spent on SM per day, and number and type of SM application used.		Binary	Current	NR	NR	General SM (unclear)	NA
Freq. of SM use	Wana 2019	Presence of SM use	Self-report measure:<br>If SM user, what platforms used, frequency of SM use, and the purpose of using SM.		Binary	Current	Examples: Facebook, Viber, WhatsApp, YouTube, and Instagram	Mixed platforms	SNS (unclear)	NA
Freq. of SM use	Ward 2022	Freq. of Facebook use per day	Self-report 1-item measure:<br>1-How many times per day Facebook checked.<br>Responses: 0 to 7+ times.		Continuous	Current	Specified: Facebook	Facebook	SNS (unclear)	NA
Freq. of SM use	Ward 2022	Freq. of Snapchat use per day	Self-report 1-item measure:<br>1-How many times per day Snapchat checked.<br>Responses: 0 to 7+ times.		Continuous	Current	Specified: Snapchat	Snapchat	SNS (unclear)	NA
Freq. of SM use	Ward 2022	Freq. of Instagram use per day	Self-report 1-item measure:<br>1-How many times per day Instagram checked.<br>Responses: 0 to 7+ times.		Continuous	Current	Specified: Instagram	Instagram	Media-sharing (unclear)	NA
Freq. of SM use	Whitehill 2020	Freq. of SM use	Self-report 1-item measure:<br>Responses: never, monthly, a few times a month, weekly, a few times a week, once a day, and more than once a day. No further information provided.		Ordinal	Current	NR	NR	General SM (unclear)	NA

Exposure	Author and year	Exposure definition	Exposure ascertainment	Validated/ objectively recorded	Exposure measure type	Exposure time period	SM platform	*SM platform*	*SM category (active/ passive use)*	*SM content*
Freq. of SM use	Whitehill 2020	Presence of Facebook use	No information reported.		Binary	Current	Specified: Facebook	Facebook	SNS (unclear)	NA
Freq. of SM use	Whitehill 2020	Presence of Twitter use	No information reported.		Binary	Current	Specified: Twitter	Twitter	Micro-blogging (unclear)	NA
Freq. of SM use	Whitehill 2020	Presence of Instagram use	No information reported.		Binary	Current	Specified: Instagram	Instagram	Media-sharing (unclear)	NA
Freq. of SM use	Widman 2014	Used technology based sexual communication to communicate with dating partners about using condoms	Self-report 1-item measure:<br>1-Ever used private technology (i.e., "electronically interacting with someone in a way that is not visible to the public, such as Snapchat, or private Facebook messaging") to communicate with dating partners about using condoms.<br>Dating partners defined as a boy/girlfriend or someone with whom participants had a romantic or sexual relationship.		Binary	Ever	Examples: texting, Snapchat, and Facebook	Mixed platforms	SNS (active use)	NA
Freq. of SM use	Widman 2014	Used technology based sexual communication to communicate with dating partners about using other forms of birth control	Self-report 1-item measure:<br>1-Ever used private technology (i.e., "electronically interacting with someone in a way that is not visible to the public, such as Snapchat, or private Facebook messaging") to communicate with dating partners about using other forms of birth control.<br>Dating partners defined as a boy/girlfriend or someone with whom participants had a romantic or sexual relationship.		Binary	Ever	Examples: texting, Snapchat, and Facebook	Mixed platforms	SNS (active use)	NA
Freq. of SM use	Widman 2014	Used technology based sexual communication to communicate with dating partners about HIV/AIDS	Self-report 1-item measure:<br>1-Ever used private technology (i.e., "electronically interacting with someone in a way that is not visible to the public, such as Snapchat, or private Facebook messaging") to communicate with dating partners about HIV/AIDS.<br>Dating partners defined as a boy/girlfriend or someone with whom participants had a romantic or sexual relationship.		Binary	Ever	Examples: texting, Snapchat, and Facebook	Mixed platforms	SNS (active use)	NA
Freq. of SM use	Widman 2014	Used technology based sexual communication to communicate with dating partners about STIs	Self-report 1-item measure:<br>1-Ever used private technology (i.e., "electronically interacting with someone in a way that is not visible to the public, such as Snapchat, or private Facebook messaging") to communicate with dating partners about STIs.<br>Dating partners defined as a boy/girlfriend or someone with whom participants had a romantic or sexual relationship.		Binary	Ever	Examples: texting, Snapchat, and Facebook	Mixed platforms	SNS (active use)	NA

**Exposure**	**Author and year**	**Exposure definition**	**Exposure ascertainment**	**Validated/ objectively recorded**	**Exposure measure type**	**Exposure time period**	**SM platform**	***SM platform***	***SM category (active/ passive use)***	***SM content***
Freq. of SM use	Widman 2014	Used technology based sexual communication to communicate with dating partners about risk of pregnancy	<u>Self-report 1-item measure:</u><br>1-Ever used private technology (i.e., "electronically interacting with someone in a way that is not visible to the public, such as Snapchat, or private Facebook messaging") to communicate with dating partners about risk of pregnancy.<br>Dating partners defined as a boy/girlfriend or someone with whom participants had a romantic or sexual relationship.		Binary	Ever	Examples: texting, Snapchat, and Facebook	Mixed platforms	SNS (active use)	NA
Freq. of SM use	Widman 2014	Used technology based sexual communication to communicate with dating partners about sexual limits	<u>Self-report 1-item measure:</u><br>1-Ever used private technology (i.e., "electronically interacting with someone in a way that is not visible to the public, such as Snapchat, or private Facebook messaging") to communicate with dating partners about sexual limits.<br>Dating partners defined as a boy/girlfriend or someone with whom participants had a romantic or sexual relationship.		Binary	Ever	Examples: texting, Snapchat, and Facebook	Mixed platforms	SNS (active use)	NA
Freq. of SM use	Wulff 2021	Freq. of WhatsApp use	<u>Self-report 1-item measure:</u><br>1-Frequency of social network use (e.g., Twitter, Facebook) and used functions (e.g., texting, view pictures, posting content)<br>Responses: never, at least once a month, at least once a week, daily, more than 1 hr/day.		Binary	Current	Specified: WhatsApp	WhatsApp	SNS (active and passive use)	NA
Freq. of SM use	Wulff 2021	Freq. of YouTube use	<u>Self-report 1-item measure:</u><br>1-Frequency of social network use (e.g., Twitter, Facebook) and used functions (e.g., texting, view pictures, posting content)<br>Responses: never, at least once a month, at least once a week, daily, more than 1 hr/day.		Binary	Current	Specified: YouTube	YouTube	Media-sharing (active and passive use)	NA
Freq. of SM use	Wulff 2021	Freq. of Instagram use	<u>Self-report 1-item measure:</u><br>1-Frequency of social network use (e.g., Twitter, Facebook) and used functions (e.g., texting, view pictures, posting content)<br>Responses: never, at least once a month, at least once a week, daily, more than 1 hr/day.		Binary	Current	Specified: Instagram	Instagram	Media-sharing (active and passive use)	NA
Freq. of SM use	Wulff 2021	Freq. of Facebook use	<u>Self-report 1-item measure:</u><br>1-Frequency of social network use (e.g., Twitter, Facebook) and used functions (e.g., texting, view pictures, posting content)<br>Responses: never, at least once a month, at least once a week, daily, more than 1 hr/day.		Binary	Current	Specified: Facebook	Facebook	SNS (active and passive use)	NA

Exposure	Author and year	Exposure definition	Exposure ascertainment	Validated/ objectively recorded	Exposure measure type	Exposure time period	SM platform	*SM platform*	*SM category (active/ passive use)*	*SM content*
Freq. of SM use	Wulff 2021	Freq. of Google+ use	<u>Self-report 1-item measure:</u><br>1-Frequency of social network use (e.g., Twitter, Facebook) and used functions (e.g., texting, view pictures, posting content)<br>Responses: never, at least once a month, at least once a week, daily, more than 1 hr/day.		Binary	Current	Specified: Google +	Google+	SNS (active and passive use)	NA
Freq. of SM use	Wulff 2021	Freq. of Twitter use	<u>Self-report 1-item measure:</u><br>1-Frequency of social network use (e.g., Twitter, Facebook) and used functions (e.g., texting, view pictures, posting content)<br>Responses: never, at least once a month, at least once a week, daily, more than 1 hr/day.		Binary	Current	Specified: Twitter	Twitter	Micro-blogging (active and passive use)	NA
Exposure to health-risk behaviour content	Baldwin 2018	Watched food/ beverage brand YouTube videos	<u>Self-report 1-item measure:</u><br>1-Ever watched any commercials/ads for food or drink products on YouTube.<br>Responses: yes/no.		Binary	Ever	Specified: YouTube	YouTube	Media-sharing (passive use)	Marketer-gen
Exposure to health-risk behaviour content	Baldwin 2018	Seen favourite food advertised on SM	<u>Self-report 1-item measure:</u><br>1-Had seen favourite food brands advertised on SM.<br>Responses: yes/no.		Binary	NR	NR	NR	General SM (passive use)	Marketer-gen
Exposure to health-risk behaviour content	Baldwin 2018	Liked a food/ beverage brand on Facebook	<u>Self-report 1-item measure:</u><br>1-Ever liked any food/beverage companies or brands on Facebook (e.g., they liked or shared any of these pages' content).<br>Responses: yes/no.		Binary	Ever	Specified: Facebook	Facebook	SNS (active use)	Marketer-gen
Exposure to health-risk behaviour content	Baldwin 2018	Entered a food/ beverage brand competition on Facebook	<u>Self-report 1-item measure:</u><br>1-Ever entered a food/beverage brand competition/contest on Facebook.<br>Responses: yes/no.		Binary	Ever	Specified: Facebook	Facebook	SNS (active use)	Marketer-gen
Exposure to health-risk behaviour content	Bayraktar 2007	Online gaming: murdering games	<u>Self-report measure:</u><br>Completed a set of questionnaires related to internet experience, internet sites used (chatting sites, music sites, popstar sites, played games online etc), and reasons for internet usage etc.<br>No further information provided.		Continuous	Current	NR	NR	Online Gaming (active use)	Marketer-gen
Exposure to health-risk behaviour content	Bayraktar 2007	Online gaming: fighting games	<u>Self-report measure:</u><br>Completed a set of questionnaires related to internet experience, internet sites used (chatting sites, music sites, popstar sites, played games online etc), and reasons for internet usage etc. No further information provided.		Continuous	Current	NR	NR	Online Gaming (active use)	Marketer-gen
Exposure to health-risk behaviour content	Bayraktar 2007	Online gaming: bombing games	<u>Self-report measure:</u><br>Completed a set of questionnaires related to internet experience, internet sites used (chatting sites, music sites, popstar sites, played games online etc), and reasons for internet usage etc. No further information provided.		Continuous	Current	NR	NR	Online Gaming (active use)	Marketer-gen

Exposure	Author and year	Exposure definition	Exposure ascertainment	Validated/ objectively recorded	Exposure measure type	Exposure time period	SM platform	*SM platform*	*SM category (active/ passive use)*	*SM content*
Exposure to health-risk behaviour content	Camenga 2018	Exposure to e-cigarette advertisements on Facebook	Self-report 1-item measure:<br>1-Recently seen advertisements on Facebook.<br>Responses: yes/no.		Binary	Current	Specified: Facebook	Facebook	SNS (passive use)	Marketer-gen
Exposure to health-risk behaviour content	Camenga 2018	Exposure to e-cigarette advertisements on Twitter	Self-report 1-item measure<br>1-Recently seen advertisements on Twitter.<br>Responses: yes/no.		Binary	Current	Specified: Twitter	Twitter	Micro-blogging (passive use)	Marketer-gen
Exposure to health-risk behaviour content	Camenga 2018	Exposure to e-cigarette advertisements on YouTube	Self-report 1-item measure<br>1-Recently seen advertisements on YouTube.<br>Responses: yes/no.		Binary	Current	Specified: YouTube	YouTube	Media-sharing (passive use)	Marketer-gen
Exposure to health-risk behaviour content	Camenga 2018	Exposure to e-cigarette advertisements on Pinterest/ Google +	Self-report 1-item measure<br>1-Recently seen advertisements on Pinterest/Google +.<br>Responses: yes/no.		Binary	Current	Specified: Google + and Pinterest	Mixed platforms	Media-sharing (passive use)	Marketer-gen
Exposure to health-risk behaviour content	Cavazos-Rehg 2014	Exposure to tobacco ads/ promotions via Facebook or Myspace	Self-report 2-item measure:<br>If during the past 30 days had:<br>1-received coupons from a tobacco company through…;<br>2-received ads from a tobacco company through….<br>Participants could select one or more responses from the following choices: the mail, E-mail, the Internet, Facebook, Myspace, a text message.<br>Responses for Facebook and Myspace combined to represent variable.		Binary	Past month	Specified: Facebook and Myspace	Facebook & Myspace	SNS (unclear)	Marketer-gen
Exposure to health-risk behaviour content	Chen 2019	Exposure to risky selfie descriptive norms	Self-report 1-item measure:<br>1-How many of friends posted risky selfies.<br>Responses: (1) nobody to (5) everybody.		Continuous	Current	NR	NR	General SM (passive use)	User-gen
Exposure to health-risk behaviour content	Coates 2019	Exposure to unhealthy mock Instagram influencer marketing	Exposed to mock Instagram profiles for 2 SM influencers (male and female). Profiles consisted of the Instagram banner and 6 images (3 test and 3 filler unbranded non-food items) of influencer holding a product (unhealthy e.g., choc cookies; healthy e.g., banana; branded non-food e.g., sneakers). Images obtained and edited from influencer YouTube channels. Participant randomly exposed to 1 of 3 mock Instagram profiles of an influencer holding a product: unhealthy snacks, healthy snacks, or branded non-food items. Counterbalancing of participants to condition, and influencer order (man first or woman first), was conducted by using randomizer.org.	Yes	Categorical	Current	Specified: Instagram	Instagram	SNS (passive use)	Marketer-gen

Exposure	Author and year	Exposure definition	Exposure ascertainment	Validated/ objectively recorded	Exposure measure type	Exposure time period	SM platform	*SM platform*	*SM category (active/ passive use)*	*SM content*
Exposure to health-risk behaviour content	Critchlow 2019	Participation with alcohol marketing on SM	Self-report 5-item measure:<br>If had:<br>1-liked an alcohol brand on SM, such as Twitter, Facebook or Instagram;<br>2-shared something related to an alcohol drinks brand, such as a status, Tweet, or picture;<br>3-followed an alcohol brand on social media;<br>4-entered a competition run by an alcoholic drink brand online or on social media; and<br>5-searched for alcoholic drinks adverts on websites, such as YouTube.<br>Responses: yes/no/none of the above.<br>A cumulative score was computed (0–5).<br>3 category variable constructed: no participation with any marketing, participation with 1 form of marketing, or participation with ≥2 forms of marketing.		Ordinal	Past month	Examples: Twitter, Facebook, Instagram, and SM	Mixed platforms	General SM (active use)	Marketer-gen
Exposure to health-risk behaviour content	Critchlow 2019	Participation with user-created alcohol promotion on SM	Self-report 1-item measure:<br>1-Updated status or uploaded pictures of themselves or friends drinking an alcoholic drink.<br>Responses: yes/no.		Binary	Past month	NR	NR	General SM (active use)	User-gen
Exposure to health-risk behaviour content	Dai 2022	Exposure to e-cigarette advertisements on SM	Self-report 1-item validated measure:<br>1-Seen e-cigarette advertisements on SM in the past 30 days. Responses: never, sometimes (once or twice), and often (more than 3 times).<br>Variable dichotomised: yes (sometimes/often)/no (never).	Yes	Binary	Past month	NR	NR	General SM (passive use)	Marketer-gen
Exposure to health-risk behaviour content	Davis 2019	Substance-related media exposure via SM	Self-report 2-item measure:<br>1,2-How often saw or heard pictures or comments on a SNS (e.g., Facebook) showing someone or talking about someone who is drunk.<br>Responses: (0) not at all to (6) every day.<br>Average of 2 items taken for analysis.		Continuous	Past 3 months	Example: Facebook	Facebook	SNS (passive use)	User + Marketer-generated
Exposure to health-risk behaviour content	Dawson 2019	% of participant posts sharing inappropriate content on Facebook	Observationally coded measure coded using Mikami and Szwedo's Facebook Coding Manual.[158]<br>Facebook profile coded over 2-month period to obtain proportion of total participant posts shared containing inappropriate content (i.e., profanity, substance use, sexual behaviour, violence, or other illegal behaviour).	Yes	Continuous	Current	Specified: Facebook	Facebook	SNS (active use)	User-gen
Exposure to health-risk behaviour content	Dawson 2019	% of participant posts containing relational aggression on Facebook	Observationally coded measure coded using Mikami and Szwedo's Facebook Coding Manual.[158]<br>Facebook profile coded over 2-month period to obtain proportion of total participant posts containing relational aggression (i.e., comments meant to criticize, ostracize, or embarrass a person or group of people).	Yes	Continuous	Current	Specified: Facebook	Facebook	SNS (active use)	User-gen

Exposure	Author and year	Exposure definition	Exposure ascertainment	Validated/ objectively recorded	Exposure measure type	Exposure time period	SM platform	*SM platform*	*SM category (active/ passive use)*	*SM content*
Exposure to health-risk behaviour content	Dawson 2019	% of participant friend posts containing relational aggression on Facebook	Observationally coded measure coded using Mikami and Szwedo's Facebook Coding Manual.[158] Facebook profile coded over 2-month period to obtain proportion of friend posts on participant timeline containing content on relational aggression (i.e., comments meant to criticize, ostracise, or embarrass a person or group of people).	Yes	Continuous	Current	Specified: Facebook	Facebook	SNS (passive use)	User-gen
Exposure to health-risk behaviour content	Dawson 2019	% of participant friend posts containing inappropriate content	Observationally coded measure coded using Mikami and Szwedo's Facebook Coding Manual.[158] Facebook profile coded over 2-month period to obtain proportion of friend posts on participant timeline containing inappropriate content (i.e., profanity, substance use, sexual behaviour, violence, or other illegal behaviour).	Yes	Continuous	Current	Specified: Facebook	Facebook	SNS (passive use)	User-gen
Exposure to health-risk behaviour content	de Bruijn 2016	Ever used alcohol branded SM page	Self-report 1-item measure: 1-Ever used a profile page on sites such as Hyves, Facebook, MSN, or Myspace containing an alcohol brand or logo. Responses: never, rarely/sometimes, and often/very often.		Binary	Ever	Examples: Hyves, Facebook, MSN, and Myspace	Mixed platforms	SNS (passive use)	Marketer-gen
Exposure to health-risk behaviour content	De Jans 2021	Exposure to snack with low nutritional value (mini donut) on Instagram	Exposed to 1 of 2 individual Instagram posts: 1-Post portraying snack high in nutritional value (i.e., strawberries). 2-Post portraying snack low in nutritional value (i.e., donuts)	Yes	Binary	Current	Specified: Instagram	Instagram	Media-sharing (passive use)	Marketer-gen
Exposure to health-risk behaviour content	Doornwaard 2014	Exposure to displays of sexual references on Facebook	Observationally coded measure using codebook based on procedures used in previous content analyses of SNS.[159] Reviewers analysed visible elements on participant Facebook timeline via content analysis: status updates, images, comments and downloaded icons to investigate sexual references on Facebook related to safe sex, risky sex, sexual behaviour, sexualised personal descriptions, revealing personal images, sexual paraphernalia, and romance. Sexual references defined as any textual or visual depiction of sexual activity or sexually suggestive behaviour. References did not need to be created by the owner (participant), they included posts, comments, tags by friends on the participant's profile. Sexual reference displayers compared with non-sexual reference displayers.	Yes	Binary	During coding period	Specified: Facebook	Facebook	SNS (active and passive use)	User-gen

Exposure	Author and year	Exposure definition	Exposure ascertainment	Validated/ objectively recorded	Exposure measure type	Exposure time period	SM platform	*SM platform*	*SM category (active/ passive use)*	*SM content*
Exposure to health-risk behaviour content	Folkvord 2020	Exposure to manipulated popular influencer Instagram post showing energy dense foods	Exposed to 1 of 2 popular SM influencers on Instagram.<br>1-Post showing vegetables (control condition)<br>2-Post showing energy dense snacks (experimental condition)<br>3-Post showing non-food products	Yes	Binary	Current	Specified: Instagram	Instagram	Media-sharing (passive use)	Marketer-gen
Exposure to health-risk behaviour content	Gascoyne 2021	Seen an advertisement for a food or drink product on SM (e. g. Facebook, Instagram)	<u>Self-report 1-item measure:</u><br>1-How often in last month saw a food or drink product on SM (e. g. Facebook, Instagram).<br>Responses: not in the last month, 1–3 times a month, 1–3 times a week and daily or almost daily.		Ordinal	Past month	Examples: Facebook and Instagram	Mixed platforms	General SM (passive use)	Marketer-gen
Exposure to health-risk behaviour content	Gascoyne 2021	Liked/ shared posts related to a food or drink product or brand (e.g., soft drink, fast food)	<u>Self-report 1-item measure:</u><br>1-How often in last month 'liked' or 'shared' posts related to a food or drink product or brand (e.g., soft drink, fast food).<br>Responses: not in the last month, 1–3 times a month, 1–3 times a week and daily or almost daily.		Ordinal	Past month	Examples: Facebook and Instagram	Mixed platforms	General SM (active use)	Marketer-gen
Exposure to health-risk behaviour content	Gerber 2021	Exposure to alcohol related content on Instagram and Snapchat	<u>Self-report 2-item measure:</u><br>1-How often see content in which alcohol is present on Instagram.<br>2-How often see content in which alcohol is present on Snapchat.<br>Responses: (1) never to (5) very often.<br>Mean of both items calculated, representing exposure to alcohol-related content on SM.		Continuous	Current	Specified: Instagram and Snapchat	Mixed platforms	Media-sharing (passive use)	User + Marketer-generated
Exposure to health-risk behaviour content	Gerber 2021	Shared alcohol related content on Instagram and Snapchat	<u>Self-report 2-item measure:</u><br>1-How often post or send content on Instagram depicting alcohol.<br>2-How often post or send content on Snapchat depicting alcohol.<br>Responses: (1) never to (5) very often.<br>Mean of both items calculated, representing exposure to alcohol-related content on SM.		Continuous	Current	Specified: Instagram and Snapchat	Mixed platforms	Media-sharing (active use)	User + Marketer-generated

Exposure	Author and year	Exposure definition	Exposure ascertainment	Validated/ objectively recorded	Exposure measure type	Exposure time period	SM platform	*SM platform*	*SM category (active/ passive use)*	*SM content*
Exposure to health-risk behaviour content	Geusens 2017	Frequency of sharing alcohol references on SNS	<u>Self-report 8-item measure:</u><br>How often privately shared the following things on any social medium and how often publicly shared the following things on any social medium:<br>1- photos or video clips referring to alcohol use;<br>2- textual updates referring to alcohol use;<br>3- photos or video clips in which they or their friends were drunk, and;<br>4- textual updates while they were drunk.<br>'Private sharing' was defined as 'references shared with a limited amount of people, e.g., through communication via direct messaging, private groups, or group chats'.<br>Responses: (0) never to (6) several times a day.		Continuous	Ever	NR	NR	SNS (active use)	User-gen
Exposure to health-risk behaviour content	Geusens 2017	Perceived number of friends sharing alcohol references online	<u>Self-report 4-item measure:</u><br>How many of their friends shared:<br>1-photos or video clips referring to alcohol use;<br>2-textual updates referring to alcohol use;<br>3-photos or video clips in which they are drunk, or;<br>4-textual updates while they are drunk.<br>Responses: (0) none to (4) all of them.		Continuous	Current	NR	NR	SNS (passive use)	User-gen
Exposure to health-risk behaviour content	Geusens 2019	Frequency of exposure to peer alcohol references on SNS	<u>Self-report 3-item measure:</u><br>How often saw videos or images:<br>1-on YouTube or similar sites or;<br>2-on other SNS, such as Facebook or Twitter, about youth drinking alcohol and;<br>3-youth being drunk.<br>Responses: (0) never to (8) all day long.<br>Factor analysis used to load all 3 items loaded onto one scale.		Continuous	Current	Examples: YouTube, Facebook, Twitter, and other SNS	Mixed platforms	SNS (passive use)	User-gen
Exposure to health-risk behaviour content	Geusens 2019	Frequency of sharing of alcohol references on SNS	<u>Self-report 1-item measure:</u><br>How often shared videos/images:<br>1-on YouTube or similar sites or;<br>2-on other SNS, such as Facebook or Twitter, about youth drinking alcohol and;<br>3- youth being drunk.<br>Responses: (0) never to (8) all day long.<br>Upper scale points collapsed after calculating the composite score with 0 (never), 1 (a few times per year), 2 (once per month), and 3 (more than once per month).		Continuous	Current	Examples: YouTube, Facebook, Twitter, and other SNS	Mixed platforms	SNS (active use)	User-gen

Exposure	Author and year	Exposure definition	Exposure ascertainment	Validated/ objectively recorded	Exposure measure type	Exposure time period	SM platform	*SM platform*	*SM category (active/ passive use)*	*SM content*
Exposure to health-risk behaviour content	Gordon 2011	Awareness of alcohol marketing on SNS	Self-report 1-item measure:<br>1-Had seen any alcohol marketing on SNS.<br>Responses: yes/no/don't know.		Binary	Current	NR	NR	SNS (passive use)	Marketer-gen
Exposure to health-risk behaviour content	Gordon 2011	Used SNS containing alcohol brands or logos	Self-report 1-item measure:<br>1-Has used SNS containing alcohol brands or logos.<br>Responses: yes/no/don't know.		Binary	Current	NR	NR	SNS (passive use)	Marketer-gen
Exposure to health-risk behaviour content	Hrywna 2020	Liked or followed a tobacco brand on SM	Self-report 1-item measure:<br>1- Had visited, followed, liked, or become a fan of a tobacco brand on sites like Instagram, Twitter, Facebook, or YouTube.		Binary	Past year	Examples: Instagram, Twitter, Facebook, and YouTube	Mixed platforms	SNS (active and passive use)	Marketer-gen
Exposure to health-risk behaviour content	Huang 2014	Number of friends who posted risky pictures partying or drinking	Self-report measure:<br>Egocentric (personal) networks created for each participant, participant asked to name 7 best friends regardless of where they live or go to school and provide basic information about each of them (alters).<br>Friends' online behaviours were assessed by asking whether alters ever "posted pictures of themselves partying or drinking alcohol online"<br>Indicator friends' online risk behaviour created using total number of alters for these items.		Continuous	Ever	NR	NR	SNS (passive use)	User-gen
Exposure to health-risk behaviour content	Lin 2012	Used SNS containing alcohol brands or logos	Self-report 1-item measure:<br>1-Used SNS containing alcohol brands or logos.<br>Responses: yes/no/don't know.		Binary	Current	NR	NR	SNS (passive use)	Marketer-gen
Exposure to health-risk behaviour content	Lin 2012	Awareness of alcohol marketing on SNS	Self-report measure:<br>If they could think of any makes or brands of alcohol that they had seen or heard advertised recently.<br>Followed by a set of questions assessing awareness across 15 types of marketing channels (including SNS).<br>Response: yes/no/don't know.		Binary	Current	NR	NR	SNS (passive use)	Marketer-gen
Exposure to health-risk behaviour content	Nesi 2017	Ever exposed to friends' SNS alcohol content	Self-report 2-item measure:<br>Whether a friend had ever:<br>1-posted a picture of themselves with alcohol, or;<br>2-posted a status, picture, or link about drinking alcohol.<br>Items combined, with endorsement of either item coded as (1) and endorsement of neither as (0).		Binary	Ever	NR	NR	SNS (passive use)	User-gen

Exposure	Author and year	Exposure definition	Exposure ascertainment	Validated/ objectively recorded	Exposure measure type	Exposure time period	SM platform	*SM platform*	*SM category (active/ passive use)*	*SM content*
Exposure to health-risk behaviour content	Nesi 2017	Exposure to SNS alcohol content posted by self	<u>Self-report 5-item measure:</u><br>If had:<br>1-posted status, picture, or link about alcohol;<br>2-posted picture of self with alcohol;<br>3-tagged friends in photos with alcohol;<br>4-posted picture of self, passed out or vomiting as result of alcohol;<br>5-posted picture of friend passed out or vomiting as a result of alcohol.		Binary	Ever	NR	NR	SNS (active use)	User-gen
Exposure to health-risk behaviour content	Ngqangashe 2021	Watched YouTube Tasty video portraying preparation of sweet snacks	Exposed to 1 of 2 short-form 'Tasty' culinary videos on YouTube.<br>1-Video portraying sweet snacks (The Best Fudgy Brownies Ever, Chocolate Peanut Brownies (Buckeye Brownies), and 6 Ways to Make Better Boxed Brownies.<br>2-Video portraying fruits and vegetables (4 Make-Ahead Vegetable-Packed Smoothies, 4 Healthier Desserts, and Fruit Salad Four Ways).	Yes	Binary	Current	Specified: YouTube	YouTube	Media-sharing (passive use)	Marketer-gen
Exposure to health-risk behaviour content	Pegg 2018	SNS alcohol exposure	<u>Self-report 1-item measure:</u><br>1-How often in the previous 6 months friends posted pictures, updates, or wall posts that showed or talked about them drinking alcohol.<br>Responses: (1) none to (8) ≥31 times.		Continuous	Past 6 months	NR	NR	SNS (active and passive use)	User-gen
Exposure to health-risk behaviour content	Pérez 2022	Exposure to tobacco related content on SM in the past 12 months (including e-cigarettes)	<u>Self-report 1-item measure:</u><br>1-In past 12 months, posted content about tobacco products (including e-cigarettes) on any SM sites.<br>Responses: yes/no.		Binary	Past year	NR	NR	General SM (active use)	User + Marketer-generated
Exposure to health-risk behaviour content	Pérez 2022	Posted tobacco related content on SM in the past 12 months (including e-cigarettes)	<u>Self-report 1-item measure:</u><br>1-In past 12 months, seen content posted about tobacco products (including e-cigarettes) on SM sites.<br>Responses: yes/no.		Binary	Past year	NR	NR	General SM (passive use)	User + Marketer-generated
Exposure to health-risk behaviour content	Qutteina 2022	Exposure to non-core foods (energy dense, low nutrient: sweetened drinks, sweets, salty/savoury snacks) on SM	<u>Self-report measure:</u><br>1-How often saw food messages posted by friends, influencers, and celebrities as well as messages posted by brands.<br>Responses: (1) not at all to (5) very often.		Ordinal	Current	NR	NR	General SM (passive use)	User + Marketer-generated

Exposure	Author and year	Exposure definition	Exposure ascertainment	Validated/ objectively recorded	Exposure measure type	Exposure time period	SM platform	*SM platform*	*SM category (active/ passive use)*	*SM content*
Exposure to health-risk behaviour content	Qutteina 2022	Exposure to branded non-core foods (energy dense, low nutrient: sweetened drinks, sweets, salty/savoury snacks) on SM	Self-report measure:<br>1-How often saw food messages posted by friends, influencers, and celebrities as well as messages posted by brands.<br>Responses: (1) not at all to (5) very often.		Ordinal	Current	NR	NR	General SM (passive use)	User + Marketer-generated
Exposure to health-risk behaviour content	Roditis 2016	Ever seen a message posted on SM about the benefits or good things of using marijuana	Self-report 1-item measure:<br>1-Ever seen a message posted on SM about benefits or good things related to using marijuana.<br>Responses: yes/no.		Binary	Ever	NR	NR	General SM (passive use)	User + Marketer-generated
Exposure to health-risk behaviour content	Shan 2022	Followed tobacco brands on Facebook/Twitter or other SM sites	Self-report 1-item measure:<br>1-In past 12 months, liked or followed any of the following brands (e.g., Marlboro, Newport, American Spirit, Vuse) on Facebook, Twitter, or other SM sites.<br>Responses; yes/no		Binary	Past year	Examples: Facebook and Twitter	Mixed platforms	General SM (active use)	Marketer-gen
Exposure to health-risk behaviour content	Sharma 2021	Exposure to tobacco adverts on SM	Self-report measure:<br>Modified Global Youth Tobacco Survey (GYTS) questionnaire.<br>Responses: yes/no. No further information reported.		Binary	Current	NR	NR	General SM (passive use)	Marketer-gen
Exposure to health-risk behaviour content	Smout 2021	Exposure to peer-generated content on SM depicting risky substance use	Self-report 1-item measure:<br>1-See pictures of kids drunk, passed out or using drugs on these sites?<br>Responses: yes/no		Binary	Current	Specified: Facebook, Myspace, and other SNS	Mixed platforms	SNS (passive use)	User-gen
Exposure to health-risk behaviour content	Trangenstein 2019	Liked/follow cannabis business pages on Facebook, Twitter and/or Instagram	Self-report measure:<br>1-Engagement: if like/follow any cannabis business pages on Facebook, Instagram and/or Twitter and if so, which ones. Engagement defined as liking, following, and/or commenting on a cannabis business page.<br>Responses: yes/no.		Binary	Current	Specified: Facebook, Twitter, and Instagram	Mixed platforms	SNS (active use)	Marketer-gen
Exposure to health-risk behaviour content	Trangenstein 2019	Liked/follow cannabis business pages on Facebook	Self-report 1-item measure:<br>1-Engagement: if like/follow any cannabis business pages on Facebook. Engagement defined as liking, following, and/or commenting on a cannabis business page.<br>Responses: yes/no.		Binary	Current	Specified: Facebook	Facebook	SNS (active use	Marketer-gen

**Exposure**	**Author and year**	**Exposure definition**	**Exposure ascertainment**	**Validated/ objectively recorded**	**Exposure measure type**	**Exposure time period**	**SM platform**	***SM platform***	***SM category (active/ passive use)***	***SM content***
Exposure to health-risk behaviour content	Trangenstein 2019	Liked/follow cannabis business pages on Twitter	Self-report 1-item measure:<br>1-Engagement: if like/follow any cannabis business pages on Twitter. Engagement defined as liking, following, and/or commenting on a cannabis business page.<br>Responses: yes/no.		Binary	Current	Specified: Twitter	Twitter	Micro-blogging (active use)	Marketer-gen
Exposure to health-risk behaviour content	Trangenstein 2019	Liked/follow cannabis business pages on Instagram	Self-report 1-item measure:<br>1-Engagement: if like/follow any cannabis business pages on Instagram. Engagement defined as liking, following, and/or commenting on a cannabis business page.<br>Responses: yes/no.		Binary	Current	Specified: Instagram	Instagram	Media-sharing (active use)	Marketer-gen
Exposure to health-risk behaviour content	Yao 2022	Exposure to content (including text and pictures) about drinking or smoking (e.g., saw drinking-related information)	Self-report 1-item measure:<br>1-How often exposed to content (including text and pictures) about drinking or smoking (e.g., saw drinking-related information) in three popular Chinese social media platforms—WeChat Moment, Qzone, and Weibo<br>Responses: (1) never to (5) always) with higher scores indicating higher frequency of SM exposure to tobacco and alcohol content		Continuous	Current	Specified: WeChat Moment, Qzone, and Weibo	Mixed Platforms	General SM (passive use)	User + Marketer-generated
Exposure to health-risk behaviour content	Whitehill 2020	Cumulative frequency of exposure to cannabis promotions (Facebook, Twitter, and Instagram)	Self-report 3-item measure:<br>When using (1-Facebook, 2-Twitter, and 3-Instagram), how often saw ads or promotions for cannabis or related products.<br>Responses: never, rarely, sometimes, most of the time, always, and do not use platform.<br>Variable dichotomised for each platform: rarely, sometimes, most of the time, and always/never and I do not use (1-Facebook, 2-Twitter, 3-Instagram).<br>Binary variables summed to give counts of exposure across Facebook, Twitter and Instagram giving cumulative exposure.		Continuous	Current	Specified: Facebook, Twitter, and Instagram	Mixed platforms	SNS (passive use)	Marketer-gen
Exposure to health-risk behaviour content	Whitehill 2020	Frequency of exposure to cannabis promotions on Facebook	Self-report 1-item measure:<br>1-When using Facebook, how often saw ads or promotions for cannabis or related products.<br>Responses: never, rarely, sometimes, most of the time, always, and do not use platform.<br>Variable dichotomised: rarely, sometimes, most of the time, and always/never and I do not use Facebook.		Binary	Current	Specified: Facebook	Facebook	SNS (passive use)	Marketer-gen

**Exposure**	**Author and year**	**Exposure definition**	**Exposure ascertainment**	**Validated/ objectively recorded**	**Exposure measure type**	**Exposure time period**	**SM platform**	***SM platform***	***SM category (active/ passive use)***	***SM content***
Exposure to health-risk behaviour content	Whitehill 2020	Frequency of exposure to cannabis promotions on Twitter	<u>Self-report 1-item measure:</u><br>1-When using Twitter, how often saw ads or promotions for cannabis or related products.<br>Responses: never, rarely, sometimes, most of the time, always, and do not use platform.<br>Variable dichotomised: rarely, sometimes, most of the time, and always/ never and I do not use Twitter.		Binary	Current	Specified: Twitter	Twitter	Micro-blogging (passive use)	Marketer-gen
Exposure to health-risk behaviour content	Whitehill 2020	Frequency of exposure to cannabis promotions on Instagram	<u>Self-report 1-item measure:</u><br>1-When using Instagram, how often saw ads or promotions for cannabis or related products.<br>Responses: never, rarely, sometimes, most of the time, always, and do not use platform.<br>Variable dichotomised: rarely, sometimes, most of the time, and always/ never and I do not use Instagram.		Binary	Current	Specified: Instagram	Instagram	Media-sharing (passive use)	Marketer-gen
Other SM activities	Chapin 2018	Number of SM platforms used	<u>Self-report measure:</u><br>Asked to circle which SM platforms used and to identify which were favourites.<br>A space was provided for students write in other platforms they were using.		Continuous	Current	Examples: Texting, Facebook, Twitter, Instagram, Pinterest, and Snapchat	Mixed platforms	SNS (unclear)	NA
Other SM activities	Gomez 2019	Signed up to more than 5 SNS	<u>Self-report measure</u>:<br>No further information reported.		Binary	NR	NR	NR	SNS (unclear)	NA
Other SM activities	Kaufman 2014	Has a Facebook account	<u>Self-report 1-item measure:</u><br>1-Had a Facebook account.<br>Responses: yes/no.		Binary	Current	Specified: Facebook	Facebook	SNS (unclear)	NA
Other SM activities	Kaufman 2014	Has a Mxit account	<u>Self-report 1-item measure:</u><br>1-Had a Mxit account.<br>Responses: yes/no.		Binary	Current	Specified: Mxit	Mxit	SNS (unclear)	NA
Other SM activities	Landry 2013	Has a Facebook account	<u>Self-report measure:</u><br>If internet used, how often, and if had accounts on any of the following SM sites: Facebook, Myspace, Twitter, Yahoo, YouTube, My yearbook, Tumblr, Google buzz, Flickr, Ustream, and other.<br>Responses: yes/no.		Binary	Current	Specified: Facebook, Myspace, Twitter, Yahoo, YouTube, My Yearbook, Tumblr, Google buzz, Flickr, Ustream, and other	Facebook	SNS (unclear)	NA
Other SM activities	Landry 2013	Has a SM account	<u>Self-report measure:</u><br>If internet used, how often, and if had accounts on any of the following SM sites: Facebook, Myspace, Twitter, Yahoo, YouTube, My yearbook, Tumblr, Google buzz, Flickr, Ustream, and other.<br>Responses: yes/no.		Binary	Current	Specified: Facebook, Myspace, Twitter, Yahoo, YouTube, My Yearbook, Tumblr, Google buzz, Flickr, Ustream, and other	Mixed platforms	SNS (unclear)	NA
Other SM activities	Molla-Esparza 2021	Number of SM platforms used	<u>Self-report 1-item measure:</u><br>1-Number of SM platforms used.		Continuous	Current	NR	NR	General SM (unclear)	NA

Exposure	Author and year	Exposure definition	Exposure ascertainment	Validated/ objectively recorded	Exposure measure type	Exposure time period	SM platform	*SM platform*	*SM category (active/ passive use)*	*SM content*
Other SM activities	Nesi 2019	Online status-seeking strategy use	Self-report 2-item measure:<br>Rated use of strategies to manage their online presence and accumulate online status indicators:<br>1-"I purposefully post on social media during 'high traffic' times (i.e., times that I know most people will see it) so that my posts/photos get more likes and comments" and;<br>2-"If something I post does not get a lot of likes or comments, I might take it down."<br>Responses: from (1) not at all true to (5) extremely true.		Continuous	Current	NR	NR	SNS (unclear)	NA
Other SM activities	Roditis 2016	Ever seen a message posted on SM about the risks or bad things of using marijuana	Self-report 1-item measure:<br>1-Ever seen a message posted on SM about the risks or bad things related to using marijuana.<br>Responses: yes/no.		Binary	Ever	NR	NR	General SM (passive use)	NA
Other SM activities	Stevens 2017	Exposure to contraception information on SNS	Self-report measure:<br>In past 30 days, where had heard about pregnancy prevention among young people.<br>Options included SNS.<br>Responses: yes/no.		Binary	Past month	NR	NR	SNS (unclear)	NA
Other SM activities	Stevens 2017	Exposure to HIV/STD information on SNS	Self-report measure:<br>In past 30 days, where had heard about HIV or STDs.<br>Options included SNS.<br>Responses: yes/no		Binary	Past month	NR	NR	SNS (unclear)	NA
Other SM activities	Suwanwong 2021	Exposure to Anti-smoking SM campaign	Self-report 1-item measure:<br>1-In past 30 days, seen any information about anti-smoking in SM.<br>Responses: yes/no.		Binary	Past month	NR	NR	General SM (passive use)	NA

Legend: Abbreviations: Freq = Frequency; IM = Instant messaging; Hrs = Hours; Marketer-gen = Marketer-generated content; Min = Minutes; NA = Not applicable; NR = Not reported; SM = Social media; SNS = Social networking sites; T = Timepoint; and User-gen = User-generated content.

Appendix 15. Exposure and outcome combinations amenable to meta-analysis

Table A. List of outcomes and exposures for which sufficient data were available to undertake meta-analysis, subgroup analysis/meta-regression[a] or sensitivity analysis

Outcome	Exposure	Exposure measure type	Outcome measure type	Common metric	Meta-analysis	Prespecified sensitivity analyses				Prespecified subgroup analyses/meta-regression[a]						
						Study design	Excl. age overlap	Adjust-ment	RoB	Sex	Age	Income	SEP	SM platform	SM category	SM content
Alcohol use	Time spent on SM	Continuous	Continuous	Std. Beta	✓	✓		✓								
		Continuous	Continuous	Std. mean difference	✓											
		Continuous	Binary	Odds ratio												
		Binary	Binary/ continuous	Odds ratio	✓	✓	✓	✓	✓		✓				✓	
	Frequency of SM use	Continuous	Continuous	Std. Beta	✓	✓		✓			✓				✓	
		Continuous	Continuous	Std. mean difference												
		Continuous	Binary	Odds ratio												
		Binary	Binary/ continuous	Odds ratio	✓	✓	✓	✓	✓	✓	✓	✓		✓	✓	
	Exposure to health-risk behaviour content	Continuous	Continuous	Std. Beta	✓	✓	✓	✓	✓		✓		✓		✓	
		Continuous	Continuous	Std. mean difference												
		Continuous	Binary	Odds ratio												
		Binary	Binary/ continuous	Odds ratio	✓	✓		✓	✓						✓	✓

Outcome	Exposure	Exposure measure type	Outcome measure type	Common metric	Meta-analysis	Prespecified sensitivity analyses				Prespecified subgroup analyses/meta-regression[a]						
						Study design	Excl. age overlap	Adjust-ment	RoB	Sex	Age	Income	SEP	SM platform	SM category	SM content
Sexual risk behaviour	Time spent on SM	Continuous	Continuous	Std. Beta	✓											
		Continuous	Continuous	Std. mean difference	✓											
		Continuous	Binary	Odds ratio												
		Binary	Binary/ continuous	Odds ratio												
	Frequency of SM use	Continuous	Continuous	Std. Beta												
		Continuous	Continuous	Std. mean difference												
		Continuous	Binary	Odds ratio	✓	✓			✓						✓	
		Binary	Binary/ continuous	Odds ratio	✓		✓	✓	✓	✓	✓	✓	✓		✓	
	Exposure to health-risk behaviour content	Continuous	Continuous	Std. Beta												
		Continuous	Continuous	Std. mean difference												
		Continuous	Binary	Odds ratio												
		Binary	Binary/ continuous	Odds ratio												
Anti-social behaviour	Time spent on SM	Continuous	Continuous	Std. Beta												
		Continuous	Continuous	Std. mean difference	✓										✓	
		Continuous	Binary	Odds ratio												
		Binary	Binary/ continuous	Odds ratio												
	Frequency of SM use	Continuous	Continuous	Std. Beta												
		Continuous	Continuous	Std. mean difference												
		Continuous	Binary	Odds ratio												
		Binary	Binary/ continuous	Odds ratio	✓	✓	✓	✓	✓			✓			✓	
	Exposure to health-risk behaviour content	Continuous	Continuous	Std. Beta												
		Continuous	Continuous	Std. mean difference												
		Continuous	Binary	Odds ratio												
		Binary	Binary/ continuous	Odds ratio												

Outcome	Exposure	Exposure measure type	Outcome measure type	Common metric	Meta-analysis	Prespecified sensitivity analyses				Prespecified subgroup analyses/meta-regression[a]						
						Study design	Excl. age overlap	Adjust-ment	RoB	Sex	Age	Income	SEP	SM platform	SM category	SM content
Tobacco use	Time spent on SM	Continuous	Continuous	Std. Beta												
		Continuous	Continuous	Std. mean difference												
		Continuous	Binary	Odds ratio												
		Binary	Binary/ continuous	Odds ratio												
	Frequency of SM use	Continuous	Continuous	Std. Beta												
		Continuous	Continuous	Std. mean difference												
		Continuous	Binary	Odds ratio												
		Binary	Binary/ continuous	Odds ratio	✓	✓	✓	✓	✓		✓	✓			✓	
	Exposure to health-risk behaviour content	Continuous	Continuous	Std. Beta												
		Continuous	Continuous	Std. mean difference												
		Continuous	Binary	Odds ratio												
		Binary	Binary/ continuous	Odds ratio	✓	✓		✓	✓			✓			✓	
Drug use	Time spent on SM	Continuous	Continuous	Std. Beta												
		Continuous	Continuous	Std. mean difference	✓											
		Continuous	Binary	Odds ratio												
		Binary	Binary/ continuous	Odds ratio	✓				✓							
	Frequency of SM use	Continuous	Continuous	Std. Beta												
		Continuous	Continuous	Std. mean difference												
		Continuous	Binary	Odds ratio												
		Binary	Binary/ continuous	Odds ratio	✓	✓		✓	✓		✓			✓	✓	
	Exposure to health-risk behaviour content	Continuous	Continuous	Std. Beta												
		Continuous	Continuous	Std. mean difference												
		Continuous	Binary	Odds ratio												
		Binary	Binary/ continuous	Odds ratio												

Outcome	Exposure	Exposure measure type	Outcome measure type	Common metric	Meta-analysis	Prespecified sensitivity analyses				Prespecified subgroup analyses/meta-regression[a]						
						Study design	Excl. age overlap	Adjust-ment	RoB	Sex	Age	Income	SEP	SM platform	SM category	SM content
Inadequate physical activity	Time spent on SM	Continuous	Continuous	Std. Beta	✓			✓	✓		✓	✓			✓	
		Continuous	Continuous	Std. mean difference												
		Continuous	Binary	Odds ratio												
		Binary	Binary/ continuous	Odds ratio												
	Frequency of SM use	Continuous	Continuous	Std. Beta												
		Continuous	Continuous	Std. mean difference												
		Continuous	Binary	Odds ratio												
		Binary	Binary/ continuous	Odds ratio												
	Exposure to health-risk behaviour content	Continuous	Continuous	Std. Beta												
		Continuous	Continuous	Std. mean difference												
		Continuous	Binary	Odds ratio												
		Binary	Binary/ continuous	Odds ratio												
Unhealthy dietary behaviour	Time spent on SM	Continuous	Continuous	Std. Beta												
		Continuous	Continuous	Std. mean difference												
		Continuous	Binary	Odds ratio												
		Binary	Binary/ continuous	Odds ratio												
	Frequency of SM use	Continuous	Continuous	Std. Beta												
		Continuous	Continuous	Std. mean difference												
		Continuous	Binary	Odds ratio												
		Binary	Binary/ continuous	Odds ratio												
	Exposure to health-risk behaviour content	Continuous	Continuous	Std. Beta												
		Continuous	Continuous	Std. mean difference												
		Continuous	Binary	Odds ratio												
		Binary	Binary/ continuous	Odds ratio	✓	✓									✓	

Outcome	Exposure	Exposure measure type	Outcome measure type	Common metric	Meta-analysis	Prespecified sensitivity analyses				Prespecified subgroup analyses/meta-regression[a]						
						Study design	Excl. age overlap	Adjust-ment	RoB	Sex	Age	Income	SEP	SM platform	SM category	SM content
Gambling	Time spent on SM	Continuous	Continuous	Std. Beta												
		Continuous	Continuous	Std. mean difference												
		Continuous	Binary	Odds ratio												
		Binary	Binary/ continuous	Odds ratio												
	Frequency of SM use	Continuous	Continuous	Std. Beta												
		Continuous	Continuous	Std. mean difference												
		Continuous	Binary	Odds ratio												
		Binary	Binary/ continuous	Odds ratio	✓	✓		✓	✓		✓				✓	
	Exposure to health-risk behaviour content	Continuous	Continuous	Std. Beta												
		Continuous	Continuous	Std. mean difference												
		Continuous	Binary	Odds ratio												
		Binary	Binary/ continuous	Odds ratio												
Multiple risk behaviours	Time spent on SM	Continuous	Continuous	Std. Beta												
		Continuous	Continuous	Std. mean difference												
		Continuous	Binary	Odds ratio												
		Binary	Binary/ continuous	Odds ratio												
	Frequency of SM use	Continuous	Continuous	Std. Beta												
		Continuous	Continuous	Std. mean difference												
		Continuous	Binary	Odds ratio												
		Binary	Binary/ continuous	Odds ratio	✓											
	Exposure to health-risk behaviour content	Continuous	Continuous	Std.Beta												
		Continuous	Continuous	Std. mean difference												
		Continuous	Binary	Odds ratio												
		Binary	Binary/ continuous	Odds ratio												

Outcome	Exposure	Exposure measure type	Outcome measure type	Common metric	Meta-analysis	Prespecified sensitivity analyses				Prespecified subgroup analyses/meta-regression[a]						
						**Study design**	**Excl. age overlap**	**Adjust-ment**	**RoB**	**Sex**	**Age**	**Income**	**SEP**	**SM platform**	**SM category**	**SM content**
Use of ENDS	Time spent on SM	Continuous	Continuous	Std. Beta												
		Continuous	Continuous	Std. mean difference												
		Continuous	Binary	Odds ratio												
		Binary	Binary/ continuous	Odds ratio												
	Frequency of SM use	Continuous	Continuous	Std. Beta												
		Continuous	Continuous	Std. mean difference												
		Continuous	Binary	Odds ratio												
		Binary	Binary/ continuous	Odds ratio												
	Exposure to health-risk behaviour content	Continuous	Continuous	Std. Beta												
		Continuous	Continuous	Std. mean difference												
		Continuous	Binary	Odds ratio												
		Binary	Binary/ continuous	Odds ratio	✓	✓		✓	✓					✓	✓	

Legend: [a] Where ≥10 studies were included in a meta-analysis; meta-regression was conducted. Abbreviations: ENDS = Electronic nicotine delivery systems; RoB = Risk of bias; SM = Social media; and Std. = Standardised.

Appendix 16. Synthesis without meta-analysis (SWiM), meta-analyses, meta-regression, subgroup, and sensitivity analyses

Alcohol use

<u>Effect direction plot</u>

Figure A demonstrates the effect direction in those studies investigating alcohol use, by exposure. Six studies investigated more than one exposure.[56,87,90,114,119,138] For time spent on social media, 15/16 studies (93.8%) reported harmful associations (95% CI 71.7 to 98.9%; participant n=100,354; sign test $p < 0.001$), 16/17 studies (94.1%) reported harmful associations for frequency of social media use (73.0 to 99.0%; participant n=390,843; sign test $p < 0.001$), and 11/12 studies (91.7%) reported harmful associations for exposure to health-risk behaviour content on social media (64.6 to 98.5%; participant n=24,247; sign test p=0.006). Other social media activities was investigated by one study which demonstrated a harmful association (20.7 to 100%; participant n=4,485; insufficient data to conduct sign test).

Figure A. Effect direction plot for studies of the association between social media use and adolescent alcohol use, by social media exposure. Arrow size indicates sample size; arrow colour indicates study risk of bias.

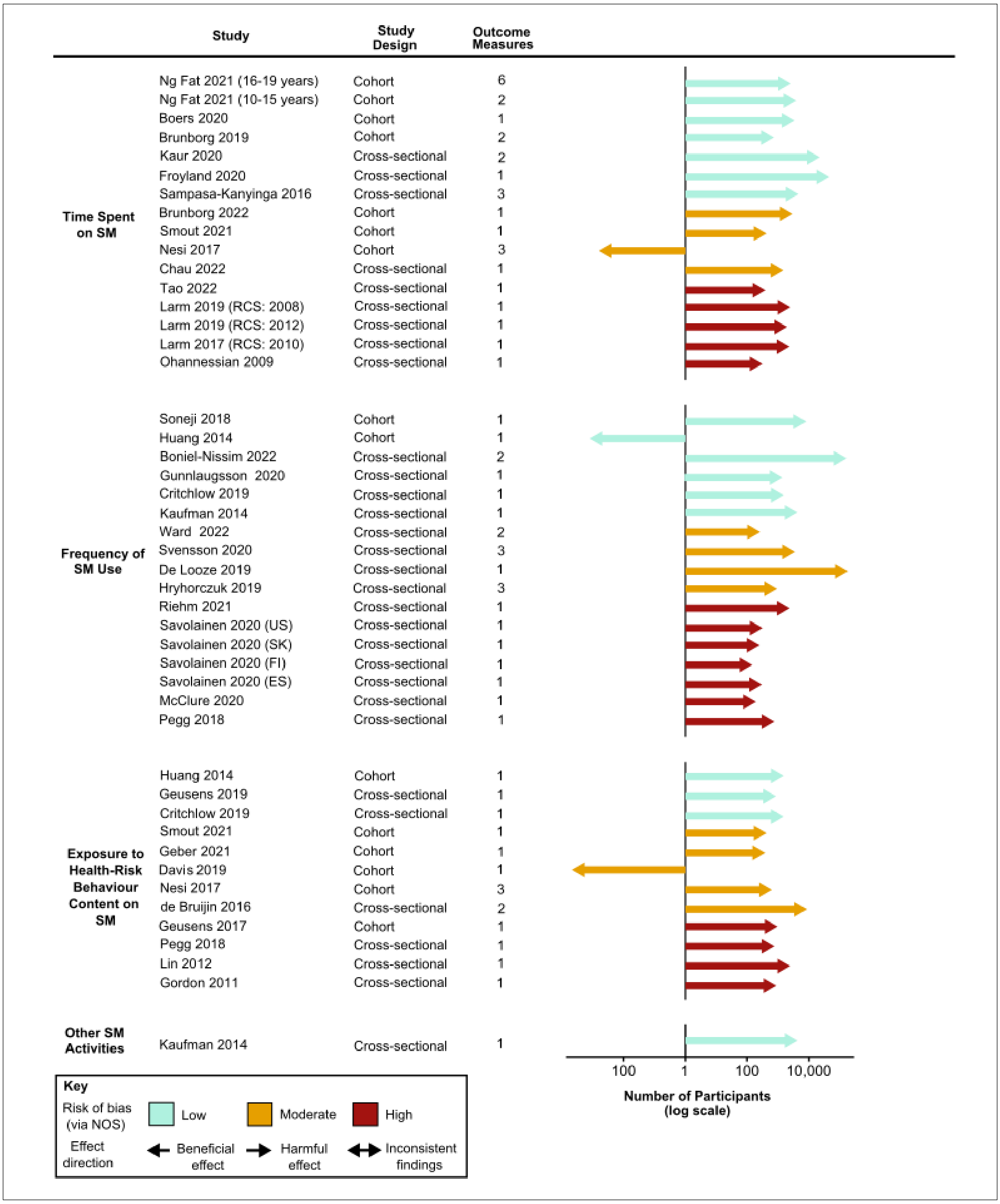

Legend: Sample size: represented by the size of the arrow, measured on a log scale. Outcome measure: number of outcome measures synthesised within each study. Studies organised by risk of bias grade, study design, and year of publication. Repeat cross-sectional studies, multiple study populations from different countries, and age subsets originating from the same study reported as separate studies. Abbreviations: ESP = Spain; FIN = Finland; KOR = South Korea; NOS = Assessed via adapted Newcastle Ottawa Scale; RCS = Repeat cross-sectional study; SM = Social media; and USA = United States.

Forest plots for meta-analyses and subgroup analyses

Figure B. Forest plot for association between frequency of social media use and alcohol use

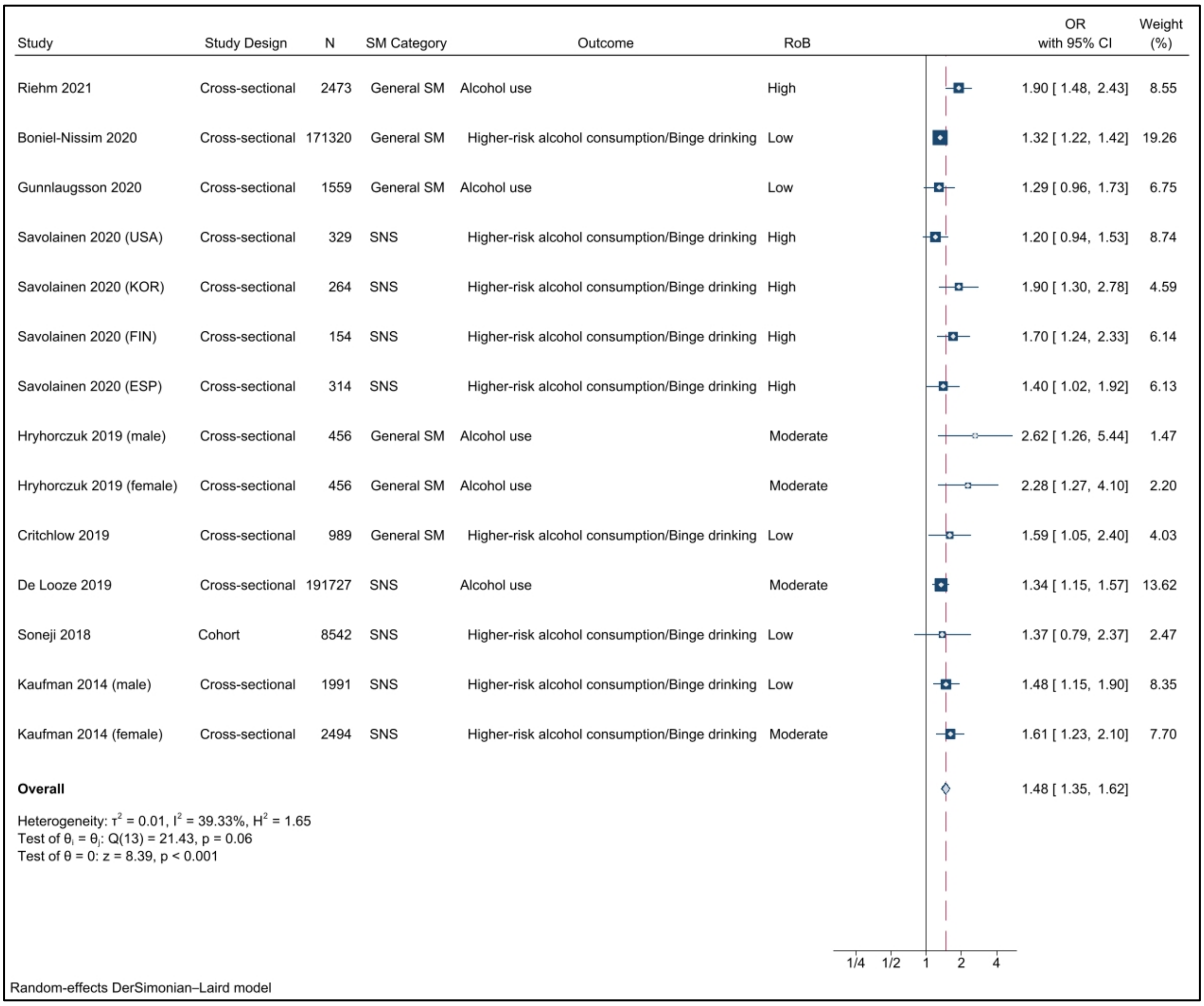

Legend: Figure presents forest plot for binary exposure (frequent/daily vs infrequent/non-daily) & binary/continuous outcome meta-analysis, with odds ratio (OR) used as common metric. Total number of study participants = 383,068. Abbreviations: CI = Confidence interval; ESP = Spain; FIN = Finland; KOR = South Korea; N = Number of study participants; OR = Odds ratio; RoB = Risk of bias; SM = Social media; and SNS = Social networking sites.

Figure C. Forest plot for association between frequency of social media use and alcohol use, stratified by average age of study participants

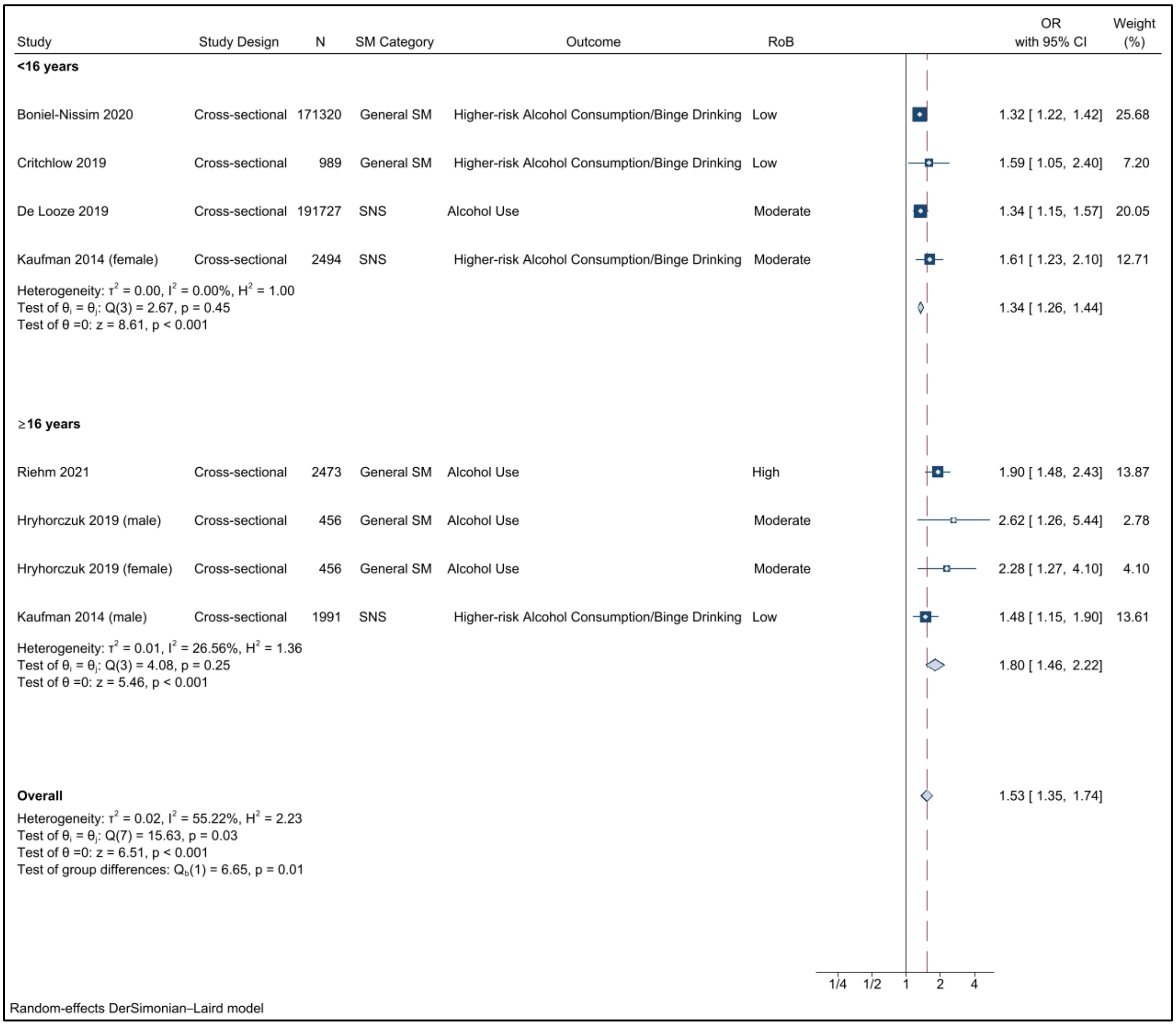

Legend: Figure presents forest plot for binary exposure (frequent/daily vs infrequent/non-daily) & binary/continuous outcome subgroup analysis, with odds ratio (OR) used as common metric. Total number of study participants = 371,906. Abbreviations: CI = Confidence interval; N = Number of study participants; OR = Odds ratio; RoB = Risk of bias; SM = Social media; and SNS = Social networking sites.

Figure D. Forest plot for association between frequency of social media use alcohol use, stratified by development status of study setting[a]

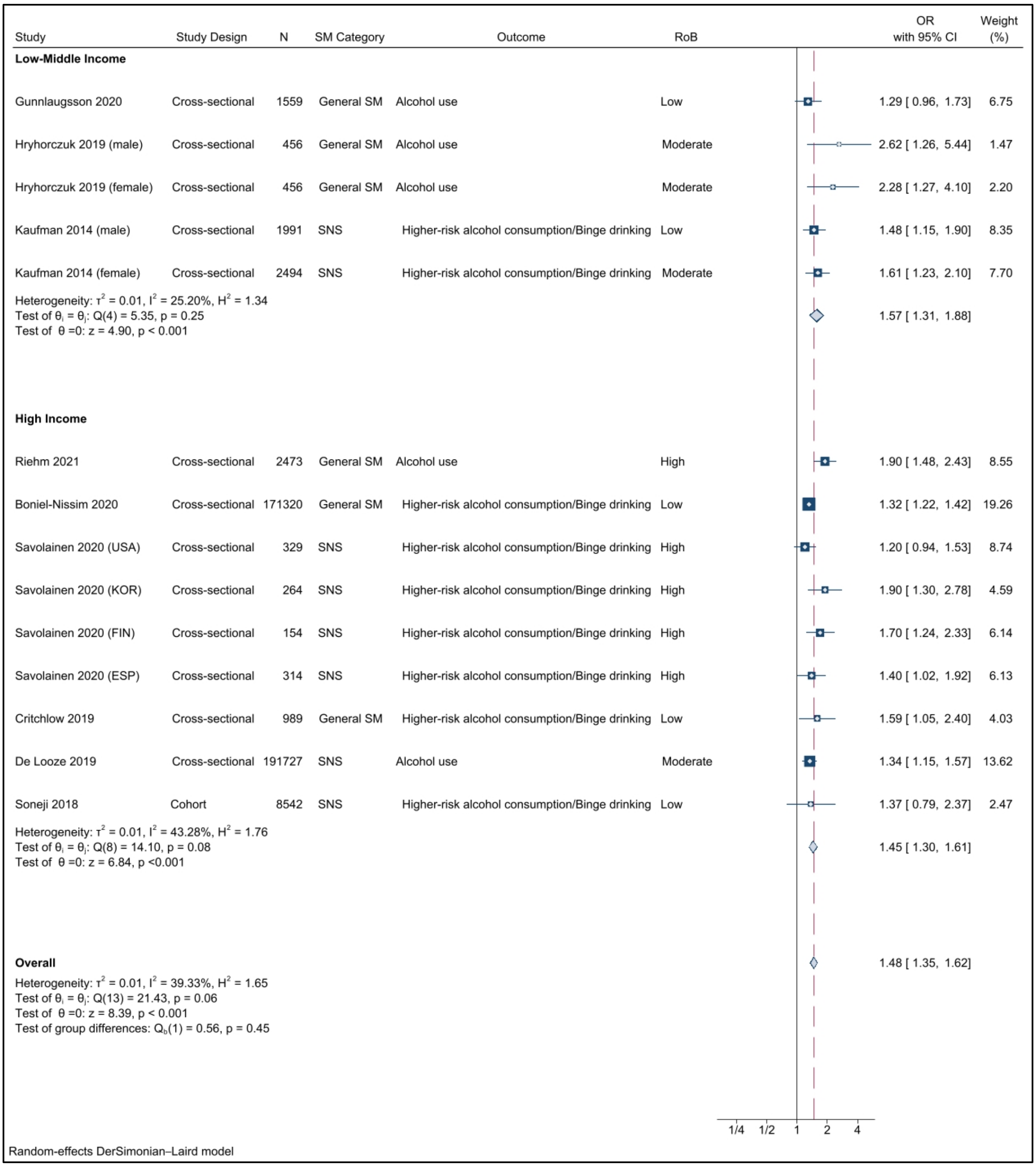

Legend: Figure presents forest plot for binary exposure (frequent/daily vs infrequent/non-daily) & binary/continuous outcome subgroup analysis, with odds ratio (OR) used as common metric. [a] Development status classified as per the World Bank Country Income Level Classification.[160] Total number of study participants = 383,068. Abbreviations: CI = Confidence interval; ESP = Spain; FIN = Finland; KOR = South Korea; N = Number of study participants; OR = Odds ratio; RoB = Risk of bias; SM = Social media; SNS = Social networking sites; and USA = United States.

Figure E. Forest plot for association between frequency of social media use and alcohol use, stratified by social media category

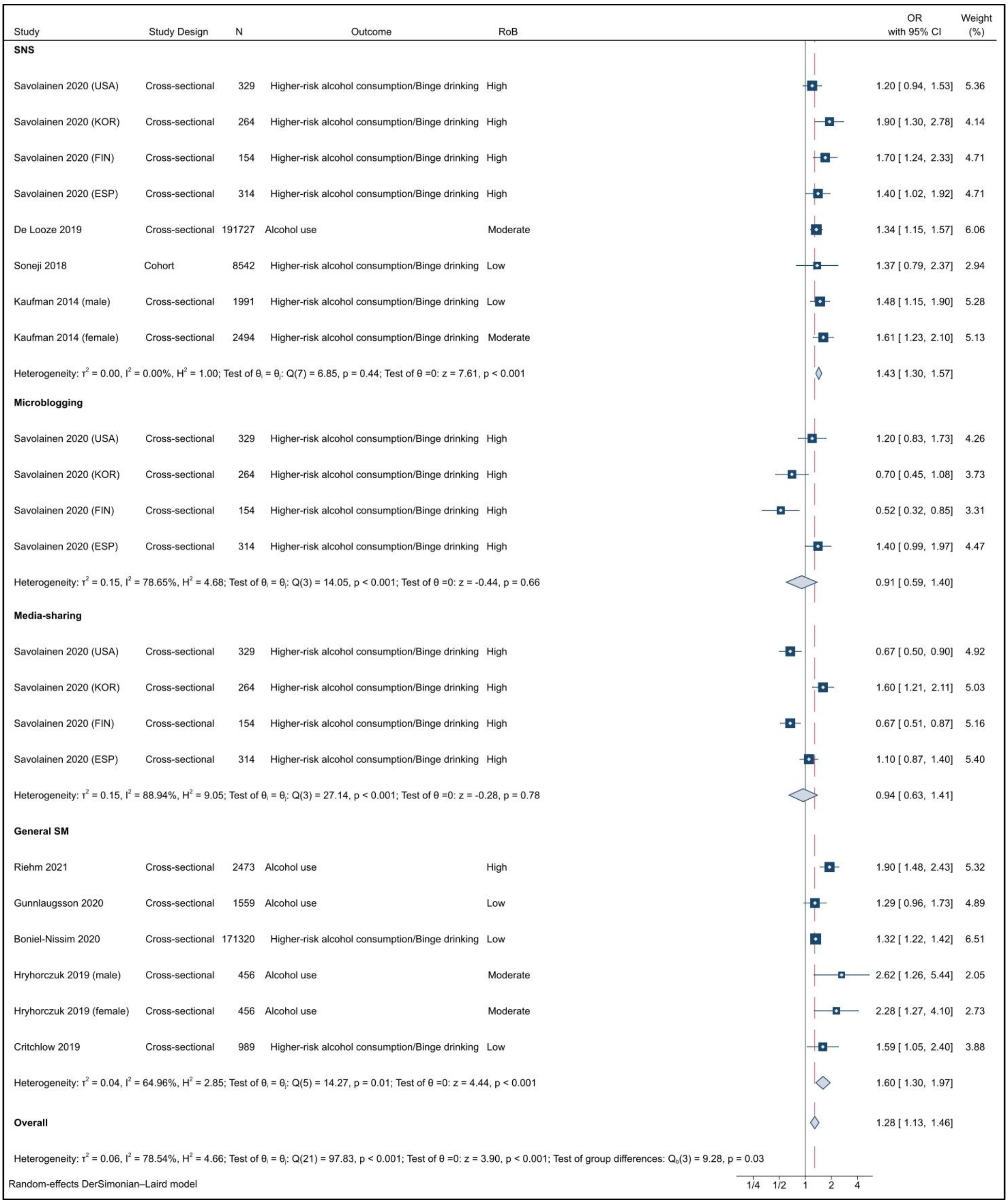

Legend: Figure presents forest plot for binary exposure (frequent/daily vs infrequent/non-daily) & binary/continuous outcome subgroup analysis, with odds ratio (OR) used as common metric. Total number of study participants = 385,190. Abbreviations: CI = Confidence interval; ESP = Spain; FIN = Finland; KOR = South Korea; N = Number of study participants; OR = Odds ratio; RoB = Risk of bias; SM = Social media; and SNS = Social networking sites.

Figure F. Forest plot for association between frequency of social media use and alcohol use, stratified by social media platform

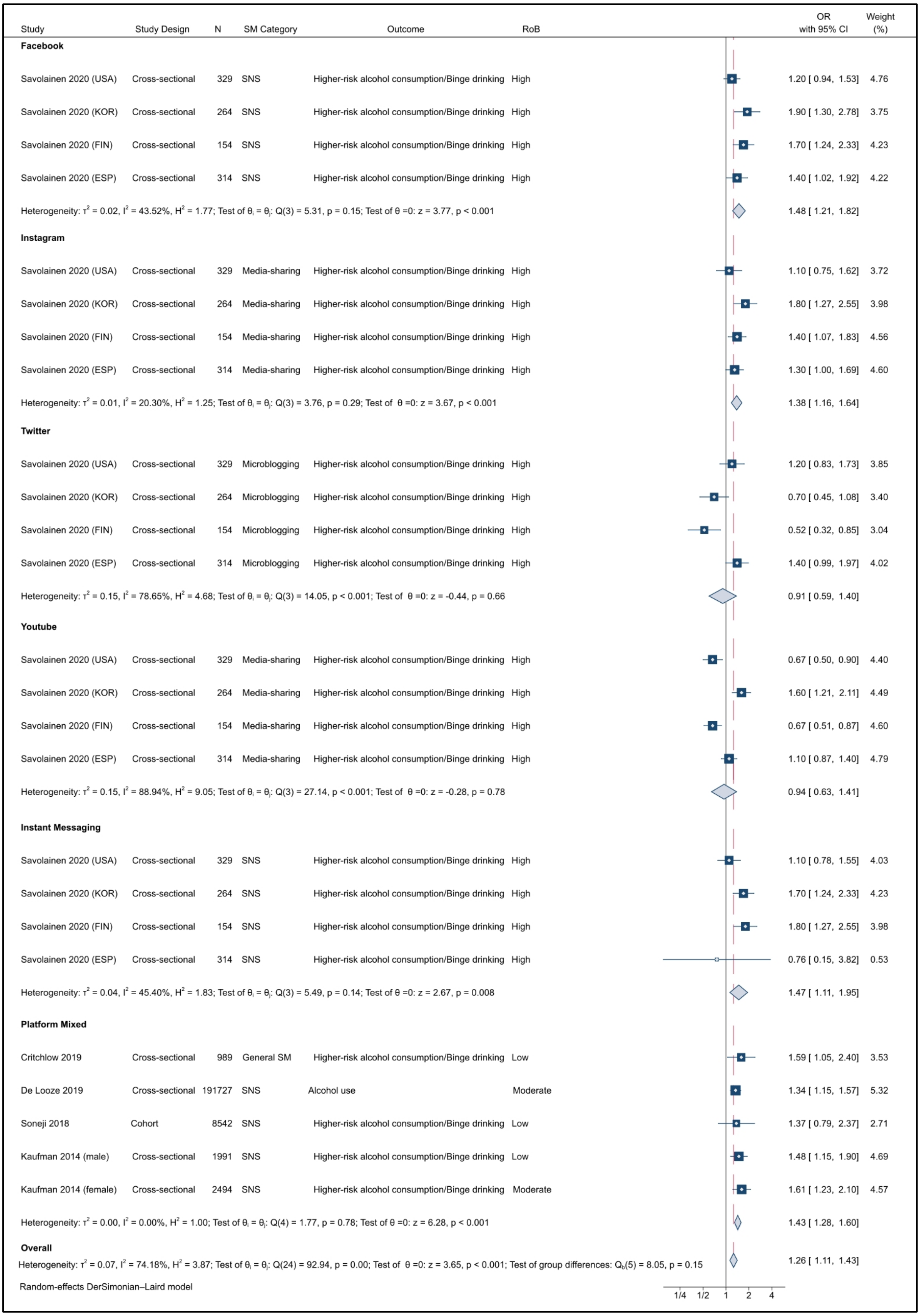

Legend: Figure presents forest plot for binary exposure (frequent/daily vs infrequent/non-daily) & binary/continuous outcome subgroup

analysis, with odds ratio (OR) used as common metric. Total number of study participants = 211,048. Abbreviations: CI = Confidence interval; ESP = Spain; FIN = Finland; KOR = South Korea; N = Number of study participants; OR = Odds ratio; RoB = Risk of bias; SM = Social media; and SNS = Social networking sites.

Figure G. Forest plot for association between frequency of social media use and alcohol use, stratified by sex

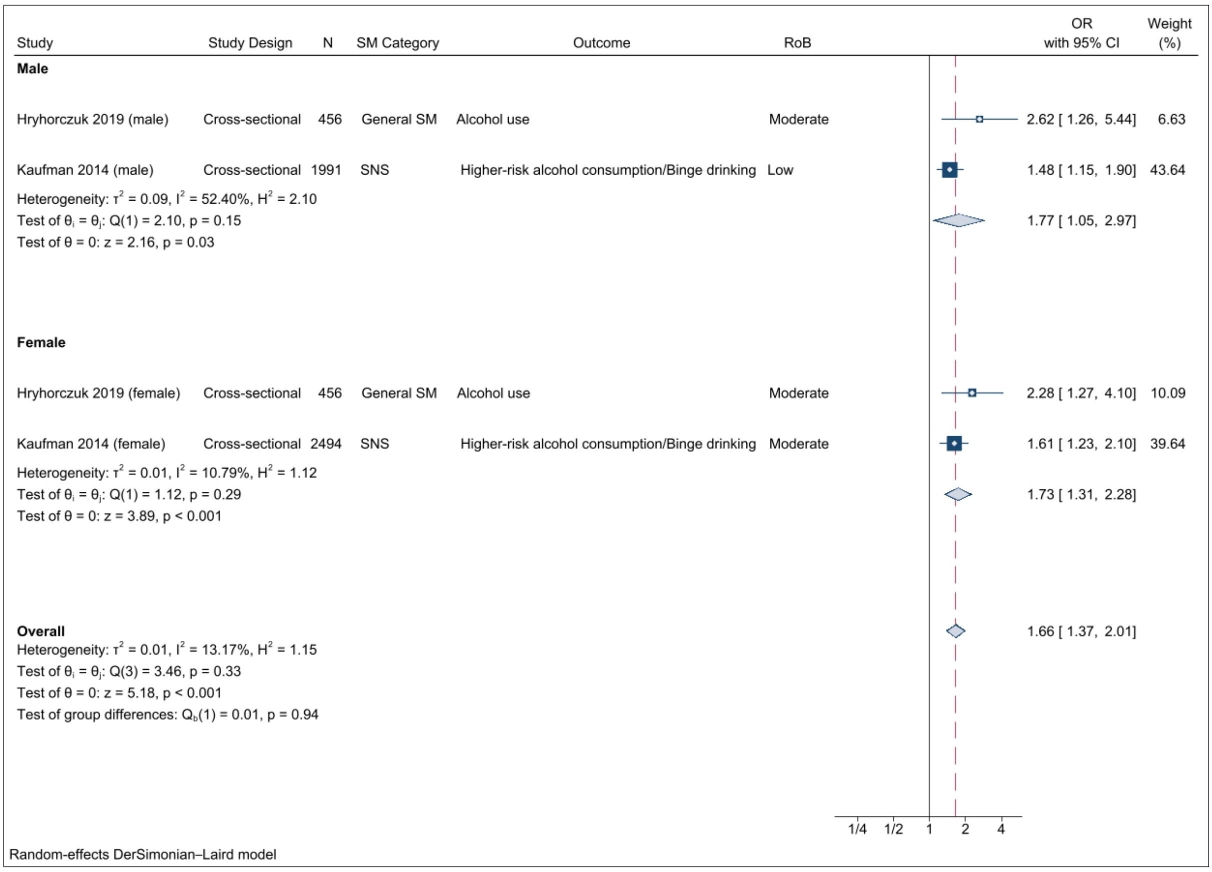

Legend: Figure presents forest plot for binary exposure (frequent/daily vs infrequent/non-daily) & binary/continuous outcome subgroup analysis, with odds ratio (OR) used as common metric. Total number of study participants = 5,397. Abbreviations: CI = Confidence interval; N = Number of study participants; OR = Odds ratio; RoB = Risk of bias; SM = Social media; and SNS = Social networking sites.

Figure H. Forest plot for association between time spent on social media and alcohol use

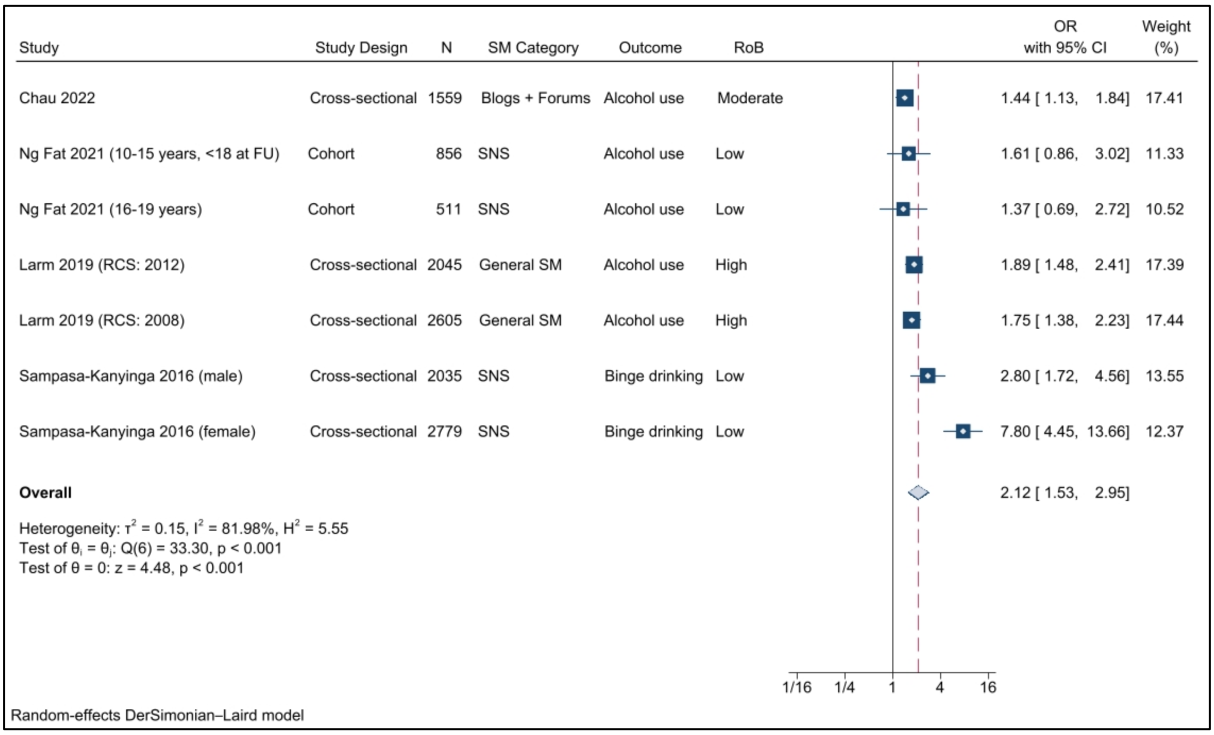

Legend: Figure presents forest plot for binary exposure (≥2 vs <2 hrs/day social media use) & binary/continuous outcome meta-analysis, with odds ratio (OR) used as common metric. Total number of study participants = 12,390. Abbreviations: CI = Confidence interval; FU = Follow up; hrs = Hours; N = Number of study participants; OR = Odds ratio; RCS = Repeat cross-sectional study; RoB = Risk of bias; SM = Social media; and SNS = Social networking sites.

Figure I. Forest plot for association between exposure to health-risk behaviour content on social media and alcohol use

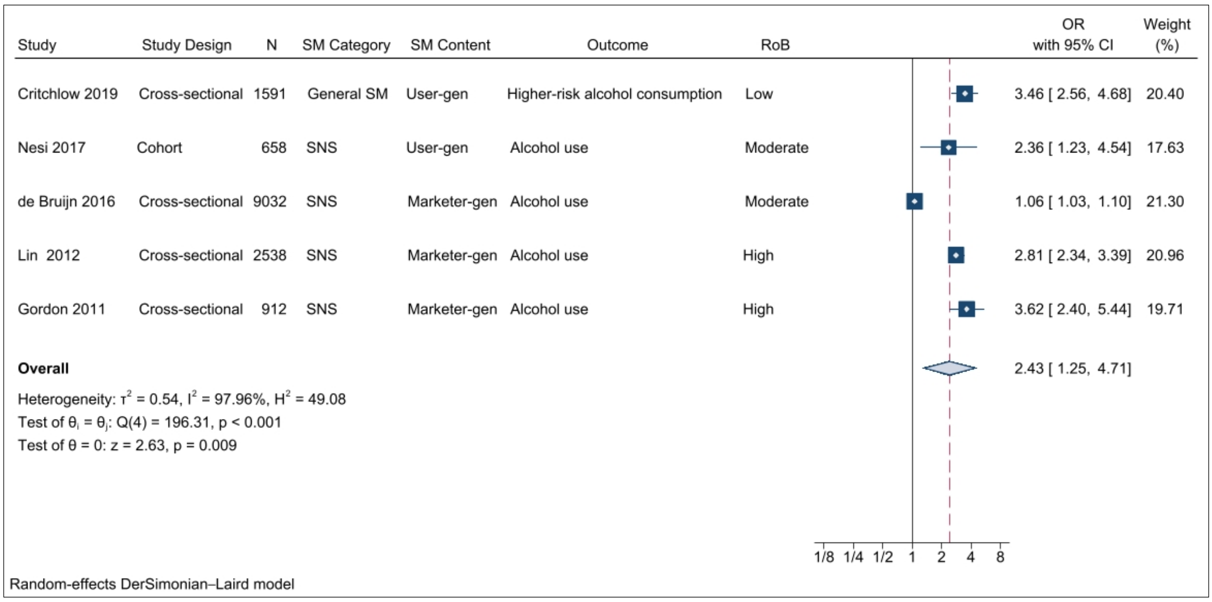

Legend: Figure presents forest plot for binary exposure (exposed vs unexposed) & binary/continuous outcome meta-analysis, with odds ratio (OR) used as common metric. Total number of study participants = 14,731. Abbreviations: CI = Confidence interval; Marketer-gen = Marketer-generated content; N = Number of study participants; OR = Odds ratio; RoB = Risk of bias; SM = Social media; SNS = Social networking sites; and User-gen = User-generated content.

Figure J. Forest plot for association between time spent on social media and alcohol use, by average age of study participants

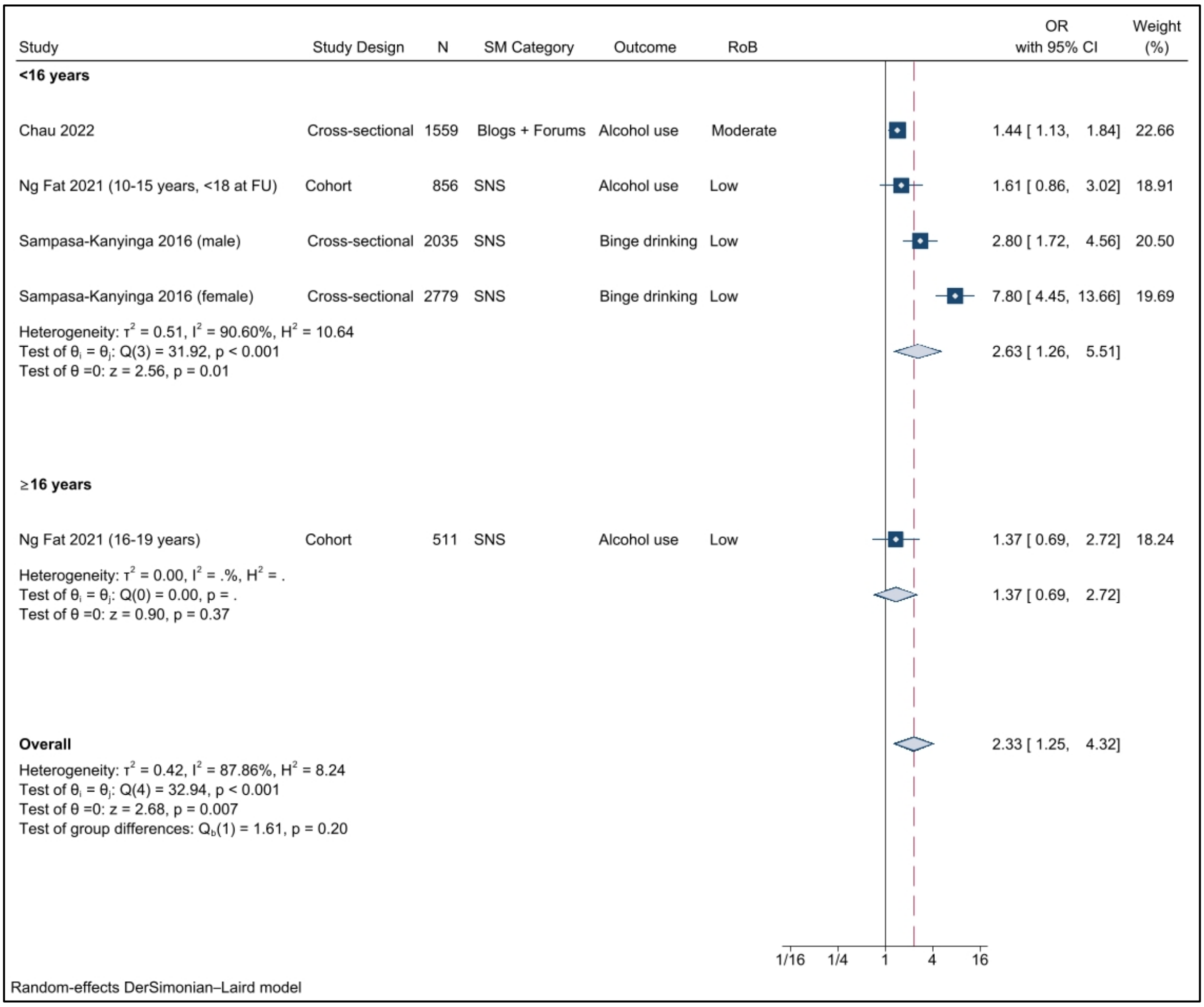

Legend: Figure presents forest plot for binary exposure (≥2 vs <2 hrs/day social media use) & binary/continuous outcome subgroup analysis, with odds ratio (OR) used as common metric. Total number of study participants = 7,740. Abbreviations: CI = Confidence interval; FU = Follow up; hrs = Hours; N = Number of study participants; OR = Odds ratio; RoB = Risk of bias; SM = Social media; and SNS = Social networking sites.

Figure K. Forest plot for association between time spent on social media and alcohol use, by social media category

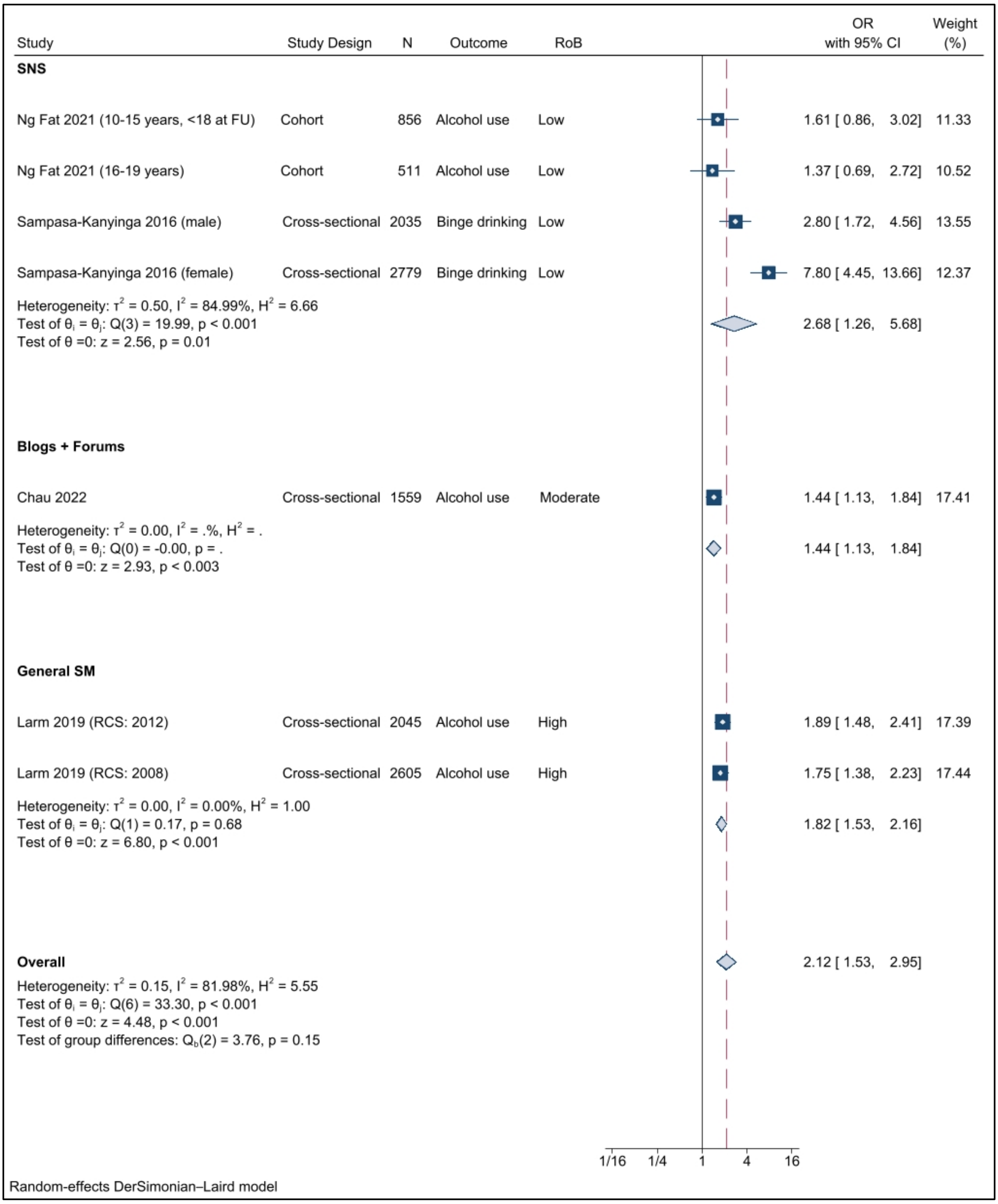

Legend: Figure presents forest plot for binary exposure (≥2 vs <2 hrs/day social media use) & binary/continuous outcome sensitivity analysis, with odds ratio (OR) used as common metric. Total number of study participants = 12,390. Abbreviations: CI = Confidence interval; FU = Follow up; hrs = Hours; N = Number of study participants; OR = Odds ratio; RCS = Repeat cross-sectional study; RoB = Risk of bias; SM = Social media; and SNS = Social networking sites.

Figure L. Forest plot for association between exposure to health-risk behaviour content on social media and alcohol use, by social media category

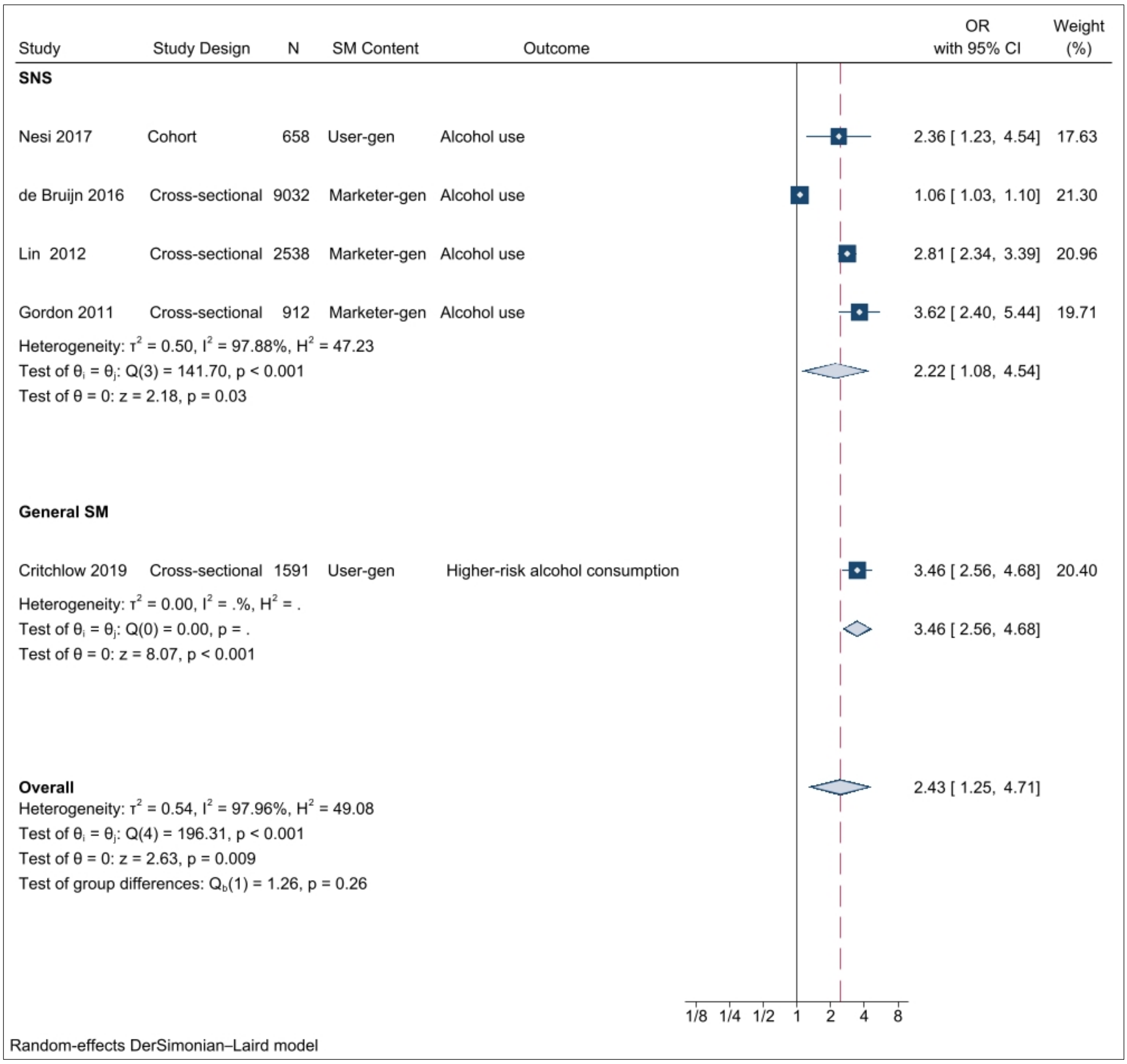

Legend: Figure presents forest plot for binary exposure (exposed vs unexposed) & binary/continuous outcome subgroup analysis, with odds ratio (OR) used as common metric. Total number of study participants = 14,731. Abbreviations: CI = Confidence interval; Marketer-gen = Marketer-generated content; N = Number of study participants; OR = Odds ratio; RoB = Risk of bias; SM = Social media; SNS = Social networking sites; and User-gen = User-generated content.

Figure M. Forest plot for association between exposure to health-risk behaviour content on social media and alcohol use, by social media content

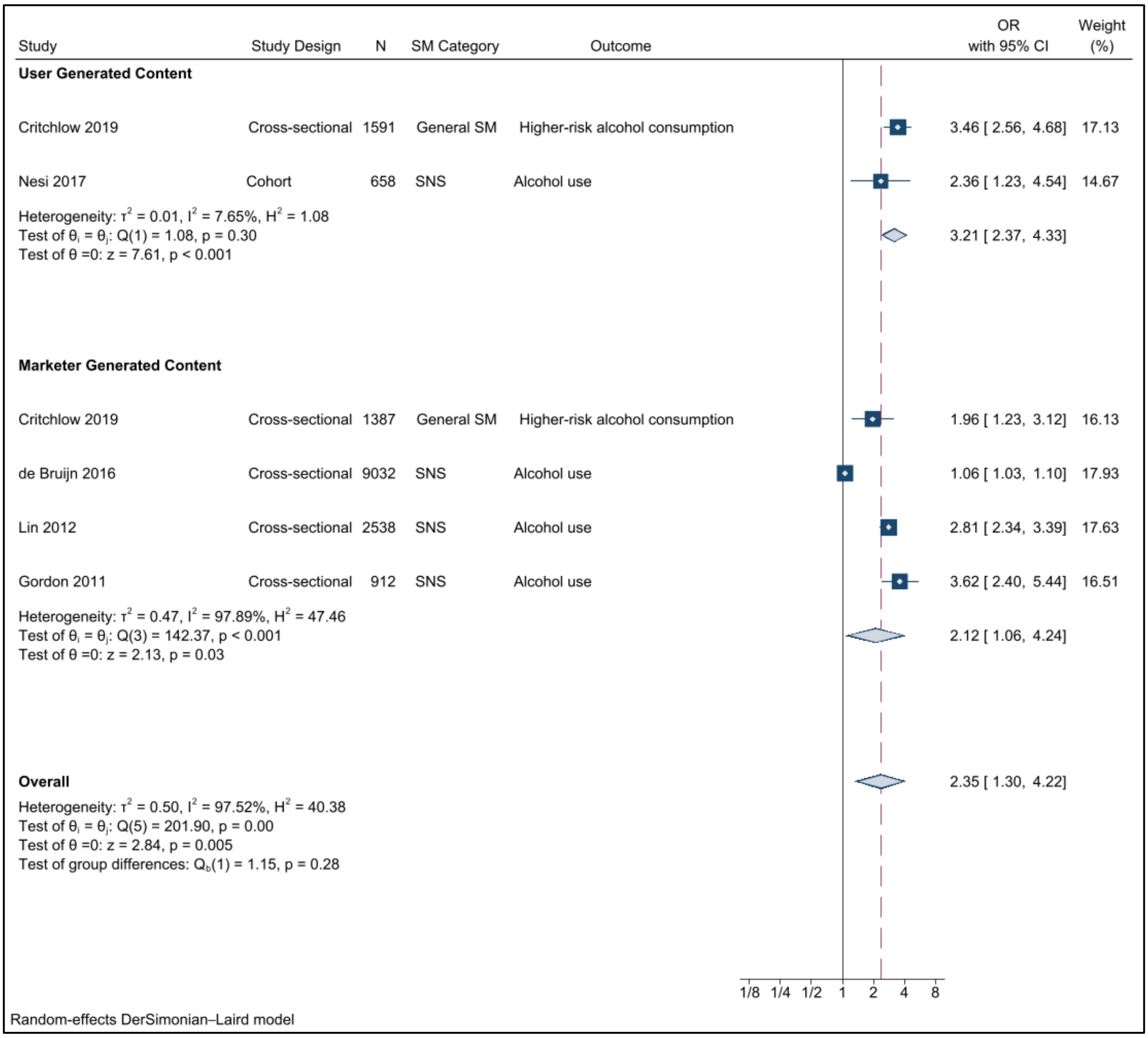

Legend: Figure presents forest plot for binary exposure (exposed vs unexposed) & binary/continuous outcome subgroup analysis, with odds ratio (OR) used as common metric. Total number of study participants = 16,118. Abbreviations: CI = Confidence interval; N = Number of study participants; OR = Odds ratio; RoB = Risk of bias; SM = Social media; and SNS = Social networking sites.

Figure N. Forest plot for association between frequency of social media use and alcohol use

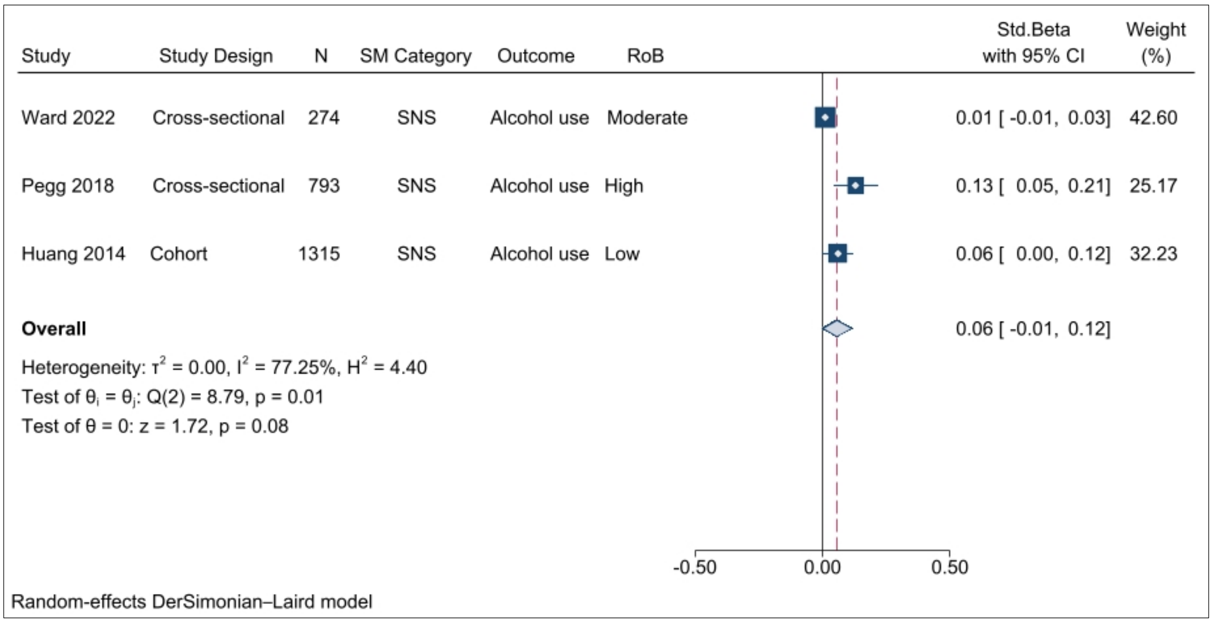

Legend: Figure presents forest plot for continuous exposure & continuous outcome meta-analysis, with standardised beta (Std. Beta) used as common metric. Total number of study participants = 2,382. Abbreviation: CI = Confidence interval; N = Number of study participants; RoB = Risk of bias; SM = Social media; SNS = Social networking sites; and Std. Beta = Standardised beta.

Figure O. Forest plot for association between time spent on social media and alcohol use

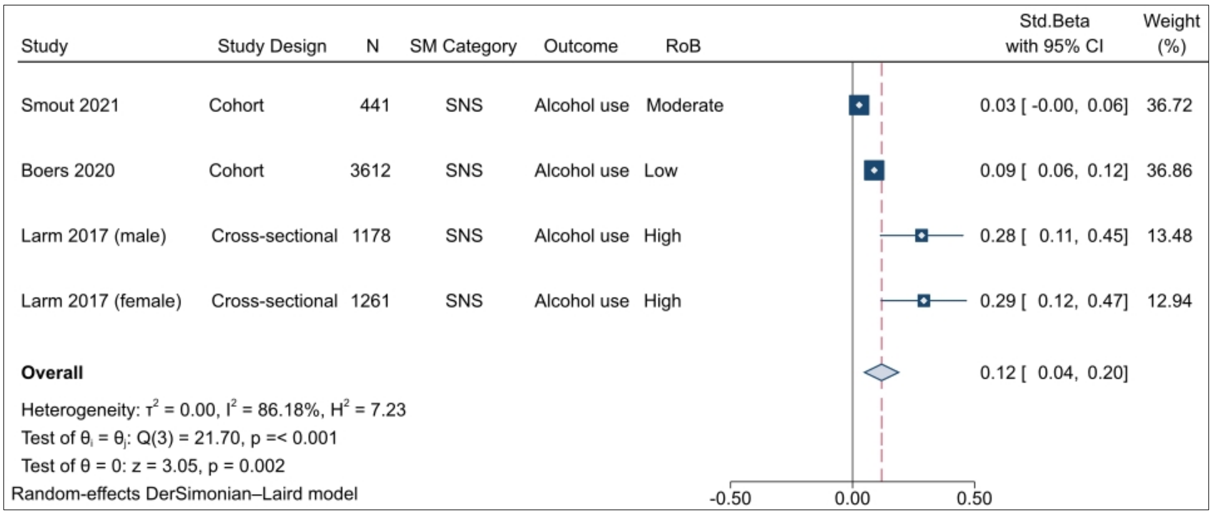

Legend: Figure presents forest plot for continuous exposure & continuous outcome meta-analysis, with standardised beta (Std. Beta) used as common metric. Total number of study participants = 6,492. Abbreviation: CI = Confidence interval; N = Number of study participants; RoB = Risk of bias; SM = Social media; SNS = Social networking sites; and Std. Beta = Standardised beta.

Figure P. Forest plot for association between time spent on social media and alcohol use

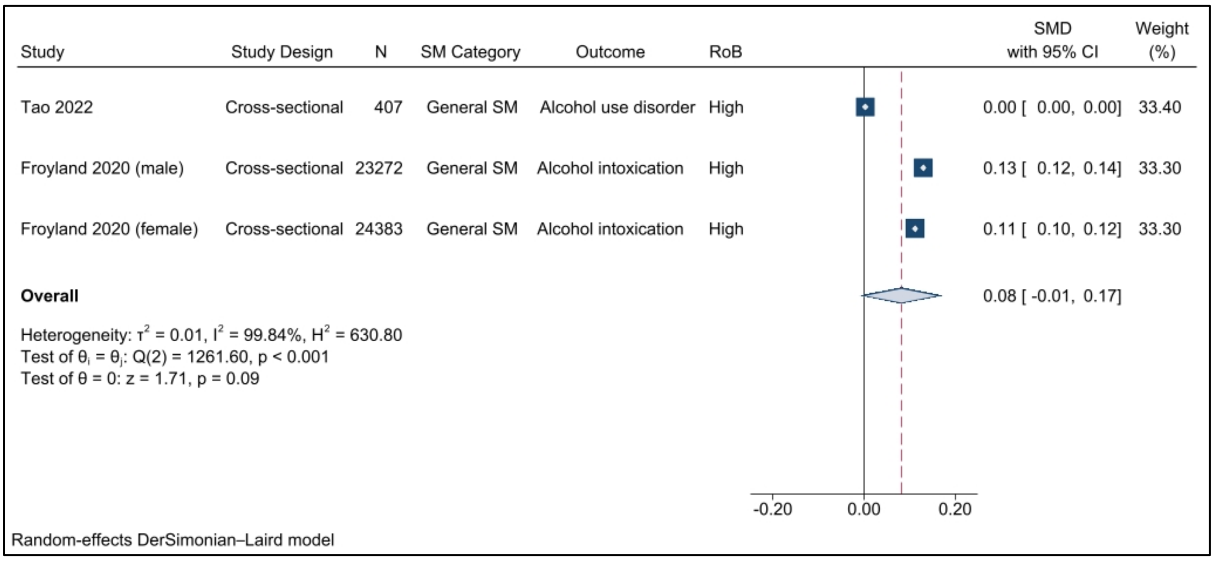

Legend: Figure presents forest plot for continuous exposure & continuous outcome meta-analysis, with standardised mean difference (SMD) used as common metric. Total number of study participants = 48,062. Abbreviation: CI = Confidence interval; N = Number of study participants; RoB = Risk of bias; SM = Social media; SMD = Standardised mean difference; and SNS = Social networking sites.

Figure Q. Forest plot for associations between exposure to health-risk behaviour content on social media and alcohol use

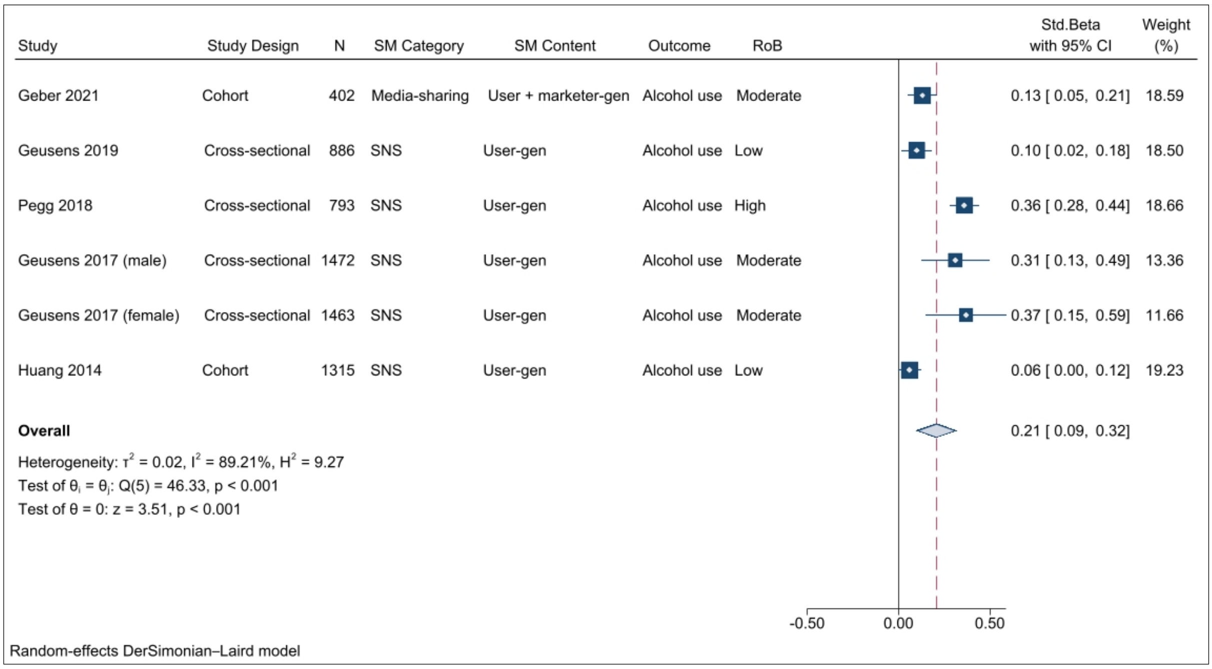

Legend: Figure presents forest plot for continuous exposure & continuous outcome meta-analysis, with standardised beta (Std.Beta) used as common metric. Total number of study participants = 6,331. Abbreviation: CI = Confidence interval; N = Number of study participants; Marketer-gen = Marketer-generated content; RoB = Risk of bias; SM = Social media; SNS = Social networking sites; Std. Beta = Standardised beta; and User-gen = User-generated content.

Figure R. Forest plot for association between exposure to health-risk behaviour content on social media and alcohol use, by average age of study participants

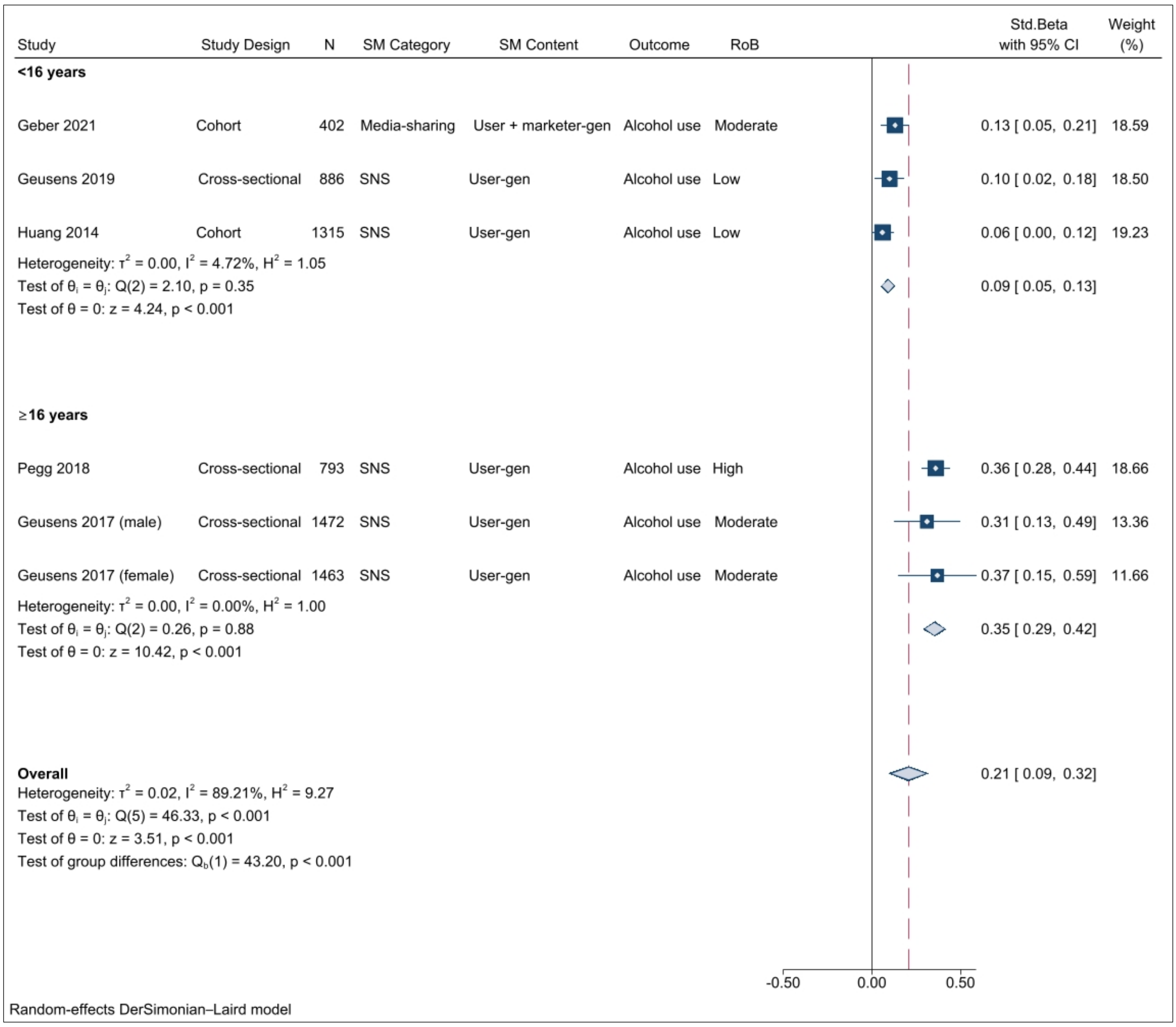

Legend: Figure presents forest plot for continuous exposure & continuous outcome subgroup analysis, with standardised beta (Std. Beta) used as common metric. Total number of study participants = 6,331. Abbreviation: CI = Confidence interval; N = Number of study participants; Marketer-gen = Marketer-generated content; RoB = Risk of bias; SM = Social media; SNS = Social networking sites; Std. Beta = Standardised beta; and User-gen = User-generated content.

Figure S. Forest plot for association between frequency of social media use and alcohol use, by average age of study participants

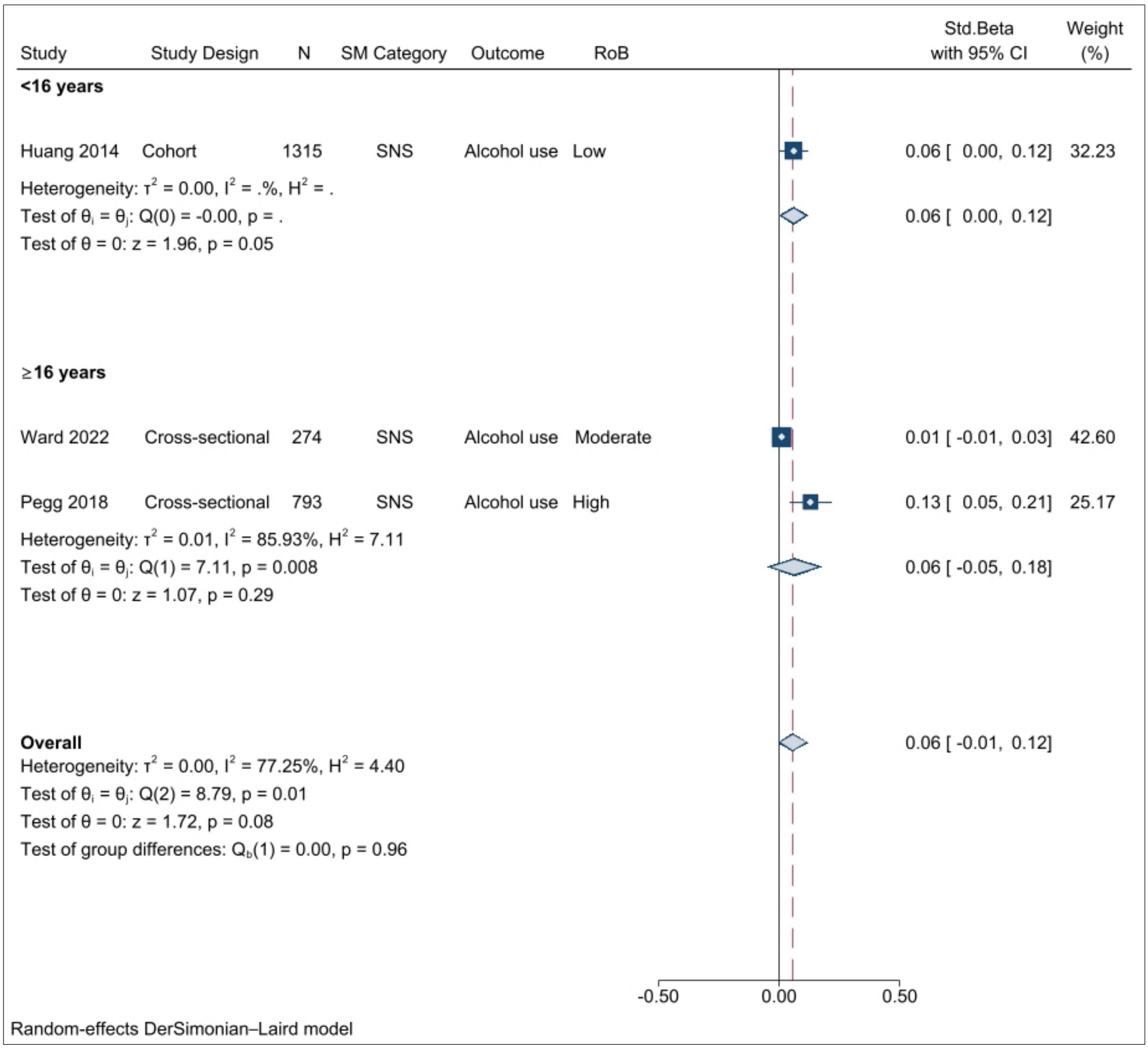

Legend: Figure presents forest plot for continuous exposure & continuous outcome subgroup analysis, with standardised beta (Std. Beta) used as common metric. Total number of study participants = 2,382. Abbreviation: CI = Confidence interval; N = Number of study participants; RoB = Risk of bias; SM = Social media; SNS = Social networking sites; and Std. Beta = Standardised beta.

Figure T. Forest plot for association between frequency of social media use and alcohol use, by social media category

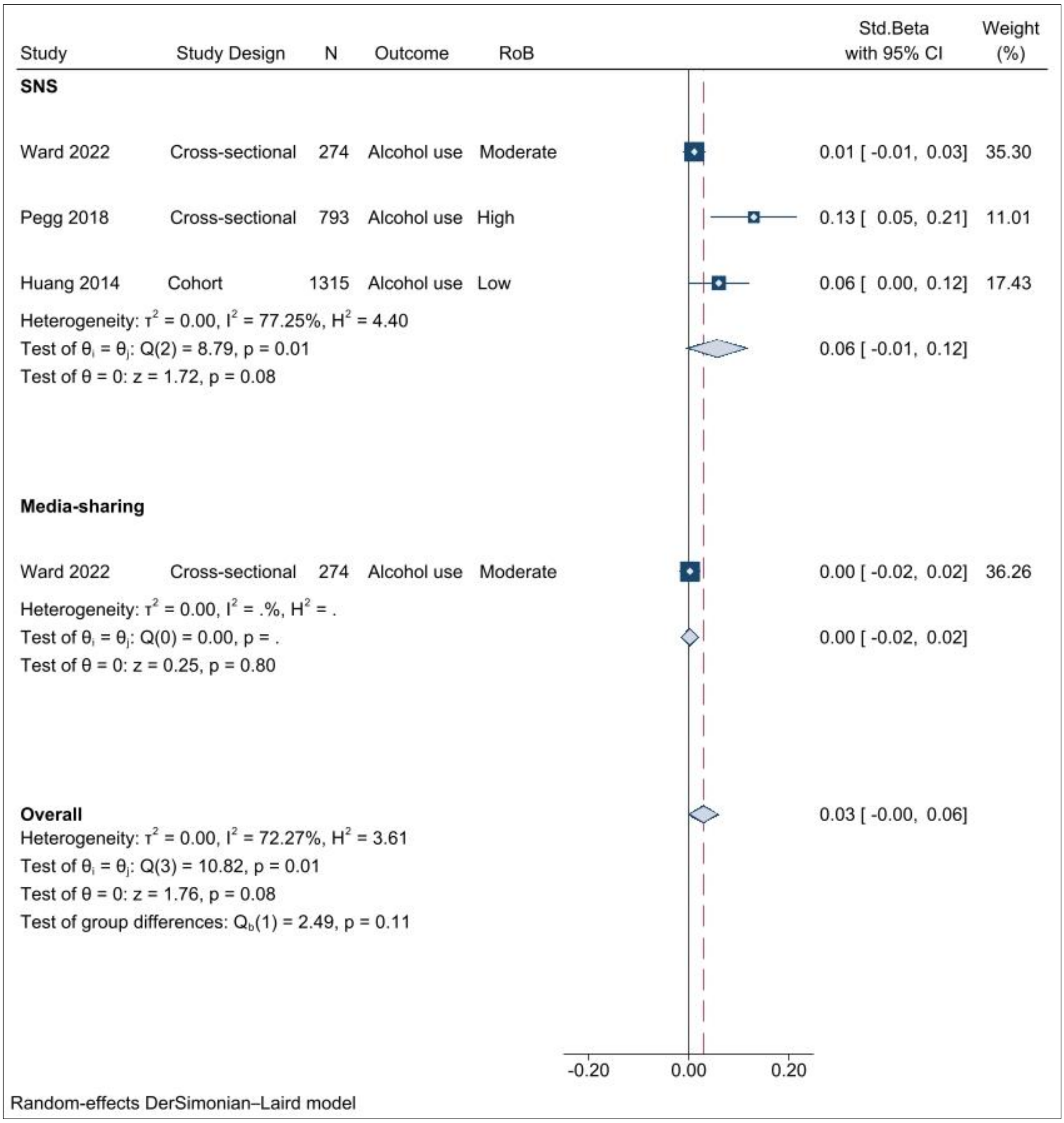

Legend: Figure presents forest plot for continuous exposure & continuous outcome subgroup analysis, with standardised beta (Std. Beta) used as common metric. Total number of study participants = 2,656. Abbreviation: CI = Confidence interval; N = Number of study participants; RoB = Risk of bias; SM = Social media; SNS = Social networking sites; and Std. Beta = Standardised beta.

Figure U. Forest plot for association between exposure to health-risk behaviour content on social media and alcohol use, by average socioeconomic position of study participants

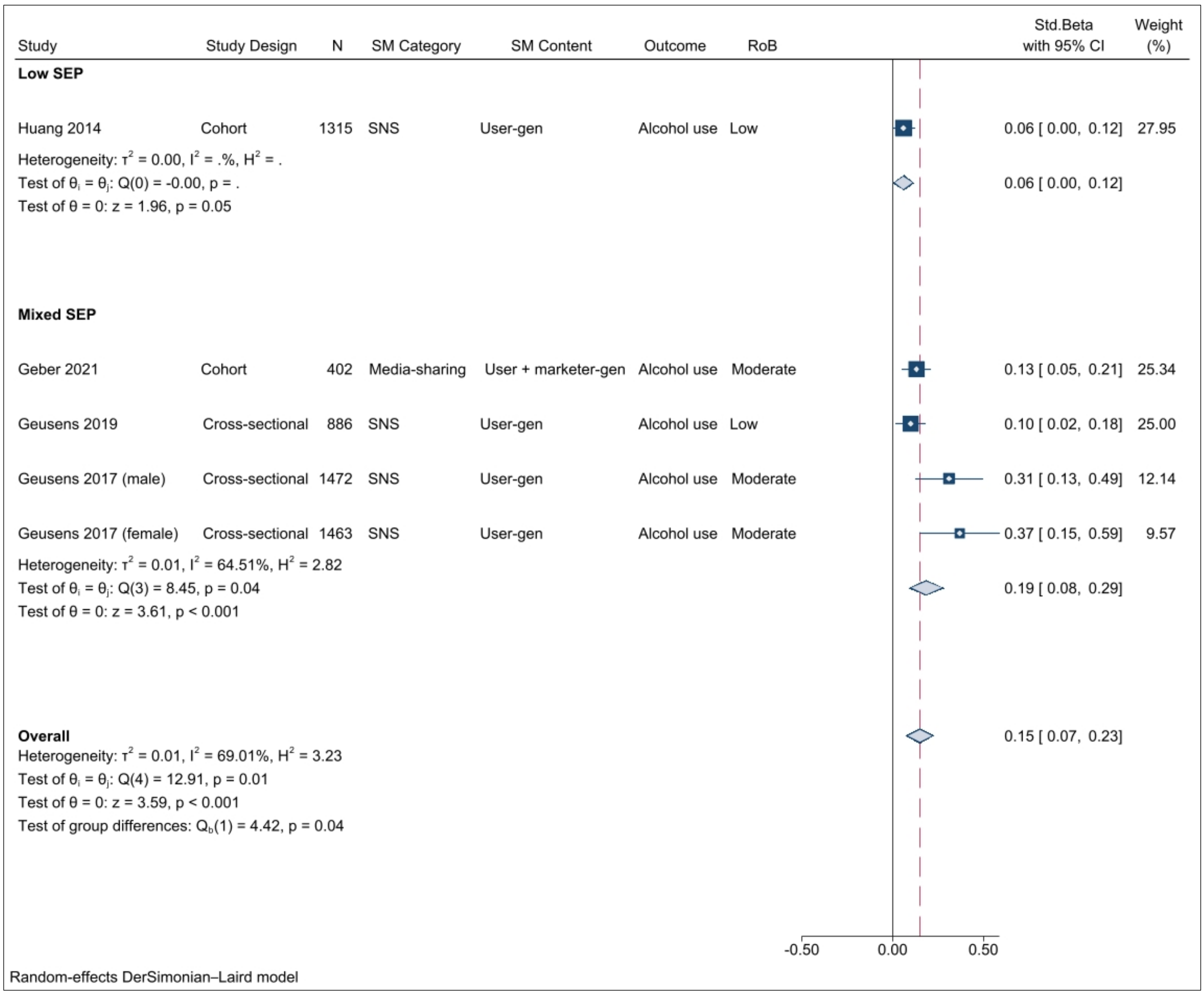

Legend: Figure presents forest plot for continuous exposure & continuous outcome subgroup analysis, with standardised beta (Std.Beta) used as common metric. Total number of study participants = 5,538. Abbreviation: CI = Confidence interval; N = Number of study participants; RoB = Risk of bias; SEP = Socioeconomic position; SM = Social media; SNS = Social networking sites; Std. Beta = Standardised beta; and User-gen = User-generated content.

Figure V. Forest plot for association between exposure to health-risk behaviour content on social media and alcohol use, by social media category

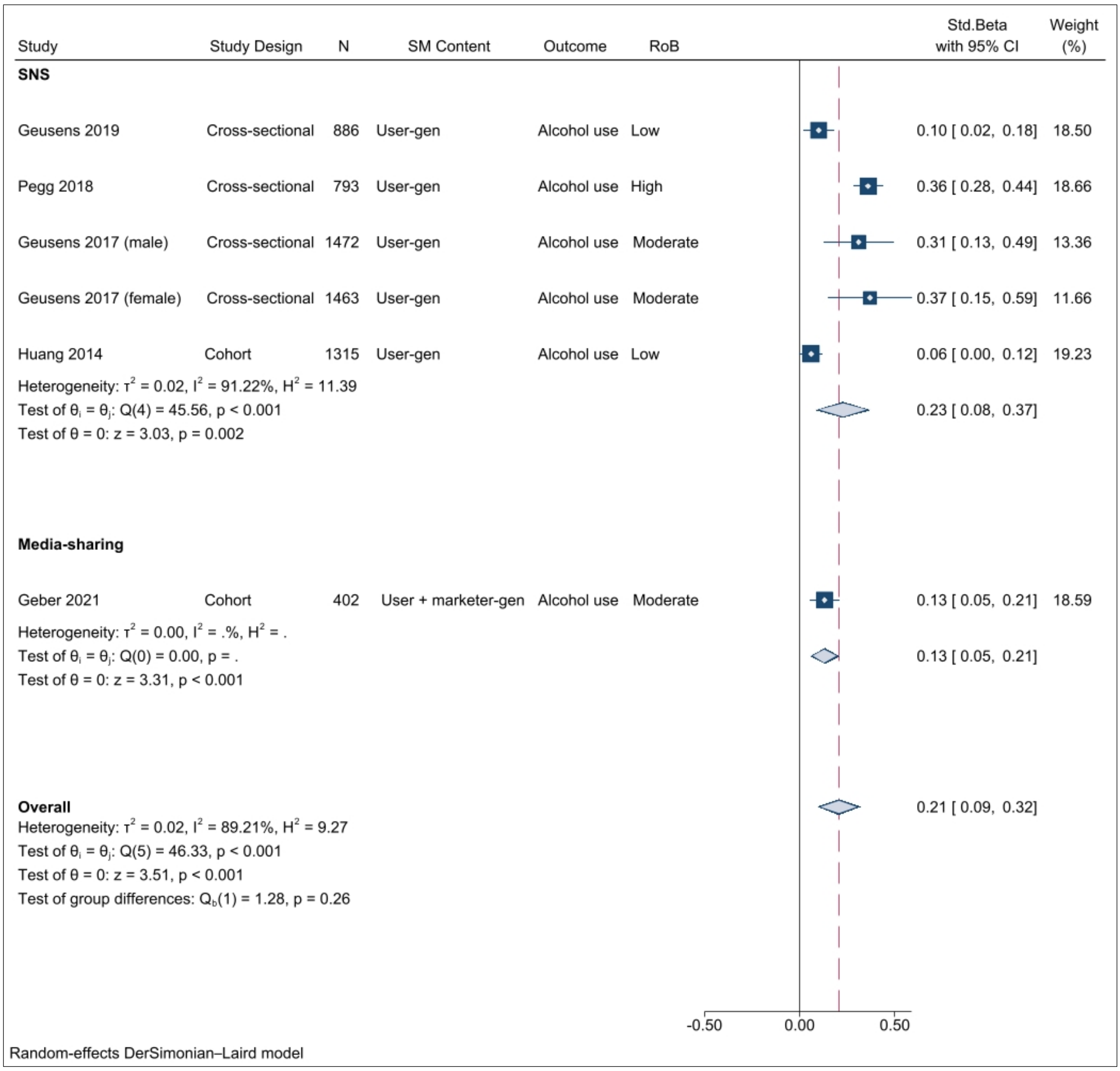

Legend: Figure presents forest plot for continuous exposure & continuous outcome subgroup analysis, with standardised beta (Std.Beta) used as common metric. Total number of study participants = 6,331. Abbreviation: CI = Confidence interval; N = Number of study participants; Marketer-gen = Marketer-generated content; RoB = Risk of bias; SM = Social media; SNS = Social networking sites; Std. Beta = Standardised beta; and User-gen = User-generated content.

Drug use

Effect direction plot

Figure W demonstrates the effect direction in those studies (n=13) investigating drug use, by exposure. Two studies investigated more than one exposure.[124,153] For time spent on social media, 5/6 studies (83.3%) reported harmful associations (95% CI 43.6 to 97.0%; participant n=727,788; sign test p=0.22). For frequency of social media use 5/6 studies (83.3%) demonstrated harmful associations (43.6 to 97.0%; participant n=117,645; sign test p=0.22) and for exposure to health-risk behaviour content on social media all studies demonstrated harmful associations (34.2 to 100.0%; study n=2; participant n=1,268; insufficient data to conduct sign test). Other social media activities was investigated by one study which demonstrated a harmful association (20.7 to 100.0%; participant n=786; insufficient data to conduct sign test).

Figure W. Effect direction plot for studies of the association between social media use and adolescent drug use, by social media exposure. Arrow size indicates sample size; arrow colour indicates study risk of bias.

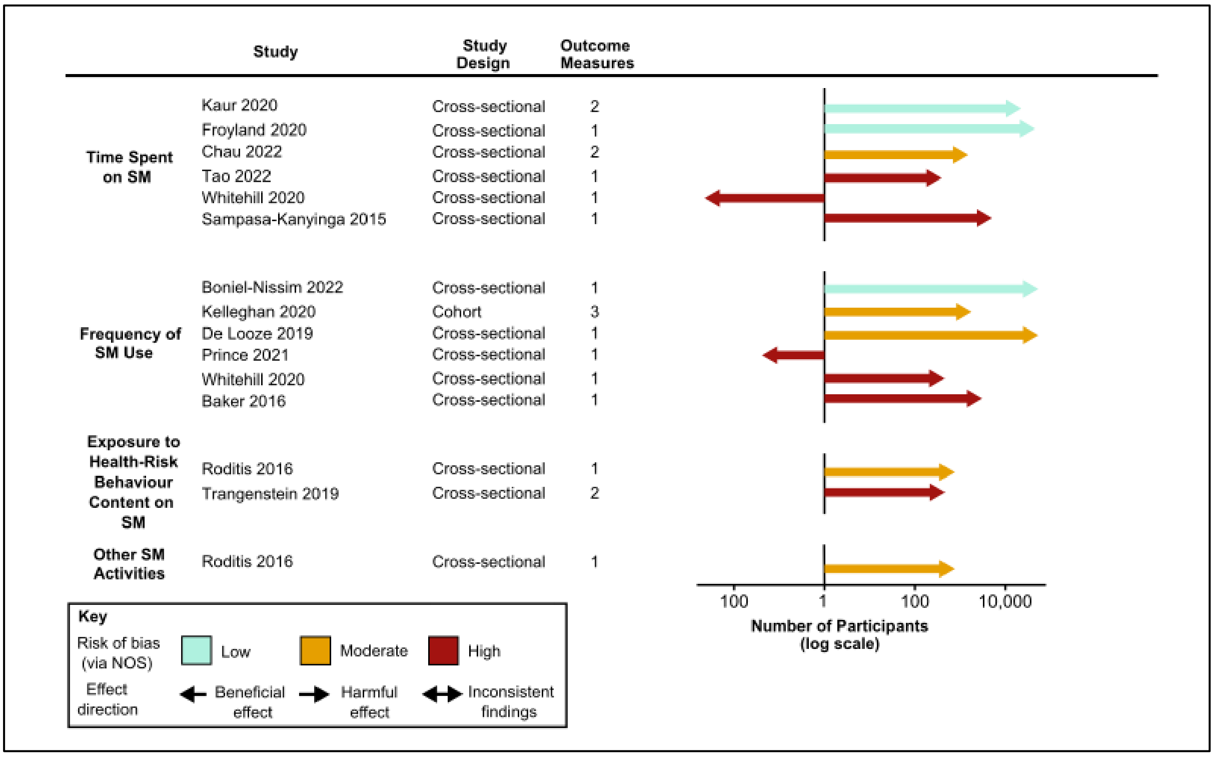

Legend: Sample size: represented by the size of the arrow, measured on a log scale. Outcome measure: number of outcome measures synthesised within each study. Studies organised by risk of bias grade, study design, and year of publication. Repeat cross-sectional studies, multiple study populations from different countries, and age subsets originating from the same study reported as separate studies. Abbreviations: NOS = Assessed via adapted Newcastle Ottawa Scale; and SM = Social media.

Forest plots for meta-analyses and subgroup analyses

Figure X. Forest plot for association between frequency of social media use and drug use

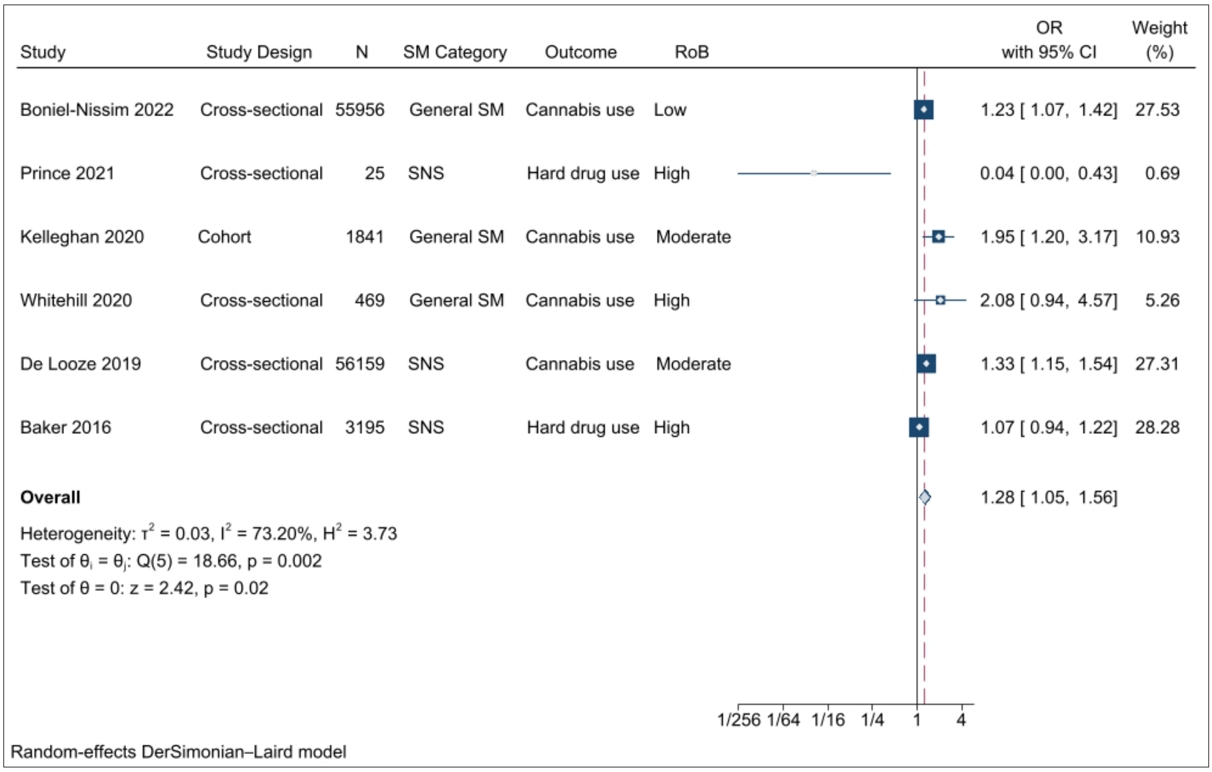

Legend: Figure presents forest plot for binary exposure (frequent/daily vs infrequent/non-daily) & binary/continuous outcome meta-analysis, with odds ratio (OR) used as common metric. Total number of study participants = 117,645. Abbreviations: CI = Confidence interval; N = Number of study participants; OR = Odds ratio; RoB = Risk of bias; SM = Social media; and SNS = Social networking sites.

Figure Y. Forest plot for association between frequency of social media use and drug use, by average age of study participants

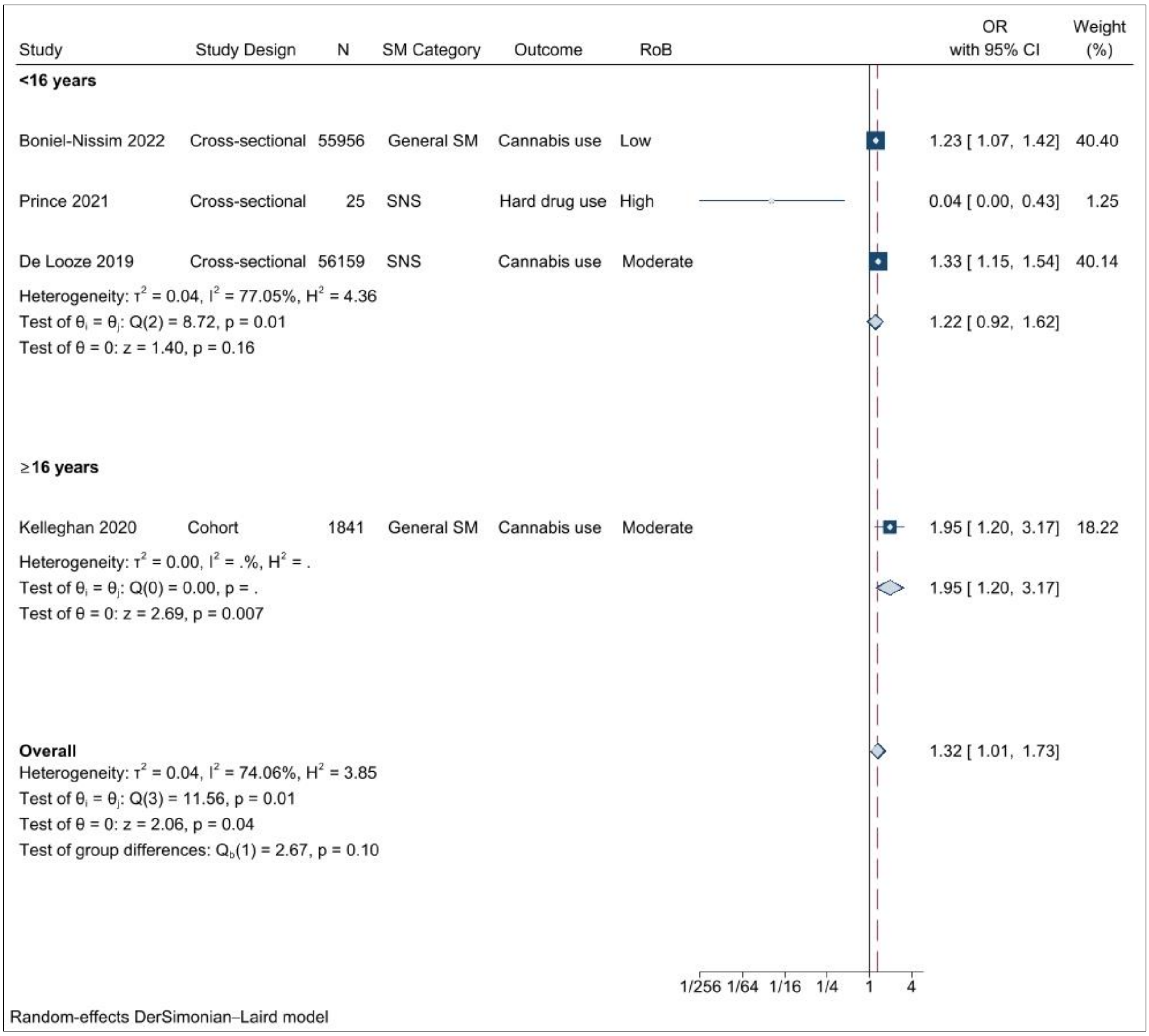

Legend: Figure presents forest plot for binary exposure (frequent/daily vs infrequent/non-daily) & binary/continuous outcome meta-analysis, with odds ratio (OR) used as common metric. Total number of study participants = 113,981. Abbreviations: CI = Confidence interval; N = Number of study participants; OR = Odds ratio; RoB = Risk of bias; SM = Social media; and SNS = Social networking sites.

Figure Z. Forest plot for association between frequency of social media use and drug use, by social media platform

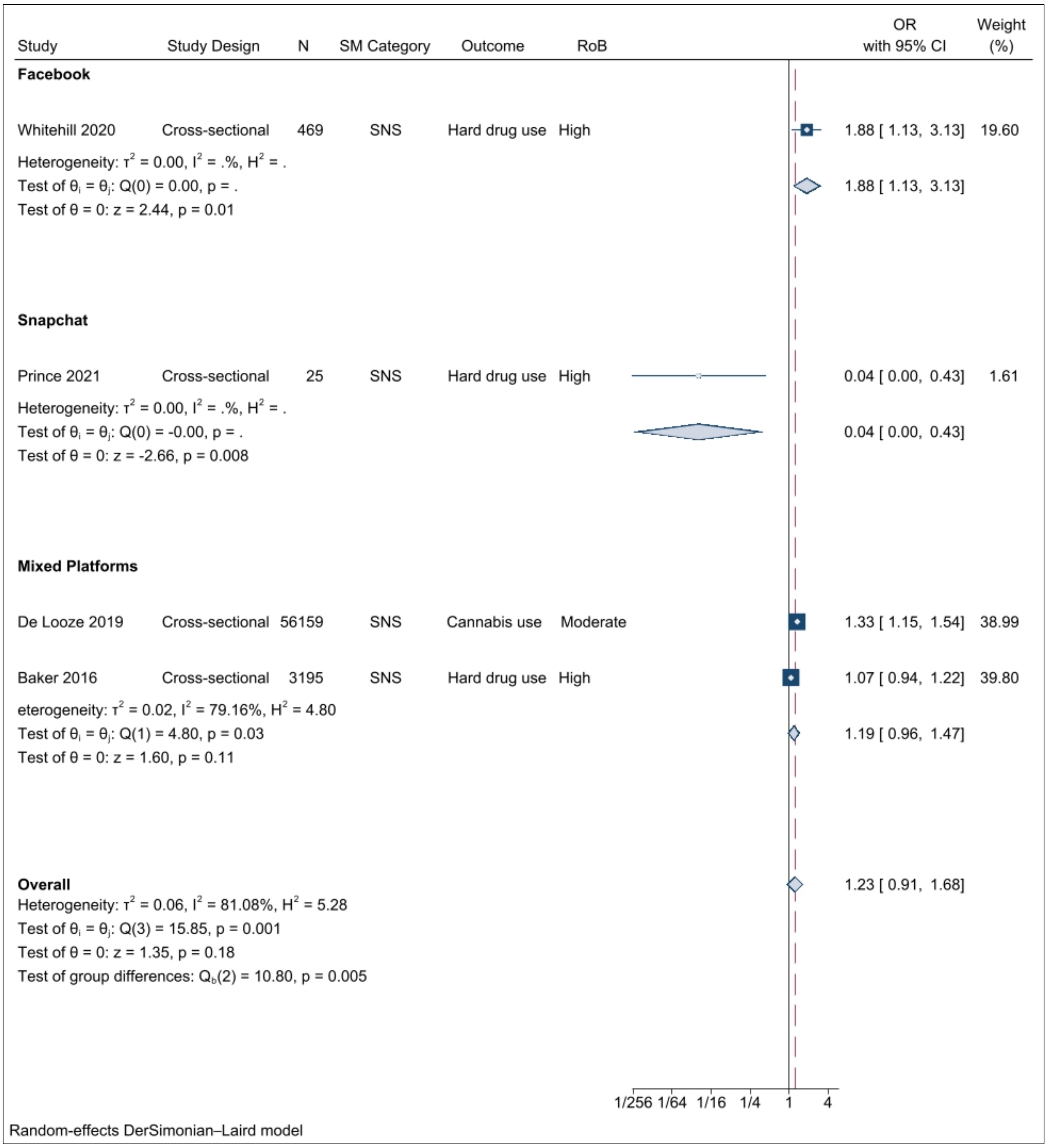

Legend: Figure presents forest plot for binary exposure (frequent/daily vs infrequent/non-daily) & binary/continuous outcome meta-analysis, with odds ratio (OR) used as common metric. Total number of study participants = 59,848. Abbreviations: CI = Confidence interval; N = Number of study participants; OR = Odds ratio; RoB = Risk of bias; SM = Social media; and SNS = Social networking sites.

Figure AA. Forest plot for association between frequency of social media use and drug use, by social media category

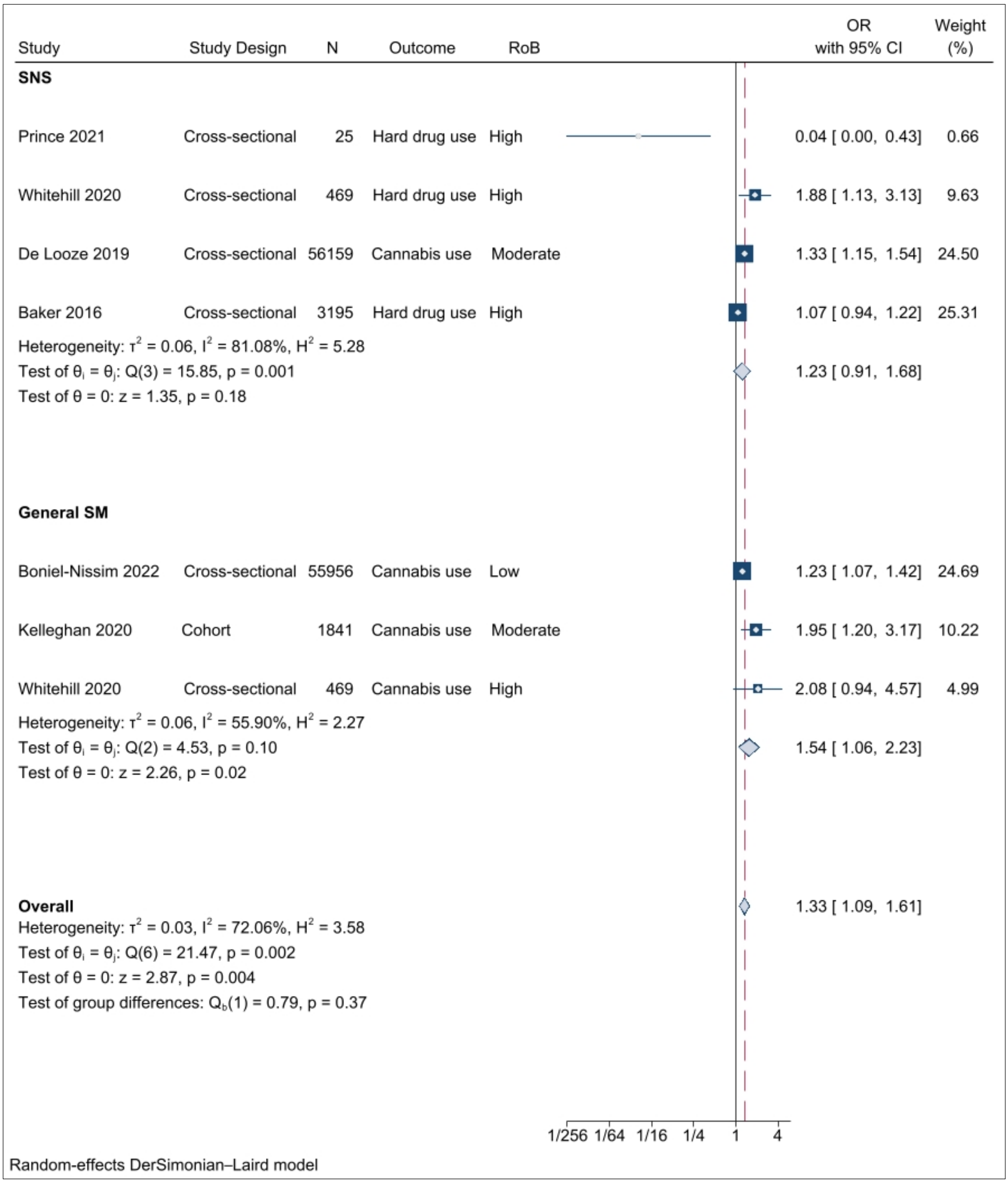

Legend: Figure presents forest plot for binary exposure (frequent/daily vs infrequent/non-daily) & binary/continuous outcome meta-analysis, with odds ratio (OR) used as common metric. Total number of study participants = 118,114. Abbreviations: CI = Confidence interval; N = Number of study participants; OR = Odds ratio; RoB = Risk of bias; SM = Social media; and SNS = Social networking sites.

Figure AB. Forest plot for association between time spent on social media and drug use

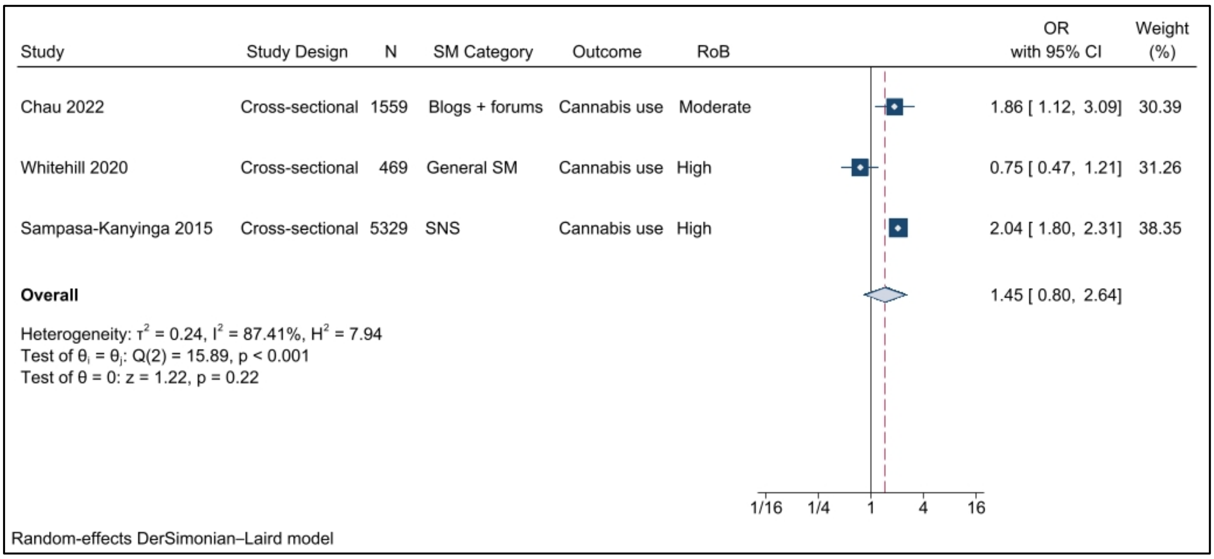

Legend: Figure presents forest plot for binary exposure (≤1hrs/day vs >1hr/day) & binary/continuous outcome meta-analysis, with odds ratio (OR) used as common metric. Total number of study participants = 7,357. Abbreviations: CI = Confidence interval; N = Number of study participants; OR = Odds ratio; RoB = Risk of bias; SM = Social media; and SNS = Social networking sites.

Figure AC. Forest plot for association between time spent on social media and drug use

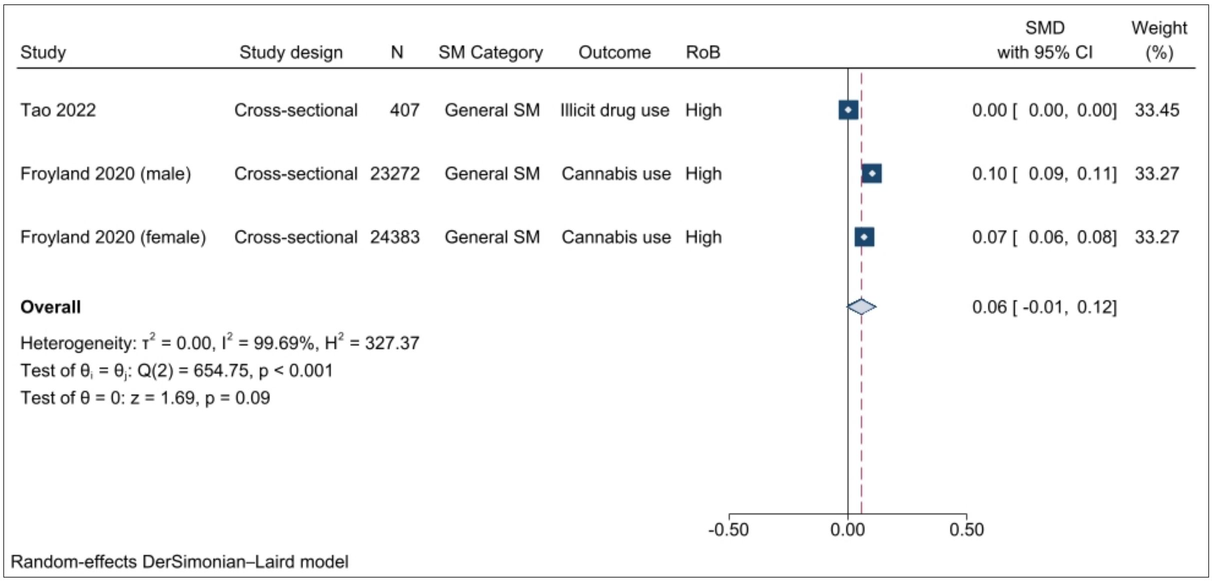

Legend: Figure presents forest plot for continuous exposure & continuous meta-analysis, with standardised mean difference (SMD) used as common metric. Total number of study participants = 48,062. Abbreviations: CI = Confidence interval; N = Number of study participants; OR = Odds ratio; RoB = Risk of bias; SM = Social media; and SMD = Standardised mean difference.

Tobacco use

Effect direction plot

Figure AD illustrates the effect direction in those studies investigating tobacco use, by exposure. One study investigated more than one exposure.[87] For time spent on social media all studies demonstrated harmful associations of social media use (95% CI 43.9 to 100%; study n=3; participant n=7216; sign test p=0.25), as did all studies investigating exposure to health-risk behaviour content (51.0 to 100%; study n = 4; participant n=24,197; sign test p=0.13). For frequency of social media use 9/10 studies (90.0%) demonstrated harmful associations (59.6 to 98.2%; participant n=431,501; sign test p=0.02). Other social media activities was investigated by one study which demonstrated a harmful association (0.00 to 79.3%; participant n=5,851; insufficient data to conduct sign test).

Figure AD. Effect direction plot for studies of the association between social media use and adolescent tobacco use, by social media exposure. Arrow size indicates sample size; arrow colour indicates study risk of bias.

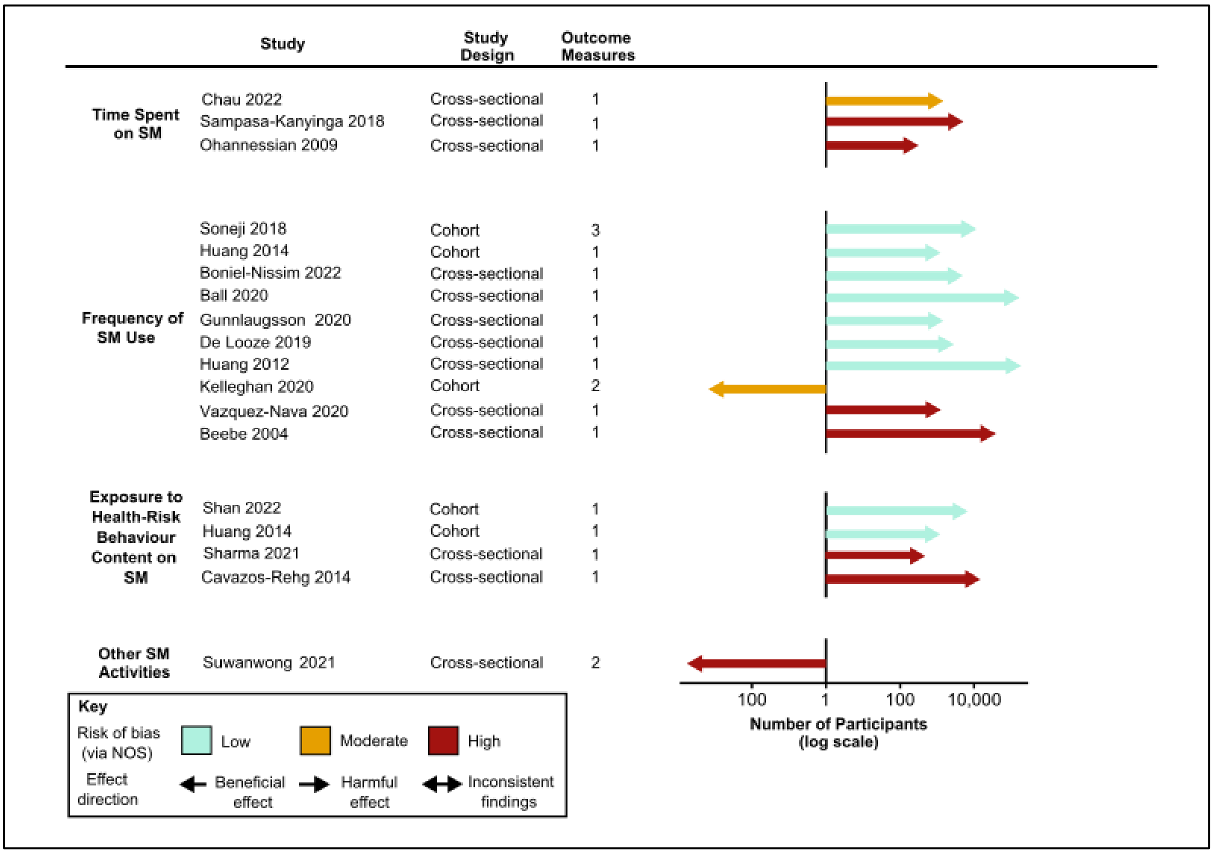

Legend: Sample size: represented by the size of the arrow, measured on a log scale. Outcome measure: number of outcome measures synthesised within each study. Studies organised by risk of bias grade, study design, and year of publication. Repeat cross-sectional studies, multiple study populations from different countries, and age subsets originating from the same study reported as separate studies. Abbreviations: NOS = Assessed via adapted Newcastle Ottawa Scale; and SM = Social media.

Forest plots for meta-analyses and subgroup analyses

Figure AE. Forest plot for association between frequency of social media use and tobacco use

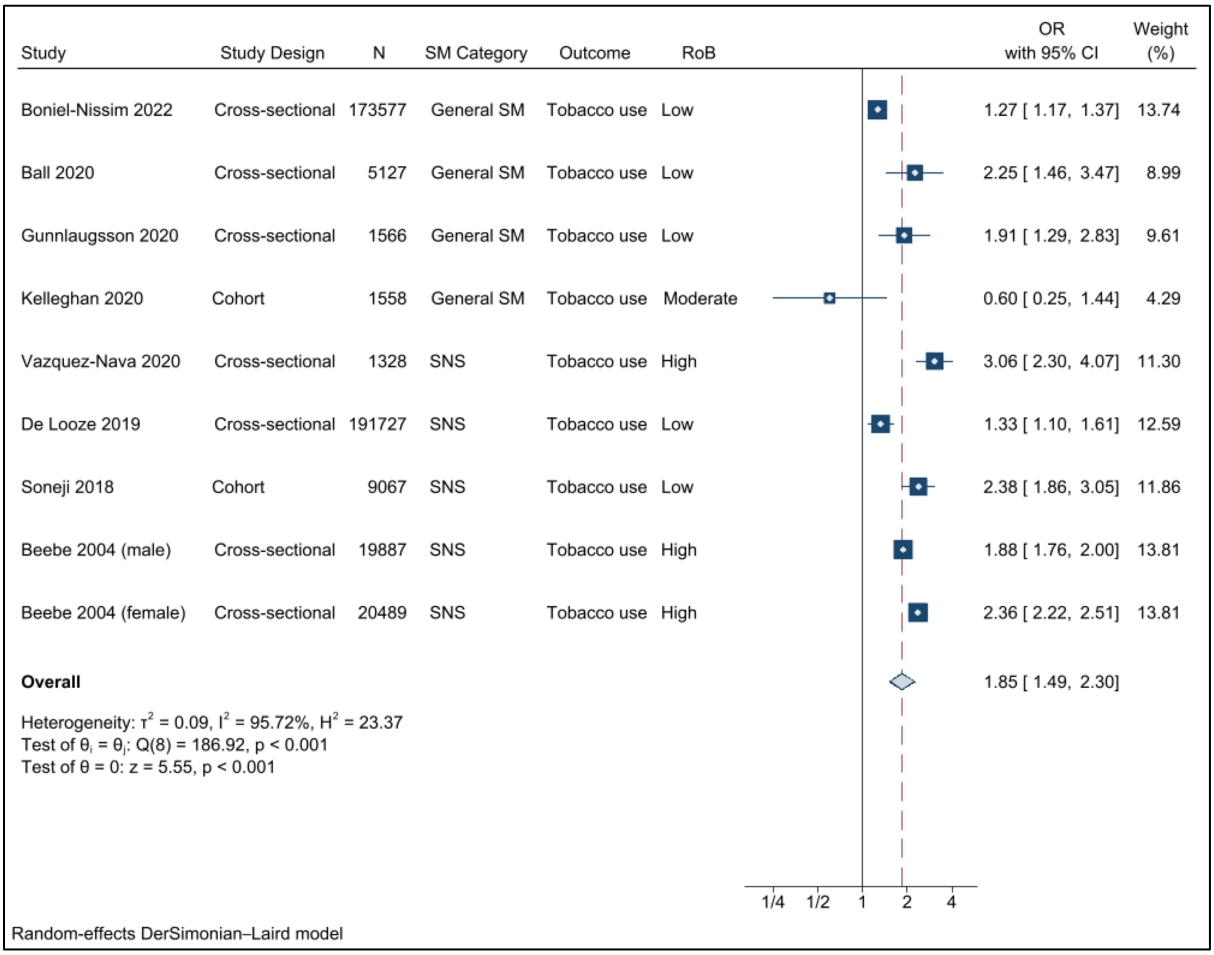

Legend: Figure presents forest plot for binary exposure (frequent vs infrequent) & binary/continuous outcome meta-analysis, with odds ratio (OR) used as common metric. Total number of study participants = 424,326. Abbreviations: CI = Confidence interval; N = Number of study participants; OR = Odds ratio; RoB = Risk of bias; SM = Social media; and SNS = Social networking sites.

Figure AF. Forest plot for association between exposure to health-risk behaviour content on social media and tobacco use

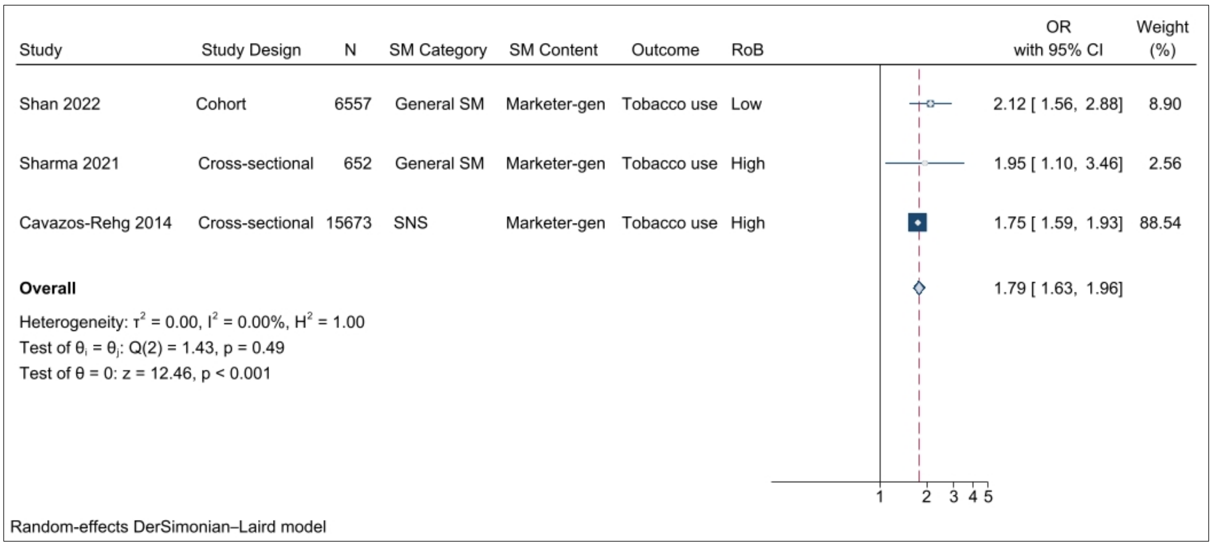

Legend: Figure presents forest plot for binary exposure (exposed vs unexposed) & binary/continuous outcome meta-analysis, with odds ratio (OR) used as common metric. Total number of study participants = 22,882. Abbreviations: CI = Confidence interval; Markter-gen = Marketer-generated content; N = Number of study participants; OR = Odds ratio; RoB = Risk of bias; SM = Social media; and SNS = Social networking sites.

Figure AG. Forest plot for association between frequency of social media use and tobacco use, by average age of study participants

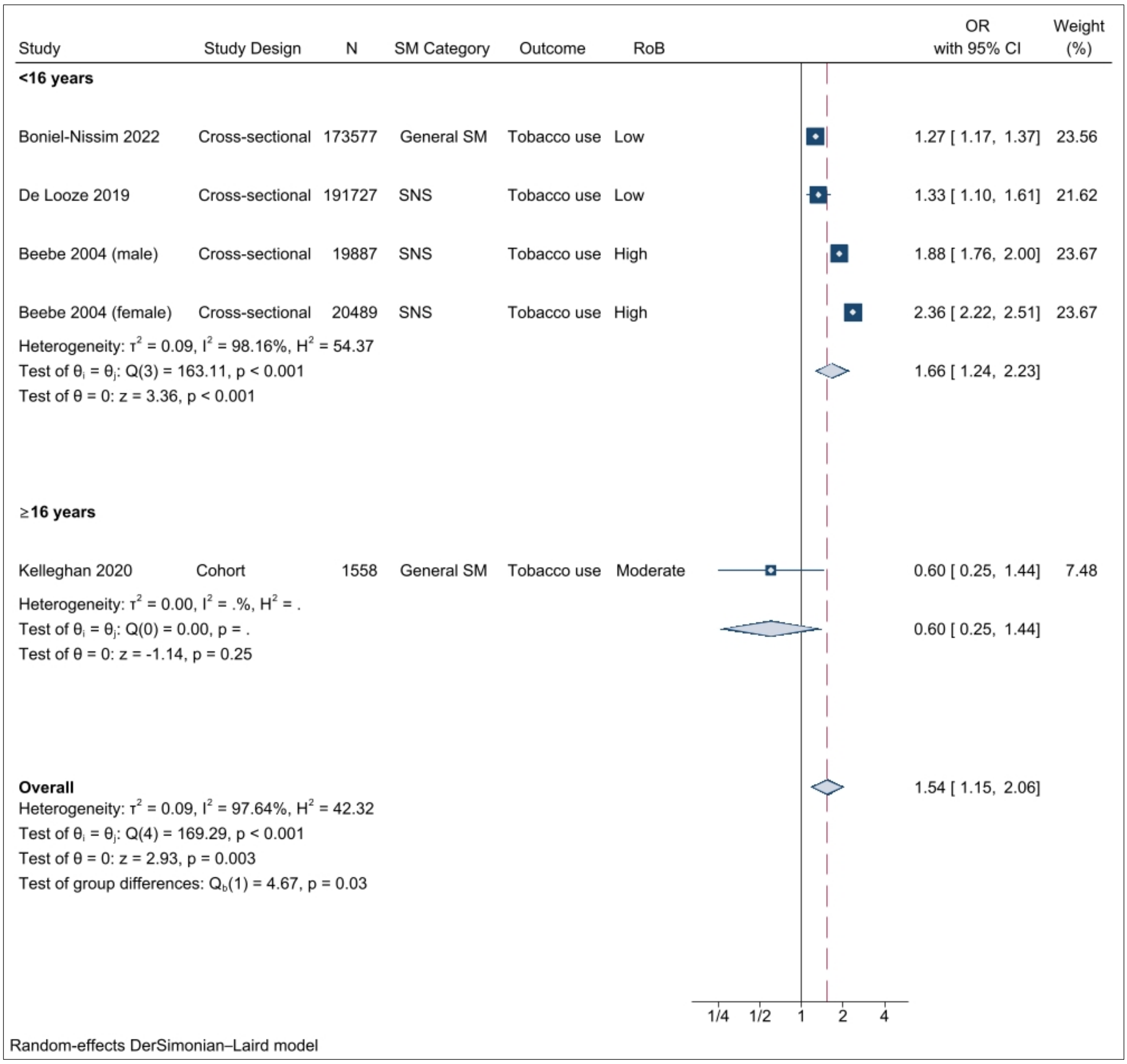

Legend: Figure presents forest plot for binary exposure (frequent vs infrequent) & binary/continuous outcome subgroup analysis, with odds ratio (OR) used as common metric. Total number of study participants = 407,238. Abbreviations: CI = Confidence interval; N = Number of study participants; OR = Odds ratio; RoB = Risk of bias; SM = Social media; and SNS = Social networking sites

Figure AH. Forest plot for association between frequency of social media use and tobacco use, by development status of study setting[a]

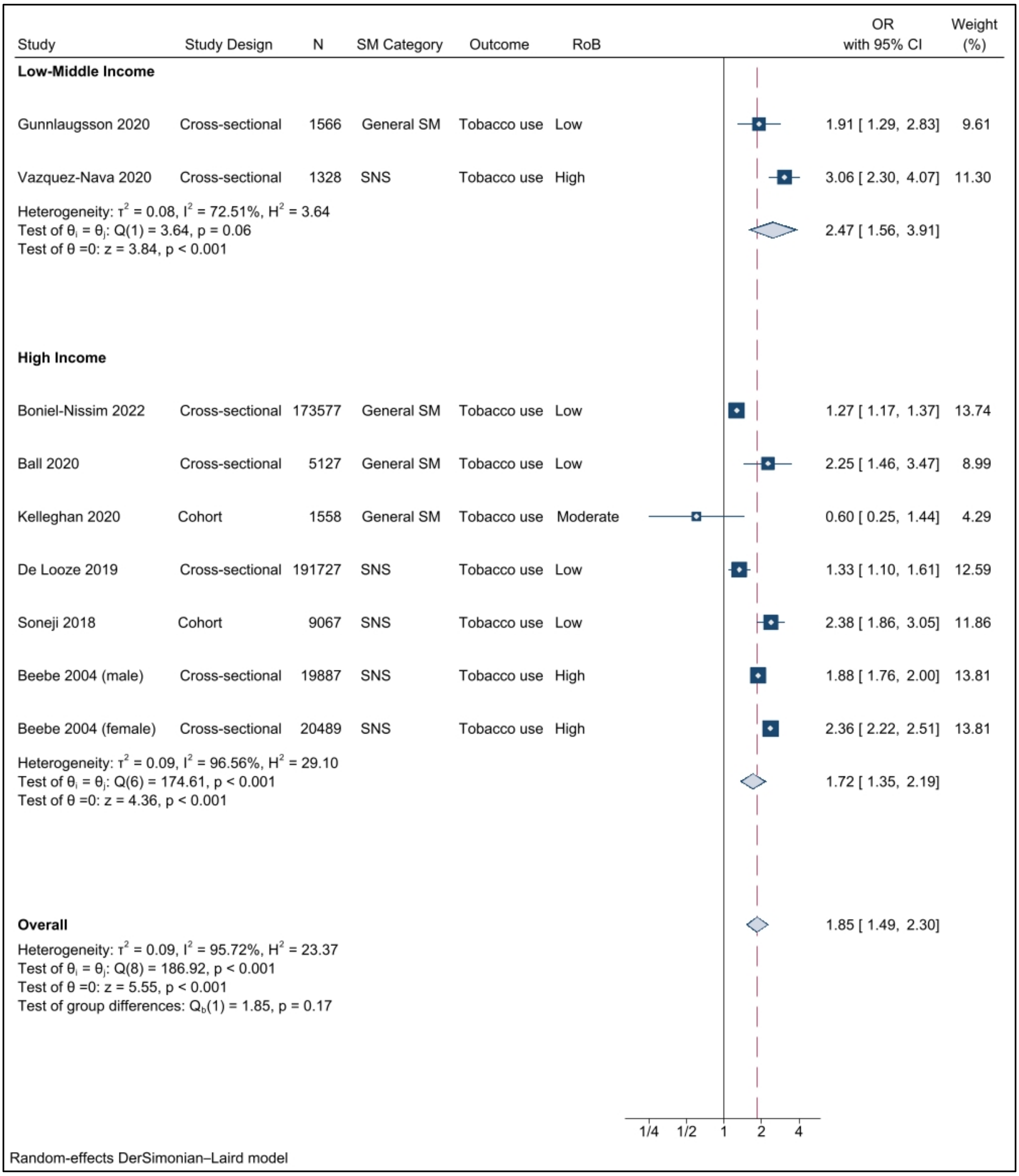

Legend: Figure presents forest plot for binary exposure (frequent vs infrequent) & binary/continuous outcome subgroup analysis, with odds ratio (OR) used as common metric. [a] Development status classified as per the World Bank Country Income Level Classification.[160] Total number of study participants = 424,326. Abbreviations: CI = Confidence interval; N = Number of study participants; OR = Odds ratio; RoB = Risk of bias; SM = Social media; and SNS = Social networking sites.

Figure AI. Forest plot for association between frequency of social media use and tobacco use, by social media category

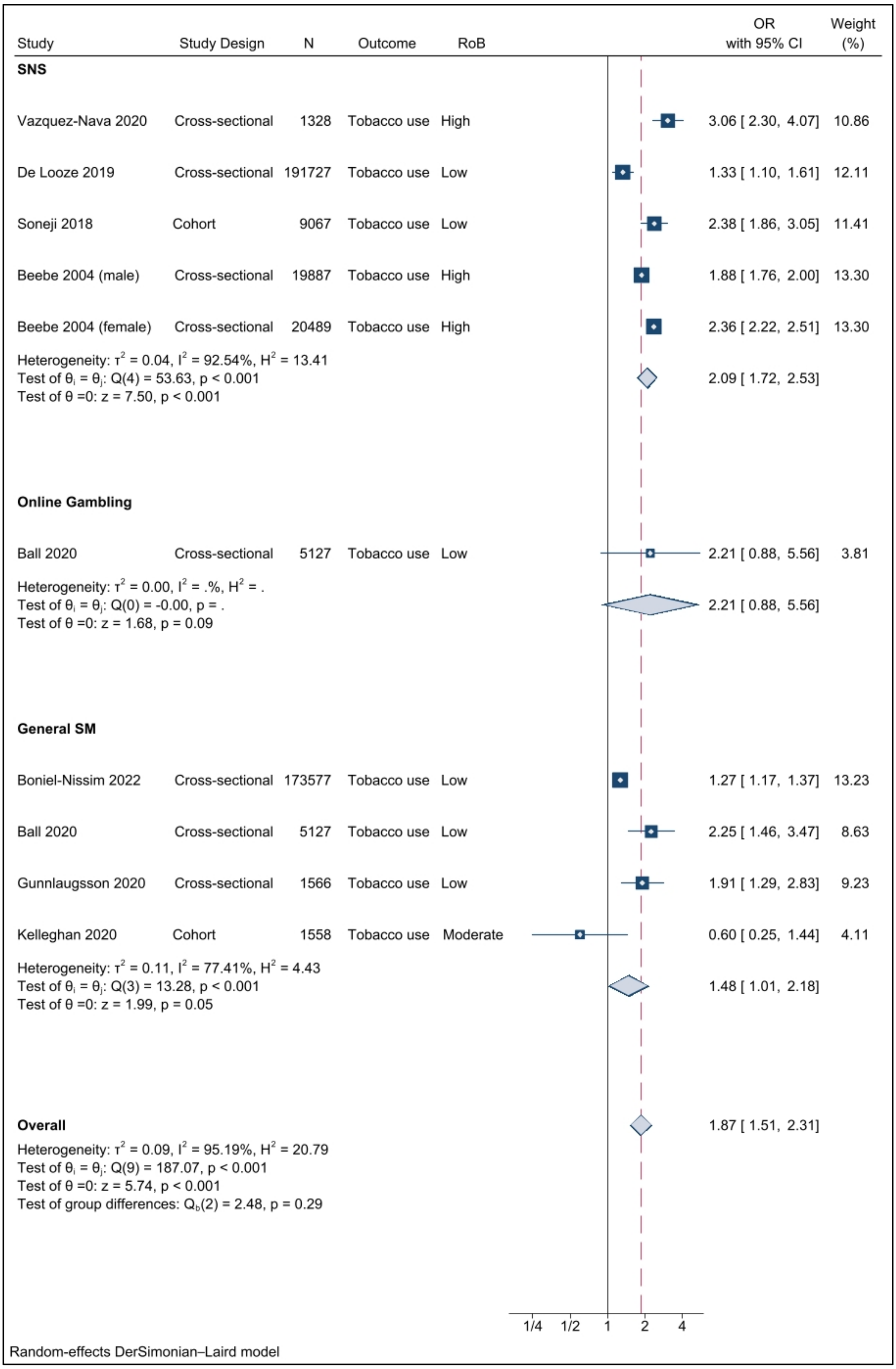

Legend: Figure presents forest plot for binary exposure (frequent vs infrequent) & binary/continuous outcome subgroup analysis, with odds ratio (OR) used as common metric. Total number of study participants = 429.453. Abbreviations: CI = Confidence interval; N = Number of study participants; OR = Odds ratio; RoB = Risk of bias; SM = Social media; and SNS = Social networking sites.

Figure AJ. Forest plot for association between exposure to health-risk behaviour content on social media and tobacco use, by development status of study setting[a]

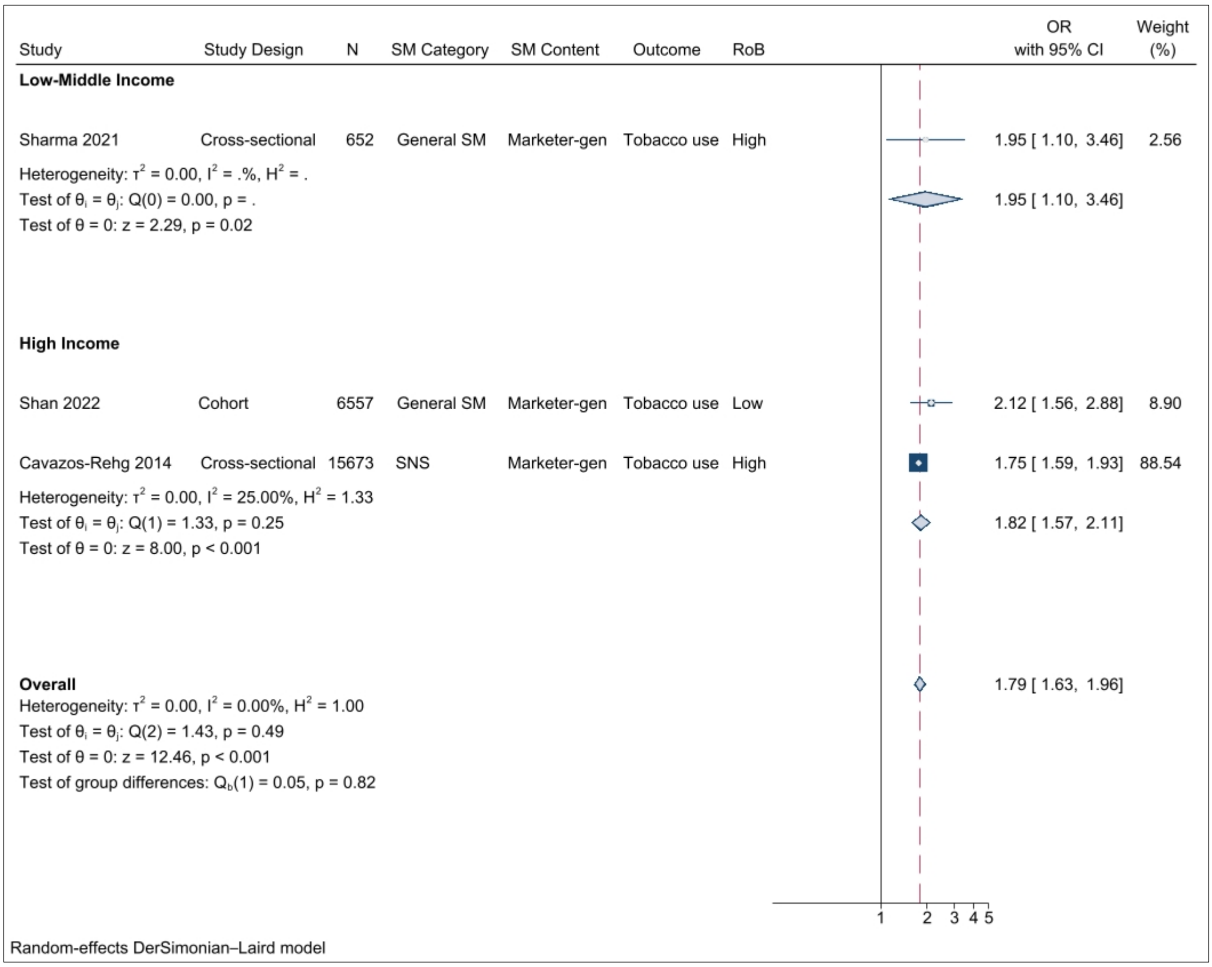

Legend: Figure presents forest plot for binary exposure (exposed vs unexposed) & binary/continuous outcome sensitivity analysis, with odds ratio (OR) used as common metric. [a] Development status classified as per the World Bank Country Income Level Classification.[160] Total number of study participants = 22,882. Abbreviations: CI = Confidence interval; Marketer-gen = Marketer-generated content; N = Number of study participants; OR = Odds ratio; RoB = Risk of bias; SM = Social media; and SNS = Social networking sites.

Figure AK. Forest plot for association between exposure to health-risk behaviour content on social media and tobacco use, by social media category

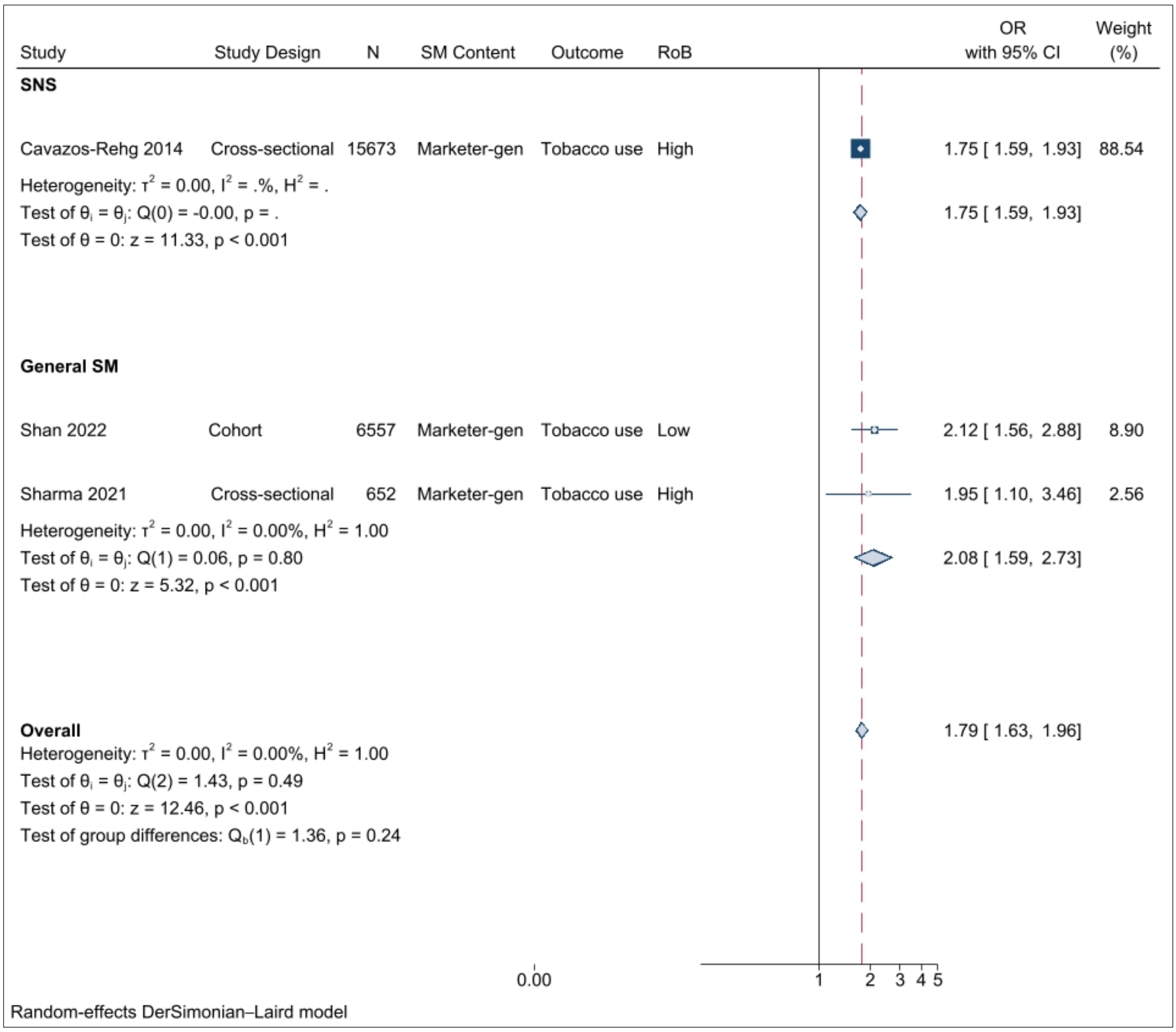

Legend: Figure presents forest plot for binary exposure (exposed vs unexposed) & binary/continuous outcome sensitivity analysis, with odds ratio (OR) used as common metric. Total number of study participants = 22,882. Abbreviations: CI = Confidence interval; Marketer-gen = Marketer-generated content; N = Number of study participants; OR = Odds ratio; RoB = Risk of bias; SM = Social media; and SNS = Social networking sites.

Use of electronic nicotine delivery systems (ENDS)

<u>Effect direction plot</u>

Figure AL demonstrates the effect direction in those studies (n=9) investigating use of electronic nicotine delivery systems (ENDS), by exposure. For time spent on social media, all studies reported harmful associations (95% CI 34.2 to 100.0%; study n=2; participant n=9,821; insufficient data to conduct sign test), for frequency of social media use, 2/3 studies (66.7%) demonstrated harmful associations (20.8 to 93.9%; participant n=18,047; sign test p=1.00) and for exposure to health-risk behaviour content on social media all studies reported harmful effects (51.0 to 100.0%; study n=4; participant n=721,322; sign test p=1.00).

Figure AL. Effect direction plot for studies of the association between social media and adolescent use of electronic nicotine delivery systems, by social media exposure. Arrow size indicates sample size; arrow colour indicates study risk of bias.

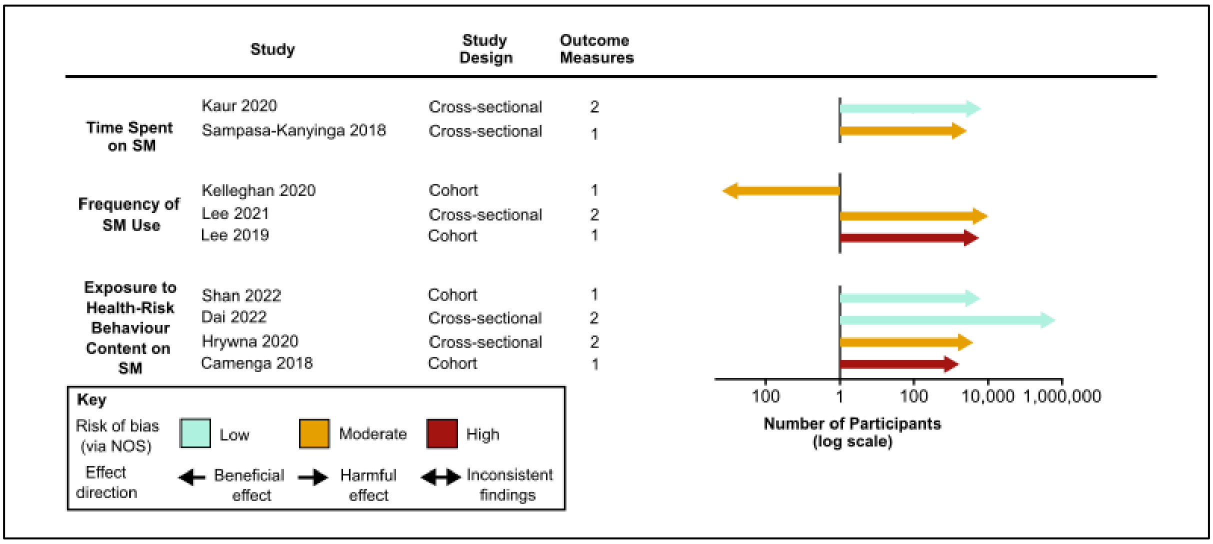

Legend: Sample size: represented by the size of the arrow, measured on a log scale. Outcome measure: number of outcome measures synthesised within each study. Studies organised by risk of bias grade, study design, and year of publication. Repeat cross-sectional studies, multiple study populations from different countries, and age subsets originating from the same study reported as separate studies. Abbreviations: NOS = Assessed via adapted Newcastle Ottawa Scale; and SM = Social media.

Forest plots for meta-analyses and subgroup analyses

Figure AM. Forest plot for association between exposure to health-risk behaviour content on social media and use of electronic nicotine delivery systems

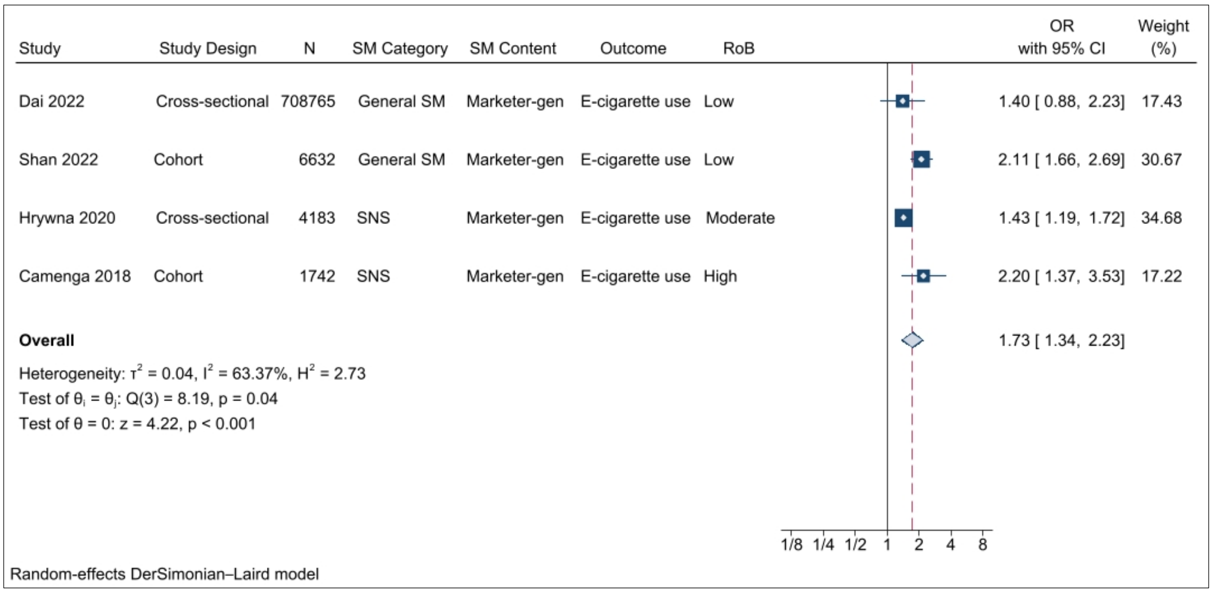

Legend: Figure presents forest plot for binary exposure (exposed vs unexposed) & binary/continuous outcome meta-analysis, with odds ratio (OR) used as common metric. Total number of study participants = 721,322. Abbreviations: CI = Confidence interval; N = Number of study participants; OR = Odds ratio; RoB = Risk of bias; SM = Social media; and SNS = Social networking sites

Figure AN. Forest plot for association between exposure to health-risk behaviour content on social media and use of electronic nicotine delivery systems, by social media category

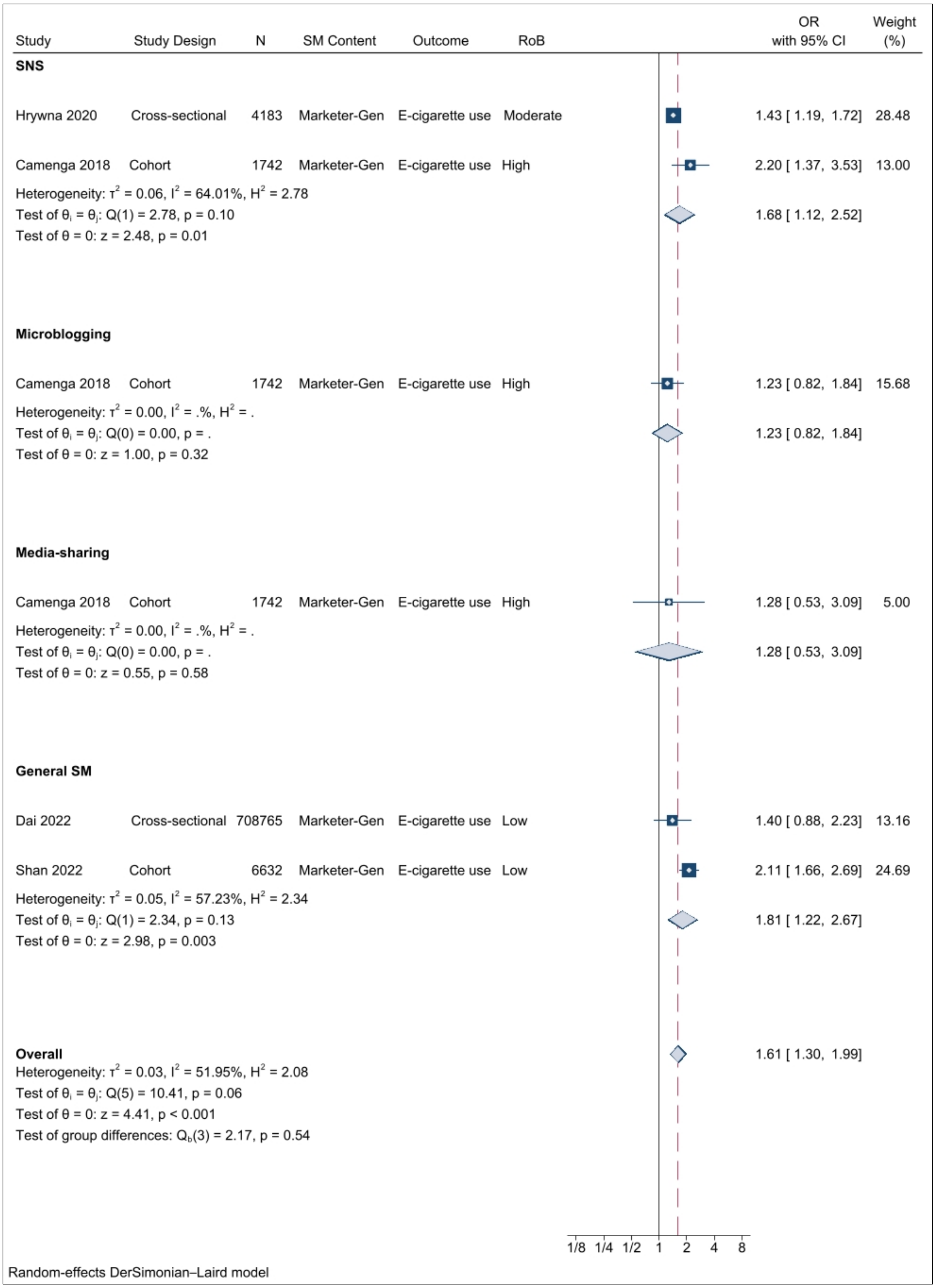

Legend: Figure presents forest plot for binary exposure (exposed vs unexposed) & binary/continuous outcome subgroup analysis, with odds ratio (OR) used as common metric. Total number of study participants = 724,716. Abbreviations: CI = Confidence interval; N = Number of study participants; OR = Odds ratio; RoB = Risk of bias; SM = Social media; and SNS = Social networking sites

Figure AO. Forest plot for association between exposure to health-risk behaviour content on social media and use of electronic nicotine delivery systems, by social media platform

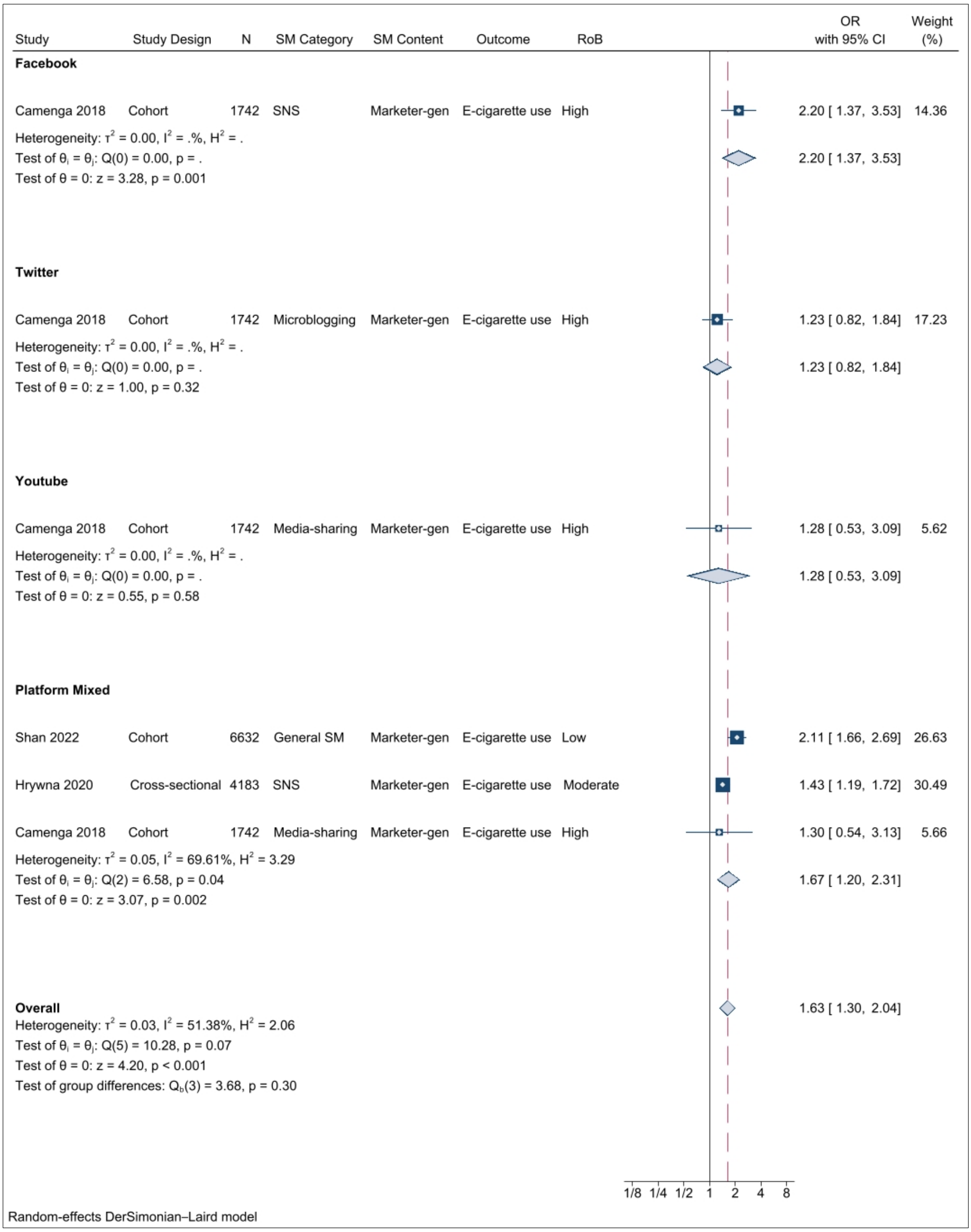

Legend: Figure presents forest plot for binary exposure (exposed vs unexposed) & binary/continuous outcome subgroup analysis, with odds ratio (OR) used as common metric. Total number of study participants = 17,783. Abbreviations: CI = Confidence interval; N = Number of study participants; OR = Odds ratio; RoB = Risk of bias; SM = Social media; and SNS = Social networking sites

Sexual risk behaviour

Effect direction plot

Figure AP demonstrates the effect direction in those studies investigating sexual risk behaviour, by exposure. Six studies investigated more than one exposure type. [60,90,98,115,150,113] After excluding one study with inconsistent findings (participant n=333),[125] for time spent on social media, 5/6 studies (83.3%) reported harmful associations (95% CI 43.6 to 97.0%; participant n=13,528; sign test p=0.22), 17/18 studies (94.5%) reported harmful associations for frequency of social media use (74.2 to 99.0%; participant n=53,433; sign test p < 0.001), all studies reported harmful associations for exposure to health-risk behaviour content on social media (34.2 to 100.0%; study n=2; participant n=138; insufficient data to conduct sign test), and 4/5 studies (80.0%) reported harmful associations of engagement in other social media activities (37.6 to 96.4%; participant n=6,141; sign test p=0.38).

Figure AP. Effect direction plot for studies of the association between social media use and adolescent engagement in sexual risk behaviour, by social media exposure. Arrow size indicates sample size; arrow colour indicates study risk of bias.

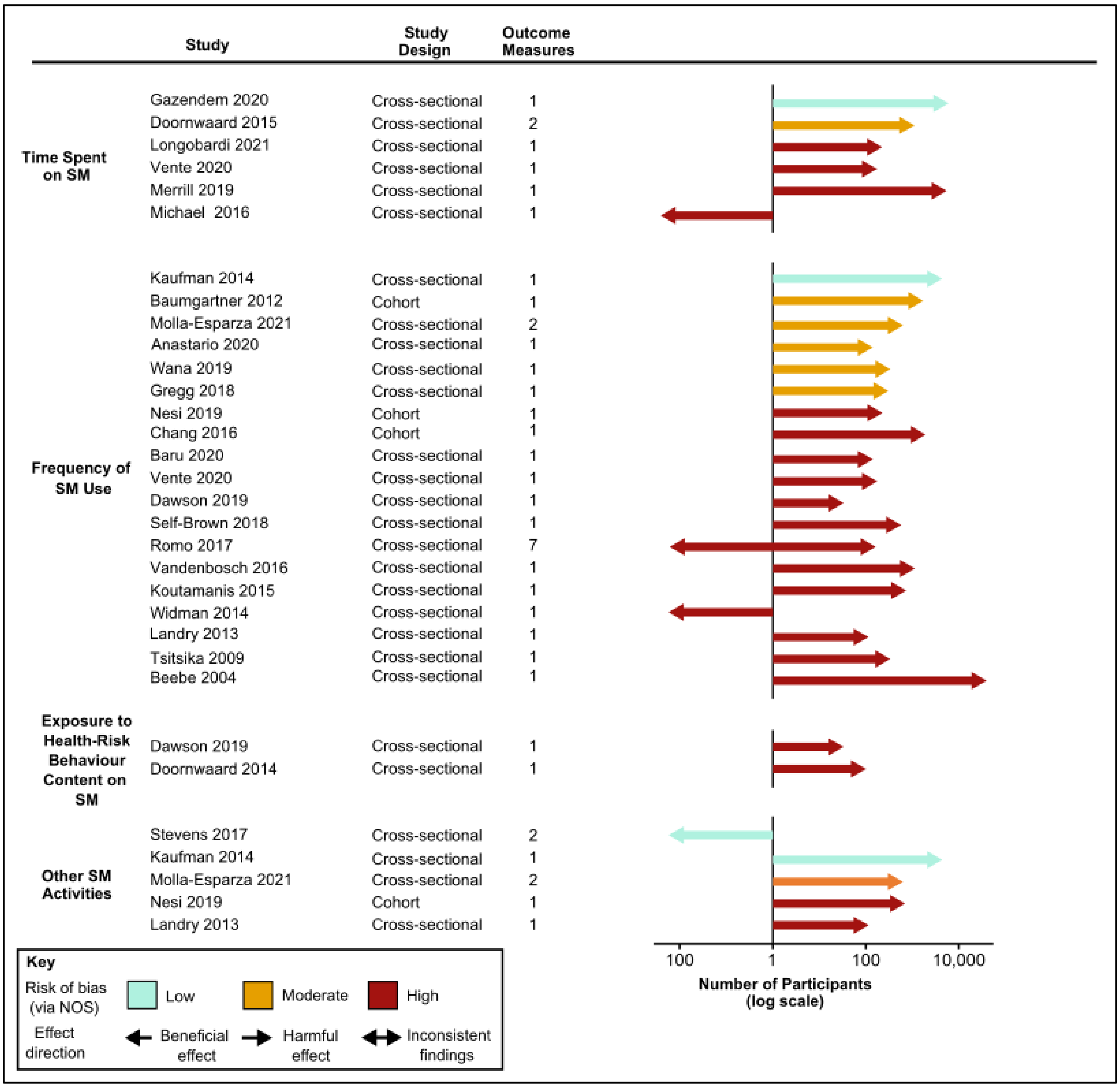

Legend: Sample size: represented by the size of the arrow, measured on a log scale. Outcome measure: number of outcome measures synthesised within each study. Studies organised by risk of bias grade, study design, and year of publication. Repeat cross-sectional studies, multiple study populations from different countries, and age subsets originating from the same study reported as separate studies. Abbreviations: NOS = Assessed via adapted Newcastle Ottawa Scale; and SM = Social media.

Forest plots for meta-analyses and subgroup analyses

Figure AQ. Forest plot for association between frequency of social media use and sexual risk behaviour

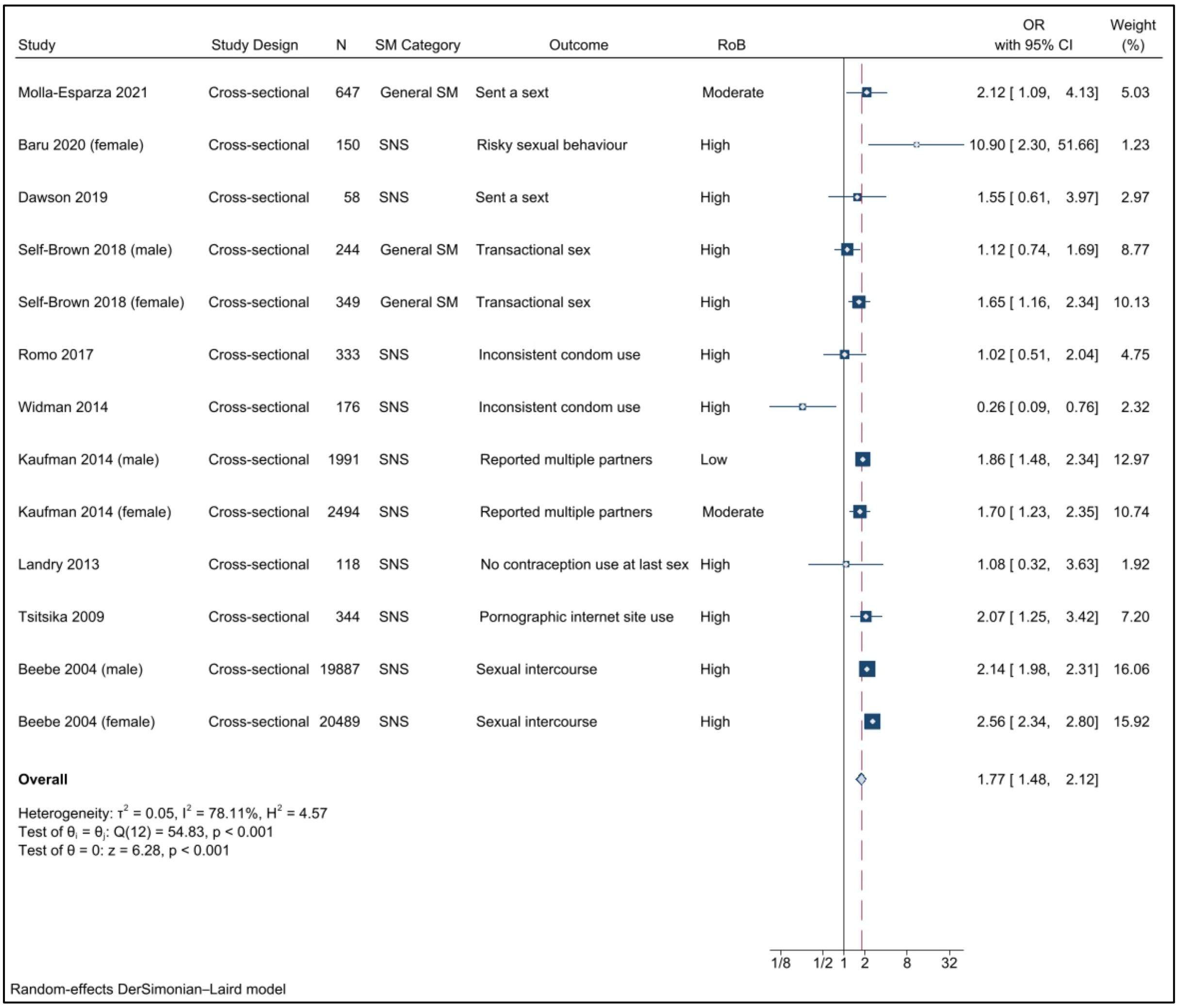

Legend: Figure presents forest plot for binary exposure (frequent/at all vs infrequent/not at all) & binary/continuous outcome meta-analysis, with odds ratio (OR) used as common metric. Total number of study participants = 47,280. Abbreviations: CI = Confidence interval; N = Number of study participants; OR = Odds ratio; RoB = Risk of bias; SM = Social media; and SNS = Social networking sites.

Figure AR. Forest plot for association between frequency of social media use and sexual risk behaviour, average age of study participants

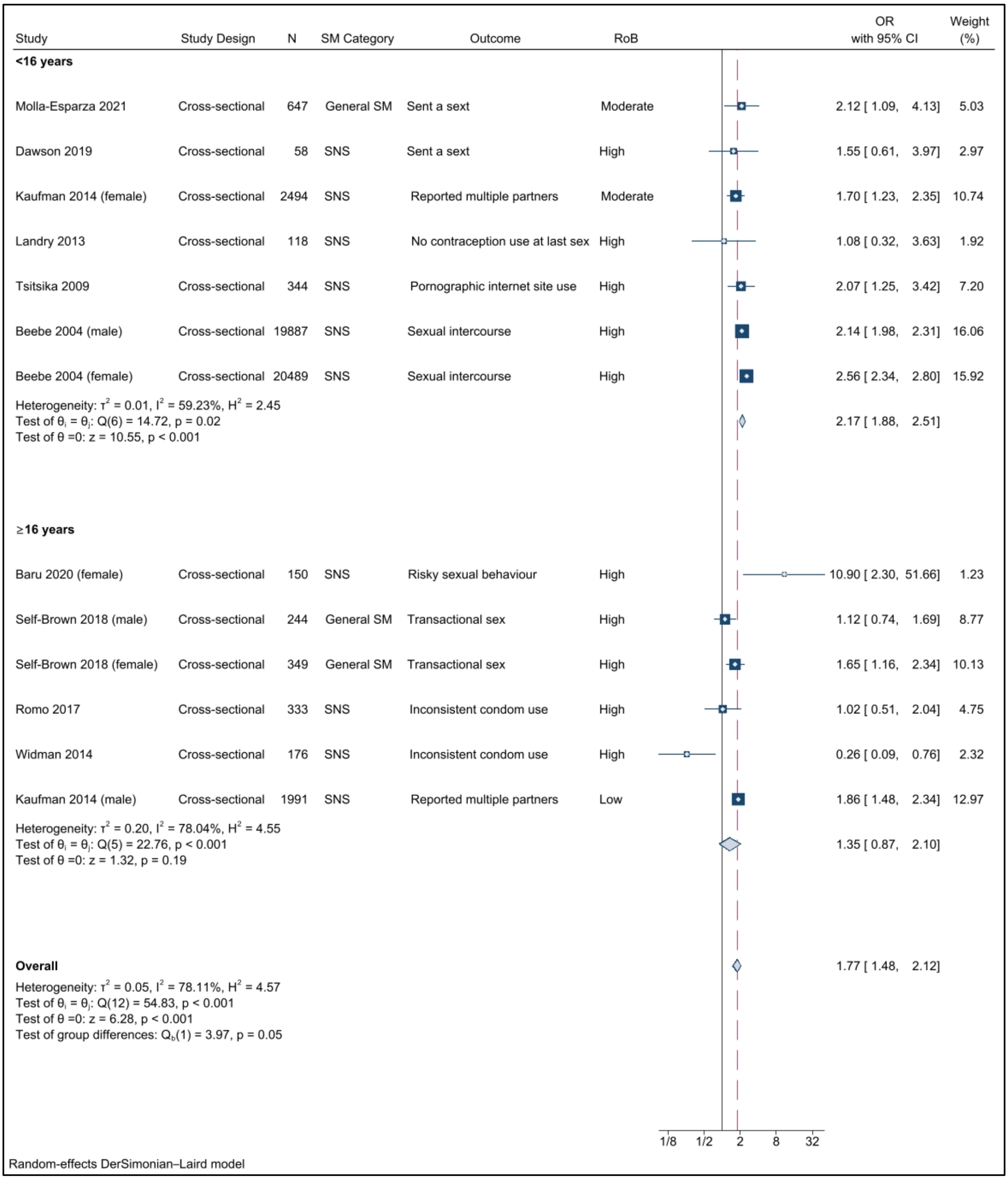

Legend: Figure presents forest plot for binary exposure (frequent/at all vs infrequent/not at all) & binary/continuous outcome subgroup analysis, with odds ratio (OR) used as common metric. Total number of study participants = 47,280. Abbreviations: CI = Confidence interval; N = Number of study participants; OR = Odds ratio; RoB = Risk of bias; SM = Social media; and SNS = Social networking sites.

Figure AS. Forest plot for association between frequency of social media use and sexual risk behaviour, by social media category

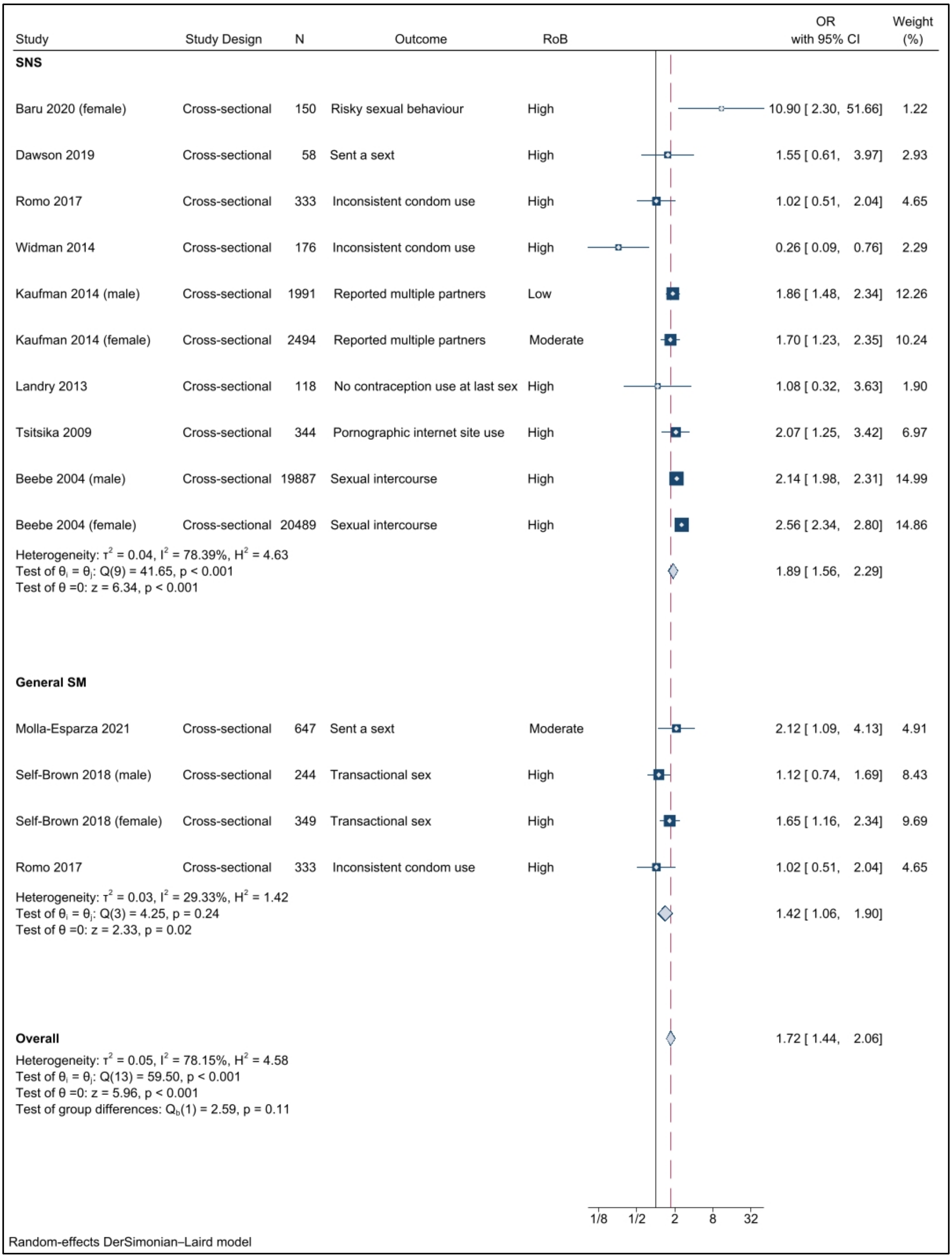

Legend: Figure presents forest plot for binary exposure (frequent/at all vs infrequent/not at all) & binary/continuous outcome subgroup analysis, with odds ratio (OR) used as common metric. Total number of study participants = 47,613. Abbreviations: CI = Confidence interval; N = Number of study participants; OR = Odds ratio; RoB = Risk of bias; SM = Social media; and SNS = Social networking sites.

Figure AT. Forest plot for association between frequency of social media use and sexual risk behaviour, by development status of study setting[a]

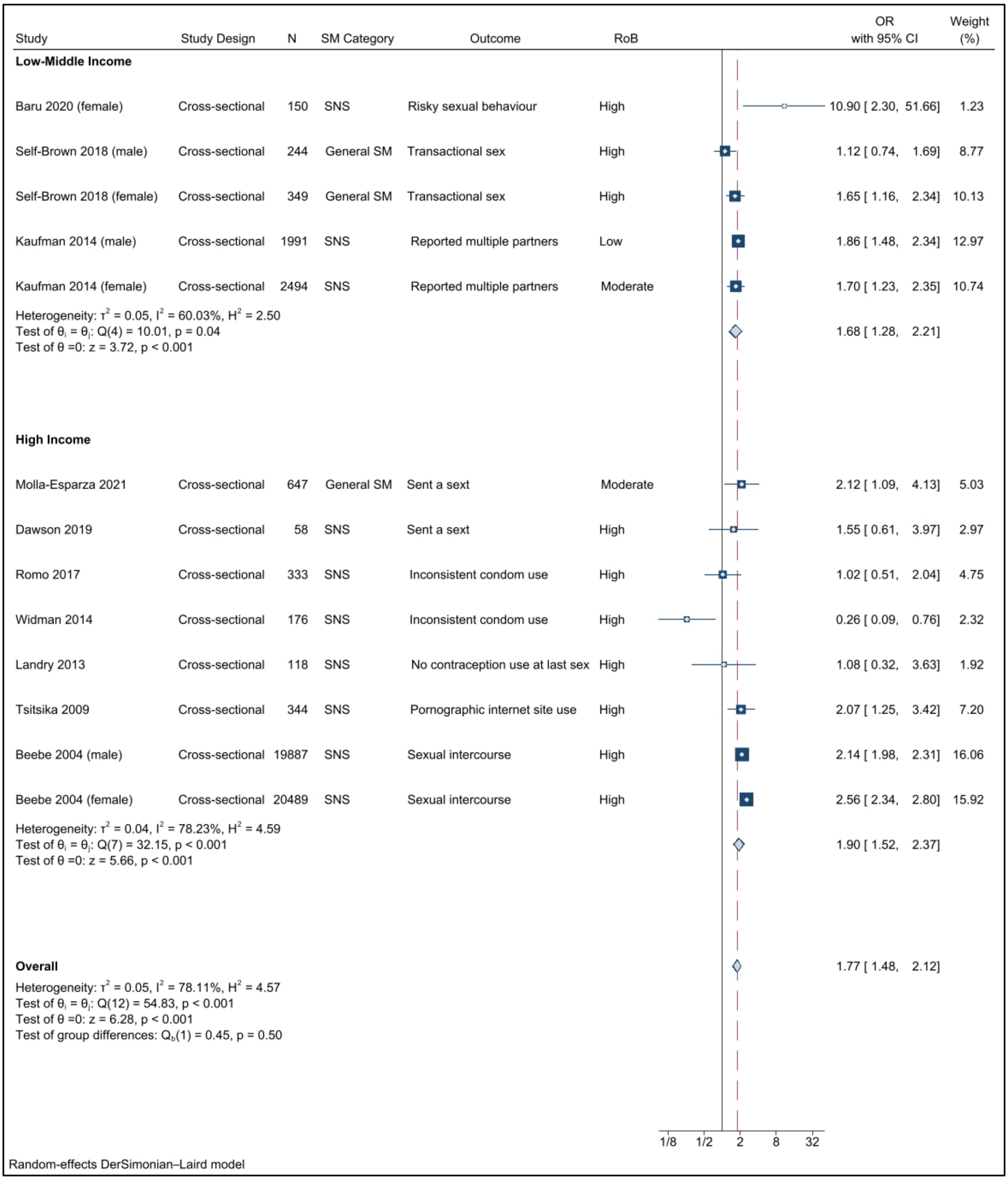

Legend: Figure presents forest plot for binary exposure (frequent/at all vs infrequent/not at all) & binary/continuous outcome subgroup analysis, with odds ratio (OR) used as common metric. [a] Development status classified as per the World Bank Country Income Level Classification.[160] Total number of study participants = 47,280. Abbreviations: CI = Confidence interval; N = Number of study participants; OR = Odds ratio; RoB = Risk of bias; SM = Social media; and SNS = Social networking sites.

Figure AU. Forest plot for association between frequency of social media use and sexual risk behaviour, by sex

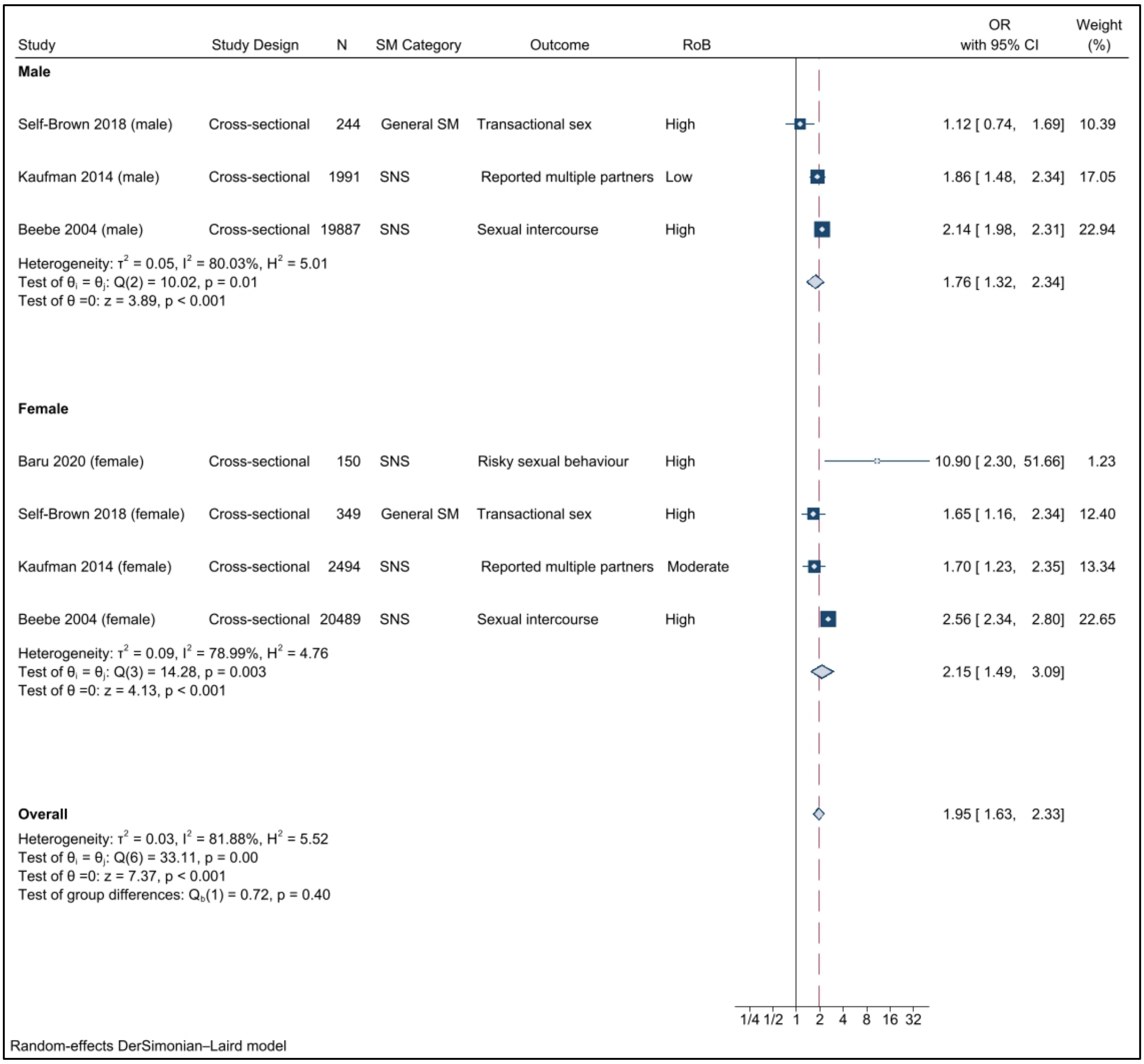

Legend: Figure presents forest plot for binary exposure (frequent/at all vs infrequent/not at all) & binary/continuous outcome subgroup analysis, with odds ratio (OR) used as common metric. Total number of study participants = 45,604. Abbreviations: CI = Confidence interval; N = Number of study participants; OR = Odds ratio; RoB = Risk of bias; SM = Social media; and SNS = Social networking sites.

Figure AV. Forest plot for association between frequency of social media use sexual risk behaviour, by average socioeconomic position of study participants

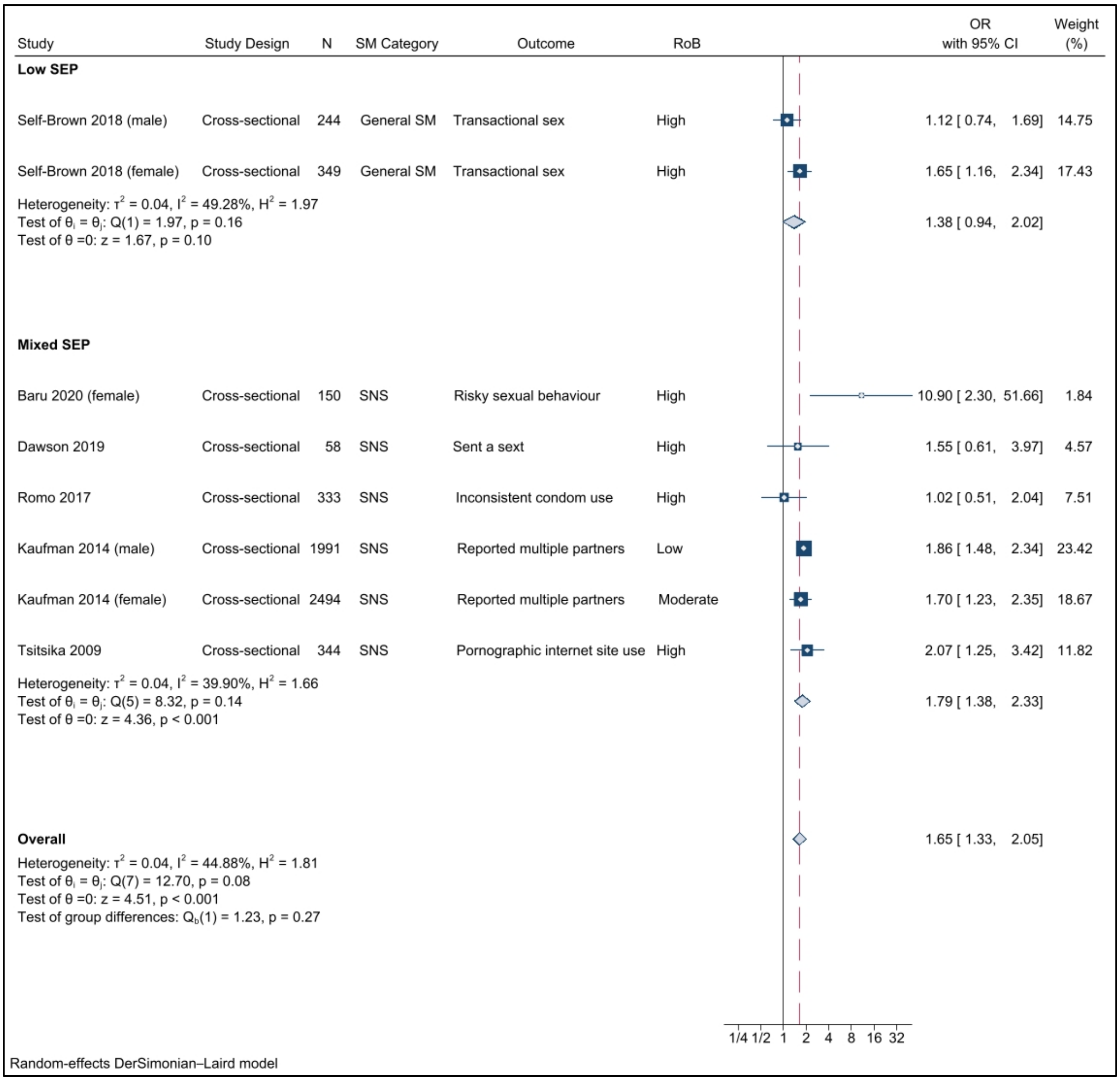

Legend: Figure presents forest plot for binary exposure (frequent/at all vs infrequent/not at all) & binary/continuous outcome subgroup analysis, with odds ratio (OR) used as common metric. Total number of study participants = 5,963. Abbreviations: CI = Confidence interval; N = Number of study participants; OR = Odds ratio; RoB = Risk of bias; SM = Social media; and SNS = Social networking sites.

Figure AW. Forest plot for association between frequency of social media use and sexual risk behaviour

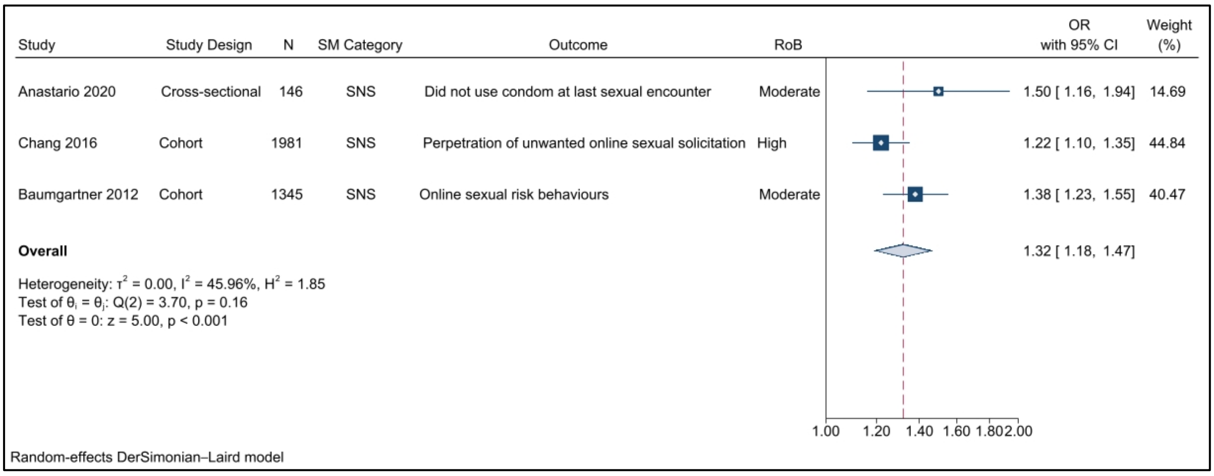

Legend: Figure presents forest plot for continuous exposure & binary outcome meta-analysis, with odds ratio (OR) used as common metric. Total number of study participants = 3,472. Abbreviations: CI = Confidence interval; N = Number of study participants; OR = Odds ratio; RoB = Risk of bias; SM = Social media; and SNS = Social networking sites.

Figure AX. Forest plot for association between frequency of social media use and sexual risk behaviour, by social media category

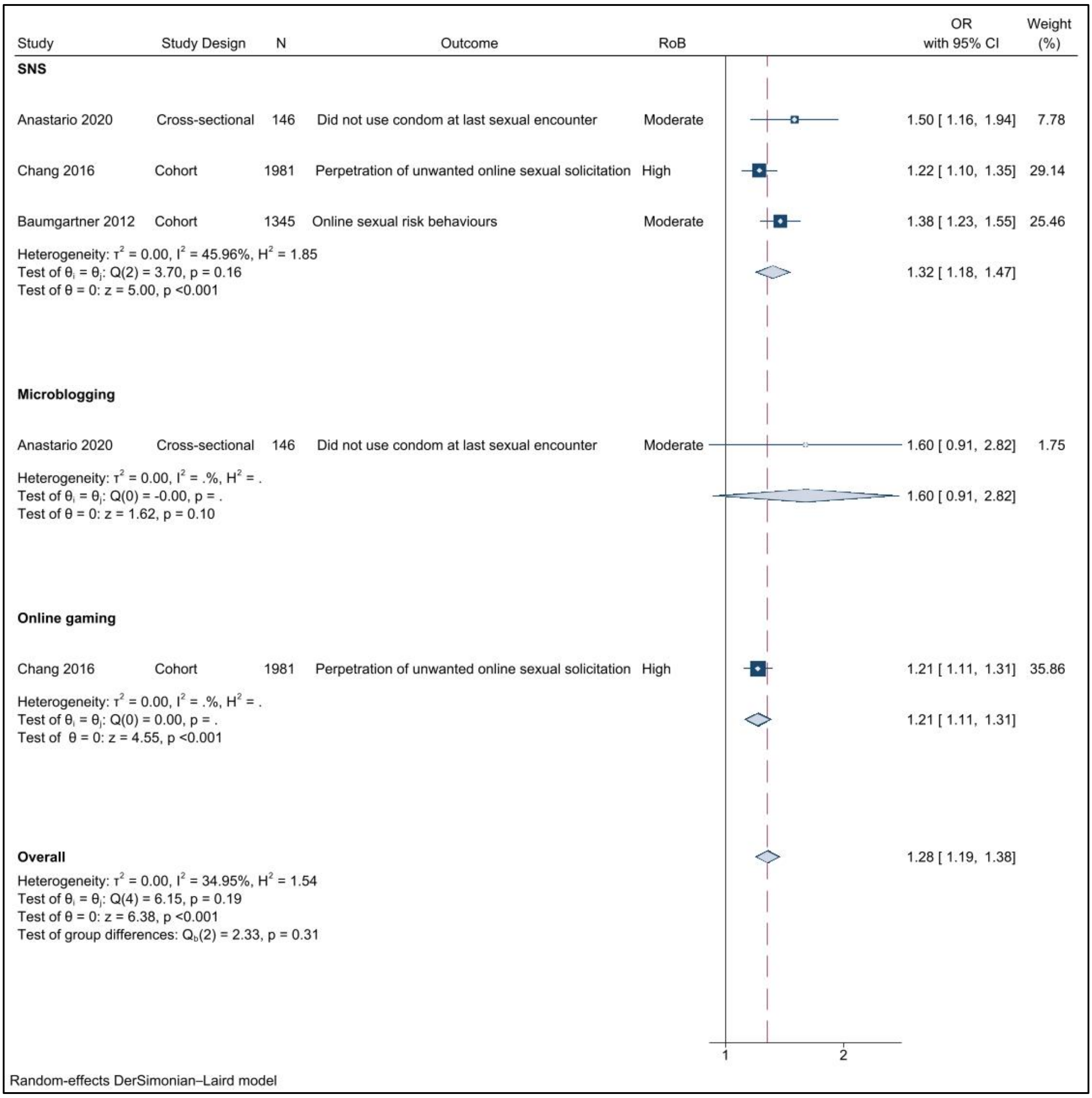

Legend: Figure presents forest plot for continuous exposure & binary outcome subgroup analysis, with odds ratio (OR) used as common metric. Total number of study participants = 5,599. Abbreviations: CI = Confidence interval; N = Number of study participants; OR = Odds ratio; SM = Social media; and SNS = Social networking sites.

Figure AY. Forest plot for association between time spent on social media and sexual risk behaviour

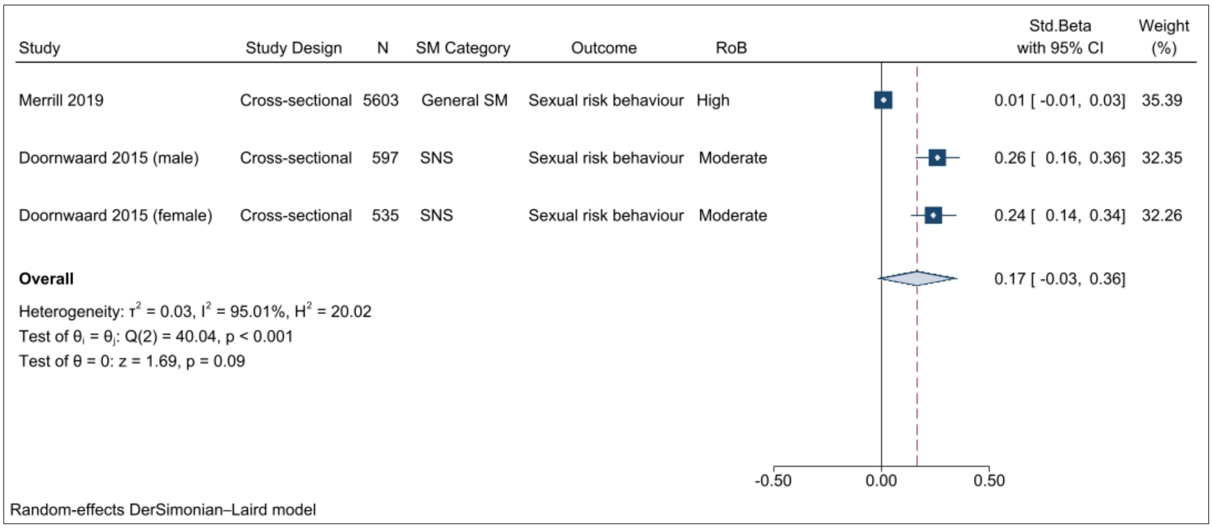

Legend: Figure presents forest plot for continuous exposure & continuous outcome meta-analysis, with standardised beta (Std.Beta) used as common metric. Total number of study participants = 6,735. Abbreviations: CI = Confidence interval; N = Number of study participants; RoB = Risk of bias; SM = Social media; SNS = Social networking sites; and Std. Beta = Standardised beta.

Figure AZ. Forest plot for association between time spent on social media and sexual risk behaviour

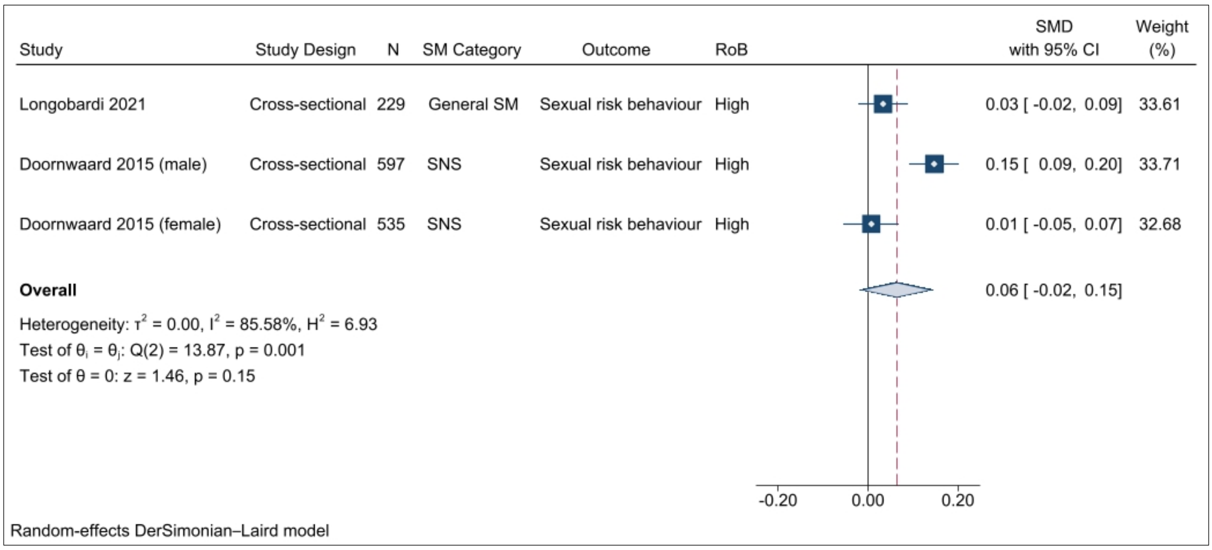

Legend: Figure presents forest plot for continuous exposure & continuous outcome meta-analysis, with standardised mean difference (SMD) used as common metric. Total number of study participants = 1,361. Abbreviations: CI = Confidence interval; N = Number of study participants; RoB = Risk of bias; SM = Social media; SMD = Standardised mean difference; and SNS = Social networking sites.

Gambling

Effect direction plot

Figure BA shows the effect direction in those studies investigating gambling behaviour, by exposure. After excluding one study demonstrating inconsistent effects (participant n=14,478),[46] for frequency of social media use all studies reported harmful associations (95% CI 56.6 to 100.0%; study n=5; participant n=7,928; sign test p=0.06). Other social media activities was investigated by one study which demonstrated a harmful association on gambling behaviours (20.7 to 100.0%; participant n=3,772; insufficient data to conduct sign test).

Figure BA. Effect direction plot for studies of the association between social media use and adolescent gambling, by social media exposure. Arrow size indicates sample size; arrow colour indicates study risk of bias

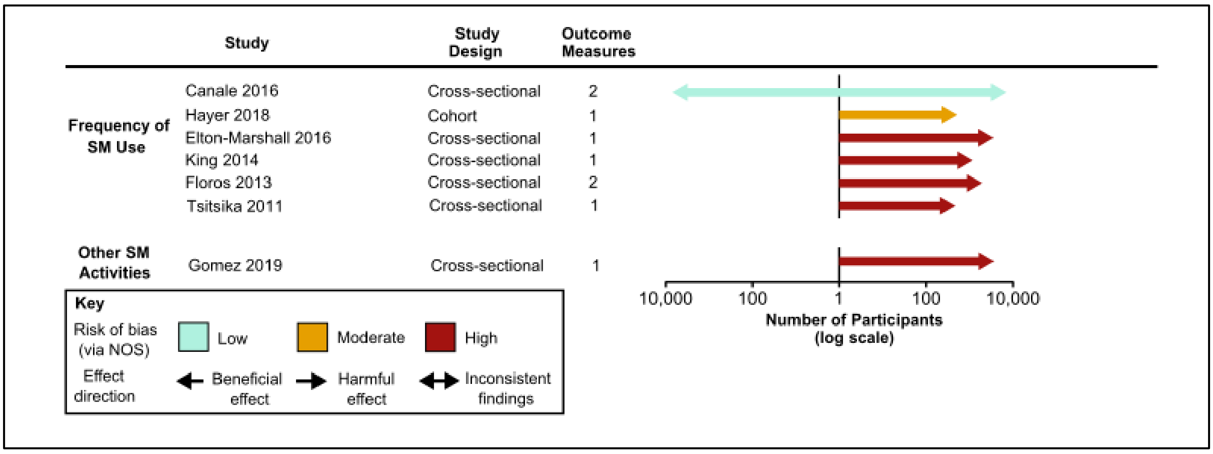

Legend: Sample size: represented by the size of the arrow, measured on a log scale. Outcome measure: number of outcome measures synthesised within each study. Studies organised by risk of bias grade, study design, and year of publication. Repeat cross-sectional studies, multiple study populations from different countries, and age subsets originating from the same study reported as separate studies. Abbreviations: NOS = Assessed via adapted Newcastle Ottawa Scale; and SM = Social media.

Forest plots for meta-analyses and subgroup analyses

Figure BB. Forest plot for association between frequency of social media use and gambling (not via social media)

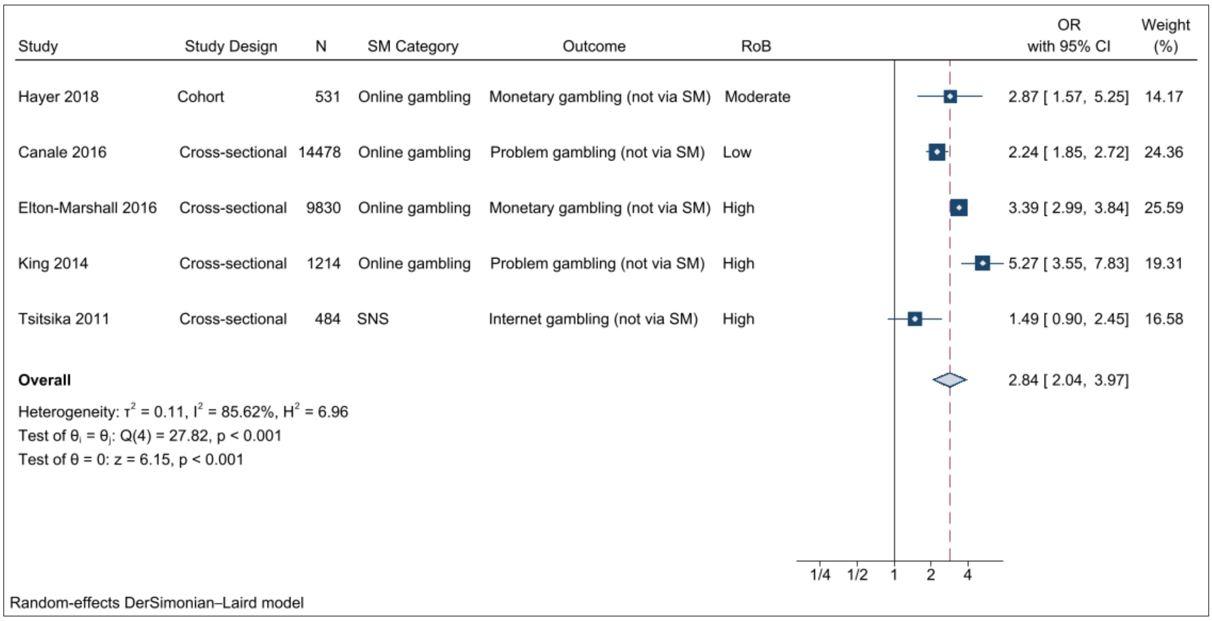

Legend: Figure presents forest plot for binary exposure (frequent/at all vs infrequent/not at all) & binary/continuous outcome meta-analysis, with odds ratio (OR) used as common metric. Total number of study participants = 26,537. Abbreviations: CI = Confidence interval; N = Number of study participants; OR = Odds ratio; RoB = Risk of bias; SM = Social media; and SNS = Social networking sites

Figure BC Forest plot for association between frequency of social media use and gambling (not via social media), by average age of study participants

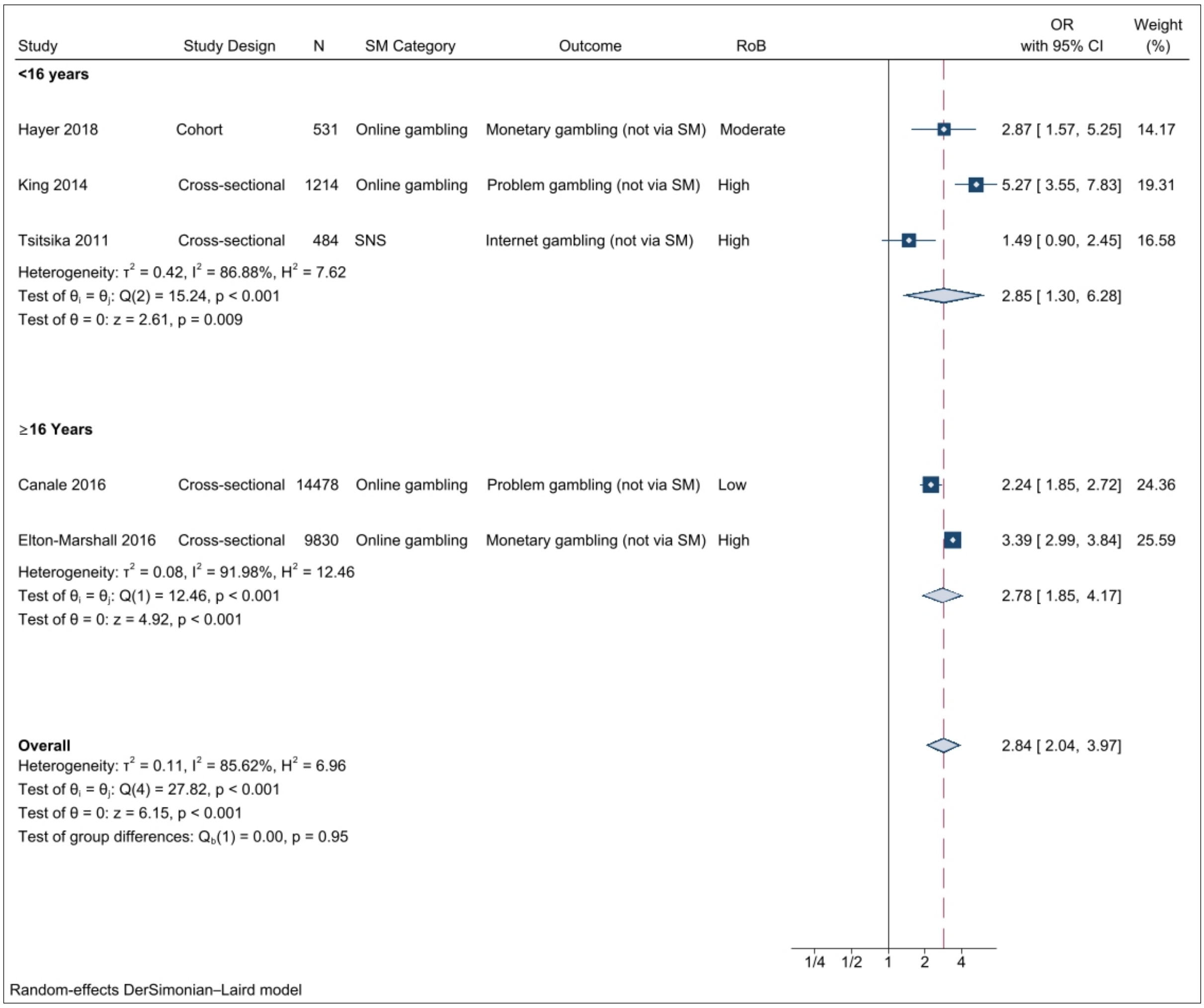

Legend: Figure presents forest plot for binary exposure (frequent/at all vs infrequent/not at all) & binary/continuous outcome subgroup analysis, with odds ratio (OR) used as common metric. Total number of study participants = 26,537. Abbreviations: CI = Confidence interval; N = Number of study participants; OR = Odds ratio; RoB = Risk of bias; SM = Social media; and SNS = Social networking sites.

Figure BD. Forest plot for association between frequency of social media use and gambling (not via social media), by social media category

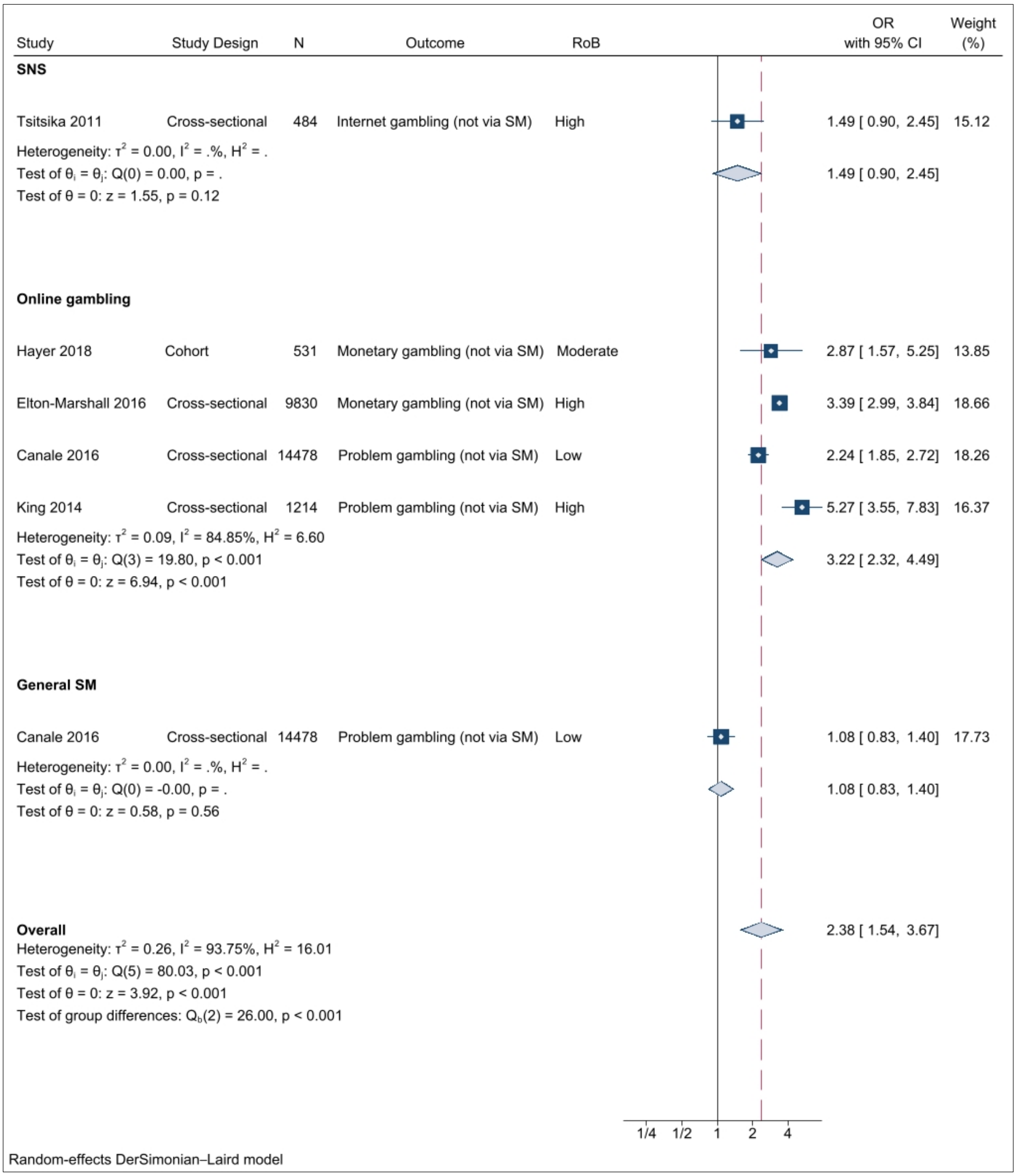

Legend: Figure presents forest plot for binary exposure (frequent/at all vs infrequent/not at all) & binary/continuous outcome subgroup analysis, with odds ratio (OR) used as common metric. Total number of study participants = 41,015. Abbreviations: CI = Confidence interval; N = Number of study participants; OR = Odds ratio; RoB = Risk of bias; SM = Social media; and SNS = Social networking sites.

Anti-social behaviour

Effect direction plot

Figure BE demonstrates the effect direction in those studies investigating anti-social risk behaviour, by exposure. One study investigated more than one exposure type.[52] Across all investigated exposures, all studies demonstrated harmful associations of social media use (time spent on social media: 95% CI 61.0 to 100.0%, study n=6, participant n=51,611, sign test p=0.03; frequency of social media use: 64.6 to 100.0%, study n=7, participant n=56,918, sign test p=0.02; and exposure to health-risk behaviour content on social media: 34.2 to 100.0%, study n=2, participant n=1,372, insufficient data to conduct sign test). Other social media activities was investigated by one study, which demonstrated a harmful effect (20.7 to 100.0%; participant n=1,167; insufficient data to conduct sign test.

Figure BE. Effect direction plot for studies of the association between social media use and adolescent engagement in anti-social behaviour, by social media exposure. Arrow size indicates sample size; arrow colour indicates study risk of bias.

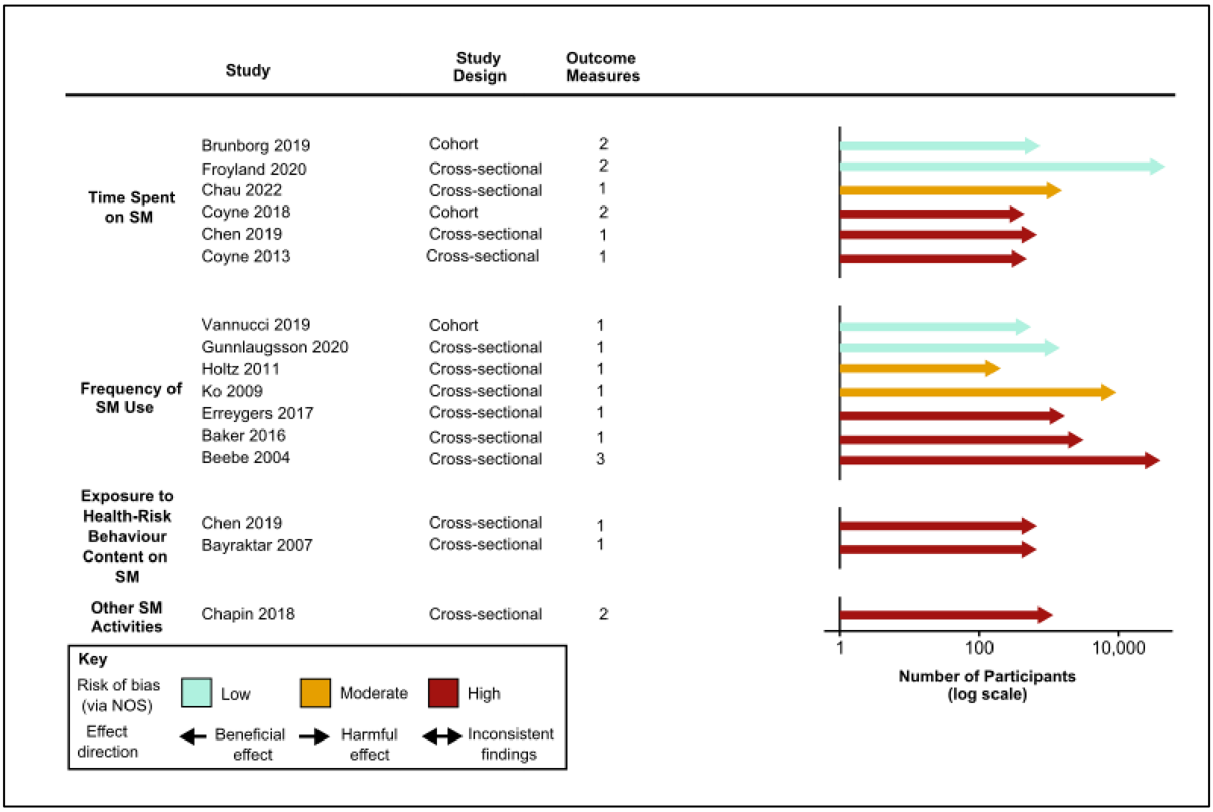

Legend: Sample size: represented by the size of the arrow, measured on a log scale. Outcome measure: number of outcome measures synthesised within each study. Studies organised by risk of bias grade, study design, and year of publication. Repeat cross-sectional studies, multiple study populations from different countries, and age subsets originating from the same study reported as separate studies. Abbreviations: NOS = Assessed via adapted Newcastle Ottawa Scale; and SM = Social media.

Forest plots for meta-analyses and subgroup analyses

Figure BF. Forest plot for association between frequency of social media use and anti-social behaviour

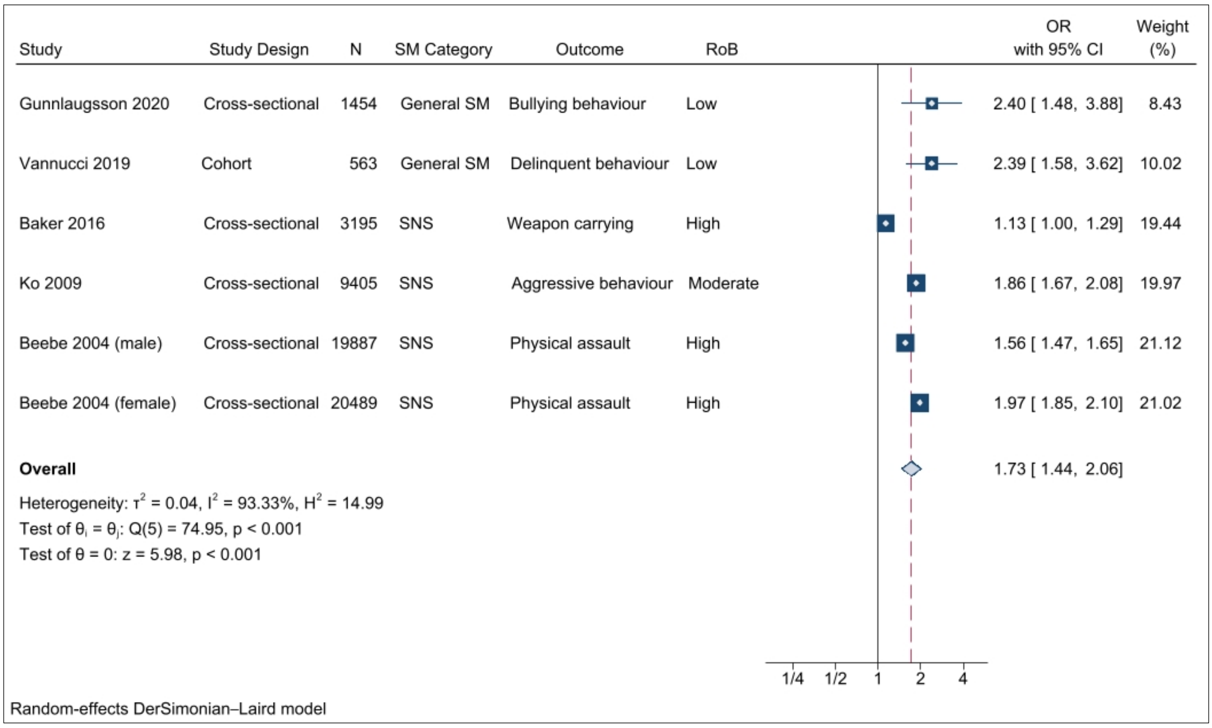

Legend: Figure presents forest plot for binary exposure (frequent/at all vs infrequent/not at all) & binary/continuous outcome meta-analysis, with odds (OR) used as common metric. Total number of study participants = 54,993. Abbreviations: CI = Confidence interval; N = Number of study participants; OR = Odds ratio; RoB = Risk of bias; SM = Social media; and SNS = Social networking sites.

Figure BG. Forest plot for association between time spent on social media and anti-social behaviour

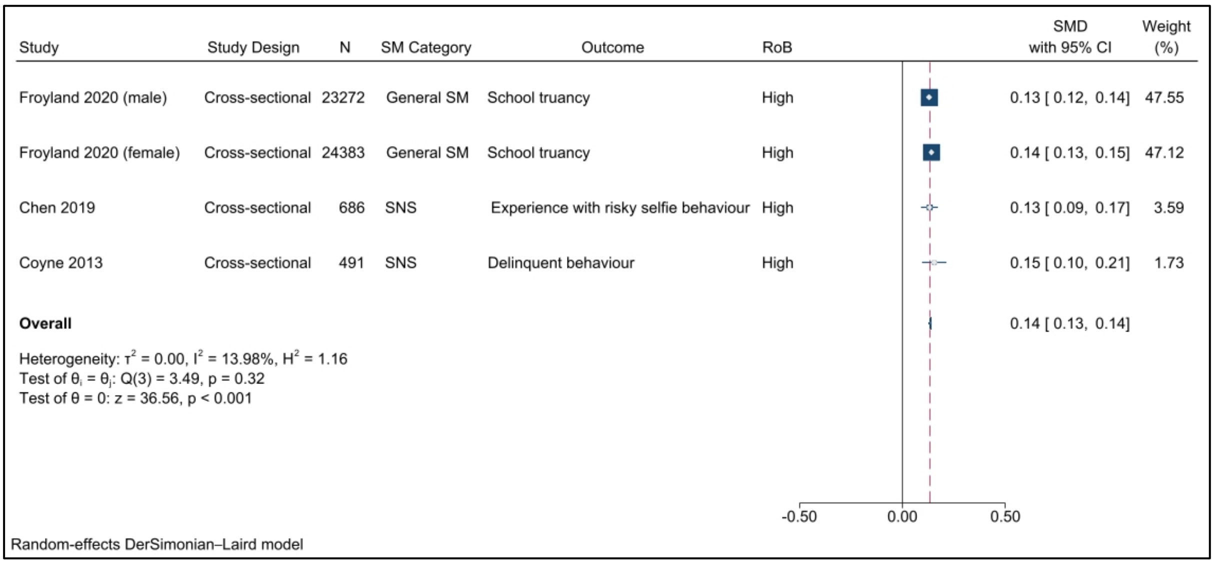

Legend: Figure presents forest plot for continuous exposure & continuous outcome meta-analysis, with standardised mean difference (SMD) used as common metric. Total number of study participants = 48,832. Abbreviations: CI = Confidence interval; N = Number of study participants; RoB = Risk of bias; SM = Social media; SMD = Standardised mean difference; and SNS = Social networking sites.

Figure BH. Forest plot for association between frequency of social media use and anti-social behaviour, by development status of study setting[a]

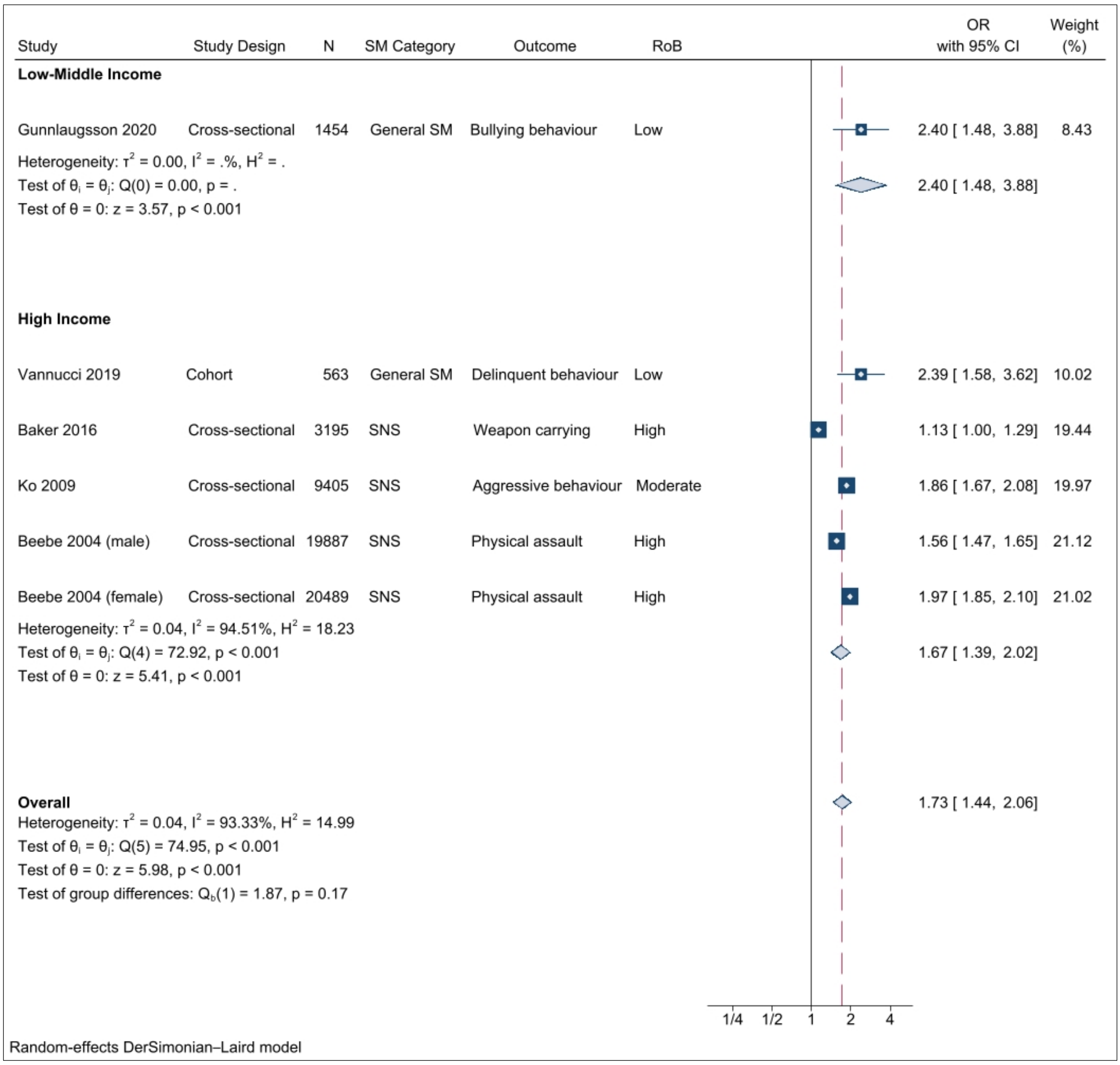

Legend: Figure presents forest plot for binary exposure (frequent/at all vs infrequent/not at all) & binary/continuous outcome subgroup analysis, with odds (OR) used as common metric. [a] Development status classified as per the World Bank Country Income Level Classification.[160] Total number of study participants = 54,993. Abbreviations: CI = Confidence interval; N = Number of study participants; OR = Odds ratio; RoB = Risk of bias; SM = Social media; and SNS = Social networking sites

Figure BI. Forest plot for association between frequency of social media use and anti-social behaviour, by social media category

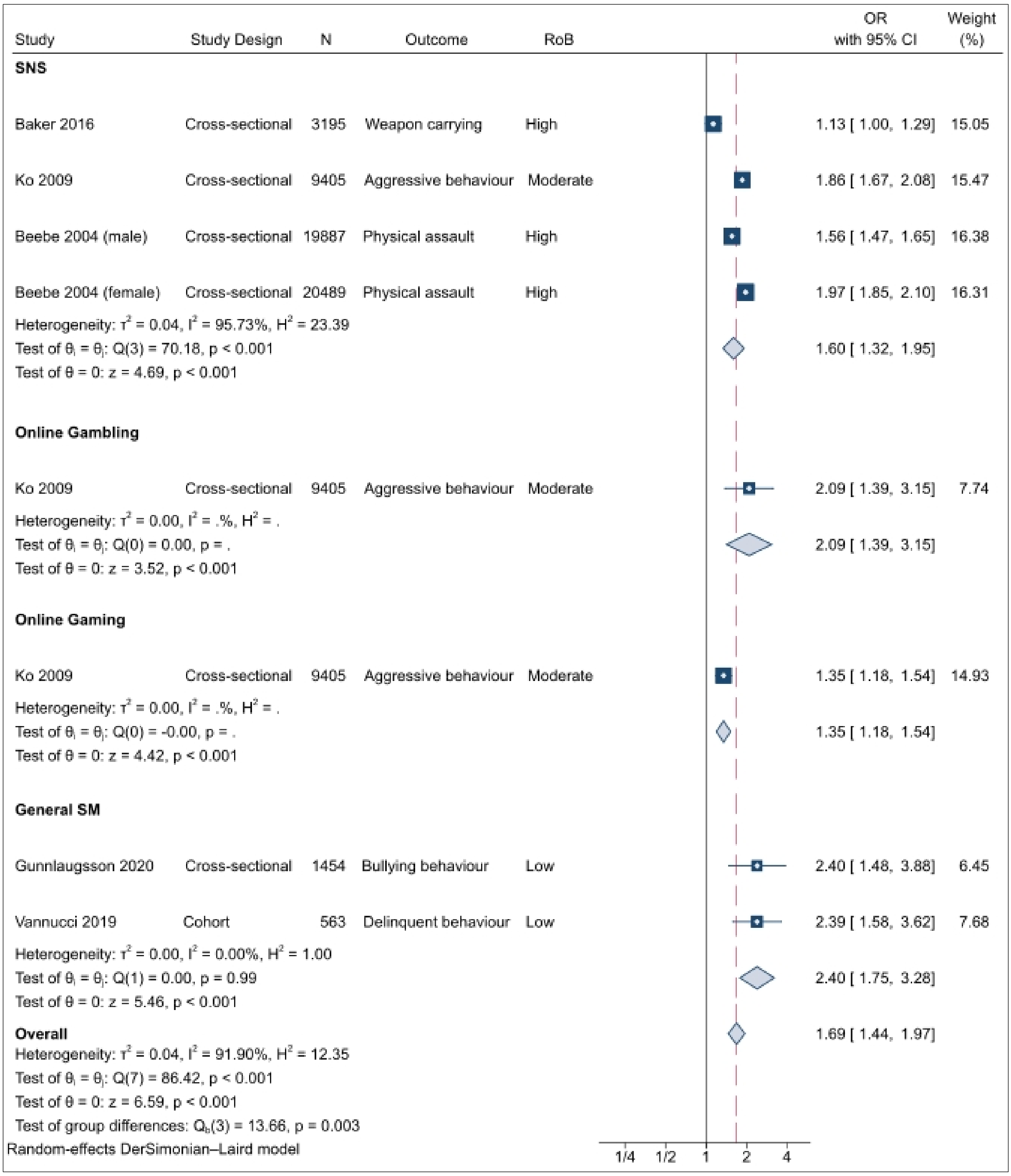

Legend: Figure presents forest plot for binary exposure (frequent/at all vs infrequent/not at all) & binary/continuous outcome subgroup analysis, with odds (OR) used as common metric. Total number of study participants = 73,803. Abbreviations: CI = Confidence interval; N = Number of study participants; OR = Odds ratio; RoB = Risk of bias; SM = Social media; and SNS = Social networking site

Figure BJ. Forest plot for association between time spent on social media and anti-social behaviour, by social media category

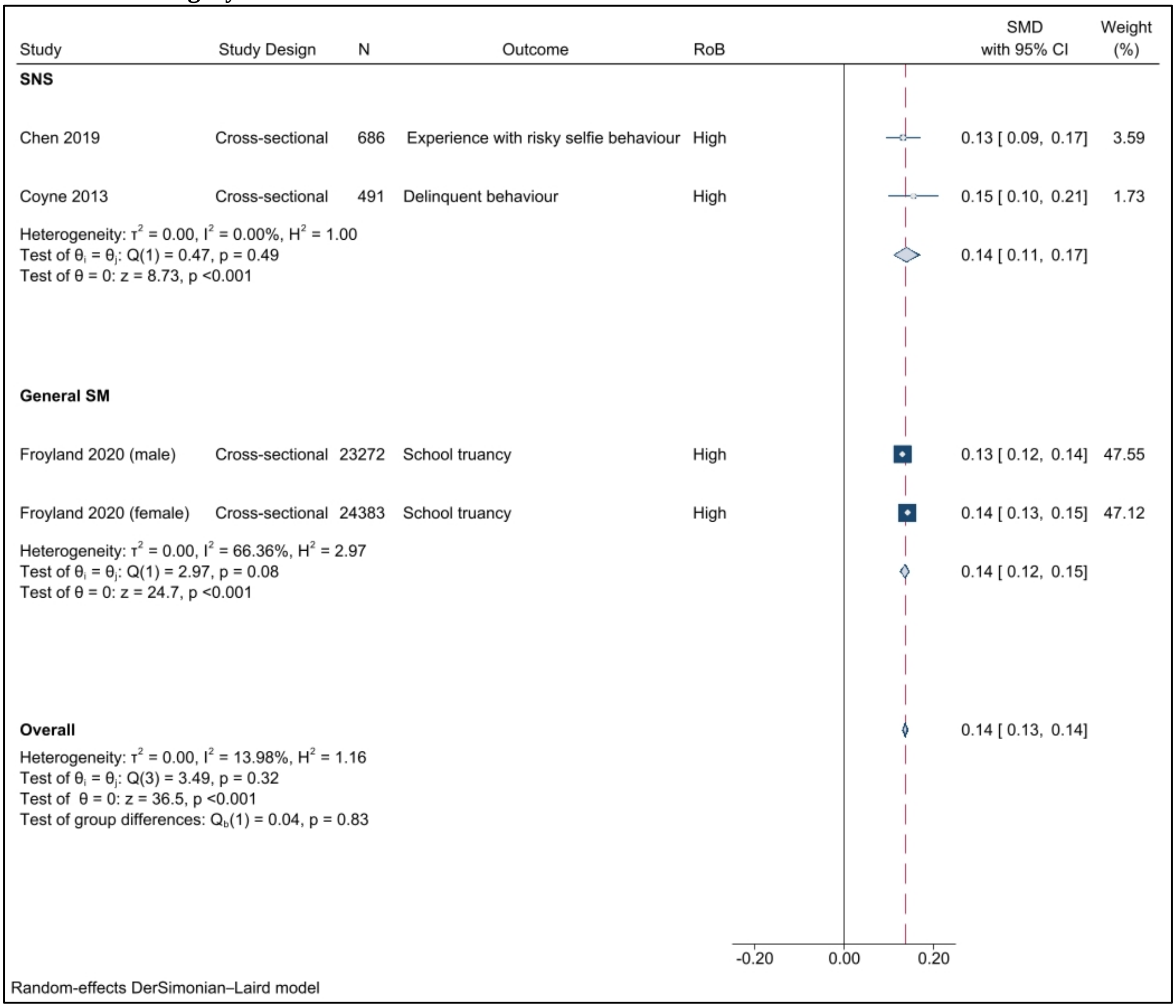

Legend: Figure presents forest plot for continuous exposure & continuous outcome meta-analysis, with standardised mean difference (SMD) used as common metric. Total number of study participants = 48,832. Abbreviations: CI = Confidence interval; N = Number of study participants; RoB = Risk of bias; SM = Social media; SMD = Standardised mean difference; and SNS = Social networking sites.

Inadequate physical activity

Effect direction plot

Figure BK demonstrates the effect direction in those studies (n=14) investigating inadequate physical activity, by exposure. After excluding those with inconsistent findings (n=3),[112,137,95] for time spent on social media, 4/8 of studies reported harmful associations (95% CI 21.5 to 78.5%; participant n = 52,475; sign test p=1.00), whilst for frequency of social media use no study reported a harmful association (0.00 to 56.1%; study n=3; participant n=57,953; sign test p=0.25).

Figure BK. Effect direction plot for studies of the association between social media use and adolescent inadequate physical activity, by social media exposure. Arrow size indicates sample size; arrow colour indicates study risk of bias.

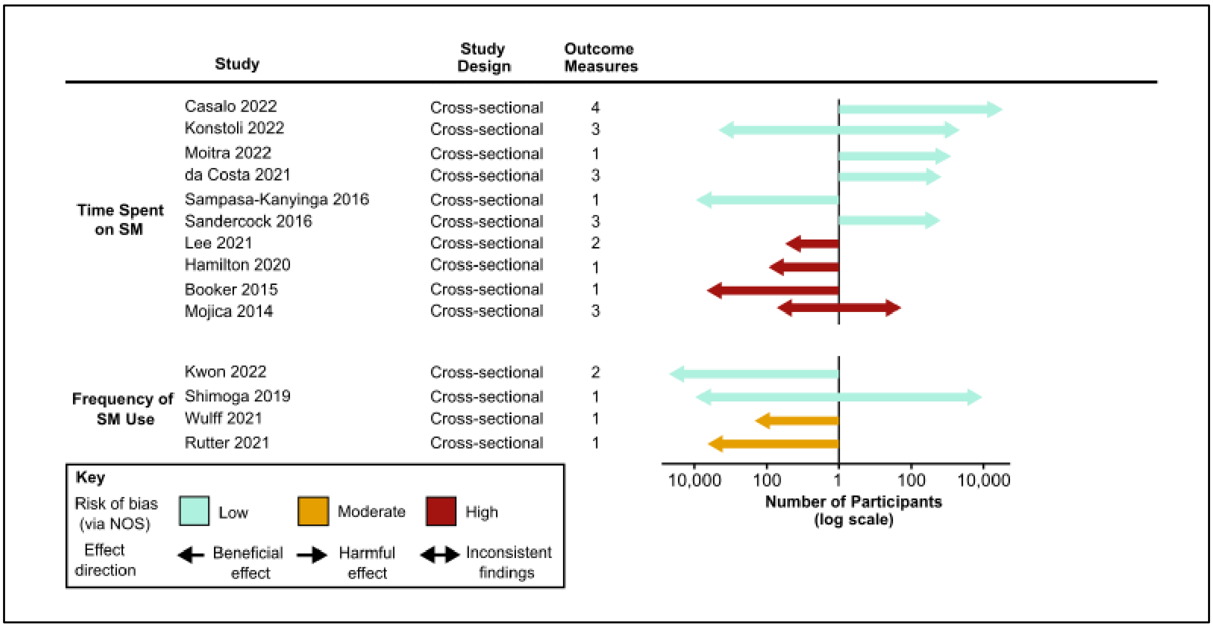

Legend: Sample size: represented by the size of the arrow, measured on a log scale. Outcome measure: number of outcome measures synthesised within each study. Studies organised by risk of bias grade, study design, and year of publication. Repeat cross-sectional studies, multiple study populations from different countries, and age subsets originating from the same study reported as separate studies. Shimoga 2019 assessed frequency of social media use and one outcome (physical activity) across three subgroups. Two of the three subgroups showed increased frequency of social media resulted in decreased physical activity, and one subgroup showed increased frequency of social media resulted in increased physical activity, thus this study was classified as demonstrating inconsistent findings. Abbreviations: NOS = Assessed via adapted Newcastle Ottawa Scale and SM = Social media.

Forest plots for meta-analyses and subgroup analyses

Figure BL. Forest plot for association between time spent on social media and inadequate physical activity

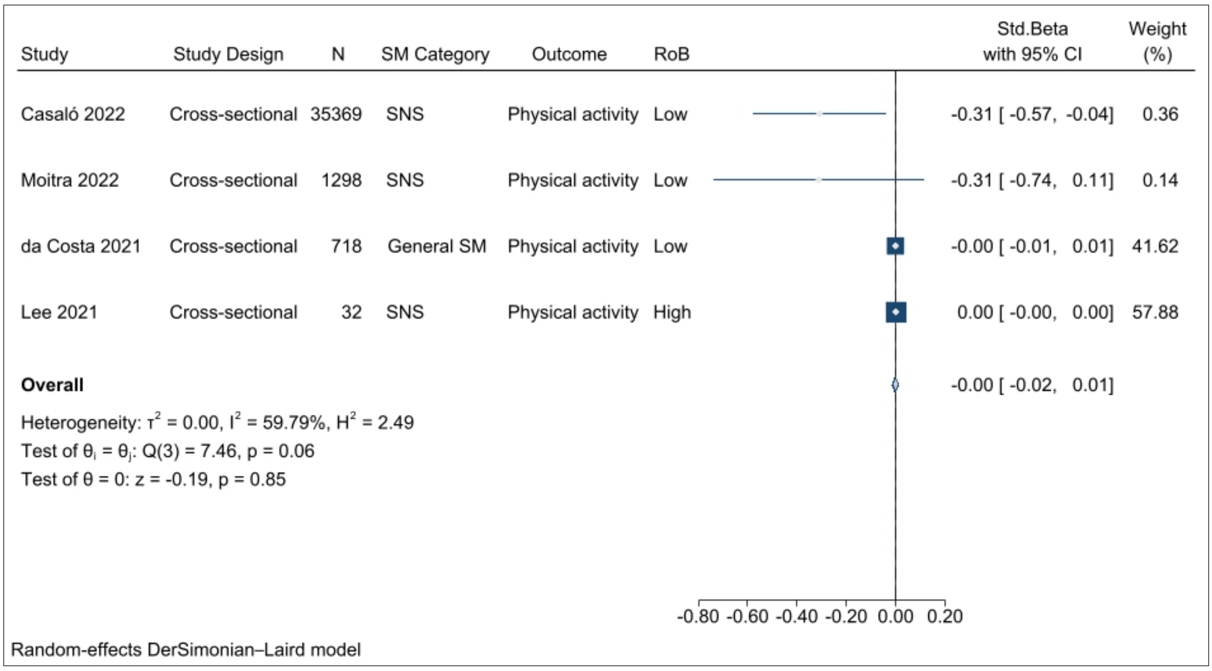

Legend: Figure presents forest plot for continuous exposure & continuous outcome meta-analysis, with standardised beta (Std.Beta) used as common metric. Total number of study participants =37,417. Abbreviations: CI = Confidence interval; N = Number of study participants; RoB = Risk of bias; SM = Social media; SNS = Social networking sites; and Std. Beta = Standardised beta.

Figure BM. Forest plot for association between time spent on social media and inadequate physical activity, by average age of study participants

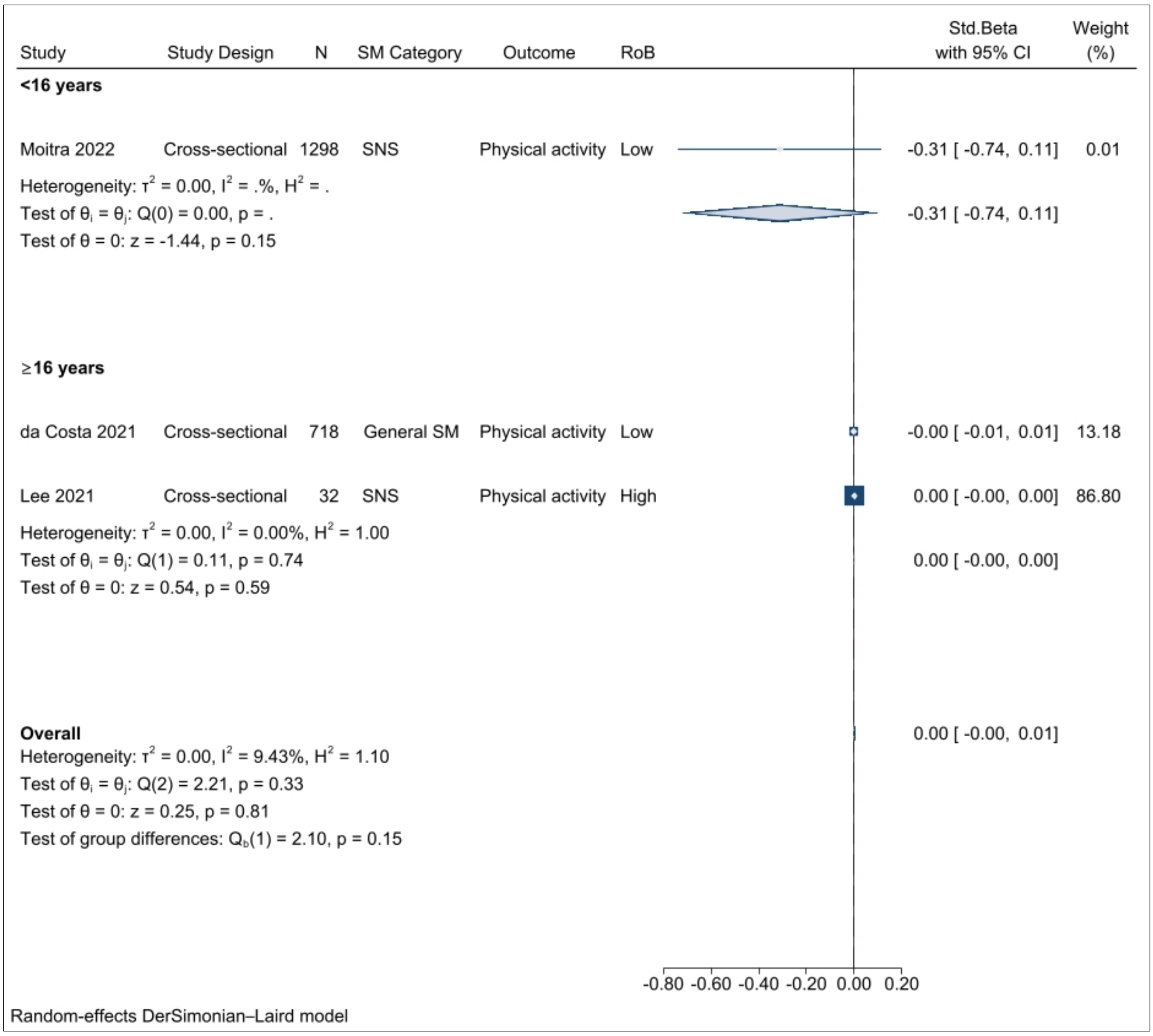

Legend: Figure presents forest plot for continuous exposure & continuous outcome subgroup analysis, with standardised beta (Std. Beta) used as common metric. Total number of study participants = 2,048. Abbreviations: CI = Confidence interval; N = Number of study participants; RoB = Risk of bias; SM = Social media; SNS = Social networking sites; and Std. Beta = Standardised beta.

Figure BN. Forest plot for association between time spent on social media and inadequate physical activity, by development status of study setting[a]

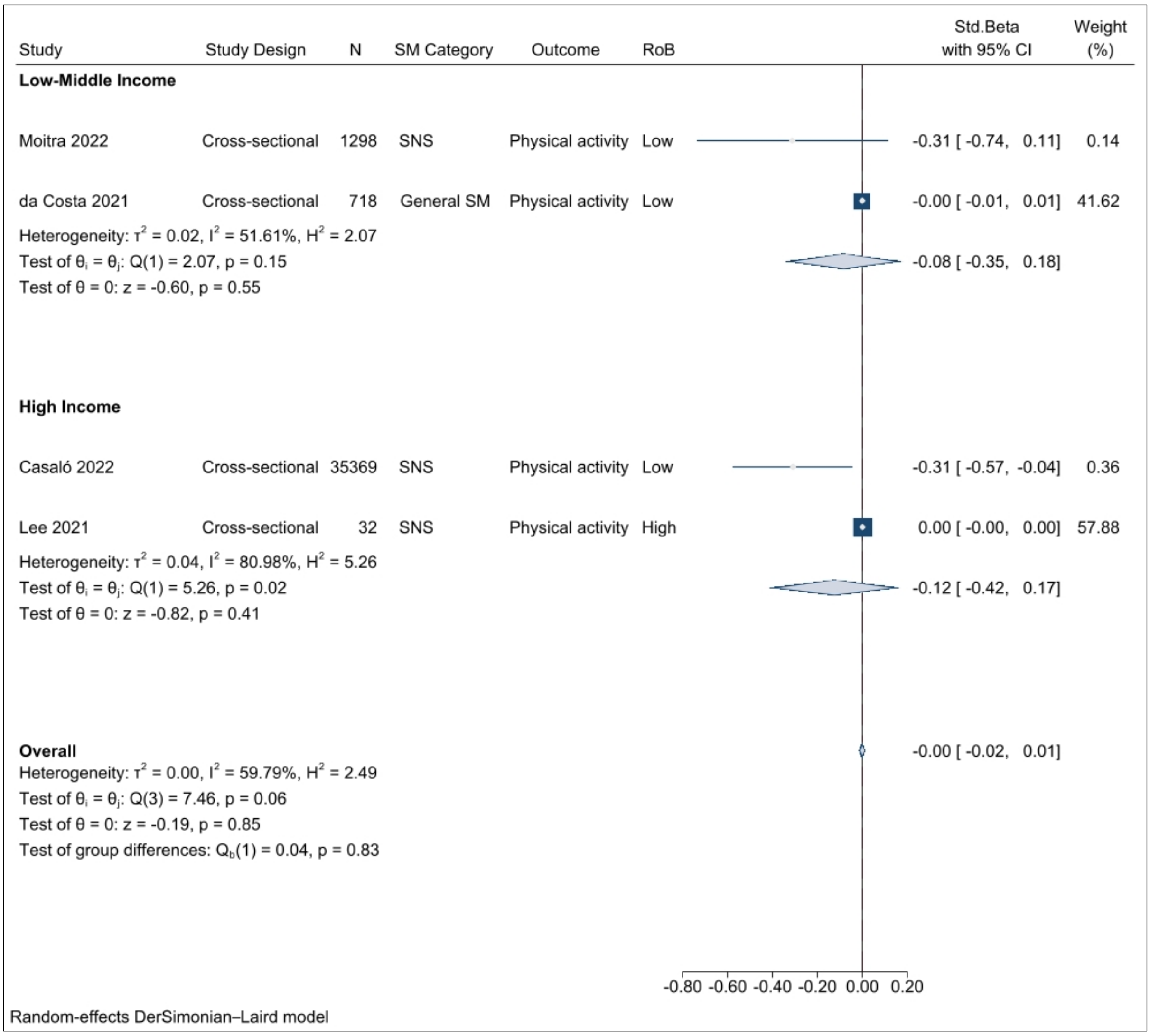

Legend: Figure presents forest plot for continuous exposure & continuous outcome subgroup analysis, with standardised beta (Std. Beta) used as common metric. [a] Development status classified as per the World Bank Country Income Level Classification.[160] Total number of study participants = 37,417. Abbreviations: CI = Confidence interval; N = Number of study participants; RoB = Risk of bias; SM = Social media; SNS = Social networking sites; and Std. Beta = Standardised beta.

Figure BO. Forest plot for association between time spent on social media and inadequate physical activity, by social media category

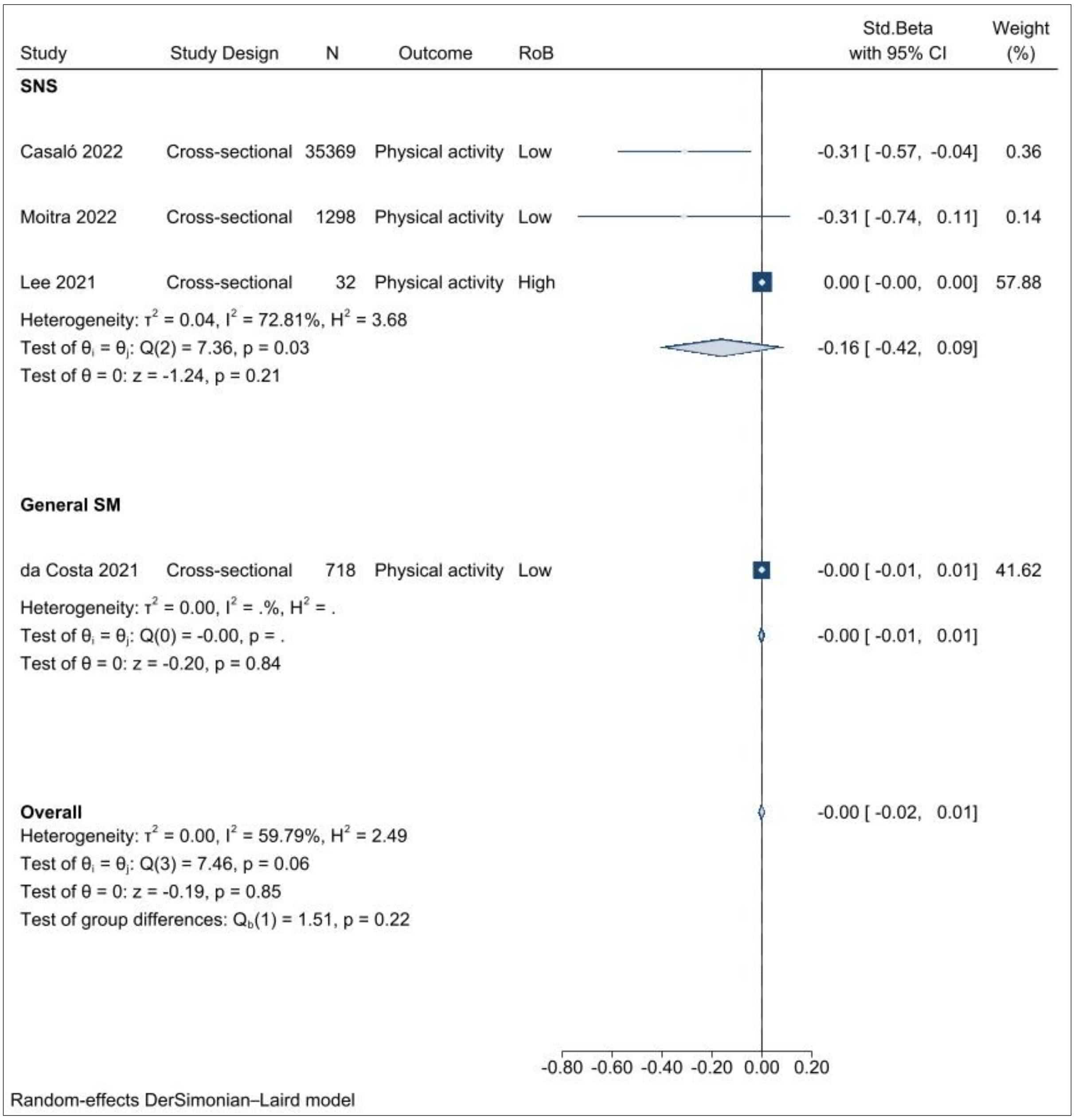

Legend: Figure presents forest plot for continuous exposure & continuous outcome subgroup analysis, with standardised beta (Std. Beta) used as common metric. Total number of study participants = 37,417. Abbreviations: CI = Confidence interval; N = Number of study participants; RoB = Risk of bias; SM = Social media; SNS = Social networking sites; and Std. Beta = Standardised beta.

Unhealthy dietary behaviour

Effect direction plot

Figure BP demonstrate the effect direction in those studies investigating unhealthy dietary behaviours, by exposure. Two studies investigated more than one exposure.[34,161] For time spent on social media and frequency of social media use, all studies reported harmful associations (time spent on social media: 95% CI 51.0 to 100.0%, study n=4, participant n=12,006, sign test p=0.13; frequency of social media use: 34.2 to 100.0%, study n=2, participant n = 826, insufficient data to conduct sign test). The relationship between exposure to health-risk behaviour content on social media and unhealthy dietary behaviours was investigated by four RCT's (two rated low risk of bias (RoB) and two rated some concerns, via the Cochrane RoB-2 Tool), and three cross-sectional studies (two rated low RoB and one moderate). Considering all seven studies together, all studies reported harmful associations of social media (64.6 to 100.0%; study n=7; participant n=10,648; sign test p=0.02). When differentiating by study design, all RCT's reported harmful effects (51.0 to 100.0%; study n=4; participant n=521; sign test p=0.13) and all cross-sectional studies reported harmful associations (43.9 to 100.0%; study n=3; participant n=10,127; sign test p=0.25).

Figure BP. Effect direction plot for studies of the association between social media use and adolescent unhealthy dietary behaviour, by social media exposure. Arrow size indicates sample size; arrow colour indicates study risk of bias.

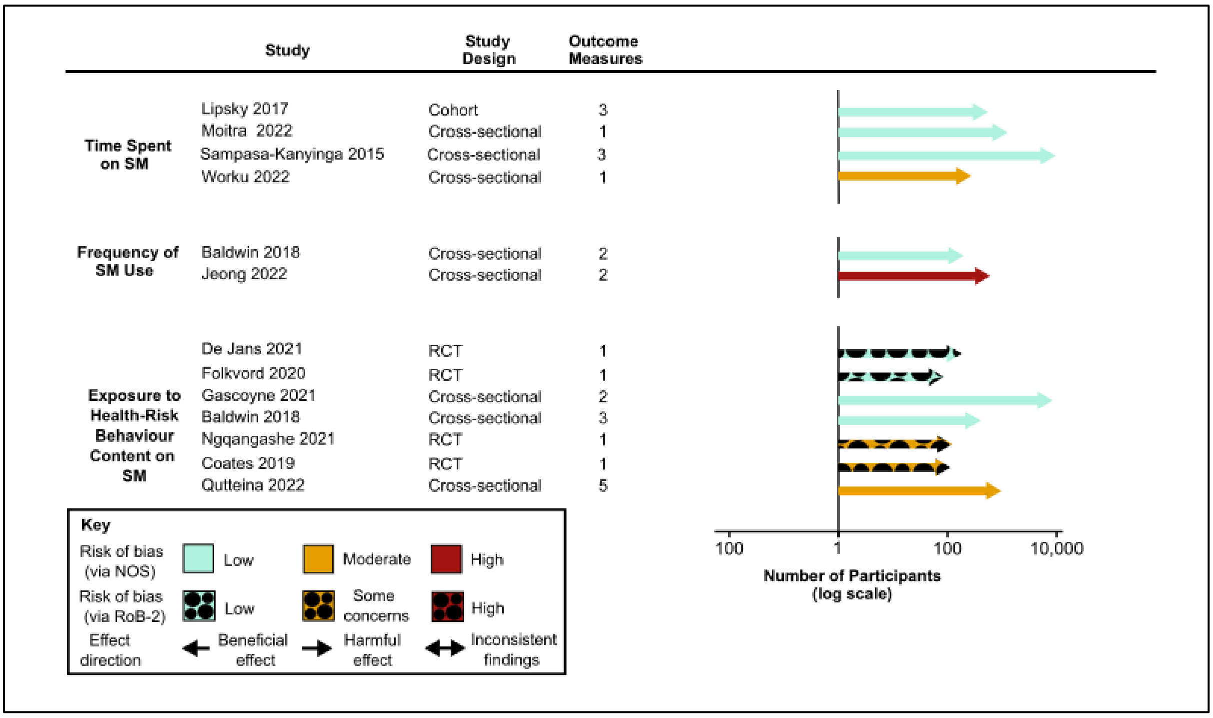

Legend: Sample size: represented by the size of the arrow, measured on a log scale. Outcome measure: number of outcome measures synthesised within each study. Studies organised by risk of bias grade, study design, and year of publication. Repeat cross-sectional studies, multiple study populations from different countries, and age subsets originating from the same study reported as separate studies. Abbreviations: NOS = Assessed via adapted Newcastle Ottawa Scale; RCT = Randomised Control Trial; RoB-2 = Assessed via Cochrane Risk of Bias 2 Tool; and SM = Social media.

Forest plots for meta-analyses and subgroup analyses

Figure BQ. Forest plot for association between exposure to health-risk behaviour content on social media and unhealthy dietary behaviour

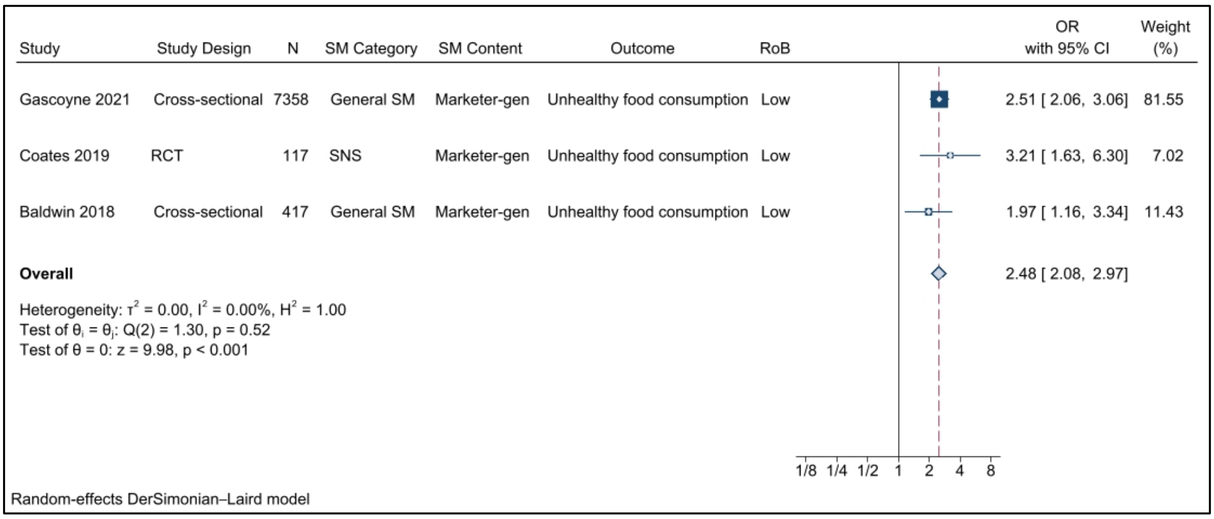

Legend: Figure presents forest plot for binary exposure (exposed vs unexposed) & binary/continuous outcome meta-analysis, with odds ratio (OR) used as common metric. Total number of study participants = 7,892. Abbreviations: CI = Confidence interval; N = Number of study participants; OR = Odds ratio; RoB = Risk of bias; SM = Social media; and SNS = Social networking sites.

Figure BR. Forest plot for association between exposure to health-risk behaviour content on social media and unhealthy dietary behaviour, by social media category

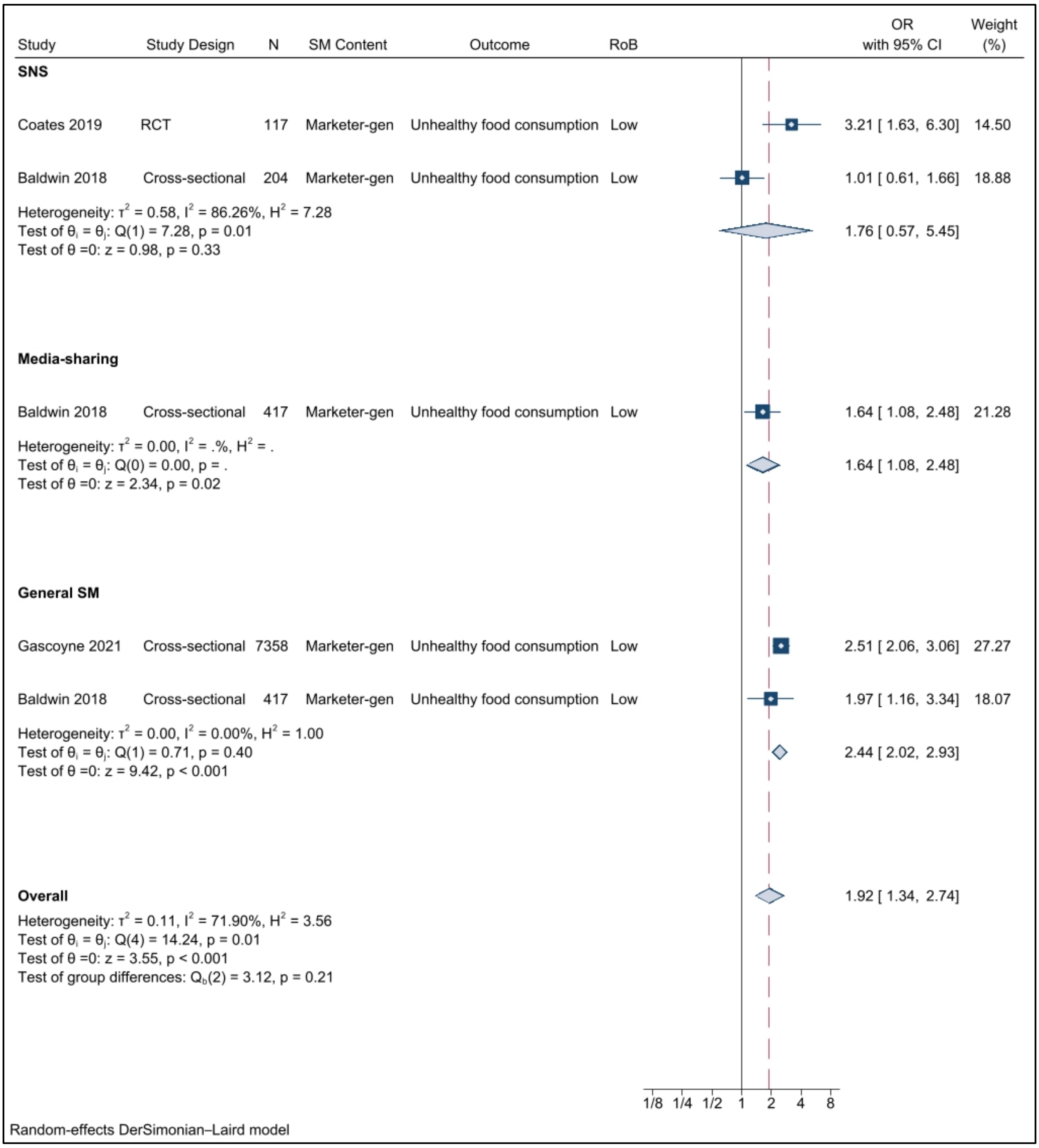

Legend: Figure presents forest plot for binary exposure (exposed vs unexposed) & binary/continuous outcome subgroup analysis, with odds ratio (OR) used as common metric. Total number of study participants = 8,513. Abbreviations: CI = Confidence interval; N = Number of study participants; OR = Odds ratio; RoB = Risk of bias; SM = Social media; and SNS = Social networking sites

Multiple risk behaviours

Effect direction plot

Figure BS demonstrates the effect direction in those studies (n=9) investigating multiple risk behaviours, by exposure. One study investigated more than one exposure type.[115] For time spent on social media, the one study investigated reported a harmful association (95% CI 20.7 to 100.0%; participant n=500; insufficient data to conduct sign test), for frequency of social media use all studies demonstrated harmful associations (51.0 to 100.0%; study n=4; participant n=44,271; sign test p=0.13). Similarly, for exposure to health-risk behaviour content on social media, all studies demonstrated harmful associations (43.9 to 100.0%; study n=3; participant n=16,110,555; sign test p=0.25) and for other social media activities, the one study investigated reported a harmful association (20.7 to 100.0%; participant n=716; insufficient data to conduct sign test).

Figure BS. Effect direction plot for studies of the association between social media use and adolescent engagement in multiple risk behaviours, by social media exposure. Arrow size indicates sample size; arrow colour indicates study risk of bias.

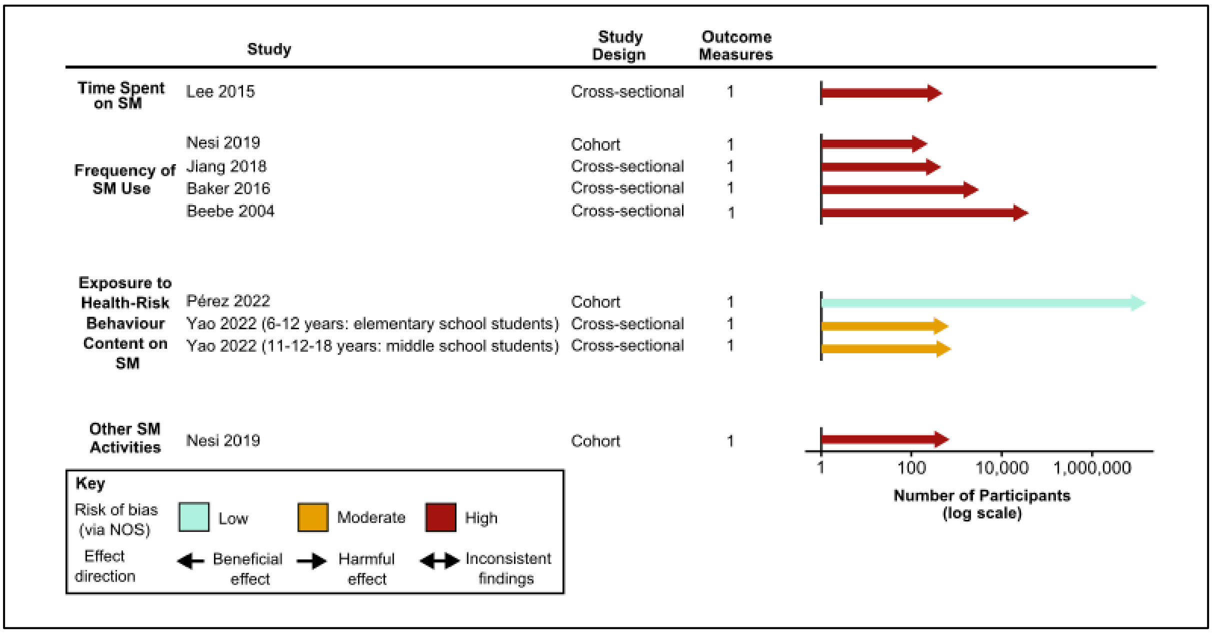

Legend: Sample size: represented by the size of the arrow, measured on a log scale. Outcome measure: number of outcome measures synthesised within each study. Studies organised by risk of bias grade, study design, and year of publication. Repeat cross-sectional studies, multiple study populations from different countries, and age subsets originating from the same study reported as separate studies. Abbreviations: NOS = Assessed via adapted Newcastle Ottawa Scale; and SM = Social media.

<u>Forest plots for meta-analyses and subgroup analyses</u>

Figure BT. Forest plot for association between frequency of social media use and multiple risk behaviours

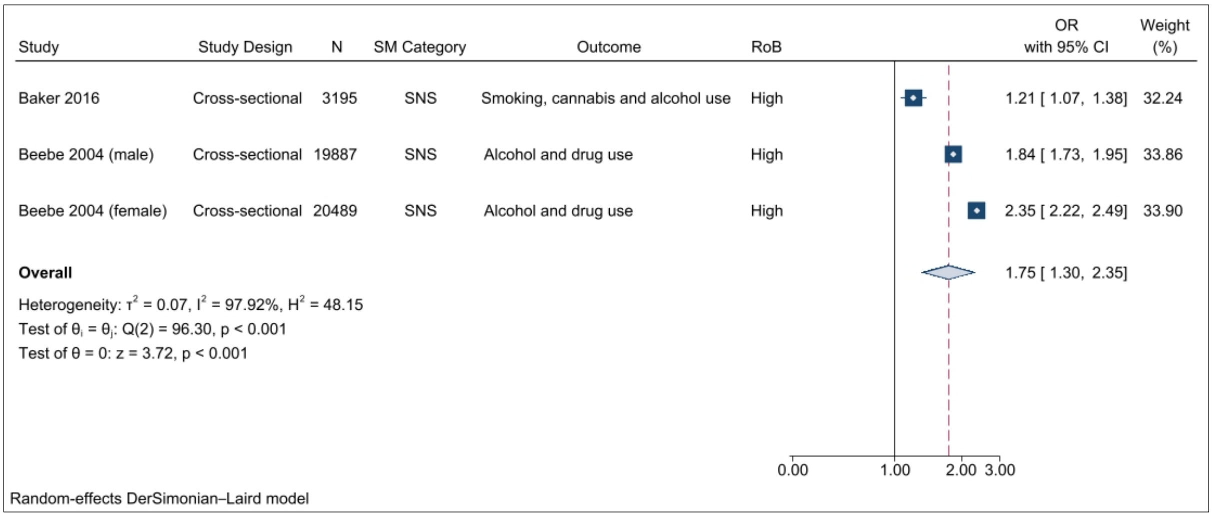

Legend: Figure presents forest plot for binary exposure (frequent/at all vs infrequent/not at all) & binary/continuous outcome meta-analysis, with odds ratio (OR) used as common metric. Total number of study participants = 43,571. Abbreviations: CI = Confidence interval; N = Number of study participants; OR = Odds ratio; RoB = Risk of bias; SM = Social media; and SNS = Social networking sites.

Sensitivity analyses

Figure BU. Forest plot for association between exposure to health-risk behaviour content on social media and use of electronic nicotine delivery systems, by study design

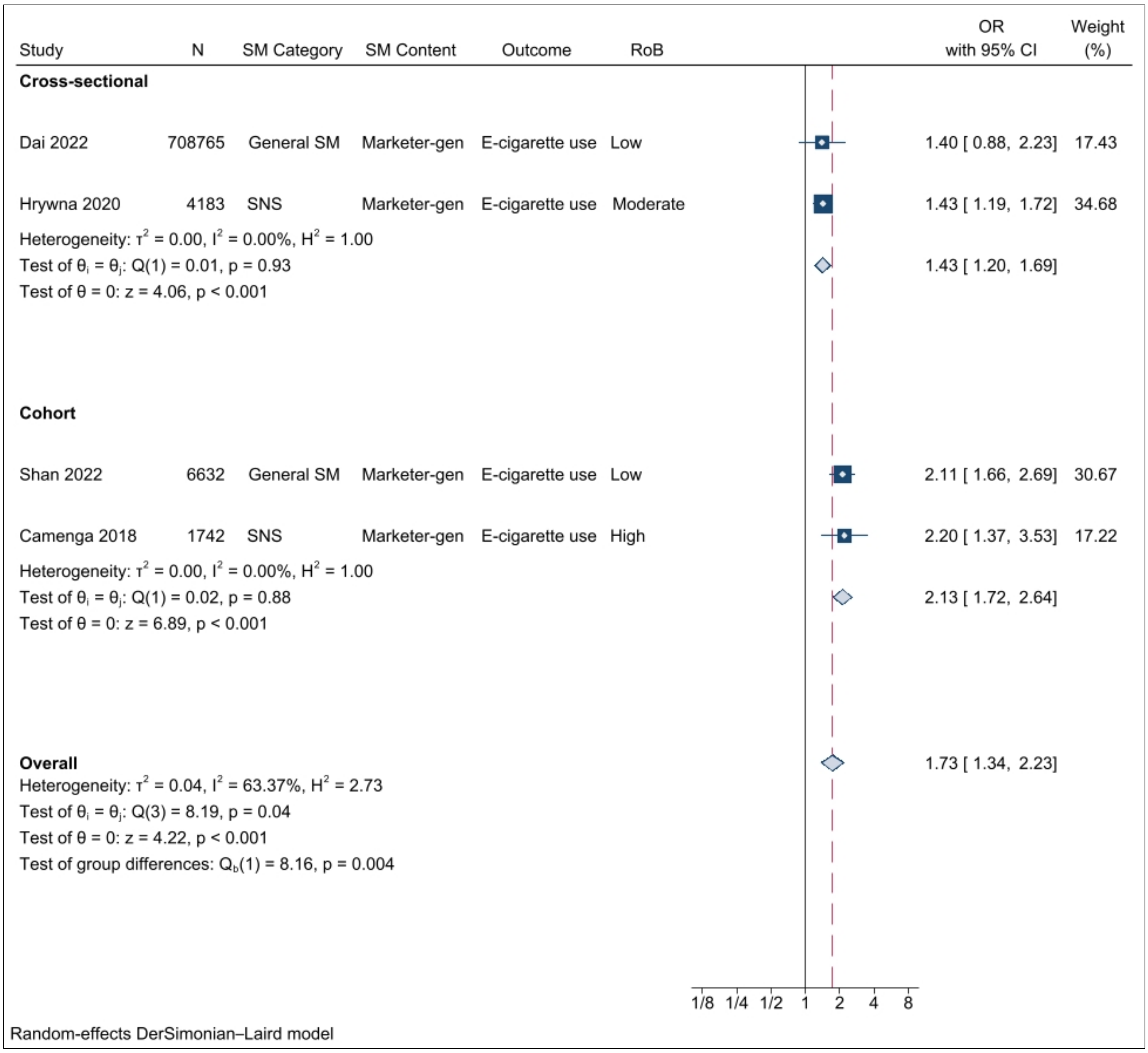

Legend: Figure presents forest plot for binary exposure (exposed vs unexposed) & binary/continuous outcome sensitivity analysis, with odds ratio (OR) used as common metric. Total number of study participants = 721,322. Abbreviations: CI = Confidence interval; N = Number of study participants; OR = Odds ratio; RoB = Risk of bias; SM = Social media; and SNS = Social networking sites

Figure BV. Forest plot for association between frequency of social media use and alcohol use, by study design

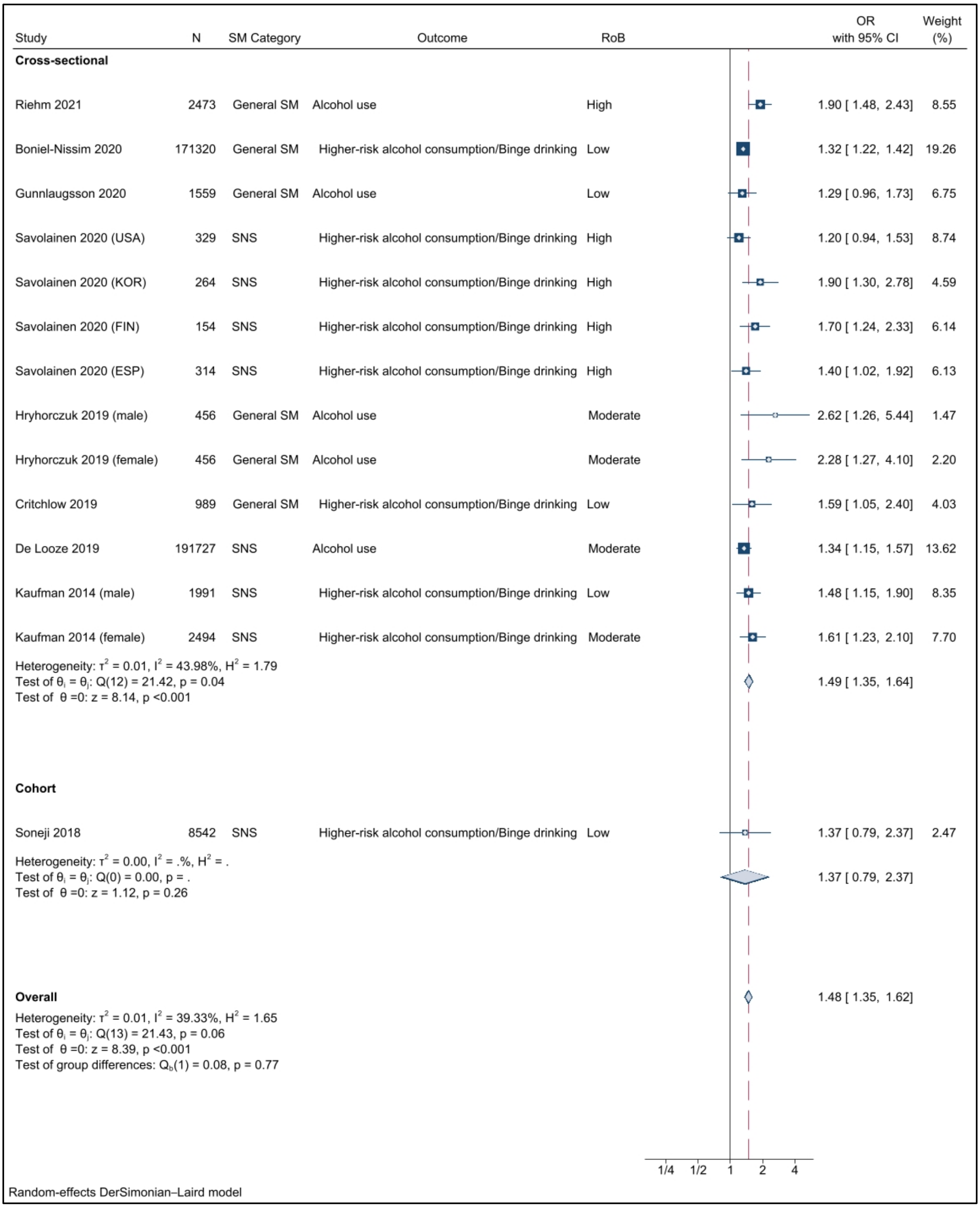

Legend: Figure presents forest plot for binary exposure (frequent/daily vs infrequent/non-daily) & binary/continuous outcome sensitivity analysis, with odds ratio (OR) used as common metric. Total number of study participants = 383,068. Abbreviations: CI = Confidence interval; ESP = Spain; FIN = Finland; KOR = South Korea; N = Number of study participants; OR = Odds ratio; RoB = Risk of bias; SM = Social media; and SNS = Social networking sites.

Figure BW. Forest plot for association between frequency of social media use and alcohol use, by study design

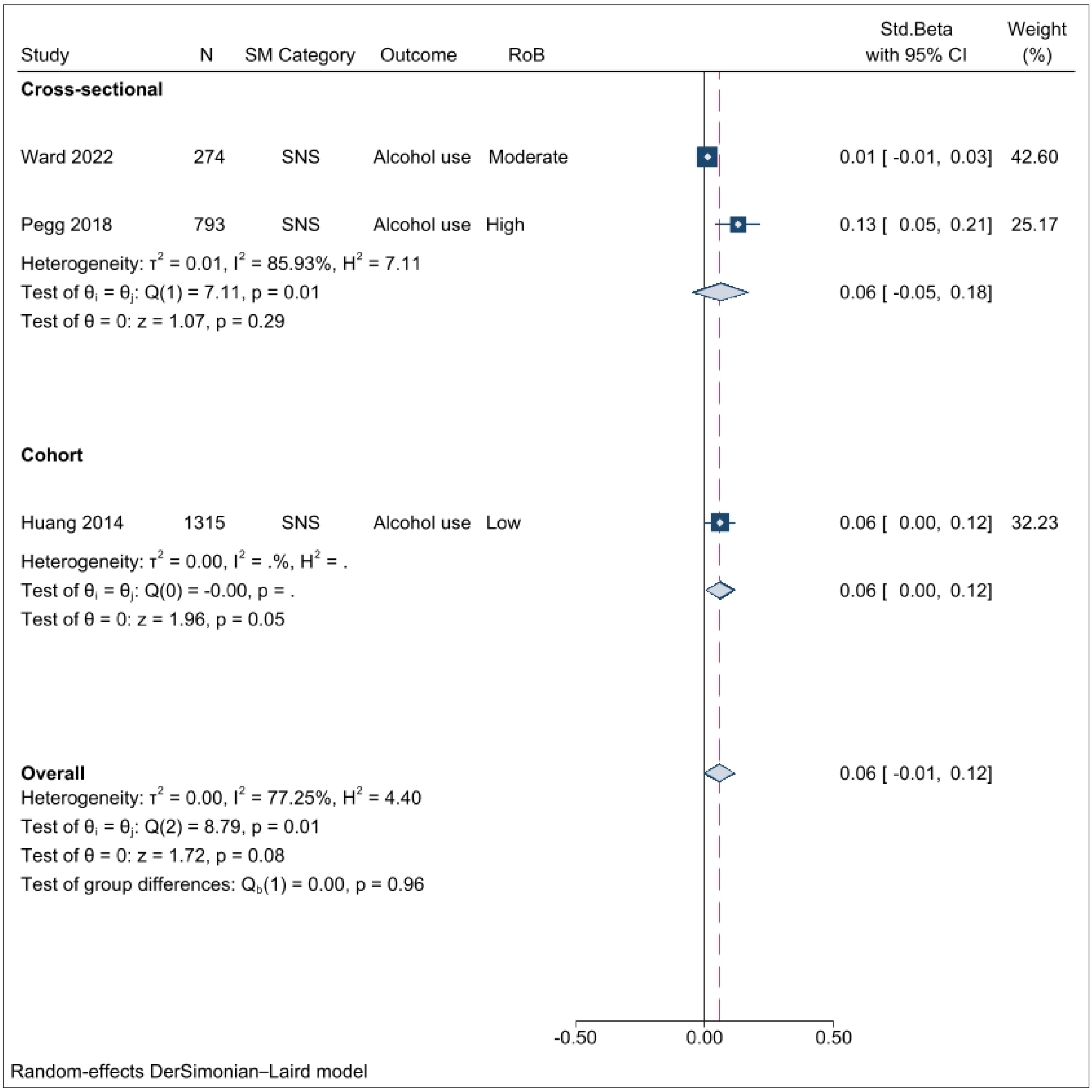

Legend: Figure presents forest plot for continuous exposure & continuous outcome sensitivity analysis, with standardised beta (Std. Beta) used as common metric. Total number of study participants = 2,382. Abbreviation: CI = Confidence interval; N = Number of study participants; RoB = Risk of bias; SM = Social media; SNS = Social networking sites; and Std. Beta = Standardised beta.

Figure BX. Forest plot for association between time spent on social media and alcohol use, by study design

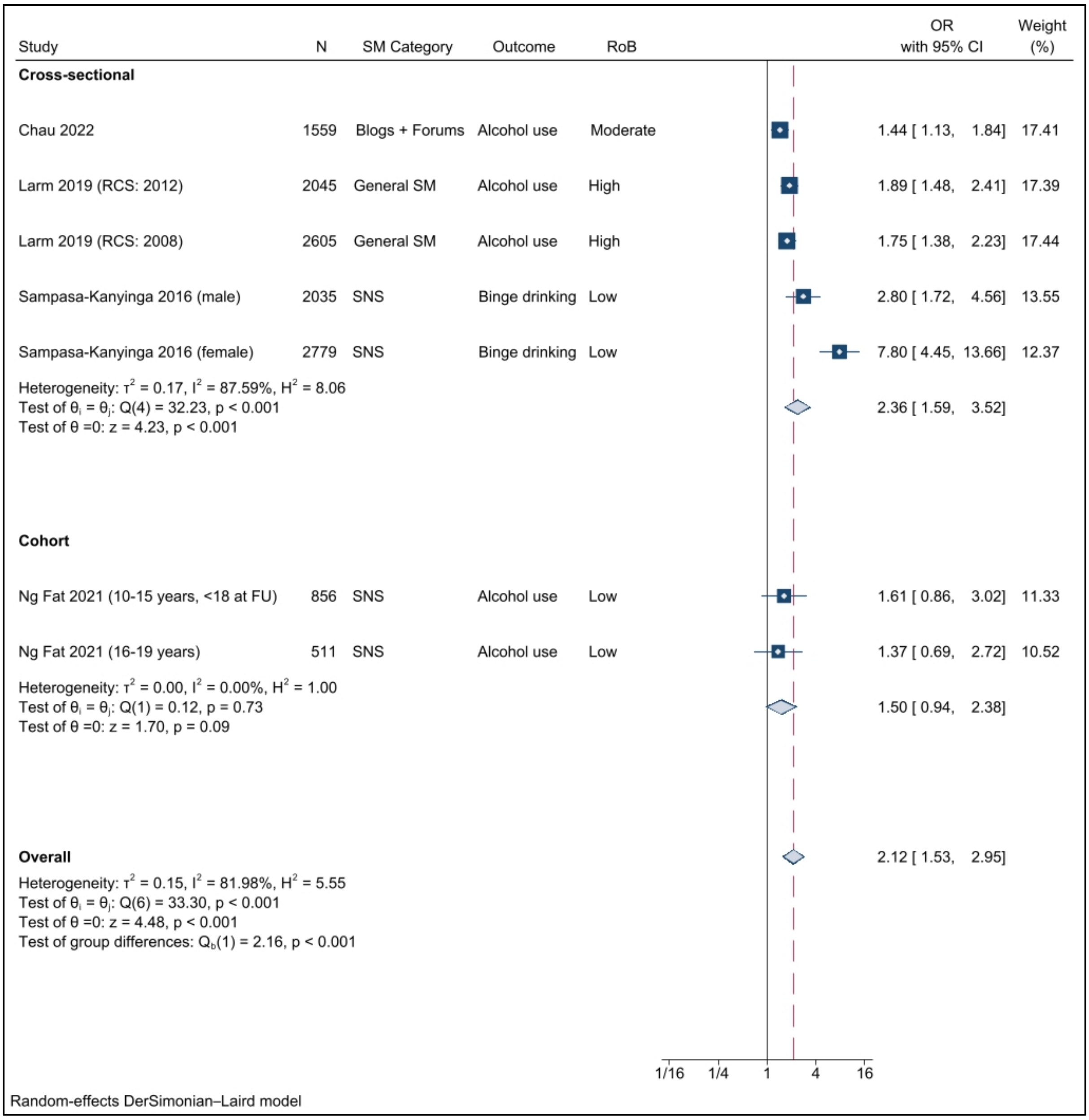

Legend: Figure presents forest plot for binary exposure (≥2 vs <2 hrs/day social media use) & binary/continuous outcome sensitivity analysis, with odds ratio (OR) used as common metric. Total number of study participants = 12,390. Abbreviations: CI = Confidence interval; FU = Follow up; hrs = Hours; N = Number of study participants; OR = Odds ratio; RCS = Repeat cross-sectional study; RoB = Risk of bias; SM = Social media; and SNS = Social networking sites.

Figure BY. Forest plot for association between time spent on social media and alcohol use, by study design

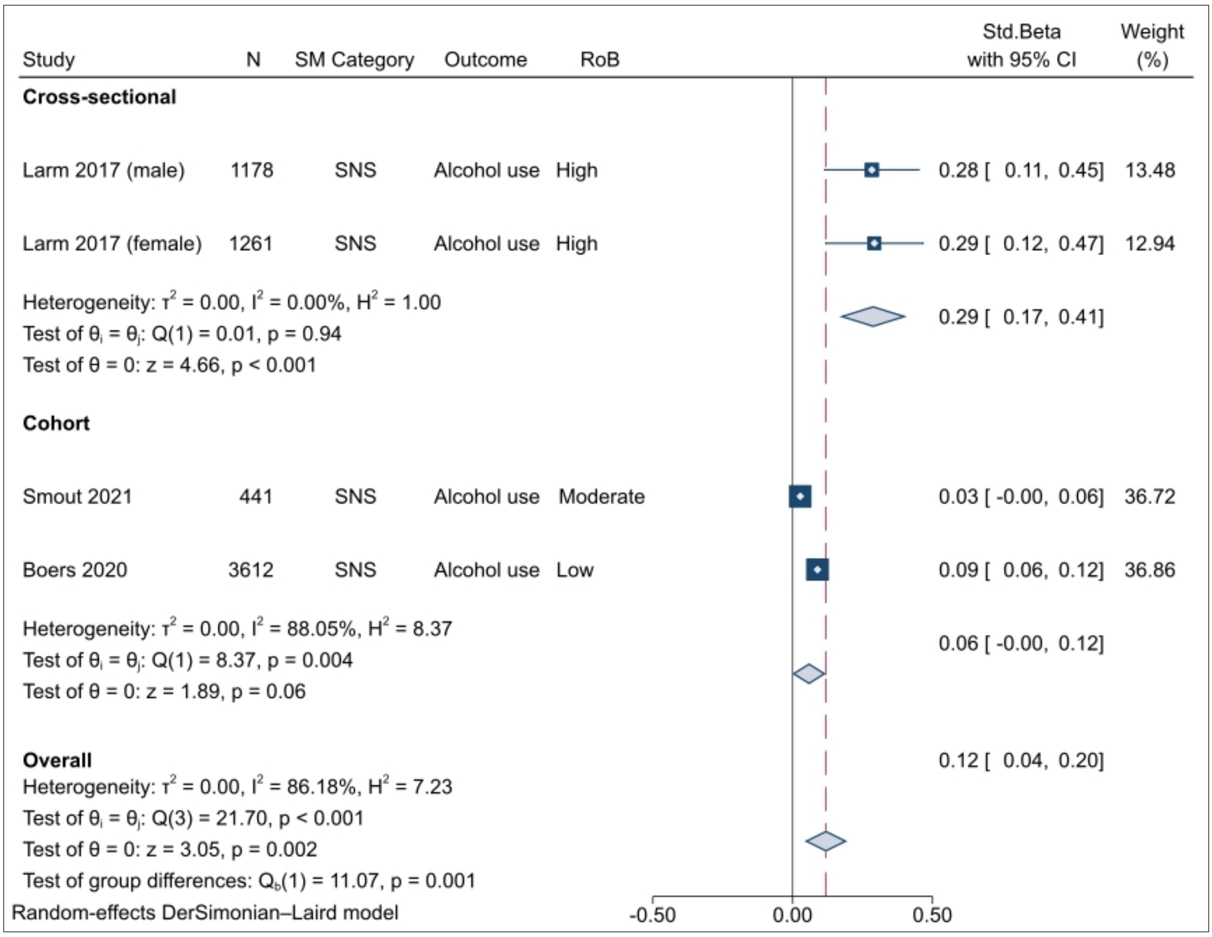

Legend: Figure presents forest plot for continuous exposure & continuous outcome sensitivity analysis, with standardised beta (Std. Beta) used as common metric. Total number of study participants = 6,492. Abbreviation: CI = Confidence interval; N = Number of study participants; RoB = Risk of bias; SM = Social media; SNS = Social networking sites; and Std. Beta = Standardised beta.

Figure BZ. Forest plot for association between exposure to health-risk behaviour content on social media and alcohol use, by study design

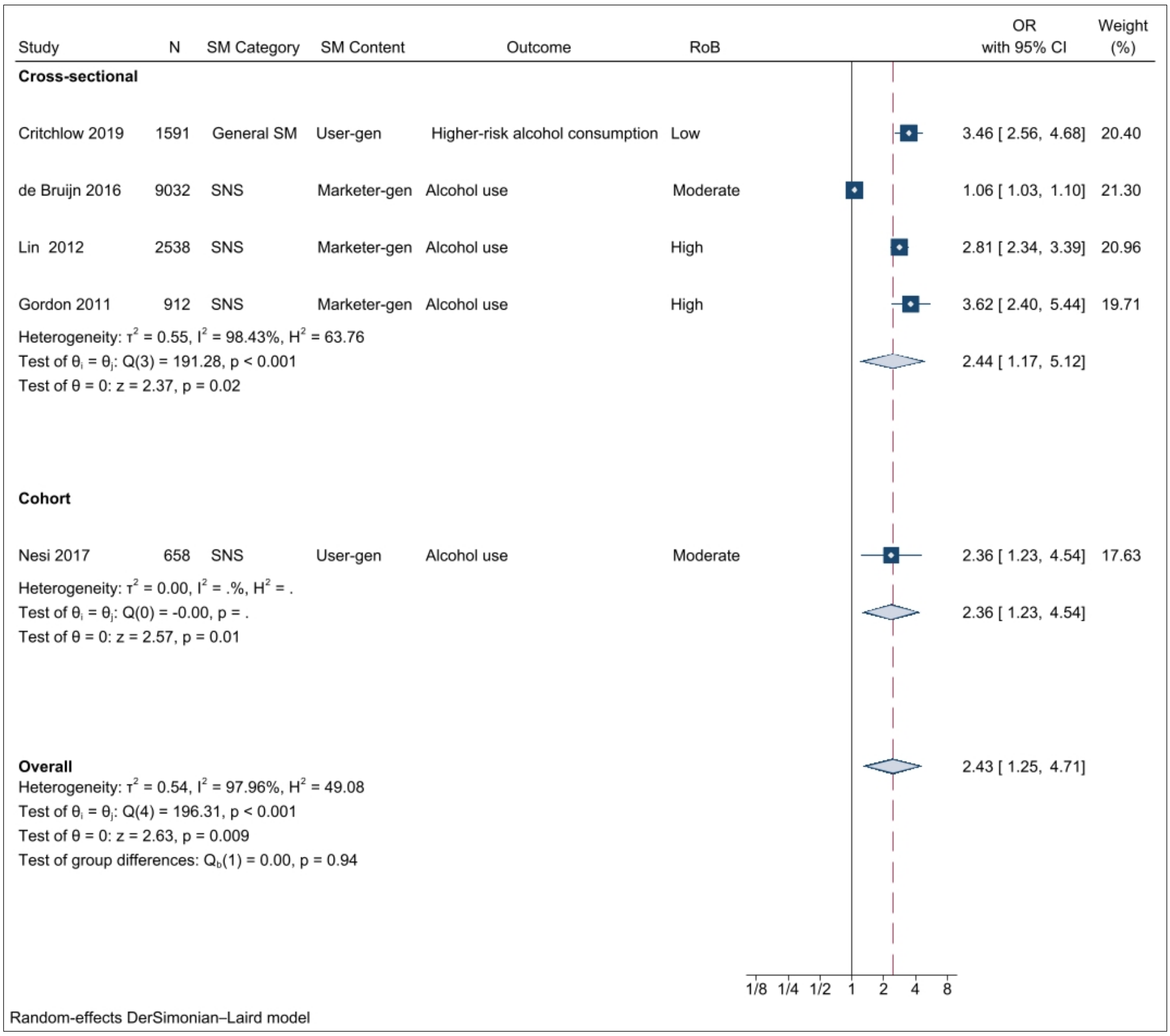

Legend: Figure presents forest plot for binary exposure (exposed vs unexposed) & binary/continuous outcome sensitivity analysis, with odds ratio (OR) used as common metric. Total number of study participants = 14,731. Abbreviations: CI = Confidence interval; Marketer-gen = Marketer-generated content; N = Number of study participants; OR = Odds ratio; RoB = Risk of bias; SM = Social media; SNS = Social networking sites; and User-gen = User-generated content.

Figure CA. Forest plot for associations between exposure to health-risk behaviour content on social media and alcohol use, by study design

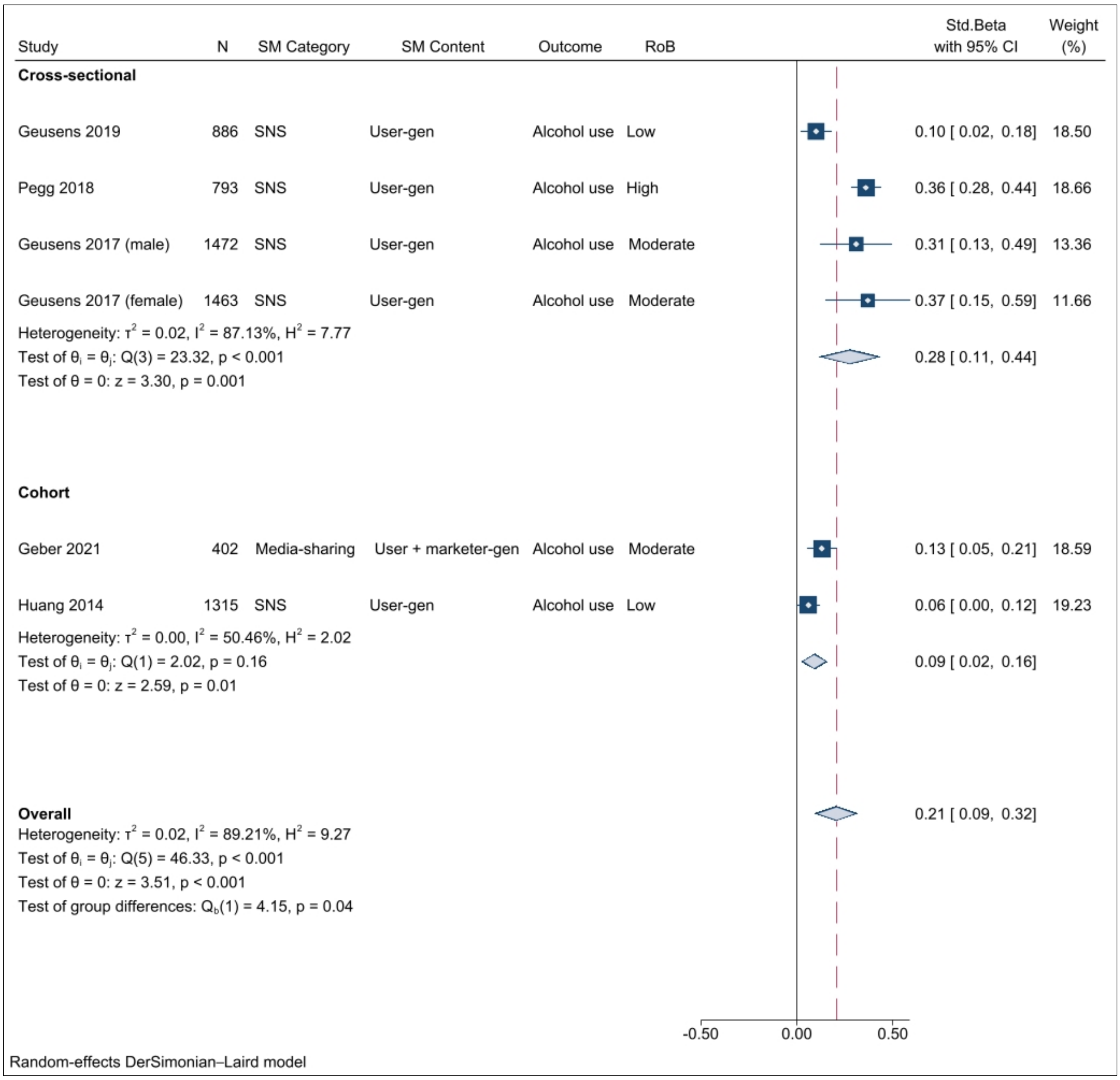

Legend: Figure presents forest plot for continuous exposure & continuous outcome sensitivity analysis, with standardised beta (Std.Beta) used as common metric. Total number of study participants = 6,331. Abbreviation: CI = Confidence interval; N = Number of study participants; Marketer-gen = Marketer-generated content; RoB = Risk of bias; SM = Social media; SNS = Social networking sites; Std. Beta = Standardised beta; and User-gen = User-generated content.

Figure CA. Forest plot for association between frequency of social media use and tobacco use, by study design

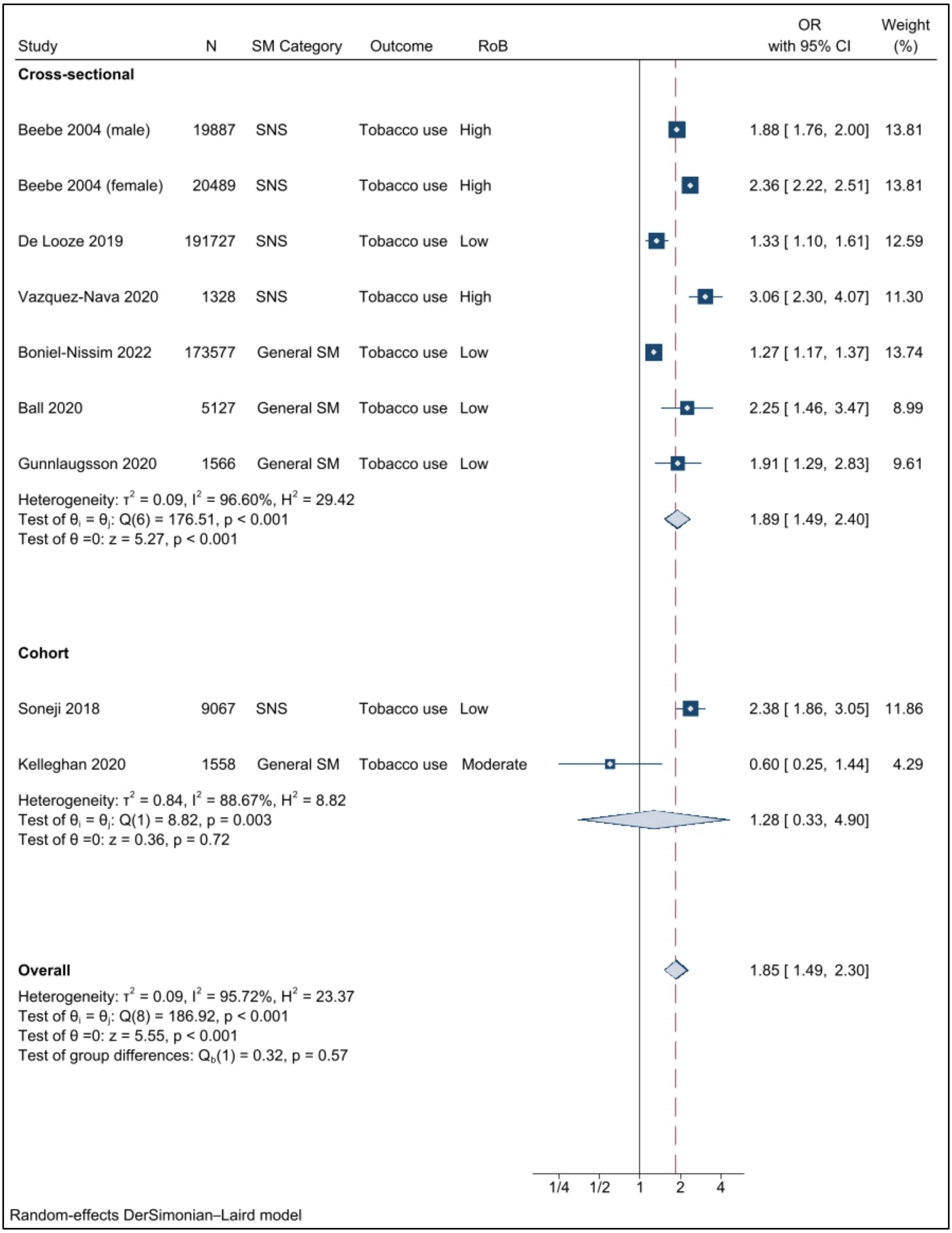

Legend: Figure presents forest plot for binary exposure (frequent vs infrequent) & binary/continuous outcome sensitivity analysis, with odds ratio (OR) used as common metric. Total number of study participants = 424,326. Abbreviations: CI = Confidence interval; N = Number of study participants; OR = Odds ratio; RoB = Risk of bias; SM = Social media; and SNS = Social networking sites.

Figure CB. Forest plot for association between frequency of social media use and drug use, by study design

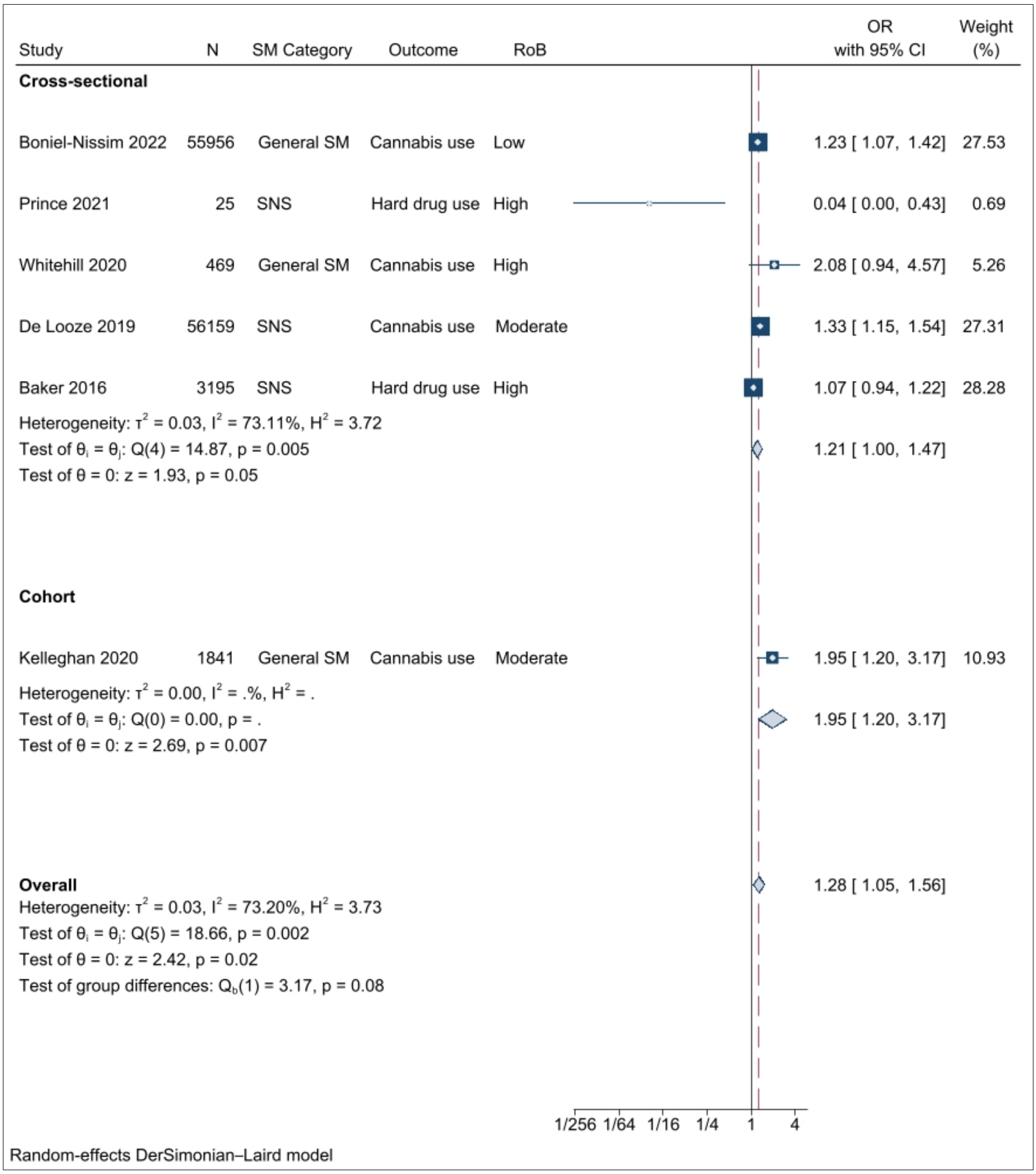

Legend: Figure presents forest plot for binary exposure (frequent/daily vs infrequent/non-daily) & binary/continuous outcome sensitivity analysis, with odds ratio (OR) used as common metric. Total number of study participants = 117,645. Abbreviations: CI = Confidence interval; N = Number of study participants; OR = Odds ratio; RoB = Risk of bias; SM = Social media; and SNS = Social networking sites.

Figure CC. Forest plot for association between exposure to health-risk behaviour content on social media and tobacco use, by study design

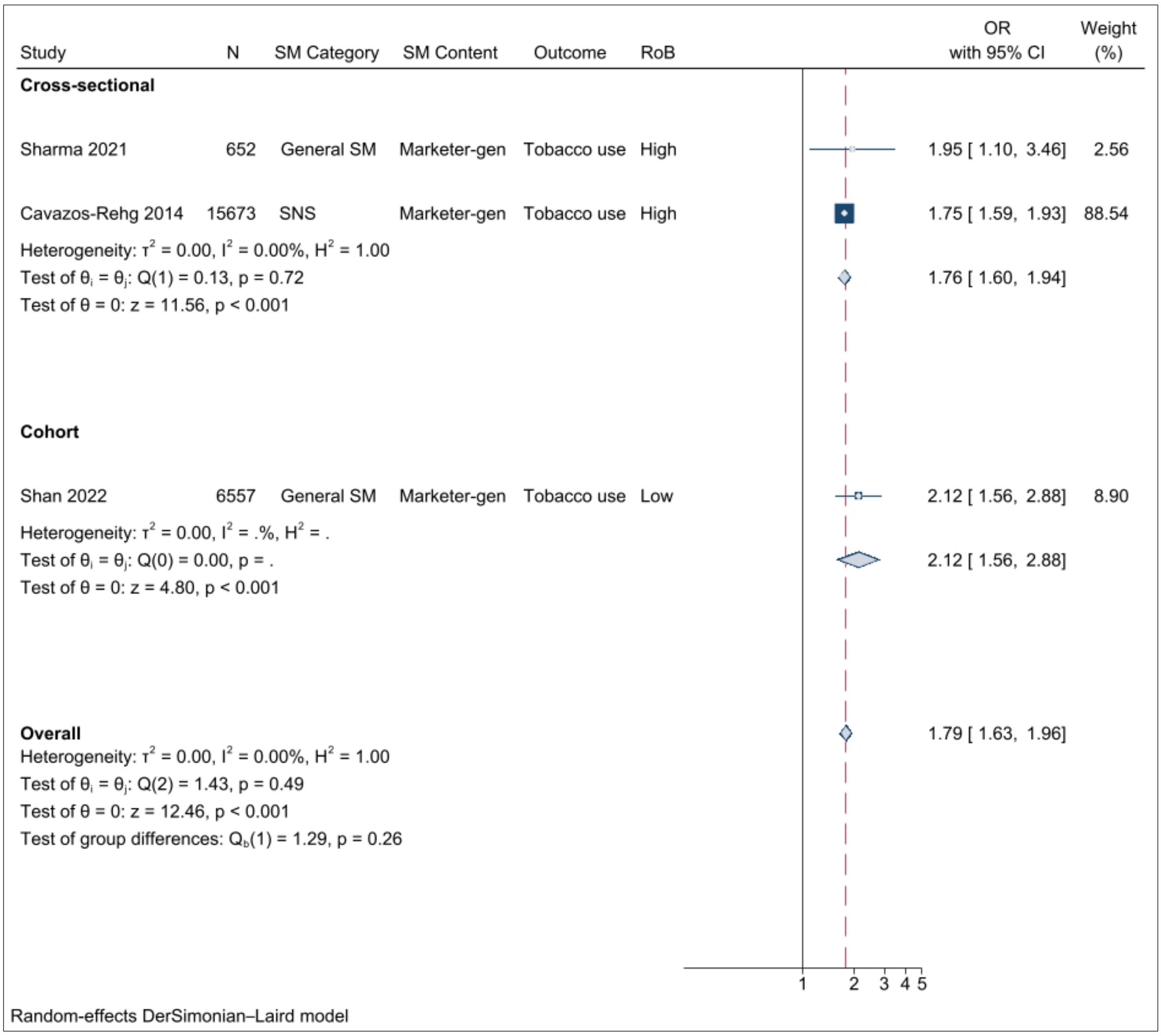

Legend: Figure presents forest plot for binary exposure (exposed vs unexposed) & binary/continuous outcome sensitivity analysis, with odds ratio (OR) used as common metric. Total number of study participants = 22,882. Abbreviations: CI = Confidence interval; Markter-gen = Marketer-generated content; N = Number of study participants; OR = Odds ratio; RoB = Risk of bias; SM = Social media; and SNS = Social networking sites.

Figure CD. Forest plot for association between frequency of social media use and sexual risk behaviour, by study design

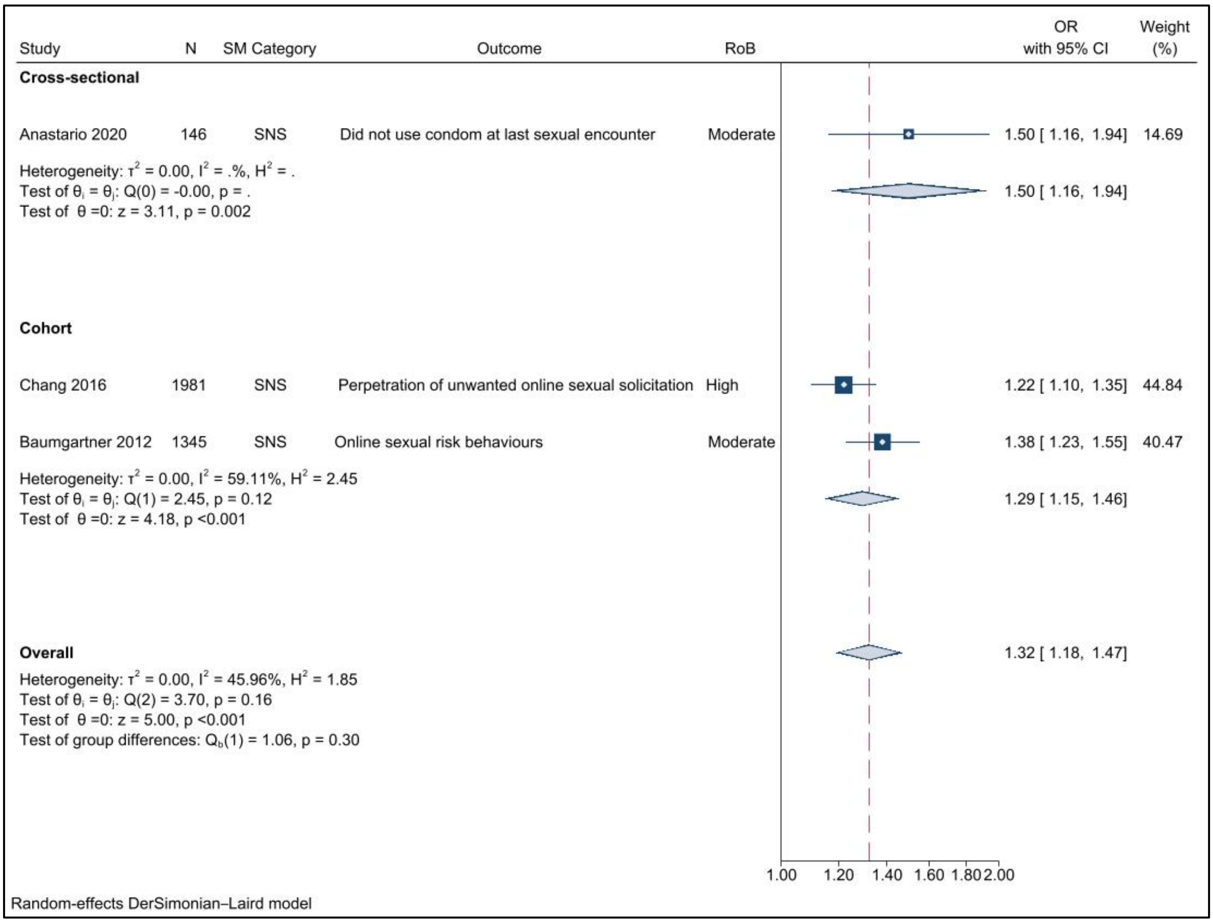

Legend: Figure presents forest plot for continuous exposure & binary outcome sensitivity analysis, with odds ratio (OR) used as common metric. Total number of study participants = 3,472. Abbreviations: CI = Confidence interval; N = Number of study participants; OR = Odds ratio; RoB = Risk of bias; SM = Social media; and SNS = Social networking sites.

Figure CE. Forest plot for association between frequency of social media use and gambling, by study design

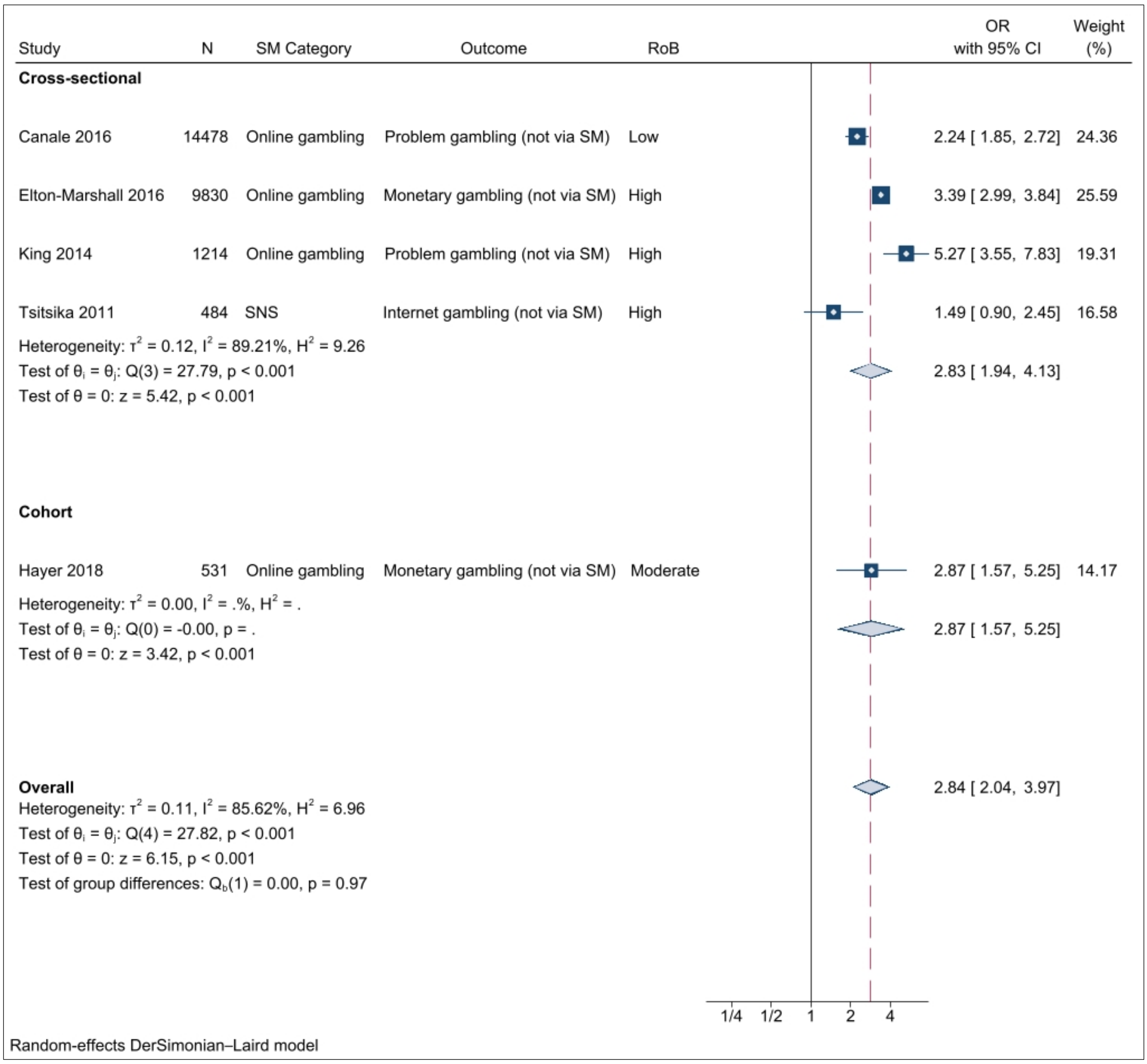

Legend: Figure presents forest plot for binary exposure (frequent/at all vs infrequent/not at all) & binary/continuous outcome sensitivity analysis, with odds ratio (OR) used as common metric. Total number of study participants = 26,537. Abbreviations: CI = Confidence interval; N = Number of study participants; OR = Odds ratio; RoB = Risk of bias; SM = Social media; and SNS = Social networking sites.

Figure CF. Forest plot for association between frequency of social media use and anti-social behaviour, by study design

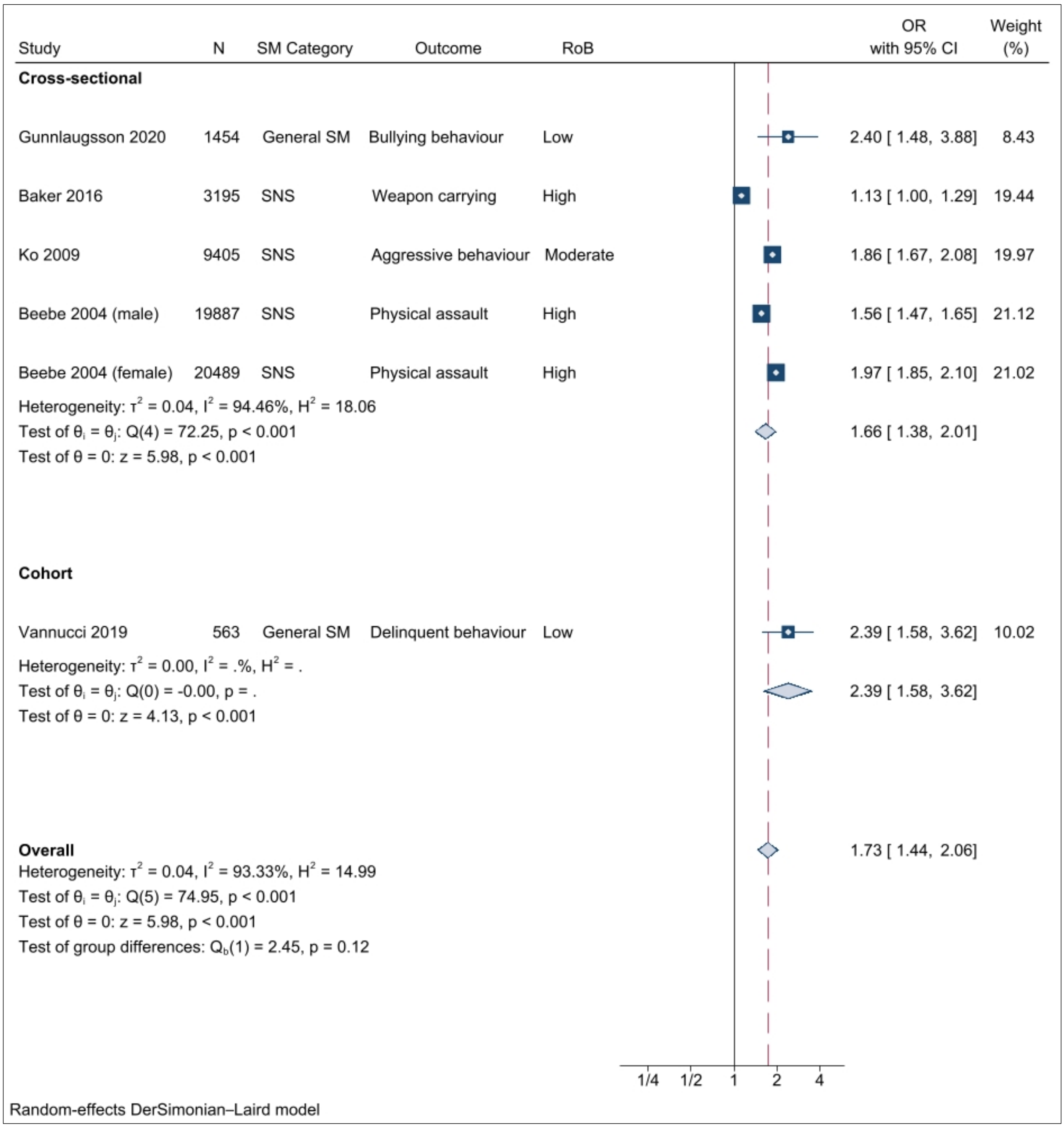

Legend: Figure presents forest plot for binary exposure (frequent/at all vs infrequent/not at all) & binary/continuous outcome sensitivity analysis, with odds (OR) used as common metric. Total number of study participants = 54,993. Abbreviations: CI = Confidence interval; N = Number of study participants; OR = Odds ratio; RoB = Risk of bias; SM = Social media; and SNS = Social networking sites

Figure CG. Forest plot for association between exposure to health-risk behaviour content on social media and unhealthy dietary behaviour, by study design

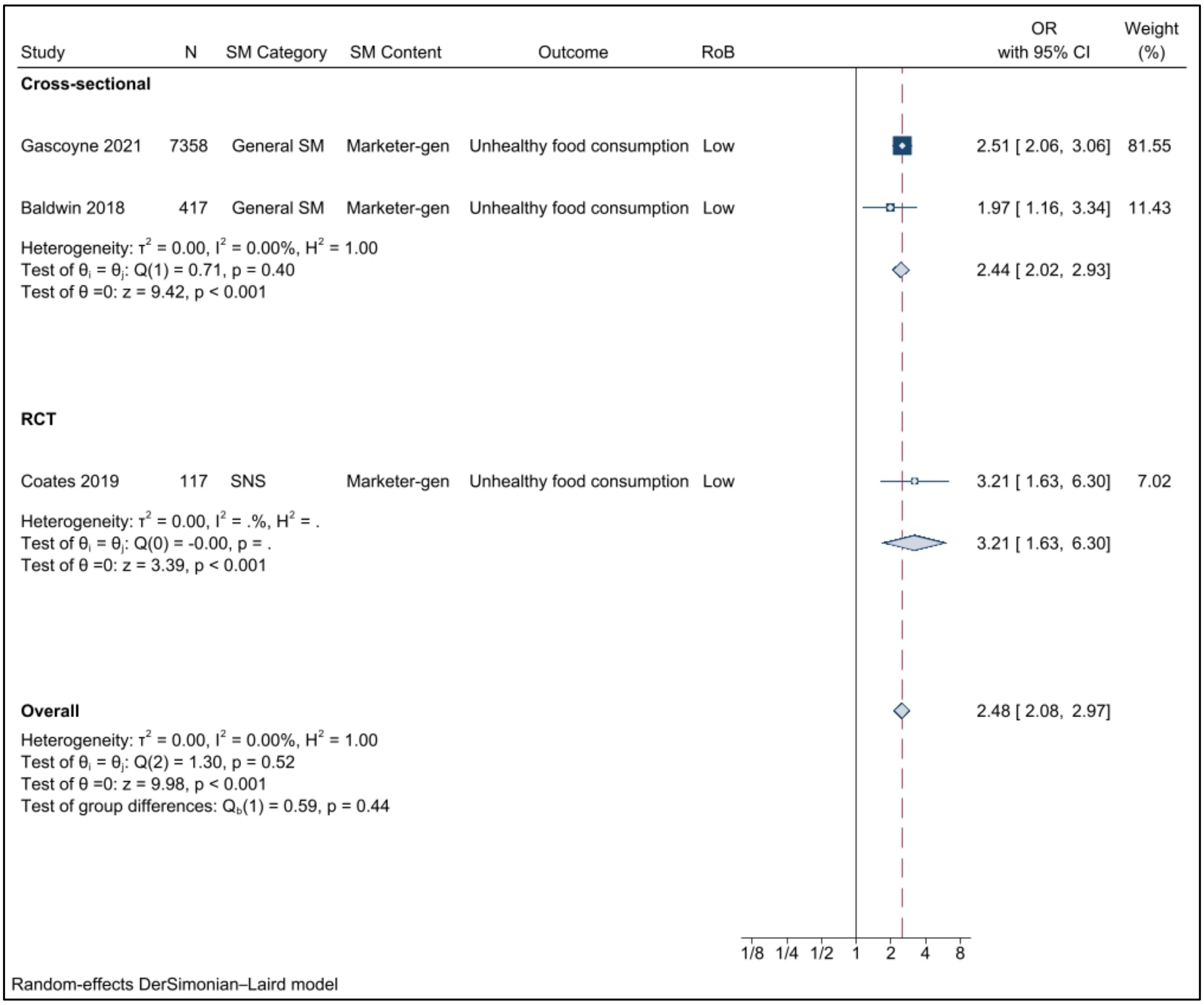

Legend: Figure presents forest plot for binary exposure (exposed vs unexposed) & binary/continuous outcome sensitivity analysis, with odds ratio (OR) used as common metric. Total number of study participants = 7,892. Abbreviations: CI = Confidence interval; N = Number of study participants; OR = Odds ratio; RoB = Risk of bias; SM = Social media; and SNS = Social networking sites.

Figure CH. Forest plot for association between frequency of social media use and alcohol use, by adjustment for critical confounding domains[a]

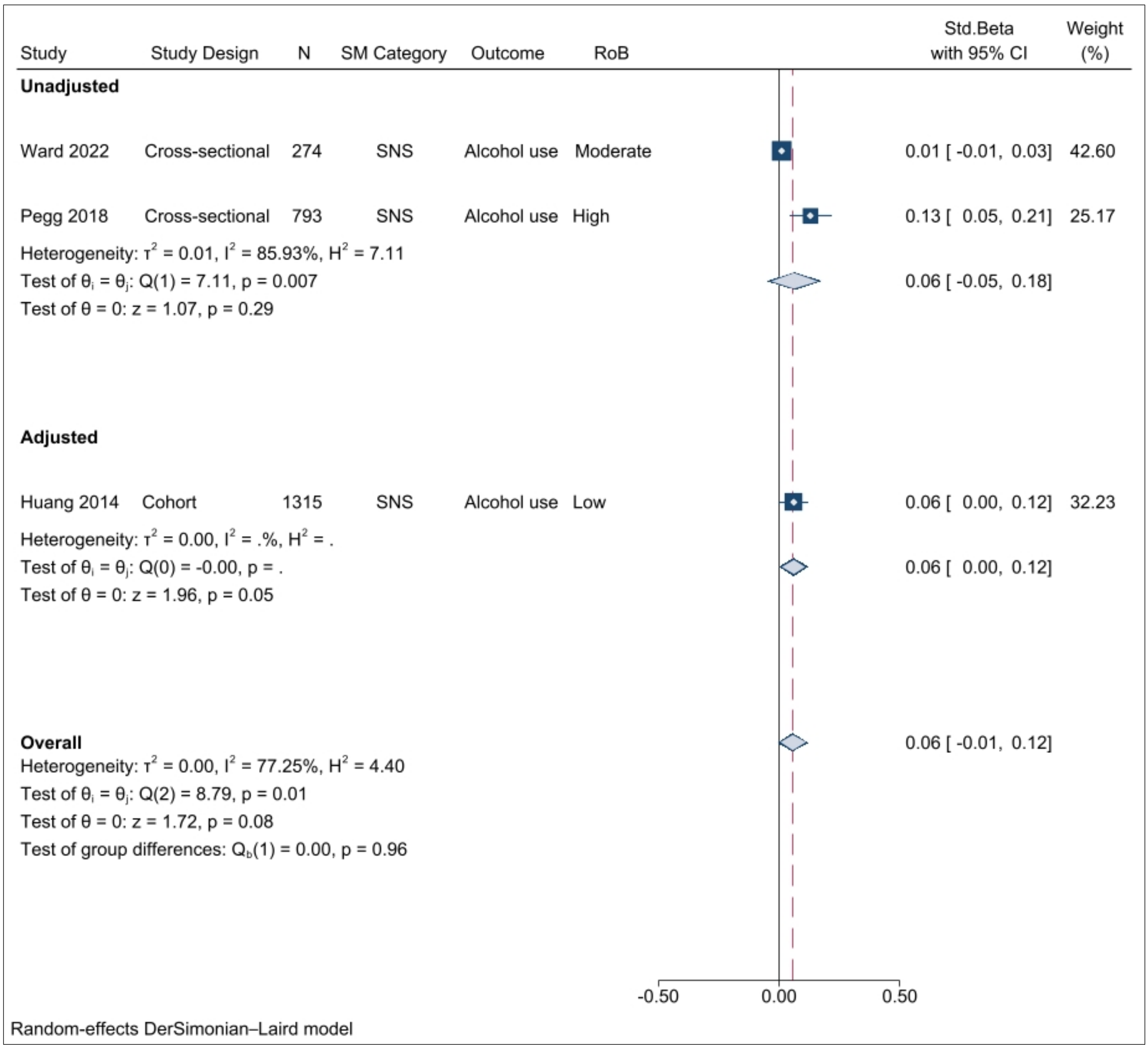

Legend: Figure presents forest plot for continuous exposure & continuous outcome sensitivity analysis, with standardised beta (Std.Beta) used as common metric. [a] Critical confounding domains: age, sex, and socioeconomic position (SEP). Total number of study participants = 2,382. Abbreviation: CI = Confidence interval; N = Number of study participants; RoB = Risk of bias; SM = Social media; SNS = Social networking sites; and Std. Beta = Standardised beta.

Figure CI. Forest plot for association between time spent on social media and alcohol use, by adjustment for critical confounding domains[a]

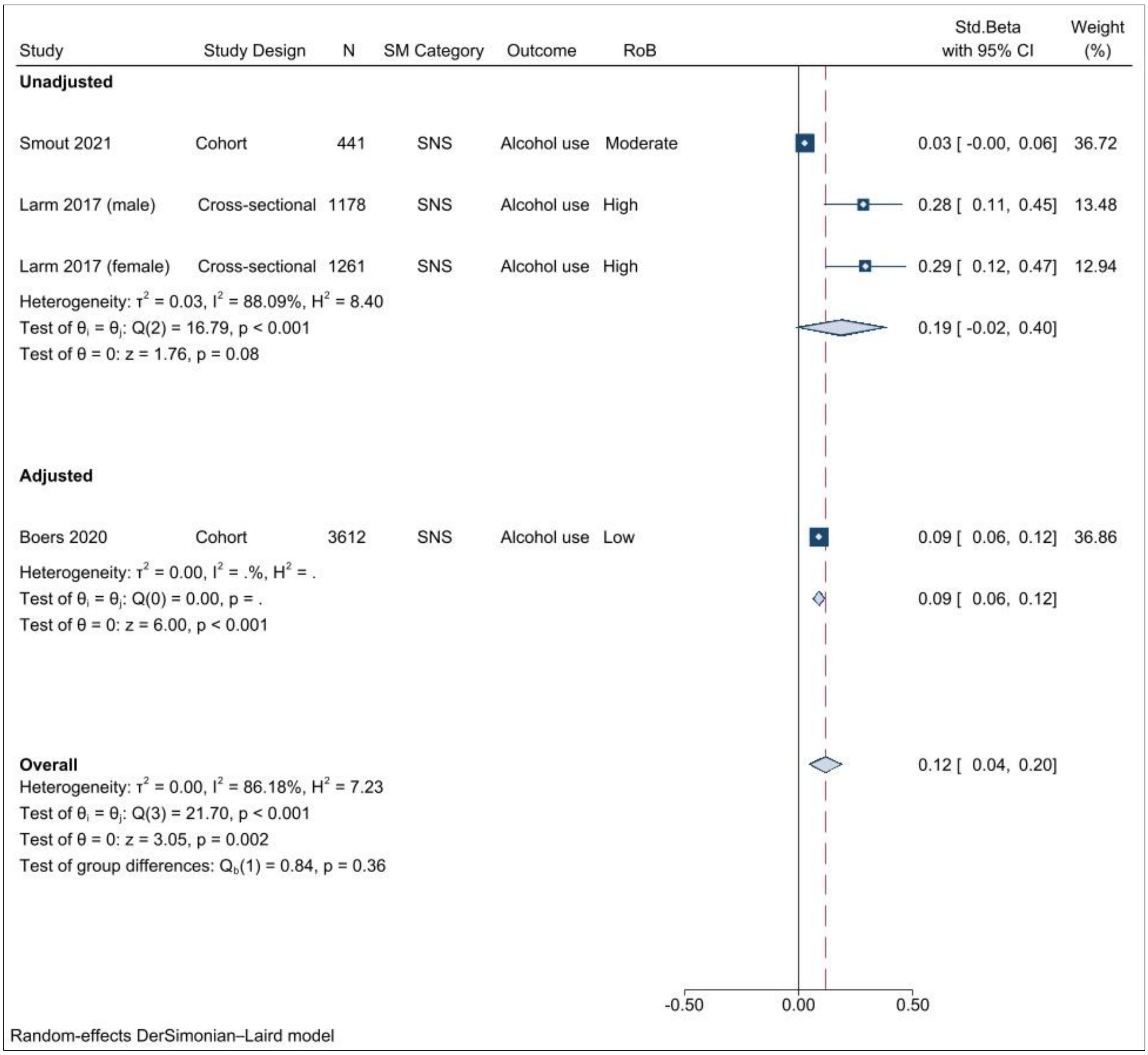

Legend: Figure presents forest plot for continuous exposure & continuous outcome sensitivity analysis, with standardised beta (Std. Beta) used as common metric. [a] Critical confounding domains: age, sex, and socioeconomic position (SEP). Total number of study participants = 6,492. Abbreviation: CI = Confidence interval; N = Number of study participants; RoB = Risk of bias; SM = Social media; SNS = Social networking sites; and Std. Beta = Standardised beta.

Figure CJ. Forest plot for association between exposure to health-risk behaviour content on social media and alcohol use, by adjustment for critical confounding domains[a]

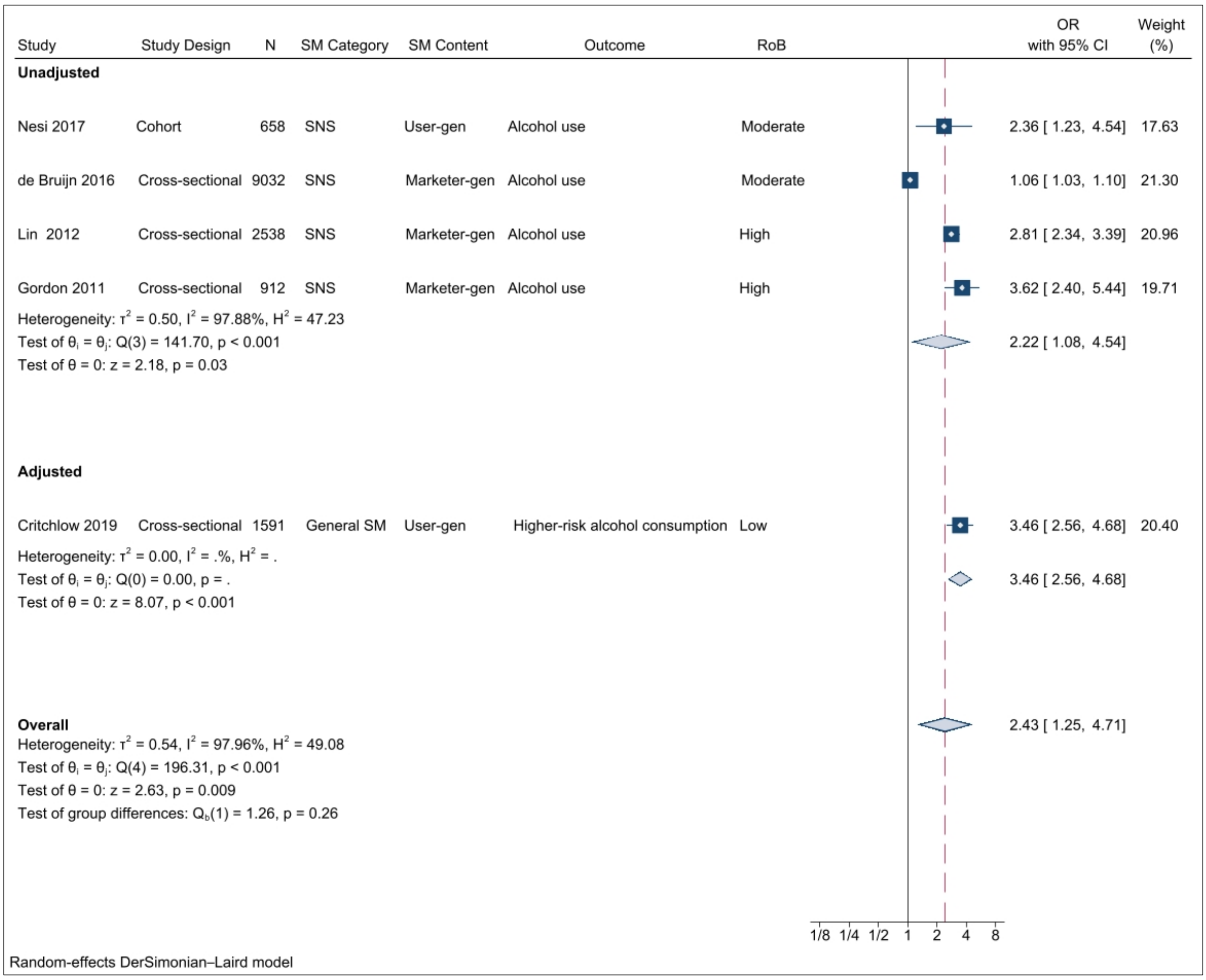

Legend: Figure presents forest plot for binary exposure (exposed vs unexposed) & binary/continuous outcome sensitivity analysis, with odds ratio (OR) used as common metric. [a] Critical confounding domains: age, sex, and socioeconomic position (SEP). Total number of study participants = 14,731. Abbreviations: CI = Confidence interval; Marketer-gen = Marketer-generated content; N = Number of study participants; OR = Odds ratio; RoB = Risk of bias; SM = Social media; SNS = Social networking sites; and User-gen = User-generated content.

Figure CK. Forest plot for association between frequency of social media use and drug use, by adjustment for critical confounding domains[a]

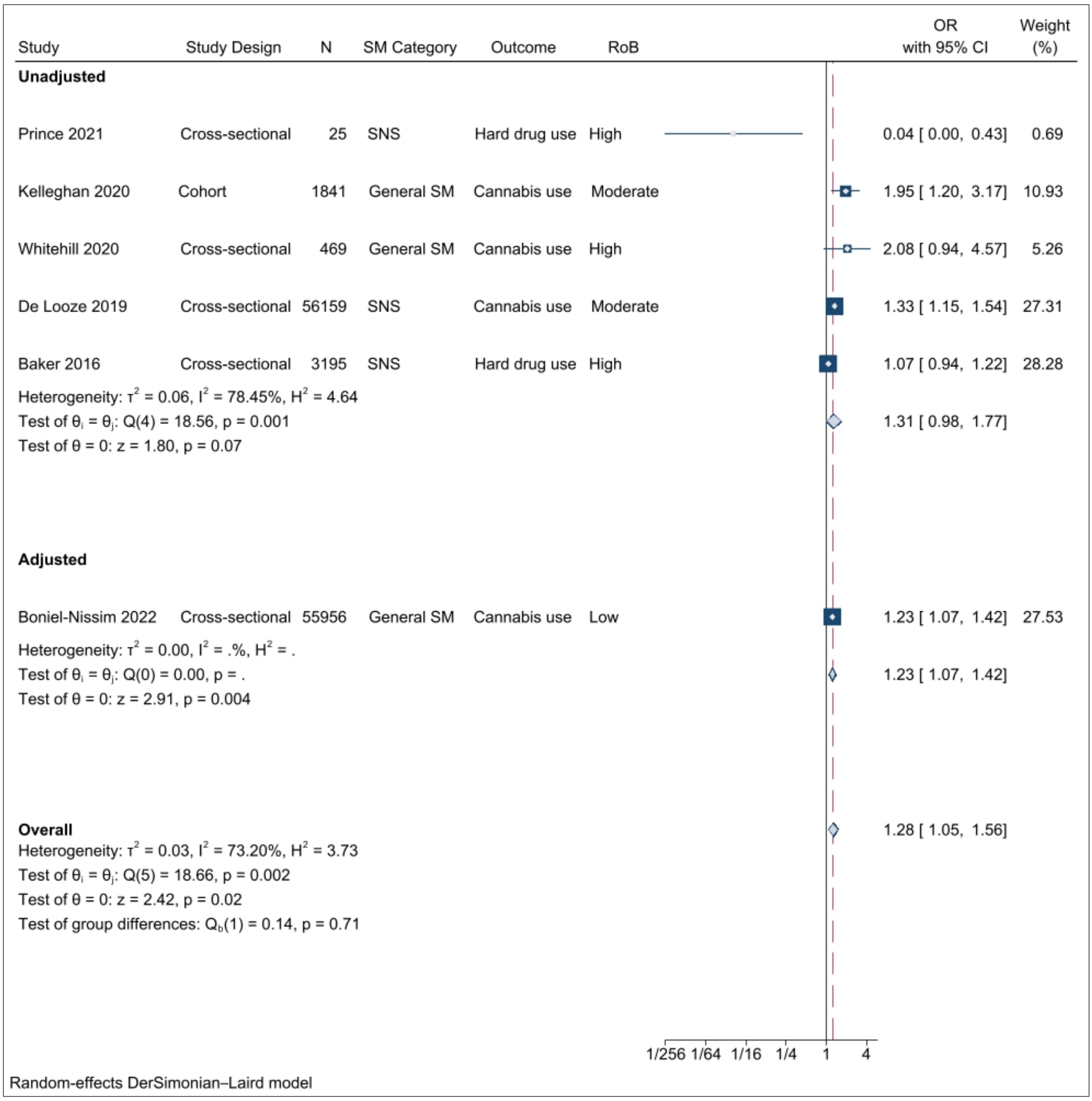

Legend: Figure presents forest plot for binary exposure (frequent/daily vs infrequent/non-daily) & binary/continuous outcome sensitivity analysis, with odds ratio (OR) used as common metric. [a] Critical confounding domains: age, sex, and socioeconomic position (SEP). Total number of study participants = 117,645. Abbreviations: CI = Confidence interval; N = Number of study participants; OR = Odds ratio; RoB = Risk of bias; SM = Social media; and SNS = Social networking sites.

Figure CL. Forest plot for association between frequency of social media use and tobacco use, by adjustment for critical confounding domains[a]

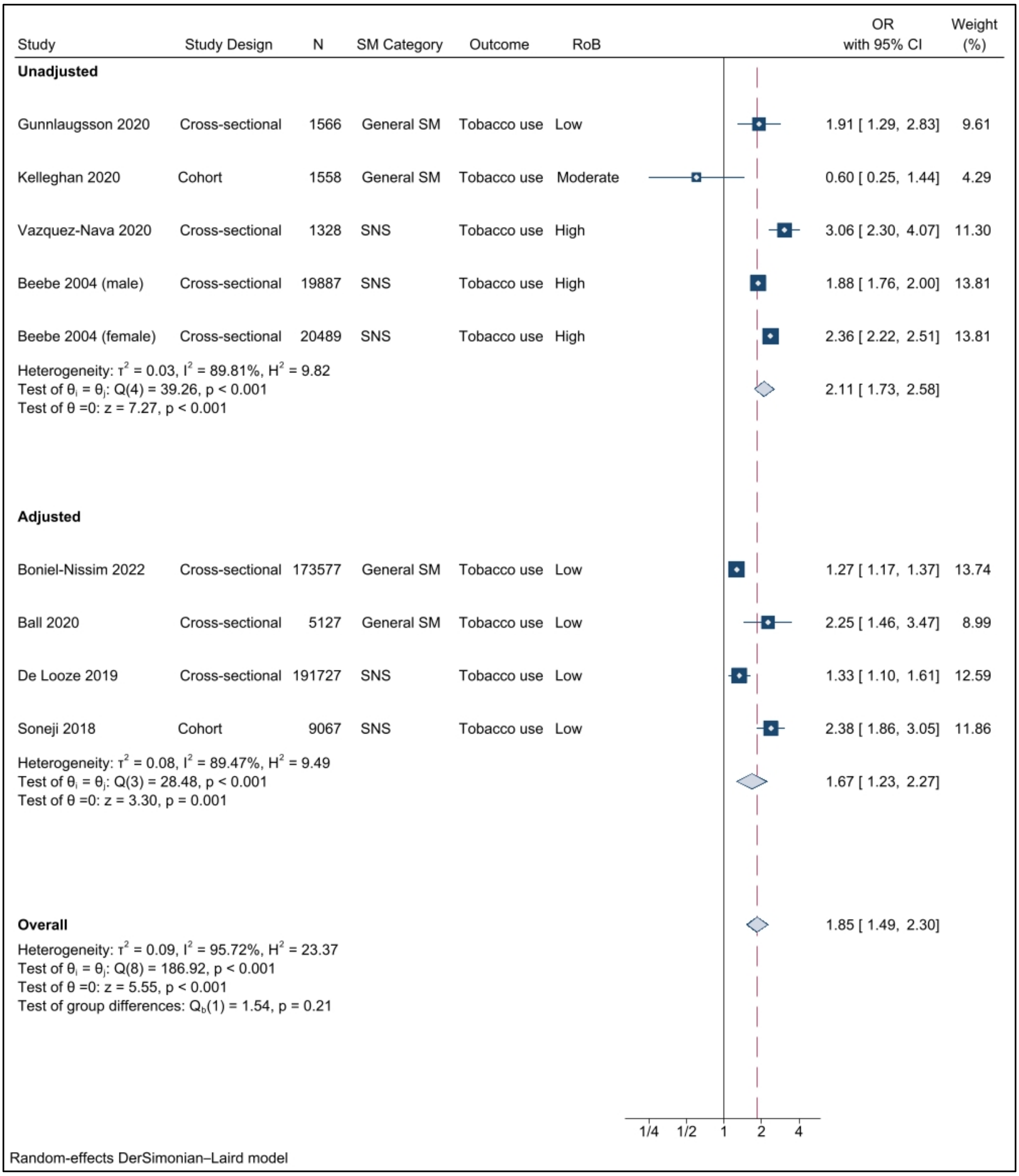

Legend: Figure presents forest plot for binary exposure (frequent vs infrequent) & binary/continuous outcome sensitivity analysis, with odds ratio (OR) used as common metric. [a] Critical confounding domains: age, sex, and socioeconomic position (SEP)**.** Total number of study participants = 424,326. Abbreviations: CI = Confidence interval; N = Number of study participants; OR = Odds ratio; RoB = Risk of bias; SM = Social media; and SNS = Social networking site

Figure CM. Forest plot for association between exposure to health-risk behaviour content on social media and tobacco use, by adjustment for critical confounding domains[a]

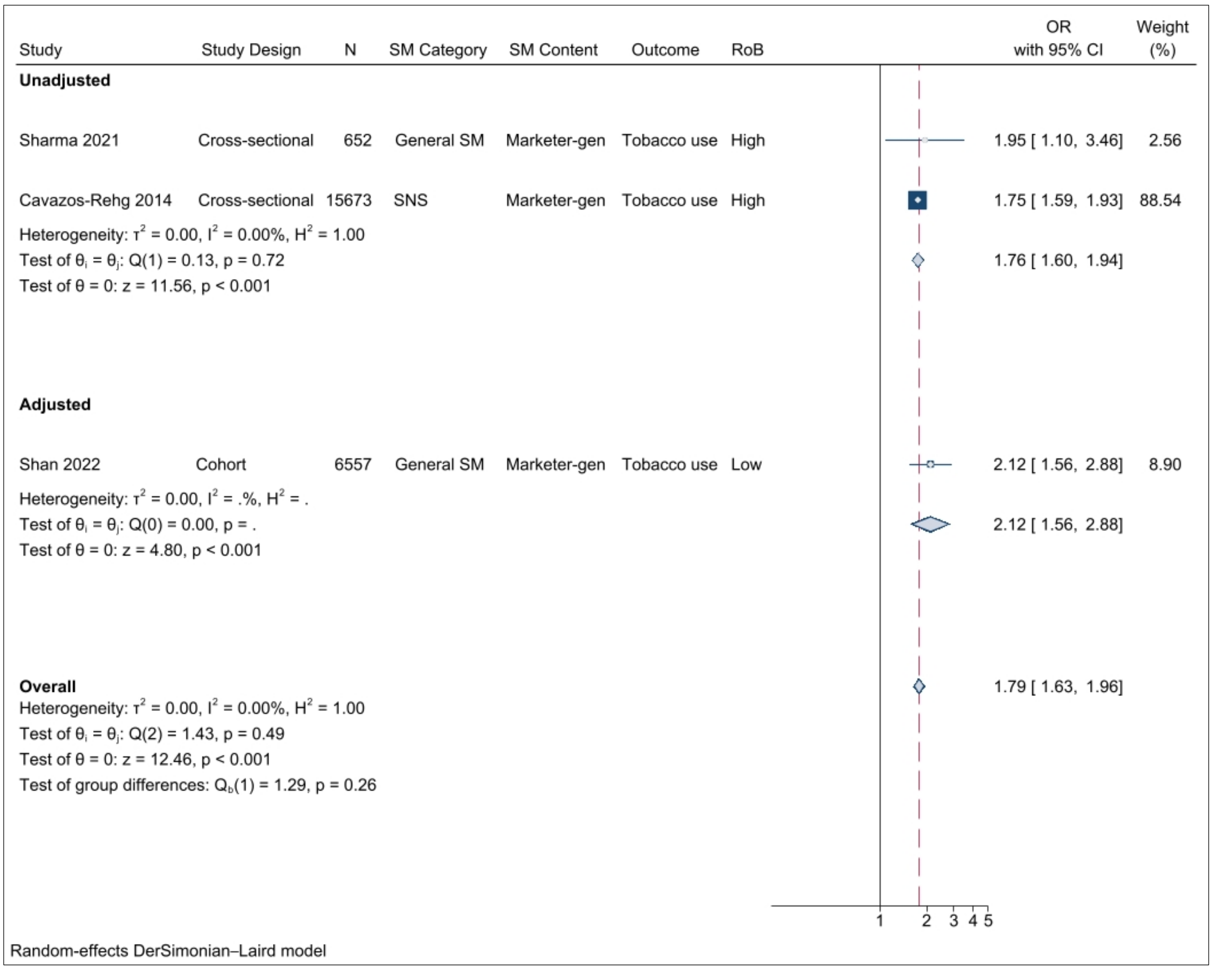

Legend: Figure presents forest plot for binary exposure (exposed vs unexposed) & binary/continuous outcome sensitivity analysis, with odds ratio (OR) used as common metric. [a] Critical confounding domains: age, sex, and socioeconomic position (SEP). Total number of study participants = 22,882. Abbreviations: CI = Confidence interval; Marketer-gen = Marketer-generated content; N = Number of study participants; OR = Odds ratio; RoB = Risk of bias; SM = Social media; and SNS = Social networking sites.

Figure CN. Forest plot for association between exposure to health-risk behaviour content on social media and use of electronic nicotine delivery systems, by adjustment for critical confounding domains[a]

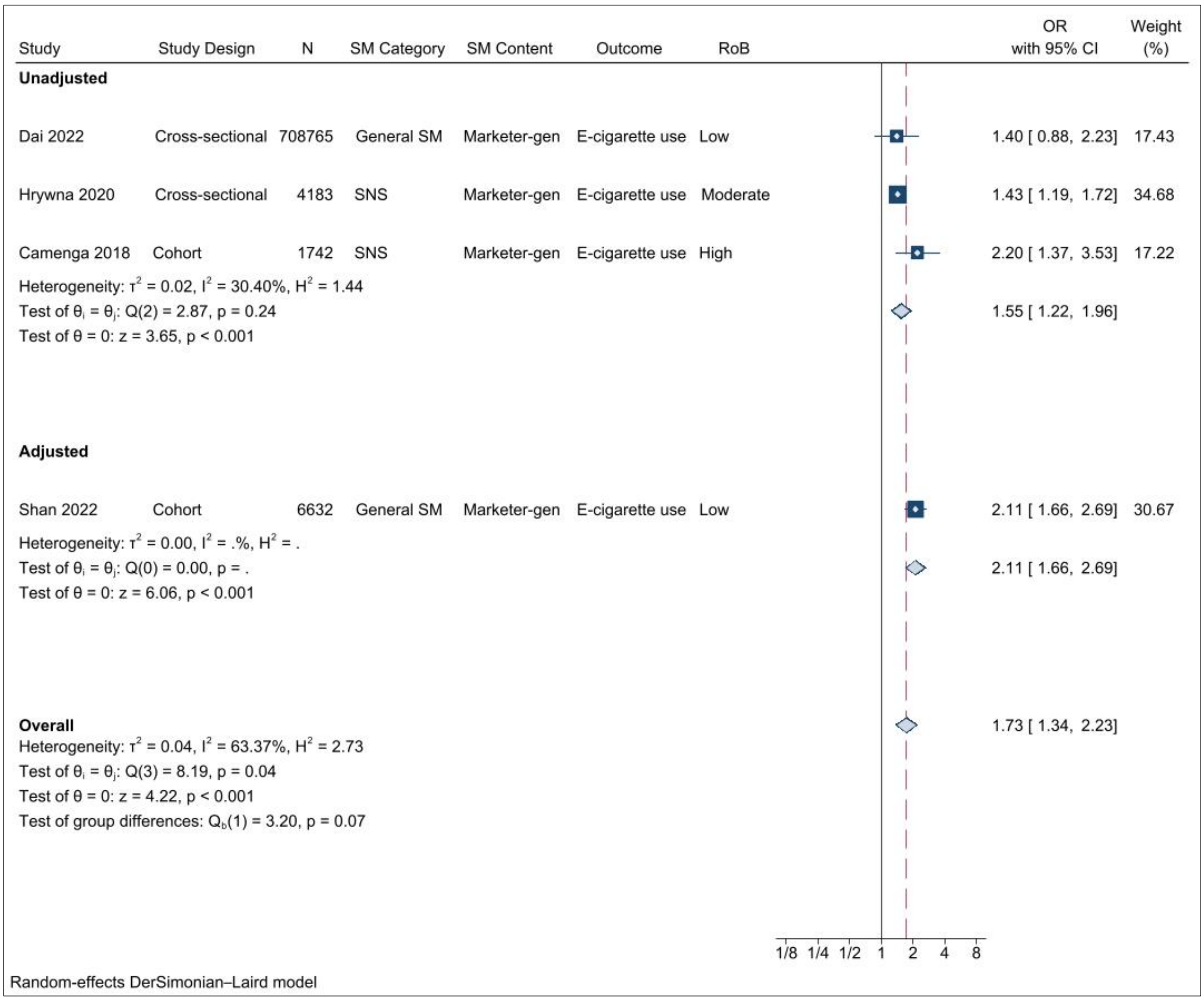

Legend: Figure presents forest plot for binary exposure (exposed vs unexposed) & binary/continuous outcome sensitivity analysis, with odds ratio (OR) used as common metric. [a] Critical confounding domains: age, sex, and socioeconomic position (SEP). Total number of study participants = 721,322. Abbreviations: CI = Confidence interval; N = Number of study participants; OR = Odds ratio; RoB = Risk of bias; SM = Social media; and SNS = Social networking sites

Figure CO. Forest plot for association between frequency of social media use and sexual risk behaviour, by adjustment for critical confounding domains[a]

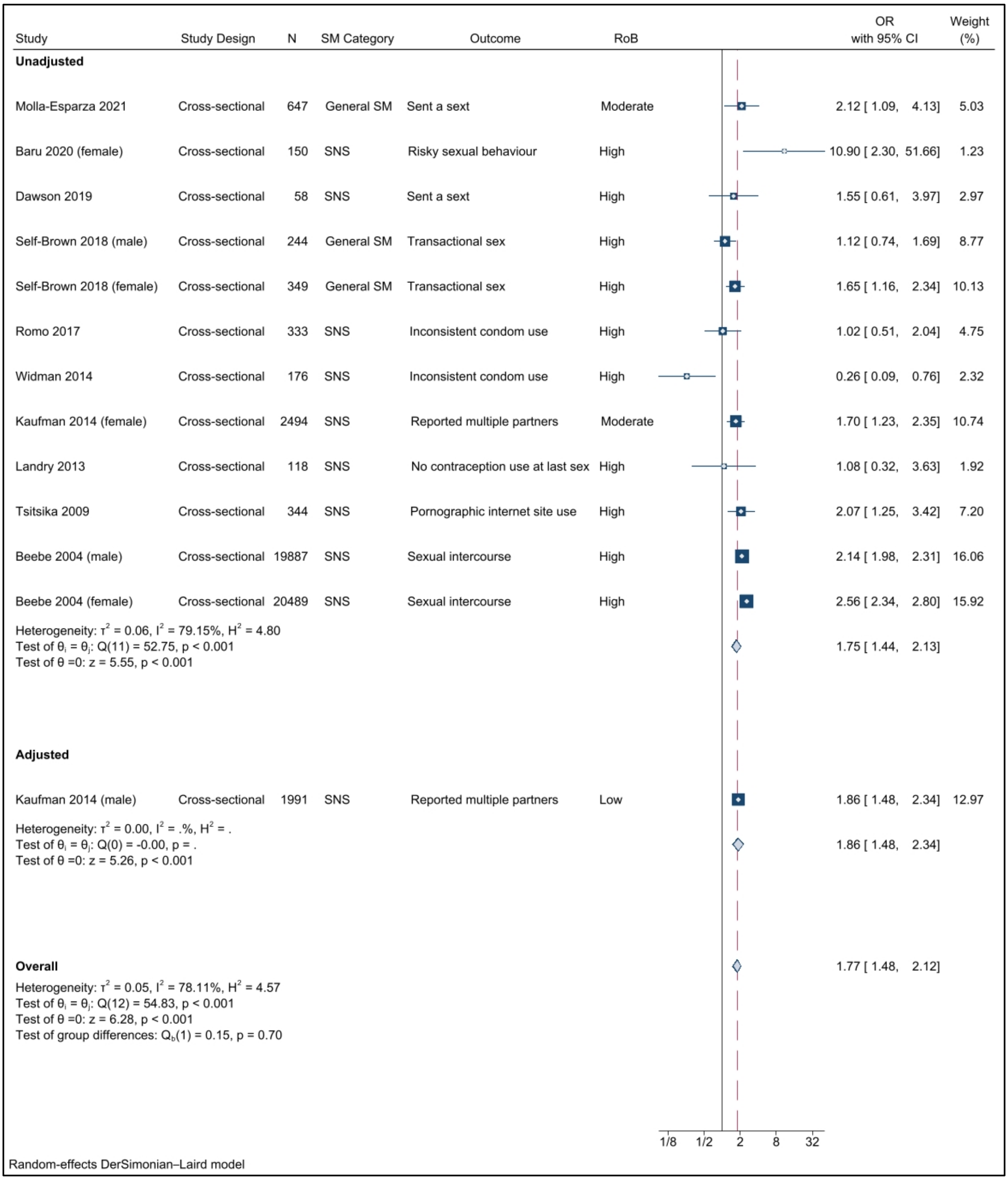

Legend: Figure presents forest plot for binary exposure (frequent/at all vs infrequent/not at all) & binary/continuous outcome sensitivity analysis, with odds ratio (OR) used as common metric. [a] Critical confounding domains: age, sex, and socioeconomic position (SEP). Total number of study participants = 47,280. Abbreviations: CI = Confidence interval; N = Number of study participants; OR = Odds ratio; RoB = Risk of bias; SM = Social media; and SNS = Social networking sites.

Figure CP. Forest plot for association between frequency of social media use and gambling, by adjustment for critical confounding domains[a]

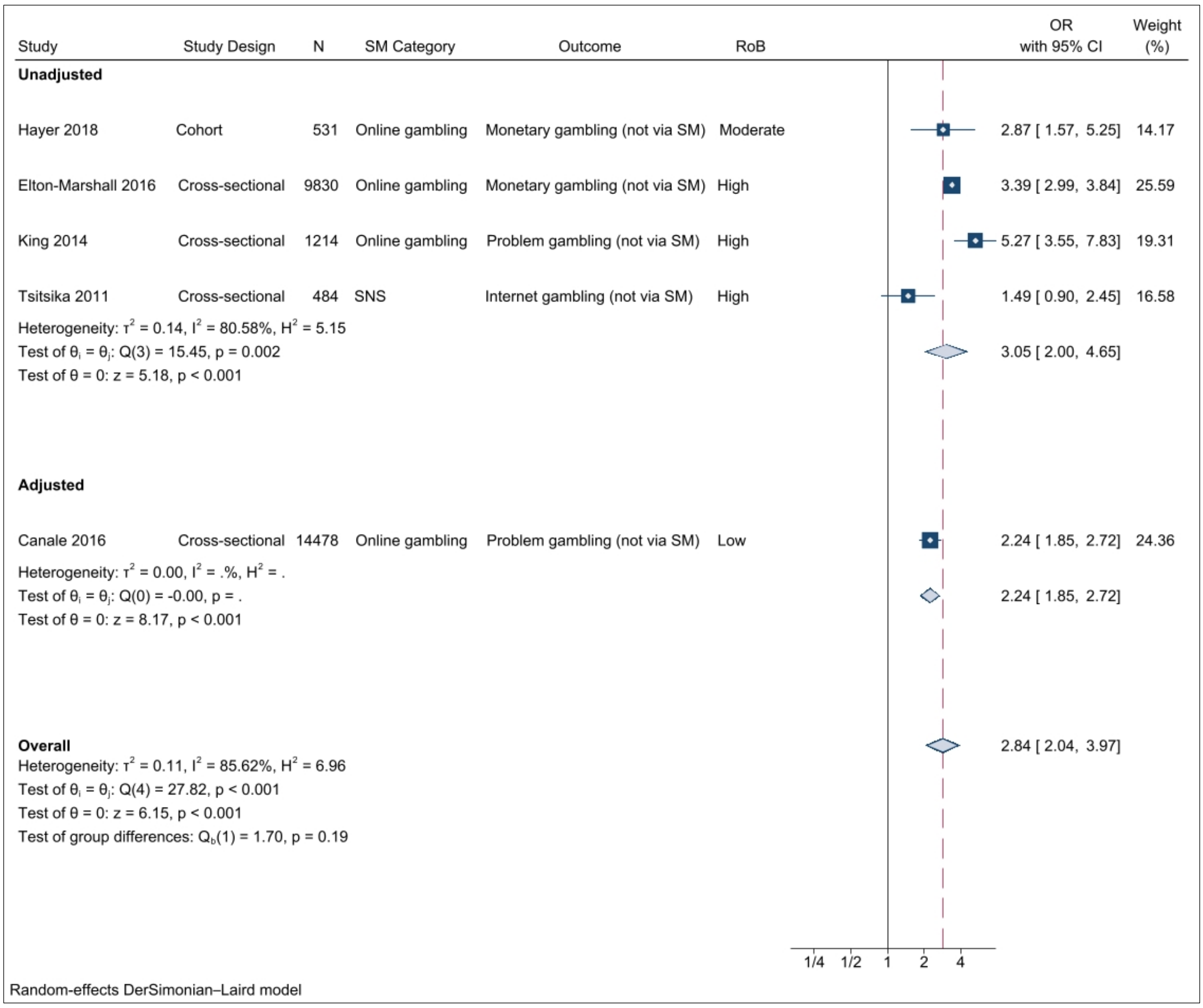

Legend: Figure presents forest plot for binary exposure (frequent/at all vs infrequent/not at all) & binary/continuous outcome sensitivity analysis, with odds ratio (OR) used as common metric. [a] Critical confounding domains: age, sex, and socioeconomic position (SEP). Total number of study participants = 26,537. Abbreviations: CI = Confidence interval; N = Number of study participants; OR = Odds ratio; RoB = Risk of bias; SM = Social media; and SNS = Social networking sites

Figure CQ. Forest plot for association between frequency of social media use and anti-social behaviour, by adjustment for critical confounding domains[a]

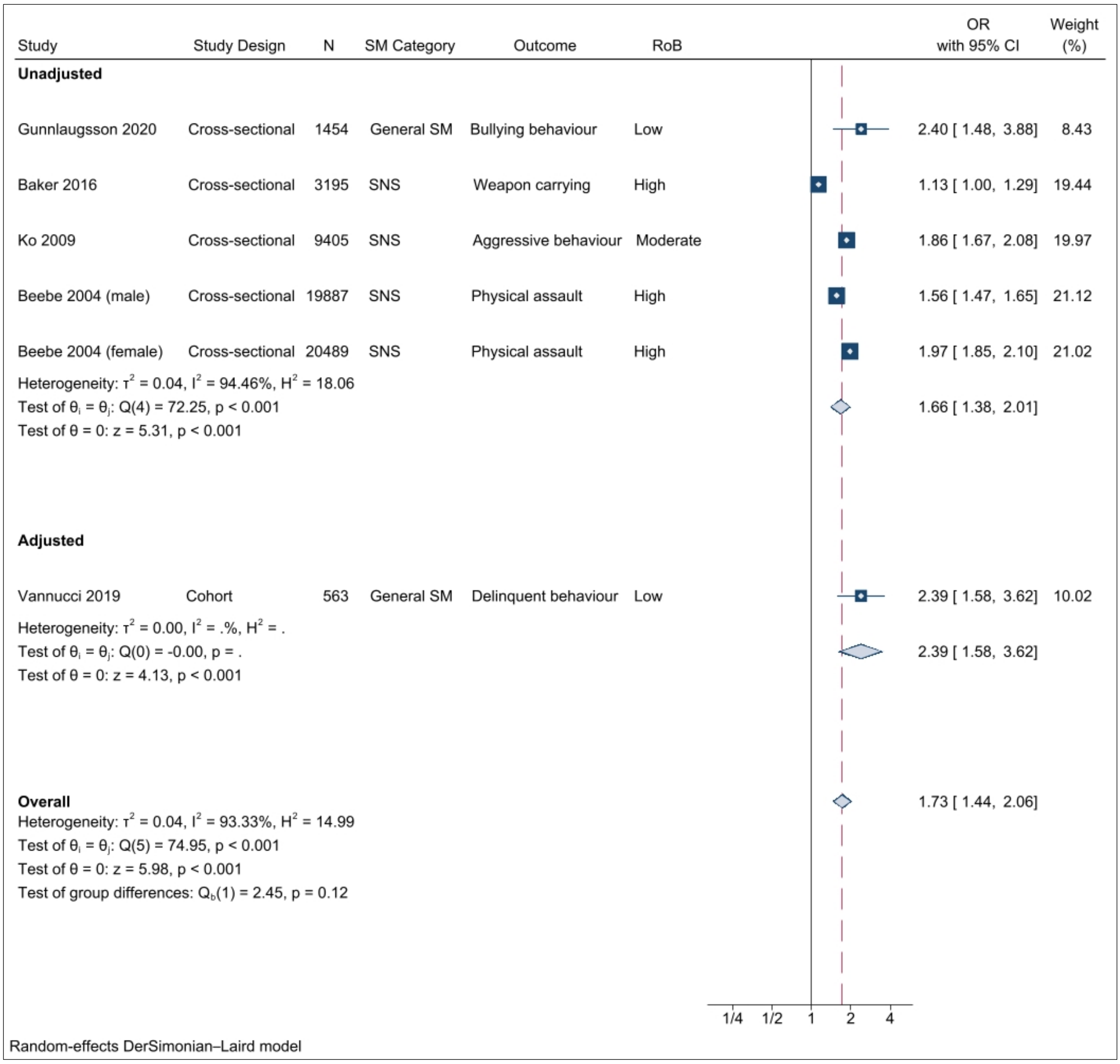

Legend: Figure presents forest plot for binary exposure (frequent/at all vs infrequent/not at all) & binary/continuous outcome sensitivity analysis, with odds (OR) used as common metric. [a] Critical confounding domains: age, sex, and socioeconomic position (SEP). Total number of study participants = 54,993. Abbreviations: CI = Confidence interval; N = Number of study participants; OR = Odds ratio; RoB = Risk of bias; SM = Social media; and SNS = Social networking sites

Figure CR. Forest plot for association between time spent on social media and inadequate physical activity, by adjustment for critical confounding domains[a]

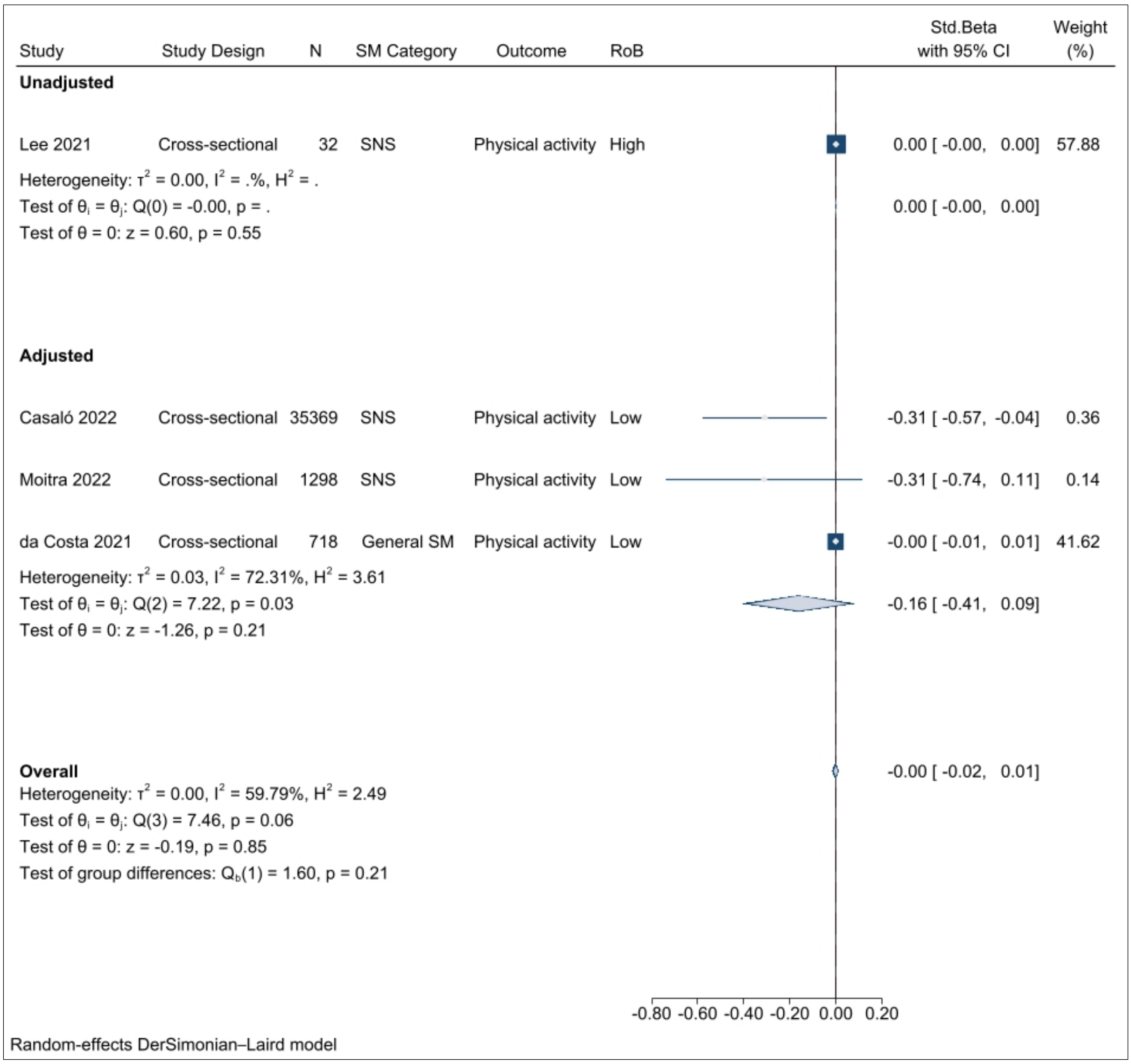

Legend: Figure presents forest plot for continuous exposure & continuous outcome sensitivity analysis, with standardised beta (Std.Beta) used as common metric. [a] Critical confounding domains: age, sex, and socioeconomic position (SEP). Total number of study participants = 37,417. Abbreviations: CI = Confidence interval; N = Number of study participants; RoB = Risk of bias; SM = Social media; SNS = Social networking sites; and Std. Beta = Standardised beta.

Figure CS. Forest plot for association between time spent on social media and alcohol use, by adjustment for critical confounding domains[a]

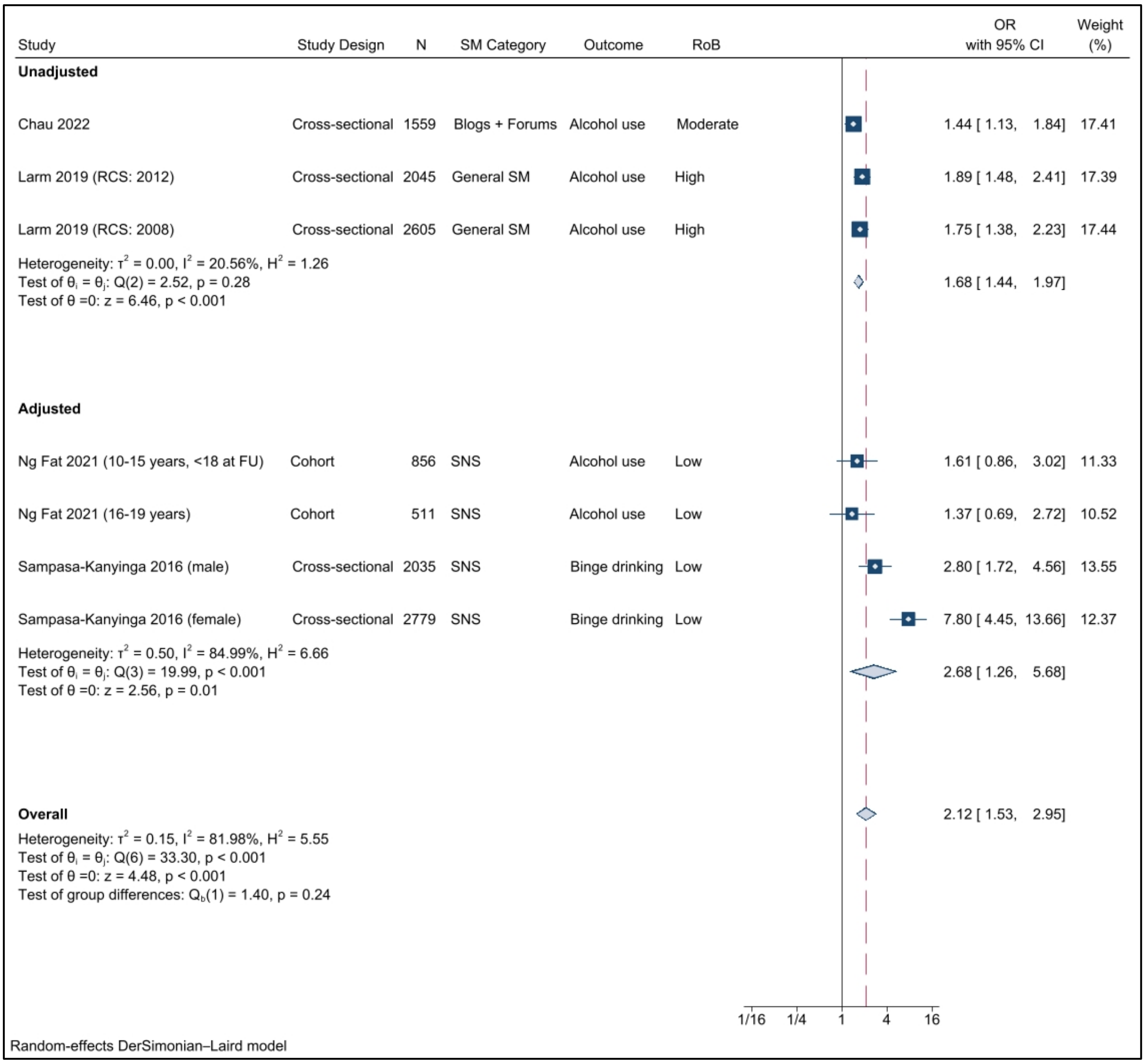

Legend: Figure presents forest plot for binary exposure (≥2 vs <2 hrs/day social media use) & binary/continuous outcome sensitivity analysis, with odds ratio (OR) used as common metric. [a] Critical confounding domains: age, sex, and socioeconomic position (SEP). Total number of study participants = 12,390. Abbreviations: CI = Confidence interval; FU = Follow up; hrs = Hours; N = Number of study participants; OR = Odds ratio; RCS = Repeat cross-sectional study; RoB = Risk of bias; SM = Social media; and SNS = Social networking sites.

Figure CT. Forest plot for association between exposure to health-risk behaviour content on social media and alcohol use, by adjustment for critical confounding domains[a]

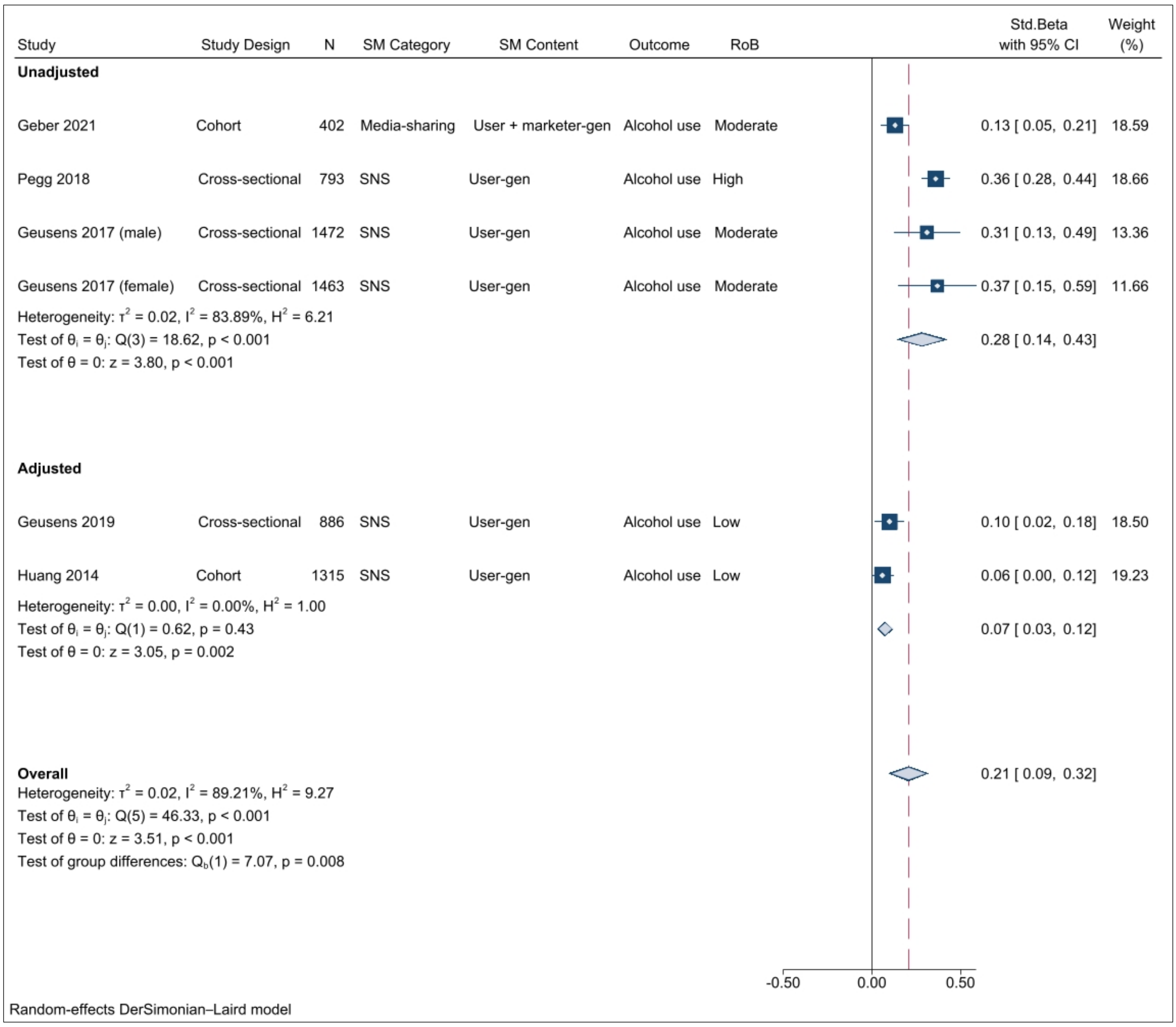

Legend: Figure presents forest plot for continuous exposure & continuous outcome sensitivity analysis, with standardised beta (Std. Beta) used as common metric. [a] Critical confounding domains: age, sex, and socioeconomic position (SEP). Total number of study participants = 6,331. Abbreviation: CI = Confidence interval; N = Number of study participants; Marketer-gen = Marketer-generated content; RoB = Risk of bias; SM = Social media; SNS = Social networking sites; Std. Beta = Standardised beta; and User-gen = User-generated content.

Figure CU. Forest plot for association between frequency of social media use and alcohol use, by adjustment for critical confounding domains[a]

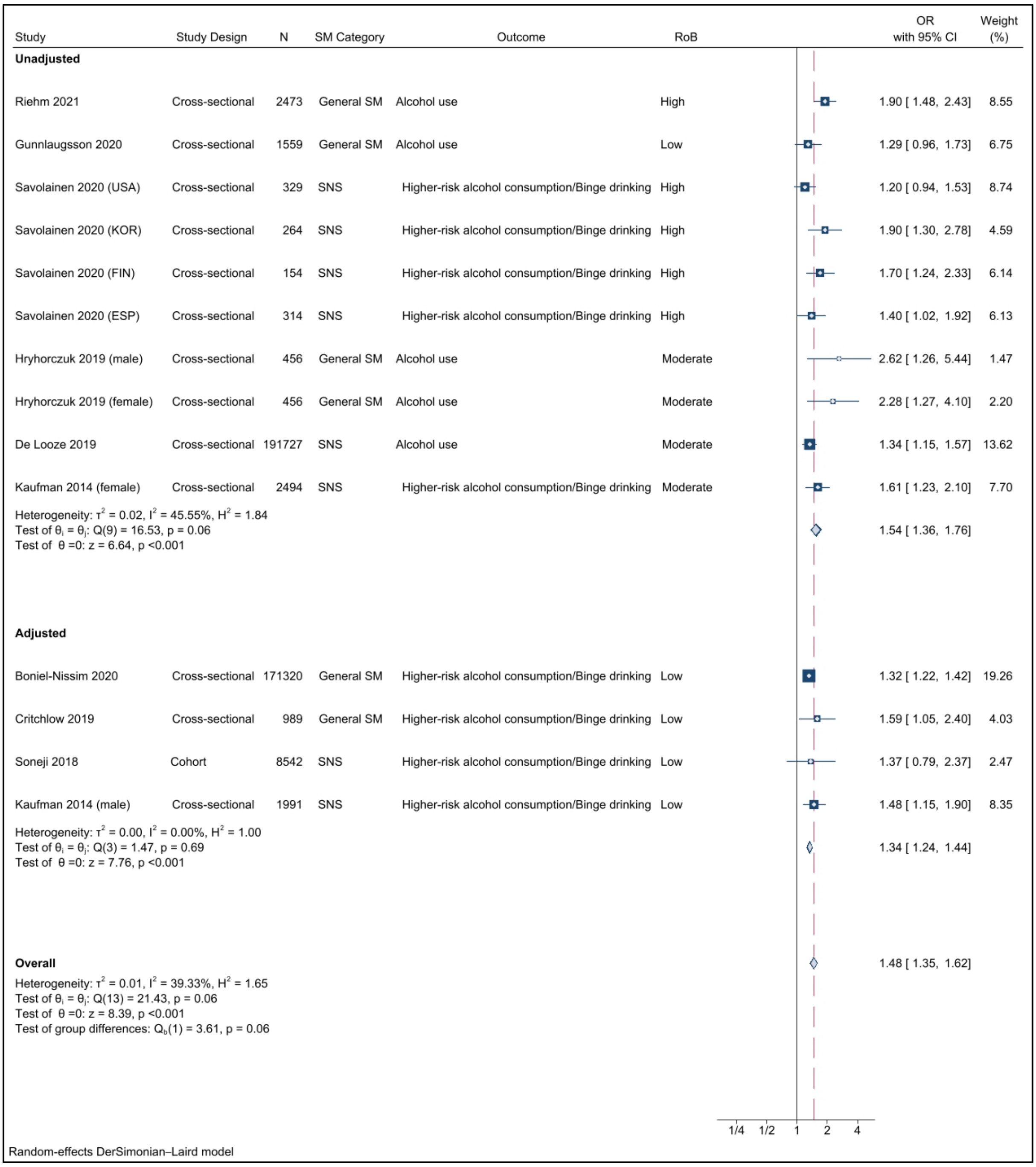

Legend: Figure presents forest plot for binary exposure (frequent/daily vs infrequent/non-daily) & binary/continuous outcome sensitivity analysis, with odds ratio (OR) used as common metric. Total number of study participants = 383,068. Abbreviations: CI = Confidence interval; ESP = Spain; FIN = Finland; KOR = South Korea; N = Number of study participants; OR = Odds ratio; RoB = Risk of bias; SM = Social media; and SNS = Social networking sites.

Figure CV. Forest plot for association between frequency of social media use and alcohol use, by risk of bias grade

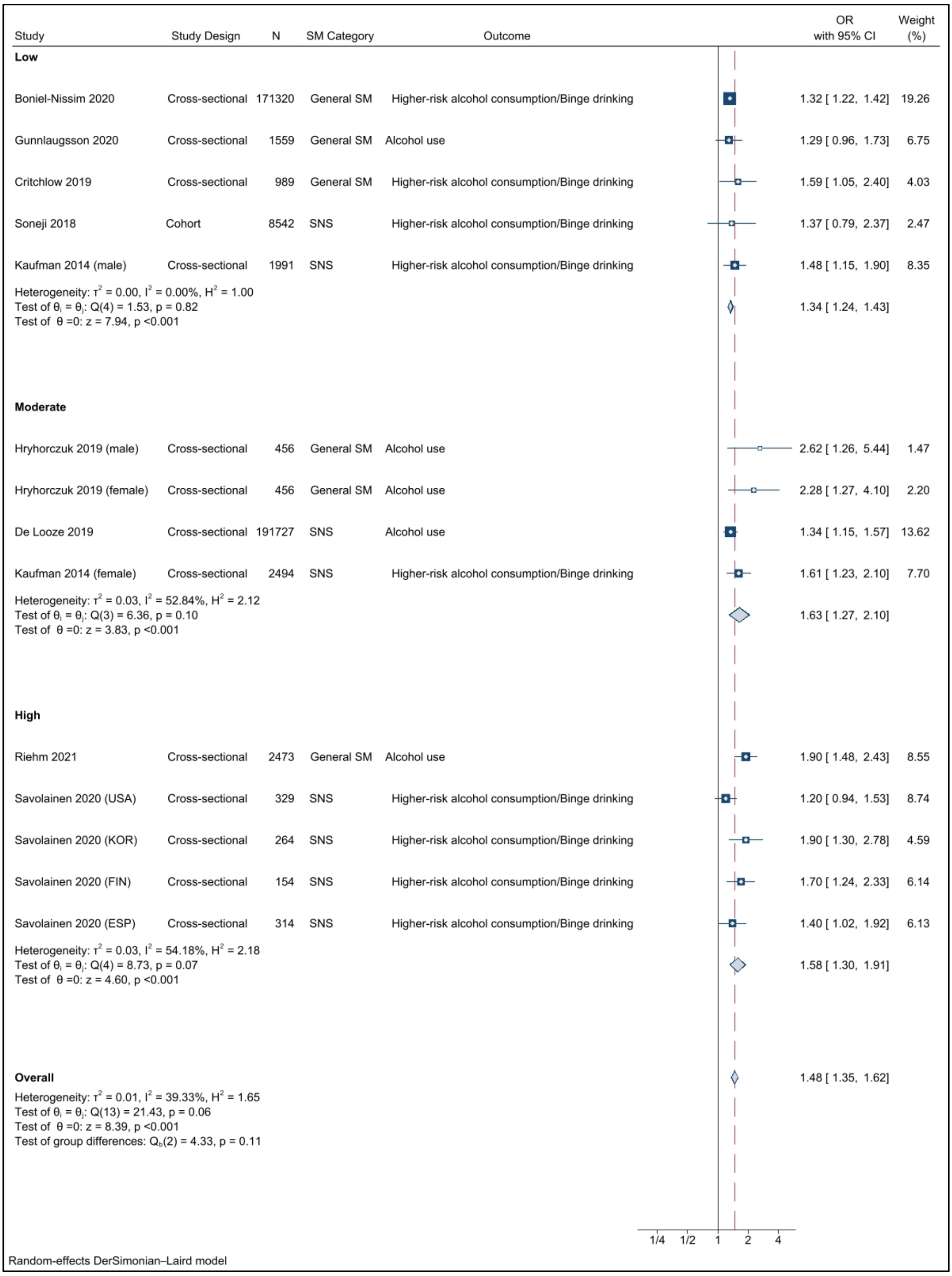

Legend: Figure presents forest plot for binary exposure (frequent/daily vs infrequent/non-daily) & binary/continuous outcome sensitivity analysis, with odds ratio (OR) used as common metric. Total number of study participants = 383,068. Abbreviations: CI = Confidence interval; ESP = Spain; FIN = Finland; KOR = South Korea; N = Number of study participants; OR = Odds ratio; SM = Social media; and SNS = Social networking sites.

Figure CW. Forest plot for association between exposure to health-risk behaviour content on social media and alcohol use, by risk of bias grade

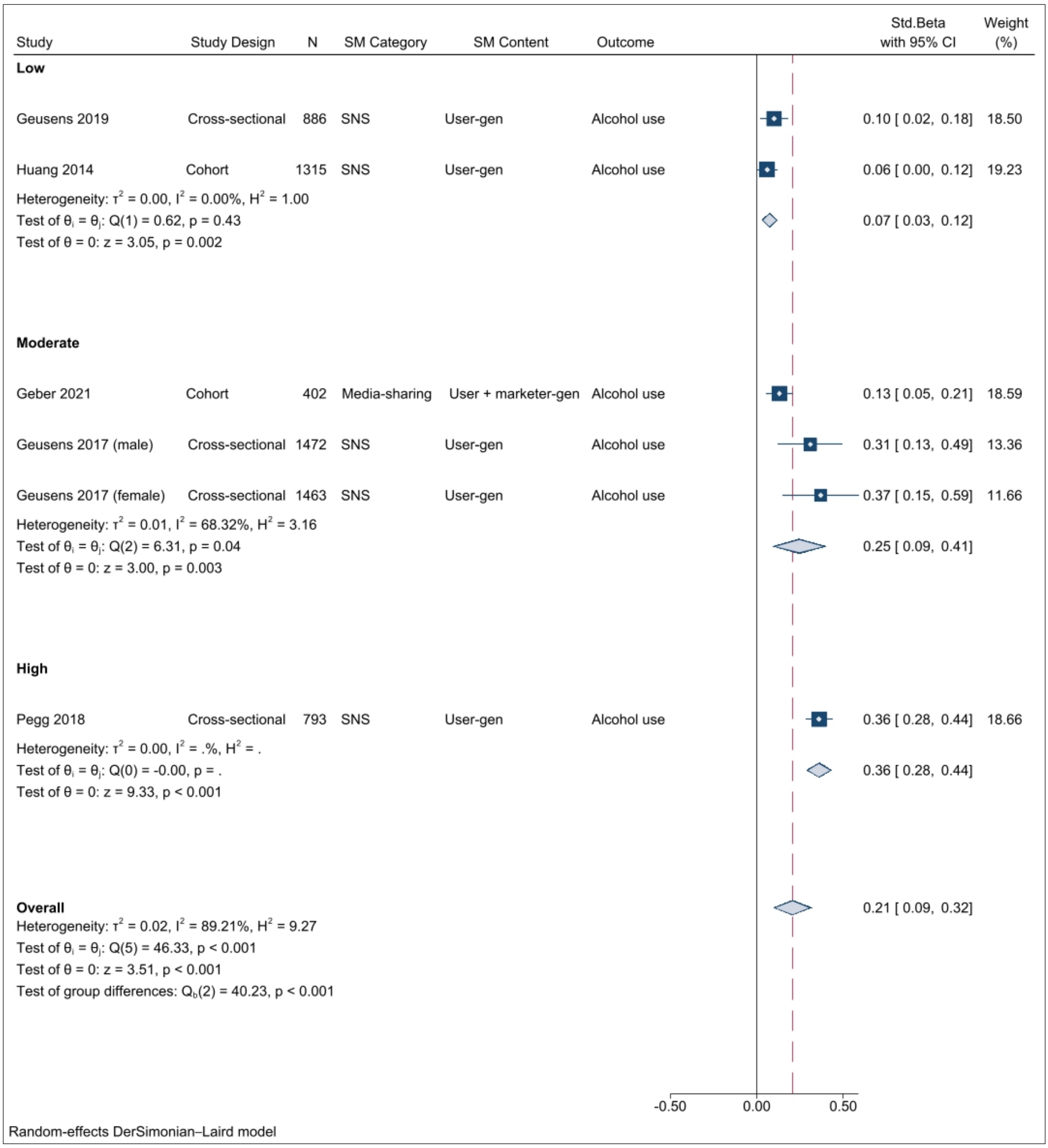

Legend: Figure presents forest plot for continuous exposure & continuous outcome sensitivity analysis, with standardised beta (Std. Beta) used as common metric. Total number of study participants = 6331. Abbreviation: CI = Confidence interval; N = Number of study participants; Marketer-gen = Marketer-generated content; SM = Social media; SNS = Social networking sites; Std. Beta = Standardised beta; and User-gen = User-generated content.

Figure CX. Forest plot for association between time spent on social media and alcohol use, by risk of bias grade

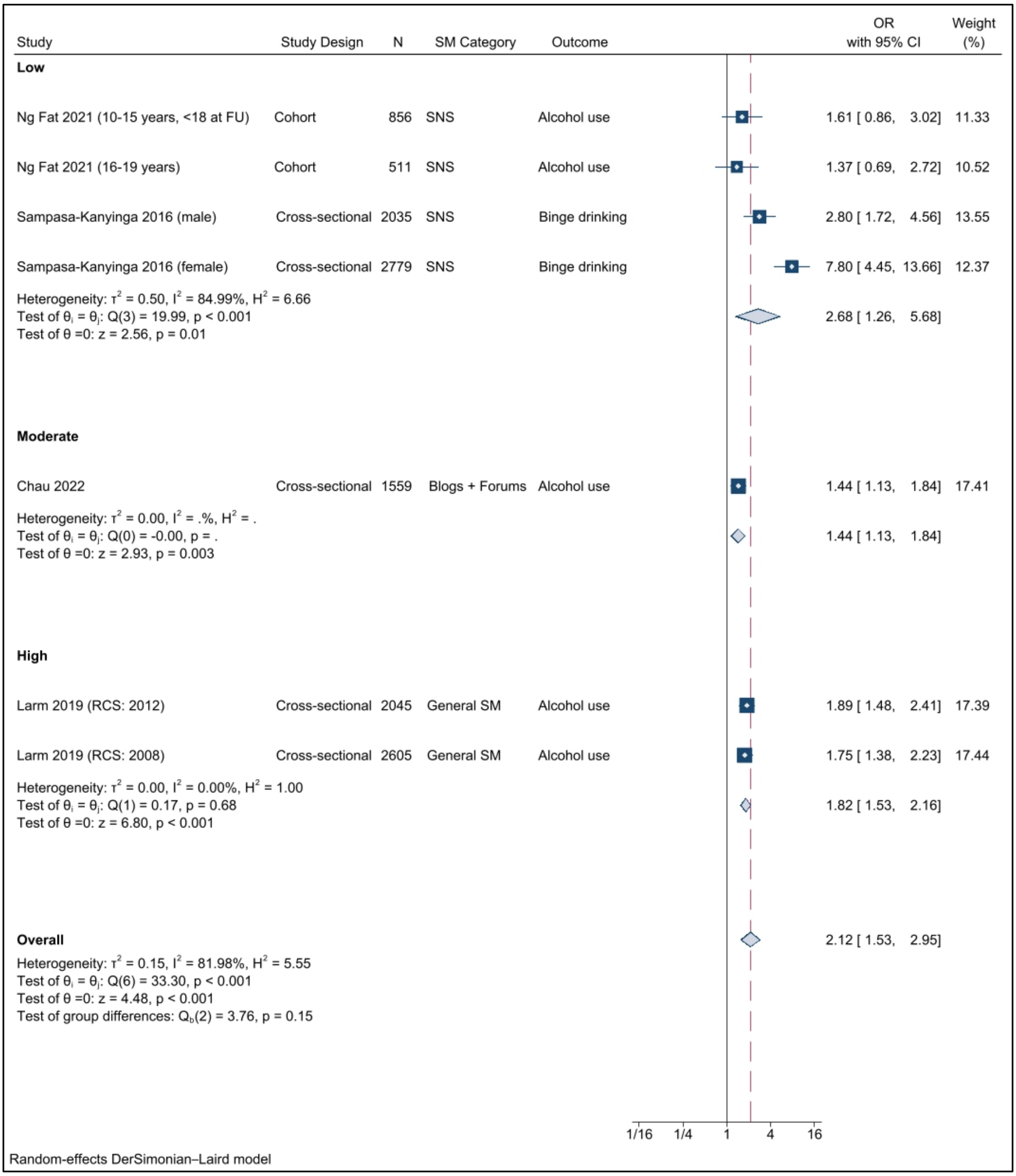

Legend: Figure presents forest plot for binary exposure (≥2 vs <2 hrs/day social media use) & binary/continuous outcome sensitivity analysis, with odds ratio (OR) used as common metric. Total number of study participants = 12,390. Abbreviations: CI = Confidence interval; FU = Follow up; hrs = Hours; N = Number of study participants; OR = Odds ratio; RCS = Repeat cross-sectional study; SM = Social media; and SNS = Social networking sites.

Figure CY. Forest plot for association between exposure to health-risk behaviour content on social media and alcohol use, by risk of bias grade

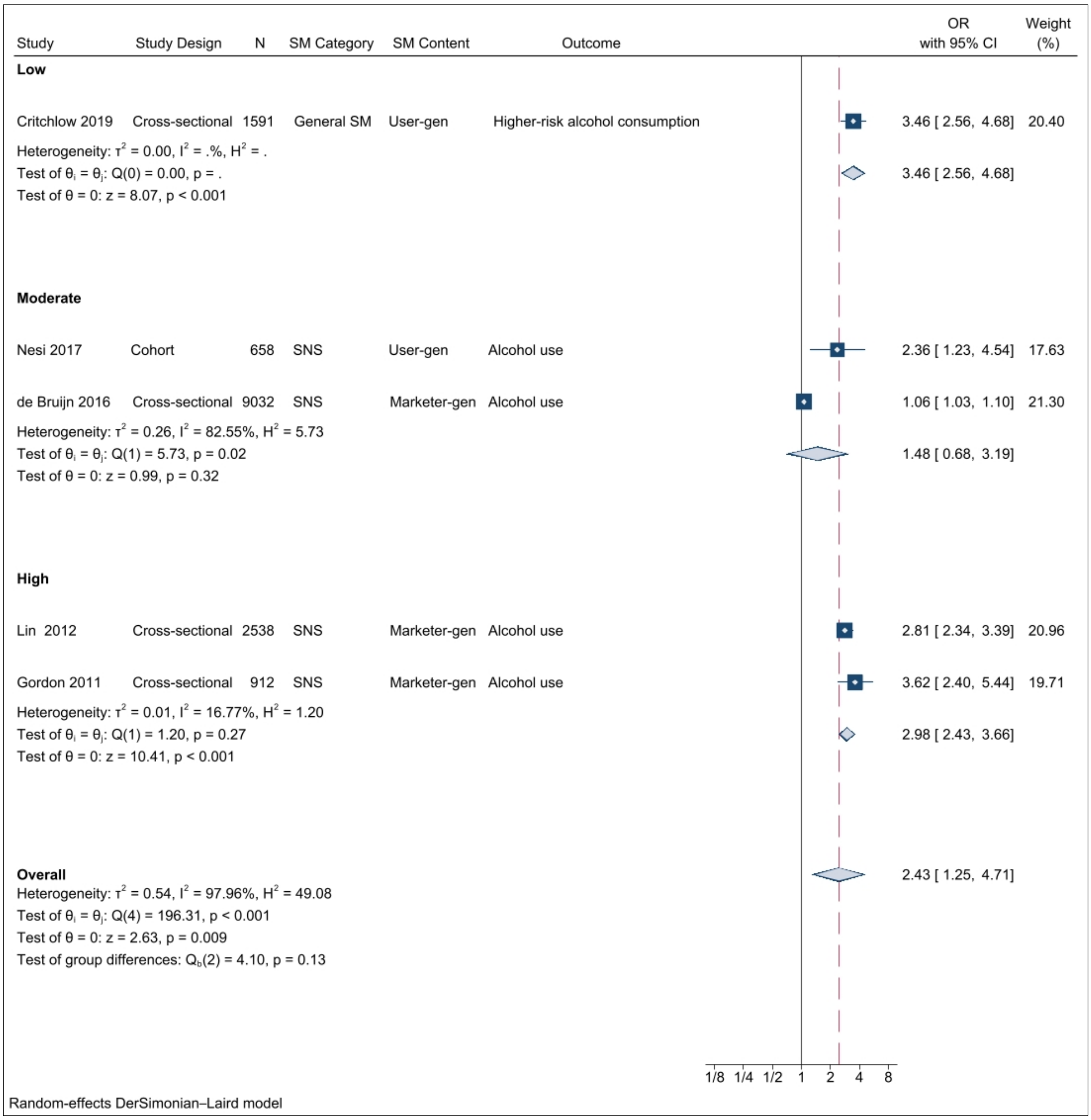

Legend: Figure presents forest plot for binary exposure (exposed vs unexposed) & binary/continuous outcome sensitivity analysis, with odds ratio (OR) used as common metric. [a] Critical confounding domains: age, sex, and socioeconomic position (SEP). Total number of study participants = 14,731. Abbreviations: CI = Confidence interval; Marketer-gen = Marketer-generated content; N = Number of study participants; OR = Odds ratio; SM = Social media; SNS = Social networking sites; and User-gen = User-generated content.

Figure CZ. Forest plot for association between frequency of social media use and drug use, by risk of bias grade

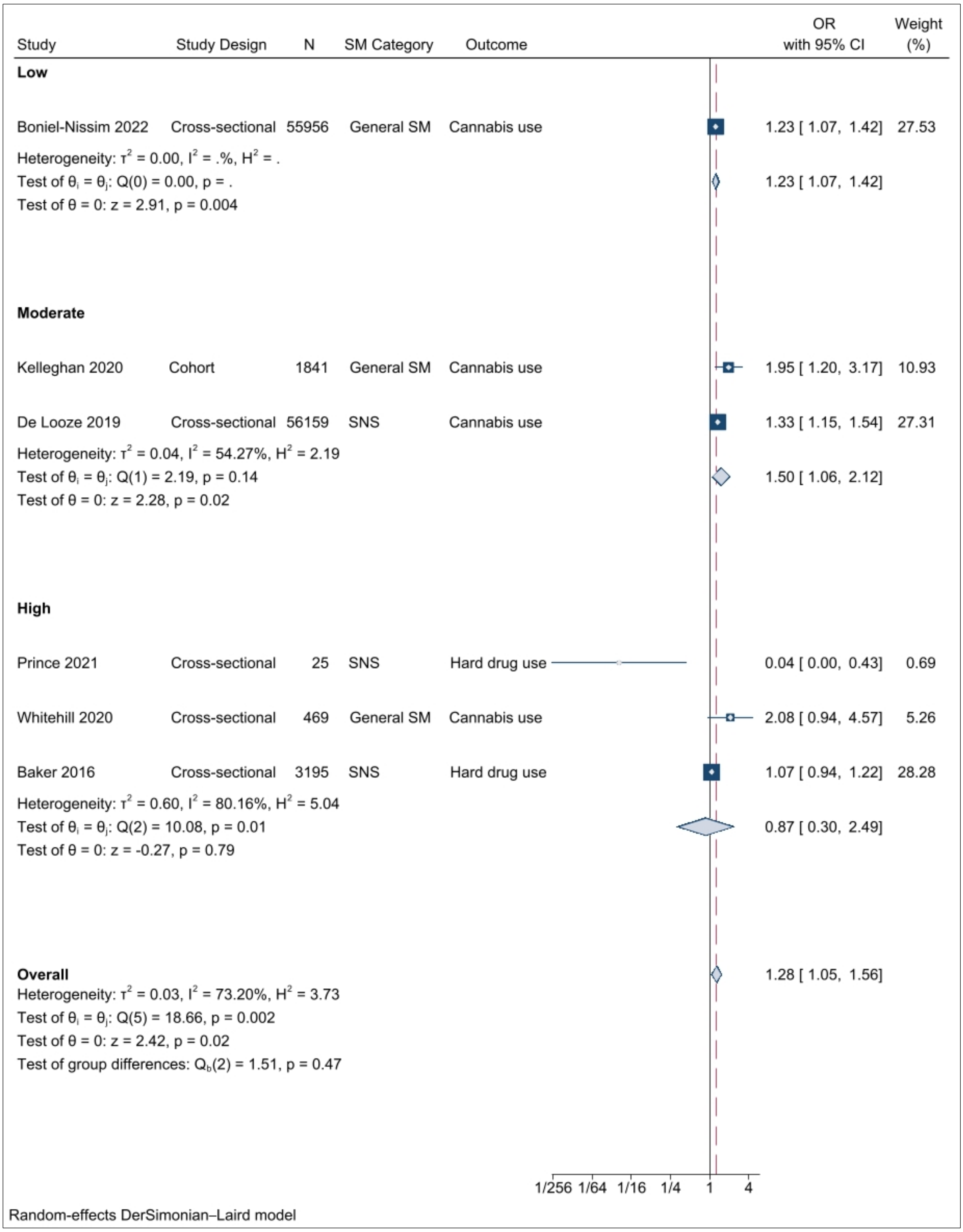

Legend: Figure presents forest plot for binary exposure (frequent/daily vs infrequent/non-daily) & binary/continuous outcome sensitivity analysis, with odds ratio (OR) used as common metric. Total number of study participants = 117,645. Abbreviations: CI = Confidence interval; N = Number of study participants; OR = Odds ratio; SM = Social media; and SNS = Social networking sites.

Figure DA. Forest plot for association between time spent on social media and drug use, by risk of bias grade

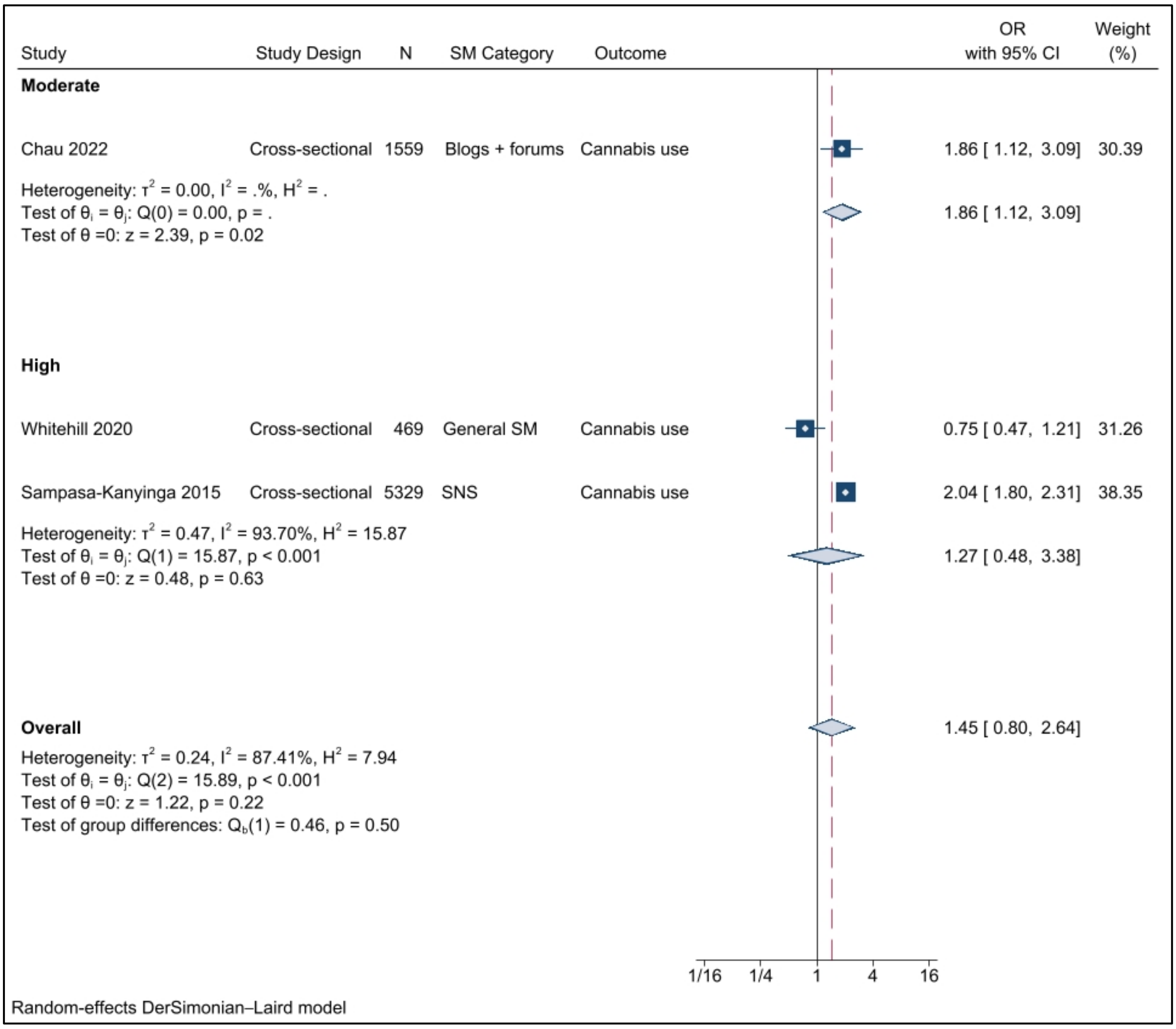

Legend: Figure presents forest plot for binary exposure (≤1hrs vs >1hr/day) & binary/continuous outcome meta-analysis, with odds ratio (OR) used as common metric. Total number of study participants = 7,357. Abbreviations: CI = Confidence interval; N = Number of study participants; OR = Odds ratio; SM = Social media; and SNS = Social networking sites.

Figure DB. Forest plot for association between frequency of social media use and sexual risk behaviour, by risk of bias grade

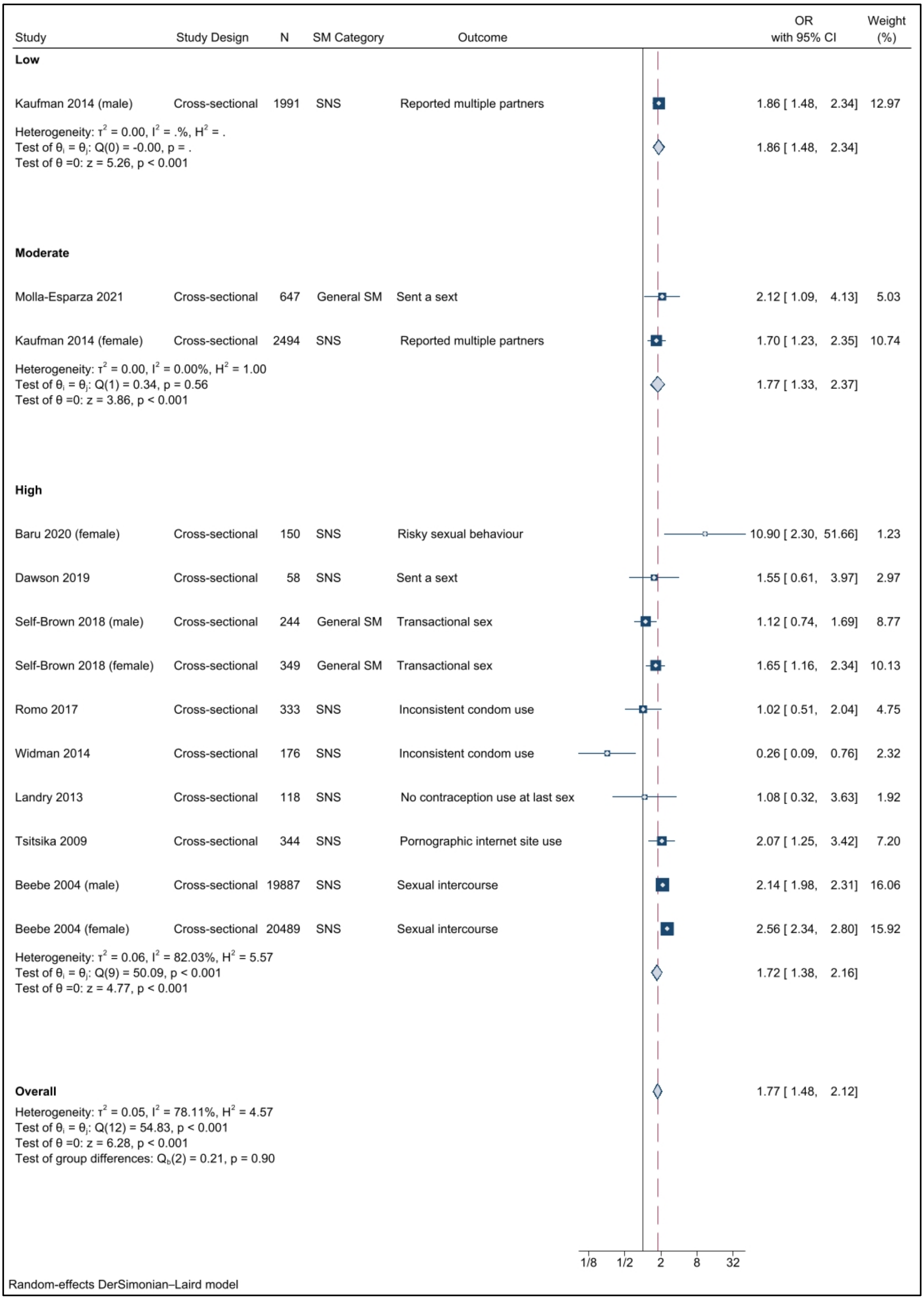

Legend: Figure presents forest plot for binary exposure (frequent/at all vs infrequent/not at all) & binary/continuous outcome sensitivity analysis, with odds ratio (OR) used as common metric. Total number of study participants = 47,280. Abbreviations: CI = Confidence interval; N = Number of study participants; OR = Odds ratio; SM = Social media; and SNS = Social networking sites.

Figure DC. Forest plot for association between frequency of social media use and sexual risk behaviour, by risk of bias grade

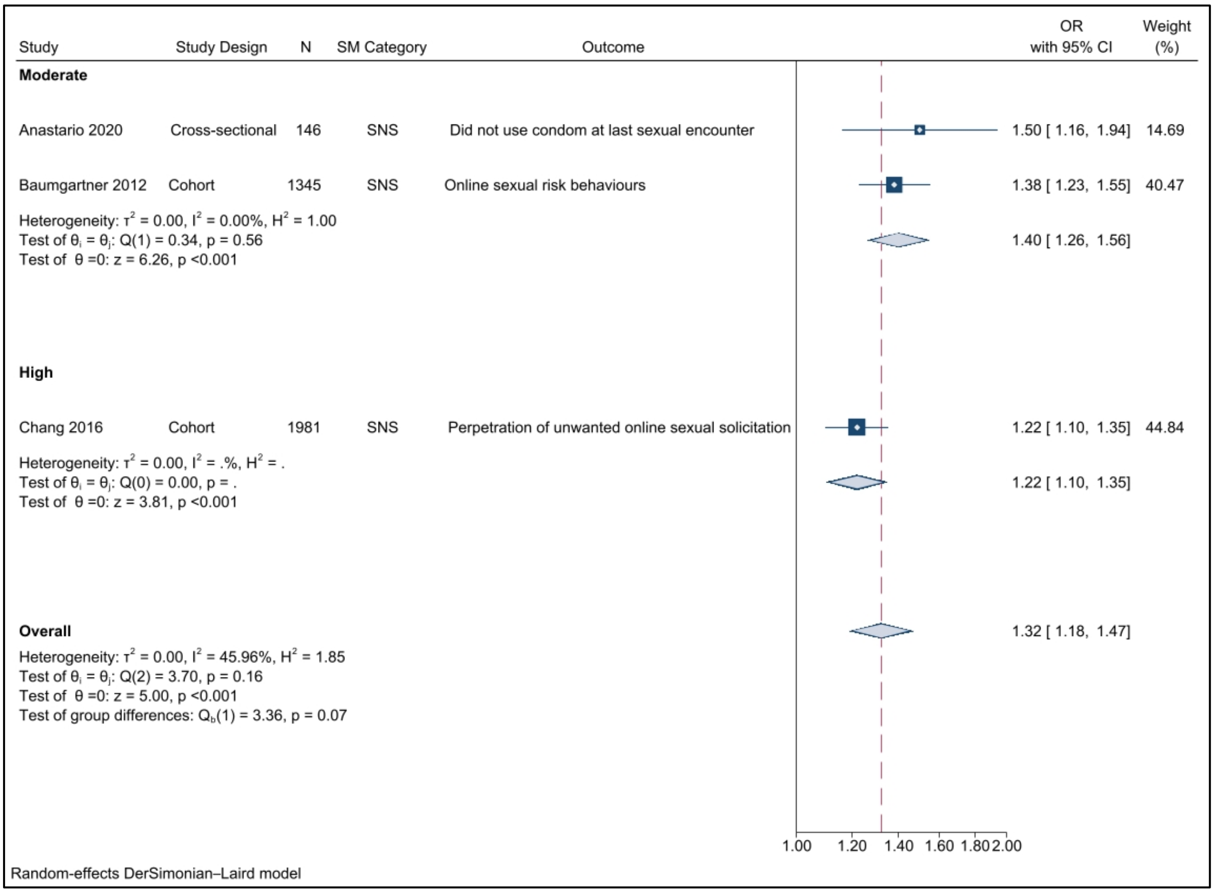

Legend: Figure presents forest plot for continuous exposure & binary outcome sensitivity analysis, with odds ratio (OR) used as common metric. Total number of study participants = 3,472. Abbreviations: CI = Confidence interval; N = Number of study participants; OR = Odds ratio; SM = Social media; and SNS = Social networking sites.

Figure DD. Forest plot for association between frequency of social media use and anti-social behaviour, by risk of bias grade

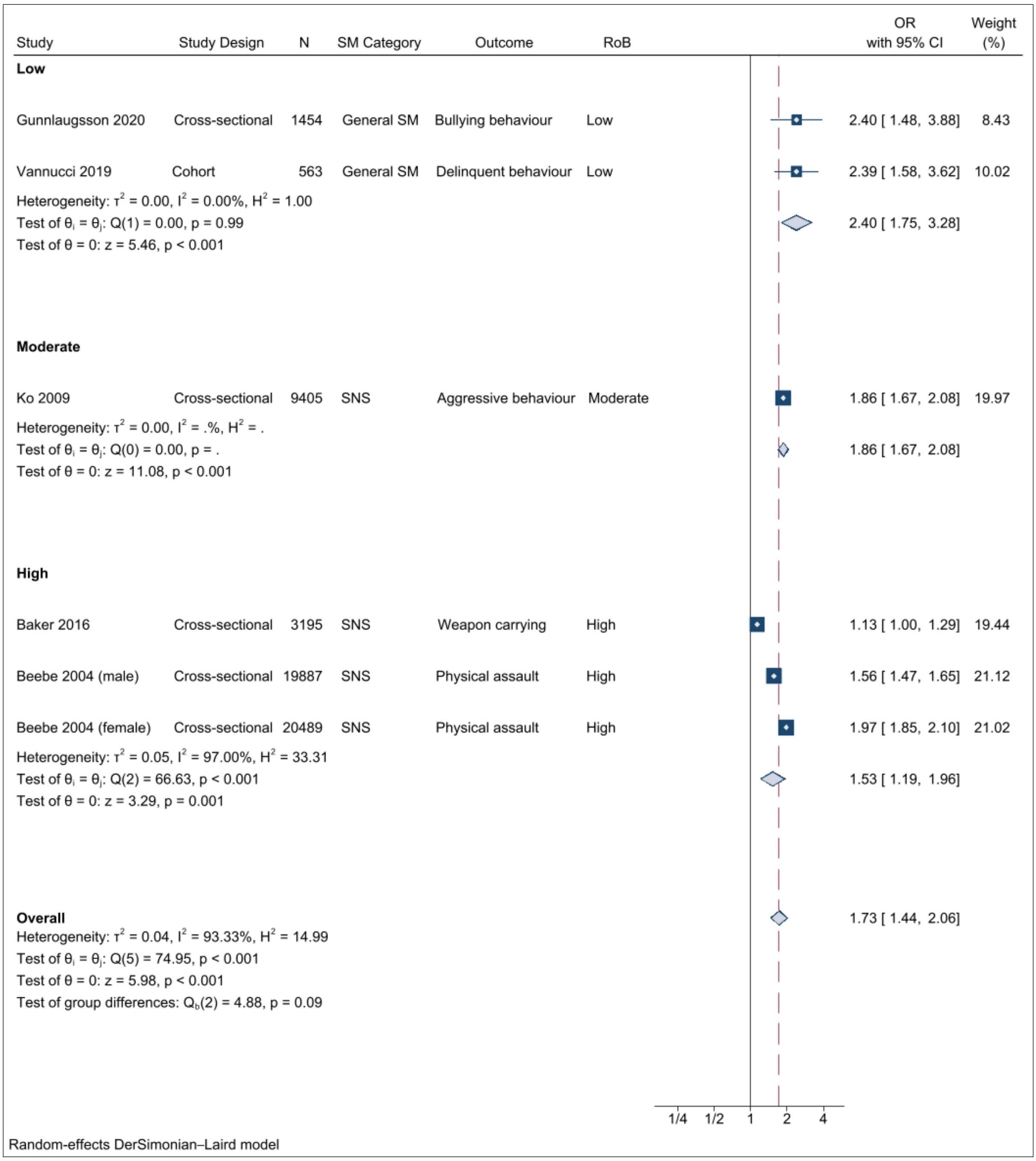

Legend: Figure presents forest plot for binary exposure (frequent/at all vs infrequent/not at all) & binary/continuous outcome sensitivity analysis, with odds (OR) used as common metric. Total number of study participants = 54,993. Abbreviations: CI = Confidence interval; N = Number of study participants; OR = Odds ratio; SM = Social media; and SNS = Social networking sites

Figure DE. Forest plot for association between frequency of social media use and tobacco use, by risk of bias grade

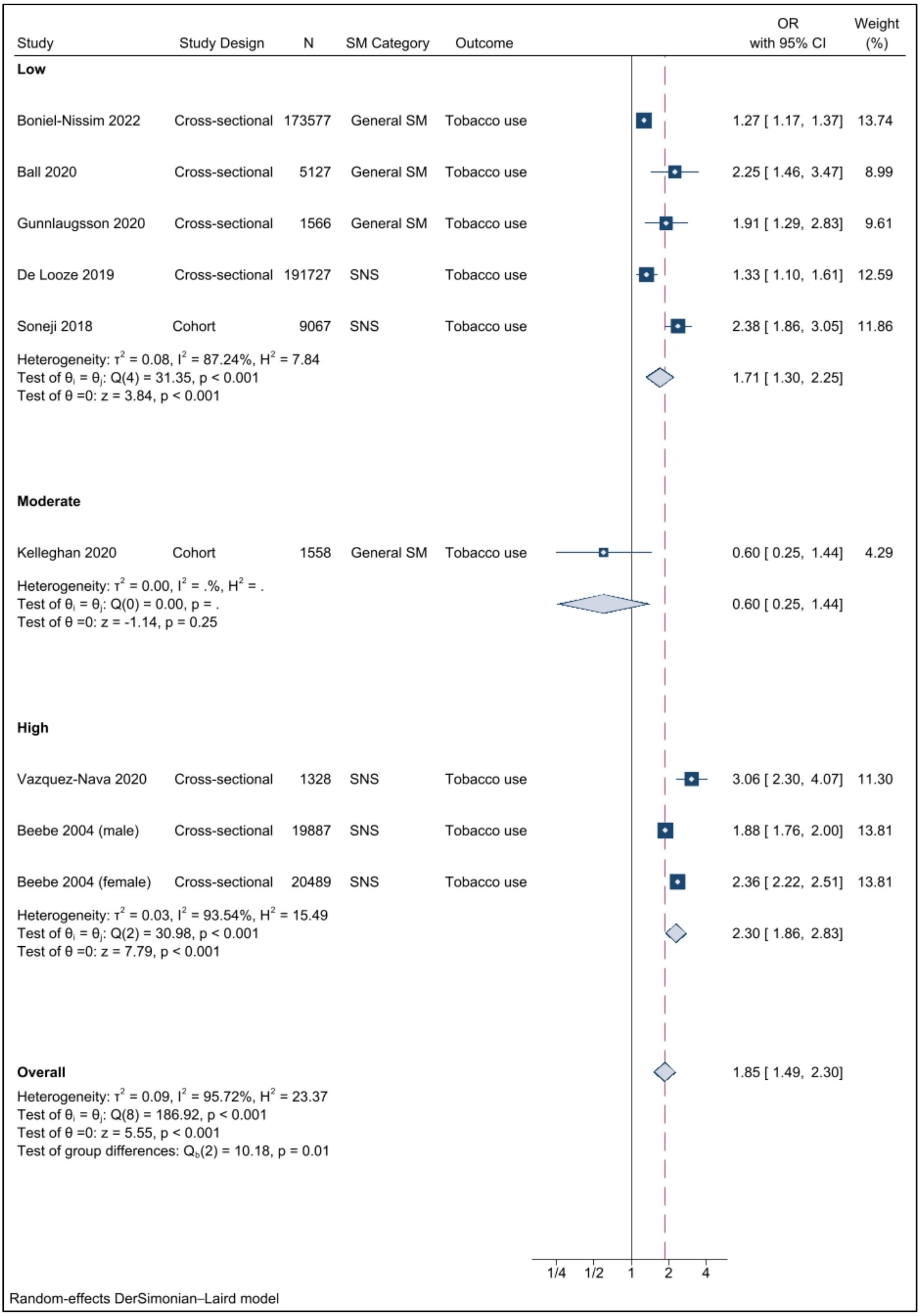

Legend: Figure presents forest plot for binary exposure (frequent vs infrequent) & binary/continuous outcome sensitivity analysis, with odds ratio (OR) used as common metric. Total number of study participants = 424,326. Abbreviations: CI = Confidence interval; N = Number of study participants; OR = Odds ratio; SM = Social media; and SNS = Social networking sites.

Figure DF. Forest plot for association between exposure to health-risk behaviour content on social media and tobacco use, by risk of bias

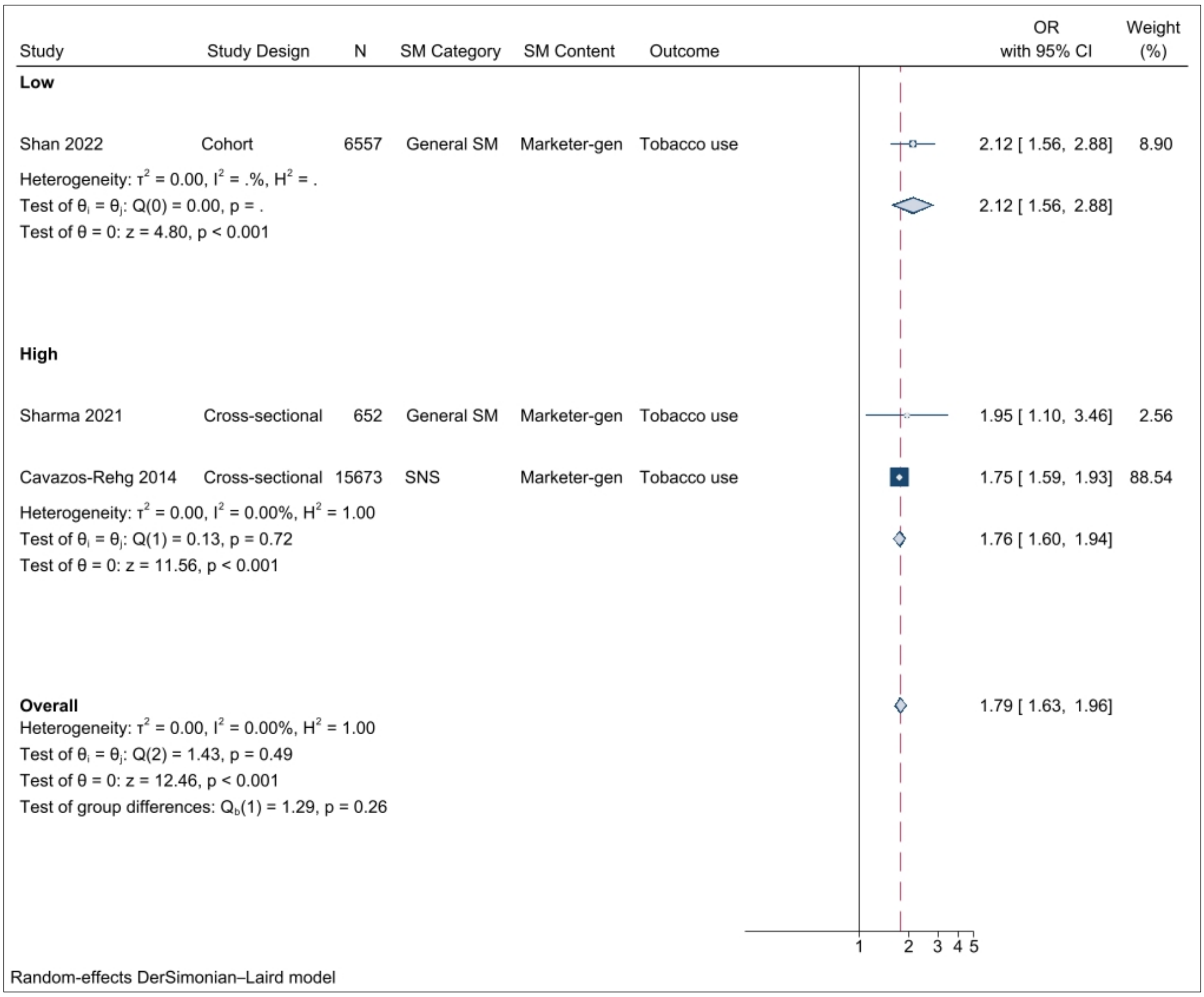

Legend: Figure presents forest plot for binary exposure (exposed vs unexposed) & binary/continuous outcome sensitivity analysis, with odds ratio (OR) used as common metric. Total number of study participants = 22,882. Abbreviations: CI = Confidence interval; Marketer-gen = Marketer-generated content; N = Number of study participants; OR = Odds ratio; SM = Social media; and SNS = Social networking sites.

Figure DG. Forest plot for association between frequency of social media use and gambling, by risk of bias grade

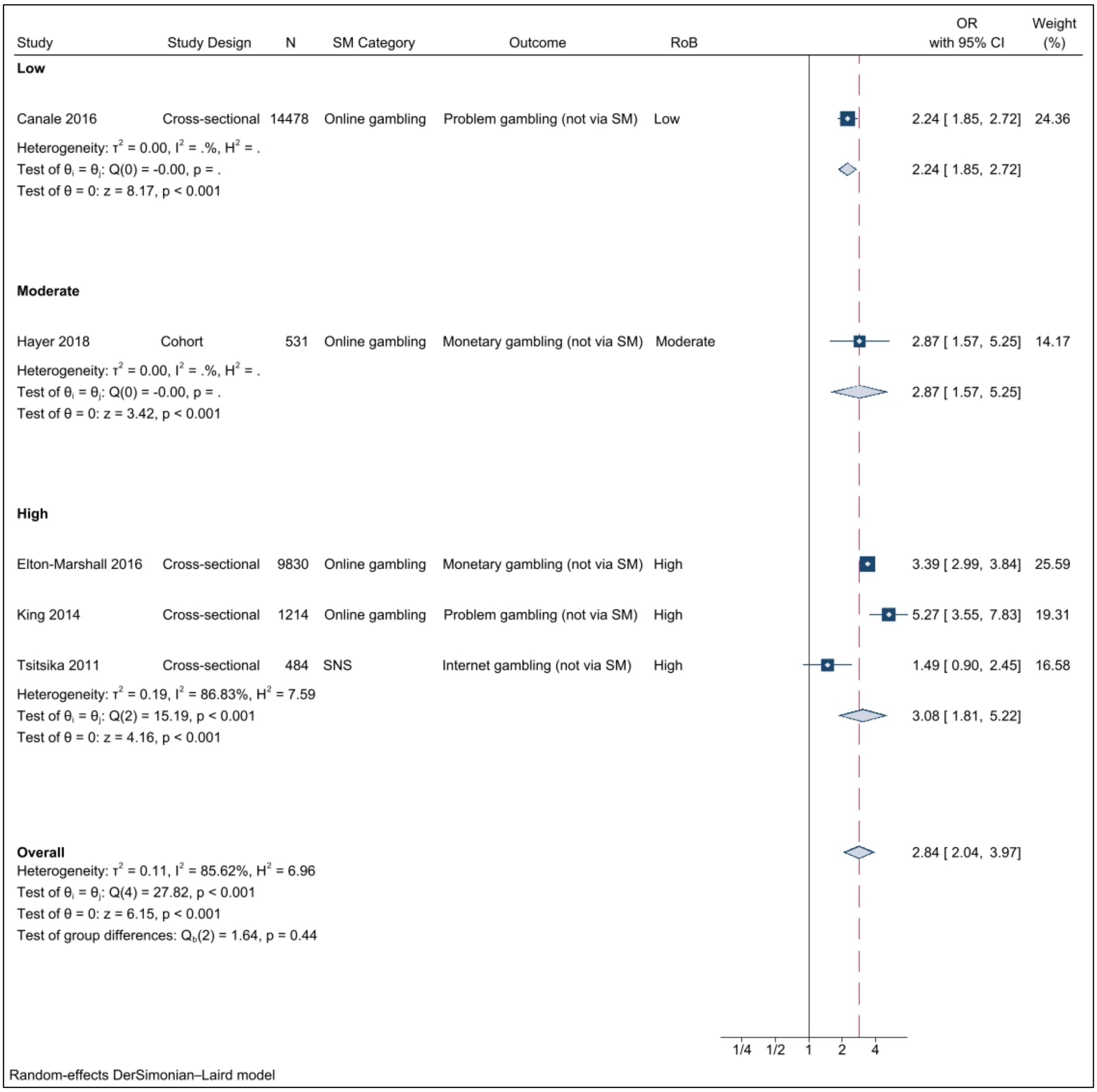

Legend: Figure presents forest plot for binary exposure (frequent/at all vs infrequent/not at all) & binary/continuous sensitivity analysis, with odds ratio (OR) used as common metric. Total number of study participants = 26,537. Abbreviations: CI = Confidence interval; N = Number of study participants; OR = Odds ratio; SM = Social media; and SNS = Social networking sites.

Figure DH. Forest plot for association between exposure to health-risk behaviour content on social media and use of electronic nicotine delivery systems, by risk of bias grade

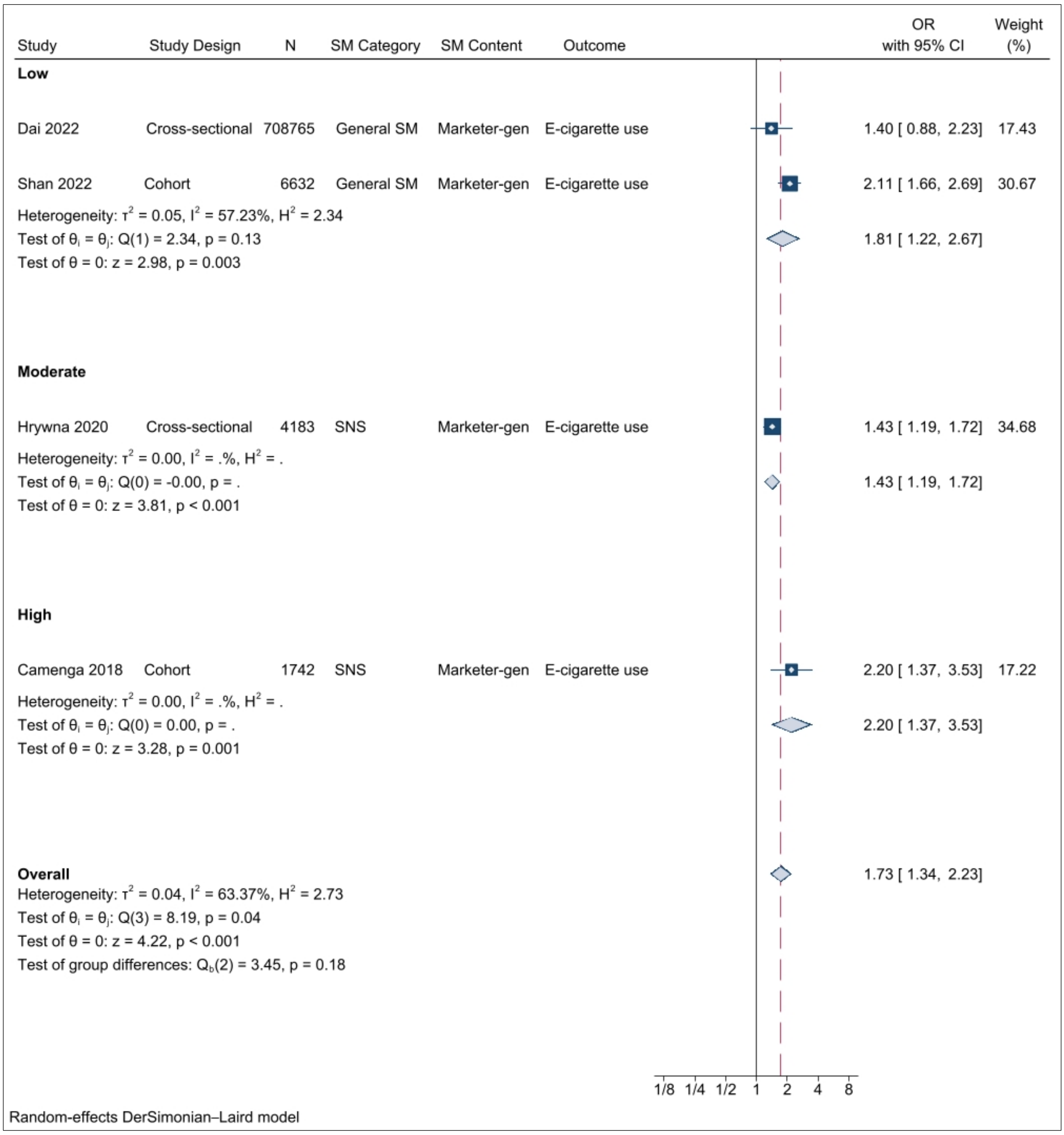

Legend: Figure presents forest plot for binary exposure (exposed vs unexposed) & binary/continuous outcome sensitivity analysis with odds ratio (OR) used as common metric. Total number of study participants = 721,322. Abbreviations: CI = Confidence interval; N = Number of study participants; OR = Odds ratio; SM = Social media; and SNS = Social networking sites.

Figure DI. Forest plot for association between time spent on social media and inadequate physical activity, by risk of bias grade

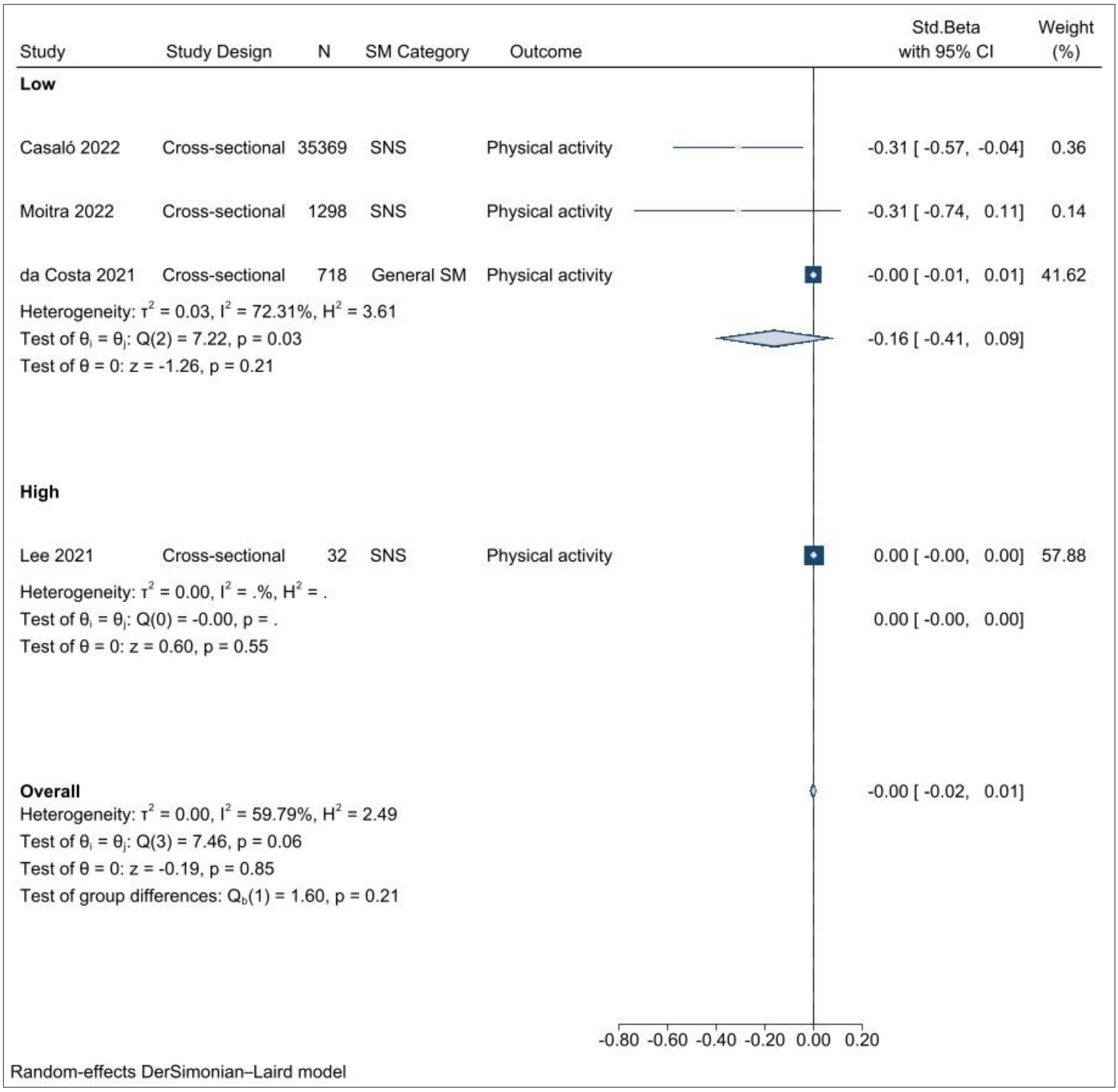

Legend: Figure presents forest plot for continuous exposure & continuous outcome sensitivity analysis, with standardised beta (Std. Beta) used as common metric. Total number of study participants =37,417. Abbreviations: CI = Confidence interval; N = Number of study participants; SM = Social media; SNS = Social networking sites; and Std. Beta = Standardised beta.

Figure DJ. Forest plot for association between time spent on social media and alcohol use, excluding datapoints which overlap 10-19 years

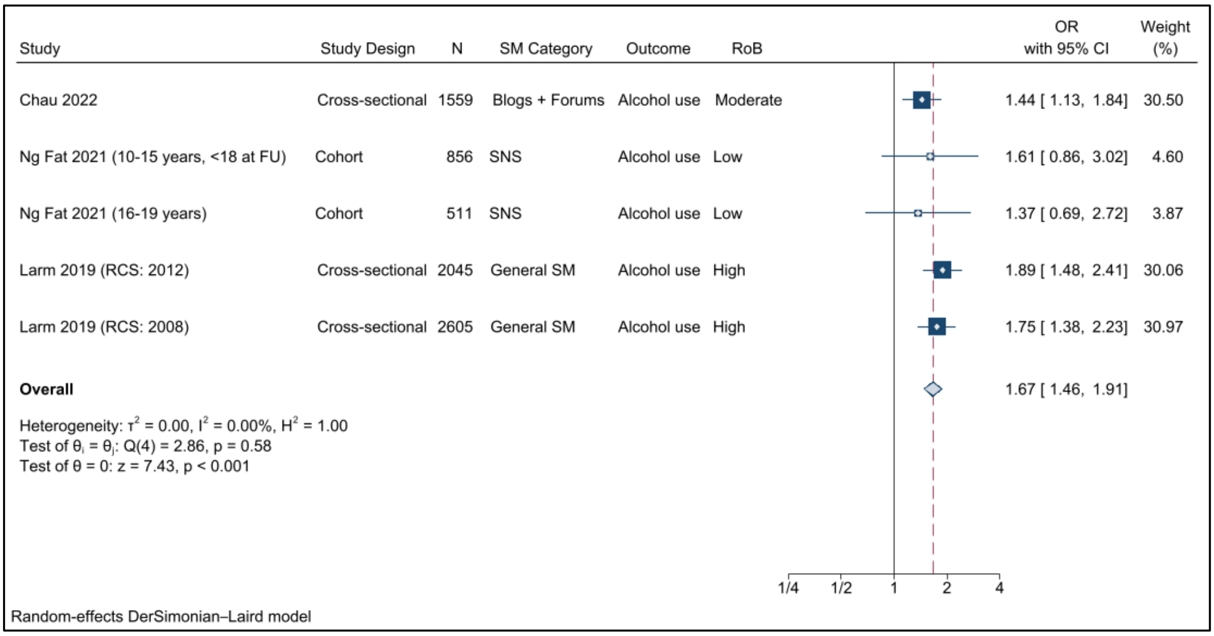

Legend: Figure presents forest plot for binary exposure (≥2 vs <2 hrs/day social media use) & binary/continuous outcome sensitivity analysis, with odds ratio (OR) used as common metric. Total number of study participants = 7,576. Abbreviations: CI = Confidence interval; FU = Follow up; hrs = Hours; N = Number of study participants; OR = Odds ratio; RCS = Repeat cross-sectional study; RoB = Risk of bias; SM = Social media; and SNS = Social networking sites.

Figure DK. Forest plot for association between frequency of social media use and alcohol use, excluding datapoints which overlap 10-19 years

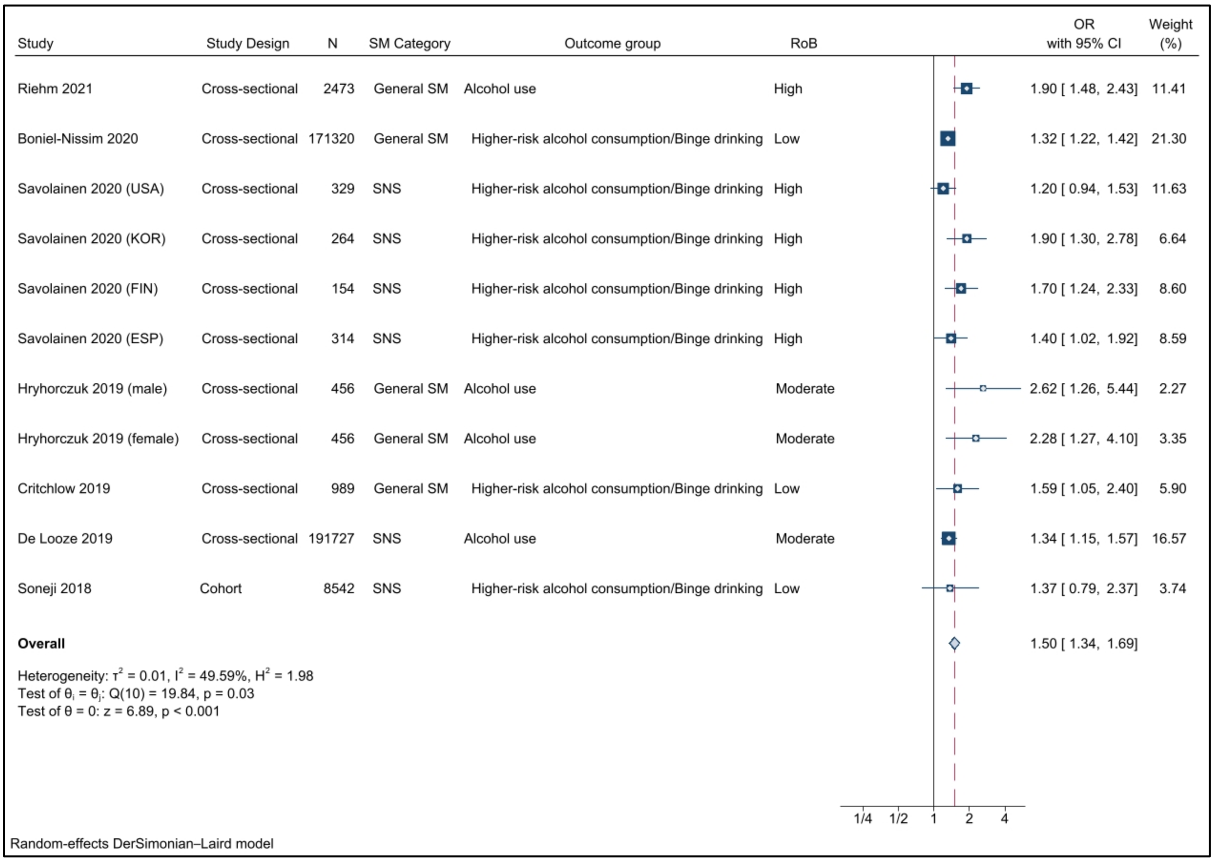

Legend: Figure presents forest plot for binary exposure (frequent/daily vs infrequent/non-daily) & binary/continuous outcome sensitivity analysis, with odds ratio (OR) used as common metric. Total number of study participants = 377,024. Abbreviations: CI = Confidence interval; ESP = Spain; FIN = Finland; KOR = South Korea; N = Number of study participants; OR = Odds ratio; RoB = Risk of bias; SM = Social media; and SNS = Social networking sites.

Figure DL. Forest plot for association between exposure to health-risk behaviour content on social media and alcohol use, excluding datapoints which overlap 10-19 years

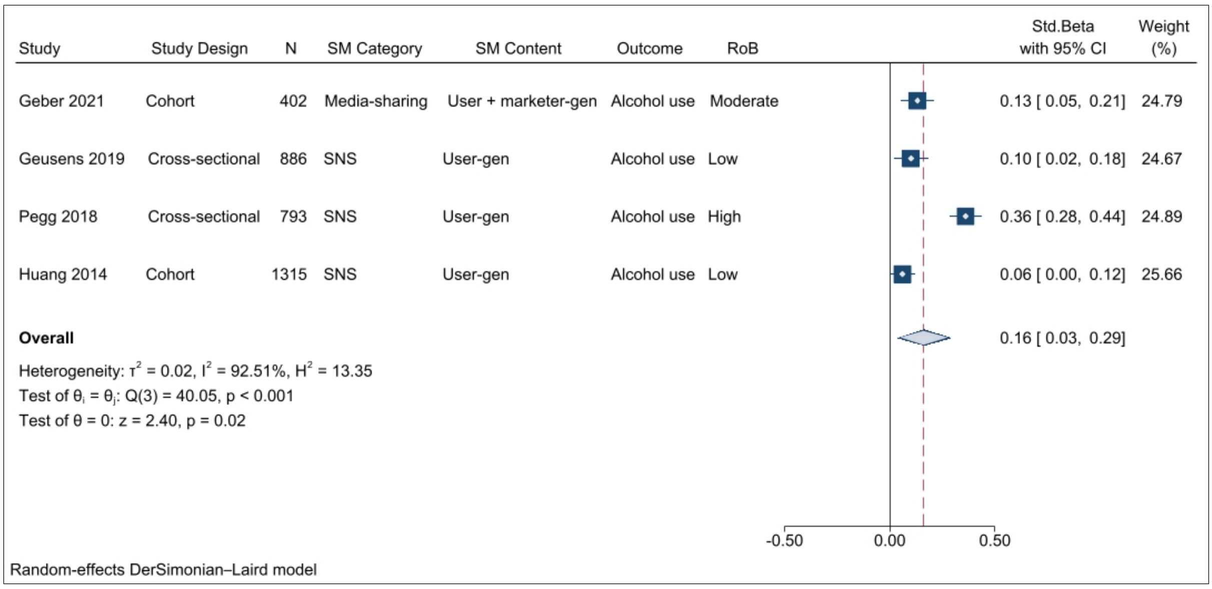

Legend: Figure presents forest plot for continuous exposure & continuous outcome sensitivity analysis, with standardised beta (Std.Beta) used as common metric. Total number of study participants = 3,396. Abbreviation: CI = Confidence interval; N = Number of study participants; Marketer-gen = Marketer-generated content; RoB = Risk of bias; SM = Social media; SNS = Social networking sites; Std. Beta = Standardised beta; and User-gen = User-generated content.

Figure DM. Forest plot for association between frequency of social media use and tobacco use, excluding datapoints which overlap 10-19 years

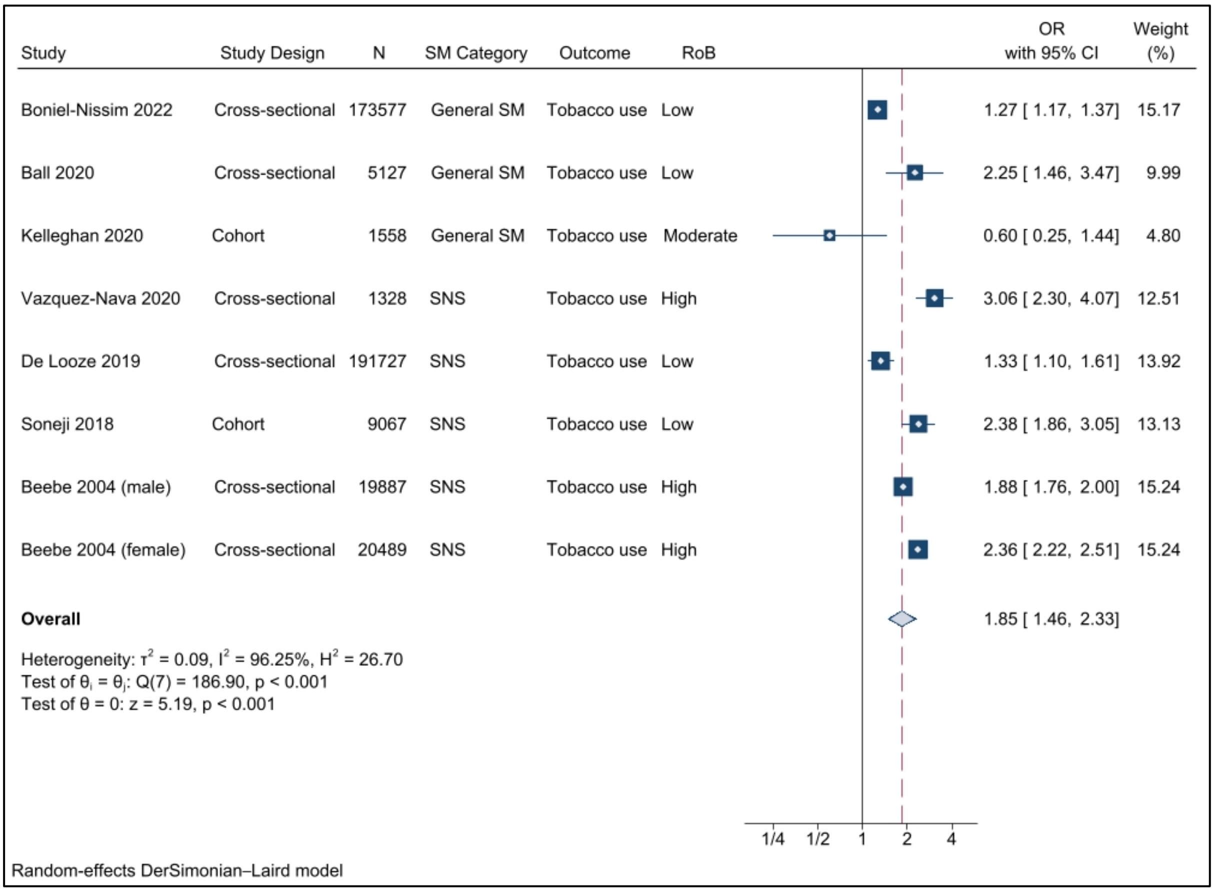

Legend: Figure presents forest plot for binary exposure (frequent vs infrequent) & binary/continuous outcome sensitivity analysis, with odds ratio (OR) used as common metric. Total number of study participants = 422,760. Abbreviations: CI = Confidence interval; N = Number of study participants; OR = Odds ratio; RoB = Risk of bias; SM = Social media; and SNS = Social networking sites.

Figure DN. Forest plot for association between frequency of social media use and sexual risk behaviour, excluding datapoints which overlap 10-19 years

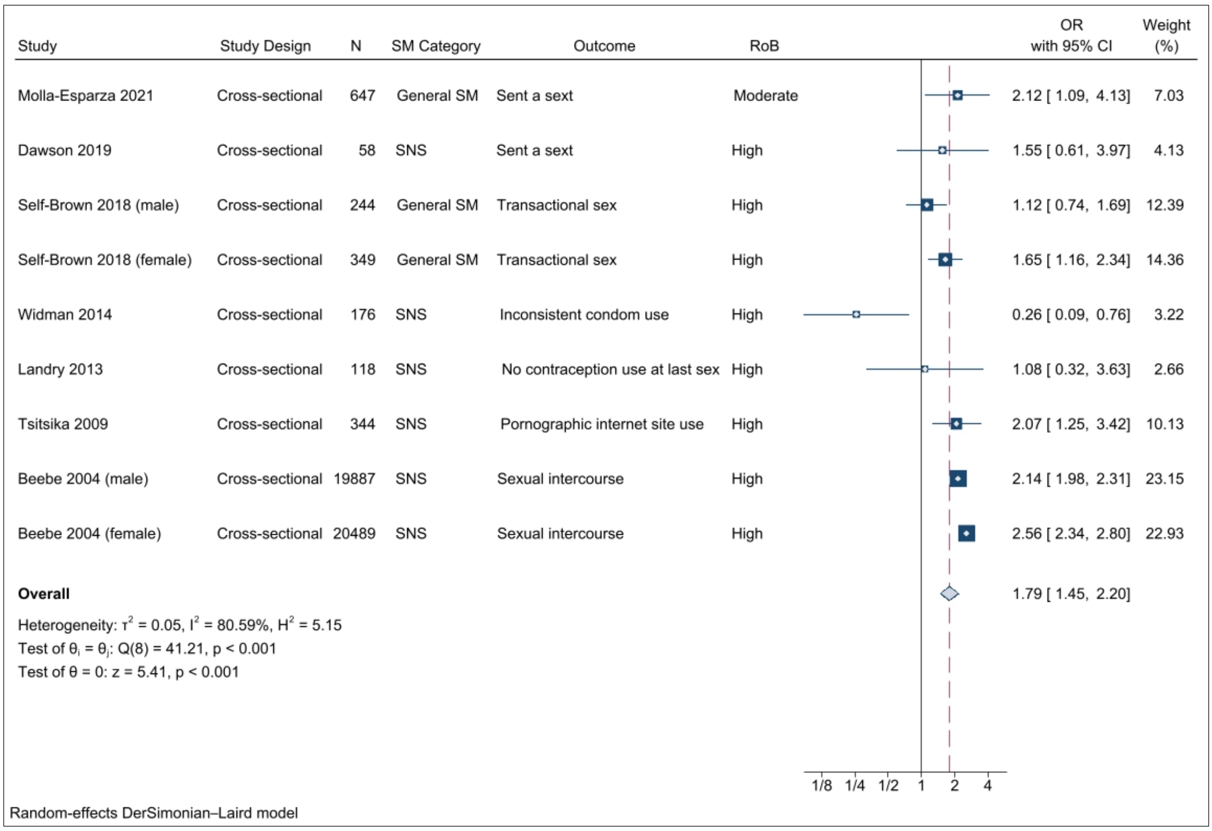

Legend: Figure presents forest plot for binary exposure (frequent/at all vs infrequent/not at all) & binary/continuous outcome sensitivity analysis, with odds ratio (OR) used as common metric. Total number of study participants = 42,312. Abbreviations: CI = Confidence interval; N = Number of study participants; OR = Odds ratio; RoB = Risk of bias; SM = Social media; and SNS = Social networking sites.

Figure DO. Forest plot for association between frequency of social media use and anti-social behaviour, excluding datapoints which overlap 10-19 years

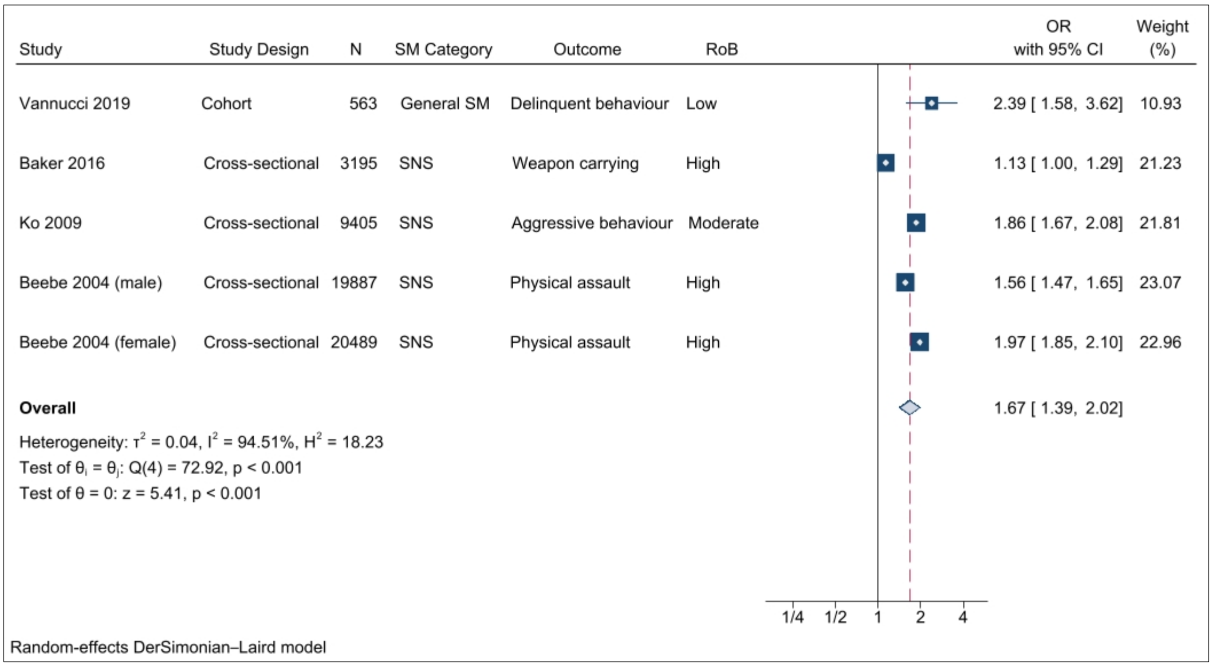

Legend: Figure presents forest plot for binary exposure (frequent/at all vs infrequent/not at all) & binary/continuous outcome sensitivity analysis, with odds (OR) used as common metric. Total number of study participants = 53,539. Abbreviations: CI = Confidence interval; N = Number of study participants; OR = Odds ratio; RoB = Risk of bias; SM = Social media; and SNS = Social networking sites.

Table A. Summary of meta-regression findings

Exposure	Outcome	Study level variable	Coefficient (95% CI)	p-value
Frequency of SM use	Tobacco use	SM category (reference category- SNS)	General SM: -0.34 (-0.68 to 0.01)	0.06
Frequency of SM use	Sexual risk behaviour	Average age of study participants (reference category- <16 years)	**≥16 years: -0.37 (-0.70 to -0.05)**	**0.03**
		SM category (reference category- General SM)	SNS: 0.29 (-0.08 to 0.66)	0.13
		Development status of study setting (reference category- Low-middle income)	High income: 0.12 (-0.22 to 0.46)	0.49

Legend: Results with $p < 0.05$ in bold. Abbreviations: 95% CI = 95% confidence interval; SM = Social media; and SNS = Social networking sites.

Appendix 17. Assessment of publication bias/small study effects

Figure A. Contour enhanced funnel plot for meta-analysis of studies investigating the effect of frequency of social media use (frequent vs infrequent) on sexual risk behaviour, and Egger's test result

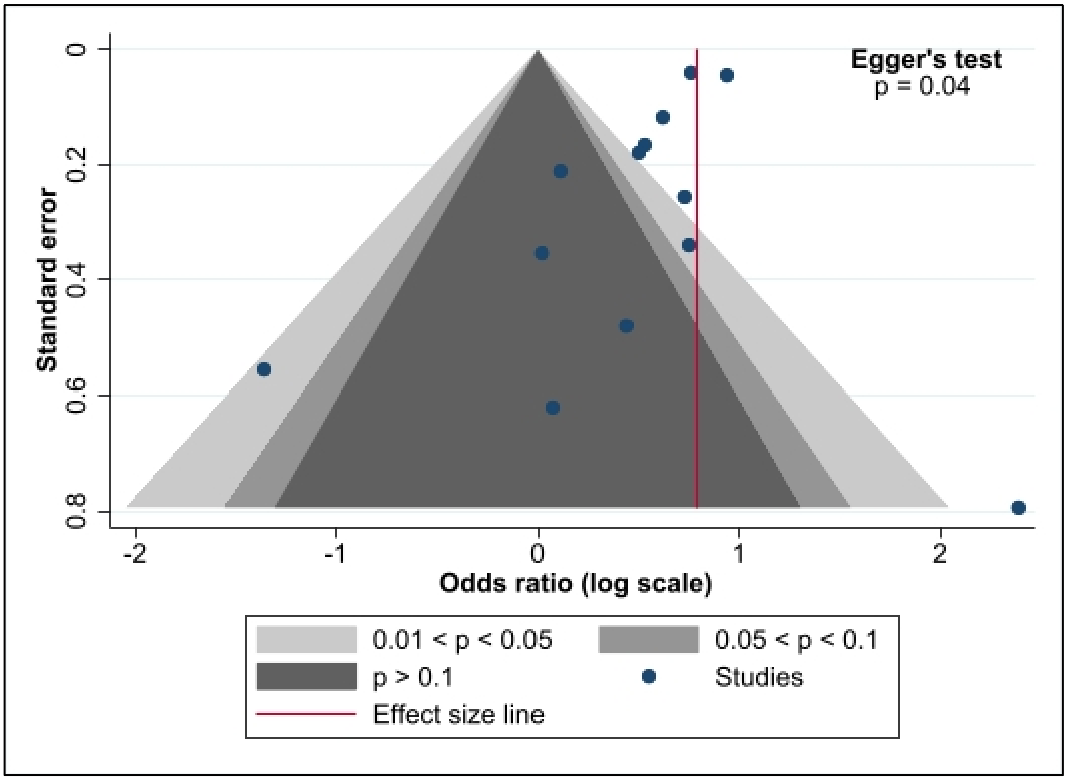

Appendix 18. Summary of findings and certainty of evidence

Table A. Summary of findings and certainty of evidence for seven priority outcomes (as per GRADE) with reasons for upgrading/downgrading of the evidence

Population/setting: Adolescents aged 10-19 years in high and low-middle income settings
Intervention: Frequent social media use
Comparison: Infrequent social media use

Outcome	Anticipated absolute effects[a] (95% CI)		Relative effect (95% CI)	No. of participants (studies)	Certainty of the evidence (GRADE)	Comments
	Risk with infrequent social media use	Risk with frequent social media use				
Alcohol use	48.9% of participants in the control group used alcohol	58.6% of exposed group participants used alcohol (56.4 to 60.8%)	**OR 1.48** (1.35 to 1.62)	383,068 (9 observational studies)	⊕⊕⊖⊖ **Low** [a,b,c,d,e]	Frequent social media use increases adolescent alcohol use. **Absolute effect calculated from Riehm 2021.**[123]
Drug use	17.0% of participants in the control group used drugs	20.8% of exposed group participants used drugs (17.7 to 24.2%)	**OR 1.28** (1.05 to 1.56)	117,645 (6 observational studies)	⊕⊖⊖⊖ **Very low** [f,g,h]	Downgraded for RoB. Frequent social media use may increase adolescent drug use. **Absolute effect calculated from Whitehill 2020.**[153]
Tobacco use	12.1% of participants in the control group used tobacco	20.3% of exposed group participants used tobacco (17.0 to 24.0%)	**OR 1.85** (1.49 to 2.30)	424,326 (8 observational studies)	⊕⊖⊖⊖ **Very low** [i,j,k]	Downgraded for RoB and inconsistency. Frequent social media use may increase adolescent tobacco use. **Absolute effect calculated from Vazquez-Nava 2020.**[149]
Electronic nicotine delivery system use	66.7% of studies demonstrated a harmful effect of social media use on adolescent use of electronic nicotine delivery systems (95% CI 20.8 to 93.9%)			18,047 (3 observational studies)	⊕⊕⊖⊖ **Very low** [l,m]	Downgraded for RoB and imprecision. Frequent social media use may increase adolescent use of ENDS.
Sexual risk behaviour	37.0% of participants in the control group engaged in sexual risk behaviours	50.9% of exposed group participants engaged in sexual risk behaviours (46.5 to 55.4%)	**OR 1.77** (1.48 to 2.12)	47,280 (10 observational studies)	⊕⊖⊖⊖ **Very low** [n,o,p,q,r]	Downgraded for RoB and publication bias. Frequent social media use may increase in adolescent sexual risk behaviours. **Absolute effect calculated from Self-Brown 2018**.[134]
Gambling	21.4% of participants in the control group engaged in gambling	43.6% of exposed group participants engaged in gambling (35.7 to 52.0%)	**OR 2.84** (2.04 to 3.97)	26,537 (5 observational studies)	⊕⊖⊖⊖ **Very low** [s,t,u]	Downgraded for RoB. Frequent social media use may increase adolescent gambling. **Absolute effect calculated from King 2012.**[93]

Outcome	Anticipated absolute effects[a] (95% CI)		Relative effect (95% CI)	No. of participants (studies)	Certainty of the evidence	Comments
	Risk with infrequent SM use	Risk with frequent SM use				
Multiple risk behaviours	41.3% of participants in the control group engaged in multiple risk behaviours	55.2% of exposed group participants engaged in multiple risk behaviours (47.8 to 62.3%)	**OR 1.75** (1.30 to 2.35)	43,571 (2 observational studies)	⊕⊖⊖⊖ **Very low** [v,x,y]	Downgraded for RoB and inconsistency. Frequent social media use may increase adolescent engagement in multiple risk behaviours. **Absolute effect calculated from Beebe 2005.**[39]

GRADE Working Group grades of evidence
High certainty ⊕⊕⊕⊕: We are very confident that the true effect lies close to that of the estimate of the effect
Moderate certainty ⊕⊕⊕⊖: We are moderately confident in the effect estimate: The true effect is likely to be close to the estimate of the effect, but there is a possibility that it is substantially different
Low certainty ⊕⊕⊖⊖: Our confidence in the effect estimate is limited: The true effect may be substantially different from the estimate of the effect
Very low certainty ⊕⊖⊖⊖: We have very little confidence in the effect estimate: The true effect is likely to be substantially different from the estimate of effect

Explanations for upgrading/downgrading the evidence
[a] Not downgraded for RoB, as most studies were low RoB, assessed exposure and outcome via validated tools, and no difference in effect size in high RoB studies was observed on stratification.
[b] Not downgraded for inconsistency as heterogeneity was moderate (I^2 = 39.3%) and all 95% confidence intervals overlapped.
[c] Not downgraded for indirectness as only 1/9 studies assessed text messaging as well as social media use.
[d] Not downgraded for imprecision as 95% confidence interval did not cross the null effect, was narrow and did not include appreciable harm or benefit.
[e] Unable to assess publication bias via a funnel plot due to insufficient data, however as a systematic search was conducted the chance of publication bias is reduced.
[f] Downgraded for RoB as half of the studies were rated high RoB, only one study assessed exposure and outcome via validated tools, and all but one study failed to adjust for critical confounding domains.
[g] Not downgraded for indirectness as majority of included studies specifically assessed social media use.
[h] Not downgraded for imprecision as 95% confidence interval did not cross the null effect, was narrow and did not include appreciable harm or benefit.
[i] Downgraded for RoB, as although majority of included studies were low/moderate RoB, the contributing high RoB studies report notably larger effect sizes.
[j] Downgraded for serious inconsistency as heterogeneity of included studies was considerable (I^2 = 95.7%).
[k] Not downgraded for imprecision as 95% confidence interval did not cross null effect, was narrow and did not include appreciable harm or benefit.
[l] Downgraded for RoB as all studies were moderate/high RoB, failed to adjust for critical confounding domains, and assessed exposure and outcome via non-validated tools.
[m] Downgraded for serious imprecision as wide 95% confidence interval suggests lack of confidence in estimate.
[n] Downgraded for RoB, as majority of studies were high RoB, failed to adjust for critical confounding domains, and assessed exposure and outcome via non-validated tools.
[o] Not downgraded for serious inconsistency as heterogeneity was reduced when stratification was performed by socioeconomic position, age, social media category and development status of study setting.
[p] Not downgraded for indirectness as only 1/10 studies assessed text messaging as well as social media use.
[q] Not downgraded for imprecision as 95% confidence interval did not cross null effect, was narrow and did not include appreciable harm or benefit.
[r] Downgraded for suspected publication bias given impression from asymmetric contour enhanced funnel plot.
[s] Downgraded for RoB as majority of studies were high RoB, failed to adjust for critical confounding domains, and assessed exposure via non-validated tools.
[t] Not downgraded for indirectness as no concerns were raised regarding population, intervention, comparator, direct comparisons, or outcome.
[u] Not downgraded for imprecision as 95% confidence interval does not cross null effect and does not include appreciable harm or benefit.
[v] Downgraded for RoB as all studies were high RoB, failed to adjust for critical confounding domains, and assessed exposure and outcome via non-validated tools.
[w] Downgraded for serious inconsistency as heterogeneity of included studies was considerable (I^2 = 97.9%) and confidence intervals show no or minimal overlap.
[x] Not downgraded for imprecision as 95% confidence interval did not cross null effect, was narrow and did not include appreciable harm or benefit.

Legend: [a] The risk in the intervention group (and its 95% confidence interval) is based on the assumed risk in the comparison group and the relative effect of the intervention (and its 95% CI). Abbreviations: CI = Confidence interval; OR = Odds ratio; and RoB = Risk of bias.

Table B. Post-hoc analysis: summary of findings and certainty of evidence for unhealthy dietary behaviour (as per GRADE) with reasons for upgrading/downgrading of the evidence

Population/setting: Adolescents aged 10-19 years in high and low-middle income settings
Intervention: Exposure to health-risk behaviour content
Comparison: No exposure to health-risk behaviour content

Outcome	Effect direction	No. of participants (studies)	Certainty of the evidence (GRADE)	Comments
Unhealthy dietary behaviour (effect direction)	All studies demonstrated a harmful effect of social media use on adolescent engagement in unhealthy dietary behaviours (51.0 to 100.0%)	521 (4 randomised control trials)	⊕⊕⊕⊖ **Moderate** [a,b]	Downgraded for indirectness. Exposure to health-risk behaviour content on social media increases adolescent engagement in unhealthy dietary behaviours.

GRADE Working Group grades of evidence
High certainty ⊕⊕⊕⊕: We are very confident that the true effect lies close to that of the estimate of the effect
Moderate certainty ⊕⊕⊕⊖: We are moderately confident in the effect estimate: The true effect is likely to be close to the estimate of the effect, but there is a possibility that it is substantially different
Low certainty ⊕⊕⊖⊖: Our confidence in the effect estimate is limited: The true effect may be substantially different from the estimate of the effect
Very low certainty ⊕⊖⊖⊖: We have very little confidence in the effect estimate: The true effect is likely to be substantially different from the estimate of effect

Explanations for upgrading/downgrading the evidence

[a] Not downgraded for RoB as all studies were RCT's, with two rated low RoB and two some concerns.
[b] Downgraded for serious indirectness of comparator, as two studies used a comparator group pertaining to healthy food exposure and the remaining two used a comparator group pertaining to exposure to non-food items.

Legend: Abbreviations: RoB = Risk of bias.

Appendix 19. PRISMA checklists

Table A. PRISMA 2020 checklist for systematic reviews and meta-analysis

Section and Topic	Item #	Checklist item	Location where item is reported
**TITLE**			
Title	1	Identify the report as a systematic review.	Manuscript: Title page<br>Manuscript: Abstract<br>Manuscript: Methods
**ABSTRACT**			
Abstract	2	See the PRISMA 2020 for Abstracts checklist.	Manuscript: Abstract<br>Appendix 19: Table B. PRISMA checklists
**INTRODUCTION**			
Rationale	3	Describe the rationale for the review in the context of existing knowledge.	Manuscript: Introduction
Objectives	4	Provide an explicit statement of the objective(s) or question(s) the review addresses.	Manuscript: Introduction
**METHODS**			
Eligibility criteria	5	Specify the inclusion and exclusion criteria for the review and how studies were grouped for the syntheses.	Manuscript: Study inclusion and exclusion criteria<br>Appendix 5: Process of social media categorisation
Information sources	6	Specify all databases, registers, websites, organisations, reference lists and other sources searched or consulted to identify studies. Specify the date when each source was last searched or consulted.	Manuscript: Search methods for identification of studies<br>Manuscript: Figure-2 PRISMA study flow chart<br>Appendix 3: Details of search strategies conducted
Search strategy	7	Present the full search strategies for all databases, registers and websites, including any filters and limits used.	Manuscript: Search methods for identification of studies<br>Appendix 3: Details of search strategies conducted
Selection process	8	Specify the methods used to decide whether a study met the inclusion criteria of the review, including how many reviewers screened each record and each report retrieved, whether they worked independently, and if applicable, details of automation tools used in the process.	Manuscript: Study inclusion and exclusion criteria<br>Manuscript: Selection of studies<br>Manuscript: Data extraction and RoB assessment
Data collection process	9	Specify the methods used to collect data from reports, including how many reviewers collected data from each report, whether they worked independently, any processes for obtaining or confirming data from study investigators, and if applicable, details of automation tools used in the process.	Manuscript: Selection of studies<br>Manuscript: Data extraction and RoB assessment

Section and Topic	Item #	Checklist item	Location where item is reported
			Appendix 8: Data extraction form exemplar
Data items	10a	List and define all outcomes for which data were sought. Specify whether all results that were compatible with each outcome domain in each study were sought (e.g. for all measures, time points, analyses), and if not, the methods used to decide which results to collect.	Manuscript: Study inclusion and exclusion criteria<br>Appendix 6: Included outcomes<br>Appendix 7: Meta-analyses and synthesis without meta-analysis (SWiM) decision rules
	10b	List and define all other variables for which data were sought (e.g. participant and intervention characteristics, funding sources). Describe any assumptions made about any missing or unclear information.	Manuscript: Study inclusion and exclusion criteria<br>Appendix 8: Data extraction form exemplar
Study risk of bias assessment	11	Specify the methods used to assess risk of bias in the included studies, including details of the tool(s) used, how many reviewers assessed each study and whether they worked independently, and if applicable, details of automation tools used in the process.	Manuscript: Data extraction and RoB assessment<br>Appendix 9: Newcastle-Ottawa Scale (NOS) risk of bias assessment
Effect measures	12	Specify for each outcome the effect measure(s) (e.g. risk ratio, mean difference) used in the synthesis or presentation of results.	Manuscript: Data synthesis<br>Appendix 10: Process for data transformations for meta-analysis
Synthesis methods	13a	Describe the processes used to decide which studies were eligible for each synthesis (e.g. tabulating the study intervention characteristics and comparing against the planned groups for each synthesis (item #5)).	Manuscript: Data synthesis<br>Appendix 7: Meta-analyses and synthesis without meta-analysis (SWiM) decision rules
	13b	Describe any methods required to prepare the data for presentation or synthesis, such as handling of missing summary statistics, or data conversions.	Manuscript: Data synthesis<br>Appendix 7: Meta-analyses and synthesis without meta-analysis (SWiM) decision rules<br>Appendix 10: Process for data transformations for meta-analysis
	13c	Describe any methods used to tabulate or visually display results of individual studies and syntheses.	Manuscript: Data synthesis<br>Appendix 7: Meta-analyses and synthesis without meta-analysis (SWiM) decision rules
	13d	Describe any methods used to synthesize results and provide a rationale for the choice(s). If meta-analysis was performed, describe the model(s), method(s) to identify the presence and extent of statistical heterogeneity, and software package(s) used.	Manuscript: Data synthesis<br>Appendix 15: Meta-analyses, meta-regression, subgroup, and sensitivity analyses
	13e	Describe any methods used to explore possible causes of heterogeneity among study results (e.g. subgroup analysis, meta-regression).	Manuscript: Data synthesis
	13f	Describe any sensitivity analyses conducted to assess robustness of the synthesized results.	Manuscript: Data synthesis

Section and Topic	Item #	Checklist item	Location where item is reported
Reporting bias assessment	14	Describe any methods used to assess risk of bias due to missing results in a synthesis (arising from reporting biases).	Manuscript: Data synthesis
Certainty assessment	15	Describe any methods used to assess certainty (or confidence) in the body of evidence for an outcome.	Manuscript: Certainty of the evidence
**RESULTS**			
Study selection	16a	Describe the results of the search and selection process, from the number of records identified in the search to the number of studies included in the review, ideally using a flow diagram.	Manuscript: Results<br>Manuscript: Figure-2 PRISMA study flow diagram
	16b	Cite studies that might appear to meet the inclusion criteria, but which were excluded, and explain why they were excluded.	Appendix 12: Characteristics of excluded studies
Study characteristics	17	Cite each included study and present its characteristics.	Manuscript: Results<br>Appendix 11: Characteristics of included studies
Risk of bias in studies	18	Present assessments of risk of bias for each included study.	Manuscript: Results<br>Appendix 13: Risk of bias domain and overall grade for included datapoints and studies<br>Appendix 18: Summary of findings and certainty of the evidence
Results of individual studies	19	For all outcomes, present, for each study: (a) summary statistics for each group (where appropriate) and (b) an effect estimate and its precision (e.g. confidence/credible interval), ideally using structured tables or plots.	Manuscript: Results<br>Manuscript: Figures 4A-C<br>Manuscript: Figures 5A-D<br>Appendix 16: Synthesis without meta-analysis, meta-analyses, meta-regression, subgroup, and sensitivity analyses
Results of syntheses	20a	For each synthesis, briefly summarise the characteristics and risk of bias among contributing studies.	Manuscript: Results<br>Appendix 13: Risk of bias domain and overall grade for included datapoints and studies<br>Appendix 18: Summary of findings and certainty of the evidence S13<br>Appendix 14: Social media measures reported in included studies
	20b	Present results of all statistical syntheses conducted. If meta-analysis was done, present for each the summary estimate and its precision (e.g. confidence/credible interval) and measures of statistical heterogeneity. If comparing groups, describe the direction of the effect.	Manuscript: Results<br>Manuscript: Figures 4A-C<br>Manuscript: Figures 5A-D<br>Appendix 16: Synthesis without meta-analysis, meta-analyses, meta-regression, subgroup, and sensitivity analyses

Section and Topic	Item #	Checklist item	Location where item is reported
	20c	Present results of all investigations of possible causes of heterogeneity among study results.	Manuscript: Results<br>Appendix 16: Synthesis without meta-analysis, meta-analyses, meta-regression, subgroup, and sensitivity analyses<br>Appendix 17: Assessment of publication bias/small study effects
	20d	Present results of all sensitivity analyses conducted to assess the robustness of the synthesized results.	Manuscript: Results<br>Appendix 16: Synthesis without meta-analysis, meta-analyses, meta-regression, subgroup, and sensitivity analyses
Reporting biases	21	Present assessments of risk of bias due to missing results (arising from reporting biases) for each synthesis assessed.	Manuscript: Results<br>Appendix 13: Risk of bias domain and overall grade for included datapoints and studies<br>Appendix 18: Summary of findings and certainty of the evidence
Certainty of evidence	22	Present assessments of certainty (or confidence) in the body of evidence for each outcome assessed.	Manuscript: Certainty of evidence<br>Appendix 18: Summary of findings and certainty of the evidence
**DISCUSSION**			
Discussion	23a	Provide a general interpretation of the results in the context of other evidence.	Manuscript: Discussion
	23b	Discuss any limitations of the evidence included in the review.	Manuscript: Discussion
	23c	Discuss any limitations of the review processes used.	Manuscript: Discussion
	23d	Discuss implications of the results for practice, policy, and future research.	Manuscript: Discussion
**OTHER INFORMATION**			
Registration and protocol	24a	Provide registration information for the review, including register name and registration number, or state that the review was not registered.	Manuscript: Methods<br>Appendix 20: Registered/published protocols
	24b	Indicate where the review protocol can be accessed, or state that a protocol was not prepared.	Manuscript: Methods<br>Appendix 20: Registered/published protocols
	24c	Describe and explain any amendments to information provided at registration or in the protocol.	Manuscript: Methods<br>Appendix 2: Deviations from protocol
Support	25	Describe sources of financial or non-financial support for the review, and the role of the funders or sponsors in the review.	Manuscript: Funding
Competing interests	26	Declare any competing interests of review authors.	Manuscript: Competing interests
Availability of data,	27	Report which of the following are publicly available and where they can be found: template data collection forms; data extracted from	Manuscript: Data sharing

Section and Topic	Item #	Checklist item	Location where item is reported
code and other materials		included studies; data used for all analyses; analytic code; any other materials used in the review.	

Table B. PRISMA 2020 structured abstract checklist

Section and Topic	Item #	Checklist item	Reported (Yes/No)
**TITLE**			
Title	1	Identify the report as a systematic review.	Yes
**BACKGROUND**			
Objectives	2	Provide an explicit statement of the main objective(s) or question(s) the review addresses.	Yes
**METHODS**			
Eligibility criteria	3	Specify the inclusion and exclusion criteria for the review.	Yes
Information sources	4	Specify the information sources (e.g. databases, registers) used to identify studies and the date when each was last searched.	Yes
Risk of bias	5	Specify the methods used to assess risk of bias in the included studies.	Yes
Synthesis of results	6	Specify the methods used to present and synthesise results.	Yes
**RESULTS**			
Included studies	7	Give the total number of included studies and participants and summarise relevant characteristics of studies.	Yes
Synthesis of results	8	Present results for main outcomes, preferably indicating the number of included studies and participants for each. If meta-analysis was done, report the summary estimate and confidence/credible interval. If comparing groups, indicate the direction of the effect (i.e. which group is favoured).	Yes
**DISCUSSION**			
Limitations of evidence	9	Provide a brief summary of the limitations of the evidence included in the review (e.g. study risk of bias, inconsistency and imprecision).	Yes
Interpretation	10	Provide a general interpretation of the results and important implications.	Yes
**OTHER**			
Funding	11	Specify the primary source of funding for the review.	Yes
Registration	12	Provide the register name and registration number.	Yes

Appendix 20. Registered/published protocols

University of Glasgow published protocol

https://www.gla.ac.uk/media/Media_718614_smxx.pdf

PROSPERO registered and updated protocol

PROSPERO ID: CRD42020179766
https://www.crd.york.ac.uk/prospero/display_record.php?ID=CRD42020179766

References

1. Schünemann HJ, Vist GE, Higgins JPT, Santesso N, Deeks JJ, Glasziou P, et al. Chapter 15: Interpreting results and drawing conclusions. In: Higgins J, Thomas J, Chandle J, Cumpston M, Li T, Page M, et al., editors. Cochrane Handbook for Systematic Reviews of Interventions version 62 (updated February 2021). Cochrane; 2021
2. Mathur MB, Vanderweele TJ. A simple, interpretable conversion from Pearson's correlation to Cohen's d for continuous exposures. Epidemiology. 2020;31(2):16–8. DOI:10.1097/EDE.0000000000001105
3. Sloan L, Quan-Haase A. The SAGE Handbook of Social Media Research Methods. London: SAGE Publications Ltd; 2017. 17 p.
4. Parke J, Wardle J, Rigbye J, Parke A. Exploring social gambling: Scoping, classification and evidence review. Birmingham, UK; 2012.
5. Aburahmah L, Al Rawi H, Izz Y, Syed L. Online social gaming and social networking sites. Procedia Comput Sci. 2016;82:72–9. DOI:10.1016/J.PROCS.2016.04.011
6. Kaakinen M, Sirola A, Savolainen I, Oksanen A. Young people and gambling content in social media: An experimental insight. Drug Alcohol Rev. 2020;39(2):152–61. DOI:10.1111/DAR.13010
7. Trifiro BM, Gerson J. Social media usage patterns: Research note regarding the lack of universal validated measures for active and passive use. Soc Media + Soc. 2019;5(2). DOI:10.1177/2056305119848743
8. Boyd DM, Ellison NB. Social network sites: definition, history, and scholarship. J Comput Commun. 2007;13(1):210–30. DOI:10.1111/J.1083-6101.2007.00393.X
9. Quercia D, Lathia N, Calabrese F, Lorenzo G, Crowcroft J. Recommending social events from mobile phone location data. 2010 IEEE Int Conf Data Min. 2010;971–6. DOI:10.1109/ICDM.2010.152
10. Millen DR, Yang M, Whittaker S, Feinberg J. Social bookmarking and exploratory research. In: ECSW'07: Proceedings of the 10th European Conference on Computer-supported Cooperative Work. London: Springer; 2007.
11. Archambault PM, van de Belt TH, Grajales FJ, Faber MJ, Kuziemsky CE, Gagnon S, et al. Wikis and collaborative writing applications in health care: A scoping review. J Med Internet Res. 2013;15(10):e210. DOI:10.2196/JMIR.2787.
12. Techopedia. Dictionary: Web conferencing [Internet]. Techopedia. 2021. Available from: Available from: https://www.techopedia.com/definition/16054/web-conferencing [accessed 2021 04 June]
13. Internet Matters. Online gaming- The basics [Internet]. 2021. Available from: https://www.internetmatters.org/resources/online-gaming-advice/the-basics/ [accessed 2021 04 June]
14. Guyatt GH, Oxman AD, Schünemann HJ, Tugwell P, Knottnerus A. GRADE guidelines: A new series of articles in the Journal of Clinical Epidemiology. J Clin Epidemiol. 2011;64(4):380–2. DOI:10.1016/J.JCLINEPI.2010.09.011
15. Thomas J, Kneale D, McKenzie JE, Brennan SE, Bhaumik S. Chapter 2: Determining the scope of the review and the questions it will address. In: Higgins J, Thomas J, Chandler J, Cumpston M, Li T, Page M, et al., editors. Cochrane Handbook for Systematic Reviews of Interventions version 62 (updated February 2021). Cochrane; 2021.

16. Stoff DM, Breiling J, Maser JD. Handbook of Anti-social Behaviour. NJ, USA: Wiley; 1997.
17. Steiner WG. Drug use [Internet]. Encyclopaedia Britanica. 2020. Available from: https://www.britannica.com/topic/druguse [accessed 2021 04 June]
18. Hilton MJ, Ann Rose C, Sweanor DT, Henningfield J. Smoking [Internet]. Encyclopaedia Britanica. 2020. Available from: https://www.britannica.com/topic/smoking-tobacco [accessed 2021 04 June]
19. U.S. Food & Drug Administration. Vaporizers, e-cigarettes, and other electronic nicotine delivery systems (ENDS) [Internet]. 2020. Available from: https://www.fda.gov/tobacco-products/products-ingredients-components/vaporizers-e-cigarettes-and-other-electronic-nicotine-delivery-systems-ends [accessed 2021 04 June]
20. Chawla N, Sarkar S. Defining "High-risk sexual behavior" in the context of substance use. J Psychosexual Heal. 2019;1(1):26–31. DOI:10.1177/2631831818822015
21. Wilber MK, Potenza MN. Adolescent gambling: Research and clinical implications. Psychiatry. 2006;3(10):40–8.
22. Stok FM, Renner B, Allan J, Boeing H, Ensenauer R, Issanchou S, et al. Dietary behavior: An interdisciplinary conceptual analysis and taxonomy. Front Psychol. 2018;9(SEP):1–12. DOI:10.3389/FPSYG.2018.01689
23. Bull FC, Armstrong TP, Dixon T, Ham S, Neiman A, Pratt M. Physical inactivity. In: Comparative quantification of health risks Global and regional burden of disease attributal to selected major risk factors. Geneva: World Health Organization; 2004. p. 729–881.
24. Higgins JPT, Eldridge S, Li T. Chapter 23: Including variants on randomized trials. In: Higgins J, Thomas J, Chandler J, Cumpston M, Li T, Page M, et al., editors. Cochrane Handbook for Systematic Reviews of Interventions version 6.2 (updated February 2021). Cochrane; 2021.
25. McKenzie JE, Brennan SE. Chapter 12: Synthezing and presenting findings using other methods. In: Higgins J, Thomas J, Chandler J, Cumpston M, Li T, Page M, et al., editors. Cochrane Handbook for Systematic Reviews of Interventions version 6.2. Cochrane; 2021.
26. Boon MH, Thomson H. The effect direction plot revisited: Application of the 2019 Cochrane Handbook guidance on alternative synthesis methods. Res Synth Methods. 2021;12(1):29–33. DOI:10.1002/JRSM.1458
27. Wells G, Shea B, O'Connell D, Peterson J. The Newcastle-Ottawa Scale (NOS) for assessing the quality of nonrandomised studies in meta-analyses [Internet]. Ottawa: The Ottawa Hospital Research Institute; 2000. Available from: http://www.ohri.ca/programs/clinical_epidemiology/oxford.asp [accessed January 05, 2020]
28. Higgins JPT, Li T, Deeks JJ. Chapter 6: Choosing effect measures and computing estimates of effect. In: Higgins J, Thomas J, Chandler J, Cumpston M, Li T, Page M, et al., editors. Cochrane Handbook for Systematic Reviews of Interventions version 6.2 (updated February 2021). Cochrane; 2021.
29. Deeks JJ, Higgins JPT, Altman DG. Chapter 10: Analysing data and undertaking meta-analyses. In: Higgins JPT, Thomas J, Chandler J, Cumpston M, Li T, Page MJ WV, editor. Cochrane Handbook for Systematic Reviews of Interventions version 6.2 (updated February 2021). Cochrane; 2021.
30. Wilson DB. Practical meta-analysis effect size calculator [Online calculator] [Internet]. Available from: https://campbellcollaboration.org/research-resources/effect-size-calculator.html [accessed 30 September,

2020]

31. Schünemann H, Oxman A, Vist G, et al. Chapter 12: Interpreting results and drawing conclusions. In: Higgins G, Green S, editors. Cochrane Handbook for Systematic Reviews of Interventions version 5.1.0. Cochrane; 2011.

32. Anastario M, FireMoon P, Ricker A, Holder S, Rink E. Self-reported exposure to sexual and reproductive health information among American Indian youth: Implications for technology based intervention. J Health Commun. 2020;25(5):412–20. DOI:10.1080/10810730.2020.1777599

33. Baker T, Pelfrey WV. Bullying victimization, social network usage, and delinquent coping in a sample of urban youth: Examining the predictions of General Strain Theory. Violence Vict. 2016;31(6):1021–43. DOI:10.1891/0886-6708.VV-D-14-00154

34. Baldwin H, Freeman B, Kelly B. Like and share: Associations between social media engagement and dietary choices in children. Public Health Nutr. 2018;21(17):3210–5. DOI:10.1017/S1368980018001866

35. Ball J, Zhang J, Stanley J, Waa A, Gurram N, Edwards R, et al. Has increasing internet use due to smartphone uptake contributed to the decline in adolescent smoking? Drug Alcohol Rev. 2022;41(2):365–76. DOI:10.1111/DAR.13378

36. Baru A, Adeoye IA, Adekunle AO. Risky sexual behavior and associated factors among sexually-active unmarried young female internal migrants working in Burayu Town, Ethiopia. PLoS One. 2020;15(10):e0240695. DOI:10.1371/JOURNAL.PONE.0240695

37. Baumgartner SE, Sumter SR, Peter J, Valkenburg PM. Identifying teens at risk: Developmental pathways of online and offline sexual risk behavior. Pediatrics. 2012;130(6):e1489–96. DOI:10.1542/PEDS.2012-0842

38. Bayraktar F, Gun Z. Incidence and correlates of internet usage among adolescents in North Cyprus. Cyberpsychology Behav. 2007;10(2):191–7. DOI:10.1089/CPB.2006.9969

39. Beebe TJ, Asche SE, Harrison PA, Quinlan KB. Heightened vulnerability and increased risk-taking among adolescent chat room users: Results from a statewide school survey. J Adolesc Heal. 2004;35(2):116–23. DOI:10.1016/S1054-139X(03)00528-7

40. Boers E, Afzali M, Conrod P. A longitudinal study on the relationship between screen time and adolescent alcohol use: The mediating role of social norms. Prev Med (Baltim). 2020;132(PG-105992-105992):105992. DOI:10.1016/J.YPMED.2020.105992

41. Boniel-Nissim M, van den Eijnden RJJM, Furstova J, Marino C, Lahti H, Inchley J, et al. International perspectives on social media use among adolescents: Implications for mental and social well-being and substance use. Comput Human Behav. 2022;129. DOI:10.1016/J.CHB.2021.107144

42. Booker CL, Skew AJ, Kelly YJ, Sacker A. Media use, sports participation, and well-being in adolescence: Cross-sectional findings from the UK Household Longitudinal Study. Am J Public Health. 2015;105(1 PG-173–179):173–9. DOI:10.2105/AJPH.2013. 301783

43. Brunborg GS, Burdzovic Andreas J. Increase in time spent on social media is associated with modest increase in depression, conduct problems, and episodic heavy drinking. J Adolesc. 2019;74:201–9. DOI:10.1016/J.ADOLESCENCE.2019.06.013

44. Brunborg G, Skogen J, Burdzovic Andreas J. Time spent on social media and alcohol use among

adolescents: A longitudinal study. Addict Behav. 2022;130:107294. DOI:10.1016/J.ADDBEH.2022.107294

45. Camenga D, Gutierrez KM, Kong G, Cavallo D, Simon P, Krishnan-Sarin S. E-cigarette advertising exposure in e-cigarette naive adolescents and subsequent e-cigarette use: A longitudinal cohort study. Addict Behav. 2018;81:78–83. DOI:10.1016/J.ADDBEH.2018.02.008

46. Canale N, Griffiths MD, Vieno A, Siciliano V, Molinaro S. Impact of internet gambling on problem gambling among adolescents in Italy: Findings from a large-scale nationally representative survey. Comput Human Behav. 2016;57:99–106. DOI:10.1016/J.CHB.2015.12.020

47. Casaló L V, Escario J-J, Ignacio Giménez-Nadal J. Tiempo destinado a Internet por los adolescentes españoles: diferencias según la práctica de deporte y el afecto recibido. Time Devoted to Internet Act by Spanish Adolesc Differ Accord to Pract Sport Affect Receiv. 2022;(177):3–20.

48. Cavazos-Rehg PA, Krauss MJ, Spitznagel EL, Grucza RA, Bierut LJ. Hazards of new media: Youth's exposure to tobacco ads/promotions. Nicotine Tob Res. 2014;16(4):437–44. DOI:10.1093/NTR/NTT168

49. Chang F-C, Chiu C-H, Miao N-F, Chen P-H, Lee C-M, Chiang J-T, et al. Predictors of unwanted exposure to online pornography and online sexual solicitation of youth. J Health Psychol. 2016;21(6):1107–18. DOI:10.1177/1359105314546775

50. Chapin JR. Follow my snaps!: Adolescents' social media use and abuse. Int J Cyber Behav Psychol Learn. 2018;8(3):1–8. DOI:10.4018/IJCBPL.2018070101

51. Chau K, Bhattacherjee A, Senapati A, Guillemin F, Chau N. Association between screen time and cumulating school, behavior, and mental health difficulties in early adolescents: A population-based study. Psychiatry Res. 2022;310:114467. DOI:10.1016/J.PSYCHRES.2022.114467

52. Chen S, Schreurs L, Pabian S, Vandenbosch L. Daredevils on social media: A comprehensive approach toward risky selfie behavior among adolescents. New Media Soc. 2019;21(11–12):2443–62. DOI:10.1177/1461444819850112

53. Coates AE, Hardman CA, Halford JCG, Christiansen P, Boyland EJ. Social media influencer marketing and children's food intake: A randomized trial. Pediatrics. 2019;143(4). DOI:10.1542/PEDS.2018-2554

54. Coyne S, Padilla-Walker L, Day R, Harper J, Stockdale L. A friend request from dear old dad: Associations between parent-child social networking and adolescent outcomes. Cyberpsychol Behav Soc Netw. 2013;17(1):8–13. DOI:10.1089/CYBER.2012.0623

55. Coyne SM, Padilla-Walker LM, Holmgren HG, Stockdale LA. Instagrowth: A longitudinal growth mixture model of social media time use across adolescence. J Res Adolesc. 2018;1–11. DOI:10.1111/JORA.12424

56. Critchlow N, MacKintosh AM, Hooper L, Thomas C, Vohra J. Participation with alcohol marketing and user-created promotion on social media, and the association with higher-risk alcohol consumption and brand identification among adolescents in the UK. Addict Res Theory. 2019;27(6):515–26. DOI:10.1080/16066359.2019.1567715

57. da Costa B, Chaput J, Lopes M, Malheiros L, da Silva I, Silva K. Association between screen time and accelerometer-measured 24-h movement behaviors in a sample of Brazilian adolescents. Public Health. 2021;195:32–8. DOI:10.1016/J.PUHE.2021.03.029

58. Dai L, He Y, Tan Y, Yu Z, Zhu J. Online e-cigarette information exposure and its association with e-cigarette use among adolescents in Shanghai, China. Int J Environ Res Public Health. 2022;19(6):3329. DOI:10.3390/IJERPH19063329

59. Davis J, Pedersen E, Tucker J, Dunbar M, Seelam R, Shih R, et al. Long-term associations between substance use-related media exposure, descriptive norms, and alcohol use from adolescence to young adulthood. Physiol Behav. 2019;48(7 PG-1311–1326):1311–26. DOI:10.1016/J.PHYSBEH.2017.03.040

60. Dawson A, Wymbs B, Evans S, DuPaul G. Exploring how adolescents with ADHD use and interact with technology. J Adolesc. 2019;71:119–37. DOI:10.1016/J.ADOLESCENCE.2019.01.004

61. de Bruijn A, Engels R, Anderson P, Bujalski M, Gosselt J, Schreckenberg D, et al. Exposure to online alcohol marketing and adolescents' drinking: A cross-sectional study in four European countries. Alcohol Alcohol. 2016;51(5):615–21. DOI:10.1093/ALCALC/AGW020

62. De Jans S, Spielvogel I, Naderer B, Hudders L. Digital food marketing to children: How an influencer's lifestyle can stimulate healthy food choices among children. Appetite. 2021;162(June 2020):105182. DOI:10.1016/J.APPET.2021.105182

63. De Looze M, van Dorsselaer S, Stevens G, Boniel-Nissim M, Vieno A, Van den Eijnden R. The decline in adolescent substance use across Europe and North America in the early twenty-first century: A result of the digital revolution? Int J Public Health. 2019;64(2):229–40. DOI:10.1007/S00038-018-1182-7

64. Doornwaard S, Moreno M, van den Eijnden R, Vanwesenbeeck I, Ter Bogt T. Young adolescents' sexual and romantic reference displays on Facebook. J Adolesc Heal. 2014;55(4):535–41. DOI:10.1016/J.JADOHEALTH.2014.04.002

65. Doornwaard S, ter Bogt T, Reitz E, van den Eijnden R. Sex-related online behaviors, perceived peer norms and adolescents' experience with sexual behavior: Testing an integrative model. PLoS One. 2015;10(6):127787. DOI:10.1371/JOURNAL.PONE.0127787

66. Elton-Marshall T, Leatherdale ST, Turner NE. An examination of internet and land-based gambling among adolescents in three Canadian provinces: Results from the Youth Gambling Survey (YGS). BMC Public Health. 2016;16:277. DOI:10.1186/S12889-016-2933-0

67. Erreygers S, Vandebosch H, Vranjes I, Baillien E, De Witte H. Nice or naughty? The role of emotions and digital media use in explaining adolescents' online prosocial and antisocial behavior. Media Psychol. 2017;20(3):374–400. DOI:10.1080/15213269.2016.1200990

68. Floros GD, Siomos K, Fisoun V, Geroukalis D. Adolescent online gambling: The impact of parental practices and correlates with online activities. J Gambl Stud. 2013;29(1):131–50. DOI:10.1007/S10899-011-9291-8

69. Folkvord F, de Bruijne M. The effect of the promotion of vegetables by a social influencer on adolescents' subsequent vegetable intake: A pilot study. Int J Environ Res Public Health. 2020;17(7). DOI:10.3390/IJERPH17072243

70. Froyland LR, Bakken A, von Soest T. Physical fighting and leisure activities among Norwegian adolescents-investigating co-occurring changes from 2015 to 2018. J Youth Adolesc. 2020;49(11):2298–310. DOI:10.1007/S10964-020-01252-8

71. Gascoyne C, Scully M, Wakefield M, Morley B. Food and drink marketing on social media and dietary intake in Australian adolescents: Findings from a cross-sectional survey. Appetite. 2021;166.

DOI:10.1016/J.APPET.2021.105431

72. Gazendam N, Cleverley K, King N, Pickett W, Phillips S. Individual and social determinants of early sexual activity: A study of gender-based differences using the 2018 Canadian Health Behaviour in School-aged Children Study (HBSC). PLoS One. 2020;15:e0238515. DOI:10.1371/JOURNAL.PONE.0238515
73. Geber S, Frey T, Friemel T. Social media use in the context of drinking onset: The mutual influences of social media effects and selectivity. J Health Commun. 2021;26(8):566–75. DOI:10.1080/10810730.2021.1980636
74. Geusens F, Beullens K. Strategic self-presentation or authentic communication? Predicting adolescents' alcohol references on social media. J Stud Alcohol Drugs. 2017;78(1):124–33. DOI:10.15288/JSAD.2017.78.124
75. Geusens F, Beullens K. The reciprocal associations between sharing alcohol references on social networking sites and binge drinking: A longitudinal study among late adolescents. Comput Human Behav. 2017;73:499–506. DOI:10.1016/J.CHB.2017.03.062
76. Geusens F, Vangeel J, Vervoort L, Van Lippevelde W, Beullens K. Disposition-content congruency in adolescents' alcohol-related social media (self-) effects: The role of the five factor model. J Stud Alcohol Drugs. 2019;80:631–40.
77. Gomez P, Feijoo S, Brana T, Varela JJ, Rial A. Minors and online gambling: Prevalence and related variables. J Gambl Stud. 2020;36(3):735–45. DOI:10.1007/S10899-019-09923-3
78. Gordon R, Harris F, Mackintosh A, Moodie C. Assessing the cumulative impact of alcohol marketing on young people's drinking: Cross-sectional data findings. Addict Res Theory. 2011;19(1):66–75. DOI:10.3109/16066351003597142
79. Gregg D, Somers C, Pernice F, Hillman S, Kernsmith. Sexting rates and predictors from an urban midwest high school. J Sch Health. 2018;88(6):423–33. DOI:10.1111/JOSH.12628
80. Gunnlaugsson G, Whitehead TA, Baboudottir FN, Balde A, Jandi Z, Boiro H, et al. Use of digital technology among adolescents attending schools in Bissau, Guinea-Bissau. Int J Environ Res Public Health. 2020;17(23):1–21. DOI:10.3390/IJERPH17238937
81. Hamilton J, Hutchinson E, Evankovich M, Ladouceur C, Silk J. Daily and average associations of physical activity, social media use, and sleep among adolescent girls during the COVID-19 pandemic. J Sleep Res. 2022;1–9. DOI:10.1111/JSR.13611
82. Hayer T, Kalke J, Meyer G, Brosowski T. Do simulated gambling activities predict gambling with real money during adolescence? Empirical findings from a longitudinal study. J Gambl Stud. 2018;34(3):929–47. DOI:10.1007/S10899-018-9755-1
83. Holtz H. Internet use and video gaming predict problem behavior in early adolescence. J Adolesc. 2011;34(1):49–58. DOI:10.1016/J.ADOLESCENCE.2010.02.004
84. Hryhorczuk N, Zvinchuk A, Shkiriak-Nyzhnyk Z, Gonzales N, Hryhorczuk D. Leisure activity and alcohol use among Ukrainian adolescents. Addict Behav Reports. 2019;10:100201. DOI:10.1016/J.ABREP.2019.100201
85. Hrywna M, Bover Manderski MT. Prevalence of electronic cigarette use among adolescents in New Jersey and association with social factors. JAMA Netw Open. 2020;3(2):e1920961.

DOI:10.1001/JAMANETWORKOPEN.2019.20961
86. Huang GC, Okamoto J, Valente TW, Sun P, Wei Y, Johnson CA, et al. Effects of media and social standing on smoking behaviors among adolescents in china. J Child Media. 2012;6(1):100–18. DOI:10.1080/17482798.2011.633411
87. Huang GC, Unger JB, Soto D, Fujimoto K, Pentz MA, Jordan-Marsh M, et al. Peer influences: The impact of online and offline friendship networks on adolescent smoking and alcohol use. J Adolesc Heal. 2014;54(5):508–14. DOI:10.1016/J.JADOHEALTH.2013.07.001
88. Jeong H, Shin K. How does adolescents' usage of social media affect their dietary satisfaction? Int J Environ Res Public Health. 2022;19(6). DOI:10.3390/IJERPH19063621
89. Jiang Q, Huang X, Tao R. Examining factors influencing internet addiction and adolescent risk behaviors among excessive internet users. Health Commun. 2018;33(12):1434–44. DOI:10.1080/10410236.2017.1358241
90. Kaufman ZA, Braunschweig EN, Feeney J, Dringus S, Weiss H, Delany-Moretlwe S, et al. Sexual risk behavior, alcohol use, and social media use among secondary school students in informal settlements in Cape Town and Port Elizabeth, South Africa. AIDS Behav. 2014;18(9):1661–74. DOI:10.1007/S10461-014-0816-X
91. Kaur N, Rutherford C, Martins S, Keyes K. Associations between digital technology and substance use among US adolescents: Results from the 2018 Monitoring the Future survey. Drug Alcohol Depend. 2020;213(108124). DOI:10.1016/J.DRUGALCDEP.2020.108124
92. Kelleghan AR, Leventhal AM, Cruz TB, Bello MS, Liu F, Unger JB, et al. Digital media use and subsequent cannabis and tobacco product use initiation among adolescents. Drug Alcohol Depend. 2020;212:108017. DOI:10.1016/J.DRUGALCDEP.2020.108017
93. King DL, Delfabbro PH, Kaptsis D, Zwaans T. Adolescent simulated gambling via digital and social media: An emerging problem. Comput Human Behav. 2014;31:305–13. DOI:10.1016/J.CHB.2013.10.048
94. Ko C-H, Yen J-Y, Liu S-C, Huang C-F, Yen C-F. The associations between aggressive behaviors and internet addiction and online activities in adolescents. J Adolesc Heal. 2009;44(6):598–605. DOI:10.1016/J.JADOHEALTH.2008.11.011
95. Kontostoli E, Jones AP, Pearson N, Foley L, Biddle SJH, Atkin AJ. The association of contemporary screen behaviours with physical activity, sedentary behaviour and sleep in adolescents: a cross-sectional analysis of the Millennium Cohort Study. Int J Behav Med. 2022; DOI:10.1007/S12529-022-10077-7
96. Koutamanis M, Vossen HGM, Valkenburg PM. Adolescents' comments in social media: Why do adolescents receive negative feedback and who is most at risk? Comput Human Behav. 2015;53:486–94. DOI:10.1016/J.CHB.2015.07.016
97. Kwon S, Kim R, Lee J-T, Kim J, Song S, Kim S, et al. Association of smartphone use with body image distortion and weight loss behaviors in Korean adolescents. JAMA Netw Open. 2022;5(5):e2213237–e2213237. DOI:10.1001/JAMANETWORKOPEN.2022.13237
98. Landry M, Gonzales F, Wood S, Vyas A. New media use and sexual behavior among Latino adolescents. Am J Health Behav. 2013;37(3):422–30. DOI:10.5993/AJHB.37.3.15
99. Larm P, Åslund C, Nilsson KW. The role of online social network chatting for alcohol use in

adolescence: Testing three peer-related pathways in a Swedish population-based sample. Comput Human Behav. 2017;71:284–90. DOI:10.1016/J.CHB.2017.02.012

100. Larm P, Raninen J, Åslund C, Svensson J, Nilsson KW. The increased trend of non-drinking alcohol among adolescents: What role do internet activities have? Eur J Public Health. 2019;29(1):27–32. DOI:10.1093/EURPUB/CKY168

101. Lee S. Analyzing negative SNS behaviors of elementary and middle school students in Korea. Comput Human Behav. 2015;43:15–27. DOI:10.1016/J.CHB.2014.10.014

102. Lee S, Han D-H, Chow A, Seo D-C. A prospective longitudinal relation between elevated use of electronic devices and use of electronic nicotine delivery systems. Addict Behav. 2019;98:106063. DOI:10.1016/J.ADDBEH.2019.106063

103. Lee P, Tse A, Wu C, Mak Y, Lee U. Temporal association between objectively measured smartphone usage, sleep quality and physical activity among Chinese adolescents and young adults. J Sleep Res. 2021;30(4):e13213. DOI:10.1111/JSR.13213

104. Lee J, Rattay K, Henry L, Killingsworth R, Tan A, Porter L, et al. Association between social media use and vaping among Florida adolescents, 2019. Prev Chronic Dis. 2021;18(2):E49. DOI:10.5888/PCD18.200550

105. Lin E, Caswell S, You RQ, Huckle T. Engagement with alcohol marketing and early brand allegiance in relation to early years of drinking. Addict Res Theory. 2012;20(4):329–38. DOI:10.3109/16066359.2011.632699

106. Lipsky L, Nansel T, Haynie D, Liu D, Li K, Pratt C, et al. Diet quality of US adolescents during the transition to adulthood: Changes and predictors. Am J Clin Nutr. 2017;105(6):1424–32. DOI:10.3945/AJCN.116.150029

107. Longobardi C, Fabris MA, Prino LE, Settanni M. The role of body image concerns in online sexual victimization among female adolescents: The mediating effect of risky online behaviors. J Child Adolesc Trauma. 2021;14(1):51–60. DOI:10.1007/S40653-020-00301-5

108. McClure AC, Gabrielli J, Cukier S, Jackson KM, Brennan ZLB, Tanski SE. Internet alcohol marketing recall and drinking in underage adolescents. Acad Pediatr. 2020;20(1):128–35. DOI:10.1016/J.ACAP.2019.08.003

109. Merrill RA, Liang X. Associations between adolescent media use, mental health, and risky sexual behaviors. Child Youth Serv Rev. 2019;103:1–9. DOI:10.1016/J.CHILDYOUTH.2019.05.022

110. Michael T. Social media and sexual reproductive health behaviour among adolescents in Bayelsa State, Nigeria. Am Int J Res Humanit Arts Soc Sci. 2016;14(2):94–8.

111. Moitra P, Madan J. Impact of screen time during COVID-19 on eating habits, physical activity, sleep, and depression symptoms: A cross-sectional study in Indian adolescents. PLoS One. 2022;17:e0264951. DOI:10.1371/JOURNAL.PONE.0264951

112. Mojica C, Parra-Medina D, Yin Z, Akopian D, Esparza L. Assessing media access and use among Latina adolescents to inform development of a physical activity promotion intervention incorporating text messaging. Health Promot Pract. 2014;15(4):548–55. DOI:10.1177/1524839913514441

113. Molla-Esparza C, López-González E, Losilla J. Sexting prevalence and socio-demographic correlates in Spanish secondary school students. Sex Res Soc Policy. 2021;18(1):97–111. DOI:10.0.3.239/S13178-

020-00434-0

114. Nesi J, Rothenberg WA, Hussong AM, Jackson KM. Friends' alcohol-related social networking site activity predicts escalations in adolescent drinking: Mediation by peer norms. J Adolesc Heal. 2017;60(6):641–7. DOI:10.1016/J.JADOHEALTH.2017.01.009

115. Nesi J, Prinstein M. In search of likes: Longitudinal associations between adolescents' digital status seeking and health-risk behaviors. J Clin Child Adolesc Psychol. 2019;48:740–8. DOI:10.1080/15374416.2018.1437733

116. Ng Fat L, Cable N, Kelly Y. Associations between social media usage and alcohol use among youths and young adults: Findings from Understanding Society. Addiction. 2021;116(11):2995–3005. DOI:10.1111/ADD.15482

117. Ngqangashe Y, Backer C. The differential effects of viewing short-form online culinary videos of fruits and vegetables versus sweet snacks on adolescents' appetites. Appetite. 2021;166(105436). DOI:10.1016/J.APPET.2021.105436

118. Ohannessian CM. Does technology use moderate the relationship between parental alcoholism and adolescent alcohol and cigarette use? Addict Behav. 2009;34(6/7):606–9. DOI:10.1016/J.ADDBEH.2009.01.001

119. Pegg K, O'Donnell A, Lala G, Barber B. The role of online social identity in the relationship between alcohol-related content on social networking sites and adolescent alcohol use. Cyberpsychol Behav Soc Netw. 2018;21(50–55):50–5. DOI:10.1089/CYBER.2016.0665

120. Adriana P, Spells C, Bluestein M, Harrell M, Emily T, Pérez A. The longitudinal impact of seeing and posting tobacco-related social media on tobacco use behaviors among youth (aged 12-17): Findings from the 2014-2016 Population Assessment of Tobacco and Health (PATH) Study. Tob Use Insights. 2022;15:1–7. DOI:10.1177/1179173X221087554

121. Prince MA, Conner BT, Davis SR, Swaim RC, Stanley LR. Risk and protective factors of current opioid use among youth living on or near American Indian reservations: An application of machine learning. Transl Issues Psychol Sci. 2021;7(2):130–40. DOI:10.1037/TPS0000236

122. Qutteina Y, Hallez L, Raedschelders M, De Backer C, Smits T. Food for teens: how social media is associated with adolescent eating outcomes. Public Health Nutr. 2022;25(2):290–302. DOI:10.1017/S1368980021003116

123. Riehm KE, Thrul J, Barrington-Trimis JL, Kelleghan A, Mojtabai R, Leventhal AM, et al. Prospective association of digital media use with alcohol use initiation and progression among adolescents. Alcohol Clin Exp Res. 2021;45(4):877–85. DOI:10.1111/ACER.14578

124. Roditis M, Delucchi K, Chang A, Halpern-Felsher B. Perceptions of social norms and exposure to pro-marijuana messages are associated with adolescent marijuana use. Prev Med (Baltim). 2016;93:171–6. DOI:10.1016/J.YPMED.2016.10.013

125. Romo DL, Garnett C, Younger AP, Stockwell MS, Soren K, Catallozzi M, et al. Social media use and its association with sexual risk and parental monitoring among a primarily Hispanic adolescent population. J Pediatr Adolesc Gynecol. 2017;30(4):466–73. DOI:10.1016/J.JPAG.2017.02.004

126. Rutter LA, Thompson HM, Howard J, Riley TN, De Jesús-Romero RD, Lorenzo-Luaces L. Social media use, physical activity, and internalizing symptoms in adolescence: Cross-sectional analysis. J

Med Internet Res Ment Heal. 2021;8(9):1–14. DOI:10.2196/26134

127. Sampasa-Kanyinga H, Hamilton HA. Use of social networking sites and risk of cyberbullying victimization: A population-level study of adolescents. Cyberpsychol Behav Soc Netw. 2015;18(12):704–10. DOI:10.1089/CYBER.2015.0145

128. Sampasa-Kanyinga H, Chaput J, Hamilton H. Associations between the use of social networking sites and unhealthy eating behaviours and excess body weight in adolescents. Br J Nutr. 2015;114(11):1941–7. DOI:10.1017/S0007114515003566

129. Sampasa-Kanyinga H, Chaput J. Use of social networking sites and alcohol consumption among adolescents. Public Health. 2016;139:88–95. DOI:10.1016/J.PUHE.2016.05.005

130. Sampasa-Kanyinga H, Chaput J. Use of social networking sites and adherence to physical activity and screen time recommendations in adolescents. J Phys Act Health. 2016;13(5):474–80. DOI:10.1123/JPAH.2015-0343

131. Sampasa-Kanyinga H, Hamilton HA. Use of social networking sites, electronic cigarettes, and waterpipes among adolescents. Public Health. 2018;164:99–106. DOI:10.1016/J.PUHE.2018.08.001

132. Sandercock GRH, Alibrahim M. Media device ownership and media use: associations with sedentary time, physical activity and fitness in English youth. Prev Med Reports. 2016;4:162–8. DOI:10.1016/J.PMEDR.2016.05.013

133. Savolainen I, Oksanen A, Kaakinen M, Sirola A, Miller B, Paek H, et al. The association between social media use and hazardous alcohol use among youths: A four-country study. Alcohol Alcohol. 2020;55(1):86–95. DOI:10.1093/ALCALC/AGZ088

134. Self-Brown S, Culbreth R, Wilson R, Armistead L, Kasirye R, Swahn MH, et al. Individual and parental risk factors for sexual exploitation among high-risk youth in Uganda. J Interpers Violence. 2018;00(5–6):1–22. DOI:10.1177/0886260518771685

135. Shan L, Azagba S. Longitudinal associations of tobacco-related social media involvement with cigarette and e-cigarette initiation among US adolescents. Eur J Pediatr. 2022;181(1):189–96. DOI:10.1007/S00431-021-04166-0

136. Sharma AD, Garg S, Singh MM, Deshmukh CP, Sharma P, Borle AL. Prevalence and social contextual factors of smokeless tobacco use: Insights from schools of Delhi, India. Asian Pacific J Cancer Prev. 2021;22(8):2351–5. DOI:10.31557/APJCP.2021.22.8.2351

137. Shimoga SV, Erlyana E, Rebello V. Associations of social media use with physical activity and sleep adequacy among adolescents: Cross-sectional survey. J Med Internet Res. 2019;21(6):e14290. DOI:10.2196/14290

138. Smout A, Chapman C, Mather M, Slade T, Teesson M, Newton N. It's the content that counts: Longitudinal associations between social media use, parental monitoring and alcohol use in an australian sample of adolescents aged 13 to 16 years. Int J Environ Res Public Health. 2021;18(14):7599. DOI:10.3390/IJERPH18147599

139. Soneji S, Yang J, Knutzen K, Moran M, Tan A, Sargent J, et al. Online tobacco marketing and subsequent tobacco use. Pediatrics. 2018;141(2):e20172927. DOI:10.1542/PEDS.2017-2927

140. Stevens R, Gilliard-Matthews S, Dunaev J, Todhunter-Reid A, Brawner B, Stewart J. Social media use and sexual risk reduction behavior among minority youth: Seeking safe sex information. Nurs Res.

2017;66(5):368–77. DOI:10.1097/NNR.0000000000000237

141. Suwanwong C, Kalapat R, Pitayarangsarit S, Chaiyasong S. Factors related to adolescent smoking: A nationally representative cross-sectional study in Thailand. Maced J Med Sci. 2021;9:1267–72. DOI:10.3889/OAMJMS.2021.7453

142. Svensson R, Johnson B. Internet use and adolescent drinking: Does it matter what young people do online? Drug Alcohol Depend. 2020;213:108138. DOI:10.1016/J.DRUGALCDEP.2020.108138

143. Tao X, Fisher CB. Exposure to social media racial discrimination and mental health among adolescents of color. J Youth Adolesc. 2022;51(1):30–44. DOI:10.1007/S10964-021-01514-Z

144. Trangenstein P, Whitehill J, Jenkins M, Jernigan D, Moreno M. Active cannabis marketing and adolescent past-year cannabis use. Drug Alcohol Depend. 2019;204:107548. DOI:10.1016/J.DRUGALCDEP.2019.107548

145. Tsitsika A, Critselis E, Kormas G, Konstantoulaki E, Constantopoulos A, Kafetzis D. Adolescent pornographic internet site use: A multivariate regression analysis of the predictive factors of use and psychosocial implications. Cyberpsychology Behav. 2009;12(5):545–50. DOI:10.1089/CPB.2008.0346

146. Tsitsika A, Critselis E, Janikian M, Kormas G, Kafetzis DA. Association between internet gambling and problematic internet use among adolescents. J Gambl Stud. 2011;27(3):389–400. DOI:10.1007/S10899-010-9223-Z

147. Vandenbosch L, Beyens I, Vangeel L, Eggermont S. Online communication predicts Belgian adolescents' initiation of romantic and sexual activity. Eur J Pediatr. 2016;175(4):509–16. DOI:10.1007/S00431-015-2666-6

148. Vannucci A, Ohannessian C. Social media use subgroups differentially predict psychosocial well-being during early adolescence. J Youth Adolesc. 2019;48(8):1469–93. DOI:10.1007/S10964-019-01060-9

149. Vazquez-Nava F, Vazquez-Rodriguez E, Vazquez-Rodriguez C, Ortega Betancourt N, Castillo Ruiz O, Rodriguez Castillejos G. Tobacco smoking by Mexican adolescents who use the WhatsApp and Facebook social networking platforms as a means of communication. J Subst Use. 2020;25(5):545–9. DOI:10.1080/14659891.2020.1738576

150. Vente T, Daley M, Killmeyer E, Grubb LK. Association of social media use and high-risk behaviors in adolescents: Cross-sectional study. J Med Internet Res Pediatr Parent. 2020;3(1):1–10. DOI:10.2196/18043

151. Wana G, Arulogun O, Roberts A, Kebede A. Predictors of risky sexual behaviour among pre-college students in Adama Town, Ethiopia. Pan Afr Med J. 2019;33(135–135):135. DOI:10.11604/PAMJ.2019.33.135.18068

152. Ward RM, Dumas TM, Lewis MA, Litt DM. Likelihood of posting alcohol-related content on social networking sites - measurement development and initial validation. Subst Use Misuse. 2022;57(7):1111–9. DOI:10.1080/10826084.2022.2064505

153. Whitehill JM, Trangenstein PJ, Jenkins MC, Jernigan DH, Moreno MA. Exposure to cannabis marketing in social and traditional media and past-year use among adolescents in states with legal retail cannabis. J Adolesc Heal. 2020;66(2):247–54. DOI:10.1016/J.JADOHEALTH.2019.08.024

154. Widman L, Nesi J, Choukas-Bradley S, Prinstein MJ. Safe sext: Adolescents' use of technology to communicate about sexual health with dating partners. J Adolesc Heal. 2014;54(5):612–4.

DOI:10.1016/J.JADOHEALTH.2013.12.009

155. Wulff H, Duan Y, Wagner P. Physical activity and social network use of adolescents in overweight and obesity treatment. Int J Environ Res Public Health. 2021;18(13):6938. DOI:10.3390/IJERPH18136938

156. Yao L, Sun X, Niu G, Zheng Y, Chinyani T. Parental mediation moderates the association between social media exposure and tobacco and alcohol use: Differences between elementary and middle school students. J Stud Alcohol Drugs. 2022;83(2):267–75. DOI:10.15288/JSAD.2022.83.267

157. Lenhart A. Teens, social media & technology overview 2015. Pew Research Center's Internet & American Life Project. 2015.

158. Mikami AY, Szwedo DE. UBC Facebook coding manual. (Unpublished manual). 2013.

159. Moreno M, Egan K, Brockman L. Development of a researcher codebook for use in evaluating social networking site profiles. J Adolesc Heal. 2011;49(1):29–35. DOI:10.1016/J.JADOHEALTH.2011.04.015

160. World Bank. World bank country and lending groups country classification [Internet]. [Online]. [Accessed 03 March, 2021]; 2021.

161. Ragelienė T, Grønhøj A. The role of peers, siblings and social media for children's healthy eating socialization: A mixed methods study. Food Qual Prefer. 2021;93(June 2020). DOI:10.1016/J.FOODQUAL.2021.104255
